# Effects of *Cucurbita Moschata* squash (Butternut) seed paste in improving zinc and iron status in children attending Early Childhood Development centres in Limpopo province, South Africa

**DOI:** 10.1371/journal.pone.0300845

**Published:** 2024-04-18

**Authors:** Selekane Ananias Motadi, Xikombiso Gertrude Mbhenyane, Mthokozisi Kwazi Zuma, Jeanne H. Freeland Graves

**Affiliations:** 1 Division of Human Nutrition, Faculty of Medicine and Health Sciences, Stellenbosch University, Cape Town, South Africa; 2 Department of Nutrition, Faculty of Health Sciences, University of Venda, Thohoyandou, South Africa; 3 Smallholder Agricultural Development, Agricultural Research Council, Pretoria, South Africa; 4 Nutritional Sciences, University of Texas at Austin, Austin, Texas, United States of America; University of Rwanda College of Medicine and Health Sciences, RWANDA

## Abstract

*Cucurbita moschata* (Butternut squash) seeds are a rich source of nutrition containing nutrients including iron, zinc, copper, calcium, potassium, and phosphorus. The aim of this study was to determine if Cucurbita Moschata squash seed paste improves zinc and iron status, anthropometric status, and dietary intake in preschool children. A pretest-posttest control group trial using cluster randomisation was conducted over 6 months. Four preschools were randomly assigned to receive 100 g of intervention or 100 g of a placebo as the control to enhance iron and zinc status. A total of 276 preschool children were recruited from eight government registered Early Childhood Development centres in Limpopo province, South Africa. The control group consumed Cucurbita moschata flesh twice-weekly, while the intervention group consumed Cucurbita moschata seed paste twice-weekly during a six-month period. Iron (serum iron, transferrin, transferrin saturation, ferritin) and zinc (serum zinc) status and anthropometric indices such as weight, height and mid upper arm circumference for children were evaluated at baseline and the endpoint. Iron and zinc-rich food consumption was measured using a 24-hour dietary recall and food record during the study, and dietary intake was estimated using a food frequency questionnaire which was conducted at the beginning and endpoint. The intervention group significantly improved in the mean serum iron 0.23 μg/dL (95% CI: 0.11;0.33); ferritin 0.21μg/dL (95% CI: 0.13;0.39), transferrin saturation 0.33% (0.23;0.74) and zinc 0.16 μmol/dl (95% CI: 0.13;0.25) at the end of the study. In addition, the intervention group exhibited greater mean weight for age of 0.13 z-score (95% CI: 0.28; 0.34) and weight for height of 0.04 z-score (95% CI: 0.12,0.05), as well as the consumption of iron (p < 0,001), zinc (p < 0,001), and vitamin C (p < 0.001). At the end of the trial, fiber (p < 0.001), riboflavin (p = 0.001), vitamin B6 (p < 0.001), and vitamin B12 (p < 0.001) were significantly higher in the control group. Thus, the inclusion of intervention in the diet of children in an impoverished area of South Africa improved the iron and zinc status of these children. This supplement could be a cost effective and sustainable approach to improve nutrient status in rural South Africa.

**Trial registration**: Pan African Clinical Trial Registry (PACTR202308740458863).

## Introduction

Deficiencies of iron and zinc often occur as part of a malnutrition cycle [1]. Iron deficiency and iron deficiency anaemia are two of the most common nutritional deficiencies among children in the world [2–4]. The WHO estimates that iron deficiency anaemia affects a quarter of the global population and is more prevalent in pre-school children, particularly in Africa [5]. In addition, a deficiency of zinc is likely to occur where iron deficiency persists because these minerals are often found in the same food sources [3]. It is estimated that zinc deficiency affects approximately one-third of the world’s population, with a prevalence of 4% to 73% across regions in Sub Saharan Africa [6]. Children are the most risk group for zinc deficiency. In 2015, Motadi et al. [7] reported iron deficiency anaemia prevalence rates of 28%, and zinc deficiency prevalence rates of 43% among 3- to 5-year-old children from Limpopo province. In 2023, Motadi et al. found that almost all (99%) the children were iron deficient while no child was found to have zinc deficiency [8].

Iron and zinc are interrelated, as zinc is a cofactor for numerous enzymes involved in iron metabolism [3]. For example, it serves as a cofactor for the synthesis of the heme portion of hemoglobin; consequently, severely deficient zinc diets may result in anaemia [9, 10]. A common reason for deficiencies of iron and zinc are consumption of staple foods and cereal crops that have low micronutrient bioavailability [11]. When iron and zinc are lacking in the diet, growth is impeded, immune function is depressed, hair loss can occur, and susceptibility to and severity of infections is increased [3, 12]. In addition, neurobehavioral abnormalities may develop due to increased number of opiate receptors in the hypothalamus [3].

One strategy to improve micronutrient status is food fortification. Since 2003 staple foods such as wheat bread flour and maize meal have been fortified with iron, zinc, folic acid, niacin, thiamine, riboflavin, pyridoxine, and vitamin A in South Africa [13]. This alone has not proven successful in mitigating micronutrient deficiencies. Another strategy is using a food-based approach [14–16]. Utilizing locally accessible crops to improve the nutrient content of the diet is a safe and cost-effective food-based strategy [16]. Rural communities have been consuming traditional vegetables and their seeds for centuries. These foods have improved household food security by providing direct access to readily accessible nutrients [17, 18]. Some of the most commonly consumed foods in Africa are squashes and pumpkins. Currently, *Cucurbita Moschata* squash (commonly known as Butternut) seeds are discarded as by-products in food processing, despite nutritional potential [19, 20]. After the flesh is removed, the best seeds are often set aside for cultivation the next season. Some communities around the world (China, South Africa, Netherlands, Austria, Paraguay, Canada, Burkina Faso, Germany, and Spain) consume the seeds raw, roasted, or cooked, but only at a domestic scale [19]. Previously, numerous communities in South Africa consumed roasted *Cucurbita Moschata* squash seeds with a maize meal porridge; however, this practice has diminished as it is associated with poverty [18, 21, 22].

*Cucurbita Moschata* squash seeds contain minerals such as 9.7 mg iron, 9.7 mg zinc, 1.343 mg copper, 46 mg calcium, 809 mg potassium, and 1233 mg phosphorus per 100 g [11, 20]. It has been proposed that consumption of these seeds could be used to improve micronutrient status and assist with combatting micronutrient deficiencies [23]. At present information has not been available on the effectiveness of *Cucurbita Moschata* seeds for enhancing nutrition in young children. This is the first investigation to analyse the nutrient content of *Cucurbita Moschata* seeds and to examine their efficacy in improving iron and zinc status in children in South Africa. Therefore, this study investigated the potential of *Cucurbita Moschata* seed as a food-based approach to improve iron and zinc status. The aim of this study was to determine efficacy of *Cucurbita Moschata* squash seed paste in improving zinc and iron status, anthropometric status and dietary intake in preschool children in Limpopo, South Africa.

## Materials and methods

### Study design

A pretest-posttest control group trial using cluster randomisation was conducted over 6 months to assess the efficacy of *Cucurbita Moschata* squash seed paste in improving zinc and iron status in preschool children. A cluster randomized control trial was conducted to compare iron and zinc status of children attending Early Childhood Development centres in two treatment groups: intervention and control. Cluster randomization was conducted at the preschool level. The children enrolled in the selected preschools were Mafukani (60), Tshaanda (20), Mukovhawabale (82), Sagole (80), Madimbo (120), Mbodi (48), Bale (48), and Lwathutwa (52). The preschools participating were randomly allocated either to the group receiving the intervention under investigation (seed paste) or to a group receiving a placebo as the control. The most compelling reason to randomize at the cluster, rather than at the individual, level is the potential for contamination. Participants within a cluster are likely to be treated similarly and exhibit similar outcomes. The primary outcome was the zinc and iron status of the intervention group. The key secondary outcome was nutritional status and dietary intakes of the children.

### Study population and recruitment

The study was conducted in eight Early Childhood Development Centers on children, aged 3 to 5 years, from June to November 2021, in the Musina Municipality, Vhembe District of South Africa. The district was selected due to the persistent high rate of food insecurity, hunger, and unemployment, which is close to 24%. According to the Vhembe District Municipality’s Integrated Development Plan of 2021–2022, 70% of the population lives below the food poverty line. This is defined as a monthly income of less than ZAR 561 00 (28 Euro) per person [24].

The researcher and research assistants visited the selected preschools and gave the principals an explanation of the study’s goal and procedure. The meeting with the children’s parents and caregivers was sought after the principal granted permission for the preschool to be included in the study. The research goals and procedures were presented to the children’s parents and caregivers during the meeting. During parents’ meeting, children were recruited. The recruitment process was conducted over 3 months (01 March to 31 May 2019) to obtain the initial 276 participants. In this rural setting, children attend preschool five days per week and receive two meals per day. These meals are prepared in pre-school kitchens with daily menus, and then consumed in their classrooms. Exclusion criteria were children with (i) physical disabilities that hindered taking anthropometric measurements; (ii) malaria and tuberculosis disease; (iii) taking medications such as antacids, famotidine and ranitidine, omeprazole, pantoprazole, proton pump inhibitors and calcium supplements (these medications alter serum zinc concentration) [25]; or (iv) being severely underweight to the extent of hospitalization. All children received a single dose of Mebendazole (500 mg) for deworming two weeks before the commencement of the study.

### Sample size determination

**Fig 1:**
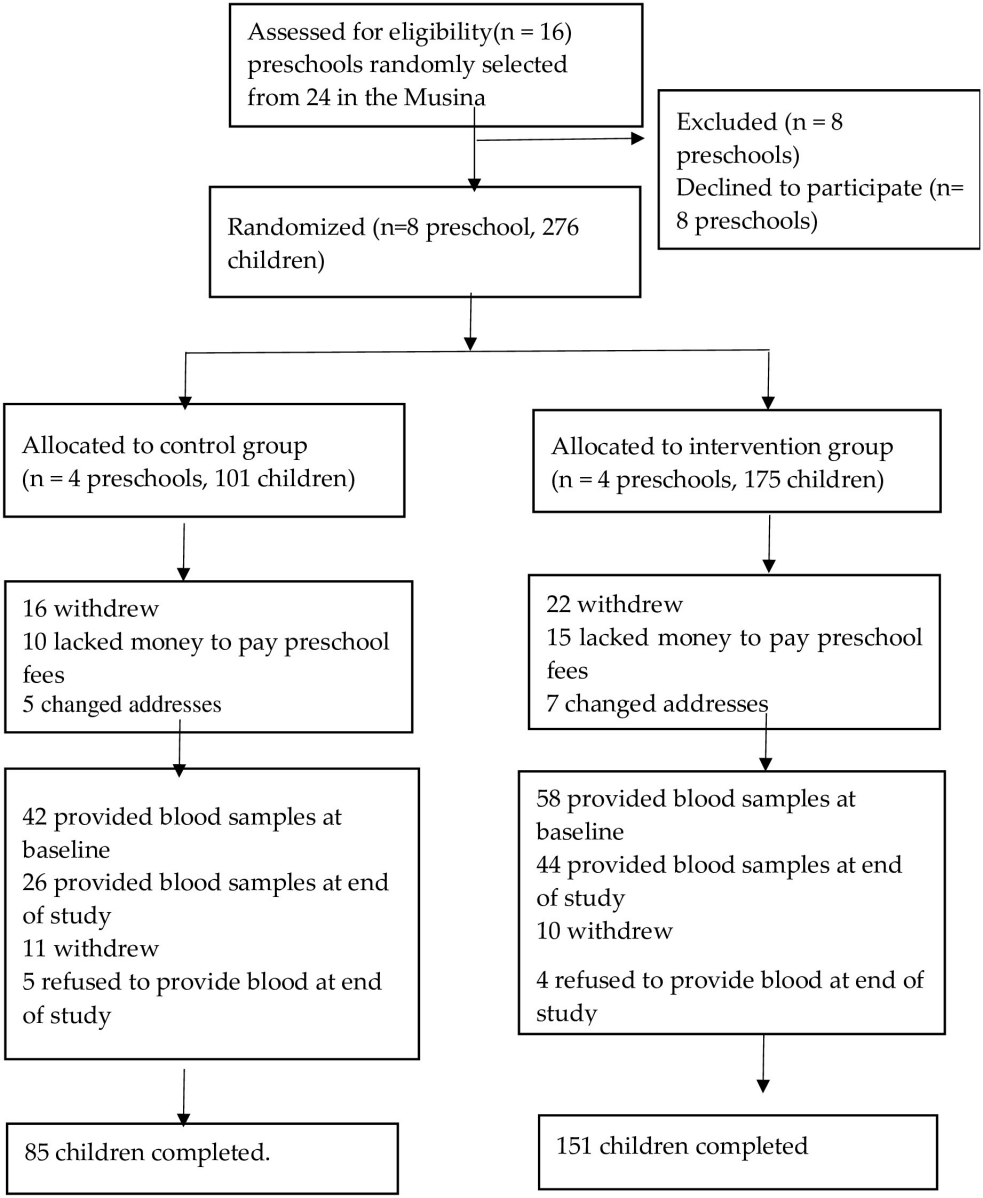
Illustrates the flow chart describing participation of children in the study.

A randomization schedule was created by a statistician who was not associated with the project. A power analysis was performed using two sample t-test. To calculate the minimum sample size, an effect size of 0.49 was assumed (impact of iron-rich supplementary foods on the hemoglobin levels of children 3–36 months), a power of 95%, two-sided alpha of 5%, and allocation ratio of 2:1 in two independent study groups [26, 27]. The minimum sample size was calculated as 206. Assuming a 34% attrition rate, the final sample was 276. However, the sample sizes for the control and experiment differed due to the variations in enrolment at the Early Childhood Development centre. A random assignment placed all eligible Early Childhood Centers in the intervention or control groups. The controls had 101 participants initially and the intervention, 175; the final numbers were 85 and 151, respectively. Fig 1 illustrates the flow chart describing participation of children in the study.

### Preparation of the intervention, and nutrient content

Cucurbita moschata squash was purchased from local farmers in the Musina municipality and Cucurbita moschata seed paste was prepared from it in the food service laboratory of the Division of Human Nutrition at Stellenbosch University for sensory evaluation. Seeds were removed from their cavity using a metal spoon and rinsed with water to remove any residue. The cleaned seeds were roasted in an oven at 135°C for 20 minutes and grounded into a powder using a portable local grinding mill. The water used in the grinding of the seeds did not add iron or zinc. After the six recipes were created, the resultant dishes were assessed for sensory evaluation. A nine-point hedonic scale with verbal anchors (1 = very dislike, 5 = neither like nor dislike, and 9 = extremely like) was used. Other preparation and ingredient-related questions were graded on a category range of 1 to 9 (1 being extremely unlikely, 5 neither likely nor unlikely, and 9 being extremely likely). This scale length was chosen due to its ideal application for hedonic consumer research [28]. Participants indicated their ratings by circling, completing, or checking the appropriate number. Samples were served in consistent portions on small disposable plates. The ingredients used in each dish were listed. Participants completed questions regarding the overall appeal of each recipe and the recipe title, appearance, flavour, and texture. The promoters, and statistician were used as participants of sensory evaluation.

The six different dishes which were developed were tested in the Aspirata Health and Safety Laboratory (Cape Town, South Africa) for microbial activity toxicity, micronutrient content, life span and nutrient stability. The total plate count microbial activity was determined following Codex principles of the Hazard Analysis Critical Control Point. The intervention was examined for a specific pathogen or its toxins (such as coliform for hygiene, yeast, moulds, and fungi) [29]. This examination was used to detect organisms that could be indicative of the possible presence of pathogens or specific spoilage organisms. Testing for microbial activity of the intervention assured that the critical raw materials were satisfactory for their intended use. The six different dishes which were developed from six recipes were given to children during piloting. The children selected the most liked or preferred dish from the six dishes. Only one dish was used for this intervention to allow for control.

During a six-month period, the intervention group consumed Cucurbita moschata seed paste twice-weekly, while the control group consumed Cucurbita moschata flesh twice-weekly. The Cucurbita moschata was chosen as control because in these preschools children consume it twice per week as indicated in their menu. The study altered the Cucurbita moschata by adding the seeds to the recipe and it was given as an intervention. The recommended daily allowance (RDA) for iron for children aged 3 years is 7 mg and for 4 to 5 years is 10 mg per day. The recommended daily allowance for zinc for children aged 3 years is 3 mg; and for those 4 to 5 years, 5 mg per day [3]. The 100 g of control had 1.1 mg of iron and 0.6 mg of zinc, while 100 g of intervention delivered 9.8 mg of iron and 4.1 mg of zinc daily. The cooks and research assistant were given a developed recipe to follow when preparing intervention and control at the preschool sites. The seed paste was consumed during lunch time at the preschools.

### Follow-up and adherence

The seeds were weighed and packed into small plastic bags for each day and delivered to the preschool site every Monday of the week before the actual feeding day. The trained field research assistants (qualified nutritionist) monitored and helped the cooks in preparation of the seed paste for better standardization. The principal investigator visited preschools on different days to monitor quality adherence. Adherence to the intervention was assessed by using a plate waste form on each feeding day at the preschool. Reasons for non-compliance were recorded such as the absence of the child from school due to illness, fear of eating seed paste because it was unfamiliar to them, fear of trying something new.

### Measurements

The following variables were measured at both baseline and endpoint; socio-demographic characteristics, anthropometrics (weight, height, mid upper arm circumference), biochemical measurements of plasma zinc, iron (serum iron, ferritin, transferrin, and transferrin saturation), and nutrient intakes.

### Socio-demographic questionnaire

An interviewer-administered questionnaire was used to collect socio demographic information at pre intervention and post intervention. Data collected included the relationship with the child (to determine whether parent or caregiver), marital status, educational level and employment status of parent/caregiver, household income, whether the child was receiving a grant or other government grant.

### Anthropometry

Anthropometric measurements (weight, height, and mid upper arm circumference) were collected in duplicate, using calibrated equipment. Children wore light clothing without shoes. Height was measured to the nearest 0.1 cm via a portable stadiometer; weight was determined to the nearest 0.01 kg on a portable solar scale (Model 0213) (Seca, Hammer Steindamm, Hamburg, Germany). Mid upper arm circumference was measured using a measuring tape. The solar scale and stadiometer were calibrated prior to use via a calibration weight and steel tape, respectively [30].

Anthropometric indicators were expressed as weight for age (WAZ), height for age (HAZ), weight for height (WHZ), and Z scores. Anthropometric status was interpreted using the World Health Organization (WHO) child growth standards [31, 32]. According to the WHO [31], the Z-score classification cut-off points for underweight, wasting, and stunting were ≤ -2 SD for WAZ, WHZ, and HAZ. Cut-off points ≤ -3 SD were considered as severe underweight, severe wasting and severe stunting. Finally, cut-off points between +2 SD to ≤ +3 S D for WHZ are denoted as overweight; the cut-off point ≥ 3 SD is referred to as obese.

### Dietary data

#### Food frequency questionnaire

The research assistants and fieldworkers administered a food frequency questionnaire (FFQ) to parents/caregivers during the first visit at the beginning and end of the study. This interview included questions about the type, amount, and frequency of food fed to the children. An adapted FFQ from the National Food consumption Survey of 2005 was administered to quantify dietary intake of the children [33]. FFQ was used to assess the full diet and to check if children had consumed food high in iron and zinc. The FFQ included the food items, portion size, and number of times ingested (per day, week, month or never). Parents/caregivers were asked to recall all the food that the child had consumed during the previous 24 hours. Food cards were utilized to assist in remembering the food items and quantities fed to the children. The foods were recorded for each child. To determine the nutrient intake, all dietary data were calculated using the FoodFinder IIITM software program (South African Medical Research Council, South Africa) and a mean daily nutrient intakes and standard deviation were calculated.

#### 24-hour dietary recall

The research assistants and fieldworkers administered a 24-hour dietary recall questionnaire to parents/caregivers at the beginning and end of the study. The field workers used food cards to assist cooks and preschool helpers to remember the food items they fed the children in the previous 24 hours Using the multiple pass method, a one-day, 24-hour recall was conducted by trained field workers to gather comprehensive data about all foods and beverages consumed at the preschool [34]. Six daily intervals were included: breakfast, in-between snack, lunch, in-between snack, dinner, and after dinner snack. The 24-hour recall included portion size, preparation method, ingredients used, time of eating, place where the meal was consumed, and description of food item and snack. The information was used to assess the type of food consumed by the children at the preschool site.

#### Food diary

At the beginning of the study the parents/caregivers were asked to complete a food diary to record the food that their children ate at home to ascertain their children’s consumption of other iron and zinc-rich meals. The information included the food type, the portion size, cooking method used, and time consumed. Over the six months’ period, they were required to record one day per week while ensuring all seven days are included. This was used to monitor if the children have consumed the iron and zinc rich food at home.

The information about the type, amount, and frequency of food fed to the child from the 24-dietary recall and food diary were included in the QFFQ to calculate the dietary intake. The consumption of iron- and zinc-rich foods was reported more than four times per week in both the intervention and control groups using data from the 24-hour dietary recall questionnaire, QFFQ, and food diary.

#### Blood collection

A 5 mL sample of non-fasting venous blood was drawn from the antecubital vein in a subsample of 65 preschool children at baseline and endpoint to determine iron and zinc status. Of these, 39 were in the supplement group and 26, controls. Blood was drawn into trace element-free vacutainer tubes. The serum was separated by centrifugation at 1000 x g for 10 minutes. Portions of serum were frozen immediately and maintained at −70°C until analyzed. Iron was measured by standardized procedures at Lancet laboratories (Polokwane, South Africa) via a STKS analyzer (Beckman Coulter Inc., Brea, CA, USA). Three-level controls were provided by the manufacturer and utilized within 2 h of blood collection. Serum zinc was measured via a 125I-radioimmunoassay. Enzyme-linked immunosorbent assays measured serum transferrin saturation (TSAT) and ferritin (Ramco Laboratories, Inc., Stafford, TX, United States), were analyzed by Synchron LX System(s), UniCel DxC 600/800 System(s) and Synchron Systems CAL 5 Plus by an immunoturbidimetric test (Human Biochemical and Diagnostic Laboratories, South Africa). The coefficients of variance for all assays were <10%.

### Ethical considerations

Ethical clearance was obtained from the Stellenbosch University Health Research Ethics Committee (S18/10/216). The study was approved by the Provincial Department of Education and Health Research Committee. Permissions and access were also received from the selected Early Childhood Development Centers. The study was performed in accordance with the principles of the Declaration of Helsinki 2008, Good Clinical Practices, and laws of South Africa. An oral and written explanation of the study, including possible risks, was provided to the parents/caregivers during a parents/caregivers meeting which was held before the study started. Parents/caregivers were given consent forms to sign for their children to participate Parents/caregivers were given consent forms to sign for their children to participate. In the presence of the researcher, parents were asked to provide second parent to sign as a witness. The witnesses were requested to fill in their contact details as well. There were two signed copies of the consent form. One copy was given to the parents/caregivers, while the second copy was kept in the researcher’s office.

### Statistical analysis

The completed questionnaires were checked for completeness, and anonymity codes were used instead of names; all were entered into Microsoft Excel (version 2016). Data on dietary intakes was also exported from the Food Finder to Microsoft Excel. All data were transferred to the Statistical Package for the Social Sciences (SPSS for Windows version 27, SPSS Inc. Chicago, IL, USA) for analysis. Descriptives such as means, standard deviations (SDs), numbers, and percentages and inferential statistics were generated for variables collected during the study. The normal distribution of the data was tested using the Shapiro-Wilk one-sample test. The analysis of variances (ANOVA) was performed to test the interaction effects between the two treatment groups at baseline and post intervention (endpoint) stage. A linear mixed-effects model was used as an intention to treat analysis for the intervention. The compound symmetry covariance structure was used to take the repeated measures into account and assess the effect of the butternut intervention as the primary analysis. The test for a significant interaction effect between intervention and time (baseline and endpoint) was the overall test for an intervention effect. The estimation for the model parameters was performed via full maximum likelihood. This was the imputation approach used to handle the missing data at the time points post-randomisation to facilitate the intention to treat analysis. The estimated intervention effects at each point considered the baseline difference at randomisation and used the difference-in-difference approach to estimate the mean difference as the intervention effect as well as the 95% confidence interval. Statistical significance was indicated by a p-value of p < 0.05.

## Results

There were slightly more girls than boys in this study, 52% to 48%. A total of 45% of the households had monthly incomes of less than 500 ZAR (25 €), while 23% had between 501 ZAR and 1000 ZAR (25–49 €). Three-quarters of the parents/caregivers of the children were unemployed and 89% had secondary education. More than half of parents/caregivers were single, as opposed to the 39% who were married. The majority of the children (94%) were beneficiaries of a government child grant and only 8% of households received other government grants (i.e., old age) (Table 1).

**Table 1:** Socio-economic characteristics of participating children and their parents/caregivers by group.

Variables	Intervention (n = 151)	Control (n = 85)
	n (%)	n (%)
**Parents/caregivers**		
** Marital status**		
Single	97 (41.1)	43 (18.2)
Married	53 (22.5)	40 (17)
Widowed	1 (0.4)	2 (0.8)
** Education**		
None	4 (1.7)	1 (0.4)
Primary level	12 (5)	8 (3.4)
Secondary level.	135 (57.2)	76 (32.2)
** Employment status**		
Employed	36 (15.3)	22 (9.3)
Unemployed	115 (48.7)	63 (26.7)
** Household income (ZAR) (€)**		
<500 (25 €)	71(30.2)	34 (14.4)
501–1000 (25–49 €)	38 (16.1)	17 (7.2)
1001–2000 (49–99 €)	27 (11.4)	20 (8.5)
2001–5000 (99–248 €)	5 (2.1)	5 (2.1)
5001 –≥ 10000 (248–497 €)	10 (4.2)	9 (3.8)
** Child social government grant**		
Yes	142 (60.2)	79 (33.5)
No	9 (3.8)	6 (2.5)
** Other government grants in the household**		
Old age	6 (2.5)	13 (5.5)
Disability	2 (0.8)	0 (0)
** Child**		
** Age (years)**		
3	32 (13.6)	15 (6.3)
4	119 (50.4)	70 (29.7)
** Sex**		
Boys	67 (28.4)	46 (19.5)
Girls	84 (35.6)	39 (16.5)

The effect of the intervention on the iron and zinc status between the intervention and control groups is presented in Table 2. A significant improvement was observed in the mean serum iron, 0.23 μg/dL (95% CI: 0.11;0.33); ferritin, 0.21 μg/dL (95% CI: 0.13;0.39); transferrin saturation, 0.33% (0.23;0.74); and zinc 0.16 μmol/dl (95% CI: 0.13;0.25). No significant improvement was seen for mean transferrin levels, 0.58 g/L (0.43;1.89).

**Table 2:** Effect of intervention on blood parameters as assessed using liner mixed effect sensitivity analysis.

	Intervention		Control		Intervention effects*	
	Baseline (n = 39)	Endpoint (n = 39)	Baseline (n = 26)	Endpoint (n = 26)		
	Mean ± SD	Mean ± SD	Mean ± SD	Mean ± SD	Mean difference^#^ (95% CI)	p- value
**Serum**						
Zinc **(μmol/dl)**	16.32 ± 4.19	19.71 ± 5.67	17.48 ± 6.20	16.72 ± 3.64	0.16 (0.13;0.25)	**0.001**
Iron **(μg/dL)**	65.87 ± 23.42	85.07 ± 22.33	82.26 ± 23.37	64.22 ± 17.08	0.23 (0.11;0.33)	**<0.001**
Transferrin **(g/L)**	2.42 ± 0.34	2.96 ± 0.31	2.49 ± 0.30	2.52 ± 0.19	0.58 (0.43;1.89)	0.626
Transferrin saturation **(%)**	14.47 ± 7.50	17.73 ± 7.52	18.57 ±8.34	17.31 ± 6.78	0.33 (0.23;0.74)	**0.001**
Ferritin < 12μg/dL	9.60 ± 1.08	15.81 ± 5.08	11.85 ± 2.65	12.36 ± 2.55	0.21 (0.13;0.39)	**0.002**

A p-value of p < 0.05 denoted statistical significance.

* Intervention effect as estimated by the linear mixed model.

^#^ The mean difference is the difference between the mean intervention and control group during baseline and endpoint.

Table 3 illustrates the effect of consumption of the intervention on the anthropometrics of the children. At the endpoint, the intervention group showed a significant improvement in mean WAZ, 0.13 z-score (95% CI: 0.28; 0.34) and WHZ, 0.04 z-score (95% CI: 0.12,0.05). No significant improvements were seen for mean HAZ, 0.28 z-scores (0.34; 1.96) or mid upper arm circumference, 0.50 cm (0.31;1.70). The addition of butternut seeds to the diets of young children created positive effects on their Weight for Age and Weight for Height.

**Table 3:** Effect of intervention on anthropometrics of the children as assessed using liner mixed effect sensitivity analysis.

	Intervention		Control		Intervention effects	
	Baseline (n = 151)	Endpoint (n = 151)	Baseline (n = 85)	Endpoint (n = 85)		
	Mean ± SD	Mean ± SD	Mean ± SD	Mean ± SD	Mean difference (95% CI)	p- value
**Weight (kg)**	14.69± 2.12	16.20± 2.22	15.22± 2.13	16.43± 2.09	1.38 (1.01; 2.75)	0.66
**Weight for age (z-score)**	−1.09 ± 1.06	0.10 ± 0.66	−1.25 ± 0.89	−0.95 ± 0.68	0.13 (0.28; 0.34)	**0.02**
**Height for age (z-score)**	−0.94 ± 0.97	−0.75 ± 0.90	−0.59 ± 1.09	−0.47 ± 0.99	0.28(0.34; 1.96)	0.06
**Weight for height (z-score)**	−0.23 ± 1.12	−0.28 ± 0.99	−0.19 ± 1.23	−0.13 ± 0.98	0.04 (0.12,0.05)	**0.03**
**Mid upper arm circumference (cm)**	15.99± 1.12	16.36± 1.16	15.92± 1.10	16.62± 1.10	0.50 (0.31; 1.70)	0.68

A p-value of p < 0.05 denoted statistical significance. * Intervention effect as estimated by the linear mixed model. ^#^ The mean difference is the difference between the mean intervention and control group during baseline and endpoint.

**Fig 2:**
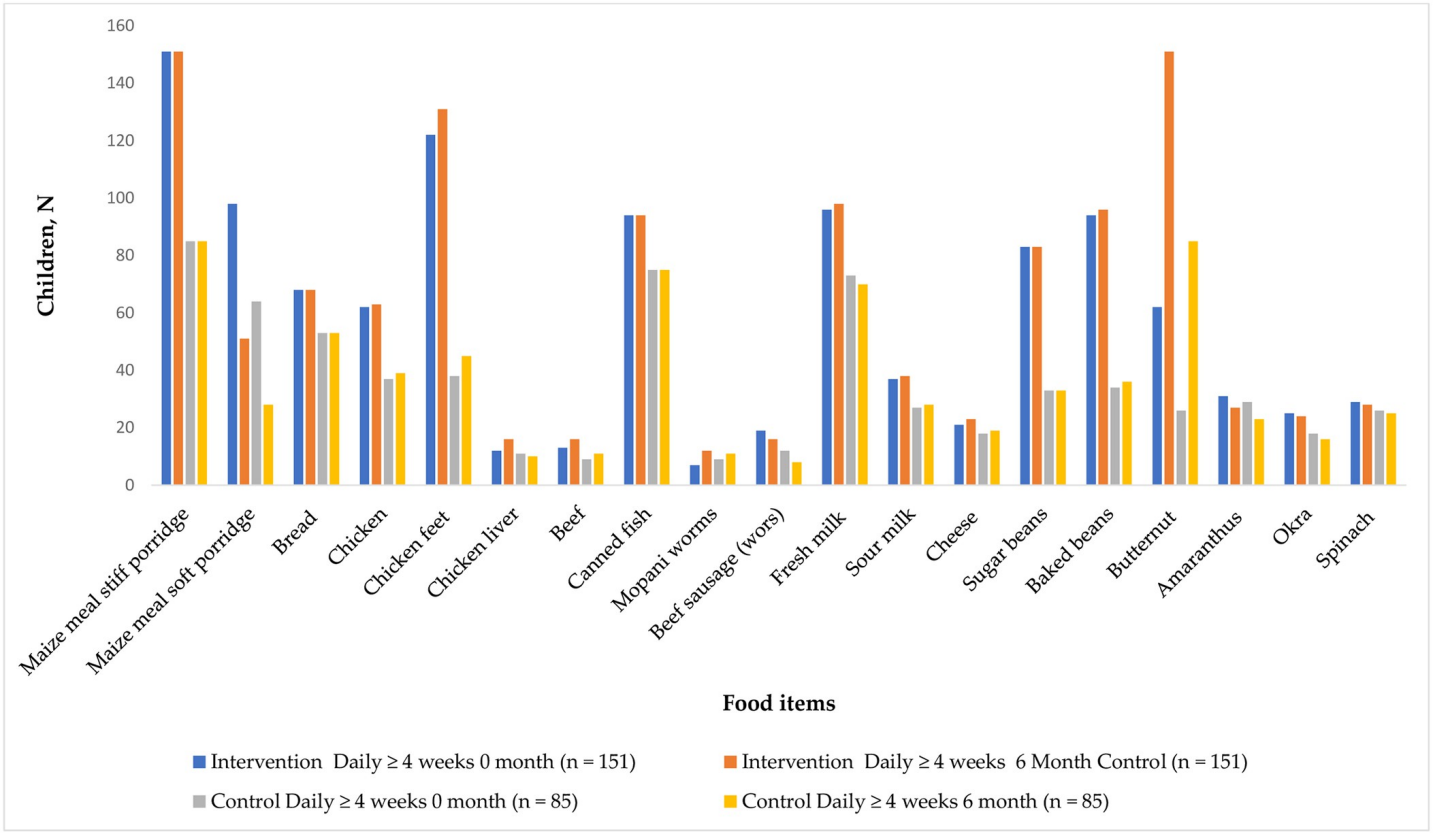
Shows consumption of iron and zinc rich foods more than four times a week in the intervention and control groups.

In the control group, there was a statistically significant increase in the consumption of chicken (p = 0.012) and chicken feet (p = 0.054). Compared to the control group, the intervention group consumed more baked beans (63.6% vs 42.3%) and sugar beans (54.9% vs 38.8%) at the end of the study. No significant differences were observed for the consumption of beef (8.6% vs 10.6%), (10.6% vs 12.95), canned fish (62.3% vs 88.2%), or beef sausage (wors) (12.6% vs 10.6%) at the end of 6 months. Fig 2. shows consumption of iron and zinc rich foods more than four times a week in the intervention and control groups.

Table 4 illustrates the nutrient intakes of the intervention and control groups. Levels of both dietary vitamin A and riboflavin increased in both groups (p <0.001 and 0.001, respectively) over the course of the study. In the children fed intervention, there was a significant improvement in the intake of iron (p < 0.001), zinc (p < 0.001). vitamin C (p <0.001), and vitamin E (p <0.001) at the end. In those who received control, a significant increase was observed for fiber (p < 0.001), vitamin B6 (p < 0.001), and vitamin B12 (p < 0.001).

**Table 4:** The ANOVA test for Nutrients intake of preschool children at 0- and 6-months consuming *Cucurbita Moschata* seeds or flesh.

Nutrients (Units)	Intervention		p-value	Control		p-value
	0 Month (Baseline) (n = 151)	6 Month (Endpoint) (n = 151)		0 Month (n = 85)	6 Month (n = 85)	
	Mean ± SD	Mean ± SD		Mean ± SD	Mean ± SD	
Energy (Kcal)	1879.50 ± 203.24	1989.46 ± 217.01	0.389	1820.36 ± 213.25	1936 ± 294.66	0.133
Carbohydrates (g)	103.17 ± 31.13	132.01 ± 11.61	0.354	112.57 ± 27.51	138.75 ± 12.93	0.776
Protein (g)	31.67 ± 13.04	34.49 ± 11.70	0.275	32.06 ± 10.20	28.62 ± 13.99	0.117
Fiber (g)	7.71 ± 3.56	8.12 ± 11.74	0.786	8.80 ± 3.85	13.68 ± 13.31	**<0.001**
Calcium (mg)	217.64 ± 133.13	233.62 ± 137.13	0.253	246.49 ± 139.17	250.62 ± 133.95	0.862
Iron (mg)	4.55 ± 2.73	8.70 ± 2.91	**<0.001**	5.25 ± 4.67	5.50 ± 2.96	0.900
Zinc (mg)	3.41 ± 2.25	4.99 ± 2.63	**<0.001**	3.98 ± 1.82	2.96 ± 2.03	0.124
Riboflavin (mg)	0.13 ± 0.18	0.62 ± 0.48	**0.001**	0.12 ± 0.50	0.53 ± 0.37	**0.001**
Vitamin B6 (mg)	0.41 ± 0.68	0. 55± 0.45	0.438	0.40 ± 0.63	0.45 ± 0.527	**<0.001**
Folate (μg)	205.84 ± 98.83	268.73 ± 100.74	0.523	202.14 ± 84.41	267.82 ± 91.01	0.648
Vitamin A (RE) (μg)	432.35 ± 84.64	5023.98 ± 123.62	**<0.001**	334.46 ± 78.23	389.62 ± 89.74	**<0.001**
Vitamin B12 (μg)	3.13 ± 3.69	4.95 ± 6.57	0.647	2.04 ± 3.07	5.42 ± 11.23	**<0.001**
Vitamin C (mg)	27.26 ± 25.90	38.55 ± 32.24	**<0.001**	26.17 ± 22.28	48.39 ± 37.42	0.430
Vitamin E (mg)	2.76 ± 2.79	9.87 ± 2.39	**<0.001**	3.52 ± 2.49	6.56 ± 2.28	**<0.001**

A p-value of p < 0.05 denoted statistical significance

## Discussion

The aim of this study was to determine the efficacy of Cucurbita Moschata squash seed paste in improving zinc and iron status, anthropometric status and dietary intake in preschool children. These results suggest that incorporation of intervention in the diet significantly improved the iron and zinc status of these young children. At the endpoint of the trial, serum ferritin levels were noticeably elevated in both the supplement and the control groups. None of the children exhibited a zinc deficiency at the outset of the study. After 6 months, serum zinc levels in the intervention group increased. But these declined in the control group, suggesting that this increase was due to the high nutrient content of the seeds.

### Serum iron level

At baseline, Motadi et al. [8] found a high prevalence of iron deficiency among young children. Additionally, the same study further revealed that more than half had not been dewormed in the same region. However, prior to the intervention, all children had received deworming. At the end of this study, serum iron and ferritin levels were noticeably elevated in both the intervention and the control groups, suggesting that this increase was due to the high nutrient content of the seeds. It is noteworthy that six months consumption of the seeds resulted in a significant improvement in serum transferrin saturation. The other iron parameters in the supplement increased but did not differ statistically between the groups. In Iran, ready-to-eat cereal and pumpkin seed kernels fortified with iron led to greater serum iron levels in adult women [35]. The fact that all children in this study had been dewormed prior to the start of the feeding trial may account for the enhancement of iron status observed.

### Serum zinc level

Unlike the previous findings by the author in 2015, none of the children exhibited a zinc deficiency at the outset of the study [8]. After 6 months, serum zinc levels in the intervention group increased. But these declined in the control group, suggesting that this increase was due to the high nutrient content of the seeds. The high compliance for both the consumption of intervention and control suggest that these foods were well-liked. Syakur et al. [36] in 2022 found that pumpkin seeds as a supplement in Indonesia improved zinc status in adults. A study was conducted by Syam et al. [37] which assessed the effect of biscuit fortified using pumpkin seeds on nutritional intake, nutritional status, and plasma zinc level of the adolescent. As many as 40 participants were chosen randomly to be provided biscuit added by pumpkin seeds (intervention) and biscuit without additional pumpkin seeds (control) for 4 weeks. The study found that micronutrient intakes of iron and zinc, were increased significantly (p = 0.000, p = 0.003, respectively. A study was conducted to develop zinc-enriched health drink mix for children: one containing pumpkin seeds and the other watermelon seeds. The purpose was to boost immunity and enhance health and nutrition status among children [38]. They concluded that the developed health drink from pumpkin seeds mix can be used for zinc enrichment, which can reduce episodes of diarrhoea, pneumonia and increase immunity among the children.

### Anthropometrics of children

The prevalence of underweight and wasting at baseline were not of public health concern [39]. Yet, at the end of the intervention, both underweight and wasting of children improved, presumably due to the intervention. These results are in line with that of Syakur et al. [36], who found that pregnant women who consumed a pumpkin seed biscuit gained weight on average, as compared to those who ate a placebo biscuit. Improvement in underweight and wasting is expected as body weight responds quickly, as compared to increases in height [37]. Yet numerous investigations have not observed changes in underweight and weight for height in response to micronutrient supplementation [37, 40, 41]. Perhaps the long supplementation period of 6 months in the present study may explain why changes in weight for age and weight for height were substantial. To date there is limited evidence available on the efficacy of *Cucurbita Moschata* seeds in the management of anthropometric status of preschool children.

### Diet consumption

The food intake from the 24-hour dietary recall and food frequency questionnaire at baseline showed low consumption of iron and zinc-rich food, which could be related to the low income and unemployment status of the parents/caregivers. Many were dependent on government grants and the low level of income makes it difficult for families to purchase animal-derived food sources due to their high cost. For unknown reasons there was a significant increase in the consumption of chicken and chicken feet in the control group at 6 months.

At the end of the study, the dietary intake of iron, zinc, vitamin C, and vitamin E increased in the group fed the intervention. This is not unexpected as pumpkin seeds are an excellent source of zinc, iron, and protein [42]. Their inclusion in the diet can help the impoverished who have difficulty affording enough protein-rich foods of animal origin to supplement their diets [43]. Peter et al. [23] suggested that the seeds could support ongoing efforts to fight micronutrient malnourishment if strengthened by nutritional education. These seeds also could be utilized to biofortify meals by adding more of a certain nutrient than is present naturally. Finally, seeds could contribute immensely to household food security by providing direct access to readily accessible nutrients at a low cost [44].

### The implementation of seeds to fight against malnutrition

This is the first investigation to analyse the nutrient content of *Cucurbita Moschata* seeds and to examine their efficacy in improving iron and zinc status in children in South Africa. Children between the ages of three and five should consume 7 to 10 mg of iron and 3 to 5 mg of zinc each day to meet their daily requirements. Researchers in several countries have analyzed the nutrient content of *Cucurbita Moschata* seeds, but data are not available for South Africa, Nigeria [45]; or Pakistan, [46]. The seeds used in this study contained 9.8 mg of iron and 4.1 mg of zinc per 100 g, which was more than adequate to meet 90 to 97% of their requirements [47]. The mineral content in the seeds is similar to that reported by Peter et al. [24] in Kenya, in which the seeds contained 9.7 mg/100 g of both iron and zinc. It was expected that during the days when the seeds were not consumed, children would not eat these seeds due to their association with poverty. Several investigations have reported that a stigma exists towards consuming indigenous leafy vegetables, especially traditional leafy vegetables and the seeds in South Africa [48–51]. Yet the children continued to eat the seeds.

Information has not been available on the effectiveness of *Cucurbita Moschata* seeds for enhancing nutrition in young children. According to Syam et al. [37], eating pumpkin seeds resulted in greater amounts of dietary iron and zinc. Other studies in Pakistan [52, 53] and Tanzania [54] have developed supplemental diets that incorporate pumpkin seeds as a component due to the product’s richness in micronutrients. According to these investigations, the consumption of seeds may effectively assist in the alleviation of micronutrient deficits [55]. Reports of the efficacy of pumpkin seeds as a nutritious food also has been observed in animals. In rats, Soltan [56] documented that giving an iron-free diet supplemented with a pumpkin seed powder without the hull, in addition with molasses and a blend of ascorbic acid for 13 weeks, caused a noticeable rise in hemoglobin, red blood cells, and serum iron. In Wistar rats, feeding pumpkin seed flour to malnourished mice significantly increased levels of serum zinc [56].

### Practical implementation of the results

The inclusion of seeds in the diets of young children could be one of the most cost effective and sustainable approaches to improve micronutrient deficiencies in less developed areas. Preschools in South Africa and low-income regions should consider the addition of these seeds to the meals in schools. Additionally, these seeds also could be incorporated in bakery products and fortified cereal products.

## Conclusion

In this research, giving children *Cucurbita Moschata* squash seeds contributed to improved serum iron and zinc status, and underweight and wasting. Children’s underweight and wasting both improved, most likely as a result of the paste made from *Cucurbita moschata* seeds. The dietary intake of iron, zinc, vitamin C, and vitamin E increased in the group fed *Cucurbita Moschata* seeds at the end of the study. These results suggest that providing *Cucurbita Moschata* seeds to low-income communities in rural areas in South Africa offered a unique, cost-effective opportunity to increase affordable, culturally appropriate nutrition in a low-income population. It is further recommended that long-term studies be conducted to assess the effects of *Cucurbita Moschata* squash seeds on other indices, such as the hemoglobin and red blood cell responses. Additionally, bio-fortification could have a profound and direct influence on nutritional status of children of rural communities where animal products are high-cost.

### Strength and limitations

A strength of the study was the use of locally available *Cucurbita Moschata* and their seeds in a population that have been consuming these traditional vegetables and their seeds for centuries [18]. The children in this research lived under low socioeconomic conditions which placed them at a great nutritional risk; the use of *Cucurbita Moschata* seeds as a meal supplement provided great benefit. Limitations was that the iron biomarker and indicator of inflammation such as C-reactive protein (CRP) was not measured due to budgetary restraints [57].

## Supporting information

S1 Dataset (PDF)

S1 Protocol (DOCX)
