# Supplementary material for: Effects of Cucurbita Moschata squash (Butternut) seed paste in improving zinc and iron status in children attending Early Childhood Development centres in Limpopo province, South Africa
Source: PLoS One. 2024 Apr 18;19(4):e0300845. doi: 10.1371/journal.pone.0300845 (PMC11025940; doi:10.1371/journal.pone.0300845)
Supplement: S1 Dataset — (PDF) [file pone.0300845.s001.pdf]

Code	B and P	Groups	Relationsh	Marital sta	Highest ed	Employem	Household	Type of inc	Monthly ir
1	1	1	1	2	5	1	2	8	3
2	1	1	1	2	5	1	2	8	3
3	1	1	3	2	4	2	3	11	4
4	1	1	1	2	5	2	2	13	4
5	1	1	1	2	4	2	1	5	3
6	1	1	1	1	6	2	2	11	3
7	1	1	1	2	5	2	2	14	3
8	1	1	3	2	5	2	2	4	3
9	1	1	1	2	5	2	2	5,1	4
10	1	1	3	1	5	2	3	5,9	3
11	1	1	1	2	7	2	1	5	4
12	1	1	1	2	5	2	2	21	7
13	1	1	3	1	6	2	5	5	1
14	1	1	1	2	6	1	2	22	4
15	1	1	1	2	5	2	1	5	3
16	1	1	3	1	6	1	1	15	3
17	1	1	3	1	6	1	1	15	3
18	1	1	3	3	5	2	5	5	2
19	1	1	1	1	4	2	5	5	2
20	1	1	1	1	5	2	2	23	6
21	1	1	1	2	6	2	2	10	3
22	1	1	1	2	5	2	2	8	7
23	1	1	3	1	5	2	3,5	5,6	3
24	1	1	1	1	5	2	5	5	1
25	1	1	1	1	5	1	1	17	3
26	1	1	3	2	4	2	1	9	2
27	1	1	1	2	4	2	2	9	3
28	1	1	3	3	1	2	3	5	3
29	1	1	1	1	4	2	2	2	6
30	1	1	1	2	5	2	3	5	4
31	1	1	3	2	4	2	1	5	2
32	1	1	1	2	6	2	5	5,9	4
33	1	1	1	2	5	2	2,5	5,9	4
34	1	1	1	1	4	2	1	5	2
35	1	1	1	1	5	2	3	5	3
36	1	1	3	2	5	2	1	5	3
37	1	1	1	1	5	2	1	4	2
38	1	1	3	2	3	2	3	5,6	7
39	1	1	1	1	4	2	3,5	5	1
40	1	1	1	1	5	2	2,5	5,1	3
41	1	1	3	1	5	2	5	5	2
42	1	1	1	1	5	2	1	5	1
43	1	1	1	2	4	2	3	5	1
44	1	1	1	1	5	2	1	5	2
45	1	1	1	1	6	1	2	24	4
46	1	1	1	2	4	2	5	5	2

47	1	1	1	1	4	1	2	13	2
48	1	1	1	1	4	2	5	5	2
49	1	1	1	1	5	2	2,5	5,1	3
50	1	1	3	1	6	1	3	6	3
51	1	1	1	2	5	1	1,5	2,5	4
52	1	1	3	3	2	1	1	9	3
53	1	1	1	1	4	2	1	5	2
54	1	1	1	2	4	2	2	9	3
55	1	1	1	1	5	1	1	5,25	4
56	1	1	1	1	5	2	3	5,6	4
57	1	1	3	1	4	1	3	1	4
58	1	1	1	1	4	2	1	5	2
59	1	1	1	2	5	2	2	9	4
60	1	2	3	1	6	2	3	2	3
61	1	2	1	2	4	4	2	15	3
62	1	2	1	1	4	2	5	5	3
63	1	2	3	1	5	2	3	5,9	3
64	1	2	1	1	5	2	1	4	3
65	1	2	2	4	2	1	5	2	1
66	1	2	1	1	5	2	1	5	2
67	1	2	3	2	4	2	2	13	7
68	1	2	3	2	5	2	2	18	7
69	1	2	3	2	5	2	2	5	7
70	1	2	1	2	5	2	2	5	2
71	1	2	1	2	5	2	2	5	2
72	1	2	1	2	4	2	2	5,9	4
73	1	2	1	2	4	2	1	5	3
74	1	2	1	2	6	1	2	11	2
75	1	2	1	1	4	2	5	5	2
76	1	2	3	1	4	1	1,5	5,17	3
77	1	2	1	2	3	2	2	14	3
78	1	2	1	2	3	2	2	14	3
79	1	2	1	1	5	2	3	12	3
80	1	2	1	2	4	2	2	8	4
81	1	2	3	1	4	1	5	5,6	4
82	1	2	3	2	5	1	3	26	3
83	1	2	3	1	4	2	1	10	7
84	1	2	1	2	5	2	5	5	3
85	1	2	1	1	4	2	1,5	5	3
86	1	2	1	2	5	2	2	11	4
87	1	2	1	2	6	1	1,5	5	1
88	1	2	1	2	4	2	1,5	5	3
89	1	2	1	2	4	2	2,5	5	2
90	1	2	1	2	4	1	2	2	5
91	1	2	1	2	4	2	1,5	5	3
92	1	2	2	1	5	2	1,5	5	3
93	1	1	1	1	6	2	5	5	1

94	1	1	1	1	5	1	1	2	3
95	1	1	3	2	1	2	3	5,6	4
96	1	1	1	2	5	1	2,5	5,11	4
97	1	1	1	1	6	2	3,5	5,6	4
98	1	1	1	1	3	2	5	5	3
99	1	1	1	1	4	2	5	5	1
100	1	1	1	2	4	2	2	11	7
101	1	1	1	1	5	2	5	5	3
102	1	1	1	2	4	2	2	5,13	4
103	1	1	3	2	4	1	3	12	4
104	1	1	1	1	5	2	3,5	5,6	4
105	1	1	1	1	6	2	5	5	2
106	1	1	1	2	5	2	2,5	5	3
107	1	1	1	1	5	2	2	10	3
108	1	1	1	1	5	2	3,5	5,6	4
109	1	1	1	1	4	2	5	5	1
110	1	1	1	2	3	1	1,5	5,12	3
111	1	1	1	1	5	2	3,5	5,6	4
112	1	1	1	1	5	2	2,5	5,11	3
113	1	1	1	1	6	2	5	5	2
114	1	1	1	2	5	2	2,5	5,11	7
115	1	1	1	1	4	2	1,5	5	3
116	1	1	1	1	7	2	1,5	5	1
117	1	1	3	1	7	2	1,5	5	1
118	1	1	1	1	7	2	1,5	5	2
119	1	1	3	2	4	2	3,5	5	3
120	1	1	3	3	1	2	3,5	5,6	4
121	1	1	3	2	5	2	3	6	3
122	1	1	1	1	6	2	5	5	2
123	1	1	1	1	4	2	5	5	1
124	1	1	1	2	4	2	2	10	4
125	1	1	3	2	6	2	3,5	15	4
126	1	1	1	1	5	1	1	8	3
127	1	1	1	1	4	2	1,5	5,1	4
128	1	1	1	1	6	1	1	10	4
129	1	1	1	1	5	1	1,5	5,12	3
130	1	1	1	2	6	1	4	1,2	5
131	1	1	1	2	5	1	4	2	2
132	1	1	1	1	5	1	1,5	5	3
133	1	1	1	1	5	1	1,5	5,12	4
134	1	1	3	2	5	1	2	2	2
135	1	2	1	2	5	1	2	15	3
136	1	2	3	1	4	2	1	9	3
137	1	2	1	2	4	2	2	4	4
138	1	2	1	2	5	2	2	9	7
139	1	2	1	1	6	1	1	3	6
140	1	2	1	1	4	1	1	12	2

141	1	2	1	2	5	2	5	5	2
142	1	2	1	1	4	1	1	12	3
143	1	2	1	1	6	2	1,5	5	3
144	1	2	1	1	6	2	1,5	5	3
145	1	2	3	1	5	2	3	9	3
146	1	2	3	1	6	2	5	5	2
147	1	2	1	1	5	2	5	5	1
148	1	2	3	2	4	2	3	6	3
149	1	2	1	1	5	2	3,5	5,6	3
150	1	2	1	1	5	2	5	5	3
151	1	2	1	1	5	2	1,5	5	2
152	1	2	1	1	5	2	4,5	5	2
153	1	2	1	2	4	1	2	15	4
154	1	2	3	2	6	1	3	2	3
155	1	2	3	2	3	2	3	6	3
156	1	2	1	1	4	1	1	12	2
157	1	2	3	1	4	2	3	6	3
158	1	2	1	1	4	2	5	5	1
159	1	2	1	2	5	2	5	5	2
160	1	2	1	1	5	1	1,5	5	2
161	1	2	3	1	6	2	4,5	5,2	5
162	1	2	3	2	3	2	3	5,7	2
163	1	2	3	2	3	2	3,5	5,7	2
164	1	2	1	2	5	2	4,5	5	2
165	1	2	1	2	5	2	4,5	5	2
166	1	2	1	1	4	2	5	5	2
167	1	2	1	1	5	2	2,5	5,11	4
168	1	2	1	2	5	2	2	10	5
169	1	2	1	1	4	2	5	5	1
170	1	2	1	1	5	2	5	5	1
171	1	2	1	2	5	2	2	20	5
172	1	2	1	1	4	1	1	1	4
173	1	2	1	1	5	1	1,5	5	3
174	1	2	1	1	3	1	1	12	3
175	1	2	1	1	4	2	5	5	2
176	1	2	1	2	5	1	2	13	4
177	1	2	1	1	4	2	5	5	2
178	1	2	1	2	4	1	2	27	5
179	1	2	1	1	5	1	1	8	4
180	1	2	3	1	5	1	5,6	8	4
181	1	2	1	1	5	2	5	5	3
182	1	2	1	1	4	2	3,5	5,6	3
183	1	2	1	1	4	1	2	9	3
184	1	2	1	2	3	2	1,5	5	3
185	1	2	3	1	4	1	1,5	5,13	4
186	1	2	1	1	5	1	1,5	5,19	4
187	1	2	3	1	4	2	1,3	5,6,9	5

188	1	2	1	2	4	1	1,5	5	2
189	1	2	1	2	6	1	4	1	5
190	1	2	2	1	4	1	5	5	1
191	1	2	3	1	5	2	5,6	5,8	4
192	1	2	2	1	5	2	2	8	4
193	1	2	1	1	4	2	5	5	1
194	1	2	1	1	6	1	1	4	3
195	1	2	1	1	3	1	1,5	5,12	3
196	1	2	1	1	4	1	1,5	5	4
197	1	2	1	2	4	2	2	9	3
198	1	2	3	1	4	2	1	8	3
199	1	2	3	2	2	2	3	5	3
200	1	2	1	1	4	2	5	5	1
201	1	2	1	1	4	2	5	5	3
202	1	2	3	1	4	1	1,5	5,8	4
203	1	2	1	1	5	2	5	5	1
204	1	2	1	1	5	2	1,5	5	1
205	1	2	1	1	4	2	5	5	3
206	1	2	1	1	4	1	2	28	3
207	1	2	1	2	5	1	2	10	4
208	1	2	1	1	5	2	2	8	3
209	1	2	1	1	5	2	3	17	4
210	1	2	1	1	5	2	3	17	4
211	1	2	1	2	4	2	2,5	5	4
212	1	2	1	1	4	2	1,5	5	2
213	1	2	3	2	4	2	2,5	5,1	3
214	1	2	1	1	4	2	5	5	1
215	1	2	1	1	3	2	5	5	1
216	1	2	1	2	4	2	2,5	5,19	4
217	1	2	1	1	4	2	1,5	5	2
218	1	2	3	1	4	1	3	12	3
219	1	2	1	1	4	1	1	8	4
220	1	2	1	1	3	1	1,5	5,12	3
221	1	2	3	2	1	2	3	5	3
222	1	2	1	1	4	2	5	5	3
223	1	2	1	2	5	1	4	19,13	4
224	1	2	3	1	4	1	3	8	3
225	1	2	1	1	4	2	3	5	3
226	1	2	1	2	4	1	2	11	3
227	1	2	3	1	5	2	3,5	5,6	3
228	1	2	1	1	6	2	3	5	3
229	1	2	3	1	4	1	3,5	5,12	4
230	1	2	3	2	4	1	3	12	3
231	1	2	1	2	4	1	1	12	3
232	1	2	1	2	3	2	2	12	3
233	1	2	1	1	4	2	1,5	5	3
234	1	2	3	1	5	1	3	12	2

235	1	2	3	1	5	1	3	12	2
236	1	2	1	1	4	2	5	5	3
237	1	2	1	1	6	2	3	5	2
238	1	2	1	1	4	2	5	5	2
239	1	2	1	1	5	2	5	5	2
240	1	2	1	1	5	2	5	5	3
241	1	2	1	1	2	2	5	5	2
242	1	2	1	1	2	2	5	5	2
243	1	2	1	1	4	2	5	5	2
244	1	2	2	1	5	1	2	29	5
245	1	2	1	2	5	2	2	20	4
246	1	2	1	1	4	2	2,5	5,1	3
247	1	2	1	1	5	2	1,5	5	3
248	1	2	3	1	5	2	1,5	5	3
249	1	2	3	2	5	2	3,5	5	4
250	1	2	1	1	5	2	1,5	5	2
251	1	2	1	2	4	2	2,5	5	7
252	1	2	1	2	4	2	2,5	5	7
253	1	2	1	1	5	2	1,5	5	2
254	1	2	3	1	5	2	3,5	5	3
255	1	2	1	1	5	2	1,5	5	2
256	1	2	1	1	6	2	5	5	2
257	1	2	1	2	5	1	1	2	2
258	1	2	3	1	5	2	4,5	5,16	4
259	1	2	3	1	5	2	4,5	5,16	4
260	1	2	1	2	5	2	2,5	5,13	4
261	1	2	3	2	5	2	3,5	5,6	4
262	1	2	1	1	4	2	1,5	5	2
263	1	2	1	1	4	2	1,5	5	2
264	1	2	1	2	6	1	4,5	5,13	4
265	1	2	1	2	6	1	4	18	6
266	1	2	1	1	4	2	1,5	5	2
267	1	2	1	1	6	2	5	5	2
268	1	2	3	1	5	1	1,5	5,12	4
269	1	2	3	2	4	2	3	6	7
270	1	2	1	2	5	2	2,5	5,13	4
271	1	2	1	2	6	1	4,5	5,9	3
272	1	2	1	2	6	2	2	3	5
273	1	2	1	1	5	2	2	15	5
274	1	2	3	2	3	2	3	5	2
275	1	2	1	1	4	2	1,5	5	3
276	1	2	1	2	6	2	2,5	5,16	4
1	2	1	3	1	5	2	3	5	2
2	2	1	3	1	5	2	3	5	2
3	2	1	2	1	5	1	2	10	3
4	2	1	1	2	5	2	2	13	4
5	2	1	1	1	4	2	1	5	1

6	2	1	1	1	6	2	5	5	2
7	2	1	1	2	5	2	1	0	7
9	2	1	1	2	5	2	2	5,11	3
10	2	1	1	2	6	2	1	5	4
11	2	1	1	1	5	2	1	5	3
12	2	1	1	1	5	2	5	5	1
13	2	1	3	1	4	2	7	5	2
14	2	1	1	2	5	2	1	5	2
16	2	1	1	2	5	2	1	5	3
17	2	1	1	2	5	2	1	5	3
18	2	1	1	2	4	1	2	9	3
19	2	1	1	1	4	2	1	5	2
20	2	1	1	1	5	2	5	5	1
22	2	1	1	2	5	2	1	5	2
23	2	1	3	3	6	2	3	5	2
24	2	1	1	2	5	2	2	10	5
25	2	1	1	1	5	2	1	5	3
26	2	1	3	2	3	2	3	30	4
90	2	1	2	2	5	1	2	2	6
27	2	1	1	2	5	2	2	2,5	4
28	2	1	3	2	2	2	3	5,6	3
29	2	1	1	1	4	2	3	5,6	3
30	2	1	3	2	5	2	3	5,6	3
31	2	1	1	1	5	2	3	31	4
32	2	1	1	2	6	1	2	9	4
33	2	1	1	2	5	2	1	5	3
34	2	1	3	2	3	2	3	5,6	3
35	2	1	3	1	5	2	1	5	1
37	2	1	3	1	5	2	3	5	3
39	2	1	1	1	5	2	1	5	2
40	2	1	1	2	5	2	1	5	3
41	2	1	1	1	4	2	1	5	2
43	2	1	1	2	4	2	1	5	3
44	2	1	1	1	5	2	1	5	2
45	2	1	1	1	5	2	2	4,5	4
46	2	1	1	2	4	2	1	5	3
47	2	1	1	1	7	2	5	5	3
48	2	1	1	1	3	2	1	5	2
49	2	1	1	2	5	2	2	4,5	4
50	2	1	3	1	6	2	3	5,6	3
51	2	1	1	2	5	1	2	10	4
53	2	1	1	1	4	2	1	5	3
54	2	1	1	2	5	1	4	1	5
55	2	1	1	2	5	1	1	8	4
57	2	1	3	1	5	2	1	5	1
60	2	2	3	1	5	1	3	2	3
61	2	2	1	2	4	2	1	5	2

62	2	2	1	2	4	2	2	10	3
63	2	2	3	2	5	1	1	4,5	4
64	2	2	3	2	3	2	1	5	2
65	2	2	1	1	4	1	1	5	2
66	2	2	3	2	4	2	3	5,6	3
67	2	2	3	2	3	2	3	5,6	4
68	2	2	1	1	4	2	8	4,5	4
69	2	2	3	1	4	2	2	5,18	6
70	2	2	1	2	5	2	2	5,8	3
71	2	2	1	1	5	2	1	5	2
72	2	2	1	2	4	2	2	5,9	3
73	2	2	1	2	4	2	4	5	3
74	2	2	1	1	6	2	2	5	2
75	2	2	1	1	4	2	1	5	1
76	2	2	1	1	5	2	1	2	1
77	2	2	1	2	4	2	1	5	3
78	2	2	1	2	4	2	1	2	2
79	2	2	1	1	4	2	1	5	1
80	2	2	1	2	4	2	2	5,6	3
81	2	2	3	2	5	2	4	5	2
82	2	2	3	1	5	1	3	5	1
83	2	2	1	1	3	2	1,3	5,6	3
84	2	2	1	2	4	2	1	5	3
85	2	2	1	1	5	2	1	5	3
86	2	2	1	2	5	2	1	2	3
87	2	2	1	2	6	1	2	5,9	4
88	2	2	1	2	4	2	2	5	2
89	2	2	1	2	4	2	3	5,6	3
91	2	2	1	2	4	2	2	5	3
92	2	2	3	1	5	2	2	5,13	4
233	2	2	1	1	5	2	3	5,2	4
93	2	1	1	1	6	2	8	4	4
95	2	1	1	1	5	2	1	5	3
96	2	1	1	2	6	1	4	31	5
97	2	1	1	1	6	2	3	5,6	3
98	2	1	1	1	3	2	1	5	2
99	2	1	1	1	4	2	5	5	3
100	2	1	1	2	4	1	4	10,5	4
101	2	1	1	1	5	1	1	4,5	4
102	2	1	1	2	3	1	2	4,5	4
104	2	1	2	1	5	1	2	10,5	4
106	2	1	1	2	5	2	2	5	3
108	2	1	2	1	5	1	2	10,5	4
109	2	1	1	1	4	1	1	12,5	4
110	2	1	1	1	3	1	1	12,5	4
111	2	1	3	1	4	2	3	5,6	3
112	2	1	1	2	5	2	2	4,5	5

113	2	1	3	2	4	2	3	12,5	3
114	2	1	1	2	5	2	5	5	2
115	2	1	1	1	3	2	1	5	3
116	2	1	3	2	5	2	3	29,5	4
117	2	1	1	2	5	2	3	29,5	4
118	2	1	3	2	5	2	3	29,5	4
119	2	1	1	1	5	2	3	5	2
120	2	1	3	3	1	2	3	7,5	3
121	2	1	3	1	4	2	1	5	1
122	2	1	1	1	6	2	5	5	2
123	2	1	1	1	5	2	5	5	2
124	2	1	1	2	5	2	1	5	4
125	2	1	3	2	6	1	4	3,5	6
126	2	1	3	1	5	1	1	8,5	5
128	2	1	1	2	6	1	4	3,5	6
129	2	1	1	1	5	1	1	12,5	3
131	2	1	1	2	5	1	2	2	6
132	2	1	1	1	5	2	1	5	2
133	2	1	1	1	5	1	1	12,5	4
134	2	1	3	2	5	1	3	2,5	6
135	2	2	1	1	5	1	2	10,5	4
136	2	2	1	1	5	1	1	10,5	4
137	2	2	1	2	4	2	2	4,5	4
138	2	2	1	2	5	2	4	5	2
139	2	2	1	1	5	1	1	3	6
140	2	2	1	1	3	2	5	5	3
141	2	2	1	1	4	2	1	5	3
142	2	2	1	1	4	2	5	5	2
143	2	2	3	2	3	2	5	5	2
144	2	2	3	2	3	2	5	5	2
145	2	2	3	1	5	2	3	5	1
146	2	2	1	1	5	2	1	5	2
147	2	2	1	1	5	2	1	5	2
148	2	2	3	3	4	2	3	5,6	4
149	2	2	1	1	5	2	1	5	2
150	2	2	1	1	5	2	1	5	2
151	2	2	1	1	5	2	5	5	2
152	2	2	1	1	5	2	5	5	2
153	2	2	3	1	4	1	1	8,5	4
154	2	2	3	2	5	2	3	2,5	4
155	2	2	1	1	5	2	1	5	3
156	2	2	1	2	4	1	1	12,5	3
157	2	2	1	1	5	2	1	5	3
158	2	2	1	1	4	2	5	5	2
159	2	2	1	1	6	2	1	5	2
160	2	2	2	1	5	2	2	5	2
161	2	2	3	1	6	2	4	1	4

162	2	2	1	1	4	1	1	8,5	4
163	2	2	1	1	4	1	1	8	4
164	2	2	1	1	5	2	2	10,5	3
165	2	2	1	1	5	2	2	10,5	3
166	2	2	3	1	4	2	1	5	2
167	2	2	1	1	5	2	1	5	1
168	2	2	1	2	4	2	2	4,5	4
169	2	2	1	1	5	2	1	5	1
170	2	2	1	2	5	2	2	2,5	4
171	2	2	1	2	5	1	4	12,5	6
172	2	2	1	1	4	1	1	8,5	4
173	2	2	1	1	4	2	1	5	3
174	2	2	1	1	4	1	1	12,5	4
175	2	2	1	1	4	2	1	5	3
176	2	2	1	1	4	2	1	5	3
178	2	2	1	2	4	2	2	27	6
179	2	2	1	1	5	2	9	10,5	5
180	2	2	1	2	5	2	3	5,6	3
182	2	2	3	1	6	1	2	11,5	4
184	2	2	1	2	2	2	5	5	3
185	2	2	3	1	4	1	3	17,5	4
186	2	2	3	1	5	2	1	5	2
187	2	2	1	1	5	1	1	8,5	3
188	2	2	1	2	5	2	5	5	2
189	2	2	3	1	2	1	3	17,5	3
190	2	2	3	2	2	1	1	12,5	3
191	2	2	1	1	6	2	1	5	3
192	2	2	1	2	4	1	2	15	6
193	2	2	1	1	4	2	1	5	1
194	2	2	3	2	3	1	1	4,5	4
195	2	2	1	1	4	2	1	5	2
196	2	2	3	2	1	2	3	5	2
199	2	2	3	1	5	2	3	5,6	3
200	2	2	1	1	4	1	2	29,5	3
201	2	2	3	1	5	2	8	4,5	5
202	2	2	3	1	4	1	1	29,5	3
203	2	2	1	1	5	2	1	5	2
204	2	2	1	1	5	2	1	5	1
206	2	2	1	1	4	2	5	5	4
208	2	2	1	1	4	2	1	5	2
209	2	2	2	2	4	2	2	5	3
210	2	2	2	2	4	2	2	5	3
211	2	2	1	1	4	2	2	10,5	5
212	2	2	1	1	4	2	1	2	4
213	2	2	3	1	6	2	1	5	4
214	2	2	1	2	4	2	5	5	2
215	2	2	3	2	3	2	2	9,5	3

216	2	2	1	1	4	2	2	5	2
217	2	2	1	1	4	2	5	5	2
218	2	2	3	2	4	1	2	29,5	4
219	2	2	3	1	5	2	1	4,5	4
221	2	2	3	2	1	2	3	5,6	3
222	2	2	1	1	4	2	1	5	4
223	2	2	1	1	5	2	2	15,5	5
224	2	2	1	1	5	2	1	5	1
225	2	2	1	2	3	2	2	9,5	3
226	2	2	1	2	4	2	1	5	2
227	2	2	3	1	4	2	5	5	2
229	2	2	3	1	5	1	2	4,5	3
230	2	2	3	1	5	2	1	12,5	4
231	2	2	3	1	5	2	1	12,5	4
232	2	2	1	2	4	2	2	10,5	3
233	2	2	3	1	5	1	2	10	3
234	2	2	3	1	4	1	3	12,5	3
235	2	2	3	1	4	1	3	12,5	3
236	2	2	1	1	4	2	1	5	1
238	2	2	3	2	1	2	3	5,6	3
239	2	2	1	1	5	2	3	5,6	3
242	2	2	3	1	5	1	2	10	3
244	2	2	3	1	4	2	2	29,5	3
245	2	2	1	2	5	2	2	27	4
246	2	2	1	1	4	2	1	5	4
247	2	2	3	1	4	2	3	5	3
248	2	2	3	1	4	2	3	5	3
249	2	2	3	1	5	1	1	4,5	4
253	2	2	1	1	5	2	1	2	3
254	2	2	3	2	5	2	3	5,6	4
255	2	2	1	1	5	2	1	5	2
256	2	2	3	1	5	2	3	8,5	4
257	2	2	1	1	5	1	1	10,5	2
258	2	2	3	2	5	2	3	5	4
259	2	2	3	2	5	2	3	5	4
260	2	2	1	2	5	1	2	13,5	4
261	2	2	3	1	5	2	1	5	2
263	2	2	1	1	4	1	1	8,5	4
264	2	2	1	1	4	1	1	13,5	5
266	2	2	1	1	4	2	1	5	2
267	2	2	1	1	6	1	1	4,5	5
268	2	2	1	1	5	2	1	5	1
269	2	2	3	2	4	2	2	5	2
273	2	2	1	1	4	2	2	15	5
274	2	2	3	2	5	2	3	5	1
275	2	2	1	1	4	2	3	10,5	5

The child r	Any other	Traditional	number of	Brick and M	number of shack		number of	Tap in Hou	Tap outsid
1	2	2	0	1	5	2	0	2	1
1	2	2	0	1	5	2	0	2	1
1	2	2	0	1	2	2	0	2	2
1	2	2	0	2	5	2	0	2	2
1	2	2	0	2	3	2	0	2	2
1	2	2	0	2	4	2	0	2	2
2	2	2	0	1	1	2	0	2	2
2	2	1	1	1	5	2	0	1	2
1	2	2	0	1	2	2	0	2	2
1	2	2	0	1	6	2	0	2	2
1	2	1	1	1	4	2	0	2	1
2	2	2	0	1	4	2	0	2	2
1	2	2	0	1	7	2	0	2	2
2	2	2	0	1	3	2	0	2	2
1	2	2	0	1	3	1	1	2	2
1	2	2	0	1	5	2	0	2	2
1	2	2	0	1	5	2	0	2	2
1	1	2	0	1	5	2	0	2	2
1	1	2	0	1	5	2	0	2	2
6	2	2	0	1	6	2	0	2	2
1	2	2	0	1	1	1	1	2	2
1	2	2	0	1	1	2	0	2	1
1	1	2	0	1	5	2	0	2	2
1	2	1	1	1	6	2	0	2	2
1	2	2	0	1	2	2	0	2	2
1	2	2	0	1	4	2	0	2	2
1	2	2	0	1	8	2	0	2	2
1	2	2	0	1	5	2	0	2	2
2	2	2	0	1	2	2	0	2	1
1	1	1	2	1	3	2	0	2	1
1	2	2	0	1	4	2	0	2	2
1	2	2	0	1	8	2	0	2	1
1	2	1	1	1	1	2	0	2	2
1	2	2	0	1	4	2	0	2	2
1	2	1	2	1	3	2	0	2	2
1	2	2	0	1	3	2	0	2	2
1	2	2	0	1	3	2	0	2	2
1	1	1	2	1	7	2	0	2	2
1	2	2	0	1	4	1	1	2	2
1	2	2	0	1	9	2	0	2	2
1	2	2	0	1	3	2	0	2	2
1	2	1	1	2	0	2	0	2	1
1	2	2	0	1	3	2	0	2	2
1	2	2	0	1	4	2	0	2	2
1	2	2	0	1	3	1	1	2	2
1	2	2	0	1	1	2	0	2	2

1	2	2	0	1	2	2	0	2	2
1	2	2	0	1	4	2	0	2	2
1	2	1	1	1	6	2	0	2	2
1	2	2	0	1	8	1	1	1	2
1	2	2	0	1	9	2	0	2	2
1	2	1	1	1	4	2	0	2	2
1	2	2	0	1	3	2	0	2	2
1	2	2	0	1	3	2	0	2	2
1	2	2	0	1	5	1	1	2	2
1	1	2	0	1	5	2	0	2	2
1	2	1	1	1	5	2	0	2	2
1	2	2	0	1	4	2	0	2	2
1	2	1	1	1	4	2	0	2	2
1	2	2	0	1	4	2	0	2	1
1	2	1	1	1	1	2	0	2	2
1	2	2	0	1	5	2	0	2	2
1	2	1	1	1	5	2	0	2	2
1	2	2	0	1	4	2	0	2	2
2	2	1	3	2	0	2	0	2	2
2	2	2	0	1	2	2	0	2	2
1	2	1	1	1	4	2	0	2	2
2	2	1	1	1	5	2	0	2	2
2	2	1	1	1	5	2	0	2	2
1	2	2	0	1	4	1	1	2	2
1	2	2	0	1	4	1	1	2	2
1	2	2	0	1	3	2	0	2	2
1	2	2	0	1	3	2	0	2	2
1	2	2	0	1	11	2	0	2	2
1	2	1	1	1	4	2	0	2	2
1	2	1	1	1	4	2	0	2	2
1	2	1	1	1	3	2	0	2	2
1	2	1	1	1	3	2	0	2	2
1	2	2	0	1	3	2	0	2	1
1	2	1	2	1	5	2	0	2	1
1	1	2		1	11	2	0	2	2
1	1	1	1	1	6	2	0	2	2
1	2	1	1	1	3	2	0	2	2
1	2	2	0	1	4	2	0	2	2
1	2	2	0	1	4	2	0	2	2
1	2	2	0	1	1	2	0	2	2
1	2	2	0	1	6	2	0	2	1
1	2	2	0	1	1	2	0	2	2
1	2	1	2	1	5	2	0	2	2
2	2	2	0	1	9	2	0	2	1
1	2	2	0	1	4	1	1	2	2
1	2	2	0	1	5	2	0	2	2
1	2	1	1	1	6	2	0	2	2

1	2	2	0	1	4	2	0	2	2
1	1	2	0	1	8	2	0	2	2
1	2	1	1	1	8	2	0	1	1
1	1	1	1	1	7	1	1	2	1
1	2	2	0	1	4	1	1	2	2
1	2	2	0	1	5	2	0	2	1
1	2	1	1	1	4	2	0	2	2
1	2	2	0	1	7	2	0	2	2
1	2	1	1	1	4	2	0	2	2
1	2	1	1	1	4	2	0	2	1
1	2	2	0	1	3	2	0	2	1
1	2	2	0	1	3	2	0	2	1
1	2	2	0	1	11	2	0	2	1
1	2	2	0	1	7	2	0	1	2
1	2	2	0	1	3	2	0	2	1
1	2	1	1	1	4	1	1	2	2
1	2	2	0	1	7	2	0	2	2
1	1	2	0	1	4	2	0	2	2
1	2	1	1	1	6	2	0	2	1
1	1	2	0	1	3	2	0	2	1
1	2	2	0	1	1	1	1	2	2
1	2	2	0	1	6	2	0	2	2
2	1	1	3	1	1	1	1	2	2
1	2	1	1	1	3	1	1	1	2
1	2	1	1	1	3	1	1	1	2
1	2	2	0	1	8	2	0	2	1
2	2	2	0	1	6	1	1	2	1
2	2	2	0	1	6	1	1	2	2
1	2	2	0	1	3	2	0	2	2
1	2	2	0	1	2	2	0	2	2
1	1	1	1	1	8	1	1	2	2
1	2	1	1	1	6	2	0	1	1
2	1	2	0	1	4	1	1	2	2
1	2	2	0	1	8	1	1	1	1
1	2	2	0	1	10	2	0	1	2
1	2	1	6	2	0	1	1	1	2
2	2	2	0	1	9	2	0	1	2
1	2	1	1	1	6	2	0	2	1
1	2	2	0	1	2	2	0	1	2
1	2	2	0	1	5	1	1	1	1
1	2	1	1	1	14	2	0	1	1
1	2	2	0	1	11	2	0	1	2
1	1	2	0	1	6	2	0	2	2
2	2	2	0	1	4	2	0	1	2
1	2	2	0	1	2	2	0	2	2
2	2	2	0	1	6	1	1	2	1
1	2	2	0	1	4	2	0	2	2

1	2	2	0	1	2	2	0	2	2
1	2	2	0	1	1	2	0	2	1
1	2	2	0	2	0	1	1	2	2
1	2	2	0	2	0	1	1	2	2
1	2	2	0	1	2	2	0	2	1
1	2	2	0	1	5	2	0	2	1
1	2	2	0	2	0	1	1	2	2
1	2	2	0	1	8	1	2	1	1
1	1,1	2	0	1	8	2	0	2	2
1	2	1	1	1	4	1	1	2	2
1	2	2	0	1	2	2	0	2	2
1	2	2	0	1	2	2	0	2	2
1	2	2	0	1	2	2	0	2	2
1	2	2	0	1	8	2	0	1	2
1	2	1	3	1	5	2	0	1	1
1	2	2	0	1	4	1	1	2	1
1	2	1	2	1	4	2	0	2	1
1	2	2	0	1	6	1	1	2	1
1	1,1	2	0	1	4	2	0	2	2
1	2	2	0	1	1	1	1	2	1
1	2	2	0	1	8	2	0	2	1
1	1,2	2	0	1	6	2	0	2	2
1	1,2	2	0	1	6	2	0	2	2
1	2	2	0	1	4	2	0	2	2
1	2	2	0	1	4	2	0	2	2
1	2	2	0	1	3	2	0	2	1
1	2	2	0	1	4	2	0	2	2
1	2	2	0	1	3	2	0	2	2
1	2	2	0	1	4	2	0	2	2
1	2	2	0	1	8	2	0	2	2
1	2	2	0	1	1	2	0	2	2
1	2	2	0	1	4	2	0	2	2
1	2	2	0	1	3	2	0	2	1
1	2	2	0	1	4	1	1	2	2
1	2	2	0	1	1	2	0	2	1
1	2	2	0	1	5	2	0	2	2
1	2	2	0	1	4	2	0	2	2
2	2	2	0	1	7	2	0	2	1
1	2	2	0	1	9	1	1	1	1
1	2	2	0	1	9	1	1	1	1
1	2	2	0	1	4	2	0	2	2
1	2	2	0	1	7	2	0	2	2
1	2	2	0	1	4	1	1	2	2
1	2	2	0	1	4	2	0	2	2
1	2	2	0	1	4	1	1	2	2
1	2	2	0	1	4	2	0	2	2
1	1,1	2	0	1	8	2	0	2	2

1	2	2	0	1	5	2	0	2	1
1	2	2	0	1	6	2	0	2	2
1	2	2	0	1	8	2	0	2	2
1	2	2	0	1	4	2	0	2	2
1	2	2	0	1	5	2	0	2	2
1	2	2	0	1	3	2	0	2	2
1	2	2	0	1	8	2	0	1	1
1	2	2	0	1	4	2	0	2	2
1	2	1	1	1	2	2	0	2	1
1	2	2	0	1	3	2	0	2	2
1	2	2	0	1	3	2	0	2	1
1	2	2	0	1	4	1	2	2	1
1	2	2	0	1	4	2	0	2	2
1	2	2	0	1	4	2	0	2	2
1	2	1	1	1	4	1	1	2	2
1	2	2	0	1	1	2	0	2	2
1	2	2	0	1	4	2	0	2	2
1	2	2	0	1	4	2	0	2	2
1	2	2	0	1	4	2	0	2	2
2	2	2	0	1	8	2	0	1	1
1	2	2	0	1	3	2	0	2	1
1	2	2	0	1	6	2	0	2	1
1	2	2	0	1	6	2	0	2	1
1	2	2	0	1	4	1	1	2	2
1	2	2	0	1	3	2	0	2	2
1	2	2	0	1	3	1	1	2	2
1	2	2	0	1	4	2	0	2	2
1	2	2	0	1	6	2	0	2	2
1	2	2	0	1	5	2	0	2	1
1	2	2	0	1	5	2	0	2	1
1	2	1	1	1	3	2	0	2	2
1	2	2	0	1	2	1	1	2	1
1	2	2	0	1	4	1	1	2	2
1	2	2	0	1	5	2	0	2	2
1	2	2	0	1	4	2	0	2	2
1	2	2	0	1	8	2	0	2	2
1	2	2	0	1	6	2	0	1	2
1	2	2	0	1	3	2	0	2	2
1	2	2	0	1	4	2	0	2	2
1	2	1	1	1	8	1	1	2	1
1	2	2	0	1	4	2	0	2	2
1	2	2	0	1	5	1	1	2	1
1	2	2	0	1	4	2	0	2	2
1	2	2	0	1	4	2	0	2	2
2	2	2	0	2	0	1	1	2	2
1	2	2	0	1	4	2	0	2	1
1	2	2	0	1	5	2	0	2	2

1	2	2	0	1	5	2	0	2	2
1	2	2	0	1	4	2	0	2	2
1	2	1	1	1	2	2	0	2	2
1	2	2	0	1	4	2	0	2	2
1	2	2	0	1	4	2	0	2	2
1	2	2	0	1	3	2	0	2	2
1	2	2	0	1	4	2	0	2	2
1	2	2	0	1	4	2	0	2	2
1	2	2	0	1	4	2	0	2	2
2	2	2	0	1	8	2	0	2	2
2	2	2	0	1	11	1	1	1	2
1	2	2	0	1	2	2	0	1	2
1	2	2	0	1	3	2	0	2	2
1	2	2	0	1	3	2	0	2	2
1	2	2	0	1	4	2	0	2	1
1	2	2	0	1	3	2	0	2	2
1	2	2	0	1	1	1	1	2	2
1	2	2	0	1	1	1	1	2	2
1	2	2	0	1	4	2	0	2	1
1	2	1	2	1	5	2	0	2	2
1	2	2	0	2	0	1	1	2	2
1	2	1	1	1	3	2	0	2	1
1	2	1	1	1	2	2	0	2	1
1	2	2	0	1	2	2	0	2	2
1	2	2	0	1	2	2	0	2	2
1	2	2	0	1	3	1	1	2	2
1	2	2	0	1	4	2	0	2	1
1	2	2	0	1	3	2	0	2	2
1	2	2	0	1	2	2	0	2	2
1	2	2	0	1	4	1	1	2	1
2	2	2	0	1	7	2	0	2	1
1	2	2	0	1	1	2	0	2	2
1	2	2	0	1	1	2	0	2	2
1	2	1	1	1	4	2	0	2	2
1	2	1	1	1	6	2	0	2	2
1	2	2	0	1	7	1	1	2	1
1	2	1	2	1	4	2	0	2	1
2	2	2	0	1	7	2	0	2	2
2	2	2	0	1	4	2	0	1	2
1	2	1	3	1	3	2	0	2	2
1	2	1	1	1	5	2	0	2	1
1	2	1	1	1	8	2	0	2	1
1	2	2	0	1	3	2	0	2	2
1	2	2	0	1	3	2	0	2	2
1	2	2	0	1	8	2	0	1	2
1	2	2	0	1	6	2	0	2	1
1	2	2	0	1	3	2	0	2	2

1	2	2	0	1	5	2	0	2	1
2	2	2	0	1	1	2	0	1	2
1	2	2	0	1	2	2	0	2	2
1	2	2	0	1	6	2	0	2	2
1	2	1	1	1	4	2	0	2	2
1	2	2	0	1	6	2	0	2	2
1	2	2	0	1	4	2	0	2	2
1	2	2	0	1	3	2	0	2	2
1	2	2	0	1	4	2	0	2	2
1	2	2	0	1	4	2	0	2	2
1	2	2	0	1	5	2	0	2	2
1	2	2	0	1	7	2	0	2	2
1	2	2	0	1	6	2	0	2	2
1	2	2	0	1	5	2	0	2	2
1	2	2	0	1	7	2	0	2	2
1	2	1	1	1	6	2	0	2	1
1	2	2	0	1	2	2	0	2	2
2	2	2	0	1	6	2	0	2	2
2	2	2	0	1	6	2	0	2	2
1	2	1	1	1	4	2	0	2	2
1	2	2	0	1	5	2	0	1	1
1	2	1	1	1	4	2	0	2	2
1	2	1	2	1	4	2	0	2	2
1	2	1	1	1	4	2	0	2	2
1	2	2	0	1	8	1	1	2	1
1	2	1	1	1	1	2	0	2	2
1	2	1	1	1	5	2	0	2	2
1	2	1	1	1	3	2	0	2	2
1	2	2	0	1	3	2	0	2	2
1	2	2	0	1	4	1	1	2	2
1	2	2	0	1	3	1	1	1	1
1	2	2	0	1	2	2	0	1	1
1	2	2	0	1	3	1	1	1	1
1	2	1	1	1	4	2	0	2	2
1	2	2	0	1	3	1	1	2	2
1	2	2	0	1	1	1	1	1	1
1	2	2	0	1	3	2	0	2	2
1	2	1	1	1	5	2	0	2	2
1	2	1	1	1	6	2	0	2	2
1	2	1	1	1	8	1	1	2	1
1	2	2	0	1	4	2	0	1	1
1	2	2	0	1	3	2	0	1	1
1	2	2	0	1	5	1	1	2	2
1	2	1	1	1	7	2	0	2	1
1	2	1	1	1	8	2	0	2	2
1	2	1	2	1	4	2	0	2	1
1	2	1	1	1	8	2	0	2	2

1	2	1	1	1	6	2	0	2	2
1	2	1	1	1	7	2	0	2	2
1	2	2	0	1	5	2	0	2	2
1	2	2	0	1	2	2	0	2	2
1	2	1	1	1	5	2	0	2	1
1	2	1	1	1	7	2	0	1	1
1	2	1	2	1	5	2	0	2	2
1	2	1	2	1	6	2	0	2	2
1	2	1	1	1	5	2	0	1	1
1	2	2	0	1	4	2	0	2	2
1	2	2	0	1	3	2	0	2	2
1	2	2	0	1	3	1	1	2	1
1	2	2	0	1	9	2	0	2	2
1	2	1	1	1	4	2	0	2	2
1	2	1	1	1	4	2	0	2	2
1	2	2	0	1	4	2	0	2	2
1	2	2	0	1	4	2	0	2	2
1	2	1	1	1	3	2	0	2	2
1	2	1	1	1	5	2	0	2	2
1	2	2	0	1	11	1	1	2	2
1	2	1	1	1	2	2	0	2	2
1	2	1	1	1	3	2	0	2	2
1	2	1	1	1	5	2	0	2	2
1	2	2	0	1	4	2	0	2	2
1	2	2	0	1	1	1	1	2	2
1	2	2	0	1	1	2	0	2	2
1	2	2	0	1	2	2	0	2	2
1	2	1	2	1	5	2	0	2	2
1	2	2	0	1	6	1	1	2	2
1	2	2	0	1	4	2	0	2	2
1	2	1	2	1	2	2	0	2	2
2	2	2	0	1	6	2	0	2	2
1	2	2	0	1	5	2	0	2	1
1	2	2	0	1	7	2	0	1	2
1	2	1	1	1	8	1	1	2	1
1	2	2	0	1	4	2	0	2	2
1	2	2	0	1	5	1	1	2	1
1	2	2	0	1	3	2	0	2	2
1	2	2	0	1	8	2	0	2	2
1	2	2	0	1	4	2	0	2	2
1	2	2	0	1	3	2	0	2	2
1	2	2	0	1	11	2	0	2	1
1	2	2	0	1	3	2	0	2	2
1	2	2	0	1	4	2	0	1	1
1	2	2	0	1	5	2	0	2	2
1	2	2	0	1	3	2	0	2	2
1	2	2	0	1	6	1	1	1	1

1	2	2	0	1	5	2	0	1	1
1	2	2	0	1	7	1	1	2	2
1	2	1	1	1	4	2	0	1	1
1	2	2	0	1	3	1	1	2	1
1	2	2	0	1	3	1	1	2	1
1	2	2	0	1	3	1	1	2	1
1	2	2	0	1	8	2	0	2	1
1	2	2	0	1	6	1	1	2	2
1	2	2	0	1	4	1	1	2	2
1	2	2	0	1	3	1	1	2	1
1	2	2	0	1	2	2	0	2	1
1	2	2	0	1	8	2	0	1	1
1	2	2	0	1	11	1	1	1	1
2	2	2	0	1	7	1	1	2	1
1	2	2	0	1	11	1	1	1	1
1	2	1	1	1	6	2	0	2	1
2	2	2	0	1	14	2	0	1	1
1	2	2	0	1	4	1	1	1	1
1	2	2	0	1	5	2	0	1	2
1	2	2	0	1	14	2	0	1	1
1	2	2	0	1	9	1	1	2	1
1	2	2	0	1	4	2	0	1	1
1	2	2	0	1	4	2	0	1	2
1	2	2	0	1	2	2	0	2	2
2	2	2	0	1	6	2	0	1	1
1	2	2	0	1	4	2	0	2	1
1	2	2	0	1	2	2	0	2	2
1	2	2	0	1	1	2	0	2	1
1	2	2	0	1	5	1	1	2	1
1	2	2	0	1	5	1	1	2	1
1	2	2	0	1	2	1	1	2	2
1	2	2	0	1	5	2	0	2	2
1	2	2	0	2	0	1	1	2	2
1	2	2	0	1	8	1	1	2	2
1	2	2	0	1	8	2	0	1	1
1	2	1	1	1	4	2	0	2	2
1	2	1	2	1	4	2	0	2	1
1	2	1	2	1	4	2	0	2	1
1	2	2	0	1	7	2	0	2	2
1	2	2	0	1	5	2	0	2	1
1	2	2	0	1	5	2	0	2	2
1	2	2	0	1	5	2	0	2	2
1	2	2	0	1	2	1	0	2	2
1	2	2	0	1	6	2	0	2	1
1	2	2	0	2	0	1	1	2	2
1	2	2	0	1	1	1	1	2	2
2	2	2	0	1	8	2	0	1	1

1	2	2	0	1	6	2	0	2	1
2	2	2	0	1	6	2	0	2	1
1	2	2	0	1	4	2	0	2	2
1	2	2	0	1	4	2	0	2	2
1	2	2	0	1	4	2	0	2	2
1	2	2	0	1	4	1	1	2	2
1	2	2	0	1	4	2	0	2	2
1	2	2	0	1	4	2	0	2	2
1	2	2	0	1	7	2	0	1	2
1	2	2	0	1	8	2	0	2	2
1	2	2	0	1	5	2	0	2	2
1	2	2	0	1	3	1	1	2	1
1	2	2	0	1	4	2	0	2	2
1	2	2	0	1	1	2	0	2	2
1	2	2	0	1	4	2	0	2	2
2	2	2	0	1	7	2	0	1	1
1	2	2	0	1	13	1	1	2	2
1	2	1	1	1	5	2	0	1	2
1	2	2	0	1	7	1	1	2	2
1	2	2	0	1	4	2	0	2	2
1	2	2	0	1	7	2	0	2	1
1	2	2	0	1	8	2	0	2	2
1	2	2	0	1	8	1	1	2	1
1	2	2	0	1	5	2	0	2	1
1	2	2	0	1	8	1	1	2	2
1	2	2	0	1	8	2	0	2	1
1	2	2	0	1	4	2	0	2	2
2	2	2	0	1	8	2	0	1	2
1	2	2	0	1	4	1	1	2	2
1	2	2	0	1	7	2	0	1	2
1	2	2	0	1	6	2	0	2	2
1	2	2	0	1	3	2	0	2	2
1	2	2	0	1	4	1	2	1	2
1	2	2	0	1	4	2	0	2	2
1	2	1	1	1	5	2	0	2	1
1	2	2	0	1	4	1	1	2	2
1	2	2	0	1	6	2	0	2	1
1	2	2	0	1	4	2	0	2	2
1	2	2	0	1	4	2	0	2	2
1	2	2	0	1	1	2	0	2	2
1	2	2	0	1	7	1	1	2	2
1	2	2	0	1	7	1	1	2	2
1	2	2	0	1	3	2	0	2	2
1	1	2	0	1	6	2	0	2	1
1	2	2	0	1	4	2	0	2	2
1	2	2	0	1	4	2	0	2	2
1	2	1	1	1	7	2	0	2	2

1	2	2	0	1	5	2	0	2	2
1	2	2	0	1	5	2	0	2	1
1	2	1	1	1	3	2	0	2	2
1	2	2	0	1	3	2	0	2	1
1	2	2	0	1	6	1	3	1	1
1	2	2	0	1	4	1	1	2	2
1	2	2	0	1	4	2	0	2	1
1	2	2	0	1	6	2	0	1	2
1	2	1	1	1	3	2	0	2	2
1	2	2	0	1	4	2	0	2	2
1	2	2	0	1	4	2	0	2	1
1	2	2	0	1	5	2	0	2	2
1	2	2	0	1	4	2	0	2	2
1	2	2	0	1	4	2	0	2	2
1	2	2	0	2	0	1	1	2	2
2	2	2	0	1	6	2	0	1	2
1	2	2	0	1	5	2	0	2	2
1	2	2	0	1	5	2	0	2	2
1	2	2	0	1	3	2	0	2	2
1	2	2	0	1	5	2	0	1	1
1	2	2	0	1	6	2	0	2	1
2	2	2	0	1	6	2	0	1	2
1	2	2	0	1	4	2	0	2	2
2	2	2	0	1	4	1	1	2	2
1	2	1	1	1	2	2	0	1	1
1	2	2	0	1	3	2	0	2	1
1	2	2	0	1	3	2	0	2	1
1	2	2	0	1	3	2	0	2	2
1	2	2	0	1	4	1	1	2	2
1	2	1	1	1	5	2	0	2	1
1	2	2	0	2	2	1	1	2	2
1	2	2	0	1	9	2	0	2	1
1	2	1	1	1	2	2	0	1	1
1	2	2	0	1	2	1	1	2	2
1	2	2	0	1	2	1	1	2	2
1	2	2	0	1	3	1	1	2	1
1	2	2	0	1	3	2	0	2	2
1	2	2	0	1	2	1	1	2	2
1	2	2	0	1	7	1	1	1	1
1	2	2	0	1	2	2	0	2	1
1	2	2	0	1	10	1	1	1	1
1	2	1	1	1	4	2	0	2	2
1	2	1	1	1	6	2	0	2	1
2	2	2	0	1	4	2	0	2	2
1	2	1	1	2	2	2	2	2	2
1	2	2	0	1	4	2	0	1	2

communal	River	Tank	Borehole	spring/wel	Flush Toile	Flush Toile	Pit latrine	No of Pit la	Bush
2	2	2	2	2	2	1	2	0	2
2	2	2	2	2	2	1	2	0	2
1	2	2	2	2	2	2	1	1	2
1	2	2	2	2	2	2	1	1	2
1	2	2	2	2	2	2	1	1	2
1	2	2	2	2	2	2	1	1	2
1	2	2	2	2	2	2	1	1	2
2	1	2	2	2	2	2	1	1	2
1	2	2	2	1	2	2	1	1	2
1	2	2	2	2	2	2	1	1	2
2	2	2	2	1	2	2	1	1	2
1	2	2	2	1	2	2	1	1	2
1	2	2	2	2	2	2	1	3	2
2	1	2	2	2	2	2	1	1	2
1	2	2	2	2	2	2	1	2	1
1	1	2	2	2	2	2	1	1	2
1	1	2	2	2	2	2	1	1	2
1	2	2	2	1	2	2	1	1	2
1	2	2	2	1	2	2	1	1	2
1	2	2	2	1	2	2	1	1	2
1	2	1	2	2	2	2	1	1	1
2	1	2	2	2	2	2	1	1	1
1	2	1	2	2	2	2	1	1	2
1	2	2	2	2	2	2	1	3	1
1	2	1	2	2	2	2	1	1	2
1	2	1	2	2	2	2	1	1	2
1	2	2	2	2	2	2	1	1	2
1	2	2	2	2	2	2	1	1	2
1	2	2	2	2	2	2	2	0	1
2	2	2	2	2	2	2	1	1	2
2	2	2	2	2	2	2	1	1	2
1	2	1	1	2	2	2	1	1	2
1	2	2	2	2	2	2	1	1	2
1	2	2	2	2	2	2	1	1	2
2	2	2	1	2	2	2	1	1	2
1	2	2	2	2	2	2	1	1	2
1	2	1	1	2	2	2	1	1	2
1	2	2	2	2	2	2	1	1	2
1	2	2	2	2	2	2	1	1	2
1	2	2	2	1	2	2	1	1	2
1	2	2	2	2	2	2	1	1	2
2	2	2	2	2	2	2	1	1	2
1	2	2	2	2	2	2	1	1	2
2	2	2	1	2	2	2	1	1	2
2	2	2	1	2	2	2	1	1	2
1	2	2	2	2	2	2	1	1	2

1	2	2	2	2	2	2	1	1	2
2	1	2	2	2	2	2	1	1	2
1	2	2	2	2	2	2	1	1	2
2	2	1	1	2	2	2	1	1	2
1	2	2	2	1	2	2	1	1	2
1	2	2	2	2	2	2	1	1	2
2	2	2	1	2	2	2	1	1	2
1	2	2	2	2	2	2	1	1	2
1	2	2	2	2	2	2	1	1	2
1	2	2	2	2	2	2	1	2	2
1	2	2	2	2	2	2	1	1	2
1	2	2	2	2	2	2	1	1	2
1	2	2	2	2	2	2	1	1	2
1	1	2	2	2	2	2	1	1	2
1	2	2	2	2	2	2	1	1	2
1	2	2	2	2	2	2	1	1	2
1	2	2	2	2	2	2	1	1	2
1	2	2	2	2	2	2	1	1	2
1	2	2	2	2	2	2	1	1	2
1	2	2	2	2	2	2	1	1	2
1	2	2	2	2	2	2	1	2	2
1	2	2	1	2	2	2	1	2	2
1	2	2	1	2	2	2	1	2	2
1	2	2	2	2	2	2	1	1	1
1	2	2	2	2	2	2	1	1	1
1	2	2	2	2	2	2	1	1	2
1	2	2	2	2	2	2	1	1	2
1	2	2	2	2	2	2	1	1	2
1	2	2	2	2	2	2	1	1	2
1	1	2	2	2	2	2	1	1	2
1	2	2	2	2	2	2	1	1	2
1	2	2	2	2	2	2	1	1	2
1	2	2	2	2	2	2	1	1	2
2	1	1	2	2	2	2	1	1	2
1	2	2	2	2	2	2	1	2	2
1	2	2	2	2	2	2	1	2	2
1	2	2	2	2	2	2	1	1	2
1	2	2	2	2	2	2	1	1	2
1	2	2	2	2	2	2	1	1	2
1	1	2	2	2	2	2	1	1	2
2	2	2	2	2	2	2	1	1	2
1	2	2	2	2	2	2	1	1	2
1	2	2	2	2	2	2	1	1	2
2	2	2	2	2	2	2	1	1	2
1	2	2	2	2	2	2	1	3	2
1	2	2	2	2	2	2	1	1	2
1	1	2	2	2	2	2	1	1	2

1	2	2	2	2	2	2	1	1	2
1	1	2	2	2	2	2	1	1	2
2	1	2	1	1	1	2	1	2	2
1	2	1	1	2	2	2	1	1	2
1	2	2	2	2	2	2	2	0	1
1	2	2	2	2	2	2	1	1	2
1	1	2	2	2	2	2	1	1	2
1	2	2	2	2	2	2	1	1	2
1	2	2	2	2	2	2	1	1	2
1	2	2	2	2	2	2	1	1	2
1	2	2	2	2	2	2	1	1	2
2	2	2	2	2	2	2	1	1	2
2	2	2	2	2	2	2	1	1	2
1	2	2	2	2	2	2	1	1	2
1	2	2	2	2	2	2	1	1	2
1	2	2	2	2	2	2	1	2	2
1	2	2	2	2	2	2	1	1	2
1	2	2	2	2	2	2	1	1	2
1	2	2	2	2	2	2	1	1	2
2	2	2	2	2	2	2	1	1	2
1	2	2	2	2	2	2	1	1	2
1	2	2	2	2	2	2	1	1	2
2	2	2	2	2	2	2	1	1	2
2	2	2	2	2	2	2	1	1	2
2	2	2	2	2	2	2	1	1	2
2	2	2	2	2	1	2	1	1	2
1	1	2	2	2	2	2	1	1	2
1	2	2	2	2	2	2	1	1	2
1	2	2	2	2	2	2	1	1	2
1	2	2	2	2	2	2	1	1	2
1	2	2	2	2	2	2	1	1	2
1	2	2	2	2	2	2	1	1	2
1	2	2	2	2	2	2	1	1	2
2	2	2	1	2	2	2	1	1	2
2	2	1	1	2	1	2	1	1	2
2	2	2	2	2	2	2	1	1	2
1	1	2	2	2	1	2	2	1	2
1	2	1	2	2	2	2	1	1	2
2	2	2	1	2	2	2	1	1	2
2	2	2	2	2	2	2	1	1	2
1	2	2	2	2	2	2	1	1	2
2	2	2	2	2	2	2	1	1	2
1	2	2	2	2	2	2	1	1	2
2	2	1	1	2	2	2	2	0	1
1	2	2	2	2	2	2	1	1	2
2	2	2	2	2	2	2	1	1	2
1	2	2	2	2	2	2	1	1	2

2	1	2	2	2	2	2	1	1	2
2	2	2	2	2	2	2	2	0	1
1	2	2	2	2	2	2	2	0	1
1	2	2	2	2	2	2	2	0	1
2	2	2	2	2	2	2	1	1	2
2	2	2	2	2	2	2	1	1	2
1	2	2	2	2	2	2	2	0	2
1	2	2	2	2	2	2	1	2	2
1	2	2	2	2	2	2	1	1	2
1	2	2	2	2	2	2	1	1	2
1	2	2	2	2	2	2	1	1	2
1	2	2	2	2	2	2	1	1	2
1	2	2	2	2	2	2	1	1	2
2	2	1	1	2	2	2	1	1	2
1	2	2	1	2	2	2	1	2	2
2	2	2	2	2	2	2	1	1	2
1	2	2	2	2	2	2	1	2	2
1	2	2	2	2	2	2	1	1	2
1	2	2	2	2	2	2	1	1	2
1	2	1	2	2	2	2	2	0	1
1	2	1	2	2	2	2	1	2	2
1	2	2	2	2	1	2	1	2	2
1	2	2	2	2	2	2	1	2	2
1	2	2	2	2	2	2	1	1	2
1	2	2	2	2	2	2	1	1	2
1	2	2	2	2	2	2	1	1	2
2	2	2	2	2	2	2	1	1	2
1	2	2	2	2	2	2	1	1	2
1	2	2	2	2	2	2	1	1	2
2	2	1	2	2	1	2	1	1	2
1	2	2	2	2	2	2	1	1	2
2	2	1	2	2	2	2	1	1	2
2	2	2	1	2	2	2	1	1	2
1	2	2	2	2	2	2	1	1	2
1	2	2	2	2	2	2	1	1	2
2	2	1	2	2	2	2	1	1	2
2	2	1	2	2	2	2	1	1	2
2	2	2	2	2	2	2	1	1	2
1	2	1	1	2	2	2	1	2	2
1	2	1	1	2	2	2	1	2	2
1	2	2	2	2	2	2	1	1	2
1	2	2	2	2	2	2	1	1	2
1	2	2	2	2	2	2	1	1	2
1	2	2	2	2	2	2	1	1	2
1	2	2	2	2	2	2	1	1	2
1	2	2	2	2	2	2	1	1	2
1	2	2	2	2	2	2	1	1	2

1	2	2	1	2	2	2	1	1	2
1	2	2	2	2	2	2	1	1	2
2	2	1	2	2	2	2	1	1	2
1	2	2	2	2	2	2	1	1	2
1	2	2	2	2	2	2	1	1	2
1	2	2	2	2	2	2	1	1	2
2	2	2	2	2	1	2	1	1	2
1	2	2	2	2	2	2	1	1	2
2	2	1	1	2	2	2	1	1	2
1	2	2	2	2	2	2	1	1	2
1	2	2	2	2	2	2	1	1	2
	2	2	2	2	2	2	1	2	2
1	2	2	2	2	2	1	1	1	2
2	2	1	2	2	2	2	1	1	2
1	2	2	2	2	2	2	1	1	2
2	2	1	2	2	2	2	1	1	2
1	2	2	2	2	2	2	1	1	2
1	2	2	2	2	2	2	1	1	2
2	2	1	2	2	2	2	1	1	2
1	2	1	1	2	2	2	1	1	2
2	2	1	2	2	2	2	1	1	2
2	2	2	2	2	2	2	1	1	2
2	2	2	2	2	2	2	1	1	2
1	2	2	2	2	2	2	1	1	2
1	2	2	2	2	2	2	1	1	2
1	2	2	2	2	2	2	1	1	2
1	2	2	2	2	2	2	1	1	2
2	2	1	2	2	2	2	1	1	2
1	2	2	2	2	2	2	1	1	2
2	2	2	2	2	2	2	1	1	2
1	2	2	2	2	2	2	1	1	2
2	2	2	2	2	2	2	1	1	2
1	2	2	2	2	2	2	1	1	2
1	2	2	2	2	2	2	1	1	2
2	2	1	2	2	2	2	1	1	2
2	2	1	2	2	2	2	1	1	2
2	2	1	1	2	2	2	1	2	2
1	2	2	2	2	2	2	1	1	2
2	2	1	2	2	2	2	1	1	2
1	2	2	2	2	2	2	1	2	2
1	2	2	2	2	2	2	1	1	2
2	2	2	2	2	2	2	1	1	2
1	2	2	2	2	2	2	1	1	2
1	2	2	2	2	2	2	1	1	2
1	2	2	2	2	2	2	2	0	1
2	2	2	2	2	2	2	1	1	2
1	2	2	2	2	2	2	1	1	2

1	2	2	2	2	2	2	1	1	2
2	2	1	2	2	2	2	1	1	2
1	2	2	2	2	2	2	1	1	2
2	2	1	2	2	2	2	1	1	2
2	2	1	2	2	2	2	1	1	2
2	2	1	2	2	2	2	1	1	2
2	2	1	2	2	2	2	1	1	2
2	2	1	2	2	2	2	1	1	2
2	2	1	2	2	2	2	1	1	2
2	2	1	2	2	2	2	1	2	2
1	2	1	2	2	2	2	1	1	2
2	2	2	2	2	2	2	1	1	2
1	2	2	2	2	2	2	1	1	2
1	2	2	2	2	2	2	1	1	2
2	2	2	2	2	2	2	2	0	1
1	2	2	2	2	2	2	1	1	2
1	2	2	2	2	2	2	2	0	1
1	2	2	2	2	2	2	2	0	1
1	2	2	2	2	2	2	1	1	2
1	1	2	2	2	2	2	1	1	2
1	2	2	2	2	2	2	2	0	1
1	2	2	2	2	2	2	1	2	2
1	1	2	2	2	2	2	1	1	2
1	2	2	2	2	2	2	2	0	1
1	2	2	2	2	2	2	2	0	1
1	2	2	2	2	2	2	1	1	2
2	2	2	2	2	2	2	1	1	2
1	2	2	2	2	2	2	1	1	2
1	2	2	2	2	2	2	2	0	1
1	2	2	2	2	2	2	1	1	2
1	2	1	2	2	2	2	1	1	2
1	2	2	2	2	2	2	1	1	2
2	2	2	1	2	2	2	1	1	2
1	1	2	2	2	2	2	1	1	2
1	2	1	2	2	2	2	1	1	2
2	1	2	2	2	2	2	1	2	2
2	2	1	2	2	2	2	1	2	2
2	2	1	2	2	2	2	1	1	2
1	2	2	2	2	2	2	1	1	2
1	2	2	2	2	2	2	1	1	2
1	2	2	2	2	2	2	1	1	2
2	2	1	2	2	2	2	1	1	2
1	2	2	2	1	2	2	1	1	2
1	2	2	2	1	2	2	1	1	2
1	2	2	2	2	2	2	1	1	2
1	2	1	2	2	2	2	1	1	2
1	2	2	2	2	2	2	1	1	2

1	2	1	2	2	2	2	1	1	2
2	2	2	2	2	2	2	1	1	2
1	2	2	2	2	2	2	1	1	2
1	2	2	2	2	2	2	1	1	2
1	2	2	2	2	2	2	1	1	2
1	2	2	2	2	2	2	1	1	2
1	2	2	2	2	2	2	1	1	2
1	2	2	2	2	2	2	1	1	2
1	2	2	2	2	2	2	1	2	2
1	2	2	2	2	2	2	1	2	2
1	2	2	2	2	2	2	1	2	2
1	2	2	2	2	2	2	1	1	2
1	2	2	2	2	2	2	1	1	2
1	2	2	2	2	2	2	1	2	2
1	2	2	2	2	2	2	1	1	2
1	2	2	2	2	2	2	1	1	2
1	2	2	2	2	2	2	1	1	2
1	2	2	2	2	2	2	1	1	2
1	2	2	2	2	2	2	1	1	2
1	2	2	2	2	2	2	1	1	2
2	2	2	2	2	2	2	1	1	2
1	2	2	2	2	2	2	1	1	2
1	2	2	2	2	2	2	1	1	2
1	2	2	2	2	2	2	1	1	2
1	2	1	1	2	2	2	1	1	2
1	2	2	2	2	2	2	1	1	2
1	2	2	2	2	2	2	1	1	2
1	2	2	2	2	2	2	1	1	2
1	2	2	2	2	2	2	1	1	2
1	2	2	2	2	2	2	1	1	2
2	2	2	2	2	2	2	1	1	2
2	2	2	2	2	2	2	1	1	2
2	2	2	2	2	2	2	1	2	1
1	2	2	2	2	2	2	1	1	2
1	2	2	2	2	2	2	1	1	2
2	2	2	2	2	2	2	1	1	2
1	2	2	2	2	2	2	1	1	2
1	2	2	2	2	2	2	1	1	2
1	2	2	2	2	2	2	1	1	2
1	2	1	1	2	2	2	1	1	2
2	2	2	2	2	2	2	1	1	2
2	2	2	2	2	2	2	1	2	2
1	2	2	2	2	2	2	1	2	2
2	2	2	2	2	2	2	1	1	2
1	2	2	2	2	2	2	1	1	2
1	2	2	2	2	2	2	1	1	2
1	2	2	2	2	2	2	1	1	2

1	2	2	2	2	2	2	1	1	2
1	2	2	2	2	2	2	1	1	2
1	2	2	2	2	2	2	1	1	2
1	2	2	2	2	2	2	1	1	2
1	2	2	2	2	2	2	1	1	2
2	2	2	2	2	2	2	1	1	2
1	2	2	2	2	2	2	1	1	2
1	2	2	2	2	2	2	1	1	2
2	2	2	2	2	2	2	2	2	1
1	2	2	2	2	2	2	1	1	2
1	2	2	2	2	2	2	1	1	2
1	2	2	2	2	2	2	1	1	2
1	2	1	2	2	2	2	1	1	2
1	2	2	2	2	2	2	1	1	2
1	2	2	2	2	2	2	1	1	2
1	2	2	2	2	2	2	1	1	2
1	2	2	2	2	2	2	1	1	2
1	2	2	2	2	2	2	1	1	2
2	2	2	2	2	2	2	1	1	2
1	2	2	2	2	2	2	1	1	2
1	2	2	2	2	2	2	1	2	2
1	2	2	2	2	2	2	1	1	2
1	2	2	2	2	2	2	1	1	2
1	2	2	2	2	2	2	1	1	2
1	2	2	2	2	2	2	2	2	1
1	2	2	2	2	2	2	1	1	2
1	2	2	2	2	2	2	2	2	1
1	2	2	2	2	2	2	1	2	2
1	2	2	2	2	2	2	1	1	2
1	2	2	2	2	2	2	1	1	2
1	2	2	2	2	2	2	1	1	2
1	1	2	2	2	2	2	1	1	2
2	1	1	2	1	2	2	1	1	2
2	2	2	2	2	1	2	1	1	2
1	2	1	1	2	2	2	1	1	2
1	2	2	2	2	2	2	1	1	2
1	1	2	2	2	2	2	1	1	2
1	2	2	2	2	2	2	1	1	2
1	2	2	2	2	2	2	1	1	2
1	2	2	2	2	2	2	1	1	2
1	2	2	1	2	2	2	1	1	2
1	2	2	2	2	2	2	1	1	2
1	2	2	1	2	2	2	1	1	2
2	2	2	2	2	2	2	1	2	2
1	2	2	2	2	2	2	1	1	2
1	2	2	2	2	2	2	1	1	2
1	2	2	2	2	2	2	1	1	2

1	2	2	2	2	2	2	1	1	2
1	2	2	2	2	2	2	1	1	2
2	2	2	2	2	2	2	1	1	2
1	2	2	2	2	2	2	1	1	2
1	2	2	2	2	2	2	1	1	2
1	2	2	2	2	2	2	1	1	2
1	2	2	2	2	1	2	1	1	2
1	2	2	2	2	2	2	1	1	2
1	2	2	2	2	2	2	1	1	2
1	2	2	2	2	2	2	1	1	2
1	2	2	2	2	2	2	1	1	2
1	2	2	2	2	2	2	1	1	2
1	2	2	2	2	1	1	1	1	2
1	2	2	2	2	2	2	1	1	2
1	2	2	2	2	1	1	1	1	2
1	2	2	2	2	2	2	1	1	2
1	2	1	1	2	1	1	1	1	2
1	2	2	2	2	2	2	1	1	2
2	2	2	2	2	2	2	1	1	2
1	2	1	1	2	1	1	1	1	2
1	2	2	1	2	2	2	1	1	2
2	2	2	2	2	2	2	1	1	2
2	2	1	1	2	2	2	1	1	2
1	2	2	2	2	2	2	1	1	2
1	2	2	2	2	2	2	1	1	2
1	2	2	2	2	2	2	1	1	2
1	2	2	2	2	2	2	1	1	2
1	2	2	2	2	2	2	1	1	2
1	2	2	2	2	2	2	1	1	2
1	2	2	2	2	2	2	1	1	2
1	2	2	2	2	2	2	1	1	2
1	2	2	2	2	2	2	1	1	2
1	2	2	2	2	2	2	2	2	1
1	2	2	2	2	2	2	1	2	2
1	2	2	2	2	2	2	1	1	2
1	2	2	2	2	2	2	1	1	2
1	2	2	2	2	2	2	1	2	2
1	2	2	2	2	2	2	1	2	2
1	2	2	2	2	2	2	1	1	2
2	2	2	2	2	2	2	1	1	2
1	2	2	2	2	2	2	1	1	2
1	2	2	2	2	2	2	1	1	2
1	2	2	2	2	2	2	2	2	1
1	2	2	2	2	2	2	1	1	2
1	2	2	2	2	2	2	1	1	2
1	2	2	2	2	2	2	2	2	1
1	2	1	1	2	1	2	1	1	2

1	2	2	2	2	2	2	1	2	2
1	2	2	2	2	1	2	1	2	2
1	2	2	2	2	2	2	1	2	2
1	2	2	2	2	2	2	1	2	2
1	2	2	2	2	2	2	1	1	2
1	2	2	2	2	2	2	1	1	2
1	2	2	2	2	2	2	1	1	2
1	2	2	2	2	2	2	1	1	2
2	2	2	2	2	2	2	1	1	2
1	2	2	2	2	2	2	1	1	2
1	2	2	2	2	2	2	1	1	2
2	2	2	2	2	2	2	1	1	2
1	2	2	2	2	2	2	1	1	2
1	2	2	2	2	2	2	1	1	2
1	2	2	2	2	2	2	1	1	2
2	2	2	2	2	2	2	1	1	2
2	2	1	1	2	2	2	1	1	2
2	2	2	2	2	2	2	1	1	2
1	2	2	2	2	2	2	1	1	2
1	2	2	2	2	2	2	1	1	2
2	2	1	2	2	2	2	1	1	2
1	2	2	2	2	2	2	1	1	2
1	1	2	2	2	2	2	1	1	2
1	2	2	2	2	2	2	1	1	2
1	2	2	2	2	2	2	1	1	2
2	2	2	2	2	2	2	1	1	2
1	2	2	2	2	2	2	1	1	2
2	2	1	2	2	2	2	1	2	2
1	2	2	2	2	2	2	1	1	2
2	2	1	1	2	1	2	1	1	2
1	2	2	2	2	2	2	1	1	1
1	2	2	2	2	2	2	1	1	2
2	2	1	2	2	2	2	1	2	2
1	2	2	2	2	2	2	1	1	2
1	2	2	2	2	2	2	1	1	2
1	2	2	2	2	2	2	1	1	2
2	2	2	2	2	2	2	1	1	2
1	2	2	2	2	2	2	1	1	2
1	2	2	2	2	2	2	1	1	2
1	2	2	2	2	2	2	2	2	1
1	2	2	2	2	2	2	1	1	2
1	2	2	2	2	2	2	1	1	2
1	2	2	2	2	2	2	1	1	2
1	2	2	2	2	2	2	1	1	2
1	2	2	2	2	2	2	1	1	2
1	2	2	2	2	2	2	1	1	2
1	2	2	2	2	2	2	1	1	2

1	2	2	2	2	2	2	1	1	2
1	2	2	2	2	2	2	1	1	2
1	2	2	2	2	2	2	1	1	2
1	2	2	2	2	2	2	1	1	2
2	2	2	2	2	2	2	1	2	2
1	2	2	2	2	2	2	1	1	2
1	2	2	2	2	2	2	1	1	2
1	2	2	2	2	2	2	1	1	2
1	2	2	2	2	2	2	1	1	2
2	2	2	2	2	2	2	1	1	2
2	2	2	2	2	2	2	1	1	2
1	2	2	2	2	2	2	1	1	2
1	2	2	2	2	2	2	1	1	2
1	2	2	2	2	2	2	1	1	2
1	2	2	2	2	2	2	2	2	1
2	2	2	1	2	2	2	1	1	2
1	2	2	2	2	2	2	1	1	2
1	2	2	2	2	2	2	1	1	2
1	2	2	2	2	2	2	1	1	2
1	2	2	2	2	2	2	1	1	2
2	2	2	2	2	2	2	1	1	2
2	2	2	2	2	2	2	1	1	2
1	2	2	2	2	2	2	1	1	2
1	2	2	2	2	2	2	1	1	2
1	2	2	2	2	2	2	1	1	2
1	2	2	2	2	2	2	1	1	2
1	2	2	2	2	2	2	1	1	2
2	2	1	2	2	2	2	1	1	2
1	1	2	2	2	2	2	1	1	2
2	2	1	2	2	2	2	1	1	2
1	1	2	2	2	2	2	2	2	1
2	2	1	2	2	2	2	1	1	2
1	1	2	2	2	2	2	1	1	2
1	2	1	2	2	2	2	1	1	2
1	2	1	2	2	2	2	1	1	2
1	2	2	2	2	2	2	1	1	2
2	2	1	2	2	2	2	1	1	2
1	2	2	2	2	2	2	2	2	1
1	2	2	2	2	2	2	1	2	2
2	1	2	2	2	2	2	1	1	2
1	1	2	2	2	2	2	1	1	2
1	2	2	2	2	2	2	1	2	2
1	1	2	2	2	2	2	1	1	2
1	2	2	2	2	2	2	1	1	2
1	2	2	2	2	2	2	1	1	2
1	2	2	2	2	2	2	1	1	2

Firewood	coal/charc	Electricity	Gas	Paraffin	Gel	cow dung	solar	Radio	TV
1	2	1	2	2	2	2	2	1	1
1	2	1	2	2	2	2	2	1	1
1	2	1	2	2	2	2	2	2	1
1	2	2	2	2	2	2	2	1	1
1	2	2	2	2	2	2	2	2	1
1	2	2	2	2	2	2	2	1	1
1	2	1	2	2	2	2	2	1	1
1	2	1	2	2	2	2	2	1	1
1	2	1	2	2	2	2	2	2	1
1	2	2	2	2	2	1	2	1	1
1	2	1	2	2	2	2	2	1	1
1	2	1	1	2	2	2	2	1	1
2	2	1	2	1	2	2	2	1	1
1	2	1	2	2	2	2	2	1	1
1	1	2	2	2	2	2	2	2	1
1	2	1	2	2	2	2	2	1	1
1	2	1	2	2	2	2	2	1	1
1	2	1	2	2	2	2	2	1	1
1	2	1	2	2	2	2	2	1	1
1	2	1	2	2	2	2	2	1	1
2	2	2	2	2	2	2	2	2	1
1	2	1	2	2	2	2	2	1	1
1	2	1	2	2	2	2	2	1	1
2	1	2	2	2	2	2	2	1	1
1	2	1	2	2	2	2	2	1	1
1	2	1	2	2	2	2	2	1	1
1	2	1	2	2	2	2	2	2	1
1	2	1	2	2	2	2	2	2	1
1	2	2	2	2	2	2	2	2	2
1	2	2	2	2	2	2	2	1	1
1	2	2	2	2	2	2	2	2	2
1	2	1	2	2	2	2	2	1	1
1	2	1	2	2	2	2	2	1	1
1	2	2	2	2	2	2	2	1	1
1	2	1	2	2	2	2	2	2	1
1	2	1	2	2	2	2	2	1	2
1	2	1	2	2	2	2	2	1	1
1	2	2	2	2	2	2	2	2	1
1	2	2	2	2	2	2	2	2	1
1	2	1	2	2	2	2	2	1	1
1	2	1	2	2	2	2	2	2	2
1	2	1	2	2	2	2	2	1	1
1	2	2	2	2	2	2	2	1	1
1	2	2	2	2	2	2	2	1	1
1	2	1	2	2	2	2	2	1	1
1	2	2	2	2	2	2	2	1	1

1	2	2	2	2	2	2	2	2	1
1	2	1	2	2	2	2	2	2	1
1	2	2	2	2	2	2	2	1	1
1	2	1	2	2	2	2	2	1	1
1	2	2	2	2	2	2	2	1	1
1	2	1	2	2	2	2	2	2	1
1	2	1	2	2	2	2	2	2	1
1	2	1	2	2	2	2	2	2	1
1	2	1	2	2	2	2	2	1	1
1	2	1	2	2	2	2	2	2	2
1	2	1	2	2	2	2	2	2	1
1	2	1	2	2	2	2	2	1	1
1	2	1	2	2	2	2	2	1	1
1	2	1	2	2	2	2	2	1	1
1	2	1	2	2	2	2	2	1	1
1	2	2	2	2	2	2	2	1	1
1	2	1	2	2	2	2	2	1	1
1	2	1	2	2	2	1	2	2	1
1	2	1	2	2	2	2	2	1	1
1	2	1	2	2	2	2	2	1	1
1	2	1	2	2	2	2	2	1	1
1	2	1	2	2	2	2	2	1	1
1	2	1	2	2	2	2	2	1	1
1	2	1	2	2	2	2	2	1	1
1	2	1	2	2	2	2	2	1	1
1	2	2	2	2	2	2	2	1	1
1	2	2	2	2	2	2	2	1	1
1	2	1	2	2	2	2	2	2	1
1	2	1	2	2	2	2	2	2	2
1	2	2	2	2	2	2	2	1	1
1	2	2	2	2	2	2	2	2	2
1	2	2	2	2	2	2	2	2	2
1	2	1	2	2	2	2	2	1	1
1	2	1	2	2	2	1	2	1	1
1	2	1	2	2	2	2	2	1	1
1	2	2	2	2	2	2	2	1	1
1	2	1	2	2	2	2	2	1	1
1	2	2	2	2	2	2	2	1	1
1	2	1	2	2	2	2	2	2	1
1	2	2	2	2	2	2	2	2	1
1	2	1	2	2	2	2	2	1	1
1	2	1	2	2	2	2	2	1	1
1	2	1	2	2	2	2	2	1	1
1	2	1	2	2	2	2	2	2	1
1	2	1	2	2	2	2	2	1	1
1	2	1	2	2	2	2	2	1	1
1	2	1	2	2	2	2	2	1	1

1	2	2	2	2	2	2	2	1	1
1	2	2	2	2	2	2	2	2	1
1	2	2	2	2	2	2	2	1	1
1	2	1	2	2	2	2	2	1	1
1	2	1	2	2	2	2	2	2	1
1	2	2	2	2	2	2	2	1	2
1	2	1	2	2	2	2	2	2	1
1	2	2	2	2	2	2	2	1	1
1	2	2	2	2	2	2	2	1	1
1	2	1	2	2	2	2	2	2	1
1	2	2	2	2	2	2	2	1	1
1	2	1	2	2	2	2	2	2	1
1	2	1	2	2	2	2	2	1	1
2	2	1	2	2	2	2	2	1	1
1	2	2	2	2	2	2	2	1	1
1	2	2	2	2	2	2	2	1	1
1	2	2	2	2	2	2	2	2	1
1	2	2	2	2	2	2	2	1	1
1	2	1	2	2	2	2	2	1	1
1	2	1	2	2	2	2	2	1	1
1	2	2	2	2	2	2	2	2	1
1	2	1	2	2	2	2	2	1	1
1	2	1	2	2	2	2	2	1	1
1	2	1	2	2	2	2	2	1	1
1	2	1	2	2	2	2	2	1	1
1	2	1	2	2	2	2	2	1	1
1	2	1	2	2	2	2	2	1	1
1	2	2	2	2	2	2	2	2	1
1	2	1	2	2	2	2	2	2	1
1	2	1	2	2	2	2	2	1	1
1	2	1	2	2	2	2	2	1	1
1	2	1	2	2	2	2	2	1	1
1	2	1	2	2	2	2	2	2	1
1	2	1	2	2	2	2	2	1	1
1	2	1	2	2	2	2	2	1	1
1	2	2	2	2	2	2	2	2	1
1	2	1	2	2	2	2	2	2	1
1	2	1	2	2	2	2	2	2	1
1	2	1	2	2	2	2	2	1	1
1	2	2	2	2	2	2	2	2	1
1	2	1	1	2	2	2	2	1	1
1	2	1	2	2	2	2	2	1	1
1	2	1	2	2	2	2	2	1	1
1	2	2	2	2	2	2	2	2	1
1	2	1	2	2	2	2	2	1	1
2	2	1	2	2	2	2	2	2	1
1	2	1	2	2	2	2	2	1	1

1	2	2	2	2	2	2	2	1	1
1	2	2	2	2	2	2	2	2	2
1	2	2	2	2	2	2	2	2	2
1	2	2	2	2	2	2	2	2	2
1	2	2	2	2	2	2	2	1	1
1	2	1	2	2	2	2	2	1	1
1	2	2	2	2	2	2	2	2	2
1	2	1	2	2	2	2	2	2	1
1	2	1	2	2	2	2	2	1	1
1	2	1	2	2	2	2	2	2	1
1	2	1	2	2	2	2	2	1	1
1	2	1	2	2	2	2	2	1	1
1	2	2	2	2	2	2	2	1	1
1	2	1	2	2	2	2	2	1	1
1	2	1	2	2	2	2	2	2	1
1	2	1	2	2	2	2	2	1	1
1	2	1	2	2	2	2	2	1	1
1	2	1	2	2	2	2	2	1	1
1	2	1	2	2	2	2	2	2	1
1	2	1	2	2	2	2	2	1	2
1	2	1	2	2	2	2	2	1	1
1	2	1	2	2	2	2	2	1	1
1	2	1	2	2	2	2	2	1	1
2	2	1	2	2	2	2	2	2	1
2	2	1	2	2	2	2	2	2	1
1	2	1	2	2	2	2	2	2	1
1	2	1	2	2	2	2	2	1	1
1	2	1	2	2	2	2	2	1	1
1	2	1	2	2	2	2	2	2	2
1	2	1	2	2	2	2	2	2	1
1	2	1	2	2	2	2	2	1	1
2	2	1	2	2	2	2	2	2	1
2	2	1	2	2	2	2	2	1	1
1	2	1	2	2	2	2	2	2	1
1	2	2	2	2	2	2	2	2	2
1	2	1	2	2	2	2	2	2	1
1	2	1	2	2	2	2	2	2	1
1	2	1	2	2	2	2	2	1	1
1	2	1	2	2	2	2	2	1	1
1	2	1	2	2	2	2	2	1	1
1	2	2	2	2	2	2	2	1	1
1	2	1	2	2	2	2	2	1	1
1	2	2	2	2	2	2	2	1	1
1	2	1	2	2	2	2	2	1	1
1	2	1	2	2	2	2	2	1	1
1	2	1	2	2	2	2	2	2	2
1	2	1	2	2	2	2	2	2	1

1	2	2	2	2	2	2	2	2	2
1	2	1	2	2	2	2	2	1	1
1	2	2	2	2	2	2	2	1	1
1	2	1	2	2	2	2	2	1	1
1	2	1	2	2	2	2	2	1	1
1	2	1	2	2	2	2	2	1	1
1	2	1	2	2	2	2	2	1	1
1	2	1	2	2	2	2	2	1	1
1	2	2	2	2	2	2	2	2	2
1	2	2	2	2	2	2	2	2	1
1	2	1	2	2	2	2	2	1	1
1	2	1	2	2	2	2	2	1	1
1	2	1	2	2	2	2	2	2	1
1	2	2	2	2	2	2	2	2	1
1	2	2	2	2	2	2	2	2	1
1	2	2	2	2	2	2	2	2	2
1	2	1	2	2	2	2	2	1	1
1	2	1	2	2	2	2	2	2	1
1	2	2	2	2	2	2	2	2	1
1	2	1	1	2	2	2	2	1	1
1	2	2	2	2	2	2	2	2	1
1	2	1	2	2	2	2	2	2	1
1	2	1	2	2	2	2	2	2	1
1	2	2	2	2	2	2	2	1	1
1	2	1	2	2	2	2	2	1	1
1	2	2	2	2	2	2	2	1	1
1	2	2	2	2	2	2	2	2	2
1	2	1	2	2	2	2	2	1	1
1	2	1	2	2	2	2	2	1	1
1	2	1	2	2	2	2	2	2	1
1	2	1	2	2	2	2	2	2	1
1	2	1	2	2	2	2	2	2	2
1	2	1	2	2	2	2	2	1	1
1	2	1	2	2	2	2	2	2	1
1	2	2	2	2	2	2	2	2	1
1	2	1	2	2	2	2	2	1	1
1	2	1	2	2	2	2	2	2	1
1	2	1	2	2	2	2	2	2	1
1	2	1	2	2	2	2	2	1	1
1	2	1	2	2	2	2	2	1	1
1	2	1	2	2	2	2	2	2	1
1	2	1	2	2	2	2	2	1	1
1	2	2	2	2	2	2	2	1	1
1	2	2	2	2	2	2	2	1	1
1	2	1	2	2	2	2	2	2	2
1	2	1	2	2	2	2	2	2	1
1	2	1	2	2	2	2	2	1	1

1	2	1	2	2	2	2	2	1	1
2	2	1	2	2	2	2	2	1	1
1	2	1	2	2	2	2	2	1	1
1	2	1	2	2	2	2	2	1	1
1	2	1	2	2	2	2	2	1	1
1	2	1	2	2	2	2	2	1	1
1	2	1	2	2	2	2	2	1	1
1	2	1	2	2	2	2	2	1	1
1	2	1	2	2	2	2	2	1	1
1	2	1	2	2	2	2	2	1	1
1	2	1	2	2	2	2	2	1	1
1	2	2	2	2	2	2	2	2	2
1	2	1	2	2	2	2	2	1	1
1	2	1	2	2	2	2	2	1	1
1	2	2	2	2	2	2	2	2	1
1	2	2	2	2	2	2	2	2	2
1	2	2	2	2	2	2	2	2	2
1	2	2	2	2	2	2	2	2	2
1	2	2	2	2	2	2	2	2	1
1	2	1	2	2	2	2	2	2	1
1	2	2	2	2	2	2	2	2	2
1	2	1	2	2	2	2	2	2	1
1	2	1	2	2	2	2	2	1	1
1	2	2	2	2	2	2	2	1	2
1	2	2	2	2	2	2	2	1	2
1	2	1	2	2	2	2	2	1	1
1	2	2	2	2	2	2	2	2	1
1	2	1	2	2	2	2	2	1	1
1	2	1	2	2	2	2	2	2	2
1	2	1	2	2	2	2	2	1	1
1	2	1	2	2	2	2	2	1	1
1	2	2	2	2	2	2	2	1	1
1	2	1	2	2	2	2	2	1	1
1	2	1	2	2	2	2	2	1	1
1	2	1	2	2	2	2	2	1	1
1	2	2	2	2	2	2	2	1	1
1	2	2	2	2	2	2	2	1	1
1	2	1	2	2	2	2	2	2	1
1	2	1	2	2	2	2	2	1	1
1	2	1	2	2	2	2	2	1	2
1	2	2	2	2	2	2	2	1	1
1	2	2	2	2	2	2	2	1	1
1	2	1	2	2	2	2	2	2	1
1	2	1	2	2	2	2	2	2	1
1	2	1	2	2	2	2	1	1	1
1	2	1	2	2	2	2	2	2	1
1	2	2	2	2	2	2	2	1	1

1	2	1	2	2	2	2	2	1	1
1	2	2	2	2	2	2	2	2	2
1	2	2	2	2	2	2	2	2	1
1	2	1	2	2	2	2	2	1	1
1	2	2	2	2	2	2	2	1	1
1	2	1	2	2	2	2	2	1	1
1	2	2	2	2	2	2	2	2	1
1	2	1	2	2	2	2	2	1	1
1	2	2	2	2	2	2	2	1	1
1	2	2	2	2	2	2	2	1	1
1	2	2	2	2	2	2	2	1	1
1	2	2	2	2	2	2	2	2	1
1	2	1	2	2	2	2	2	1	1
1	2	1	2	2	2	2	2	1	1
1	2	1	2	2	2	2	2	1	1
1	2	1	2	2	2	2	2	2	1
1	2	1	2	2	2	2	2	1	1
1	2	1	2	2	2	2	2	1	1
1	2	1	2	2	2	2	2	1	1
2	2	2	2	2	2	2	2	2	1
1	2	2	2	2	2	2	2	2	1
1	2	1	2	2	2	2	2	1	1
1	2	1	2	2	2	2	2	1	1
1	2	2	2	2	2	2	2	2	2
1	2	1	2	2	2	2	2	1	1
1	2	2	2	2	2	2	2	1	1
1	2	1	2	2	2	2	2	1	1
1	2	2	2	2	2	2	2	2	1
1	2	2	2	2	2	2	2	1	1
1	2	2	2	2	2	2	2	1	1
1	2	1	1	2	2	2	2	1	1
1	2	1	2	2	2	2	2	2	2
2	1	2	2	2	2	2	2	1	1
1	2	1	2	2	2	2	2	1	1
1	2	1	2	2	2	2	2	1	1
1	2	2	2	2	2	2	2	1	1
1	2	2	2	2	2	2	2	2	1
1	2	1	2	2	2	2	2	1	1
1	2	2	2	2	2	2	2	1	1
1	2	1	2	2	2	2	2	1	1
1	2	2	2	2	2	2	2	1	1
1	2	2	2	2	2	2	2	1	1
1	2	1	2	2	2	2	2	1	1
1	2	1	2	2	2	2	2	1	1
1	2	1	2	2	2	2	2	2	1
1	2	1	2	2	2	2	2	1	2
1	2	1	2	2	2	2	2	1	1

1	2	1	2	2	2	2	2	1	1
1	2	1	2	2	2	2	2	1	1
1	2	2	2	2	2	2	2	2	1
1	2	2	2	2	2	2	2	1	1
1	2	2	2	2	2	2	2	1	1
1	2	1	2	2	2	2	2	1	1
1	2	1	2	2	2	2	2	1	1
1	2	1	2	2	2	2	2	1	1
1	2	1	2	2	2	2	2	2	2
1	2	2	2	2	2	2	2	1	1
1	2	2	2	2	2	2	2	1	1
1	2	1	2	2	2	2	2	1	1
1	2	1	2	2	2	2	2	1	1
1	2	1	2	2	2	2	2	1	2
1	2	1	2	2	2	2	2	2	2
1	2	1	2	2	2	2	2	2	2
1	2	1	2	2	2	2	2	2	2
1	2	1	2	2	2	2	2	1	1
1	2	2	2	2	2	2	2	1	1
1	2	1	2	2	2	2	2	1	1
1	2	2	2	2	2	2	2	1	2
1	2	1	2	2	2	2	2	1	1
1	2	1	2	2	2	2	2	1	1
1	2	2	2	2	2	2	2	2	1
1	2	2	2	2	2	2	2	1	1
1	2	1	2	2	2	2	2	2	2
1	2	1	2	2	2	2	2	1	1
1	2	1	2	2	2	2	2	1	1
1	2	1	2	2	2	2	2	1	1
1	2	1	2	2	2	2	2	1	1
1	2	1	2	2	2	2	2	2	1
1	2	1	2	2	2	2	2	2	1
1	2	2	2	2	2	2	2	2	1
1	2	1	2	2	2	2	2	1	1
1	2	1	2	2	2	2	2	1	1
1	2	2	2	2	2	2	2	1	2
1	2	2	2	2	2	2	2	1	1
1	2	2	2	2	2	2	2	1	1
1	2	2	2	2	2	2	2	1	1
1	2	2	2	2	2	2	2	1	1
1	2	2	2	2	2	2	2	1	1
1	2	1	2	2	2	2	2	1	1
1	2	2	2	2	2	2	2	1	1
1	2	2	2	2	2	2	2	1	1
1	2	2	2	2	2	2	2	2	1
1	2	1	2	2	2	2	2	1	1
1	2	1	2	2	2	2	2	1	1

1	2	1	2	2	2	2	2	1	1
1	2	2	2	2	2	2	2	1	1
1	2	2	2	2	2	2	2	1	1
1	2	2	2	2	2	2	2	1	1
1	2	2	2	2	2	2	2	1	1
1	2	2	2	2	2	2	2	1	1
1	2	1	2	2	2	2	2	1	1
1	2	1	2	2	2	2	2	2	1
1	2	2	2	2	2	2	2	2	1
1	2	1	2	2	2	2	2	1	1
1	2	1	2	2	2	2	2	1	1
1	2	1	2	2	2	2	2	1	1
1	2	1	2	2	2	2	2	2	1
1	2	1	2	2	2	2	2	1	1
1	2	1	2	2	2	2	2	2	1
1	2	1	2	2	2	2	2	2	1
1	2	1	1	2	2	2	2	2	1
1	2	2	2	2	2	2	2	1	1
1	2	2	2	2	2	2	2	2	1
1	2	1	1	2	2	2	2	2	1
1	2	1		2	2	2	2	1	1
1	2	1	2	2	2	2	2	1	1
1	2	1	2	2	2	2	2	2	1
1	2	1	2	2	2	2	2	2	1
1	2	1	2	2	2	2	2	1	1
1	2	1	2	2	2	2	2	2	1
1	2	2	2	2	2	2	2	1	1
1	2	2	2	2	2	2	2	2	2
1	2	1	2	2	2	2	2	1	1
1	2	1	2	2	2	2	2	1	1
1	2	1	2	2	2	2	2	1	1
1	2	1	2	2	2	2	2	1	1
1	2	2	2	2	2	2	2	2	2
1	2	1	2	2	2	2	2	2	1
1	2	1	2	2	2	2	2	1	1
1	2	1	2	2	2	2	2	1	1
1	2	1	2	2	2	2	2	1	1
1	2	1	2	2	2	2	2	1	1
1	2	1	2	2	2	2	2	1	1
1	2	1	2	2	2	2	2	1	1
1	2	2	2	2	2	2	2	1	2
1	2	1	2	2	2	2	2	1	1
1	2	2	2	2	2	2	2	2	2
1	2	1	2	2	2	2	2	1	1
1	2	1	2	2	2	2	2	1	1
1	2	1	2	2	2	2	2	1	2
1	2	1	2	2	2	2	2	1	1

1	2	1	2	2	2	2	2	1	1
2	2	2	2	2	2	2	2	1	1
2	2	1	2	2	2	2	2	1	1
2	2	1	2	2	2	2	2	1	1
1	2	1	2	2	2	2	2	1	1
1	2	1	2	2	2	2	2	1	1
1	2	1	2	2	2	2	2	1	1
1	2	1	2	2	2	2	2	2	1
1	2	2	1	2	2	2	2	2	1
1	2	1	2	2	2	2	2	1	1
1	2	1	2	2	2	2	2	2	1
1	2	1	2	2	2	2	2	1	1
1	2	1	2	2	2	2	2	2	1
1	2	1	2	2	2	2	2	2	2
1	2	1	2	2	2	2	2	1	1
1	2	1	2	2	2	2	2	1	1
1	2	1	2	2	2	2	2	1	1
1	2	2	2	2	2	2	2	1	1
1	2	1	2	2	2	2	2	1	1
1	2	2	2	2	2	2	2	1	1
1	2	1	2	2	2	2	2	2	1
1	2	2	2	2	2	2	2	2	1
1	2	2	2	2	2	2	2	1	1
1	2	1	2	2	2	2	2	2	2
1	2	1	2	2	2	2	2	1	1
1	2	2	2	2	2	2	2	2	2
1	2	1	2	2	2	2	2	1	1
1	2	1	2	2	2	2	2	1	1
1	2	1	2	2	2	2	2	1	1
1	2	1	2	2	2	2	2	2	1
1	2	1	2	2	2	2	2	1	1
1	2	1	2	2	2	2	2	2	1
1	2	1	2	1	2	2	2	1	1
1	2	1	2	2	2	2	2	1	1
1	2	2	2	2	2	2	2	2	1
1	2	2	2	2	2	2	2	2	1
1	2	2	2	2	2	2	2	1	1
1	2	1	2	2	2	2	2	2	1
1	2	1	2	2	2	2	2	1	1
1	2	2	2	2	2	2	2	2	2
1	2	1	2	2	2	2	2	1	1
1	2	1	2	2	2	2	2	1	1
1	2	2	2	2	2	2	2	1	1
2	2	1	2	2	2	2	2	1	1
1	2	1	2	2	2	2	2	1	1
1	2	2	2	2	2	2	2	2	2
1	2	1	2	2	2	2	2	1	1

1	2	2	2	2	2	2	2	1	1
1	2	1	2	2	2	2	2	2	1
1	2	1	2	2	2	2	2	1	1
1	2	1	2	2	2	2	2	2	2
1	2	1	2	2	2	2	2	1	1
1	2	1	2	2	2	2	2	1	1
1	2	1	2	2	2	2	2	2	1
1	2	1	2	2	2	2	2	1	1
1	2	1	2	2	2	2	2	1	1
1	2	1	2	2	2	2	2	1	1
2	2	1	2	2	2	2	2	2	1
1	2	1	2	2	2	2	2	1	1
1	2	2	2	2	2	2	2	2	1
1	2	2	2	2	2	2	2	2	1
1	2	1	2	2	2	2	2	2	1
2	2	1	2	2	2	2	2	2	1
1	2	1	2	2	2	2	2	1	1
1	2	1	2	2	2	2	2	1	1
1	2	1	2	2	2	2	2	1	2
1	2	1	2	2	2	2	2	2	1
1	2	1	2	2	2	2	2	1	1
2	2	1	2	2	2	2	2	2	1
1	2	2	2	2	2	2	2	1	1
1	2	1	2	2	2	2	2	1	1
1	2	2	2	2	2	2	2	2	1
1	2	2	2	2	2	2	2	1	1
1	2	2	2	2	2	2	2	2	1
1	2	1	2	2	2	2	2	2	1
1	2	2	2	2	2	2	2	2	2
1	2	1	2	2	2	2	2	1	1
1	2	2	2	2	2	2	2	2	2
1	2	1	2	2	2	2	2	2	1
1	2	2	2	2	2	2	2	1	1
1	2	2	2	2	2	2	2	2	2
1	2	2	2	2	2	2	2	2	2
1	2	1	2	2	2	2	2	1	1
1	2	1	2	2	2	2	2	2	1
1	2	1	2	2	2	2	2	1	1
1	2	1	2	2	2	2	2	1	1
1	2	1	2	2	2	2	2	2	1
1	2	1	2	2	2	2	2	1	1
1	2	2	2	2	2	2	2	1	1
1	2	1	2	2	2	2	2	1	1
1	2	2	2	2	2	2	2	1	1
1	2	2	2	2	2	2	2	1	2
1	2	1	2	2	2	2	2	1	1

DSTV	Video/DVI	House Tel	Stove	Microwave	Dining tabl	Sofas	Play statio	Local form	spaza shop
1	1	2	1	1	1	2	2	1	1
1	1	2	1	1	1	2	2	1	1
1	2	2	1	1	2	1	2	1	1
1	2	2	2	2	2	2	2	1	1
1	2	2	2	2	1	2	2	2	2
1	2	2	1	1	2	1	2	1	1
1	1	2	1	2	2	2	2	1	1
1	1	2	1	1	2	1	2	1	1
1	1	2	1	2	1	2	2	1	1
1	2	2	1	2	1	2	2	1	1
1	1	2	1	1	1	2	2	1	1
1	2	2	1	1	1	1	2	1	1
1	1	2	1	1	2	1	2	1	1
1	1	1	1	1	2	1	2	2	1
1	2	2	1	2	1	1	2	1	1
1	1	2	1	2	2	1	2	1	1
1	1	2	1	2	2	1	2	1	1
1	2	2	1	1	2	1	2	1	1
1	1	2	1	1	2	2	2	1	1
1	2	2	1	1	1	2	2	1	1
1	1	2	2	2	2	2	2	1	1
1	2	1	1	1	1	1	2	1	1
1	1	2	1	1	1	1	2	1	1
1	2	2	1	2	2	2	2	1	1
1	1	2	1	2	2	2	2	1	1
1	2	2	1	2	2	2	2	1	1
1	2	2	1	2	1	1	2	1	1
2	2	2	1	2	1	1	2	1	1
2	2	2	2	2	1	2	2	1	1
2	2	2	2	2	1	1	2	1	1
2	2	2	2	2	1	2	2	2	1
1	2	2	1	1	2	1	2	1	1
1	2	2	1	2	2	2	2	1	1
1	1	1	1	2	1	2	1	1	1
1	2	2	1	2	2	2	2	1	1
2	2	2	1	2	2	1	2	1	1
1	2	2	1	2	1	1	2	1	1
2	2	2	2	2	1	2	2	1	1
1	2	2	2	2	1	1	2	1	1
1	2	1	1	1	1	2	2	1	1
2	2	2	1	2	2	2	2	1	1
2	2	2	2	1	2	2	2	1	1
2	1	2	2	2	2	2	2	1	1
2	1	2	1	2	1	2	2	1	2
2	1	2	1	1	2	2	2	1	1
1	2	2	2	2	2	2	2	1	1

1	2	1	2	2	2	2	2	1	1
2	1	2	2	2	1	2	2	1	1
1	1	2	2	2	1	1	2	1	1
1	1	2	1	1	1	1	2	1	1
2	2	2	2	2	1	1	2	1	1
1	2	2	1	2	2	1	2	1	1
2	1	2	1	2	2	2	2	1	1
1	1	2	1	2	2	1	2	1	1
1	1	2	1	1	2	1	2	1	1
2	2	2	1	2	2	2	2	1	1
1	1	2	1	2	2	2	2	1	1
1	1	2	1	2	2	2	2	1	1
1	1	2	1	1	2	2	2	1	1
1	2	2	1	2	2	1	2	1	1
1	1	2	1	1	1	1	2	1	1
1	1	1	1	2	1	2	2	1	1
1	1	2	1	1	1	1	2	1	1
1	2	2	1	2	1	1	2	1	1
2	1	2	1	2	2	2	2	1	2
1	1	2	1	2	2	2	2	2	1
1	1	2	1	1	2	1	2	1	1
1	1	2	1	2	1	2	2	1	1
1	1	2	1	2	1	2	2	1	1
1	2	2	1	2	2	2	2	2	2
1	2	2	1	2	2	2	2	2	2
1	2	2	2	2	2	1	2	1	1
1	1	2	2	2	1	1	2	2	1
1	2	1	1	1	2	2	2	1	1
2	2	2	1	2	2	2	2	2	1
1	1	2	2	1	2	1	2	1	1
2	2	2	1	2	2	2	2	1	1
2	2	2	1	2	2	2	2	1	1
2	2	2	1	2	2	2	2	1	1
1	2	2	1	1	1	1	2	1	1
1	1	2	1	1	1	1	1	1	1
1	1	2	1	2	1	2	2	1	1
1	1	2	1	2	1	2	2	1	1
1	1	1	2	1	1	1	2	1	1
1	2	2	1	2	2	2	2	1	1
1	2	2	2	2	2	2	2	1	1
1	2	2	1	1	1	1	2	2	1
2	1	2	1	2	2	2	2	2	1
1	2	2	1	2	2	1	2	2	1
1	2	2	1	2	2	2	2	2	1
1	2	2	1	1	2	1	2	2	1
1	2	2	1	1	2	1	2	2	1
1	1	2	1	1	1	1	2	1	1

2	1	1	2	2	2	2	2	1	1
1	2	2	2	2	1	1	2	1	1
1	1	2	1	1	1	1	2	1	1
1	1	2	1	1	1	1	2	1	1
2	2	2	1	2	2	2	2	1	1
2	2	2	2	2	2	2	2	1	1
1	1	2	1	1	1	1	2	1	1
1	1	2	2	1	1	1	2	2	1
2	1	2	2	2	2	2	2	1	2
1	1	2	1	1	2	2	2	1	1
1	2	2	2	2	2	2	2	1	1
1	2	2	1	2	1	2	2	1	1
1	1	2	1	1	2	2	2	1	1
1	1	2	1	1	1	1	2	1	1
1	2	2	2	2	2	2	2	1	1
1	2	2	2	1	1	2	2	1	1
1	1	2	2	2	2	1	2	1	1
1	2	2	2	2	2	2	2	1	2
1	1	2	1	2	2	1	2	1	2
1	1	2	1	2	1	1	2	1	1
1	1	2	1	2	2	2	2	1	1
1	2	2	1	2	2	2	2	1	1
1	2	2	1	2	1	2	2	1	1
1	2	2	1	2	1	2	2	1	1
1	2	2	1	2	1	2	2	1	1
1	2	2	1	1	2	1	2	1	1
1	2	2	1	2	1	1	2	1	1
1	1	2	1	1	2	1	2	2	1
1	2	2	1	2	1	2	2	1	1
1	1	2	1	2	2	2	2	1	1
1	1	2	1	1	1	1	2	1	1
2	2	2	1	1	2	1	2	1	1
1	1	2	1	2	1	2	2	1	1
1	2	2	1	1	1	1	2	1	1
1	2	2	1	1	1	1	2	1	1
1	2	2	1	1	1	1	2	1	1
1	2	2	1	1	1	1	2	1	1
1	1	2	1	1	1	1	2	1	1
1	2	2	1	2	1	2	2	1	1
1	2	2	1	2	1	2	2	1	1
1	1	2	1	1	1	1	2	1	1
1	2	2	1	2	2	1	2	1	2
1	2	2	1	2	2	1	2	1	1
1	2	2	2	2	1	2	2	1	1
1	2	2	1	2	2	2	2	1	1
1	2	2	1	2	1	1	2	1	1
1	1	2	1	1	1	1	2	1	1

1	2	1	2	2	2	2	2	1	1
2	2	2	2	2	2	2	2	1	1
2	2	2	2	2	2	2	2	2	2
2	2	2	2	2	2	2	2	2	2
1	2	2	2	2	2	2	2	1	1
1	2	2	1	1	1	2	2	1	1
2	2	2	2	2	2	2	2	1	1
1	2	2	1	2	1	1	2	1	1
1	2	2	1	1	2	1	2	1	1
1	2	2	1	2	2	2	2	1	2
1	1	2	1	2	1	1	2	1	1
1	1	2	1	2	1	1	2	1	1
1	1	2	1	1	2	2	2	1	1
1	2	2	1	1	1	1	2	1	1
1	1	2	1	1	1	1	2	1	1
1	1	2	1	1	1	2	2	1	1
1	1	2	1	2	1	2	2	1	1
1	2	2	1	2	1	2	2	1	1
1	1	2	1	2	2	2	2	1	1
2	2	2	1	2	2	2	2	1	1
1	1	2	1	1	1	1	2	1	1
1	2	2	1	2	1	1	2	1	1
1	2	2	1	2	1	1	2	1	1
1	2	2	1	1	1	2	2	1	1
1	2	2	1	1	1	2	2	1	1
1	2	2	1	2	2	2	2	1	1
1	2	2	1	1	2	2	2	1	2
1	1	2	1	1	1	1	2	1	1
2	2	2	1	2	2	2	2	1	1
1	2	2	1	1	1	1	2	1	1
1	1	2	1	2	1	2	2	1	1
1	2	2	1	2	2	1	2	1	1
1	2	2	1	1	1	2	2	1	1
1	1	2	1	1	2	2	2	1	1
2	2	2	2	2	2	2	2	1	1
1	1	2	1	1	2	1	2	1	1
1	2	2	1	2	2	1	2	1	1
1	2	1	1	1	1	1	2	1	1
1	2	2	1	2	1	1	2	1	1
1	2	2	1	2	1	1	2	1	1
1	1	1	2	2	2	1	2	1	1
1	1	1	1	1	1	1	2	1	1
1	2	2	2	2	2	2	2	1	1
1	1	2	1	2	2	2	2	1	2
1	1	2	1	1	1	1	2	1	2
2	2	2	1	2	2	2	2	1	1
1	1	2	1	2	2	1	2	1	1

2	2	2	2	2	1	2	2	1	1
1	1	2	1	1	1	1	2	1	1
1	2	1	2	2	1	1	2	1	1
1	1	2	1	1	2	2	2	1	1
1	1	2	1	1	2	2	2	1	1
1	1	1	1	1	1	1	2	1	1
1	1	1	1	1	1	1	2	1	1
1	1	2	1	2	1	1	2	1	1
2	2	2	2	2	2	2	2	1	1
1	2	2	2	2	1	2	2	1	1
1	1	2	1	2	1	1	2	1	1
1	2	2	1	2	2	2	2	1	1
2	1	2	1	2	2	2	2	1	1
2	2	2	2	2	2	2	2	1	1
1	2	2	2	2	2	1	2	1	1
2	2	2	2	2	2	2	2	1	1
1	2	2	1	2	2	1	2	1	2
1	2	1	1	2	2	1	2	1	1
1	1	1	1	2	2	2	1	1	1
1	1	2	1	2	2	2	2	1	1
1	2	2	2	2	1	1	2	1	1
1	1	2	1	2	1	1	2	1	1
1	1	2	1	2	1	1	2	1	1
1	2	2	2	2	2	1	2	1	1
2	2	2	1	2	2	1	2	1	1
1	2	2	2	2	2	2	2	1	2
2	2	2	2	1	2	2	2	1	1
1	1	1	1	2	1	1	2	1	1
1	1	2	1	1	2	1	2	1	1
1	2	2	1	2	2	2	2	1	1
2	2	2	1	2	2	2	2	1	1
2	2	2	1	2	1	2	2	1	1
1	1	2	1	2	1	2	2	1	2
1	1	2	1	2	2	1	2	1	1
1	1	2	1	2	2	1	2	1	1
1	1	2	1	1	1	1	2	1	1
1	2	2	1	2	2	1	2	1	1
2	1	2	1	1	1	1	2	1	1
1	2	2	1	2	2	1	2	1	1
2	1	2	1	2	2	1	2	1	1
1	2	2	1	2	2	2	2	1	1
1	2	2	1	1	2	1	2	1	1
1	1	2	2	2	2	2	2	1	1
1	1	2	2	2	2	2	2	1	1
2	2	2	1	2	2	2	2	1	1
1	2	2	1	2	1	1	2	1	1
1	2	2	1	2	2	1	2	1	1

1	2	2	1	2	2	1	2	1	1
1	1	2	1	2	2	2	2	1	1
1	1	2	1	1	1	1	2	2	1
1	2	2	1	2	2	2	2	1	1
1	2	2	1	2	2	1	2	1	1
1	1	2	1	2	2	1	2	1	1
2	1	2	1	2	2	2	2	1	1
1	1	1	1	1	2	1	2	1	1
1	2	2	1	2	2	2	2	1	1
1	2	2	1	1	1	1	2	1	1
1	2	2	1	1	1	2	2	1	1
2	2	2	2	2	2	2	2	1	2
1	2	2	1	1	2	2	2	1	1
1	2	2	1	1	2	2	2	1	1
1	2	2	2	2	2	1	2	1	1
2	2	2	2	2	1	2	2	1	1
2	2	2	2	2	2	2	2	1	1
2	2	2	2	2	2	2	2	1	1
1	2	2	2	2	2	2	2	1	1
1	2	2	1	1	2	2	2	1	1
2	2	2	2	2	2	2	2	1	1
1	2	2	1	1	1	2	2	1	1
1	1	2	1	2	2	2	2	1	1
2	2	2	2	2	2	2	2	1	1
2	2	2	2	2	2	2	2	1	1
1	1	2	1	2	1	2	2	1	2
1	2	2	2	2	2	1	2	1	1
2	1	2	1	2	1	1	2	1	1
2	2	2	1	2	2	2	2	1	1
1	2	2	1	1	2	1	2	1	1
1	1	2	1	1	1	1	2	1	1
1	2	2	2	2	2	2	2	1	1
1	1	2	1	1	1	1	2	1	1
1	1	2	1	2	2	1	2	1	2
1	1	2	1	1	2	1	2	1	1
2	2	2	1	2	1	2	2	1	1
1	2	2	1	2	2	2	2	1	1
1	2	2	1	2	1	1	2	1	1
2	2	2	1	2	2	2	2	1	1
2	2	2	2	2	1	2	2	1	1
1	2	2	1	2	2	2	2	1	1
1	1	2	1	1	1	1	2	1	1
1	1	2	1	2	1	2	2	1	1
1	1	2	1	2	1	2	2	1	1
1	1	2	2	1	2	1	2	1	1
1	2	2	1	2	1	2	2	1	1
1	2	2	2	2	2	2	2	1	1

1	2	2	1	1	2	1	2	1	1
2	2	2	2	2	2	2	2	1	1
1	2	2	1	1	2	2	2	1	1
1	2	2	1	1	1	1	2	1	1
1	2	2	1	1	2	2	2	1	1
1	1	2	1	1	1	1	2	2	1
1	1	2	1	1	1	1	2	1	1
1	2	2	1	1	2	1	2	1	1
1	2	2	2	2	2	2	2	1	1
1	2	2	2	2	2	2	2	1	1
1	2	2	2	1	2	2	2	1	1
1	1	2	2	1	2	2	2	1	1
1	1	2	1	1	1	1	2	2	1
1	2	2	1	1	2	1	2	1	1
1	2	2	1	1	2	1	2	1	1
1	2	2	1	2	2	1	2	1	1
1	2	2	1	2	2	2	2	1	1
2	2	2	1	1	2	1	2	2	1
1	1	2	1	1	1	1	2	1	1
1	2	2	1	2	1	1	2	1	1
1	2	2	2	2	1	2	2	1	1
1	1	2	2	2	2	1	2	1	1
1	2	2	1	1	2	1	2	1	1
2	2	2	2	2	2	2	2	1	1
1	1	2	2	1	1	1	2	1	1
1	2	2	2	2	2	2	2	1	1
1	2	2	1	1	2	1	2	1	1
1	2	2	2	2	2	1	2	1	1
1	2	2	2	2	2	1	2	1	1
2	2	2	2	2	2	2	2	1	1
1	2	2	1	1	1	1	2	1	1
2	2	2	2	2	1	2	2	1	1
1	2	2	1	1	1	1	2	1	1
2	2	2	1	2	1	2	2	1	1
1	2	2	1	1	2	2	2	1	1
1	2	2	2	1	1	2	2	1	1
1	2	1	2	2	2	2	2	1	2
1	2	2	1	2	2	1	2	1	1
2	1	2	2	2	1	1	2	1	1
1	1	2	1	2	1	1	2	1	1
2	2	2	2	2	1	1	2	1	1
1	2	2	2	2	1	1	2	1	1
1	1	1	1	1	2	2	2	1	1
1	1	2	2	2	1	1	2	1	1
2	2	2	1	2	2	1	2	1	1
2	2	2	2	2	1	1	2	1	1
1	2	2	1	1	1	1	2	1	1

1	2	2	1	2	2	2	2	1	1
1	2	2	1	1	1	1	2	1	1
1	2	2	1	1	2	2	2	1	1
1	2	2	2	2	2	2	2	1	1
2	2	2	2	2	1	1	2	1	1
1	2	2	2	1	1	1	2	1	1
1	2	2	1	2	2	2	2	1	1
1	2	2	1	2	2	1	2	1	1
2	2	2	2	1	1	2	2	1	1
1	2	2	2	2	2	2	2	1	1
1	2	2	2	2	2	1	2	1	1
1	1	2	1	2	1	1	2	1	1
1	2	2	1	1	1	2	1	1	1
2	2	2	1	2	2	2	2	1	1
2	2	2	1	2	2	2	2	1	1
2	2	2	2	2	2	2	2	1	1
2	2	2	2	2	2	2	2	1	1
1	2	2	1	2	2	2	2	1	1
2	2	2	2	2	1	1	2	1	1
1	2	2	1	1	1	1	2	1	1
2	2	2	2	2	2	2	2	1	1
1	2	2	1	2	2	1	2	1	1
1	2	2	1	2	2	1	2	1	1
1	2	2	2	2	2	2	2	1	1
2	2	2	2	2	2	2	2	1	1
2	2	2	1	2	2	2	2	1	1
1	1	2	1	2	2	2	2	1	1
1	2	2	1	2	2	2	2	1	1
1	2	2	1	1	1	1	2	1	1
1	2	2	1	2	2	1	2	1	1
1	2	2	1	2	2	2	2	2	1
1	1	2	1	1	1	1	2	2	1
1	1	2	2	2	1	1	2	1	1
1	1	2	1	1	1	1	2	1	1
1	1	2	1	1	1	1	2	1	1
2	2	2	2	2	2	2	2	1	1
2	1	2	1	2	2	2	2	1	1
2	2	2	1	2	2	1	2	1	1
1	2	2	2	2	2	1	2	1	1
1	2	2	2	2	2	2	2	1	1
1	2	2	1	2	2	2	2	1	1
1	2	2	1	1	2	2	2	1	1
1	2	2	1	2	2	2	2	1	1
1	2	2	2	2	1	2	2	1	1
1	2	2	2	2	2	2	2	1	1
1	2	2	2	2	1	2	2	1	1
1	2	2	1	1	1	1	2	1	1

1	2	2	1	2	1	1	2	1	1
1	1	2	2	2	1	1	2	2	1
1	2	2	2	1	1	2	2	1	1
1	2	2	2	2	1	2	2	1	1
1	2	2	2	2	1	2	2	1	1
1	2	2	2	2	1	2	2	1	1
1	2	2	1	1	2	1	2	1	1
1	2	2	1	1	1	1	2	1	1
1	2	2	2	2	2	2	2	1	1
1	1	2	1	2	2	2	2	1	1
1	1	2	1	2	1	2	2	1	1
1	2	2	2	1	1	1	2	1	1
1	2	2	1	1	1	1	2	1	1
1	2	2	1	2	2	2	2	2	1
1	2	2	1	1	1	1	2	1	1
1	2	2	2	1	1	2	2	1	1
1	1	1	1	1	1	1	2	1	1
1	2	2	1	1	1	1	2	1	1
1	2	2	1	2	2	2	2	1	1
1	1	1	1	1	1	1	2	1	1
1	1	2	1	2	2	1	2	1	1
1	2	2	1	2	1	1	2	1	1
1	1	2	1	2	1	2	2	1	1
1	2	2	2	2	2	2	2	1	1
1	2	2	1	1	1	1	2	1	1
1	2	2	1	2	2	2	2	1	1
1	2	2	1	2	2	2	2	1	1
2	2	2	2	2	2	2	2	1	1
1	1	2	1	1	1	1	2	1	1
1	1	2	1	1	1	1	2	1	1
1	2	2	1	1	2	2	2	1	1
1	2	2	1	1	1	2	2	1	1
2	2	2	2	2	2	2	2	2	1
1	2	2	1	1	1	1	2	1	1
1	2	2	1	1	1	1	2	1	1
1	2	2	1	1	1	1	2	1	1
1	2	2	1	2	2	2	2	1	1
1	2	2	1	2	2	2	2	1	1
1	2	2	1	1	1	2	2	1	1
1	1	2	1	1	1	1	2	1	1
2	2	2	2	2	2	2	2	1	1
1	1	2	1	2	2	2	2	1	1
2	2	2	2	2	2	2	2	1	2
1	1	2	1	1	1	2	2	1	1
1	1	2	1	2	2	2	2	1	1
2	2	2	2	2	2	2	2	1	1
1	1	2	1	1	1	1	2	1	1

1	2	2	1	2	1	1	2	1	1
1	2	2	1	2	1	1	2	1	1
1	1	2	1	2	2	2	2	1	1
1	1	2	1	2	2	2	2	1	1
1	1	2	1	2	2	2	2	1	1
1	2	2	1	1	2	2	2	1	1
1	1	2	1	2	1	2	2	2	1
2	2	2	1	2	2	2	2	1	2
1	2	2	1	1	1	1	2	1	1
1	1	2	1	1	1	1	2	2	2
1	2	2	1	2	2	1	2	1	1
1	2	2	1	2	1	1	2	1	1
1	1	2	1	1	2	2	2	1	1
2	2	2	2	2	2	2	2	1	2
1	2	2	1	1	2	1	2	1	1
1	2	2	1	1	1	1	2	1	1
1	2	2	1	1	1	1	2	2	1
1	2	2	2	1	1	1	2	1	1
1	1	2	1	1	1	1	2	1	1
2	2	2	2	2	2	1	2	1	1
1	1	2	2	2	1	1	2	1	1
1	2	2	2	2	2	1	2	1	1
1	1	2	1	2	1	1	2	1	1
2	2	2	1	2	2	2	2	1	1
1	2	2	1	2	2	1	2	1	1
2	2	2	2	2	2	2	2	1	1
1	2	2	1	1	2	1	2	1	1
1	1	2	1	1	1	1	2	1	1
1	2	2	1	2	2	2	1	1	1
1	2	2	1	1	2	1	2	1	1
1	1	2	1	2	2	2	2	1	1
2	2	2	2	2	2	2	2	1	1
1	2	2	1	1	2	1	2	1	1
1	1	2	1	2	2	1	2	1	1
2	1	2	2	2	2	2	2	1	1
1	2	2	2	2	2	1	2	1	1
1	2	2	1	2	2	1	2	1	1
1	2	2	2	2	2	1	2	1	1
1	2	2	1	2	2	2	2	2	1
2	2	2	2	2	2	2	2	1	1
1	1	2	1	1	1	1	2	1	1
1	1	2	1	1	1	1	2	1	1
1	2	2	1	2	2	1	2	1	2
1	1	2	1	1	1	1	2	1	1
1	2	2	1	2	2	2	2	1	1
2	2	2	1	1	2	2	2	1	1
1	1	2	1	1	1	1	2	1	1

1	1	2	1	2	2	2	2	1	1
1	1	2	1	2	1	2	2	1	1
1	2	2	1	2	2	1	2	1	1
2	2	2	2	2	2	2	2	1	1
1	2	2	2	2	1	2	2	1	1
1	1	2	1	2	2	1	2	1	1
1	2	2	1	2	1	2	2	1	1
1	2	2	2	2	1	1	2	1	1
1	1	2	1	1	2	1	2	1	1
1	2	2	1	1	2	1	2	1	1
1	2	2	1	2	2	2	2	1	1
1	2	2	1	2	2	1	2	1	1
1	1	2	1	2	2	2	2	1	1
1	1	2	1	2	2	2	2	1	1
2	2	2	1	2	2	2	2	1	1
1	1	2	1	1	2	2	2	1	1
1	2	2	1	1	1	1	2	1	1
1	2	2	1	1	1	1	2	1	1
2	2	2	2	2	2	2	2	1	1
1	2	2	1	2	2	1	2	1	1
1	1	2	1	2	1	1	2	1	1
1	1	2	1	1	2	2	2	1	1
1	1	2	1	2	2	1	2	1	1
1	2	2	1	1	2	2	2	1	1
1	2	2	2	2	2	2	2	1	1
1	2	2	1	2	1	2	2	1	1
1	2	2	1	2	1	2	2	1	1
1	2	2	1	2	2	1	2	1	1
2	2	2	2	2	1	1	2	1	1
1	2	2	1	1	2	1	2	1	1
2	2	2	2	2	2	2	2	1	1
1	2	2	1	1	2	2	2	1	1
1	1	2	1	2	2	2	2	1	1
2	2	2	2	2	1	2	2	1	1
2	2	2	2	2	1	2	2	1	1
1	1	2	1	2	2	2	2	1	1
1	2	2	1	2	2	1	2	1	1
2	2	2	2	1	1	2	2	1	1
1	2	2	2	1	1	1	2	1	1
1	2	2	1	2	2	2	2	1	1
1	2	2	2	1	1	1	2	1	1
1	2	2	2	2	2	1	2	1	1
1	1	2	1	1	2	1	2	1	1
1	2	2	1	2	1	2	2	1	1
2	2	2	2	2	2	2	2	1	1
1	1	2	1	1	1	2	2	1	1

shoprite	pick n pay	Spar	Boxer	Usave	Checkers	Choppies	Game	street ven	Savemor
1	1	1	1	1	1	1	1	2	2
1	1	1	1	1	1	1	1	2	2
1	2	2	1	2	2	2	2	1	2
1	1	1	1	1	2	2	1	2	2
2	2	2	2	2	2	2	2	2	2
1	1	1	1	2	1	2	1	1	2
1	2	2	1	2	2	2	2	2	2
1	1	1	1	1	1	2	2	1	2
1	2	1	1	1	2	2	2	1	2
1	2	1	1	1	2	2	2	2	2
1	2	1	1	1	2	1	2	2	2
1	1	1	1	1	1	2	2	1	2
1	2	1	1	1	2	1	2	1	2
1	2	2	1	1	2	2	2	1	1
1	2	1	1	1	2	2	2	2	2
1	1	1	1	1	1	1	1	2	2
1	1	1	1	1	1	1	1	2	2
1	2	1	1	2	2	1	2	1	2
1	2	1	1	1	2	2	2	2	2
1	2	1	1	2	2	1	1	1	2
1	2	1	1	2	2	2	2	1	2
1	1	1	1	1	2	2	2	1	2
1	2	1	1	1	2	2	2	1	2
1	2	1	1	1	2	2	2	1	2
1	2	1	1	1	2	1	2	1	2
1	2	1	1	1	2	1	2	1	2
1	1	1	1	1	2	2	2	2	2
1	2	2	2	2	2	2	2	1	2
1	1	1	1	1	1	2	1	1	2
1	2	2	2	2	2	2	2	2	2
2	2	2	1	2	2	2	2	2	2
1	2	1	1	1	1	2	2	1	1
1	2	2	1	1	2	2	2	1	2
1	1	1	1	1	1	1	1	2	1
1	2	1	1	1	2	2	2	1	2
1	2	1	1	1	2	2	2	2	2
1	2	1	1	1	2	2	2	2	2
2	2	2	2	1	2	2	2	2	2
1	2	1	1	1	2	2	2	1	2
1	1	1	1	1	1	2	1	2	2
1	2	1	1	1	1	1	1	1	2
1	2	1	1	1	2	2	2	1	2
1	1	1	1	1	2	2	2	2	2
1	2	1	1	1	2	1	2	2	2
1	2	1	1	1	2	2	2	2	2
2	2	1	1	2	2	2	2	2	2

1	1	1	1	1	2	2	1	2	2
1	2	2	1	1	2	2	2	1	2
1	2	2	1	1	2	1	2	1	2
1	1	1	1	1	1	1	1	1	1
1	2	1	1	1	1	1	1	2	2
1	2	1	1	1	1	1	2	2	2
1	1	1	1	1	2	2	2	1	1
1	2	1	1	1	2	2	2	1	2
1	2	1	1	1	2	2	2	1	2
1	2	2	1	1	2	2	2	1	2
1	2	1	1	1	1	2	2	1	2
1	2	1	1	1	2	2	2	1	2
1	2	1	1	1	2	2	2	1	2
1	1	1	2	1	2	2	1	1	2
1	1	1	1	1	2	1	1	1	2
1	1	1	1	1	1	1	1	1	2
1	1	1	1	1	1	1	1	2	1
1	2	1	1	2	2	2	2	2	2
1	2	1	1	1	2	2	1	1	2
1	1	1	1	1	2	1	1	2	2
1	2	1	1	1	2	2	2	1	2
1	2	1	1	1	1	1	1	1	1
1	2	1	1	1	1	1	1	1	1
1	2	2	2	1	1	2	2	1	2
1	2	2	2	1	1	2	2	1	2
1	2	1	1	1	2	2	1	2	2
1	2	1	1	1	2	2	2	2	2
1	1	1	1	1	1	1	1	1	2
1	2	1	1	1	2	2	2	2	2
1	1	1	1	1	1	1	1	1	1
1	1	1	1	1	2	2	2	2	2
1	1	1	1	1	2	2	2	2	2
1	1	1	1	1	2	1	2	1	2
1	2	1	1	2	2	2	1	2	2
1	1	1	1	1	2	1	1	1	2
1	2	1	1	1	2	1	2	1	2
1	1	1	1	1	2	1	2	1	2
1	1	1	1	2	1	2	2	1	2
1	2	2	1	2	2	2	2	2	2
1	2	1	1	1	2	1	1	2	2
1	2	1	1	1	2	1	1	1	2
1	2	1	1	1	2	1	1	1	2
1	2	1	1	1	2	2	2	1	2
1	2	1	1	1	1	2	1	1	2
1	1	1	1	1	2	1	1	1	1
1	2	1	1	1	2	2	1	1	2
1	1	1	1	1	2	2	1	1	2

1	1	1	1	1	2	2	2	2	2
1	1	2	2	2	2	2	2	2	2
1	2	1	1	1	1	2	2	2	2
1	1	1	1	1	2	2	2	2	2
1	2	2	2	1	2	2	2	1	2
1	2	1	1	1	2	2	2	1	2
1	2	2	1	1	2	2	2	1	2
1	1	1	1	1	2	2	1	2	2
1	2	1	1	1	2	2	2	2	2
1	1	1	1	1	1	2	2	1	2
1	2	1	1	1	2	1	2	2	2
1	1	1	1	1	1	2	2	1	2
1	2	1	2	1	2	2	1	2	2
1	2	1	1	2	1	2	1	1	2
1	2	1	1	1	2	1	2	2	2
1	2	1	1	2	2	2	2	1	2
1	2	2	1	2	2	2	2	1	2
1	2	1	1	2	2	2	2	2	2
1	1	1	1	1	1	1	2	1	2
1	2	1	1	2	2	2	1	1	2
1	2	1	1	2	2	1	2	1	2
1	1	1	1	2	1	2	2	1	2
1	1	1	1	1	1	2	1	1	2
1	1	1	1	1	1	2	1	1	2
1	1	1	1	1	1	2	1	1	2
1	2	1	1	1	2	2	2	1	1
1	2	1	1	1	2	1	2	1	2
2	2	1	1	2	2	2	2	2	2
1	2	1	1	1	2	1	1	1	2
1	2	1	1	1	2	2	2	1	2
1	1	1	1	1	1	1	2	2	2
1	1	1	1	1	1	2	1	1	2
1	2	1	1	2	2	1	2	1	2
1	1	1	1	2	2	2	2	2	2
1	1	1	1	2	1	2	2	2	2
1	2	1	1	2	2	2	2	2	2
1	1	1	2	1	2	2	1	2	2
1	1	1	1	1	2	2	2	1	2
1	2	1	1	2	2	2	2	2	2
1	2	1	1	2	2	2	2	2	2
1	1	1	1	1	1	1	1	1	1
1	2	2	1	2	2	2	2	2	2
1	2	1	1	2	2	2	2	2	2
1	2	1	1	2	2	2	1	2	2
1	2	1	1	2	2	2	2	1	2
1	1	1	1	1	1	2	1	1	1
1	2	1	1	2	2	2	2	1	2

1	1	1	1	1	1	2	2	1	2
1	1	1	1	1	1	2	2	1	2
1	2	1	1	1	1	2	2	2	1
1	2	1	1	1	1	2	2	2	1
1	1	1	1	1	1	2	2	1	2
1	1	1	1	1	1	1	1	1	1
1	2	1	1	2	2	2	2	1	2
1	1	1	1	1	1	1	1	1	1
1	1	1	1	2	2	2	2	1	2
1	2	2	1	2	1	2	2	2	2
1	2	1	1	1	2	1	2	1	1
1	2	1	1	1	2	1	2	1	1
1	2	1	1	2	2	1	1	2	2
1	1	1	1	1	1	2	1	1	2
1	1	1	1	1	1	1	1	1	2
1	2	1	1	1	2	2	1	1	2
1	1	1	1	1	1	2	1	1	2
1	2	1	1	1	1	2	2	1	2
1	2	1	1	2	2	2	2	2	1
1	2	1	1	2	2	2	2	2	2
1	1	1	1	1	1	1	1	1	1
1	2	1	1	2	2	2	2	1	2
1	2	1	1	2	2	2	2	1	2
1	2	1	1	1	1	2	1	1	2
1	2	1	1	1	1	2	1	1	2
1	1	1	1	1	1	2	2	1	2
1	2	1	2	2	2	2	2	2	2
1	2	1	1	1	1	2	2	2	2
1	2	1	2	2	2	2	2	1	2
1	2	1	1	1	1	2	2	1	2
1	2	1	1	1	2	2	1	1	2
1	2	1	1	1	1	2	2	1	2
1	2	1	1	2	2	2	1	2	2
1	2	1	1	1	2	1	2	1	2
1	2	1	1	2	1	2	2	1	2
1	1	1	1	1	1	2	2	2	2
1	1	1	1	1	2	2	1	1	2
1	2	1	1	2	1	2	1	1	2
1	2	1	1	2	1	2	2	1	2
1	2	1	1	2	1	2	2	1	2
1	2	1	1	1	1	2	1	1	2
1	1	1	1	1	2	2	1	1	1
1	2	1	1	1	2	2	2	2	2
1	2	2	1	2	2	2	2	2	2
1	2	1	1	2	2	2	2	2	2
1	2	1	1	1	1	1	1	2	2
1	2	1	1	2	1	2	2	1	2

1	2	2	1	2	2	2	2	2	2
1	1	1	1	1	1	1	1	1	2
1	1	1	1	1	1	2	2	1	2
1	2	2	1	2	2	2	2	1	2
1	2	2	1	2	2	2	2	1	2
1	1	1	1	1	1	1	1	1	2
1	1	1	1	1	1	1	1	1	2
1	2	2	1	2	2	2	2	1	2
1	2	1	1	2	2	2	2	2	2
1	1	1	1	1	2	2	2	1	2
1	2	2	1	2	2	2	1	1	2
1	2	1	1	1	2	2	2	1	1
1	1	1	1	1	1	2	1	2	2
1	1	1	1	1	1	1	2	1	2
1	2	2	1	2	1	2	2	2	2
1	2	1	1	1	2	2	2	2	1
2	2	1	1	2	2	2	2	2	2
1	2	1	1	1	2	1	1	2	2
1	2	1	1	2	1	2	1	1	2
1	2	2	2	2	2	2	2	2	2
1	2	1	1	2	1	2	1	2	2
1	1	1	1	1	1	1	1	1	2
1	1	1	1	1	1	1	1	1	2
1	2	1	1	1	2	1	2	1	1
1	2	2	1	2	1	2	2	1	2
1	2	1	1	2	2	2	2	2	2
1	2	1	1	1	2	2	2	1	2
1	2	1	1	2	1	1	2	2	2
1	2	2	1	1	2	2	2	1	2
1	2	1	1	1	2	2	2	1	2
1	2	1	1	1	2	2	2	1	2
1	1	1	1	2	1	2	2	2	2
2	1	2	1	2	2	2	2	2	2
1	2	1	1	2	2	2	2	1	2
1	2	1	1	1	1	2	2	1	2
1	1	1	1	1	1	1	1	1	1
1	2	1	1	2	2	2	2	1	2
1	1	1	1	1	1	1	2	1	2
1	2	1	1	1	2	2	2	1	2
1	2	1	1	1	2	1	2	1	2
1	2	1	1	1	2	1	2	1	2
1	2	1	1	2	2	2	2	2	2
1	2	1	1	2	2	2	2	1	2
1	2	1	1	2	2	2	2	1	2
1	2	2	1	2	2	2	2	1	2
1	1	2	1	1	1	2	1	2	2
1	2	1	1	2	2	2	2	1	2

1	2	1	1	2	2	2	2	1	2
1	1	1	1	1	1	1	1	2	2
1	2	1	1	1	2	2	1	1	2
1	1	1	1	1	1	2	2	2	2
1	1	1	1	1	1	1	1	1	1
1	1	1	1	1	1	1	1	1	1
2	2	1	2	2	2	2	2	1	2
1	1	1	1	1	1	1	2	1	2
1	1	1	1	1	2	2	2	1	2
1	1	1	1	1	1	1	2	2	2
1	2	1	1	1	1	2	1	1	2
1	2	2	2	1	2	2	2	2	2
1	2	1	1	1	2	1	1	1	2
1	2	1	1	1	2	1	1	1	2
1	2	1	1	1	2	2	2	2	2
2	2	1	1	2	2	2	2	2	2
1	2	1	1	1	2	2	2	1	2
1	2	1	1	1	2	2	2	1	2
1	2	1	1	1	2	2	2	1	2
1	2	2	1	1	2	2	2	1	2
1	2	1	1	1	2	2	2	1	2
1	2	1	1	1	2	2	1	1	2
1	1	1	1	1	1	1	1	1	1
1	2	1	1	1	2	2	2	1	2
1	2	1	1	1	2	2	2	1	2
1	2	1	1	1	2	2	2	2	2
1	2	1	1	1	2	2	2	2	2
1	2	2	1	1	2	2	2	1	2
1	2	1	1	1	2	2	1	1	2
1	1	1	1	1	2	2	1	2	2
1	2	1	1	1	1	1	1	1	2
1	2	1	1	1	2	2	2	1	2
1	2	1	1	1	2	2	1	1	2
1	1	1	1	1	2	2	1	2	1
1	2	1	1	1	2	2	1	1	2
1	2	2	1	2	2	2	2	2	2
1	2	1	1	1	2	2	2	1	2
1	1	1	1	1	1	1	1	1	2
1	2	2	1	1	2	1	1	1	2
1	2	1	1	1	2	2	1	1	2
1	2	1	1	1	2	2	2	1	2
1	2	1	1	1	2	2	1	1	2
1	1	1	1	1	1	1	1	1	2
1	1	1	1	1	1	1	1	1	2
1	2	1	2	2	2	2	1	1	2
1	1	1	1	1	1	1	1	1	2
1	1	1	1	1	2	2	2	2	2

1	1	1	1	1	1	1	1	1	2
1	1	1	1	1	2	2	2	2	2
1	2	1	1	1	2	2	2	2	2
1	1	1	1	2	1	2	1	1	2
1	1	1	1	1	1	1	1	1	2
1	2	1	1	2	2	2	1	2	2
1	2	2	2	1	2	2	2	2	2
1	1	1	1	1	1	1	1	2	2
1	1	1	1	1	1	1	1	2	2
1	1	1	1	1	1	1	1	2	2
1	1	1	1	1	1	1	1	2	2
2	2	1	1	2	2	2	2	2	2
1	2	1	1	1	2	2	2	1	2
1	1	1	1	1	1	1	1	2	2
1	1	1	1	1	2	2	1	1	2
1	2	2	2	2	2	2	2	1	2
1	2	1	1	2	2	2	2	1	2
1	2	1	1	1	2	2	2	1	2
1	1	1	1	1	1	1	1	2	2
1	2	2	2	2	2	2	2	2	2
1	2	1	1	2	2	2	2	1	1
1	2	1	1	1	2	2	2	1	2
1	2	1	1	1	1	1	1	1	2
1	2	1	1	2	1	2	2	1	2
1	1	1	1	1	2	2	1	1	2
1	2	2	2	1	2	2	2	2	2
1	1	1	1	1	2	2	2	1	1
1	2	1	2	2	2	2	2	2	2
1	1	1	1	1	2	2	1	1	2
1	2	2	2	1	2	2	2	2	2
1	1	1	2	2	2	2	1	2	2
1	2	1	1	2	2	2	2	1	2
1	2	1	1	2	2	2	2	1	2
1	2	1	1	1	2	2	2	2	2
1	2	1	1	1	2	1	1	1	2
1	2	2	1	1	2	2	2	1	2
1	2	2	2	2	2	2	2	1	2
1	2	1	1	1	2	2	2	1	2
1	1	1	1	1	1	1	1	1	2
1	1	1	1	1	1	1	1	1	2
1	2	1	1	2	2	2	2	2	2
1	1	1	1	2	2	2	2	1	2
1	2	1	1	1	2	2	1	1	2
1	2	1	1	2	2	2	2	1	2
1	2	1	1	2	2	2	2	2	2
1	1	2	1	1	1	1	1	1	2
1	2	1	1	1	2	2	1	1	2

1	2	1	1	1	2	2	2	2	2
1	2	1	1	1	2	2	2	2	2
1	1	1	1	2	2	2	2	2	2
1	1	1	1	1	2	2	2	2	2
1	1	1	1	1	1	1	1	1	1
1	1	2	1	1	2	2	1	1	2
1	2	1	1	1	2	2	1	2	2
1	2	1	1	1	2	2	1	1	2
1	2	2	1	1	2	2	2	2	2
1	1	1	2	2	2	2	2	2	2
1	2	1	1	1	2	2	1	1	2
1	2	1	1	2	2	2	1	2	2
1	2	1	1	2	2	2	1	1	2
1	2	1	1	2	2	2	2	1	2
1	2	1	1	1	2	2	2	1	2
1	2	1	1	2	2	2	2	1	2
1	2	1	1	2	2	2	2	1	2
1	2	1	1	2	2	2	1	1	2
1	1	1	1	1	1	1	1	1	1
1	2	1	1	1	2	2	1	2	2
1	1	1	1	1	1	1	1	2	2
2	2	1	1	1	2	2	2	1	2
1	2	1	1	1	2	2	1	1	2
1	2	1	1	2	2	2	2	2	2
1	2	1	1	1	2	2	1	1	2
1	2	1	1	1	2	2	1	2	2
1	1	1	1	1	2	2	1	1	2
1	1	1	1	1	2	2	1	1	2
1	2	1	1	1	2	2	1	1	2
1	2	1	1	2	2	2	1	1	2
2	2	2	1	2	2	2	2	2	2
1	1	1	1	1	2	2	2	1	2
1	2	2	1	1	2	2	2	2	2
1	1	1	1	1	2	1	1	2	2
1	1	1	1	1	2	2	1	1	2
1	2	1	1	1	2	2	2	2	2
1	2	1	1	1	1	1	2	1	2
1	2	1	1	1	2	2	2	2	2
1	2	1	1	1	2	2	2	2	2
1	2	2	1	1	2	2	2	2	2
1	1	1	1	2	2	2	2	2	2
1	1	1	2	1	1	2	1	1	2
1	1	1	1	2	2	2	1	2	2
1	2	1	1	2	2	2	2	1	2
1	1	1	1	2	2	2	2	2	2
1	2	1	1	2	2	2	2	1	2
1	1	1	2	2	2	2	2	1	2

1	2	1	1	1	2	1	1	1	2
1	2	1	1	2	2	2	2	2	2
1	2	1	1	2	2	2	2	1	2
1	2	1	1	1	2	2	2	1	2
1	2	1	1	1	2	2	2	1	1
1	2	1	1	1	2	2	2	1	1
1	2	1	1	2	1	2	2	1	2
1	2	1	1	1	2	2	1	1	2
2	2	2	1	2	2	2	2	2	2
1	1	1	1	2	2	2	1	1	2
1	2	1	1	1	1	1	1	1	1
1	2	1	1	2	2	2	1	1	2
1	2	1	1	2	2	2	1	1	2
1	2	2	1	2	2	2	2	1	2
1	2	1	1	2	2	2	1	1	2
1	2	1	1	2	2	2	2	1	2
1	2	2	1	1	1	2	1	1	1
1	2	1	1	2	2	2	2	1	2
1	2	1	2	2	2	2	2	2	2
1	2	2	1	1	1	2	1	1	1
1	1	1	1	1	1	1	1	1	2
1	2	1	1	2	2	2	2	1	2
1	1	1	1	1	1	1	1	1	1
1	2	2	1	1	1	2	2	1	2
1	1	1	1	2	2	2	1	1	2
1	1	1	1	1	1	1	1	1	1
1	1	1	1	1	1	1	1	2	2
1	1	1	1	1	1	1	1	1	1
1	1	1	1	1	1	1	1	1	1
1	1	1	1	1	1	1	1	1	1
1	1	1	1	1	2	1	2	2	2
1	1	1	1	1	1	1	1	1	2
1	2	2	1	2	2	2	2	2	2
1	1	1	1	1	1	2	1	1	2
1	1	1	1	2	1	2	2	2	2
1	1	1	1	1	1	2	2	1	2
1	2	1	1	2	1	2	2	1	2
1	2	1	1	2	1	2	2	1	2
1	2	2	1	2	2	2	2	2	2
1	1	1	1	1	1	2	1	2	2
1	2	1	1	2	2	2	1	2	2
1	1	1	1	1	1	2	2	2	2
2	2	2	1	2	2	2	2	2	2
1	1	1	1	1	2	1	2	1	1
1	1	1	1	1	2	2	2	1	2
1	1	1	1	1	1	1	2	1	1
1	1	1	1	1	1	1	1	1	2

1	2	1	1	2	2	2	2	2	2
1	2	1	1	2	2	2	2	2	2
1	1	1	1	1	1	2	1	2	2
1	1	1	1	1	1	2	1	2	2
1	1	1	1	1	1	1	2	2	2
1	1	1	1	2	1	2	1	1	2
1	2	2	1	2	2	2	2	1	2
1	2	1	1	2	2	2	2	2	2
1	2	1	1	1	1	2	2	2	2
1	2	1	1	2	2	2	2	1	2
1	1	1	1	1	1	1	1	2	2
1	1	1	1	1	2	2	2	2	2
1	1	1	1	1	1	1	1	1	1
2	2	2	1	2	2	2	2	2	2
1	2	1	1	1	1	2	1	1	2
1	1	1	1	2	2	2	2	2	2
1	2	1	1	2	2	2	2	1	2
1	2	1	1	2	2	2	2	2	2
1	1	1	1	1	1	1	1	1	1
1	1	1	1	1	1	2	1	2	2
1	1	1	1	2	2	2	2	2	2
1	2	1	1	1	2	2	2	2	2
1	2	1	1	2	2	2	2	2	2
1	2	2	1	1	2	2	2	1	2
1	2	1	1	1	2	2	2	1	2
1	2	1	1	1	2	2	1	2	2
1	2	1	1	2	2	2	2	2	2
1	1	1	1	1	1	1	1	1	2
1	2	2	1	1	2	2	2	2	2
1	1	1	1	2	1	1	1	1	2
1	1	1	1	1	1	1	1	2	2
1	2	1	1	2	2	2	2	1	2
1	1	1	1	1	1	1	1	2	2
1	1	1	1	1	1	1	2	2	2
1	1	1	1	1	2	2	2	1	2
1	1	1	1	1	2	2	2	2	2
1	2	2	2	2	2	2	2	2	2
1	2	1	1	2	2	2	2	1	2
1	2	2	1	2	2	2	2	1	2
1	2	1	1	2	2	2	2	2	2
1	1	1	1	1	1	1	1	1	1
1	1	1	1	1	1	1	1	1	1
1	2	2	1	2	2	2	2	1	2
1	1	1	1	1	1	1	1	2	2
1	2	1	1	1	2	1	1	1	2
1	2	1	1	1	2	1	2	1	2
1	1	1	1	1	1	1	1	1	2

1	1	1	1	1	1	1	2	2	2
1	2	2	1	1	2	2	2	1	2
1	1	1	1	1	1	2	2	1	2
2	2	2	2	2	2	2	2	1	2
1	1	1	2	2	2	2	2	2	2
1	1	1	1	1	1	2	1	1	1
1	2	1	1	2	2	2	2	1	2
1	1	1	1	2	2	1	2	2	2
1	2	1	1	1	1	2	2	1	2
1	2	2	1	2	2	2	2	2	2
1	1	1	1	1	2	2	2	1	2
1	2	2	1	2	1	2	2	2	2
1	1	1	1	1	1	1	1	1	2
1	1	1	1	1	1	1	1	1	2
1	2	2	1	2	2	2	2	1	2
1	2	1	1	2	2	2	1	2	2
2	1	1	1	1	1	2	1	1	1
1	2	1	1	1	1	2	1	1	1
1	2	2	2	2	2	2	2	1	2
1	1	1	1	1	2	2	2	1	2
1	2	1	1	1	2	2	2	1	2
1	2	1	1	2	2	2	1	2	2
1	1	1	1	1	1	1	1	1	1
1	2	1	1	1	2	1	2	1	2
1	2	2	1	1	2	2	2	1	2
1	1	1	1	1	2	2	2	1	2
1	1	1	1	1	2	2	2	1	2
1	2	1	1	2	2	2	2	2	2
1	2	1	1	1	2	2	2	1	2
1	2	1	1	1	1	2	1	1	2
1	2	2	1	1	2	2	2	1	2
1	1	1	1	1	2	2	1	2	2
1	1	1	1	1	2	2	2	2	2
1	1	1	1	1	2	2	2	1	2
1	1	1	1	1	2	2	2	1	2
1	2	2	1	1	2	2	2	1	2
1	2	1	1	2	2	2	2	2	2
1	1	1	1	1	1	1	1	1	1
1	2	1	1	2	2	2	2	2	2
1	2	1	1	1	2	2	2	1	2
1	2	1	1	2	2	2	2	2	2
1	1	1	1	1	2	2	2	2	2
1	1	1	1	1	2	2	1	1	2
1	1	1	1	1	2	1	1	1	2
1	1	1	1	2	2	2	2	2	2
1	1	1	1	1	2	2	1	1	1

OK grocery	pick up wil	shops unk	Garden	Orchard/F	Field in the	Fields awa	Small hold	Large farn	Refrigerato
2	2	2	2	1	1	2	2	1	1
2	2	2	2	1	1	2	2	1	1
2	2	2	2	2	1	1	2	2	1
2	2	2	2	2	2	2	2	2	1
2	2	2	2	2	2	2	2	2	1
1	1	1	2	2	2	1	2	2	1
2	2	2	1	1	1	2	2	2	1
1	1	1	1	2	1	1	2	2	1
2	2	2	1	2	2	2	2	2	1
2	2	2	1	2	1	1	2	2	1
2	2	2	1	1	2	2	1	2	1
2	2	2	1	1	1	2	2	2	1
2	2	1	1	2	1	1	2	2	1
2	2	2	1	2	2	2	2	2	1
2	2	2	1	2	1	2	2	2	1
1	2	2	1	1	1	1	1	1	1
1	2	2	1	1	1	1	1	1	1
2	2	2	2	2	2	1	2	2	1
2	2	1	1	1	2	1	2	2	1
2	2	2	2	2	2	2	2	2	1
2	2	2	2	2	2	2	2	2	1
2	2	2	1	1	2	2	2	2	1
2	2	2	1	2	1	2	2	2	1
2	2	1	2	2	2	1	2	1	1
2	2	1	1	2	1	2	2	1	1
2	2	1	1	2	1	2	2	2	1
2	2	2	1	2	1	1	2	2	1
2	2	1	2	2	2	1	2	2	2
1	2	2	2	2	2	2	2	2	2
2	2	2	2	2	2	2	1	2	1
2	2	2	2	2	2	2	2	2	2
2	2	2	1	2	1	1	2	2	1
2	2	2	2	2	2	1	2	2	1
1	1	1	2	1	2	1	2	1	1
2	2	2	2	2	1	2	2	2	2
2	2	2	1	2	1	1	2	2	1
2	2	2	2	2	2	2	2	2	1
2	2	2	2	2	1	2	2	2	2
2	2	2	1	2	2	1	2	2	1
2	2	2	1	2	1	2	2	2	1
2	1	2	2	2	2	2	2	2	1
2	2	2	2	2	2	2	2	2	1
2	2	2	1	2	1	1	2	2	2
2	2	2	1	2	2	2	2	2	1
2	2	2	2	2	1	2	1	2	2
2	2	2	2	2	2	2	2	2	1

2	2	2	2	2	2	2	2	2	1
2	2	1	2	2	1	1	2	2	2
2	2	2	2	2	1	1	2	2	1
1	2	2	1	2	1	1	2	2	1
2	2	2	1	2	2	1	2	2	1
2	2	2	2	1	1	1	2	2	1
2	2	2	2	2	1	2	2	2	2
2	2	2	2	2	2	1	2	2	1
2	2	2	1	2	1	1	2	2	1
2	2	2	2	2	2	1	2	2	1
2	2	2	1	1	1	1	2	2	1
2	2	2	2	2	2	2	2	2	1
2	2	2	1	1	1	1	2	2	1
1	2	2	1	2	2	2	2	2	1
2	2	1	2	2	1	2	2	2	1
2	2	2	2	2	2	1	2	2	1
1	2	2	2	2	1	1	2	2	1
2	2	2	2	2	2	1	2	2	1
2	2	2	1	2	1	2	2	2	2
2	2	2	2	2	1	2	2	2	1
2	2	2	1	2	1	2	2	2	1
2	2	2	1	2	1	1	2	2	1
2	2	2	1	2	1	1	2	2	1
2	2	2	2	2	1	2	1	2	2
2	2	2	2	2	1	2	1	2	2
2	2	2	1	2	1	1	2	2	1
2	2	2	2	2	2	2	2	2	2
1	2	2	1	2	2	1	2	2	1
1	2	2	2	2	2	2	2	2	1
1	1	2	2	2	1	1	2	2	1
2	2	2	2	2	1	1	2	2	1
2	2	2	2	2	1	1	2	2	1
2	2	2	1	2	2	1	2	2	2
2	2	2	2	2	2	1	2	2	1
2	1	2	1	2	1	1	2	2	1
2	2	2	2	2	2	1	2	2	1
2	2	2	1	2	1	1	2	2	2
2	2	2	2	2	2	1	2	2	1
2	2	2	2	2	1	1	2	2	1
1	2	2	2	2	2	2	2	2	2
2	2	2	1	2	1	2	2	2	1
2	2	2	2	2	1	2	2	2	2
2	2	2	1	2	1	2	2	2	2
2	2	2	2	2	1	2	2	2	1
2	2	2	1	2	1	2	2	2	1
2	2	2	1	2	2	2	2	2	1
2	2	2	1	2	1	2	2	2	1

2	2	1	1	2	1	2	2	2	2
2	2	2	1	1	1	2	2	2	2
2	2	2	1	2	1	1	1	1	1
2	2	2	1	2	1	1	2	2	1
2	2	2	1	2	1	2	2	2	2
1	2	2	1	2	2	2	2	2	2
2	2	2	1	2	1	2	2	2	1
2	2	2	2	2	1	2	2	2	1
2	2	2	1	2	2	2	2	2	1
2	2	1	1	1	2	1	2	2	2
2	2	2	1	2	2	2	2	2	1
2	2	2	1	2	2	2	2	2	1
2	2	2	1	2	1	2	2	2	2
2	2	2	2	2	1	2	2	2	1
2	2	2	1	2	2	2	2	2	1
2	2	1	1	2	2	1	2	2	2
2	2	2	2	2	2	2	2	2	1
2	2	2	1	2	2	2	2	2	2
2	1	2	1	2	2	1	2	2	1
2	2	1	2	2	2	2	2	2	1
2	2	2	1	2	1	1	2	2	1
2	2	2	1	2	1	2	2	2	2
2	2	2	2	1	2	1	2	2	1
2	2	2	2	1	2	1	2	2	1
2	2	2	2	1	2	1	2	2	1
2	2	1	1	2	2	2	2	2	1
2	2	2	1	2	2	2	2	2	1
2	2	2	1	2	2	2	2	2	1
2	2	2	1	2	2	2	2	2	1
2	2	2	2	2	2	2	2	2	2
2	2	2	1	1	2	1	1	1	1
2	2	2	1	1	1	2	2	2	1
2	2	2	1	2	2	2	2	2	1
2	2	2	1	1	2	2	2	2	1
2	2	2	1	2	2	1	2	2	1
2	2	2	1	1	2	2	2	2	1
2	2	2	2	1	2	2	2	2	1
2	2	1	1	2	1	2	2	2	1
2	2	2	2	1	1	2	2	2	1
2	2	2	2	1	2	2	2	2	1
1	2	2	1	2	1	1	2	2	1
2	2	2	1	2	1	2	2	2	1
2	2	2	1	2	1	1	2	2	1
2	2	2	2	2	2	2	2	2	2
2	2	2	1	2	2	2	2	2	2
1	1	2	1	1	1	2	2	2	1
2	2	2	2	2	2	2	2	2	1

2	2	2	2	2	2	2	2	2	1
1	2	1	2	2	2	2	2	2	2
2	2	2	2	2	1	1	2	2	2
2	2	2	2	2	1	1	2	2	2
2	2	2	2	2	1	1	2	2	1
1	1	2	1	1	2	1	1	1	1
2	2	1	2	2	2	2	2	2	2
2	1	2	1	2	1	1	2	2	1
2	2	1	2	2	2	2	2	2	2
2	2	2	1	2	2	2	2	2	1
2	2	2	1	2	2	2	2	2	2
2	2	2	1	2	2	2	2	2	2
1	2	2	2	2	2	2	2	2	1
1	2	2	1	1	2	1	2	2	1
2	2	2	1	2	1	1	2	2	1
2	1	2	2	2	1	1	2	2	1
1	2	2	1	1	2	2	2	2	1
2	2	2	1	2	2	2	2	2	1
2	2	2	2	2	2	2	2	2	2
2	2	2	2	2	2	2	2	2	2
2	2	1	1	2	2	1	2	2	1
2	2	2	2	2	1	2	2	2	1
2	2	2	2	2	1	2	2	2	1
2	2	2	2	1	2	2	2	2	1
2	2	2	2	1	2	2	2	2	1
2	2	2	2	2	2	2	2	2	1
2	2	2	2	2	2	2	2	2	1
2	2	2	1	2	2	1	2	2	1
2	2	2	2	2	2	2	2	2	1
2	2	2	1	2	2	1	2	2	1
2	2	2	2	2	1	1	2	2	1
2	2	2	2	2	2	2	2	2	1
2	2	2	2	2	1	2	2	2	1
2	2	2	2	2	2	2	2	2	1
2	2	2	2	2	2	2	2	2	2
2	2	2	1	2	2	2	2	2	1
2	2	2	1	2	2	2	2	2	1
2	2	2	1	2	2	2	2	2	1
2	2	2	1	1	1	1	2	2	1
2	2	2	1	1	1	1	2	2	1
1	2	2	2	2	2	2	2	2	1
1	2	2	2	2	1	2	2	2	1
2	2	2	2	2	2	2	2	2	2
2	2	2	1	2	2	2	2	2	1
2	2	2	2	2	2	2	2	2	1
2	2	2	2	2	2	2	2	2	2
2	2	2	2	2	2	2	2	2	1

2	2	2	2	1	2	2	2	2	2
2	2	2	1	2	2	2	2	2	1
2	2	2	2	2	2	2	2	2	1
2	2	2	1	2	1	2	2	2	1
2	2	2	1	2	1	2	2	2	1
2	2	2	2	2	2	2	2	2	1
2	2	1	1	2	2	1	2	2	1
2	2	1	2	2	2	2	2	2	1
2	2	2	1	2	2	2	2	2	1
2	2	2	2	2	1	1	2	2	2
2	2	2	2	1	2	2	2	2	2
2	2	2	1	2	2	2	2	2	2
2	2	2	1	2	2	2	2	2	1
2	2	2	1	2	2	1	2	2	2
2	2	2	1	2	2	2	2	2	1
2	2	2	2	2	2	2	2	2	2
2	2	2	2	2	2	2	2	2	1
2	2	2	2	2	2	2	2	2	1
2	2	2	2	2	2	2	2	2	1
2	2	2	1	2	2	2	2	2	1
2	2	2	2	2	1	2	2	2	1
1	1	2	2	1	2	2	2	2	1
1	1	2	2	2	2	2	2	2	1
2	2	1	2	2	2	2	2	2	1
2	2	2	2	2	1	1	2	2	2
2	2	2	2	2	2	2	2	2	2
2	2	2	2	2	2	2	2	2	2
1	2	2	1	2	2	1	2	2	1
2	2	1	2	2	2	1	2	2	2
2	2	2	2	2	2	1	2	2	1
2	2	2	2	2	1	2	2	2	1
2	2	2	2	2	1	2	2	2	1
2	2	2	2	2	2	2	2	2	1
2	2	2	2	2	1	2	2	2	1
2	2	2	2	2	2	2	2	2	1
2	2	2	1	2	2	2	2	2	1
2	2	2	1	1	2	2	2	2	2
2	2	2	2	2	1	1	2	2	1
2	2	2	2	2	2	2	2	2	1
2	2	2	2	2	2	2	2	2	1
2	2	2	2	2	1	1	2	2	1
2	2	2	2	2	2	2	2	2	1
2	2	2	1	2	1	2	2	2	1
2	2	2	1	2	1	2	2	2	1
2	2	1	1	2	2	2	2	2	2
2	2	2	2	2	2	2	2	2	1
2	2	2	2	2	2	2	2	2	1

2	2	2	2	2	2	2	2	2	1
2	2	2	1	2	2	2	2	2	1
2	2	2	1	2	1	2	1	2	1
2	2	2	1	2	2	2	2	2	1
1	1	1	2	2	2	2	2	2	1
2	2	2	2	2	2	2	2	2	1
2	2	2	2	2	2	2	2	2	1
2	2	2	1	2	2	2	2	2	1
2	2	2	1	2	2	2	2	2	1
2	2	2	1	2	2	2	2	1	1
1	2	2	2	1	1	1	2	2	1
2	2	2	2	2	2	2	2	2	2
2	2	1	1	2	1	2	2	2	1
2	1	2	1	2	1	2	2	2	1
2	2	1	2	2	1	2	2	2	1
2	2	2	1	2	2	2	2	2	2
2	2	2	1	2	2	2	2	2	2
2	2	2	1	2	2	2	2	2	2
2	2	2	1	2	1	2	2	2	2
2	2	2	1	1	1	2	2	2	1
2	2	1	2	2	2	2	2	2	2
2	2	2	1	2	1	2	2	2	1
2	2	2	2	1	1	1	2	2	1
2	2	1	2	2	2	2	2	2	2
2	2	1	2	2	2	2	2	2	2
2	2	2	2	2	1	2	2	2	1
2	2	1	2	2	1	2	2	2	1
2	2	2	2	2	1	2	2	2	1
2	2	1	2	2	1	2	2	2	1
2	2	1	1	2	1	2	2	2	1
2	2	2	1	2	1	2	2	2	1
2	2	1	2	2	2	2	2	2	1
2	2	1	2	2	2	2	2	2	1
1	2	2	1	2	1	2	2	2	1
2	2	2	1	2	1	1	2	2	1
2	2	2	1	2	1	2	2	2	1
2	2	1	1	2	1	1	2	2	2
2	2	2	2	2	2	2	2	2	1
2	2	2	2	1	1	2	2	2	1
2	2	2	1	2	1	2	2	2	1
2	2	1	1	2	1	2	2	2	1
2	2	1	1	2	1	2	2	2	1
2	2	2	2	2	1	2	2	2	2
2	2	2	2	2	1	2	2	2	2
1	2	2	2	2	2	1	2	2	1
1	2	2	2	2	2	2	2	2	1
2	2	2	2	2	2	2	2	2	1

1	1	1	2	2	2	2	2	2	1
2	2	2	1	2	2	2	2	2	2
2	2	2	2	2	2	2	2	2	1
2	2	2	1	2	1	2	2	2	1
2	2	2	2	2	2	2	2	2	2
2	2	2	2	2	1	2	2	2	1
2	2	2	2	2	2	2	2	2	1
2	2	2	2	2	2	2	2	2	1
2	2	2	2	2	2	2	2	2	1
2	2	2	2	2	2	2	2	2	1
2	2	2	2	2	2	2	2	2	2
2	2	2	2	2	2	2	2	2	2
2	2	2	2	2	1	2	2	2	1
2	2	2	2	2	2	2	2	2	1
2	2	2	2	2	1	2	2	2	1
2	2	2	2	1	2	2	1	2	2
2	2	2	2	2	1	2	2	2	1
2	2	2	2	2	1	2	2	2	1
2	2	2	2	2	2	2	2	2	1
2	2	2	1	2	2	2	2	2	1
2	2	2	1	2	1	1	2	2	1
2	2	2	2	2	2	2	2	2	1
2	2	2	2	2	1	1	2	2	1
2	2	2	2	2	2	1	2	2	1
2	2	2	1	1	1	1	2	2	1
2	2	2	2	2	2	2	2	2	1
2	2	2	2	2	2	1	2	2	1
2	2	2	2	2	2	2	2	2	1
2	2	2	2	2	1	2	2	2	1
2	2	2	2	2	2	2	2	2	1
2	2	2	1	2	2	1	2	2	1
2	2	2	2	2	2	2	2	2	1
2	2	2	1	2	2	1	2	2	1
2	2	2	2	2	2	2	2	2	1
2	2	2	2	2	1	1	2	1	1
2	2	2	2	2	2	2	2	2	1
2	1	2	2	2	2	1	2	2	1
2	2	2	2	2	1	1	2	2	1
2	2	2	2	2	2	1	2	2	1
2	2	2	1	1	1	1	1	2	1
2	2	2	1	2	1	1	2	2	1
2	2	2	2	2	2	2	2	2	1
2	1	2	2	2	2	1	2	2	1
2	2	2	2	2	2	2	2	2	1
2	2	2	2	2	2	2	2	2	1
1	2	2	1	2	2	1	2	2	1
2	2	2	2	2	1	2	2	2	1

2	2	2	2	2	2	2	2	2	2
2	2	2	2	2	2	2	2	2	1
2	2	2	2	2	2	2	2	2	2
2	2	2	2	2	2	2	2	2	2
1	2	2	2	2	2	2	2	2	1
2	2	2	2	2	2	1	2	2	1
2	2	2	1	2	1	2	2	2	1
2	2	2	1	2	1	2	2	2	1
2	2	2	2	2	1	2	2	2	1
2	2	2	2	2	2	1	2	2	1
2	2	2	1	1	1	1	2	2	1
2	2	2	1	2	2	2	2	2	1
2	2	2	2	2	2	2	2	2	1
2	2	2	2	2	2	2	2	2	2
2	2	2	2	2	2	2	2	2	2
2	2	2	2	2	2	2	2	2	2
2	2	2	2	2	2	2	2	2	2
2	2	2	1	2	1	2	2	2	2
1	2	2	2	2	2	2	2	2	1
2	2	2	1	2	2	2	2	2	1
2	2	2	2	2	2	2	2	2	1
2	2	2	2	2	1	1	2	2	1
2	2	2	2	2	1	2	2	2	2
2	2	1	2	2	2	2	2	2	2
2	2	2	2	2	1	2	2	2	2
2	2	2	2	2	2	2	2	2	1
2	2	2	2	2	1	2	2	2	1
2	2	2	1	2	1	1	2	2	1
2	2	2	1	2	1	2	2	2	1
2	2	2	1	2	2	2	2	2	1
2	2	2	1	2	1	2	2	2	1
2	2	2	1	2	1	2	2	2	1
2	2	2	1	2	1	1	2	2	1
2	2	2	1	2	2	2	2	2	1
2	2	2	1	2	1	2	2	2	1
2	2	2	2	2	2	2	2	2	1
1	2	2	1	2	2	2	2	2	2
2	2	2	2	2	2	2	2	2	1
2	2	2	2	2	2	2	2	2	1
2	2	2	2	2	2	2	2	2	1
2	2	2	1	2	2	2	2	2	1
2	2	2	1	1	1	2	2	2	1
2	2	2	1	2	2	2	2	2	1
2	2	2	2	1	2	2	2	2	2
2	2	2	2	2	2	2	1	2	2
2	2	2	2	1	2	2	2	2	1
2	2	2	1	1	2	2	2	2	1

2	2	2	1	2	1	1	2	2	1
2	2	2	1	2	1	2	2	2	1
2	2	2	1	1	2	2	2	2	1
2	2	2	2	2	2	2	2	2	1
2	2	2	2	2	2	2	2	2	1
2	2	2	2	2	2	2	2	2	1
2	2	2	2	2	1	2	2	2	1
2	2	2	2	2	2	2	2	2	1
2	2	2	1	2	2	2	2	2	1
2	2	2	2	2	2	1	2	2	2
1	2	2	2	2	2	2	2	2	2
2	2	2	1	1	2	2	1	1	1
2	2	2	1	2	1	1	2	2	1
2	2	2	2	2	1	2	2	2	1
2	2	2	1	2	1	1	2	2	1
2	2	2	1	2	2	2	2	2	1
1	2	2	1	2	2	1	2	2	1
2	2	2	2	1	2	2	2	2	1
2	2	2	1	2	2	2	2	2	1
1	2	2	1	2	2	1	2	2	1
2	2	2	1	2	1	1	2	2	1
2	2	2	1	1	2	2	2	2	1
1	2	2	2	2	2	2	2	2	2
2	2	2	2	2	2	2	2	2	2
2	2	2	1	1	1	2	2	2	1
1	2	2	2	2	2	2	2	2	1
2	2	2	1	2	2	2	2	2	2
1	2	2	2	2	2	2	2	2	2
1	2	2	2	2	2	1	2	2	1
1	2	2	2	2	2	1	2	2	1
2	2	2	2	2	2	2	2	2	1
2	2	2	1	1	1	1	2	2	1
2	2	2	2	2	2	2	2	2	2
2	2	2	1	2	1	1	2	2	1
2	2	2	1	1	2	2	2	2	1
2	2	2	1	1	2	2	2	2	1
1	2	2	1	2	2	2	2	2	2
1	2	2	1	2	2	2	2	2	2
2	2	2	2	2	2	2	2	2	1
1	2	2	1	1	1	2	2	2	1
2	2	2	2	2	2	2	2	2	1
2	2	2	2	2	2	2	1	2	1
2	2	2	2	2	2	2	2	2	2
1	2	2	2	2	2	2	2	2	1
2	2	2	2	2	1	2	2	2	1
2	2	2	2	2	1	2	2	2	2
2	2	2	1	2	2	2	2	2	1

2	2	2	2	2	2	2	2	2	1
2	2	2	2	2	2	2	2	2	1
2	2	2	2	2	2	2	2	2	1
2	2	2	2	2	2	2	2	2	1
2	2	1	2	2	2	2	2	2	1
2	2	2	2	2	2	2	2	2	1
2	2	2	1	2	1	2	2	2	1
2	2	2	2	2	2	2	2	2	1
2	2	2	1	2	2	2	2	2	1
2	2	2	2	2	1	2	2	2	1
2	2	2	2	2	2	2	2	2	1
2	2	2	2	2	2	2	2	2	1
2	2	2	2	2	1	2	2	2	1
2	2	2	2	2	2	2	2	2	2
2	2	2	2	2	2	2	2	2	1
2	2	2	2	2	2	2	2	2	1
2	2	2	1	2	1	2	2	2	1
2	2	2	1	2	2	1	2	2	1
2	2	2	1	2	1	2	2	2	1
2	2	2	1	2	2	2	2	2	1
2	2	2	2	2	2	2	2	2	2
2	2	2	2	2	2	2	2	2	1
2	2	2	2	2	2	2	2	2	1
2	2	2	2	2	2	2	2	2	2
2	2	2	2	2	1	2	2	2	2
2	2	2	2	2	2	2	2	2	1
2	2	2	2	2	1	2	2	2	1
1	2	2	1	2	2	2	2	2	2
2	2	2	2	2	2	2	2	2	1
2	2	2	1	2	2	2	2	2	1
2	2	2	2	2	2	2	2	2	1
2	2	2	2	2	1	2	2	2	2
2	2	2	1	2	1	1	2	2	2
2	2	1	2	1	1	1	1	1	1
1	2	2	1	2	2	1	2	2	2
2	2	2	1	1	2	2	2	2	1
2	2	2	2	2	2	2	2	2	2
2	2	2	2	2	1	2	2	2	2
2	2	2	2	2	1	2	2	2	1
2	2	2	2	2	2	2	2	2	1
2	2	2	2	2	2	2	2	2	1
2	2	2	2	2	2	2	2	2	1
2	2	2	2	2	1	2	2	2	1
2	2	1	1	2	1	2	2	2	1
2	2	2	2	2	2	2	2	2	1
2	1	2	2	2	2	2	2	2	1
2	2	2	1	1	2	2	2	2	1

2	2	2	2	2	2	2	2	2	1
2	2	2	2	2	2	2	2	2	1
2	2	2	2	2	1	2	2	2	1
2	2	2	2	2	2	2	2	2	2
2	2	2	2	2	2	1	2	2	1
2	2	2	1	2	1	1	2	2	1
1	2	2	2	2	2	2	2	2	2
2	2	2	2	2	2	2	2	2	1
2	2	2	1	2	1	1	2	2	1
2	2	2	2	2	2	2	2	2	1
2	2	2	2	2	2	2	2	2	1
2	2	2	2	2	2	2	2	2	1
1	2	2	1	1	2	2	2	2	1
1	2	2	1	2	1	2	2	2	1
2	2	2	2	2	1	2	2	2	2
2	2	2	1	2	1	1	1	1	1
2	2	2	2	2	1	2	2	2	1
2	2	2	2	2	1	2	2	2	1
2	2	2	2	2	1	2	2	2	2
2	2	2	1	2	1	2	2	2	1
2	2	2	2	2	2	2	2	2	1
2	2	2	1	2	1	1	1	1	1
2	2	1	2	2	2	1	1	1	1
2	2	2	1	2	1	1	2	2	1
2	2	2	1	1	2	2	2	2	1
2	2	1	2	2	1	2	2	2	1
2	2	1	2	2	1	2	2	2	1
2	2	2	2	2	2	2	2	2	1
2	2	2	1	1	1	2	2	2	2
2	2	1	1	2	1	1	2	2	1
2	2	1	2	2	1	2	2	2	2
2	2	1	2	2	1	2	2	2	1
2	2	2	2	2	1	2	2	2	1
2	2	1	2	2	2	2	2	2	2
2	2	2	2	2	2	2	2	2	2
1	2	2	2	2	1	2	2	2	1
2	2	2	2	2	2	2	2	2	1
1	1	2	1	2	1	2	2	2	1
2	2	2	2	1	1	2	2	2	1
2	2	1	1	2	2	2	2	2	1
2	2	2	1	2	1	2	2	2	1
2	2	2	1	2	2	2	1	2	1
2	2	2	2	1	1	1	2	2	1
2	2	2	2	2	1	2	2	2	1
2	2	2	1	2	2	2	2	2	2
1	2	2	2	2	2	2	2	2	1

Deep freez	Dry room s	Cupboard	Open fire c	Open fire i	Gas stove	Paraffin st	Electric stc	Coal stove	Gel stove
1	2	2	1	1	2	2	1	2	2
1	2	2	1	1	2	2	1	2	2
1	2	2	1	2	2	2	1	2	2
1	1	1	1	1	2	2	2	2	2
2	2	1	1	2	2	2	2	2	2
1	1	1	1	2	2	2	1	2	2
1	2	2	1	2	2	2	1	2	2
1	2	2	1	2	2	2	1	2	2
1	2	1	1	2	2	2	1	2	2
1	1	1	1	2	2	2	2	2	2
2	2	2	1	1	2	2	1	2	2
1	1	1	1	2	1	2	1	2	2
2	1	1	1	2	2	2	1	2	2
1	1	2	1	2	2	2	1	2	2
2	2	2	1	1	2	2	1	2	2
1	1	1	1	1	1	2	1	2	2
1	1	1	1	1	1	2	1	2	2
1	2	1	1	2	2	2	1	2	2
1	2	2	1	2	2	2	1	2	2
1	2	1	1	2	2	2	1	2	2
2	2	1	1	1	2	2	2	2	2
1	1	1	1	2	2	2	1	2	2
1	1	1	1	2	2	2	1	2	2
2	1	1	1	1	2	2	1	2	2
1	2	2	1	2	2	2	1	2	2
1	2	1	1	2	2	2	1	2	2
1	1	1	1	1	2	2	1	2	2
1	1	1	1	2	2	2	1	2	2
2	2	1	1	2	2	2	2	2	2
1	2	2	1	1	2	2	2	2	2
2	2	1	1	2	2	2	2	2	2
1	1	1	1	2	2	2	1	2	2
1	2	1	1	1	2	2	1	2	2
1	2	2	1	1	2	1	1	2	2
1	2	1	1	1	2	2	1	2	2
1	1	1	1	2	2	2	1	2	2
1	2	2	1	2	2	2	2	2	2
1	2	2	2	1	2	2	2	2	2
1	2	2	1	1	2	2	2	2	2
1	1	2	1	2	2	2	1	2	2
1	1	2	1	2	2	2	1	2	2
1	2	1	1	2	2	2	1	2	2
2	1	1	1	2	2	2	2	2	2
1	2	1	1	1	2	1	2	2	2
1	2	2	1	1	2	2	1	2	2
1	2	2	2	1	2	2	2	2	2

1	2	2	1	2	2	2	2	2	2
1	2	2	1	2	2	2	2	2	2
1	1	1	1	1	2	2	2	2	2
1	2	1	2	1	2	2	1	2	2
1	2	2	1	2	2	2	2	2	2
1	1	1	1	1	2	2	1	2	2
1	2	2	1	1	2	2	1	2	2
1	1	1	1	2	2	2	1	2	2
1	1	1	1	1	2	2	1	2	2
1	1	1	1	1	2	2	1	2	2
1	1	1	1	1	2	2	1	2	2
1	1	1	1	2	2	2	1	2	2
1	1	1	1	1	2	2	1	2	2
1	1	1	1	1	2	2	1	2	2
1	1	2	1	2	2	2	1	2	2
1	2	1	1	1	2	2	1	2	2
1	1	1	2	1	2	2	1	2	2
1	2	2	1	2	2	2	1	2	2
1	2	2	1	1	2	2	1	2	2
1	2	1	1	1	2	2	2	2	2
1	2	1	1	1	2	2	1	2	2
1	1	1	1	1	2	2	1	2	2
1	1	1	1	1	2	2	1	2	2
1	2	2	1	1	2	2	1	2	2
1	2	2	1	1	2	2	1	2	2
1	1	1	1	1	2	2	2	2	2
1	2	1	1	1	2	2	1	2	2
1	1	1	1	1	2	2	1	2	2
1	2	2	2	1	2	2	2	2	2
1	1	2	1	1	2	2	2	2	2
1	2	2	1	1	2	2	2	2	2
1	2	2	1	1	2	2	2	2	2
1	2	1	1	1	2	2	1	2	2
1	2	2	1	1	2	2	1	2	2
1	1	1	1	2	2	2	1	2	2
1	1	1	1	1	2	2	2	2	2
1	1	1	1	1	2	2	1	2	2
1	1	2	1	1	2	2	2	2	2
1	1	1	1	1	2	2	1	2	2
2	2	1	1	2	2	2	2	2	2
1	2	1	1	1	2	2	1	2	2
1	2	2	1	2	2	2	1	2	2
1	2	2	1	1	2	2	1	2	2
1	2	2	1	2	2	2	1	2	2
1	2	2	1	1	2	2	1	2	2
1	2	2	1	1	2	2	1	2	2
1	1	2	1	1	2	2	1	2	2

2	1	2	1	1	2	2	2	2	2
1	2	2	2	1	2	2	2	2	2
1	2	2	1	1	2	2	1	2	2
1	1	1	1	1	2	2	1	2	2
1	2	2	1	1	2	2	1	2	2
2	2	2	2	1	2	2	2	2	2
2	1	1	1	1	2	2	1	2	2
1	2	1	2	1	2	2	2	2	2
1	2	2	2	1	2	2	2	2	2
1	2	1	1	2	2	2	1	2	2
1	1	1	1	1	2	2	2	2	2
1	1	1	1	1	2	2	2	2	2
1	2	2	1	2	2	2	1	2	2
1	2	1	2	2	2	2	1	2	2
1	1	1	1	1	2	2	2	2	2
1	2	1	1	1	2	2	2	2	2
1	2	1	1	2	2	2	2	2	2
1	2	2	1	1	2	2	2	2	2
1	1	1	1	1	2	2	1	2	2
1	1	1	1	1	2	2	1	2	2
2	2	2	1	1	2	2	2	2	2
1	2	2	1	2	2	2	1	2	2
1	2	1	1	1	2	2	1	2	2
1	2	1	1	1	2	2	1	2	2
1	2	1	1	1	2	2	1	2	2
1	2	1	1	1	2	2	1	2	2
1	2	1	1	1	2	2	1	2	2
1	2	1	1	1	2	2	2	2	2
1	1	1	1	1	2	2	2	2	2
1	2	2	1	2	2	2	1	2	2
1	1	1	1	1	2	2	1	2	2
1	2	1	2	1	2	2	1	2	2
1	2	1	1	1	2	2	1	2	2
1	2	2	1	2	2	2	1	2	2
1	2	2	1	2	1	2	1	2	2
1	2	2	1	2	2	1	2	2	2
1	2	2	2	2	2	2	1	2	2
1	2	2	1	1	2	2	1	2	2
1	2	2	1	2	2	2	1	2	2
1	2	2	1	1	2	2	2	2	2
1	2	2	1	1	1	2	1	2	2
1	2	2	2	1	2	2	1	2	2
1	1	1	1	1	2	2	1	2	2
1	2	1	1	2	2	2	2	2	2
1	2	1	1	2	2	2	1	2	2
1	2	1	2	2	2	2	1	2	2
1	2	1	1	2	2	2	1	2	2

1	2	2	2	1	2	2	2	2	2
2	2	2	1	2	2	2	2	2	2
2	2	2	1	2	2	2	2	2	2
2	2	2	1	2	2	2	2	2	2
1	1	1	1	1	2	2	2	2	2
1	2	1	1	1	2	2	1	2	2
2	2	2	1	2	2	2	2	2	2
1	2	1	1	1	2	2	1	2	2
1	2	2	2	1	2	2	1	2	2
1	2	2	1	2	2	2	1	2	2
1	2	2	1	2	2	2	1	2	2
1	2	2	1	2	2	2	1	2	2
1	2	2	1	2	2	2	2	2	2
1	2	1	1	1	2	2	1	2	2
1	2	1	2	1	2	2	1	2	2
1	2	1	2	1	2	2	1	2	2
1	2	1	1	1	2	2	1	2	2
1	2	1	1	1	2	2	1	2	2
2	2	1	1	2	2	2	1	2	2
2	2	2	1	1	2	2	1	2	2
1	2	2	1	2	2	2	1	2	2
1	2	2	1	2	2	2	1	2	2
1	2	2	1	2	2	2	1	2	2
2	2	2	2	2	2	2	1	2	2
2	2	2	2	2	2	2	1	2	2
1	2	2	1	2	2	2	1	2	2
1	2	1	1	2	2	2	1	2	2
1	1	1	1	1	2	2	1	2	2
1	1	1	1	2	2	2	1	2	2
1	1	1	1	2	2	2	1	2	2
1	2	2	1	2	2	2	1	2	2
1	1	1	2	2	2	2	1	2	2
1	2	2	2	2	2	2	1	2	2
2	2	1	1	1	2	2	1	2	2
2	2	2	1	2	2	2	2	2	2
1	1	2	1	2	2	2	2	2	2
1	2	2	1	1	2	2	1	2	2
1	1	2	1	2	2	2	2	2	2
1	1	1	1	1	2	2	1	2	2
1	1	1	1	1	2	2	1	2	2
1	1	2	1	2	2	2	2	2	2
2	2	2	1	2	2	2	1	2	2
2	1	1	1	1	2	2	2	2	2
1	2	1	1	2	2	2	1	2	2
1	2	1	2	1	2	2	1	2	2
2	2	2	1	2	2	2	1	2	2
1	1	1	1	1	2	2	1	2	2

2	2	2	1	2	2	2	2	2	2
1	2	1	1	2	2	2	1	2	2
1	1	2	1	1	2	2	2	2	2
2	2	1	1	2	2	2	1	2	2
2	2	1	1	2	2	2	1	2	2
1	2	1	1	1	2	2	1	2	2
1	2	1	1	2	2	2	1	2	2
1	2	2	1	2	2	2	1	2	2
1	2	2	1	1	2	2	2	2	2
2	1	1	1	2	2	2	2	2	2
1	2	1	1	2	2	2	1	2	2
1	2	2	1	1	2	2	1	2	2
1	1	2	1	1	2	2	1	2	2
2	2	1	1	1	2	2	2	2	2
1	2	1	1	1	2	2	2	2	2
2	2	2	1	2	2	2	2	2	2
1	2	1	1	2	2	2	1	2	2
1	2	2	1	2	2	2	2	2	2
1	1	2	1	1	2	2	1	2	2
1	1	1	1	2	2	2	1	1	2
1	2	2	1	2	2	2	2	2	2
1	2	1	1	2	2	2	1	2	2
1	1	1	1	2	2	2	1	2	2
2	2	2	1	1	2	2	2	2	2
1	2	2	1	2	2	2	1	2	2
1	2	2	2	1	2	2	2	2	2
2	2	1	1	2	2	2	2	2	2
1	1	2	1	2	2	2	1	2	2
1	2	2	1	2	2	2	1	2	2
1	1	1	1	2	2	2	1	2	2
2	2	2	1	1	2	2	1	2	2
1	2	2	1	2	2	2	1	2	2
1	2	1	2	1	2	2	1	2	2
1	2	2	1	2	2	2	1	2	2
1	1	2	1	1	2	2	1	2	2
1	1	2	1	2	2	2	1	2	2
1	2	1	1	1	2	2	1	2	2
1	1	1	1	1	2	2	1	2	2
1	2	2	1	2	2	2	2	2	2
1	2	1	1	1	2	2	1	2	2
1	1	1	1	2	2	2	1	2	2
1	2	2	1	1	2	2	1	2	2
1	1	1	1	1	2	2	1	2	2
1	1	1	1	1	2	2	1	2	2
2	2	2	1	2	2	2	1	2	2
1	2	2	1	2	2	2	1	2	2
2	2	1	1	1	2	2	1	2	2

2	2	1	1	1	2	2	1	2	2
1	1	2	1	1	2	2	1	2	2
1	2	2	1	1	2	2	1	2	2
1	2	2	1	1	2	2	1	2	2
1	1	2	1	1	2	2	1	2	2
1	2	2	1	2	2	2	1	2	2
1	1	2	1	2	2	2	1	2	2
1	1	2	1	2	2	2	1	2	2
1	2	2	1	2	2	2	1	2	2
1	1	1	1	2	2	2	1	2	2
1	2	1	1	1	2	2	1	2	2
1	2	2	1	2	2	2	2	2	2
1	2	2	2	1	2	2	1	2	2
1	2	2	2	1	2	2	1	2	2
1	2	2	1	2	2	2	2	2	2
1	2	2	1	2	2	2	2	2	2
2	2	2	1	1	2	2	2	2	2
2	2	2	1	1	2	2	2	2	2
1	2	2	1	2	2	2	2	2	2
1	1	1	1	1	2	2	1	2	2
2	2	2	1	2	2	2	2	2	2
1	2	2	1	2	2	2	1	2	2
1	1	1	1	1	2	2	1	2	2
2	2	2	1	2	2	2	2	2	2
2	2	2	1	2	2	2	2	2	2
1	2	2	2	1	2	2	1	2	2
1	2	2	1	2	2	2	2	2	2
2	2	2	1	1	2	2	1	2	2
2	2	2	1	2	2	2	1	2	2
1	2	2	1	1	2	2	1	2	2
1	2	2	1	2	2	2	1	2	2
1	2	2	1	2	2	2	2	2	2
1	1	2	1	1	2	2	1	2	2
1	2	2	1	1	2	2	1	2	2
1	1	1	1	1	2	2	1	2	2
1	2	2	1	2	2	2	2	2	2
1	2	2	2	1	2	2	2	2	2
1	1	2	1	1	2	2	1	2	2
1	2	1	1	1	2	2	1	2	2
2	2	2	1	1	2	2	2	2	2
1	2	2	1	1	2	2	2	2	2
1	2	2	1	2	2	2	2	2	2
1	2	1	1	1	2	2	1	2	2
1	2	1	1	1	2	2	1	2	2
2	2	2	1	2	2	2	2	2	2
1	2	1	2	1	2	2	1	2	2
2	2	2	1	2	2	2	2	2	2

1	2	1	1	2	2	2	1	2	2
2	2	2	1	2	2	2	2	2	2
1	2	2	1	2	2	2	2	2	2
2	2	1	1	2	1	2	1	2	2
1	2	2	1	1	2	2	2	2	2
1	2	1	1	1	2	2	1	2	2
2	2	2	1	2	2	2	2	2	2
1	2	2	1	2	2	2	1	2	2
1	2	2	1	2	2	2	2	2	2
1	2	2	1	2	2	2	2	2	2
1	2	2	1	2	2	2	2	2	2
1	2	2	1	2	2	2	2	2	2
1	2	1	1	1	2	2	1	2	2
1	2	2	1	2	2	2	1	2	2
2	2	1	1	1	2	2	1	2	2
1	2	1	1	2	2	2	1	2	2
2	2	1	1	2	2	2	1	2	2
1	2	2	1	1	2	2	1	2	2
1	2	2	1	2	2	2	1	2	2
1	2	1	2	1	2	2	1	2	2
1	2	1	1	2	2	2	2	2	2
1	1	1	1	1	2	2	1	2	2
1	1	1	1	1	2	2	1	2	2
1	1	1	1	1	2	2	2	2	2
1	1	1	1	1	2	2	1	2	2
1	2	2	2	1	2	2	2	2	2
1	1	1	1	1	1	2	1	2	2
1	2	1	2	1	2	2	2	2	2
1	1	1	1	2	2	2	2	2	2
1	2	2	2	1	2	2	2	2	2
1	2	1	1	2	2	2	2	2	2
1	2	1	1	2	2	2	1	2	2
1	2	1	1	2	2	2	1	2	2
1	2	2	2	1	2	2	1	2	2
1	1	1	1	1	2	2	1	2	2
2	2	1	1	2	2	2	2	2	2
1	1	1	2	1	2	2	2	2	2
1	1	1	1	1	2	2	1	2	2
1	1	1	1	1	2	2	2	2	2
1	1	1	1	1	2	2	1	2	2
1	2	1	1	2	2	2	2	2	2
1	2	1	1	2	2	2	2	2	2
2	1	1	2	1	2	2	1	2	2
1	2	1	1	1	2	2	1	2	2
1	2	1	2	1	2	2	1	2	2
1	2	1	1	1	2	2	2	2	2
1	2	1	1	1	2	2	1	2	2

1	2	1	1	1	2	2	1	2	2
1	2	1	2	1	2	2	1	2	2
1	2	2	1	2	2	2	2	2	2
1	2	2	1	2	2	2	2	2	2
1	2	1	1	1	2	2	2	2	2
1	2	1	1	1	2	1	2	2	1
1	2	1	1	1	2	2	1	2	2
1	2	1	1	1	2	2	1	2	2
1	2	1	1	1	2	2	2	2	2
2	2	2	1	2	2	2	2	2	2
1	1	1	1	2	2	2	2	2	2
1	2	1	1	1	2	2	2	2	2
1	2	1	1	1	2	2	1	2	2
2	2	1	1	1	2	2	1	2	2
1	2	1	1	2	2	2	1	2	2
2	2	1	1	1	2	2	2	2	2
2	2	1	1	1	2	2	2	2	2
1	2	1	1	1	2	2	1	2	2
1	2	1	1	1	2	2	2	2	2
1	2	1	2	1	2	2	1	2	2
1	2	2	1	2	2	2	2	2	2
1	1	1	1	1	2	2	1	2	2
1	2	1	1	1	2	2	1	2	2
1	2	2	1	2	2	2	2	2	2
2	2	2	1	1	2	2	2	2	2
1	1	1	1	2	2	2	1	2	2
1	2	1	1	1	2	2	1	2	2
1	2	1	1	1	2	1	1	2	2
1	2	1	1	1	2	2	1	2	2
2	2	1	1	1	2	2	1	2	2
1	2	2	1	1	2	2	1	2	2
2	2	2	2	1	2	2	1	2	2
1	2	2	2	1	2	2	2	2	2
1	2	1	2	1	2	2	1	2	2
1	1	1	1	1	2	2	1	2	2
1	2	1	1	2	2	2	2	2	2
2	1	2	1	1	2	2	1	2	2
1	2	1	2	1	2	2	1	2	2
1	2	1	2	1	2	2	2	2	2
1	2	2	1	2	2	2	2	2	2
2	2	2	1	1	2	2	2	2	2
2	2	1	1	2	2	2	1	2	2
2	2	2	1	1	2	2	2	2	2
1	2	1	1	2	2	2	2	2	2
1	2	2	1	2	2	2	2	2	2
1	2	1	1	1	2	2	2	2	2
1	2	1	1	1	2	2	1	2	2

1	1	1	1	1	2	2	1	2	2
1	2	2	1	2	2	2	2	2	2
1	2	1	1	1	2	2	2	2	2
1	1	1	1	1	2	2	2	2	2
1	1	1	1	1	2	2	2	2	2
1	1	1	1	1	2	2	2	2	2
1	2	1	1	2	2	2	1	2	2
2	2	1	1	1	2	2	1	2	2
1	2	1	2	1	2	2	2	2	2
1	2	2	1	1	2	2	1	2	2
1	2	1	1	2	2	2	1	2	2
1	1	1	1	1	2	2	1	2	2
1	2	1	1	1	2	2	1	2	2
1	2	2	2	1	2	2	1	2	2
1	2	1	1	1	2	2	1	2	2
1	2	1	1	1	2	2	2	2	2
1	1	1	1	2	1	2	1	2	2
1	2	1	1	1	2	2	1	2	2
1	2	2	2	1	2	2	2	2	2
1	1	1	1	2	1	2	1	2	2
1	1	1	1	1	2	2	1	2	2
1	2	1	1	2	2	2	1	2	2
1	2	1	1	2	2	2	1	2	2
1	2	1	1	1	2	2	2	2	2
1	2	1	1	2	2	2	1	2	2
1	2	1	1	2	2	2	1	2	2
1	2	2	1	2	2	2	1	2	2
2	2	1	1	2	2	2	2	2	2
1	2	1	2	1	2	2	1	2	2
1	2	1	2	1	2	2	1	2	2
2	2	2	1	2	2	2	1	2	2
1	2	1	1	1	2	2	1	2	2
2	2	1	1	2	2	2	2	2	2
1	1	1	1	1	2	2	1	2	2
1	2	1	1	2	2	2	1	2	2
1	1	1	1	1	2	2	1	2	2
1	2	1	1	1	2	2	1	2	2
1	2	1	1	1	2	2	1	2	2
1	2	1	1	2	2	2	1	2	2
1	2	2	1	2	2	2	1	2	2
2	2	2	1	2	2	2	2	2	2
2	2	2	1	2	2	2	1	2	2
2	2	2	1	2	2	2	2	2	2
1	2	1	1	2	2	2	1	2	2
2	2	2	1	2	2	2	1	2	2
1	2	2	1	1	2	2	1	2	2
1	2	1	1	1	2	2	1	2	2

1	2	2	1	2	2	2	1	2	2
1	2	2	1	2	2	2	1	2	2
1	2	2	2	2	2	2	1	2	2
1	2	2	2	2	2	2	1	2	2
1	1	2	1	2	2	2	2	2	2
1	1	1	1	1	2	2	1	2	2
1	1	2	1	1	2	2	1	2	2
1	2	2	1	2	2	2	1	2	2
1	2	1	1	2	2	2	1	2	2
1	2	1	1	1	2	2	1	2	2
1	2	2	1	1	2	2	1	2	2
1	2	1	1	1	2	2	1	2	2
2	2	2	1	1	2	2	1	2	2
2	2	2	1	2	2	2	1	2	2
1	1	1	1	2	2	2	1	2	2
1	1	2	1	2	2	2	1	2	2
1	1	1	1	1	2	2	2	2	2
1	2	2	1	2	2	2	1	2	2
1	1	1	1	1	2	2	1	2	2
1	2	2	1	2	2	2	2	2	2
1	2	2	1	2	2	2	1	2	2
1	2	2	1	2	2	2	1	2	2
1	2	2	1	2	2	2	1	2	2
2	2	1	1	2	2	2	1	2	2
1	2	2	1	2	2	2	1	2	2
1	2	2	1	2	2	2	2	2	2
2	2	2	1	2	2	2	1	2	2
1	2	1	1	2	2	2	1	2	2
1	2	1	1	2	2	2	1	2	2
1	2	1	1	2	2	2	1	2	2
1	2	2	1	2	2	2	1	2	2
2	2	2	1	2	2	2	2	2	2
1	1	1	1	2	2	2	1	2	2
1	1	1	1	2	2	2	1	2	2
2	2	1	1	1	2	2	2	2	2
1	2	1	2	1	2	2	2	2	2
1	2	2	1	2	2	2	1	2	2
1	2	2	1	2	2	2	2	2	2
2	2	1	1	1	2	2	1	2	2
1	2	2	1	2	2	2	2	2	2
1	1	1	1	1	2	2	1	2	2
1	1	1	1	1	2	2	1	2	2
2	2	1	1	1	2	2	2	2	2
1	1	1	2	2	2	2	1	2	2
1	1	1	1	2	2	2	1	2	2
1	1	1	1	2	2	2	1	2	2
1	1	1	1	1	2	2	1	2	2

1	2	2	1	2	2	2	2	2	2
1	2	1	1	2	2	2	1	2	2
1	1	1	1	1	2	2	1	2	2
1	2	1	1	2	2	2	1	2	2
1	1	2	1	1	2	2	2	2	2
1	1	1	1	1	2	2	1	2	2
1	2	1	1	2	2	2	1	2	2
1	2	2	1	2	2	2	2	2	2
1	1	1	1	1	2	2	1	2	2
1	2	2	1	2	2	2	1	2	2
1	2	1	2	2	2	2	1	2	2
1	2	1	2	1	2	2	1	2	2
1	2	2	1	2	2	2	2	2	2
1	2	2	1	2	2	2	2	2	2
2	2	2	1	2	2	2	1	2	2
1	2	2	2	2	2	2	1	2	2
1	2	2	1	1	2	2	1	2	2
1	2	2	1	1	2	2	1	2	2
1	2	2	1	2	2	2	2	2	2
2	2	1	1	1	2	2	1	2	2
1	2	1	1	2	2	2	1	2	2
1	2	2	2	2	2	2	1	2	2
1	2	1	2	1	2	2	2	2	2
1	2	1	1	1	2	2	1	2	2
1	2	1	1	1	2	2	2	2	2
1	2	2	1	2	2	2	1	2	2
1	2	2	1	2	2	2	1	2	2
1	2	1	1	2	2	2	1	2	2
2	1	1	1	1	2	2	2	2	2
1	2	2	1	1	2	2	1	2	2
2	2	2	1	1	2	2	2	2	2
2	2	2	1	2	2	2	1	2	2
1	2	2	1	1	2	2	2	2	2
2	2	1	1	1	2	2	2	2	2
2	2	1	1	1	2	2	2	2	2
1	2	2	2	1	2	2	1	2	2
1	2	1	1	2	2	2	1	2	2
2	2	1	1	1	2	2	2	2	2
1	2	1	1	1	2	2	1	2	2
2	2	1	1	2	2	2	1	2	2
1	2	1	1	1	2	2	1	2	2
1	2	2	1	1	2	2	2	2	2
1	1	1	1	1	2	2	1	2	2
2	2	2	1	2	2	2	2	2	2
1	2	2	1	1	2	2	2	2	2
1	2	1	1	2	2	2	1	2	2

Microwav	Wonder b	flu	throat	asthma	pneumoni	TB	covid	stomach a	fever
1	2	2	2	2	2	2	2	2	2
1	2	2	2	2	2	2	2	2	2
1	2	1	2	2	2	2	2	2	2
2	1	2	2	2	2	2	2	2	2
2	2	1	2	2	2	2	2	2	2
1	2	1	1	2	2	2	2	1	2
2	2	1	2	1	2	2	2	2	1
1	2	1	1	2	2	2	2	1	2
2	2	1	2	2	2	2	2	2	1
1	1	1	2	2	2	2	2	2	1
1	2	1	2	2	2	2	2	2	2
1	2	1	2	2	2	2	2	2	1
1	2	2	2	2	2	2	2	2	2
1	2	1	1	2	2	2	2	2	2
2	2	1	1	2	2	2	2	2	1
1	1	1	2	2	2	2	2	1	1
1	1	1	2	2	2	2	2	1	1
1	2	1	2	2	2	2	2	2	2
1	2	2	2	2	2	2	2	2	2
1	2	1	2	2	2	2	2	2	2
2	2	1	2	2	2	2	2	2	2
1	2	1	2	2	2	2	2	2	2
1	2	1	2	2	2	2	2	2	2
2	2	1	2	2	2	2	2	2	2
2	2	2	2	2	2	2	2	1	2
2	2	2	2	2	2	2	2	2	2
2	1	1	2	2	2	2	2	2	2
2	2	2	2	2	2	2	2	2	2
2	2	2	2	2	2	2	2	2	2
2	2	2	2	2	2	2	2	2	2
2	2	1	2	2	2	2	2	2	2
1	2	1	2	2	2	2	2	2	2
2	2	1	2	2	2	2	2	2	1
1	2	2	2	2	2	2	2	2	2
2	2	2	2	2	2	2	2	2	2
2	1	1	2	2	2	2	2	1	1
2	2	1	1	2	2	2	2	1	2
2	2	2	2	2	2	2	2	2	2
2	2	1	2	2	2	2	2	2	1
1	2	1	1	2	2	2	2	1	1
2	2	1	2	2	2	2	2	2	2
2	2	2	2	2	2	2	2	2	2
2	1	1	2	2	2	2	2	2	2
2	2	1	2	2	2	2	2	1	2
1	2	2	2	2	2	2	2	2	2
2	2	1	2	2	2	2	2	2	2

2	2	1	2	2	2	2	2	2	2
2	2	1	2	2	2	2	2	1	2
2	2	1	2	2	2	2	2	2	1
1	2	1	2	2	2	2	2	1	2
2	2	1	1	2	2	2	2	1	2
2	1	2	2	2	2	2	2	2	2
2	2	2	2	2	2	2	2	2	2
2	1	2	2	2	2	2	2	2	2
1	1	1	2	2	2	2	2	1	1
2	1	1	2	2	2	2	2	1	2
2	1	1	2	2	2	2	2	1	1
2	1	2	1	2	2	2	2	1	2
1	1	1	2	2	2	2	2	2	1
2	2	1	2	2	2	2	2	1	2
2	2	1	2	2	2	2	2	2	2
2	2	1	1	2	2	2	2	2	2
1	1	1	2	2	2	2	2	2	1
2	2	1	2	2	2	2	2	2	2
2	2	1	2	2	2	2	2	2	2
2	2	1	2	2	2	2	2	1	2
1	2	2	2	2	2	2	2	2	2
2	2	2	2	2	2	2	2	2	2
2	2	2	2	2	2	2	2	2	2
2	2	2	2	2	2	2	2	2	2
2	2	2	2	2	2	2	2	2	2
2	1	2	2	2	2	2	2	1	1
2	2	1	2	2	2	2	2	1	2
1	2	1	2	2	2	2	2	2	2
2	2	1	2	2	2	2	2	2	2
1	2	2	2	2	2	2	2	2	2
2	2	2	2	2	2	2	2	2	2
2	2	1	2	2	2	2	2	2	2
2	2	2	2	2	2	2	2	2	2
2	2	2	2	2	2	2	2	1	2
1	2	1	1	2	2	2	2	1	1
2	2	2	2	2	2	2	2	2	2
2	2	1	2	2	2	2	2	2	2
2	2	1	1	2	2	2	2	1	2
2	1	1	1	2	2	2	2	1	1
2	2	1	2	2	2	2	2	1	2
1	2	1	2	2	2	2	2	2	1
2	2	1	1	2	1	2	2	1	1
2	2	1	2	2	2	2	2	1	1
2	2	1	2	2	2	2	2	1	1
1	1	1	2	2	2	2	2	2	2
1	2	1	2	2	2	2	2	1	1
1	2	1	2	2	2	2	2	2	2

2	2	2	2	2	2	2	2	2	2
2	2	1	2	2	2	2	2	2	2
1	1	1	2	2	2	2	2	1	2
1	1	1	1	2	2	2	2	1	1
2	2	2	2	2	2	2	2	2	2
2	2	2	2	2	2	2	2	2	2
1	2	1	2	2	2	2	2	2	2
2	2	1	2	2	2	2	2	2	2
2	2	1	2	2	2	2	2	2	2
1	2	1	2	2	2	2	2	1	2
2	1	2	2	2	2	2	2	2	2
2	2	2	2	2	2	2	2	2	2
1	2	1	1	2	2	2	2	1	1
1	2	1	1	2	2	2	2	1	2
2	1	2	2	2	2	2	2	2	2
1	2	2	2	2	2	2	2	2	2
2	2	2	2	2	2	2	2	2	2
2	2	1	2	2	2	2	2	2	2
2	1	1	1	2	2	2	2	2	1
2	2	1	2	2	1	2	2	1	2
2	2	1	2	2	2	2	2	2	2
2	2	1	2	2	2	2	2	1	2
2	2	1	2	2	2	2	2	2	2
2	2	1	2	2	2	2	2	2	2
2	2	1	2	2	2	2	2	2	2
1	2	1	2	2	2	2	2	1	2
2	2	1	2	2	2	2	2	1	2
2	2	1	2	2	2	2	2	2	2
2	2	2	2	2	2	2	2	2	2
2	2	2	2	2	2	2	2	2	2
1	1	2	2	2	2	2	2	2	2
1	2	2	2	2	2	2	2	2	2
2	2	2	2	2	2	2	2	2	2
1	2	2	2	2	2	2	2	2	2
1	2	1	2	2	2	2	2	2	2
2	2	1	2	2	2	2	2	2	2
1	2	1	1	2	2	2	2	2	2
1	2	1	2	2	2	2	2	1	2
2	2	1	2	2	2	2	2	2	2
2	2	1	2	2	2	2	2	2	2
2	2	2	2	2	2	2	2	1	2
2	2	1	2	2	2	2	2	1	2
2	1	1	2	2	2	2	2	1	2
2	2	1	2	2	2	2	2	2	2
2	2	2	2	2	2	2	2	2	2
2	2	1	2	2	2	2	2	2	2
1	2	2	2	2	2	2	2	2	2

2	2	1	2	2	2	2	2	2	2
2	2	1	2	2	2	2	2	2	2
2	2	1	2	2	2	2	2	2	2
2	2	1	2	2	2	2	2	1	1
2	1	1	2	2	2	2	2	1	1
1	2	1	2	2	2	2	2	2	2
2	2	2	2	2	2	2	2	2	2
2	2	1	2	2	2	2	2	2	2
2	2	1	2	2	2	2	2	2	2
2	2	1	1	2	2	2	2	1	2
2	2	1	1	2	2	2	2	1	1
2	2	1	1	2	2	2	2	1	1
2	2	2	2	2	2	2	2	2	2
1	2	1	1	1	2	2	2	1	2
1	1	1	2	2	2	2	2	2	2
1	1	1	2	2	2	2	2	2	2
2	1	1	2	2	2	2	2	2	2
1	1	1	2	2	2	2	2	2	2
2	2	2	2	2	2	2	2	2	2
2	2	1	2	2	2	2	2	2	2
2	2	1	2	2	2	2	2	2	2
2	2	1	2	2	2	2	2	2	2
2	2	1	2	2	2	2	2	2	2
2	2	2	2	2	2	2	2	2	2
1	2	2	2	2	2	2	2	2	2
2	2	2	2	2	2	2	2	2	2
2	2	1	2	2	2	2	2	2	2
1	1	1	2	2	2	2	2	1	1
2	2	1	2	2	2	2	2	1	2
1	2	1	2	2	2	2	2	2	2
2	2	1	1	2	2	2	2	2	2
2	2	1	2	2	2	2	2	2	2
1	2	1	2	2	2	2	2	2	2
1	2	2	2	2	2	2	2	2	2
2	2	1	2	2	2	2	2	2	2
1	2	2	2	2	2	2	2	2	2
2	2	1	2	2	2	2	2	1	2
2	2	1	1	2	2	2	2	2	2
2	1	1	2	2	2	2	2	2	2
2	1	1	2	2	2	2	2	2	2
2	2	1	1	2	2	2	2	2	2
1	2	1	2	2	2	2	2	1	1
2	1	1	2	2	2	2	2	2	2
2	2	1	2	2	2	2	2	1	2
2	2	1	2	2	2	2	2	2	2
2	2	2	2	2	2	2	2	2	2
2	1	1	2	2	2	2	2	2	2

2	2	1	2	2	2	2	2	2	2
1	2	1	1	2	2	2	2	2	2
2	2	1	2	2	2	2	2	1	2
1	2	1	2	2	2	2	2	2	2
1	2	2	2	2	2	2	2	2	2
2	2	1	2	2	2	2	2	2	2
1	2	1	2	2	2	2	2	2	2
2	2	2	2	2	2	2	2	1	2
2	2	1	2	2	2	2	2	1	2
2	1	1	1	2	2	2	2	2	2
2	1	1	2	2	2	2	2	1	2
2	2	1	2	2	2	2	2	1	2
2	2	1	2	2	2	2	2	1	2
2	2	2	2	2	2	2	2	2	2
2	2	1	2	2	2	2	2	2	2
2	2	1	2	2	2	2	2	2	2
2	2	1	2	2	2	2	2	1	2
2	2	2	2	2	2	2	2	2	2
2	2	1	2	2	2	2	2	2	2
2	1	1	2	2	2	2	2	2	1
2	2	1	2	2	2	2	2	1	2
2	1	1	1	2	2	2	2	2	2
2	1	1	1	2	2	2	2	2	2
2	2	1	2	2	2	2	2	2	2
2	2	1	1	2	2	2	2	2	1
2	2	1	2	2	2	2	2	2	2
1	2	2	2	2	2	2	2	2	1
2	2	1	2	2	2	2	2	2	2
1	2	1	2	2	2	2	2	2	2
2	1	1	2	2	2	2	2	1	1
2	2	1	1	2	2	2	2	1	1
2	2	1	2	2	2	2	2	2	2
2	2	1	2	2	2	2	2	1	2
2	2	1	2	2	2	2	2	2	1
2	2	2	2	2	2	2	2	2	2
1	2	1	2	2	2	2	2	2	2
2	2	1	2	2	2	2	2	2	2
1	1	1	2	2	2	2	2	1	2
2	2	1	2	2	2	2	2	2	2
2	1	1	2	2	2	2	2	2	2
2	2	1	1	2	2	2	2	2	1
2	2	1	2	2	2	2	2	2	2
2	1	1	2	2	2	2	2	2	2
2	1	1	2	2	2	2	2	1	2
2	2	1	2	2	2	2	2	2	2
2	2	1	2	2	2	2	2	2	2
2	2	1	2	2	2	2	2	2	2

2	2	1	2	2	2	2	2	2	2
2	2	1	2	2	2	2	2	2	2
1	2	1	2	2	2	2	2	1	1
2	2	1	2	2	2	2	2	2	2
2	2	1	2	2	2	2	2	2	2
2	2	1	2	2	2	2	2	2	2
2	2	1	1	2	2	2	2	2	2
1	2	1	1	2	2	2	2	2	2
2	2	1	1	2	2	2	2	2	2
1	2	1	2	2	2	2	2	2	2
1	1	1	2	2	2	2	2	2	2
2	2	1	2	2	2	2	2	2	2
1	2	1	2	2	2	2	2	2	2
1	2	1	2	2	2	2	2	2	2
2	2	1	2	2	2	2	2	2	2
2	2	1	2	2	2	2	2	2	2
2	2	2	2	2	2	2	2	2	2
2	2	2	2	2	2	2	2	2	2
2	2	1	2	2	2	2	2	2	2
2	1	1	1	2	2	2	2	1	1
2	2	1	2	2	2	2	2	2	2
1	2	1	2	2	2	2	2	2	2
2	1	1	2	2	2	2	2	1	1
2	2	1	2	2	2	2	2	2	2
2	2	1	2	2	2	2	2	2	2
2	2	1	2	2	2	2	2	2	2
2	2	1	2	2	2	2	2	2	2
2	2	1	2	2	1	2	2	1	1
2	2	1	2	2	2	2	2	2	2
1	2	1	2	2	2	2	2	1	2
1	2	1	2	2	2	2	2	2	2
2	2	1	2	2	2	2	2	1	2
1	2	1	2	2	2	2	2	2	2
2	2	1	1	2	2	2	2	1	2
1	2	1	2	2	2	2	2	2	2
2	2	1	2	2	2	2	2	2	2
2	2	1	2	2	2	2	2	2	2
2	2	2	2	2	2	2	2	2	2
2	2	1	2	2	2	2	2	2	2
2	2	1	2	2	2	2	2	1	1
2	2	1	2	2	2	2	2	1	2
1	2	2	2	2	2	2	2	2	2
2	2	1	2	2	2	2	2	1	1
2	2	1	2	2	2	2	2	1	1
1	2	1	1	2	2	2	2	2	2
2	2	1	2	2	2	2	2	1	2
2	2	1	2	2	2	2	2	2	2

1	2	1	1	2	2	2	2	2	2
2	2	1	2	1	2	2	2	2	2
2	2	1	2	2	2	2	2	2	2
1	1	1	2	2	2	2	2	1	1
2	2	1	2	2	2	2	2	2	2
2	2	2	2	2	2	2	2	2	2
2	2	1	2	2	2	2	2	1	2
1	2	1	2	2	2	2	2	1	2
2	2	1	2	2	2	2	2	2	2
2	2	1	2	2	2	2	2	2	2
2	2	2	2	2	2	2	2	1	2
2	2	1	2	2	2	2	2	2	2
2	2	2	2	2	2	2	2	2	2
1	2	1	2	2	2	2	2	1	2
1	1	1	2	2	2	2	2	1	1
2	2	1	2	2	2	2	2	1	2
2	2	1	2	2	2	2	2	1	1
2	2	2	2	2	2	2	2	2	2
2	2	1	2	2	2	2	2	1	2
2	2	2	2	2	2	2	2	1	2
2	2	2	2	2	2	2	2	2	2
2	1	1	1	2	2	2	2	1	1
1	1	1	2	2	2	2	2	1	2
2	1	1	2	2	2	2	2	1	1
2	1	1	1	2	2	2	2	1	1
2	2	1	1	2	2	2	2	2	2
2	1	1	2	2	2	2	2	1	1
2	2	2	2	2	2	2	2	1	2
2	1	1	1	2	2	2	2	2	2
2	2	1	1	2	2	2	2	2	2
2	2	1	2	2	2	2	2	2	1
2	2	2	2	2	2	2	2	2	2
2	2	2	2	2	2	2	2	2	1
2	2	1	2	2	2	2	2	2	2
1	1	1	2	2	2	2	2	2	2
2	2	1	2	2	2	2	2	2	2
2	2	1	2	2	2	2	2	1	1
2	1	1	2	2	2	2	2	1	1
2	1	1	2	2	2	2	2	2	2
1	1	1	2	2	2	2	2	1	1
2	2	1	1	2	2	2	2	1	2
2	2	2	2	2	2	2	2	2	2
2	2	1	2	2	2	2	2	1	1
2	2	1	2	2	2	2	2	2	2
2	2	1	2	2	2	2	2	1	2
2	2	1	2	2	2	2	2	2	2
1	1	1	2	2	2	2	2	1	1

2	2	1	2	2	2	2	2	1	1
1	2	1	1	2	2	2	2	1	1
2	2	1	2	2	2	2	2	2	2
2	2	1	2	2	2	2	2	2	2
2	2	1	2	2	2	2	2	2	2
2	2	1	2	2	2	2	2	2	2
2	2	1	2	2	2	2	2	1	1
2	2	1	2	2	2	2	2	1	1
2	2	1	2	2	2	2	2	2	2
2	2	1	2	2	2	2	2	2	2
2	1	1	2	2	2	2	2	1	1
2	2	1	2	2	2	2	2	2	2
1	2	1	2	2	2	2	2	1	1
2	2	1	2	2	2	2	2	1	1
2	2	1	2	2	2	2	2	1	1
2	2	2	1	2	2	2	2	1	1
2	2	1	2	2	2	2	2	1	1
2	2	1	1	2	2	2	2	1	1
2	2	1	2	2	2	2	2	1	2
1	2	1	1	2	2	2	2	1	1
2	2	1	2	2	2	2	2	2	2
2	1	1	1	2	2	2	2	2	2
2	2	1	2	2	2	2	2	1	1
2	2	1	1	2	2	2	2	2	1
2	2	1	2	2	2	2	2	1	1
2	1	1	2	2	2	2	2	1	1
2	2	1	2	2	2	2	2	1	1
2	2	1	2	2	2	2	2	1	1
1	2	1	2	2	2	2	2	1	1
2	2	1	2	2	2	2	2	1	1
2	2	2	2	2	2	2	2	2	2
1	2	1	1	2	2	2	2	1	1
2	2	1	2	2	2	2	2	2	2
1	2	1	2	2	2	2	2	2	2
1	1	1	1	2	2	2	2	1	1
2	2	1	2	2	2	2	2	2	2
2	2	1	2	2	2	2	2	1	2
2	2	1	2	2	2	2	2	2	2
2	2	1	2	2	2	2	2	2	2
2	2	1	2	2	2	2	2	2	2
2	2	1	2	2	2	2	2	2	2
1	2	1	1	2	2	2	2	1	1
2	2	1	2	2	2	2	2	2	2
2	2	2	2	2	2	2	2	2	2
2	2	2	2	2	2	2	2	2	2
2	2	1	2	2	2	2	2	2	2
2	2	1	2	2	2	2	2	2	2

1	1	1	2	2	1	2	2	1	2
2	2	2	2	2	2	2	2	2	2
2	2	2	2	2	2	2	2	1	2
2	1	1	2	2	2	2	2	1	1
2	1	1	2	2	2	2	2	2	1
2	1	1	2	2	2	2	2	1	1
1	2	1	2	2	2	2	2	2	1
1	2	1	1	2	2	2	2	1	1
2	2	1	1	2	2	2	2	2	2
2	2	1	1	2	2	2	2	2	2
2	2	2	2	2	2	2	2	2	2
1	2	1	1	2	2	2	2	2	2
1	2	1	2	2	2	2	2	1	1
2	2	2	2	2	2	2	2	2	2
1	2	1	2	2	2	2	2	1	1
1	2	1	2	2	2	2	2	2	1
1	1	1	1	2	2	2	2	1	2
1	2	1	2	2	2	2	2	2	2
2	2	1	2	2	2	2	2	1	2
1	1	1	1	2	2	2	2	1	2
1	1	1	1	2	2	2	2	1	2
2	2	1	2	2	2	2	2	1	2
2	2	1	2	2	2	2	2	2	2
2	1	1	1	2	2	2	2	1	1
1	2	1	2	2	2	2	2	1	2
2	2	1	2	2	2	2	2	2	2
2	2	1	2	2	2	2	2	2	2
2	2	1	2	2	2	2	2	2	2
2	2	1	2	2	2	2	2	2	2
1	2	1	2	2	2	2	2	2	2
1	2	1	1	2	2	2	2	1	2
1	1	1	2	2	2	2	2	1	1
2	2	1	2	2	2	2	2	2	2
1	1	1	1	2	2	2	2	1	1
1	2	1	2	2	2	2	2	2	1
1	1	1	2	2	2	2	2	1	1
2	2	1	2	2	2	2	2	2	2
2	2	1	2	2	2	2	2	2	2
2	2	1	2	2	2	2	2	1	2
2	2	1	1	2	2	2	2	1	1
2	2	1	2	2	2	2	2	2	2
2	2	1	2	2	2	2	2	1	2
2	2	1	2	2	2	2	2	2	2
2	2	1	2	2	2	2	2	1	2
2	2	1	1	2	2	2	2	1	1
2	2	1	2	2	2	2	2	1	1
1	1	1	2	2	2	2	2	2	2

2	2	1	2	2	2	2	2	2	2
2	2	1	2	2	2	2	2	1	2
1	2	2	2	2	2	2	2	2	2
1	2	2	2	2	2	2	2	2	2
2	2	1	2	2	2	2	2	2	2
1	1	1	2	2	2	2	2	1	1
2	2	2	2	2	2	2	2	1	1
2	2	1	1	2	2	2	2	1	2
1	2	1	1	2	2	2	2	1	2
2	2	2	2	2	2	2	2	2	1
2	2	1	2	2	2	2	2	2	2
2	2	1	2	2	2	2	2	2	2
1	2	1	2	2	2	2	2	1	1
2	2	1	2	2	2	2	2	1	2
1	1	1	2	2	2	2	2	1	1
1	2	1	1	2	2	2	2	1	2
2	2	2	2	2	2	2	2	2	2
1	2	1	2	2	2	2	2	2	2
1	1	1	2	2	2	2	2	2	2
2	2	2	2	2	2	2	2	1	2
2	2	2	2	2	2	2	2	2	2
2	2	2	2	2	2	2	2	2	2
2	2	1	2	2	2	2	2	1	2
2	2	1	1	2	2	2	2	2	2
2	2	1	2	2	2	2	2	1	1
2	2	1	1	2	2	2	2	2	1
1	2	1	2	2	2	2	2	1	1
1	1	1	2	2	2	2	2	1	2
2	2	1	2	2	2	2	2	1	2
1	2	1	2	2	2	2	2	2	2
2	2	1	2	2	2	2	2	2	2
2	2	1	1	2	1	2	2	1	1
1	2	1	1	2	2	2	2	1	2
2	2	1	2	2	2	2	2	2	2
2	2	1	2	2	2	2	2	2	2
2	2	1	2	2	2	2	2	2	1
2	2	2	2	2	2	2	2	2	2
2	2	1	2	2	2	2	2	1	1
2	2	2	2	2	2	2	2	2	2
2	2	1	2	2	2	2	2	1	2
1	1	1	2	2	2	2	2	1	2
1	1	1	2	2	2	2	2	1	2
2	2	2	2	2	2	2	2	2	2
1	2	1	2	2	2	2	2	2	2
2	1	1	2	2	2	2	2	1	2
1	2	1	2	2	2	2	2	1	1
1	1	1	2	2	2	2	2	1	2

2	2	1	2	2	2	2	2	2	2
2	2	1	2	2	2	2	2	2	2
1	1	1	2	2	2	2	2	1	1
2	2	1	2	2	2	2	2	2	2
2	2	1	2	2	2	2	2	2	2
1	1	1	2	2	2	2	2	2	2
2	2	1	2	2	2	2	2	2	2
2	2	1	2	2	2	2	2	2	2
1	1	1	2	2	2	2	2	1	1
2	2	1	1	2	2	2	2	2	2
2	2	1	2	2	2	2	2	2	2
2	2	1	2	2	2	2	2	2	2
2	2	1	2	2	2	2	2	2	2
2	2	2	2	2	2	2	2	2	2
2	2	1	2	2	2	2	2	1	1
1	2	1	2	2	2	2	2	2	1
1	2	1	2	2	2	2	2	1	1
1	2	1	1	2	2	2	2	1	1
2	2	1	1	1	2	2	2	1	1
2	2	1	1	2	2	2	2	1	1
2	2	1	2	2	2	2	2	2	1
1	2	1	2	2	2	2	2	2	2
2	2	2	2	2	2	2	2	2	2
1	1	1	2	2	2	2	2	1	1
2	2	2	2	2	2	2	2	2	1
2	2	1	2	2	2	2	2	2	2
2	2	2	2	2	2	2	2	2	2
2	2	1	2	2	2	2	2	2	2
2	1	1	2	2	2	2	2	1	2
1	2	2	2	2	2	2	2	2	2
2	2	2	2	2	2	2	2	1	2
1	2	1	2	2	2	2	2	2	2
2	2	1	2	2	2	2	2	2	2
2	2	1	2	2	2	2	2	2	2
2	2	2	2	2	2	2	2	2	2
2	2	1	2	2	2	2	2	1	1
2	2	1	2	2	2	2	2	2	2
1	2	1	1	2	1	2	2	1	1
2	2	1	2	2	2	2	2	2	2
2	2	1	2	2	2	2	2	1	2
2	2	2	2	2	2	2	2	2	2
2	2	1	2	2	2	2	2	2	2
1	1	1	2	2	2	2	2	1	1
2	2	1	2	2	2	2	2	1	2
2	2	1	2	2	2	2	2	2	2
1	2	1	2	2	2	2	2	1	2

head ache	vomiting	diarrhoea	chicken po	worm infe	hepatitis	ear ache	ear dischar	ear inflam	toothache
2	2	2	2	2	2	2	2	2	2
2	2	2	2	2	2	2	2	2	2
2	2	1	2	2	2	2	2	2	2
2	2	2	2	2	2	2	2	2	2
2	2	2	2	2	2	2	2	2	2
2	2	2	2	1	2	2	2	2	2
2	2	2	2	2	2	1	2	2	2
1	2	2	2	2	2	2	2	2	2
2	2	2	2	2	2	2	2	2	2
2	2	2	2	2	2	2	2	2	2
1	2	2	2	1	2	2	2	2	2
2	2	2	2	2	2	2	2	2	2
2	2	2	2	2	2	2	2	2	2
2	2	2	2	2	2	2	2	2	2
2	1	2	2	2	2	2	2	2	2
2	1	1	2	2	2	2	2	2	2
2	1	1	2	2	2	2	2	2	2
2	2	2	2	2	2	2	2	2	2
2	2	2	2	2	2	2	2	2	2
2	1	1	2	2	2	2	2	2	2
2	2	2	2	2	2	2	2	2	2
2	2	2	2	2	2	2	2	2	2
2	2	2	2	2	2	2	2	2	2
2	2	2	2	2	2	2	2	2	2
2	2	2	2	2	2	2	2	2	2
2	2	2	2	2	2	2	2	2	2
2	1	2	2	2	2	2	2	2	2
2	2	2	2	2	2	2	2	2	2
2	2	2	2	2	2	2	2	2	2
2	2	2	2	2	2	2	2	2	2
2	2	1	2	2	2	2	2	2	2
2	2	2	2	2	2	2	2	2	2
2	2	2	2	2	2	2	2	2	2
2	2	1	2	2	2	2	2	2	2
2	2	2	2	2	2	2	2	2	2
1	2	1	2	2	2	1	2	2	2
2	2	2	2	2	2	1	1	2	2
2	2	2	2	2	2	2	2	2	2
2	2	2	2	2	2	2	2	2	2
2	1	1	2	2	2	2	2	2	2
2	2	2	2	2	2	2	2	2	2
2	2	2	2	2	2	2	2	2	2
1	2	1	2	2	2	2	2	2	2
1	2	1	1	2	2	2	2	2	2
2	2	2	2	2	2	2	2	2	2
2	2	2	2	2	2	1	2	2	2

2	2	2	2	2	2	2	2	2	2
2	2	1	2	2	2	2	2	2	2
2	2	2	2	2	2	2	2	2	2
2	2	2	2	2	2	2	2	2	2
1	2	1	1	2	2	1	1	2	2
2	2	2	2	2	2	2	2	2	2
2	2	2	2	2	2	2	2	2	2
2	2	2	2	2	2	2	2	2	2
2	1	2	2	2	2	2	2	2	2
2	2	2	2	2	2	1	2	2	2
1	1	1	2	2	2	1	2	2	2
1	2	2	2	2	2	2	2	2	2
1	1	2	2	2	2	2	2	2	2
2	2	2	2	2	2	2	2	2	2
2	2	2	2	2	2	2	2	2	2
2	2	2	2	2	2	2	2	2	2
1	1	1	2	2	2	2	2	2	2
1	2	1	2	2	2	2	2	2	2
2	2	2	2	2	2	2	2	2	2
2	1	1	2	2	2	2	2	2	2
2	2	2	2	2	2	2	2	2	2
2	2	2	2	2	2	2	2	2	2
2	2	2	2	2	2	2	2	2	2
2	2	2	2	2	2	2	2	2	2
2	2	2	2	2	2	2	2	2	2
2	1	1	2	2	2	2	2	2	2
2	2	2	2	2	2	2	2	2	2
2	2	2	2	2	2	2	2	2	2
2	2	2	2	2	2	2	2	2	2
2	2	2	2	2	2	2	2	2	2
2	2	2	2	2	2	2	2	2	2
2	2	2	2	2	2	1	1	2	2
1	2	1	2	1	2	2	2	2	2
2	1	2	2	2	2	2	2	2	2
1	1	1	2	2	2	2	2	2	2
2	2	2	2	2	2	1	2	2	2
2	2	2	2	2	2	2	2	2	2
1	1	2	2	2	2	2	2	2	2
1	1	1	2	2	2	2	2	2	2
2	2	1	2	2	2	2	2	2	2
2	2	2	2	2	2	2	2	2	2
1	1	1	2	2	2	2	2	2	2
2	2	1	2	2	2	2	2	2	2
2	1	1	2	2	2	2	2	2	2
2	2	2	1	2	2	2	2	2	2
2	1	1	2	2	2	2	2	2	2
2	2	1	2	2	2	2	2	2	2

2	2	2	2	2	2	2	2	2	2
1	2	2	2	2	2	2	2	2	2
2	1	1	2	2	2	2	2	2	2
2	1	1	2	2	2	1	2	2	2
2	2	2	2	2	2	2	2	2	2
2	2	2	2	2	2	2	2	2	2
2	2	2	2	2	2	1	2	2	2
2	2	1	2	2	2	2	2	2	2
2	2	2	2	2	2	2	2	2	2
2	1	1	2	2	2	2	2	2	2
2	2	2	2	2	2	2	2	2	2
2	2	2	2	2	2	1	1	2	2
2	2	2	2	2	2	2	2	2	2
1	1	1	2	2	2	2	2	2	2
2	2	2	2	2	2	2	2	2	2
2	2	2	2	2	2	2	2	2	2
2	2	2	2	2	2	2	2	2	2
2	1	1	2	2	2	2	2	2	2
1	2	2	1	1	2	2	2	2	2
2	2	2	2	2	2	2	2	2	2
2	1	2	2	2	2	2	2	2	1
2	2	1	2	2	2	2	2	2	2
2	1	2	2	2	2	2	2	2	2
2	1	2	2	2	2	2	2	2	2
2	1	2	2	2	2	2	2	2	2
2	2	1	2	2	2	1	2	2	2
2	2	1	2	2	2	2	2	2	2
2	2	2	2	2	2	2	2	2	2
2	2	2	2	1	2	2	2	2	2
2	2	2	2	2	2	2	2	2	2
1	2	2	2	2	2	2	2	2	2
2	2	2	2	2	2	2	2	2	2
2	2	2	2	2	2	2	2	2	2
1	2	1	2	2	2	1	2	2	2
1	2	1	2	2	2	1	2	2	2
1	1	1	2	2	2	1	2	2	2
2	2	2	2	2	2	2	2	2	2
2	1	1	2	2	2	2	2	2	2
1	1	1	1	2	2	2	2	2	2
2	2	2	2	2	2	2	2	2	2
2	1	2	2	2	2	2	2	2	2
1	2	1	2	2	2	1	2	2	2
1	1	1	2	2	2	2	2	2	2
2	2	2	2	2	2	2	2	2	2
2	2	2	2	2	2	2	2	2	2
2	2	2	2	2	2	2	2	2	2
2	2	2	2	2	2	2	2	2	2

2	1	1	2	2	2	2	2	2	2
2	2	1	2	2	2	2	2	2	2
2	2	2	2	2	2	1	2	2	2
1	1	1	2	2	2	1	2	2	2
1	1	1	2	2	2	1	1	1	2
2	2	2	2	2	2	2	2	2	2
2	2	2	2	2	2	2	2	2	2
2	2	2	2	2	2	2	2	2	2
2	1	1	2	2	2	2	2	2	2
1	2	1	2	2	2	2	2	2	2
1	1	1	2	2	2	1	2	2	2
1	1	1	2	2	2	2	2	2	2
2	2	2	2	2	2	2	2	2	2
2	1	2	2	1	2	2	2	2	2
2	2	2	2	2	2	2	2	2	2
2	2	2	2	2	2	2	2	2	2
2	2	2	2	2	2	2	2	2	2
2	2	2	2	2	2	2	2	2	2
2	2	2	2	2	2	2	2	2	2
2	2	2	2	2	2	2	2	2	2
2	2	2	2	2	2	2	2	2	2
2	2	2	2	2	2	2	2	2	2
2	2	2	2	2	2	2	2	2	2
2	2	2	2	2	2	2	2	2	2
2	2	2	2	2	2	1	1	1	2
2	2	2	2	2	2	2	2	2	2
2	2	2	2	2	2	2	2	2	2
1	2	1	2	2	2	2	2	2	2
1	1	1	2	2	2	1	2	2	2
2	2	2	2	2	2	2	2	2	2
1	1	1	2	2	2	2	2	2	2
2	2	2	2	2	2	2	2	2	2
2	2	2	2	2	2	2	2	2	2
2	2	1	2	2	2	2	2	2	2
2	2	2	2	2	2	2	2	2	2
2	2	2	2	2	2	2	2	2	2
2	2	2	2	2	2	2	2	2	2
1	1	2	2	2	2	1	2	2	2
2	2	2	2	2	2	2	2	2	2
2	2	2	2	2	2	2	2	2	2
2	2	1	2	2	2	2	2	2	2
2	1	1	2	1	2	2	2	2	2
2	2	2	2	2	2	2	2	2	2
1	2	1	2	2	2	2	2	2	2
2	2	2	2	2	2	2	2	2	2
2	2	2	2	2	2	2	2	2	2
2	2	1	2	2	2	1	2	2	2

1	2	1	2	2	2	1	2	2	2
1	2	2	2	2	2	2	2	2	2
1	1	1	2	2	2	2	2	2	2
2	2	2	2	2	2	1	1	2	2
2	2	2	2	2	2	2	2	2	2
2	2	1	1	2	2	2	2	2	2
2	2	2	2	2	2	2	2	2	2
2	2	2	2	2	2	2	2	2	2
1	2	1	2	2	2	2	2	2	2
1	2	1	2	2	2	2	2	2	2
2	2	2	2	2	2	2	2	2	2
2	2	2	2	2	2	2	2	2	2
2	2	1	2	1	2	2	2	2	2
2	2	2	2	2	2	2	2	2	2
1	2	2	2	2	2	2	2	2	2
2	2	2	2	2	2	2	2	2	2
1	2	2	2	2	2	2	2	2	2
2	2	1	2	2	2	2	2	2	2
1	2	1	2	2	2	2	2	2	2
2	2	2	2	2	2	2	2	2	2
1	2	1	2	2	2	2	2	2	2
2	1	1	2	2	2	2	2	2	2
2	2	2	1	2	2	2	2	2	2
2	2	2	2	2	2	2	2	2	2
1	1	1	2	2	2	2	2	2	2
2	1	2	2	2	2	2	2	2	2
2	1	2	2	2	2	2	2	2	2
2	1	2	2	2	2	2	2	2	2
2	2	2	2	2	2	2	2	2	2
2	1	1	2	2	2	1	1	1	2
1	1	1	2	2	2	2	2	2	2
2	2	2	2	2	2	2	2	2	2
2	2	1	2	2	2	2	2	2	2
1	2	2	2	2	2	2	2	2	2
2	2	2	2	2	2	2	2	2	2
1	2	2	2	2	2	2	2	2	2
1	1	2	2	2	2	2	2	2	2
1	2	2	2	2	2	1	1	2	2
2	2	2	2	2	2	2	2	2	2
1	1	2	2	2	2	2	2	2	2
1	2	2	2	2	2	2	2	2	2
2	2	2	2	2	2	2	2	2	2
2	1	1	2	2	2	2	2	2	2
1	2	1	2	2	2	1	1	2	2
2	2	2	2	2	2	2	2	2	2
2	2	2	2	2	2	2	2	2	2
2	2	2	2	2	2	1	2	2	2

2	2	1	2	2	2	2	2	2	2
2	2	2	2	2	2	2	2	2	2
2	2	1	2	2	2	2	2	2	2
2	2	2	2	2	2	2	2	2	2
1	2	2	2	2	2	2	2	2	2
2	2	1	2	2	2	2	2	2	2
1	2	1	2	2	2	2	2	2	2
2	2	2	2	2	2	2	2	2	2
1	2	1	2	2	2	2	2	2	2
2	2	2	2	2	2	2	2	2	2
2	2	1	2	2	2	2	2	2	2
2	2	2	2	2	2	1	1	2	2
2	2	1	2	2	2	2	2	2	2
2	2	1	2	2	2	2	2	2	2
2	2	2	2	2	2	2	2	2	2
2	2	2	2	2	2	2	2	2	2
2	2	2	2	2	2	2	2	2	2
2	2	2	2	2	2	2	2	2	2
2	2	2	2	2	2	2	2	2	2
1	1	1	2	1	2	2	2	2	2
2	2	2	2	2	2	2	2	2	2
2	2	2	2	2	2	2	2	2	2
2	2	1	2	2	2	2	2	2	2
2	2	2	2	2	2	2	2	2	2
2	2	2	2	2	2	2	2	2	2
2	2	1	2	1	2	2	2	2	2
2	2	2	2	2	2	2	2	2	2
1	2	2	2	1	2	2	2	2	2
2	2	2	2	2	2	2	2	2	2
2	1	1	2	2	2	2	2	2	2
2	2	2	2	2	2	2	2	2	2
2	2	1	2	2	2	2	2	2	2
2	2	2	2	2	2	2	2	2	2
1	2	2	1	2	2	1	1	2	2
2	2	2	2	2	2	2	2	2	2
2	2	2	2	2	2	2	2	2	2
2	2	2	2	2	2	2	2	2	2
2	2	2	2	2	2	2	2	2	2
2	2	1	2	2	2	2	2	2	2
1	1	1	2	2	2	2	2	2	2
2	2	1	2	2	2	1	1	2	2
2	2	2	2	2	2	2	2	2	2
1	1	1	2	2	2	2	2	2	2
1	1	1	2	2	2	2	2	2	2
1	2	1	2	2	2	1	2	2	2
2	1	1	2	1	2	2	2	2	2
2	2	2	2	2	2	2	2	2	2

1	1	2	1	1	2	2	2	2	2
2	2	2	2	2	2	2	2	2	2
2	2	1	2	2	2	2	2	2	2
2	2	2	2	2	2	2	2	2	2
1	2	2	2	2	2	2	2	2	2
2	1	1	2	2	2	2	2	2	2
1	1	1	2	2	2	2	2	2	2
1	2	2	2	2	2	2	2	2	2
2	2	2	2	2	2	2	2	2	2
2	2	2	2	2	2	2	2	2	2
2	2	2	2	2	2	2	2	2	2
2	2	1	1	2	2	2	2	2	2
2	1	1	2	2	2	2	2	2	2
1	1	2	2	2	2	2	2	2	2
1	1	1	2	2	2	2	2	1	2
2	1	1	2	1	2	1	2	1	2
1	1	1	2	2	2	2	2	2	2
2	2	2	2	2	2	2	2	2	2
1	2	2	2	2	2	2	2	2	2
1	2	2	2	2	2	2	2	2	2
2	2	2	2	2	2	2	2	2	2
1	1	1	2	2	2	2	2	2	2
1	2	2	2	2	2	2	2	2	2
2	2	1	2	2	2	2	2	2	2
1	1	1	2	2	2	2	2	2	2
1	1	1	2	2	2	2	2	2	2
1	1	1	2	2	2	2	2	2	1
2	2	1	2	2	2	2	2	2	2
2	2	2	2	2	2	1	1	2	2
2	2	1	2	2	2	2	2	2	2
1	1	1	2	2	2	2	2	2	2
2	2	2	2	2	2	2	2	2	2
2	2	2	2	2	2	2	2	2	2
2	2	2	2	2	2	2	2	2	2
2	2	1	2	2	2	2	2	2	2
2	2	2	2	2	2	2	2	2	2
1	1	1	2	2	2	2	2	2	2
1	1	1	2	2	2	2	2	2	2
2	2	2	2	2	2	2	2	2	2
1	1	1	1	2	2	2	2	2	2
1	1	1	1	2	2	2	2	2	2
2	2	2	2	2	2	2	2	2	2
1	1	1	2	2	2	2	2	2	2
2	2	1	2	2	2	2	2	2	2
1	1	1	2	2	2	1	1	2	2
2	1	2	2	2	2	2	2	2	2
1	1	1	2	2	2	2	2	2	2

1	1	1	2	2	2	2	2	2	2
1	1	1	2	2	2	2	2	2	2
2	2	2	2	2	2	2	2	2	2
2	2	2	2	2	2	2	2	2	2
2	2	2	2	2	2	2	2	2	2
1	1	1	2	2	2	2	2	2	2
1	1	1	2	2	2	2	2	2	2
1	1	1	2	2	2	2	2	2	2
2	1	2	2	2	2	2	2	2	2
2	2	2	2	2	2	2	2	2	2
2	2	1	2	2	2	2	2	2	2
1	2	2	2	2	2	1	2	2	2
1	1	1	2	2	2	2	2	2	2
1	1	1	2	2	2	2	2	2	2
1	1	1	2	2	2	2	2	2	2
1	1	1	2	2	2	2	2	2	2
1	1	1	2	2	2	2	2	2	2
1	1	1	2	2	2	2	2	2	2
2	2	2	2	2	2	2	2	2	2
1	1	1	2	2	2	2	2	2	2
2	2	2	2	2	2	2	2	2	2
2	2	2	2	2	2	2	2	2	2
1	1	1	2	2	2	2	2	2	2
1	1	1	2	1	2	2	2	2	2
1	1	1	2	2	2	2	2	2	2
1	1	1	2	2	2	2	2	2	2
1	1	1	2	2	2	2	2	2	2
1	1	1	2	2	2	1	2	2	2
1	1	1	2	2	2	2	2	2	2
1	1	1	2	2	2	2	2	2	2
2	2	2	2	2	2	2	2	2	2
2	1	1	1	2	2	2	2	2	2
2	2	2	2	2	2	2	2	2	2
2	2	2	2	2	2	2	2	2	2
2	1	1	2	2	2	1	2	2	2
2	2	2	2	2	2	2	2	2	2
2	2	2	2	2	2	2	2	2	2
2	2	2	2	2	2	2	2	2	2
1	2	2	2	2	2	2	2	2	2
2	2	2	2	2	2	2	2	2	2
2	2	2	2	2	2	2	2	2	2
2	2	1	2	2	2	2	2	2	2
2	2	2	2	2	2	2	2	2	2
1	1	2	2	2	2	2	2	2	2
2	2	2	2	2	2	2	2	2	2
2	2	2	2	2	2	2	2	2	2
1	2	2	2	2	2	2	2	2	2

2	2	2	2	2	2	2	2	2	2
2	2	2	2	2	2	2	2	2	2
1	1	1	2	2	2	2	2	2	2
2	1	1	2	2	2	2	2	2	2
1	1	1	2	2	2	2	2	2	2
1	1	1	2	2	2	2	2	2	2
2	1	1	2	2	2	1	2	2	2
1	1	1	2	2	2	2	2	2	2
1	2	1	2	2	2	2	2	2	2
2	2	2	2	2	2	1	2	2	2
2	2	1	2	2	2	2	2	2	2
1	2	2	2	2	2	2	2	2	2
1	2	1	2	2	2	2	2	2	2
2	2	2	2	2	2	2	2	2	2
1	1	2	2	2	2	1	2	1	2
2	2	2	2	2	2	2	2	2	2
2	1	2	2	2	2	2	2	2	2
2	2	1	2	2	2	2	2	2	2
2	1	1	2	2	2	2	2	2	2
2	1	2	2	2	2	2	2	2	2
1	2	2	2	2	2	2	2	2	2
1	2	1	2	2	2	2	2	2	2
2	2	1	2	2	2	2	2	2	2
1	2	2	2	2	2	2	2	2	2
1	2	1	2	2	2	2	2	2	2
2	2	2	2	2	2	2	2	2	2
1	1	2	2	2	2	2	2	2	2
2	2	2	2	2	2	2	2	2	2
2	2	2	2	2	2	2	2	2	2
2	2	1	2	2	2	2	2	2	2
2	2	2	2	2	2	2	2	2	2
2	2	1	2	2	2	2	2	2	2
2	2	2	2	2	2	2	2	2	2
1	2	2	2	2	2	2	2	2	1
2	1	1	2	2	2	2	2	2	2
1	2	1	2	2	2	2	2	2	2
2	2	2	2	2	2	2	2	2	2
2	2	2	2	2	2	2	2	2	2
1	1	1	2	2	2	2	2	2	2
2	2	2	2	2	2	2	2	2	2
2	2	2	2	2	2	2	2	2	2
1	2	2	2	2	2	2	2	2	2
2	2	1	2	2	2	2	2	2	2
2	2	2	2	2	2	2	2	2	2
1	1	1	2	2	2	2	2	2	2
1	1	1	2	1	2	2	2	2	2
1	2	2	2	2	2	1	1	2	2

2	2	2	2	2	2	2	2	2	2
2	2	2	2	2	2	2	2	2	2
2	2	2	2	2	2	2	2	2	2
2	2	2	2	2	2	2	2	2	2
2	2	2	2	2	2	2	2	2	2
1	1	2	2	2	2	2	2	2	2
2	2	2	2	2	2	2	2	2	2
1	1	1	2	2	2	1	2	2	2
1	1	1	2	2	2	1	2	2	2
2	2	2	2	2	2	2	2	2	2
2	2	2	2	2	2	2	2	2	2
2	2	2	2	2	2	2	2	2	2
1	1	1	2	2	2	2	2	2	2
2	2	2	2	2	2	1	2	2	2
1	1	1	2	2	2	2	2	2	1
1	1	1	2	2	2	1	2	2	2
2	2	2	2	2	2	2	2	2	2
2	2	2	2	2	2	2	2	2	2
2	1	2	2	2	2	2	2	2	2
1	1	1	2	2	2	2	2	2	2
2	2	2	2	2	2	2	2	2	2
2	2	2	2	2	2	2	2	2	2
2	1	1	2	2	2	2	2	2	2
1	2	2	2	2	2	2	2	2	2
2	1	1	2	2	2	2	2	2	2
1	2	2	2	2	2	2	2	2	2
2	1	1	2	1	2	1	2	2	2
2	1	1	2	2	2	2	2	2	2
1	1	1	2	2	2	2	2	2	2
2	2	2	2	2	2	2	2	2	2
2	2	1	2	2	2	2	2	2	2
1	1	1	2	2	2	2	2	2	2
1	1	1	2	2	2	2	2	2	2
2	2	2	2	2	2	2	2	2	2
2	1	1	2	2	2	2	2	2	2
2	2	2	2	2	2	2	2	2	2
2	2	2	2	2	2	2	2	2	2
1	1	1	2	2	2	2	2	2	2
2	2	2	2	2	2	2	1	2	2
1	1	1	2	2	2	2	2	2	2
1	1	1	1	1	2	2	2	2	2
1	1	1	1	1	2	2	2	2	2
2	2	2	2	2	2	2	2	2	2
2	2	2	2	2	2	2	2	2	2
2	2	1	2	2	2	2	2	2	2
1	1	1	2	2	2	2	2	2	2
1	1	1	1	1	2	2	2	2	2

2	2	2	2	2	2	2	2	2	2
2	2	2	2	2	2	2	2	2	2
1	1	1	2	2	2	2	2	2	2
2	2	2	2	2	2	2	2	2	2
2	2	2	2	2	2	2	2	2	2
1	2	1	2	2	2	2	2	2	2
2	2	2	2	2	2	2	2	2	2
2	2	1	2	2	2	2	2	2	2
1	1	1	2	2	2	1	2	2	2
2	1	2	2	2	2	2	2	2	2
2	2	2	2	2	2	2	2	2	2
2	2	2	2	2	2	2	2	2	2
2	2	2	2	2	2	2	2	2	2
2	2	2	2	2	2	2	2	2	2
1	1	1	2	2	2	2	2	2	2
1	2	2	2	2	2	2	2	2	2
1	1	1	2	1	2	2	2	2	2
1	1	1	2	2	2	2	2	2	2
2	1	1	2	2	2	1	2	2	2
1	1	1	2	1	2	2	2	2	2
1	1	2	2	2	2	1	2	2	2
2	2	1	1	2	2	2	2	2	2
1	1	1	1	2	2	2	2	2	2
1	1	1	2	1	2	2	2	2	2
2	2	2	2	2	2	2	2	2	2
2	2	2	2	2	2	2	2	2	2
2	2	2	2	2	2	2	2	2	2
2	2	2	2	2	2	2	2	2	1
1	1	1	2	2	2	2	2	2	2
2	2	2	2	2	2	2	2	2	2
2	2	1	2	2	2	2	2	2	2
2	2	2	2	2	2	2	2	2	2
2	2	2	2	2	2	2	2	2	2
2	2	2	2	2	2	2	2	2	2
2	2	2	2	2	2	2	2	2	2
1	1	1	2	1	2	1	2	2	1
1	1	1	2	2	2	2	2	2	2
1	1	1	2	2	2	2	2	2	1
2	2	2	2	2	2	2	2	2	2
2	2	1	2	2	2	2	2	2	2
2	2	2	2	2	2	2	2	2	2
2	2	2	2	2	2	1	1	2	2
1	2	1	1	1	2	2	2	2	2
2	1	1	2	2	2	1	2	2	2
2	1	2	2	2	2	2	2	2	2
2	2	1	2	2	2	1	1	2	2

gastro	mouth	appendicit	ulcer	piles	wound	allergy	tumor	itchinng	epilepsy
2	2	2	2	2	2	2	2	2	2
2	2	2	2	2	2	2	2	2	2
2	2	2	2	2	2	2	2	2	2
1	2	2	2	2	2	2	2	2	2
2	2	2	2	2	2	2	2	2	2
1	1	2	1	2	1	1	2	1	2
2	2	2	2	2	2	2	2	2	2
2	2	2	2	2	2	1	2	2	2
2	2	2	2	2	2	2	2	2	2
2	2	2	2	2	2	2	2	1	2
2	1	2	2	2	2	2	2	2	2
2	2	2	2	2	2	2	2	2	2
2	2	2	2	2	2	2	2	2	2
2	2	2	2	2	2	2	2	2	2
2	2	2	2	2	2	2	2	2	2
2	2	2	2	2	2	2	2	2	2
2	2	2	2	2	2	2	2	2	2
2	2	2	2	2	2	2	2	2	2
2	2	2	2	2	2	2	2	2	2
2	2	2	2	2	2	2	2	2	2
2	2	2	2	2	2	2	2	2	2
2	2	2	2	2	2	2	2	2	2
2	2	2	2	2	2	2	2	2	2
2	2	2	2	2	2	2	2	2	2
2	2	2	2	2	2	2	2	2	2
2	2	2	2	2	2	2	2	2	2
1	2	2	2	2	2	1	2	2	2
2	2	2	2	2	1	2	2	2	2
2	2	2	2	2	1	2	2	2	2
2	1	2	2	2	1	2	2	2	2
2	2	2	2	2	2	2	2	2	2
2	2	2	2	2	2	2	2	2	2
2	2	2	2	2	2	2	2	2	2
2	2	2	2	2	1	2	2	1	2
2	2	2	2	2	2	2	2	2	2
2	2	2	2	2	2	2	2	2	2
2	2	2	2	2	2	2	2	2	2
2	2	2	2	2	2	2	2	2	2
2	2	2	2	2	2	2	2	2	2
1	1	2	2	2	2	2	2	2	2
2	1	2	2	2	1	2	2	2	2
2	2	2	2	2	2	2	2	2	2
1	2	2	2	2	2	2	2	2	2
2	2	2	2	2	2	2	2	2	2
2	2	2	2	2	2	2	2	2	2
2	2	2	2	2	2	2	2	2	2

2	2	2	2	2	2	2	2	2	2
2	2	2	2	2	2	2	2	2	2
2	2	2	2	2	2	2	2	2	2
2	2	2	2	2	2	2	2	2	2
2	2	2	2	2	2	2	2	2	2
2	2	2	2	2	2	2	2	2	2
2	2	2	2	2	2	2	2	2	2
2	2	2	2	2	2	2	2	2	2
2	1	2	2	2	2	2	2	2	2
1	2	2	2	2	2	2	1	2	2
1	2	2	2	2	2	2	2	2	2
2	1	2	2	2	2	2	2	2	2
1	2	2	2	2	2	2	2	2	2
1	2	2	2	2	2	2	2	2	2
2	2	2	2	2	2	2	2	2	2
1	2	2	2	2	2	2	2	2	2
2	2	2	2	2	1	2	2	2	2
2	2	2	2	2	1	2	2	2	2
2	2	2	2	2	2	2	2	2	2
1	2	2	2	2	1	2	2	2	2
2	2	2	2	2	2	2	2	2	2
2	2	2	2	2	2	2	2	2	2
2	2	2	2	2	2	2	2	2	2
2	2	2	2	2	2	2	2	2	2
2	2	2	2	2	2	2	2	2	2
1	2	2	2	2	2	2	2	2	2
2	2	2	2	2	1	2	2	2	2
2	2	2	2	2	2	2	2	2	2
2	2	2	2	2	2	2	2	2	2
2	2	2	2	2	2	2	2	2	2
2	2	2	2	2	1	2	2	2	2
2	2	2	2	2	2	2	2	2	2
2	2	2	2	2	2	2	2	2	2
2	2	2	2	2	2	2	2	2	2
1	2	2	2	2	1	2	2	1	2
2	2	2	2	2	2	2	2	2	2
2	2	2	2	2	2	2	2	2	2
1	1	2	2	2	2	2	2	2	2
2	1	2	2	2	2	2	2	2	2
2	2	2	2	2	1	2	2	1	2
2	2	2	2	2	2	2	2	2	2
1	2	2	2	2	2	2	2	2	2
1	2	2	2	2	2	2	2	2	2
1	1	2	2	2	2	2	2	2	2
2	1	2	2	2	1	2	2	2	2
2	2	2	2	2	2	2	2	2	2
2	2	2	2	2	2	2	2	2	2

2	2	2	2	2	1	2	2	2	2
2	2	2	2	2	2	2	2	2	2
2	2	2	2	2	2	2	2	2	2
1	1	2	2	2	2	2	2	1	2
2	2	2	2	2	2	1	2	2	2
2	2	2	2	2	2	2	2	2	2
2	2	2	2	2	2	2	2	2	2
2	2	2	2	2	2	2	2	2	2
2	1	2	2	2	2	2	2	2	2
2	2	2	2	2	1	2	1	2	2
2	2	2	2	2	2	2	2	2	2
2	2	2	2	2	1	2	2	2	2
2	1	2	2	2	1	2	2	2	2
1	1	2	2	2	1	2	2	2	2
2	2	2	2	2	2	2	2	2	2
2	2	2	2	2	2	2	2	2	2
2	2	2	2	2	2	2	2	2	2
1	2	2	2	2	2	2	2	2	2
2	2	2	2	2	2	2	2	2	2
2	2	2	2	2	1	1	2	1	2
2	2	2	2	2	2	2	2	2	2
2	1	2	2	2	1	2	2	2	2
2	2	2	2	2	2	2	2	2	2
2	2	2	2	2	2	2	2	2	2
2	2	2	2	2	2	2	2	2	2
2	2	2	2	2	2	2	2	2	2
2	2	2	2	2	2	2	2	2	2
2	2	2	2	2	2	2	2	2	2
2	2	2	2	2	1	2	2	2	2
2	2	2	2	2	2	2	2	2	2
2	2	2	2	2	2	2	2	2	2
2	2	2	2	2	2	2	2	2	2
2	2	2	2	2	2	2	2	2	2
2	2	2	2	2	2	2	2	2	2
2	2	2	2	2	2	2	2	2	2
2	2	2	2	2	2	2	2	2	2
2	2	2	2	2	2	2	2	2	2
2	2	2	2	2	2	2	2	2	2
2	2	2	2	2	2	2	2	2	2
1	2	2	2	2	2	2	2	2	2
1	2	2	2	2	2	2	2	2	2
2	2	2	2	2	2	2	2	2	2
1	1	2	2	2	2	2	2	2	2
2	2	2	2	2	2	2	2	2	2
2	2	2	2	2	2	2	2	2	2
2	2	2	2	2	2	2	2	2	2
2	2	2	2	2	2	2	2	2	2

2	2	2	2	2	2	2	2	2	2
2	2	2	2	2	1	2	2	2	2
2	2	2	2	2	2	2	2	2	2
1	2	2	2	2	2	2	2	2	2
1	1	2	2	2	1	1	2	1	2
2	2	2	2	2	2	2	2	2	2
2	2	2	2	2	2	2	2	2	2
2	2	2	2	2	2	2	2	2	2
2	2	2	2	2	2	2	2	2	2
2	2	2	2	2	2	2	2	2	2
1	1	2	2	2	1	2	2	2	2
1	1	2	2	2	2	2	2	2	2
2	2	2	2	2	2	2	2	2	2
2	2	2	2	2	2	2	2	2	2
2	2	2	2	2	2	2	2	2	2
2	2	2	2	2	2	1	2	2	2
2	2	2	2	2	2	2	2	2	2
2	2	2	2	2	2	2	2	2	2
2	2	2	2	2	2	2	2	2	2
2	2	2	2	2	2	2	2	2	2
2	2	2	2	2	2	2	2	2	2
2	2	2	2	2	2	2	2	2	2
2	2	2	2	2	2	2	2	2	2
2	2	2	2	2	2	2	2	2	2
2	2	2	2	2	1	2	2	2	2
2	2	2	2	2	2	1	2	2	2
2	2	2	2	2	1	2	2	2	2
1	1	2	2	2	2	1	2	2	2
1	2	2	1	2	1	2	2	1	2
2	2	2	2	2	2	2	2	2	2
2	1	2	2	2	2	2	2	2	2
2	2	2	2	2	2	2	2	2	2
2	2	2	2	2	2	2	2	2	2
2	2	2	2	2	2	2	2	2	2
2	2	2	2	2	2	2	2	2	2
2	2	2	2	2	2	2	2	2	2
2	2	2	2	2	2	2	2	2	2
1	1	2	2	2	1	2	2	2	2
2	2	2	2	2	2	2	2	2	2
2	2	2	2	2	2	2	2	1	2
2	1	2	2	2	2	2	2	2	2
1	2	2	2	2	1	1	2	1	2
2	2	2	2	2	2	2	2	2	2
1	2	2	2	2	2	2	2	2	2
2	2	2	2	2	2	2	2	2	2
2	2	2	2	2	2	2	2	2	2
2	2	2	2	2	2	2	2	2	2

2	2	2	2	2	2	2	2	2	2
2	2	2	2	2	2	2	2	2	2
2	2	2	2	2	2	2	2	2	2
2	2	2	2	2	2	2	2	2	2
2	2	2	2	2	2	2	2	2	2
2	2	2	2	2	2	2	2	2	2
2	2	2	2	2	2	2	2	2	2
2	2	2	2	2	2	2	2	2	2
2	2	2	2	2	2	2	2	2	2
2	2	2	2	2	1	2	2	2	2
2	2	2	2	2	2	2	2	2	2
1	2	2	2	2	2	2	2	2	2
2	2	2	2	2	1	2	2	2	2
2	2	2	2	2	1	2	2	2	2
2	2	2	2	2	2	2	2	2	2
2	2	2	2	2	2	2	2	2	2
1	2	2	2	2	2	2	2	1	2
2	2	2	2	2	1	1	2	1	2
2	2	2	2	2	2	2	2	2	2
2	2	2	2	2	2	2	2	2	2
2	2	2	2	2	2	2	2	2	2
2	2	1	2	2	2	2	2	2	2
1	1	2	2	2	2	2	2	2	2
2	2	2	2	2	1	2	2	2	2
1	1	2	2	2	1	2	2	2	2
2	2	2	2	2	2	2	2	2	2
2	2	2	2	2	1	2	2	1	2
2	2	2	2	2	2	2	2	2	2
2	2	2	2	2	2	2	2	2	2
1	1	2	2	2	1	2	2	2	2
1	1	2	2	2	1	1	2	2	2
2	2	2	2	2	1	2	2	2	2
2	2	2	2	2	2	1	2	2	2
2	1	2	2	2	2	2	2	2	2
2	2	2	2	2	1	2	2	2	2
2	2	2	2	2	2	2	2	2	2
2	2	2	2	2	2	2	2	2	2
1	2	2	2	2	2	2	2	2	2
2	2	2	2	2	2	2	2	2	2
2	2	2	2	2	2	2	2	2	2
2	1	2	2	2	2	2	2	2	2
2	2	2	2	2	2	2	2	2	2
1	1	2	2	2	2	2	2	1	2
1	1	2	2	2	2	2	2	1	2
2	2	2	2	2	2	2	2	2	2
2	2	2	2	2	2	2	2	2	2
2	2	2	2	2	2	2	2	2	2

2	2	2	2	2	2	2	2	2	2
2	2	2	2	2	2	2	2	2	2
2	2	2	2	2	2	2	2	2	2
2	2	2	2	2	2	2	2	2	2
2	2	2	2	2	2	2	2	2	2
2	2	2	2	2	2	2	2	2	2
2	2	2	2	2	2	2	2	2	2
2	2	2	2	2	2	2	2	2	2
2	2	2	2	2	2	2	2	2	2
2	2	2	2	2	2	2	2	2	2
2	2	2	2	2	2	1	2	2	2
2	2	2	2	2	2	2	2	2	2
2	2	2	2	2	2	2	2	2	2
2	2	2	2	2	1	2	2	2	2
2	2	2	2	2	2	2	2	2	2
2	2	2	2	2	2	2	2	2	2
2	2	2	2	2	2	2	2	2	2
2	2	2	2	2	2	2	2	2	2
2	2	2	2	2	2	2	2	2	2
1	2	2	2	2	1	2	2	1	2
2	2	2	2	2	2	2	2	2	2
2	2	2	2	2	2	2	2	2	2
1	2	2	2	2	2	2	2	1	2
2	2	2	2	2	2	2	2	2	2
2	2	2	2	2	2	2	2	2	2
2	2	2	2	2	2	2	2	2	2
2	2	2	2	2	2	2	2	2	2
2	2	2	2	2	2	2	2	2	2
2	2	2	2	2	1	2	2	2	2
2	2	2	2	2	2	2	2	2	2
2	2	2	2	2	2	2	2	2	2
2	2	2	2	2	2	2	2	2	2
2	2	2	2	2	2	1	2	1	2
2	2	2	2	2	2	1	2	2	2
2	2	2	2	2	2	2	2	2	2
2	2	2	2	2	2	2	2	2	2
2	2	2	2	2	2	2	2	2	2
2	2	2	2	2	1	2	2	1	2
2	2	2	2	2	2	2	2	2	2
2	2	2	2	2	1	2	2	2	2
2	2	2	2	2	2	2	2	2	2
2	2	2	2	2	2	2	2	2	2
1	2	2	2	2	1	2	2	2	2
1	2	2	2	2	1	2	2	2	2
2	2	2	2	2	2	2	2	2	2
1	2	2	2	2	2	2	2	1	2
2	2	2	2	2	2	2	2	2	2

2	1	2	2	2	2	1	2	2	2
2	2	2	2	2	2	2	2	2	2
2	2	2	2	2	2	2	2	2	2
1	2	2	2	2	2	1	2	2	2
2	2	2	2	2	2	2	2	2	2
2	2	2	2	2	2	2	2	2	2
2	2	2	2	2	2	2	2	2	2
2	2	2	2	2	2	2	2	2	2
2	2	2	2	2	2	2	2	2	2
2	2	2	2	2	2	2	2	2	2
2	2	2	2	2	1	2	2	2	2
2	2	2	2	2	2	2	2	2	2
2	2	2	2	2	2	2	2	2	2
2	2	2	2	2	2	2	2	2	2
1	2	2	2	2	1	2	2	2	2
2	2	2	2	2	1	2	2	1	2
1	2	2	2	2	2	2	2	2	2
2	2	2	2	2	2	2	2	2	2
2	2	2	2	2	2	2	2	2	2
2	2	2	2	2	2	2	2	2	2
2	2	2	2	2	2	2	2	2	2
2	2	2	2	2	2	2	2	2	2
2	2	2	2	2	1	2	2	1	2
2	2	2	2	2	1	2	2	2	2
2	2	2	2	2	2	2	2	2	2
1	1	2	2	2	1	2	2	2	2
2	2	2	2	2	2	2	2	2	2
2	2	2	2	2	2	2	2	2	2
2	2	2	2	2	2	2	2	2	2
1	2	2	2	2	1	1	2	2	2
2	2	2	2	2	2	2	2	2	2
2	2	2	2	2	2	2	2	2	2
2	2	2	2	2	2	2	2	2	2
2	2	2	2	2	2	2	2	2	2
2	2	2	2	2	2	2	2	2	2
2	2	2	2	2	2	2	2	2	2
2	1	2	2	2	2	2	2	2	2
2	2	2	2	2	1	1	2	2	2
2	2	2	2	2	2	2	2	2	2
1	2	2	2	2	1	2	2	1	2
2	2	2	2	2	2	2	2	2	2
2	2	2	2	2	2	2	2	2	2
1	2	2	2	2	2	2	2	2	2
2	2	2	2	2	2	2	2	2	2
2	2	2	2	2	2	2	2	2	2
2	2	2	2	2	2	2	2	2	2
1	2	2	2	2	1	2	2	2	2

1	2	2	2	2	2	2	2	2	2
2	2	2	2	2	2	2	2	2	2
2	2	2	2	2	2	2	2	2	2
2	2	2	2	2	2	2	2	2	2
2	2	2	2	2	2	2	2	2	2
2	2	2	2	2	2	2	2	2	2
1	2	2	2	2	2	2	2	2	2
1	2	2	2	2	2	2	2	2	2
2	2	2	2	2	2	2	2	2	2
2	2	2	2	2	2	2	2	2	2
2	2	2	2	2	2	2	2	2	2
2	2	2	2	2	2	2	2	2	2
1	2	2	2	2	2	2	2	2	2
1	2	2	2	2	2	2	2	2	2
1	2	2	2	2	2	2	2	2	2
1	2	2	2	2	2	2	2	2	2
1	2	2	2	2	2	2	2	2	2
1	2	2	2	2	2	2	2	2	2
2	2	2	2	2	2	2	2	2	2
2	2	2	2	2	2	2	2	2	2
2	2	2	2	2	2	2	2	2	2
2	1	2	2	2	2	2	2	2	2
1	2	2	2	2	2	2	2	2	2
1	1	2	2	2	2	2	2	2	2
1	2	2	2	2	2	2	2	2	2
1	2	2	2	2	2	2	2	2	2
1	2	2	2	2	2	2	2	2	2
1	2	2	2	2	2	2	2	2	2
1	2	2	2	2	2	2	2	2	2
1	2	2	2	2	2	2	2	2	2
2	2	2	2	2	2	2	2	2	2
1	2	2	2	2	2	2	2	2	2
2	2	2	2	2	2	2	2	2	2
2	2	2	2	2	2	2	2	2	2
2	1	2	2	2	2	1	2	1	2
2	2	2	2	2	2	2	2	2	2
2	2	2	2	2	2	2	2	2	2
2	2	2	2	2	2	2	2	2	2
2	2	2	2	2	2	2	2	2	2
2	2	2	2	2	2	2	2	2	2
2	2	2	2	2	2	2	2	2	2
1	1	2	2	2	1	2	2	2	2
2	2	2	2	2	2	2	2	2	2
1	2	2	2	2	2	2	2	2	2
2	2	2	2	2	2	2	2	2	2
2	2	2	2	2	2	2	2	2	2
2	2	2	2	2	2	2	2	2	2

2	2	2	2	2	1	2	2	2	2
2	2	2	2	2	2	2	2	2	2
2	2	2	2	2	2	2	2	2	2
2	2	2	2	2	2	2	2	2	2
1	2	2	2	2	2	2	2	2	2
2	2	2	2	2	2	2	2	2	2
1	2	2	2	2	2	2	2	2	2
1	1	2	2	2	1	1	2	2	2
2	2	2	2	2	2	2	2	2	2
2	2	2	2	2	2	2	2	2	2
1	2	2	2	2	2	2	2	2	2
2	2	2	2	2	2	2	2	2	2
1	2	2	2	2	2	2	2	2	2
2	2	2	2	2	2	2	2	2	2
1	2	2	2	2	2	2	2	2	2
2	2	2	2	2	2	2	2	2	2
1	2	2	2	2	2	2	2	2	2
2	2	2	2	2	2	2	2	2	2
1	1	2	2	2	2	2	2	2	2
1	2	2	2	2	2	2	2	2	2
2	1	2	2	2	2	2	2	2	2
2	2	2	2	2	2	2	2	2	2
2	2	2	2	2	2	2	2	2	2
1	2	2	2	2	2	2	2	2	2
2	2	2	2	2	2	2	2	2	2
2	2	2	2	2	1	2	2	2	2
1	2	2	2	2	2	2	2	2	2
2	2	2	2	2	2	2	2	2	2
1	2	2	2	2	1	2	2	2	2
1	2	2	2	2	1	2	2	2	2
1	1	2	2	2	1	2	2	1	2
1	2	2	2	2	2	2	2	2	2
2	2	2	2	2	2	2	2	2	2
1	1	2	2	2	1	2	2	1	2
2	2	2	2	2	2	2	2	2	2
2	2	2	2	2	2	2	2	2	2
2	2	2	2	2	1	2	2	2	2
2	2	2	2	2	1	2	2	2	2
1	2	2	2	2	2	2	2	2	2
2	2	2	2	2	2	1	2	2	2
2	2	2	2	2	2	2	2	2	2
2	1	2	2	2	2	1	2	2	2
2	2	2	2	2	1	2	2	2	2
2	2	2	2	2	2	2	2	2	2
1	2	2	2	2	1	2	2	1	2
1	2	2	2	2	2	2	2	2	2
2	2	2	2	2	2	2	2	2	2

2	2	2	2	2	2	2	2	2	2
2	2	2	2	2	2	2	2	2	2
2	2	2	2	2	2	2	2	2	2
2	2	2	2	2	2	2	2	2	2
2	2	2	2	2	2	2	2	2	2
1	2	2	2	2	1	2	2	2	2
2	2	2	2	2	2	2	2	2	2
1	1	2	1	2	1	1	2	1	2
1	1	2	1	2	1	1	2	1	2
2	2	2	2	2	2	2	2	2	2
2	2	2	2	2	2	2	2	2	2
2	2	2	2	2	2	2	2	2	2
1	1	2	2	2	1	1	2	2	2
2	2	2	2	2	2	2	2	2	2
1	2	2	2	2	2	2	2	2	2
2	2	2	2	2	2	2	2	2	2
2	2	2	2	2	2	2	2	2	2
2	2	2	2	2	2	2	2	2	2
2	2	2	2	2	1	1	2	1	2
2	1	2	2	2	2	2	2	2	2
2	2	2	2	2	2	2	2	2	2
2	2	2	2	2	2	2	2	2	2
2	2	2	2	2	2	2	2	2	2
2	2	2	2	2	1	2	2	2	2
1	2	2	2	2	2	2	2	2	2
2	2	2	2	2	2	2	2	2	2
1	2	2	2	2	2	2	2	2	2
1	2	2	2	2	2	1	2	2	2
2	2	2	2	2	2	2	2	2	2
2	2	2	2	2	2	2	2	2	2
2	2	2	2	2	2	2	2	2	2
1	1	2	2	2	1	1	2	2	2
1	1	2	1	2	2	2	2	2	2
2	2	2	2	2	2	2	2	2	2
1	2	2	2	2	2	2	2	2	2
2	2	2	2	2	1	2	2	2	2
2	2	2	2	2	2	2	2	2	2
1	2	2	2	2	2	2	2	2	2
2	2	2	2	2	2	2	2	2	2
2	1	2	2	2	2	2	2	2	2
1	1	2	2	2	2	2	2	2	2
1	1	2	2	2	2	2	2	2	2
2	2	2	2	2	2	2	2	2	2
2	2	2	2	2	2	2	2	2	2
2	2	2	2	2	2	2	2	2	2
2	1	2	2	2	2	2	2	2	2
1	2	2	2	2	2	2	2	2	2

2	2	2	2	2	2	2	2	2	2
2	2	2	2	2	2	2	2	2	2
1	1	2	2	2	1	1	2	1	2
2	2	2	2	2	2	2	2	2	2
2	2	2	2	2	2	2	2	2	2
2	2	2	2	2	1	2	2	2	2
2	2	2	2	2	2	2	2	2	2
2	2	2	2	2	2	2	2	2	2
2	2	2	2	2	2	2	2	2	2
2	1	2	2	2	2	2	2	2	2
2	2	2	2	2	2	2	2	2	2
2	2	2	2	2	2	2	2	2	2
2	2	2	2	2	2	2	2	2	2
2	2	2	2	2	2	2	2	2	2
1	2	2	2	2	2	2	2	2	2
2	2	2	2	2	2	2	2	2	2
1	1	2	2	2	2	1	2	2	2
1	1	2	2	2	2	2	2	2	2
1	1	2	2	2	1	2	2	2	2
1	1	2	2	2	2	2	2	2	2
1	1	2	2	2	1	1	2	2	2
2	2	2	2	2	2	2	2	2	2
2	1	2	2	2	2	2	2	2	2
1	2	2	2	2	1	1	2	2	2
2	2	2	2	2	2	2	2	2	2
2	2	2	2	2	2	2	2	2	2
2	2	2	2	2	2	2	2	2	2
2	2	2	2	2	2	2	2	2	2
1	2	2	2	2	2	2	2	2	2
2	2	2	2	2	2	2	2	2	2
2	2	2	2	2	2	1	2	2	2
2	2	2	2	2	2	2	2	2	2
2	2	2	2	2	2	2	2	2	2
2	2	2	2	2	2	2	2	2	2
2	2	2	2	2	2	2	2	2	2
1	1	2	2	2	2	2	2	2	2
2	2	2	2	2	1	2	2	2	2
1	2	2	2	2	2	1	2	2	2
2	2	2	2	2	2	2	2	2	2
2	2	2	2	2	2	2	2	2	2
2	2	2	2	2	2	2	2	2	2
2	1	2	2	2	1	2	2	2	2
2	2	2	2	2	2	2	2	2	2
2	2	2	2	2	2	1	2	2	2
2	2	2	2	2	2	2	2	2	2
2	2	2	2	2	1	1	2	1	2

malaria	ZINC(10.7-	IRON (9-21	TRANSFER	% SATURA	FERRITIN (	Age (m)	Sex	Weight (kg	Height (cm
2						52,14	2	16,5	105
2						52,14	2	16,3	105,4
2						50,33	2	17	102,8
2						43,27	1	11,8	95,8
2						55,43	2	15,9	104
2						48,03	2	16,2	102
2						60,91	2	21,1	111,7
2		14	38,7	18,1	11,6	45,8	2	17	100
2	32,5	13,8	29	23,8	10,4	56,54	2	17	105
2	16,7	11,7	44,1	13,3	11,8	48,99	2	17,5	102
2	14,3	19,6	37,9	25,9	8,6	47,7	1	15,8	102,9
2						36,96	1	12,7	94,4
2	10,2	17	33,8	25,1	9,9	44,68	1	16	97,8
2		23,9	38	31,4	11,1	36,99	1	15	96,3
2						46,92	2	15,6	104,1
2	17,4	20,5	31,2	32,9	10,8	38,64	2	11	88
2	14,4	14,6	37,8	19,3	11,5	38,64	2	15,1	96,5
2						39,69	1	15	95,9
2	13	17,6	35,6	24,7	11,8	52,93	2	18	103,5
2						35,06	2	17	102,1
2						36,14	1	13	94
2						36,83	2	17	105
2						49,45	2	16	100,5
2						49,74	2	17	108,6
2	24,3	5,5	38,7	7,5	7,7	34,4	2	15	93
2						48,33	1	15	101
2						49,94	2	14	102,7
2						61,93	2	15	99
2						50,6	2	18	104
2	17,4	55	35,5	77,5	10,1	48,85	1	16	96,3
2						56,9	2	15	100,8
2	11,2	16,6	37,1	22,4	9,9	50,07	1	15,5	104,6
2	15,2	14,6	32,7	22,3	9,9	49,77	1	18	99
2	17,4	10,5	41,9	12,5	11,8	51,29	1	19,5	99,5
2						37,75	1	12	91,5
2						53,09	1	14	99
2						41,56	1	15	97
2	15,6	10,5	29,3	17,9	11,9	47,74	2	14	96
2						43,4	2	14	98,2
2						50,79	2	17	105,1
2						52,4	1	15,5	101
2						43,76	1	15	97
2						40,15	2	15	95
2	24,7	13,6	34,1	19,9	26,1	57,36	1	17,5	105,2
2						54,44	1	16	103,3
2						52,83	2	14	96,6

2						46,19	2	13	95
2	14,1	10,5	41	12,8	11,9	43,01	2	15	102,3
2						48,49	2	16,5	103,5
2	13,4	17,2	35,5	24,2	10,5	53,03	2	18	110,5
2	25,8	20	25,3	39,5	8,5	55,26	2	15,5	107,3
2						38,77	1	13	92,2
2	17,7	8,9	35,4	12,6	9,2	46,62	1	14	100,5
2						37,91	2	13	96,6
2						41,3	1	15	101,2
2	12,9	14,2	35	20,3	10,1	37,62	2	10	90,5
2						35,12	1	13	92
2	12,6	18,8	43,5	21,6	11,5	49,54	1	16	99,5
2						38,6	2	15	95
2						62,62	1	19,6	109
2		19	36,6	26	10,8	56,38	1	15	98,2
2	11,1	11,1	30,2	18,4	11,8	48,56	1	14,3	96,8
2						42,71	1	12,7	94
2						45,01	2	13,9	96,6
2	13,7	15	33,3	22,5	10,5	56,84	1	16,6	103
2	20,8	7	42,8	8,2	11,3	38,24	2	13,9	92
2	12,2	17	33,6	25,3	10,3	54,74	2	18,1	106,7
2						55,49	1	17,7	107,8
2						41,79	1	15,9	95,6
2	11,8	9	37,7	11,9	10,9	38,57	2	12,2	94
2						45,86	1	17,1	105,2
2						41,92	2	15,3	103,1
2						54,44	1	17,7	109,4
2	20,9	14,7	35,9	20,5	11,8	42,38	1	15,3	97,8
2						43,47	2	14,7	97,3
2						44,29	1	15	95
2						54,9	2	15,6	102,3
2						37,26	2	11,7	82,8
2						51,71	2	13,5	97,6
2						47,44	1	17,1	106,3
1						41	1	15,9	98,5
2						56,9	1	18,6	108,1
2						37,78	1	17,4	98,5
2						32,56	2	11,7	88
2	63,8	15,5	36,4	21,3	11,7	49,02	1	15,5	102
2						43,86	1	13	92
2	11	18	32	28,1	10,7	45,54	2	15,6	98,7
2	10,7	18,2	32,3	28,2	11,5	55,49	2	16	101,2
2						36,53	2	12	90,5
2						55,46	2	15	99
2						52,04	1	15	100
2	33,4	7	52,5	6,7	10,3	47,41	2	14	94
2		15,7	36	21,8	10,9	40,25	1	16,4	102,7

2						34,79	2	13,7	94,2
2	16,1	14,9	37,8	19,7	8,5	50,04	2	16	99,6
2		19,3	33,1	29,2	10,2	47,8	2	16,4	102,5
2		13,4	42,6	15,7	11,2	56,87	1	18,1	104,8
2		16,2	37,6	21,5	10,4	51,42	1	19,8	112,7
2						48,2	2	15,7	78
2	21,1	11,5	31,3	18,4	11,1	41,95	2	13,4	92,9
2	29,6	14,2	40,9	17,4	11,4	42,61	1	15,6	97,8
2		18,6	38,4	24,2	8,3	57,63	1	15	105
2	12,8	10,5	36,2	14,5	9,2	43,3	1	14	96
2	14,8	5,2	40,7	6,4	10,8	43,14	2	12	88
2						48,99	2	17	105
2						39,23	1	11	91
2						54,87	2	18	108,2
2						48,46	2	17	101
2						44,06	1	12	94,5
2						38,05	1	15	99
2	17,4	13,3	31,5	21,1	11,1	56,31	1	18	110,5
2						56,44	1	11	101
2	12,7	12	39,7	15,1	10,5	55,66	2	12	105,5
2	23,2	20,7	37,4	27,7	11,6	51,58	1	18	109,4
2						52,44	1	18	106,5
1						54,01	2	16	104,6
1						47,15	1	15	102,4
1						39,49	2	14	92,5
2						49,12	1	11	93,4
2						51,88	2	16	98
2						46,59	1	16,5	108,2
2	10,9	11,6	32,1	18,1	11,8	42,84	2	15,5	101
2	10,8	9,4	29,4	16	10,4	56,21	2	18	108,9
2						48,43	1	16	98,7
2	16,3	11,6	37,3	15,5	8,9	44,45	2	14	97,5
2						50	1	13	100,6
2						35,61	2	11	88,7
2	129,6	5,7	31,1	9,2	11	40,51	1	14	90,5
2	35,3	10,7	35,5	15,1	10,7	56,21	1	13	99
2						39,03	2	15	94,3
2	20	9,5	40	11,9	9, 9	45,57	2	14	102,6
2						48,95	1	12	94
2	16,1	5,9	34,8	8,5	11,3	50,76	1	17	108
2						55,52	2	16	102,3
2						47,67	1	15	102,4
2						53,16	2	18,5	107,2
2	12,3	9,6	39,8	12,1	10,7	55,85	1	15	88
2	16,6	11,1	43,7	12,7	11,6	51,32	2	15	99,8
2						47,34	1	15	94,2
2						51,32	2	12	97

2	38,5	19,4	33,3	29,1	11,4	57,03	1	17	103
2	39,1	13,5	38,7	17,4	10,1	55,98	2	15	104,6
2						56,18	1	14	102,8
2	17,1	5,1	42	6,1	11,6	37,52	1	15	91,5
2						46,98	1	12	89,7
2						41,1	2	15	94,5
2						48	1	15	100,5
2						56,38	1	20	104
2						40,64	2	13	92,5
2						39,59	2	14	94,5
2						57,92	1	16	106
2						55,26	1	20,5	107,2
2						50,66	1	16	105,1
2	13,8	9,5	46,5	10,2	11,3	43,99	2	14	98
2						56,87	2	17	107,8
2	26,6	15	31,9	23,5	10,9	46,23	2	15	103,2
2						57,89	1	17	103,4
2		18,4	33,8	27,2	10,2	37,75	1	18	88
2						51,65	2	14	103,4
2	24,9	12,7	29,1	21,8	9,9	57,1	2	14	100
2	23,9	29,1	39,1	37,2	11,1	44,81	1	16	97,6
2						57,46	1	14	101
2						36,17	2	13	94,6
2						37,55	1	15	93,9
2						54,21	2	15	101,8
2						53,59	1	13	99,5
2						35,68	2	14	90,8
2						52,83	2	16	102,2
2						54,28	2	13	96,7
2	17,9	16,5	40,1	20,6	10,9	52,6	2	13	96
2	14,7	14,5	37,1	19,5	11,5	43,66	1	13	96,9
2	11,8	12,9	33,1	19,5	9,7	56,8	2	16	106,5
2	22,4	15,1	36,4	20,7	10,3	58,02	2	15	101
2						41,13	1	15	97,6
2	27,3	9,2	45,5	10,1	11,3	36,63	1	11	90,5
2		8,1	43,4	9,3	11,8	40,02	2	15	93,2
2						41,13	2	14	94
2						42,94	1	12	90
2						54,31	1	14	95,4
2						47,67	1	15	97,3
2						43,2	2	13	96
2						51,55	2	19	105,5
2						44,75	1	14	92
2						50,14	1	12	92
2	20,6	11,9	37,4	15,9	10,9	40,71	1	12	96,5
2						43,47	1	15	96
2						48,85	1	19	108

2		11,9	38,6	15,4	11,4	49,87	2	15	95,6
2						49,97	1	17	103,6
2						48,85	1	13	94
2						48,95	1	15	101
2						46,75	1	15	97,4
2	25,7	22,2	35,3	31,4	11,6	36,17	2	15	106
2	17,6	30,6	33,2	46,1	10,5	55	2	15	105,7
2						48,92	2	12	99,5
2						55,75	1	18	102
2						49,15	2	14	99
2						50,4	2	14	93,5
2						52,93	1	14	102
2						48,43	1	14	97,4
2						54,87	2	16	101,3
2						39,43	2	13	95,4
2	108,6	13	43,2	15	8,5	38,21	2	11	92
2	40,4	14,5	30,1	24,1	10,1	55,62	1	15	103,5
2						43,24	2	17	102,6
2	11,3	12,1	32,5	18,6	11,2	45,83	1	12,5	86,4
2						40,57	1	12	94
2						57,92	2	16	101,3
2	15,2	10,9	33,7	16,2	10,9	56,34	2	17	109
2						38,97	1	11	92
2						48,72	2	14	96,5
2						51,42	1	13	100,2
2						56,11	2	22	114
2						57,95	1	16	106,5
2	43,2	15,6	39,2	19,9	11,6	56,08	1	13	100
2	18,5	23,8	39,4	30,2	11,4	42,28	2	17	96,7
2	19,5	13,7	41,2	16,6	11,2	46,36	1	11	92,2
2	21,1	17,4	32,4	26,9	11,7	51,94	1	14	102
2						47,67	1	13	98,5
2						41,17	2	13	95
2						43,14	1	14	96
2						55,85	2	17	103,5
2						49,94	1	16	110
2	16,8	14,9	32,8	22,7	11,9	39,06	1	12	91,8
2						45,5	1	13	96
2						35,12	1	12	92,5
2						37,16	1	12	94,3
2						43,56	1	13	93,4
2	20,5	12,6	40,9	15,4	10,9	47,8	1	16	98,4
2						41,26	2	14	92,5
2	19,3	14	40,9	17,1	9,2	35,42	2	10	86,2
2	18,7	17,8	35,8	24,9	8,6	46,82	1	12,5	95,6
2	20,8	9,5	39,8	11,9	10,7	43,07	2	15	93
2						39,92	1	13	95,6

2	21,4	11,9	33,2	17,9	9,1	53,98	2	12,5	97,3
2						48,46	1	14	95
2						36,44	2	14	95
2						48,2	2	13	95,5
2						49,81	2	17	100
2						31,61	2	10	88,3
2						47,8	2	13	98
2						34,79	2	14	93,5
2	22	13,6	35,6	19,1	11,9	45,37	2	16	100
2						42,41	2	12	94
2						49,45	1	16	98
2						50,23	1	19	104
2						47,05	1	14	95,4
2						58,02	2	15	102
2						48,43	2	16	101,5
2						37,22	1	11	89,5
2						52,24	1	16	100,9
2						52,24	2	14	99
2	16,6	13,4	28,9	23,2	10,5	55,2	2	15	103,5
2	17,3	6,7	35,4	9,5	9,4	56,48	2	20	110
2						37,95	2	16	98,7
2	39,7	15,8	34	23,2	7,7	47,11	2	14	99
2	28,1	12,1	35,8	16,9	11,8	53,98	1	17	103,5
2	13,6	12,4	36,6	16,9	10,6	45,04	2	19	99,8
2	30,3	12,6	35,9	17,5	8,7	45,04	2	19	99
2						48,62	1	15	99
2						44,45	1	15	96,5
2	13,5	9	31	14,5	9,1	52,67	2	15	101
2	12,2	17,5	36,4	24	9,4	50,3	2	17	98
2	19,6	9,9	35,8	13,8	11,4	48,49	1	14	99
2						44,25	1	14	95
2						38,37	2	14	96,5
2						44,25	1	16	100
2						53,32	2	19	110,5
2						50,56	1	17	106
2						49,28	2	15	100,5
2						44,12	1	17	106,5
2						57,95	2	16	103,5
2	12,8	17,5	35	25	10,7	58,25	1	17	109,2
2	12,3	8	30,6	13,1	9,4	53,75	1	17	109
2	13,1	10,5	41	12,8	7,4	47,87	2	15	98,5
2						44,85	1	14	103,5
2						59,07	2	17,3	107,4
2						59,07	2	17,3	109,5
2						57,26	2	17,4	107,5
2						50,2	1	12,55	101
2						62,36	2	17,4	107,9

2						54,97	2	17,3	106
2						67,84	2	22,6	117,5
2						63,47	2	17,4	109,5
2						55,92	2	18,9	106,1
2	13,3	19,8	35,9	27,6	59,1	54,64	1	15,95	107,2
2						43,89	1	13,95	98,4
2	15,2	6,5	34,3	9,5	84,7	51,61	1	15,65	103,1
2						43,93	1	16,2	101,6
2						45,57	2	12,55	92
2						45,57	2	16,2	102
2						46,62	1	15,75	101,5
2	15	7	33,5	10,4	45,5	59,86	2	18,9	107,4
2						41,99	2	18,35	106,6
2						43,76	2	14,9	97,8
2						56,38	2	17,65	104,4
2						56,67	2	19,95	103,5
2	20,9	13,7	43,4	15,8	26,1	41,33	2	14,8	97
2						55,26	1	15,75	104,8
2						50,33	2	15,5	105
2						56,84	2	15,9	106,4
2						68,83	2	15,9	103,3
2						57,49	2	17,85	108
2	17,3	7,3	37,8	9,7	39,9	55,75	1	17,45	101
2						63,8	2	16,25	102,5
2	12,5	13,6	37,3	18,2	37	56,97	1	15,85	109,7
2	12,5	12,3	33,9	18,1	58,3	56,67	1	18,35	102,3
2	14,4	13,8	38,6	17,9	64,8	58,18	1	15,85	103,7
2						44,65	1	12,45	95,7
2						48,46	1	17	109,3
2						50,3	2	14,45	102,7
2						57,69	2	18,3	109,5
2						59,3	1	16,45	105,4
2						47,05	2	16	99,6
2	15,1	7,7	34,9	11	27,9	64,26	1	18,8	109,5
2						61,34	1	17,25	107,7
2						49,91	2	15,05	100,6
2						53,09	2	14,2	99,1
2	15	9	39,3	11,5	17,4	59,73	2	16,9	106,5
2						55,39	2	17,45	109
2	21,8	20,1	34,7	29	40,3	59,93	2	19,5	115,2
2						62,16	2	18,75	111
2	16,6	19,5	40,7	24	45,2	53,52	1	14,6	103,5
2						44,81	2	15,5	101,5
2						48,2	1	16,9	106,2
2						42,02	1	14,2	97
2						63,21	1	16,65	102,5
2	21,5	7,9	37,7	10,5	42,5	69,45	1	20,6	112,9

2						55,39	1	15,65	105
2						49,54	1	13,25	98,6
2						51,84	2	14,4	103,1
2						63,67	1	18,3	107,2
2	14,2	4	39,3	5,1	12,4	45,08	2	14,1	95,7
2	14,4	6	30,7	9,8	89,6	61,57	2	18,1	110,5
2						62,32	1	18,45	110
2						48,62	1	17,15	100
2	23,2	7,1	36,2	9,8	69,7	40,48	2	13	98,1
2						52,7	1	19,1	109,6
2						48,76	2	16,55	108
2						61,27	1	18,05	113,7
2						49,22	1	15,95	102,7
2						50,3	2	15,65	100,4
2						51,12	1	15,05	99,5
2						61,73	2	16,55	107
2						58,55	2	13,8	101
2						44,09	2	12,85	87
2						54,28	1	18,1	110,5
2	12,9	7,7	34,1	11,3	46,2	47,84	1	17,5	103
2						63,74	1	20	112,5
2						44,62	1	18,6	104
2						39,39	2	12,85	92
2						55,85	1	16,6	106
2						50,73	1	13,3	96
2	33,9	14,7	33,8	21,7	68,7	52,37	2	17,2	102,7
2						62,32	2	17,15	107,6
2						43,37	2	12,85	96,1
2						54,24	2	13,3	97,9
2						58,87	1	16,2	103,2
2						42,78	2	16,55	100,8
2						46,85	1	17,8	108
2	15	18,2	35,9	25,3	53,1	56,64	2	17,05	103,5
2	15,7	13,6	33,6	20,2	27,3	54,41	2	16,45	106,2
2	10,8	7,1	41,4	8,6	39,1	63,47	1	18,3	108,6
2	16,5	24	37,2	32,3	54,2	58,02	1	22,5	117
2						54,8	2	16,3	93
2	10,8	11,1	32,9	16,9	49,9	48,56	2	14,2	98,5
2						49,22	1	16,3	101
2	17,4	11,6	36,1	16,1	71,8	64,23	1	17,5	109
2	14,2	6,7	40,1	8,4	13,9	49,54	2	13,05	91,6
2						45,57	1	11,85	94,5
2						54,87	2	17,6	104,4
2						50,46	1	13,2	97,8
2						44,45	1	14,6	103
2	13,9	16,2	33,6	24,1	33,1	62,72	1	19,8	113,5
2						62,85	1	17,9	102,6

2	15,4	16	36,9	21,7	13	62,06	2	17,8	109,4
2	16,3	14,8	34,1	21,7	34,8	57,99	1	18,4	114
2						58,84	1	18,55	110,6
2						60,42	2	17,6	109,5
2						53,55	1	16	107
2						45,9	2	14,2	97,7
2						55,52	1	12,5	98,2
2						58,28	2	17,35	100,5
2						52,99	1	17,6	111,4
2	10,7	19	34,6	27,5	32,8	49,25	2	16,75	104,1
2						62,62	2	20,1	112,3
2						54,83	1	17,35	101,9
2						50,86	2	13,45	107
2						56,41	1	15,25	103,5
2						46,92	1	15,15	93,6
2	23,2	12,7	36,9	17,2	25,3	62,62	1	14,6	103,8
2	13,8	13,3	37,5	17,7	31,9	51,98	2	15,15	107,5
2						55,36	1	13,2	96,4
2	25,3	11,2	36	15,6	47,4	57,17	1	18,55	113
2						61,93	2	17,1	105,5
2						54,28	1	16,7	106,7
2						59,76	2	18,85	109
2	15,4	4,8	36,6	6,6	31,8	62,46	1	18,6	114
2	14,2	8,4	43,7	9,6	12,5	57,92	2	16,35	103
2						53,95	1	17,45	99
2						57,92	2	13,3	101
2	14,7	13,3	33	20,2	77,9	63,64	1	16,7	106,1
2	17,5	15,9	33,5	23,7	20,9	62,59	2	16,7	108,1
2						62,78	1	16,25	105,9
2	16,6	6,7	38,3	8,7	13,6	44,12	1	15,85	95,2
2						54,6	1	13,85	94,8
2						47,7	2	16,7	99,9
2						54,6	1	16,7	104,3
2						62,98	1	21,3	108,7
2						47,24	2	13,55	96,5
2						46,19	2	16	99,1
2						64,53	1	18,8	109,6
2						61,86	1	22,6	112,1
2						50,6	2	16,15	102,6
2	15,9	6,2	43,6	7,1	15,3	57,26	2	19	109,5
2						63,47	2	20,75	112,7
2	14,7	14,9	30,2	24,7	22,6	52,83	2	17,35	107
2						64,49	1	17,35	105,8
2						44,35	1	18,85	92,3
2						57,86	2	16,95	106
2	14	4,9	28,3	8,7	44,2	63,31	2	15,05	102
2	13	18,3	38,3	23,9	27,2	51,02	1	16,35	101,7

2						63,67	1	14,1	106
2						42,38	2	15,4	99
2						43,79	1	17,05	97
2						60,45	2	16,15	104,6
2						59,83	1	15	104
2						41,89	2	14,75	94,2
2						59,04	2	16,95	104,8
2						60,48	2	15,29	101
2	13	4,1	31,3	6,5	101	58,81	2	15,29	99
2	16,8	8,6	36,1	11,9	169	49,87	1	14,1	101
2	11,4	15	35,7	21	61,5	63,01	2	17,5	110
2	15,4	11,4	37,8	15,1	75,5	64,26	2	16,6	104,7
2						47,38	1	16,9	102
2	29	4,1	44,4	4,6	22,1	42,84	1	12,95	94,6
2	10,3	4,6	40,5	5,7	17,1	46,23	2	15,3	97
2						49,18	1	12,75	93,5
2	32,1	7,4	42,3	8,7	41,4	60,52	1	14,75	98,3
2						65,87	1	16,1	101,5
2						57,76	2	19,45	109,5
2						56,38	1	13,1	96,6
2	13,9	23,3	38	30,7	36,8	46,92	1	12,85	101,3
2						49,71	1	16,6	97,7
2						55,06	1	20,55	112,5
2						56,08	2	15,65	99,5
2						56,18	1	17,55	107,1
2						55,1	1	13,6	97
2						55,33	1	14,65	105,5
2						41,99	2	16,15	104,9
2	25,7	22,2	35,3	31,4	33,6	52,96	1	15,85	100,5
2	15,1	16,4	33	24,8	102	61,21	1	16,25	110
2						55,16	2	13,5	102,3
2						61,96	1	15,85	104,6
2						59,14	1	15,3	105
2						54,64	1	14,35	99,6
2						61,04	2	17,6	103,6
2						45,6	2	14,7	100,4
2						44,42	1	12,45	96,5
2						61,8	1	16,4	106,3
2						52,04	1	14,3	100
2						52,14	2	17,45	106,5
2						62,55	2	18,45	112,5
2						45,17	1	12,85	94,6
2						54,9	2	15,25	100,3
2						57,63	1	14,35	103,3
2						62,32	2	24,5	117,7
2	15	13,9	38,9	17,9	32,1	64,13	1	18,3	108,5
2	13,9	11,3	36,8	15,4	31,2	62,29	1	14,55	102,5

2	16,8	16,9	39	21,7	135	48,49		2	18,55	101
2						52,53		1	12,99	96
2						58,12		1	16,25	106
2						53,88		1	15,35	101,7
2						49,35		1	15,25	100
2						62,06		2	18,5	107,6
2						56,11		1	16,65	110,5
2	11,4	2,1	34,3	3,1	87,8	45,24		1	14	95
2						51,71		1	14,3	99,5
2						41,33		1	14,4	96,5
2						43,37		1	13,6	98,5
2	20,5	12,6	40,9	15,4	23,7	53,98		1	17,15	104,5
2						47,44		2	14,55	99,4
2	16,9	13,6	36	18,9	54,9	41,59		2	11,6	90,5
2	12,2	19,6	34	28,8	56,3	53,03		1	13,95	100,6
2	18,7	11,5	36,7	15,7	34,3	49,28		2	14,7	96
2						60,16		2	13,9	100
2	21,4	11,9	33,2	17,9	30,1	46,09		1	13,9	100,5
2						54,41		2	13,85	99
2						49,15		1	15,5	97,4
2						56,02		2	17,05	103
2	13,6	2,9	50,2	2,9	9,3	41		2	14,7	96,4
1						48,59		2	14,2	98,3
2						55,79		1	15,8	101,7
2						56,57		1	20	108,5
2						53,39		1	14,8	99,4
2						64,36		2	17	105
2						54,77		2	16,85	104,2
2	13,8	15,3	29,6	15,3	53	61,54		2	15,8	105,5
2						62,82		2	21,25	113,5
2						44,29		2	17,05	103,7
2	14,3	8,7	34,8	12,5	31,5	53,45		2	14,35	103,5
2	14,3	9,3	37,6	12,4	89,4	60,32		1	17,6	108
2	13,9	8,8	37	11,9	25,4	51,38		2	19,3	104
2	15,9	8,8	32,7	13,5	73,1	51,38		2	18,3	104
2						54,97		1	16,05	102,9
2						56,64		2	17,5	101,5
2						50,79		1	15,6	100,3
2	15,7	11,8	37,8	15,6	51,7	44,71		2	17,95	99,5
2						54,83		1	15,1	104,2
2						59,66		2	20,15	113,4
2						50,6		1	17	104
2						56,9		1	18,25	110
2	12,8	17,5	35	25	36,7	52,6		1	18,15	114,3
2	13,1	8,5	30,4	14	57,3	60,09		1	16,9	112,4
2	14,2	7,2	41,8	8,6	23,8	54,21		2	15,8	102

WHZ	HAZ	WAZ	BAZ	MUAC (cm	MUACZ	Dewormin	Health Cor	Type of He	RtHB
-0,24	-0,15	-0,25	-0,24	15,7	-0,43	1	2	0	1
-0,48	-0,05	-0,34	-0,48	15	-0,98	1	2	0	1
0,59	-0,43	0,12	0,6	16,5	0,21	2	1	1	1
-2,05	-0,98	-1,96	-2,06	13,5	-2	1	2	0	1
-0,47	-0,77	-0,77	-0,42	16	-0,27	1	1	1	1
0,2	-0,32	-0,07	0,19	15,4	-0,57	2	1	1,2, 3	2
1,08	0,27	0,96	1,19	16,5	-0,03	2	1	1	2
1,21	-0,51	0,49	1,21	16	-0,05	1	1	1	1
0,11	-0,68	-0,33	0,15	16,4	-0,01	1	1	1	1
1,11	-0,44	0,45	1,11	18	1,27	2	1	1	1
-0,2	0,08	-0,1	-0,25	15,3	-0,67	1	2	0	1
-0,89	-0,34	-0,78	-0,91	15	-0,54	1	2	0	1
0,98	-0,7	0,25	0,98	15,6	-0,35	1	2	0	1
0,58	0,15	0,51	0,58	17	0,94	2	2	0	1
-0,72	0,33	-0,28	-0,79	16,5	0,29	2	1	1	1
-1,53	-2,56	-2,44	-1,13	14,5	-1,11	1	1	1	1
0,56	-0,33	0,17	0,54	16,2	0,29	1	2	0	1
0,66	-0,41	0,23	0,69	15,4	-0,33	2	2	0	1
1,1	-0,59	0,36	1,12	17	0,5	1	2	0	1
0,75	1,81	1,49	0,53	16,8	0,85	2	2	0	1
-0,54	-0,3	-0,51	-0,54	15	-0,51	1	1	1	1
0,11	2,24	1,31	-0,13	17	0,94	1	1	1	1
0,38	-0,86	-0,28	0,41	16,5	0,23	1	2	0	1
-0,68	1,01	0,17	-0,73	15	-0,93	2	1	1	1
1,21	-0,56	0,53	1,28	16	0,26	1	2	0	1
-0,37	-0,45	-0,53	-0,41	15	-0,92	1	2	0	1
-1,74	-0,4	-1,37	-1,76	15,2	-0,77	1	1	1	1
	-2,55	-1,72	0,03	14		2	2	0	1
0,99	-0,18	0,54	1	16,5	0,2	2	2	0	1
1,28	-1,6	-0,1	1,29	16	-0,2	2	2	0	1
-0,46	-1,65	-1,31	-0,36	15,3	-0,84	2	1	1	1
-0,78	0,15	-0,43	-0,81	14,6	-1,28	2	1	1	1
1,98	-1,09	0,66	1,9	18	1,08	2	1	1	1
2,7	-1,16	1,08	2,54	15,2	-0,86	2	2	0	1
-0,93	-1,22	-1,31	-0,83	13,4	-1,93	2	2	0	1
-0,73	-1,48	-1,4	-0,7	15	-1,06	2	1	1	1
0,44	-0,44	0,05	0,45	16	0,05	2	1	1	1
-0,27	-1,72	-1,21	-0,12	16,1	-0,02	2	2	0	1
-0,74	-0,62	-0,86	-0,75	14,5	-1,22	2	1	1	1
0,09	0,05	0,09	0,08	15,5	-0,55	1	1	1	1
-0,01	-0,96	-0,6	-0,04	15,6	-0,6	1	2	0	1
0,44	-0,76	-0,15	0,46	16	-0,03	1	2	0	1
0,79	-0,96	-0,02	0,87	16	0,09	2	2	0	1
0,41	-0,6	-0,1	0,36	16,1	-0,41	2	2	0	1
-0,15	-0,69	-0,52	-0,18	15,6	-0,67	2	2	0	1
-0,4	-0,96	-0,83	-0,34	14,6	-1,12	1	1	4	1

-0,99	-1,77	-1,68	-0,8	15,3	-0,61	1	1	1	1
-0,8	-0,85	-1,04	-0,76	15,5	-0,6	2	1	1	1
0,09	-0,02	0,03	0,06	15,2	-0,74	1	1	1	1
-0,43	1	0,35	-0,42	14,5	-1,42	1	1	1	1
-1,5	-0,01	-0,95	-1,51	16,4	0,02	1	2	0	1
-0,16	-1,2	-0,78	-0,05	15,3	-0,37	1	2	0	1
-1,04	-0,33	-0,91	-1,09	14	-1,67	1	2	0	1
-1,33	-0,19	-0,99	-1,4	15,5	-0,25	2	2	0	1
-0,42	0,65	0,08	-0,52	16,3	0,28	1	2	0	1
-3,4	-1,75	-3,14	-3,14	13	-2,49	2	1	1	1
-0,12	-0,65	-0,4	-0,04	15,9	0,23	1	1	1	1
0,64	-0,95	-0,15	0,61	17	0,45	1	2	0	1
0,79	-0,72	0,12	0,85	15,5	-0,26	2	1	1	1
	-0,3	0,38	0,78	16,5		1	2	0	1
0,2	-2,01	-1,12	0,2	15	-1,16	1	1	1	1
-0,04	-1,45	-0,9	0	14,7	-1,16	2	2	0	1
-0,81	-1,34	-1,33	-0,72	14,3	-1,3	1	2	0	1
-0,49	-1,23	-1,05	-0,4	15	-0,83	2	2	0	1
0,31	-1,02	-0,43	0,26	16,4	-0,19	2	2	0	1
0,5	-1,45	-0,47	0,69	15,5	-0,25	2	2	0	1
0,46	-0,08	0,27	0,49	16,4	0,03	2	2	0	1
-0,04	0,17	0,11	-0,02	15,6	-0,7	1	2	0	1
1,35	-0,82	0,46	1,4	16	0,04	2	2	0	1
-1,59	-0,98	-1,58	-1,49	14,4	-1,19	2	2	0	1
0,16	0,89	0,62	0,13	16,5	0,25	1	2	0	1
-0,73	0,83	-0,02	-0,88	15	-0,76	2	2	0	1
-0,4	0,65	0,18	-0,33	15	-1,1	2	2	0	1
0,5	-0,36	0,13	0,49	15,2	-0,58	1	1	1	1
0,06	-0,85	-0,47	0,09	15,4	-0,47	2	1	1	1
0,84	-1,32	-0,19	0,91	16,5	0,31	2	2	0	1
-0,32	-1,09	-0,88	-0,26	15,1	-0,96	2	2	0	1
0,58	-3,75	-1,81	1,14	14,4	-1,16	2	1	1	1
-1,07	-1,8	-1,78	-0,91	14,5	-1,39	1	2	0	1
-0,08	0,91	0,49	-0,09	16,2	-0,01	1	2	0	1
0,78	0,02	0,54	0,75	16,6	0,5	2	1	1	1
0,42	0,07	0,35	0,43	16,5	-0,13	1	2	0	1
1,73	0,57	1,52	1,72	16,8	0,77	2	1	1	1
-0,74	-1,63	-1,36	-0,5	14	-1,39	1	1	1	1
-0,22	-0,31	-0,34	-0,26	17	0,47	2	2	0	1
-0,12	-1,98	-1,24	0,05	15,5	-0,4	2	1	1	1
0,48	-0,79	-0,17	0,5	17	0,69	2	1	1	1
0,23	-1,41	-0,72	0,29	15,6	-0,58	1	2	0	1
-1,01	-1,59	-1,53	-0,78	14,6	-0,96	2	2	0	1
-0,07	-1,9	-1,22	0,05	15,2	-0,89	2	2	0	1
-0,16	-1,14	-0,81	-0,18	16,5	0,03	2	2	0	1
0,16	-2,16	-1,18	0,39	17,5	0,98	2	2	0	1
0,25	1,2	0,84	0,16	15,2	-0,5	1	1	1	1

-0,15	-0,3	-0,26	-0,16	15,5	-0,16	1	2	0	1
0,58	-1,14	-0,33	0,63	15,5	-0,54	2	2	0	1
0,23	-0,17	0,04	0,21	17	0,63	1	2	0	1
0,85	-0,64	0,16	0,78	17,5	0,51	1	1	1	1
0,06	1,8	1,17	0,23	16,6	0,12	1	2	0	1
5,15	-6,05	-0,33	6,5	17,3	0,82	2	2	0	1
-0,15	-1,75	-1,1	0,07	15,4	-0,43	2	2	0	1
0,71	-0,4	0,25	0,71	15,5	-0,36	2	2	0	1
-1,24	-0,68	-1,21	-1,23	17	0,17	2	2	0	1
-0,12	-0,94	-0,62	-0,08	17	0,69	2	2	0	1
-0,42	-3,13	-2,1	0,06	16,5	0,39	2	2	0	1
0,11	0,26	0,23	0,08	17	0,6	2	2	0	1
-1,84	-1,58	-2,17	-1,74	14,5	-1,03	2	2	0	1
0,07	0,24	0,22	0,1	16,5	0,1	1	2	0	1
0,99	-0,61	0,27	1	18	1,28	2	2	0	1
-1,57	-1,41	-1,89	-1,5	14	-1,59	1	2	0	1
0,04	0,65	0,4	-0,04	17	0,9	1	2	0	1
-0,46	0,66	0,17	-0,36	18	0,83	2	2	0	1
-3,92	-1,41	-3,44	-3,86	17,3	0,4	1	1	1	1
-4,19	-0,46	-2,98	-4,27	15,9	-0,35	1	1	1	1
-0,22	1,03	0,51	-0,15	17	0,38	1	1	1	1
0,43	0,27	0,45	0,42	15,5	-0,68	1	2	0	1
-0,52	-0,47	-0,62	-0,5	16,9	0,4	1	2	0	1
-0,67	0,04	-0,43	-0,73	17	0,54	1	2	0	1
0,48	-1,51	-0,52	0,67	17	0,85	1	2	0	1
-2,37	-2,3	-2,99	-2,18	14,5	-1,33	2	2	0	1
0,93	-1,73	-0,46	1,01	18	1,19	1	1	1	1
-0,89	1,48	0,31	-0,9	17,5	0,89	2	1	1	1
-0,11	0,16	0,01	-0,19	17,5	1,11	1	2	0	1
-0,08	0,23	0,12	-0,04	17,4	0,69	1	2	0	1
0,8	-0,99	-0,07	0,78	16,9	0,43	1	2	0	1
-0,59	-0,94	-0,95	-0,55	16,1	0,06	1	2	0	1
-1,92	-0,75	-1,74	-1,94	15,1	-0,89	2	2	0	1
-1,7	-1,93	-2,18	-1,42	14,5	-1,03	2	1	5	1
1,01	-1,89	-0,37	1,21	16,5	0,45	1	2	0	1
-1,58	-1,82	-2,18	-1,51	15	-1,16	1	1	1	1
0,94	-0,97	0,08	1,04	17,5	1,22	1	2	0	1
-1,72	0,16	-1,04	-1,82	14,8	-1,01	1	2	0	1
-1,46	-2,14	-2,28	-1,3	15	-0,94	1	2	0	1
-0,52	0,83	0,18	-0,49	16,4	0,01	1	2	0	1
-0,02	-1,16	-0,72	0,04	17	0,44	1	2	0	1
-0,67	-0,03	-0,48	-0,72	16,3	0,05	2	1	1	1
0,6	0,23	0,55	0,63	17,5	0,83	2	2	0	1
2,28	-4,17	-1,09	2,32	16	-0,43	2	2	0	1
-0,24	-1,25	-0,93	-0,18	16,5	0,19	1	2	0	1
1	-1,9	-0,45	1,09	16,5	0,2	1	2	0	1
-2,46	-1,9	-2,7	-2,25	15	-0,96	1	2	0	1

0,57	-1,04	-0,28	0,5	15,5	-0,82	1	1	1	1
-1,31	-0,7	-1,25	-1,27	15	-1,06	2	2	0	1
-1,54	-0,99	-1,62	-1,53	16,5	-0,11	2	1	1	1
1,55	-1,19	0,45	1,71	18	1,58	1	1	1	1
-0,53	-2,91	-2,13	-0,26	15,5	-0,5	2	1	1	1
0,9	-1,22	-0,11	1,02	18	1,5	2	2	0	1
-0,27	-0,52	-0,5	-0,3	16	-0,17	1	2	0	1
2,05	-0,75	0,87	1,89	19	1,41	2	2	0	1
-0,45	-1,67	-1,23	-0,23	14,9	-0,81	2	2	0	1
0,05	-1	-0,53	0,14	15,5	-0,29	1	2	0	1
-0,74	-0,5	-0,76	-0,73	17	0,16	1	1	1	1
1,62	0,07	1,11	1,56	19,2	1,56	1	1	1	1
-0,54	0,19	-0,24	-0,56	17,7	0,86	2	2	0	1
-0,7	-0,75	-0,91	-0,68	15	-0,81	2	1	1	1
-0,51	-0,1	-0,36	-0,47	16,5	0,06	1	1	1	1
-1	0,21	-0,54	-1,08	15,1	-0,77	2	2	0	1
0,48	-1,05	-0,34	0,42	16,3	-0,29	1	2	0	1
4,27	-2,12	1,76	4,55	17	0,91	2	2	0	1
-1,9	-0,45	-1,49	-1,91	15	-0,97	2	2	0	1
-1,14	-1,85	-1,86	-1	14,8	-1,25	1	2	0	1
1,02	-0,77	0,24	1,02	16,3	0,15	2	1	1	1
-1,15	-1,52	-1,7	-1,13	15	-1,19	2	2	0	1
-0,9	-0,43	-0,83	-0,9	15,1	-0,53	2	2	0	1
1,06	-0,57	0,45	1,15	18	1,58	2	2	0	1
-0,69	-1,13	-1,13	-0,62	15,5	-0,63	2	2	0	1
-1,69	-1,43	-2	-1,65	15,2	-0,93	2	2	0	1
0,84	-1,37	-0,17	1,05	17	0,97	1	1	1	1
0,01	-0,87	-0,53	0,04	16,7	0,29	1	2	0	1
-1,35	-2,29	-2,26	-1,12	15,7	-0,47	1	1	1	1
-1,2	-2,27	-2,14	-0,96	14,9	-1,07	1	1	5	1
-1,14	-0,77	-1,23	-1,15	15	-0,77	2	1	1	1
-0,94	-0,38	-0,81	-0,91	16	-0,3	1	2	0	1
-0,51	-1,73	-1,38	-0,4	15,5	-0,71	1	2	0	1
0,32	-0,22	0,09	0,31	16	0,07	2	2	0	1
-1,72	-1,3	-1,92	-1,65	15,6	-0,06	2	2	0	1
1,17	-1,4	-0,01	1,34	16,6	0,55	1	2	0	1
0,16	-1,36	-0,67	0,3	15,5	-0,33	2	2	0	1
-0,59	-2,35	-1,8	-0,37	15,5	-0,37	2	2	0	1
0,01	-2,41	-1,49	0,09	17	0,28	2	2	0	1
0,38	-1,22	-0,48	0,4	16	-0,16	1	2	0	1
-1,2	-1,14	-1,45	-1,11	16	0,01	2	2	0	1
1,28	0,04	0,88	1,29	18,4	1,45	1	2	0	1
0,7	-2,1	-0,75	0,86	15	-0,81	2	2	0	1
-1,04	-2,73	-2,37	-0,8	14,2	-1,6	2	1	1,6	1
-1,99	-0,43	-1,6	-2,09	15,5	-0,29	1	1	1	1
0,64	-0,96	-0,12	0,69	16,5	0,34	2	2	0	1
0,67	1,1	1,1	0,7	17	0,48	1	2	0	1

0,67	-2,06	-0,82	0,83	15,9	-0,22	2	1	1	1
0,44	-0,07	0,24	0,4	17	0,44	1	1	1	1
-0,54	-2,13	-1,65	-0,4	15,2	-0,78	2	1	1	1
-0,37	-0,53	-0,58	-0,4	15,5	-0,57	2	2	0	1
0,36	-1,08	-0,4	0,38	16,5	0,22	2	1	1	1
-1,61	2,64	0,35	-2,01	16,5	0,59	2	1	1	1
-1,55	-0,34	-1,19	-1,54	16	-0,26	1	1	1	1
-3,02	-1,03	-2,53	-2,96	13	-2,72	2	2	0	1
1,38	-1,12	0,21	1,26	18	0,85	2	1	1,7	1
-0,92	-1,17	-1,31	-0,85	15	-0,92	2	1	1	1
0,26	-2,61	-1,4	0,55	15,4	-0,62	2	2	0	1
-1,37	-0,79	-1,39	-1,37	14,6	-1,36	2	2	0	1
-0,4	-1,29	-1,05	-0,37	15,5	-0,55	2	2	0	1
0,21	-1,31	-0,68	0,27	17	0,45	2	2	0	1
-1,07	-0,74	-1,13	-1,04	15	-0,7	1	2	0	1
-2,5	-1,45	-2,41	-2,3	14,5	-1,1	2	2	0	1
-0,91	-0,78	-1,07	-0,92	16,6	-0,02	2	2	0	1
0,64	0,5	0,71	0,57	17,5	1,1	2	2	0	1
0,69	-3,56	-1,72	0,99	16	-0,1	1	2	0	1
-1,46	-1,03	-1,59	-1,43	15,5	-0,28	2	2	0	1
0,21	-1,65	-0,89	0,29	15,5	-0,7	2	2	0	1
-0,77	0,23	-0,32	-0,74	17	0,42	2	2	0	1
-2,06	-1,29	-2,15	-2,02	15	-0,61	2	2	0	1
-0,37	-1,71	-1,28	-0,23	16	-0,12	1	2	0	1
-1,83	-1,02	-1,84	-1,83	15	-1,01	2	2	0	1
1,04	1,37	1,62	1,2	19	1,69	2	2	0	1
-0,85	-0,39	-0,76	-0,83	18	0,77	1	2	0	1
-1,79	-1,59	-2,17	-1,74	15,5	-0,79	2	2	0	1
1,93	-0,84	0,79	1,98	17,5	1,12	2	2	0	1
-2,11	-2,25	-2,79	-1,91	14,5	-1,25	2	2	0	1
-1,37	-0,68	-1,32	-1,38	17	0,37	2	1	1	1
-1,47	-0,94	-1,56	-1,48	15	-0,9	2	2	0	1
-0,99	-1,11	-1,28	-0,89	15,5	-0,33	2	2	0	1
-0,12	-0,92	-0,61	-0,08	17	0,69	2	2	0	1
0,44	-0,93	-0,28	0,48	18	1,09	2	2	0	1
-1,65	1,4	-0,19	-1,6	15,5	-0,6	2	2	0	1
-0,99	-1,35	-1,44	-0,88	16	0,15	2	1	1	1
-0,96	-1,24	-1,38	-0,9	16	-0,09	1	2	0	1
-1,14	-0,51	-1,04	-1,14	16,5	0,67	2	1	1	1
-1,53	-0,4	-1,26	-1,58	15,5	-0,16	2	1	1	1
-0,42	-1,61	-1,22	-0,29	16	-0,02	2	1	1	1
0,86	-0,98	-0,02	0,85	17,5	0,84	1	1	1	1
0,48	-1,76	-0,68	0,7	16	0,06	2	2	0	1
-2,28	-2,58	-2,96	-1,92	15	-0,59	2	1	1	1
-1,33	-1,51	-1,8	-1,25	15,5	-0,5	2	2	0	1
1,21	-1,87	-0,28	1,43	17	0,75	2	1	1	1
-0,87	-0,53	-0,89	-0,88	14	-1,47	2	1	1	1

-1,99	-2,12	-2,55	-1,77	15	-1,02	2	1	1	1
0,09	-1,85	-1,05	0,18	17	0,49	2	2	0	1
-0,05	-0,37	-0,24	-0,06	17,5	1,31	2	2	0	1
-1,09	-1,89	-1,83	-0,89	14,5	-1,32	2	2	0	1
1,21	-1,02	0,16	1,24	18	1,25	1	2	0	1
-2,86	-1,37	-2,6	-2,71	14,2	-1,17	2	1	1	1
-1,64	-1,25	-1,81	-1,55	15	-0,89	1	2	0	1
0,26	-0,49	-0,08	0,3	17	1	2	2	0	1
0,49	-0,45	0,04	0,48	16	-0,04	2	2	0	1
-1,8	-1,53	-2,04	-1,61	14,8	-0,94	2	2	0	1
0,94	-1,28	-0,15	0,93	16	-0,22	1	2	0	1
1,52	-0,01	0,99	1,46	17,4	0,69	2	2	0	1
0,01	-1,58	-0,94	0,08	15,4	-0,58	2	2	0	1
-0,73	-1,51	-1,38	-0,63	15,2	-0,94	2	2	0	1
0,16	-0,49	-0,2	0,16	16,5	0,26	2	2	0	1
-1,49	-1,66	-1,98	-1,36	14,5	-0,96	2	2	0	1
0,36	-0,96	-0,36	0,32	16	-0,32	2	2	0	1
-0,92	-1,54	-1,53	-0,81	14	-1,84	2	1	1	1
-1,06	-0,86	-1,2	-1,02	15,7	-0,49	1	2	0	1
0,86	0,44	0,89	0,94	16,4	0	1	2	0	1
0,78	0,36	0,71	0,69	17	0,9	2	1	1	1
-0,92	-0,92	-1,16	-0,88	14,6	-1,21	2	2	0	1
0,46	-0,59	-0,06	0,41	17	0,29	2	1	1	1
2,54	-0,45	1,42	2,55	17,7	1,18	1	1	1	1
2,71	-0,65	1,42	2,74	17,6	1,11	1	1	1	1
0,04	-0,95	-0,55	0,03	16	-0,19	1	2	0	1
0,54	-0,98	-0,21	0,58	15	-0,8	1	2	0	1
-0,51	-1,13	-1,02	-0,45	14,4	-1,49	1	2	0	1
1,65	-1,55	0,12	1,7	16,6	0,28	2	2	0	1
-0,73	-0,93	-1,06	-0,73	14	-1,72	1	1	8	1
0,09	-1,31	-0,71	0,16	14,8	-0,95	1	2	0	1
-0,37	-0,29	-0,42	-0,41	15,5	-0,26	2	2	0	1
0,54	-0,11	0,29	0,5	15	-0,79	1	1	1	1
0,18	0,96	0,74	0,23	17	0,49	1	2	0	1
-0,08	0,4	0,2	-0,09	16	-0,26	1	2	0	1
-0,4	-0,84	-0,78	-0,37	15	-0,92	2	2	0	1
-0,19	1,47	0,73	-0,22	16	-0,04	2	1	1	1
-0,28	-1,17	-0,89	-0,21	17	0,38	1	1	1	1
-0,79	0,15	-0,36	-0,72	15,2	-1,07	1	1	1	1
-0,75	0,65	-0,04	-0,68	15,2	-0,94	1	1	1	1
0,04	-1,13	-0,67	0,1	16	-0,1	1	1	1	1
-1,69	0,64	-0,76	-1,82	15,5	-0,43	2	2	0	1
-0,21	-0,45	-0,37	-0,15	15,6	-0,65	1	2	0	1
-0,67	0,01	-0,37	-0,61	15,6	-0,65	1	2	0	1
-0,17	-0,21	-0,21	-0,12	16,4	-0,02	1	2	0	1
-2,42	-0,69	-2,02	-2,46	15	-0,98	1	2	0	1
	-0,67	-0,57	-0,25	17		1	2	0	1

0,09	-0,27	-0,09	0,12	16,4	0,03	1	2	0	1
	-0,45	-0,65	-0,61	17,5		1	2	0	1
	-0,36	0,51	1,11	16		1	2	0	1
1,09	-0,36	0,51	1,11	18	1,08	1	2	0	1
-1,04	0,15	-0,56	-1,02	15,5	-0,75	1	2	0	1
-0,65	-0,44	-0,7	-0,68	15,5	-0,4	1	2	0	1
-0,35	-0,39	-0,47	-0,38	16,4	-0,02	1	2	0	1
0,34	0,32	0,41	0,29	18	1,31	1	2	0	1
-0,78	-2,42	-1,92	-0,44	14,9	-0,93	1	2	0	1
0,2	0,01	0,13	0,16	17	0,68	1	2	0	1
0,06	-0,1	-0,03	0,01	17,5	0,89	1	2	0	1
0,8	-0,54	0,23	0,85	17,5	0,66	1	2	0	1
0,64	1,7	1,43	0,55	17,5	1,13	1	2	0	1
0,11	-0,77	-0,39	0,14	15	-0,8	1	2	0	1
0,68	-0,79	-0,04	0,71	17	0,42	1	2	0	1
2,26	-1,03	0,86	2,22	16	-0,3	1	2	0	1
0,21	-0,62	-0,23	0,22	16,5	0,44	1	2	0	1
-0,65	-0,45	-0,69	-0,66	16	-0,41	1	2	0	1
-0,99	0,09	-0,6	-1,03	15,9	-0,23	1	2	0	1
-0,99	-0,41	-0,86	-0,96	15,3	-0,84	1	2	0	1
	-2,29	-1,75	-0,3	16,1		1	2	0	1
0,02	-0,13	-0,03	0,07	17,5	0,72	1	2	0	1
1,26	-1,34	-0,01	1,15	17,6	0,61	1	2	0	1
	-1,98	-1,22	0,16	16,3		1	2	0	1
-1,68	0,41	-0,77	-1,59	15,5	-0,82	1	2	0	1
1,52	-1,15	0,27	1,38	18,8	1,28	1	2	0	1
-0,34	-1,01	-0,85	-0,37	16,8	0,02	1	2	0	1
-1,39	-1,2	-1,65	-1,35	13,8	-1,78	1	2	0	1
-0,81	1,46	0,36	-0,77	17	0,49	1	2	0	1
-1,35	-0,45	-1,15	-1,36	14,1	-1,71	1	2	0	1
-0,02	0,18	0,14	0,04	16,5	0,04	1	2	0	1
-0,3	-0,77	-0,66	-0,32	16,5	-0,21	1	2	0	1
0,58	-0,77	-0,09	0,6	16,3	0,14	1	2	0	1
	-0,37	-0,01	0,28	18		1	2	0	1
	-0,43	-0,43	-0,26	16		1	2	0	1
-0,38	-0,89	-0,8	-0,35	15,3	-0,69	1	2	0	1
-0,77	-1,61	-1,48	-0,65	15,6	-0,52	1	2	0	1
-0,29	-0,72	-0,59	-0,23	17,2	0,47	1	2	0	1
-0,46	0,35	-0,05	-0,43	16	-0,27	1	2	0	1
-0,53	1,14	0,46	-0,39	15,8	-0,52	1	2	0	1
	0,02	0,02	-0,03	17,8		1	2	0	1
-1,21	-0,53	-1,12	-1,22	14,6	-1,38	1	2	0	1
-0,22	0	-0,16	-0,28	16,1	0,05	1	2	0	1
-0,18	0,78	0,34	-0,2	16,5	0,17	1	2	0	1
-0,16	-0,51	-0,4	-0,16	16,4	0,32	1	2	0	1
	-1,7	-0,81	0,39	17		1	2	0	1
	-0,2	0,29	0,55	17		1	2	0	1

-0,76	-0,42	-0,75	-0,77	16	-0,42	1	2	0	1
-1,27	-1,15	-1,55	-1,26	14,8	-1,11	1	2	0	1
-1,48	-0,55	-1,28	-1,47	15,4	-0,65	1	2	0	1
	-0,78	-0,16	0,44	17,2		1	2	0	1
-0,11	-1,46	-0,94	0,01	16,1	0,04	1	2	0	1
	-0,02	-0,21	-0,35	17		1	2	0	1
	-0,06	-0,01	0	17		1	2	0	1
1,28	-0,72	0,41	1,23	17,1	0,55	1	2	0	1
-1,66	-0,21	-1,22	-1,73	15	-0,72	1	2	0	1
0,37	0,93	0,83	0,44	17,8	0,84	1	2	0	1
-0,87	1,01	0,04	-0,94	15,5	-0,51	1	2	0	1
	0,83	-0,1	-0,94	15,8		1	2	0	1
-0,06	-0,17	-0,15	-0,09	16,2	-0,07	1	2	0	1
0,14	-0,98	-0,52	0,18	15,9	-0,23	1	2	0	1
-0,02	-1,14	-0,72	-0,03	16,4	0	1	2	0	1
	-0,79	-0,92	-0,66	15,9		1	2	0	1
-1,55	-1,78	-2,06	-1,4	14	-1,96	1	2	0	1
0,67	-3,48	-1,61	1,18	15,5	-0,4	1	2	0	1
-0,41	0,92	0,35	-0,3	17	0,28	1	2	0	1
0,88	0,09	0,62	0,83	17	0,52	1	2	0	1
	0,3	0,45	0,36	17,7		1	2	0	1
1,3	0,79	1,32	1,27	18	1,28	1	2	0	1
-0,48	-1,62	-1,22	-0,26	14,5	-1,13	1	2	0	1
-0,34	-0,26	-0,36	-0,34	17	0,23	1	2	0	1
-0,69	-1,89	-1,61	-0,6	16,5	0,08	1	2	0	1
0,75	-0,7	0,06	0,77	17	0,51	1	2	0	1
	-0,73	-0,69	-0,36	15,6		1	2	0	1
-1,37	-1,14	-1,55	-1,28	14,2	-1,48	1	2	0	1
-1,33	-2,01	-2,07	-1,14	16	-0,25	1	2	0	1
0,01	-1,19	-0,74	-0,04	16,5	-0,19	1	2	0	1
0,72	0,12	0,54	0,67	18	1,45	1	2	0	1
-0,02	1,4	0,82	0	16	-0,13	1	2	0	1
0,47	-1,02	-0,32	0,51	15,7	-0,52	1	2	0	1
-0,54	-0,16	-0,43	-0,53	16,5	0,11	1	2	0	1
	-0,47	-0,15	0,18	17,5		1	2	0	1
0,45	1,84	1,51	0,75	17,5	0,47	1	2	0	1
2,2	-3,17	-0,53	2,37	16,5	0,11	1	2	0	1
-0,63	-1,22	-1,16	-0,56	15,5	-0,5	1	2	0	1
0,54	-0,56	0	0,5	16,2	-0,07	1	2	0	1
	-0,47	-0,51	-0,35	16,5		1	2	0	1
-0,2	-2,96	-1,9	0,19	17	0,58	1	2	0	1
-1,72	-1,61	-2,12	-1,62	15	-0,83	1	2	0	1
0,64	-0,62	0,05	0,67	18	1,11	1	2	0	1
-1,15	-1,45	-1,65	-1,1	15,5	-0,61	1	1	1	1
-1,11	0,58	-0,41	-1,21	17,5	0,97	1	2	0	1
	0,62	0,44	0,08	18		1	2	0	1
	-1,65	-0,26	1,06	17,8		1	1	1	1

	-0,31	-0,38	-0,31	16,5		1	2	0	1
-1,01	1,21	0,2	-0,79	18	0,77	1	2	0	1
-0,17	0,38	0,2	-0,07	16,3	-0,32	1	2	0	1
-0,47	-0,15	-0,33	-0,4	17	0,32	1	2	0	1
-0,96	0,24	-0,46	-0,94	18	0,93	1	2	0	1
-0,46	-1,08	-0,95	-0,4	18	1,35	1	2	0	1
-1,87	-1,92	-2,43	-1,78	16	-0,42	1	2	0	1
1,34	-1,86	-0,3	1,36	18,6	1,39	1	1	2	1
-0,91	1,29	0,25	-0,78	18	0,95	1	1	1	1
0,13	0,02	0,09	0,11	18	1,26	1	1	1	1
	0,25	0,51	0,51	17,8		1	2	0	1
1,02	-1,04	0,02	0,93	17,5	0,58	1	1	1	1
-3,18	0,48	-1,75	-3,32	17	0,55	1	2	0	1
-0,72	-0,87	-1	-0,73	16	-0,45	1	2	0	1
1,23	-1,99	-0,34	1,32	17	0,55	1	1	1	1
	-1,38	-1,76	-1,27	15,8		1	1	1	1
-1,82	0,45	-0,9	-1,89	15,5	-0,58	1	2	0	1
-0,86	-2,3	-2	-0,76	16,5	-0,08	1	1	1	1
-0,7	1,09	0,31	-0,52	17,3	0,37	1	2	0	1
	-1,14	-0,68	0,08	17,5		1	2	0	1
-0,42	0,08	-0,21	-0,41	16,5	-0,04	1	2	0	1
0,42	-0,18	0,22	0,49	17,1	0,4	1	2	0	1
	0,75	0,03	-0,66	18,4		1	2	0	1
0,09	-1,28	-0,72	0,15	16,5	0,03	1	2	0	1
1,66	-1,58	0,12	1,56	19	1,5	1	2	0	1
-2,02	-1,72	-2,31	-1,88	14,7	-1,34	1	2	0	1
	-1	-0,81	-0,28	16,5		1	2	0	1
	-0,65	-0,91	-0,8	16,1		1	2	0	1
	-0,96	-0,96	-0,53	16		1	2	0	1
1,39	-1,25	0,23	1,45	17,5	0,98	1	2	0	1
0,01	-2,57	-1,59	0,11	15,5	-0,74	1	2	0	1
1,03	-0,78	0,19	1,04	18,5	1,62	1	2	0	1
0,1	-0,48	-0,23	0,07	16,9	0,21	1	2	0	1
	-0,4	0,91	1,59	19		1	2	0	1
-0,78	-1,54	-1,43	-0,65	15	-0,88	1	2	0	1
0,69	-0,78	-0,02	0,71	15,9	-0,14	1	2	0	1
	-0,37	-0,03	0,26	17		1	2	0	1
	0,43	1,36	1,58	20		1	2	0	1
0,03	-0,51	-0,3	0,04	15,3	-0,71	1	2	0	1
0,4	0,23	0,45	0,47	18	1,05	1	2	0	1
	0,24	0,69	0,79	17,9		1	2	0	1
-0,09	0,22	0,09	-0,09	17	0,5	1	2	0	1
	-1,15	-0,59	0,17	16,5		1	2	0	1
3,81	-1,98	1,44	3,84	18,5	1,6	1	2	0	1
-0,15	-0,61	-0,44	-0,1	16,5	0,04	1	2	0	1
	-2,04	-1,79	-0,65	17,5		1	2	0	1
0,42	-0,63	-0,12	0,38	17	0,4	1	2	0	1

	-1,03	-2,1	-2,17	15,5		1	2	0	1
0,25	-0,27	-0,01	0,22	16,5	0,41	1	2	0	1
1,81	-0,77	0,78	1,81	18,3	1,5	1	2	0	1
-0,41	-1,21	-0,98	-0,33	16	-0,38	1	2	0	1
-1,02	-1,12	-1,35	-1,01	16	-0,55	1	2	0	1
0,76	-1,41	-0,31	0,9	17	0,79	1	2	0	1
0,12	-1,01	-0,52	0,17	16	-0,35	1	2	0	1
-0,28	-1,98	-1,39	-0,15	16,1	-0,31	1	1	1	1
0,17	-2,25	-1,29	0,3	15,7	-0,57	1	1	1	1
-1,07	-0,65	-1,11	-1,09	15,5	-0,59	1	2	0	1
	-0,29	-0,58	-0,65	16,5		1	2	0	1
	-1,56	-1,08	-0,09	16		1	2	0	1
0,72	-0,08	0,41	0,67	17	0,53	1	2	0	1
-0,71	-1,21	-1,19	-0,64	15	-0,75	1	2	0	1
0,61	-1,29	-0,38	0,69	16,7	0,45	1	2	0	1
-0,66	-2,28	-1,82	-0,49	16	-0,21	1	2	0	1
0	-2,39	-1,51	-0,01	18	0,68	1	2	0	1
	-2,15	-1,23	0,25	16,3		1	2	0	1
0,66	0,17	0,59	0,73	18	1,04	1	1	1	1
-0,99	-2,35	-2,13	-0,89	14,9	-1,23	1	2	0	1
-2,21	-0,18	-1,59	-2,3	16	-0,14	1	2	0	1
1,39	-1,38	0,1	1,36	17	0,45	1	2	0	1
0,49	1,25	1,14	0,64	19,5	1,73	1	1	1	1
0,34	-1,85	-0,93	0,43	16	-0,29	1	2	0	1
0,02	-0,06	0	0,03	17,5	0,53	1	2	0	1
-0,65	-2,14	-1,76	-0,57	15,2	-0,98	1	2	0	1
-1,62	-0,31	-1,22	-1,61	14,6	-1,43	1	1	1	1
-0,48	1,28	0,41	-0,64	16,4	0,34	1	2	0	1
0,34	-1,13	-0,48	0,3	17	0,33	1	2	0	1
	0,06	-0,85	-1,38	17,5		1	2	0	1
-2,11	-1,12	-2,02	-2,05	14	-1,89	1	2	0	1
	-1,15	-1,09	-0,54	18		1	2	0	1
-1,02	-0,84	-1,16	-1,01	15,3	-1,02	1	2	0	1
-0,57	-1,52	-1,33	-0,56	15,7	-0,6	1	2	0	1
	-1,46	-0,39	0,83	17,2		1	2	0	1
-0,62	-0,38	-0,65	-0,65	16	-0,05	1	2	0	1
-1,56	-0,98	-1,63	-1,55	15	-0,8	1	2	0	1
	-0,78	-0,82	-0,52	16,9		1	2	0	1
-0,7	-1,14	-1,17	-0,7	16,3	-0,1	1	2	0	1
0,08	0,2	0,18	0,09	16	-0,2	1	2	0	1
	0,3	-0,14	-0,55	17,5		1	2	0	1
-0,8	-1,53	-1,45	-0,7	15	-0,82	1	2	0	1
-0,15	-1,54	-1,05	-0,06	16	-0,26	1	2	0	1
-1,37	-1,04	-1,53	-1,35	15,1	-1,12	1	2	0	1
	1,45	2	1,65	18,4		1	2	0	1
	-0,56	-0,19	0,2	18		1	1	1	1
	-1,59	-1,76	-1,05	14,3		1	2	0	1

2	-0,62	0,94	2,01	18,4	1,54	1	2	0	1
-0,96	-2,09	-1,93	-0,85	14,5	-1,43	1	2	0	1
-0,57	-0,52	-0,67	-0,57	17	0,15	1	2	0	1
-0,27	-0,97	-0,78	-0,29	16	-0,37	1	2	0	1
0,02	-0,81	-0,49	0	16,2	-0,08	1	2	0	1
	-0,7	-0,08	0,54	18,5		1	2	0	1
-1,32	0,68	-0,36	-1,21	15,8	-0,58	1	2	0	1
0,09	-1,45	-0,79	0,17	15,4	-0,52	1	2	0	1
-0,59	-1,21	-1,14	-0,59	15,3	-0,8	1	2	0	1
0,1	-0,52	-0,23	0,11	16,2	0,2	1	2	0	1
-0,95	-0,34	-0,85	-1,01	15,5	-0,38	1	2	0	1
0,34	-0,37	0	0,31	18	0,92	1	2	0	1
-0,53	-0,87	-0,88	-0,5	15,5	-0,48	1	2	0	1
-1,45	-2,31	-2,25	-1,09	14,5	-1,18	1	2	0	1
-1,11	-1,12	-1,42	-1,1	15,7	-0,55	1	2	0	1
0,34	-1,9	-0,94	0,49	16,5	0,23	1	2	0	1
-1,23	-2,17	-2,1	-1,05	14,3	-1,72	1	2	0	1
-1,13	-0,26	-0,92	-1,18	14,2	-1,49	1	2	0	1
-1,06	-1,78	-1,77	-0,91	15	-1,03	1	2	0	1
0,72	-1,38	-0,35	0,73	17,4	0,73	1	2	0	1
0,58	-1,06	-0,27	0,62	17	0,43	1	2	0	1
0,25	-0,73	-0,26	0,28	15,9	-0,01	1	2	0	1
-0,59	-1,27	-1,16	-0,51	15,7	-0,35	1			
0,05	-1,19	-0,71	0,01	16,5	-0,09	1	2	0	1
1,09	0,2	0,85	1,08	18	0,82	1	2	0	1
-0,19	-1,43	-1,01	-0,19	16	-0,35	1	2	0	1
	-1,5	-0,9	0,12	15		1	2	0	1
0,18	-0,65	-0,28	0,21	16,3	-0,04	1	2	0	1
	-1,1	-1,28	-0,89	15,5		1	2	0	1
	0,49	0,92	0,9	16,9		1	2	0	1
0,43	0,62	0,64	0,36	17,3	0,93	1	2	0	1
-1,61	-0,65	-1,42	-1,59	15	-1,01	1	2	0	1
-0,15	-0,33	-0,26	-0,13	17,4	0,33	1	2	0	1
1,78	-0,28	1,01	1,79	18	1,2	1	2	0	1
1,18	-0,28	0,61	1,19	17	0,54	1	2	0	1
-0,03	-0,83	-0,54	-0,07	16	-0,4	1	2	0	1
1,22	-1,47	-0,12	1,24	18	1,06	1	2	0	1
0,2	-0,92	-0,43	0,18	16,5	0,08	1	2	0	1
1,95	-0,48	1,01	1,97	16,5	0,35	1	2	0	1
-0,99	-0,53	-0,97	-0,99	15,3	-0,9	1	2	0	1
0,21	0,78	0,72	0,35	17	0,34	1	2	0	1
0,36	-0,06	0,19	0,32	16	-0,26	1	2	0	1
-0,2	0,48	0,22	-0,12	17	0,19	1	2	0	1
-1,23	1,99	0,49	-1,01	15,5	-0,68	1	2	0	1
-1,58	0,62	-0,52	-1,41	14	-2,02	1	2	0	1
-0,1	-1,08	-0,73	-0,05	15,5	-0,63	1	2	0	1

Immunisat	Stiff MM p	Soft MM p	Samp	Rice	Macaroni	spaghetti	Oats	Cornflakes	All bran
1	2,7	1,7	10	3,6	1,5	1,5	10	1,5	1,5
1	2,7	1,7	10	3,6	1,5	1,5	10	1,5	1,5
1	1,7	2,7	1,5	1,5	1,5	1,5	10	1,5	10
1	3,7	1,7	1,5	1,5	1,6	1,5	10	1,6	10
1	3,7	1,6	1,8	2,6	1,6	10	10	1,6	10
1	2,6	10	1,5	2,5	10	1,5	1,6	10	10
1	1,6	2,7	1,5	1,5	1,5	10	10	10	10
1	2,7	1,7	10	1,5	1,6	1,5	1,6	10	10
1	2,7	1,7	10	2,6	2,5	2,5	10	1,5	10
1	2,7	1,6	1,5	1,5	1,6	1,6	10	1,7	10
1	2,7	2,6	2,6	2,6	1,5	1,5	10	10	10
1	2,7	2,7	10	2,5	1,6	1,6	10	1,5	10
1	3,7	2,5	2,5	2,6	2,5	2,5	10	1,6	10
1	2,7	10	2,5	2,6	1,5	10	10	1,7	10
1	1,5	3,6	1,5	1,5	1,5	1,5	10	1,5	1,5
1	3,7	1,6	10	1,5	1,5	1,5	1,6	1,6	1,6
1	3,6	1,6	10	1,5	1,5	1,5	1,6	1,6	1,6
1	3,7	1,5	2,5	2,6	2,5	2,5	1,5	1,5	10
1	1,7	1,5	2,5	2,5	1,5	1,5	10	10	10
1	3,6	2,5	2,5	2,5	2,5	2,5	10	1,5	10
1	3,7	2,6	10	2,5	2,5	2,5	10	10	10
1	2,6	1,6	1,5	2,6	2,5	2,5	10	1,5	10
1	2,7	1,6	1,5	2,5	2,5	2,5	10	1,5	10
1	3,7	1,5	2,5	2,5	2,5	2,5	10	10	10
1	2,7	1,6	2,5	2,5	2,5	2,5	1,8	1,5	10
1	3,7	1,6	2,5	2,6	2,5	2,5	10	2,5	10
1	4,7	1,7	1,5	1,5	10	10	10	10	10
1	1,6	1,5	1,5	1,5	1,5	1,5	1,5	1,5	10
1	3,7	1,7	1,5	2,5	10	10	10	10	10
1	3,7	1,6	1,5	1,5	2,6	2,6	10	10	10
1	2,7	1,5	1,5	1,5	1,5	10	10	10	10
1	2,7	1,7	1,5	2,5	1,5	1,5	10	1,5	10
1	3,7	1,6	1,5	2,5	10	10	10	1,5	10
1	2,7	10	1,5	1,5	10	1,8	10	1,5	10
1	1,6	1,7	1,5	1,5	1,8	10	10	10	10
1	1,7	1,6	2,5	2,5	1,5	10	10	10	10
1	3,7	1,6	1,6	1,7	10	10	10	10	10
1	1,7	10	10	1,5	10	10	10	10	10
1	3,7	1,5	1,5	1,5	10	10	10	1,5	10
1	2,7	2,7	1,5	1,5	1,6	1,6	10	1,7	10
1	2,7	1,7	1,5	1,5	1,6	1,5	1,7	1,5	10
1	2,7	1,7	1,5	1,5	1,6	1,6	10	1,6	10
1	2,7	1,6	1,5	1,5	10	10	10	10	10
1	2,7	1,7	1,5	1,5	1,5	1,5	10	1,5	10
1	1,6	2,7	1,5	1,5	10	1,5	10	1,8	1,8
1	1,7	10	1,5	1,5	10	10	10	10	1,6

1	2,7	10	1,5	1,5	10	10	10	10	10
1	3,7	1,5	2,8	3,5	10	10	10	1,5	10
1	2,6	1,5	1,5	1,5	10	10	10	10	10
1	2,7	2,7	1,5	1,5	1,5	1,5	1,5	1,5	10
1	2,7	1,7	1,7	1,5	1,7	1,7	10	1,5	10
1	2,7	1,6	1,8	1,5	10	10	10	1,7	10
1	1,5	1,6	1,5	1,5	1,5	10	10	1,5	10
1	3,7	2,7	1,5	2,5	10	10	10	1,6	10
1	3,7	2,7	1,5	2,6	10	10	10	10	10
1	3,7	2,7	1,5	2,6	1,5	1,5	1,6	1,6	1,6
1	2,7	2,7	1,5	2,6	1,5	1,5	10	10	10
1	4,7	2,7	1,5	2,6	10	10	10	10	10
1	3,7	2,7	2,5	2,6	1,5	1,5	1,5	1,5	10
1	2,6	2,7	2,6	1,5	1,6	1,6	10	1,5	10
1	2,7	1,5	10	2,5	3,5	10	10	2,6	10
1	2,7	10	1,8	10	10	1,5	10	10	10
1	2,7	1,6	1,5	1,6	10	10	10	1,6	10
1	3,7	2,6	1,6	2,6	10	10	10	10	10
1	1,5	3,7	10	1,5	1,5	1,5	10	1,5	10
1	3,7	1,6	1,8	1,5	10	10	1,5	10	10
1	3,7	1,6	2,6	2,6	2,5	2,5	10	10	10
1	3,7	1,6	2,5	2,5	2,5	2,5	10	1,8	10
1	3,7	1,6	2,5	2,5	2,5	2,5	10	1,8	10
1	1,5	3,7	10	1,5	10	10	10	1,8	10
1	1,5	3,7	10	1,5	10	10	10	1,8	10
1	3,7	1,7	2,7	2,7	10	10	10	10	10
1	3,7	1,7	10	1,5	10	10	10	10	10
1	2,7	10	1,8	1,6	1,6	10	10	1,7	10
1	2,7	1,7	1,5	1,5	1,6	1,6	1,7	3,7	10
1	2,7	1,7	10	10	10	10	10	10	10
1	2,7	10	1,5	2,5	10	10	10	10	10
1	2,7	10	1,5	2,5	10	10	10	10	10
1	2,7	2,6	1,7	1,5	10	10	10	10	10
1	2,7	1,7	10	1,6	1,5	1,5	10	10	10
1	3,7	1,6	1,5	1,5	1,6	1,5	1,7	1,7	10
1	3,7	1,5	10	2,6	2,5	2,5	10	1,6	10
1	3,7	1,6	2,5	2,5	2,5	2,5	10	10	10
1	3,7	10	1,8	1,5	10	10	10	10	10
1	3,7	2,7	1,5	1,5	1,5	1,5	10	10	10
1	3,6	2,7	2,5	3,6	1,5	1,5	1,5	10	10
1	2,6	1,5	1,5	2,5	1,5	1,5	1,5	1,6	10
1	2,7	1,7	10	1,5	1,5	1,5	10	1,6	10
1	2,6	1,5	10	2,5	10	1,5	10	1,5	10
1	2,6	1,5	10	1,5	10	1,5	10	1,5	1,5
1	2,6	1,5	10	1,5	10	1,5	10	1,6	1,5
1	2,6	1,5	10	1,5	10	1,5	10	1,6	1,6
1	3,7	1,6	1,5	2,6	1,5	1,5	1,5	1,5	10

1	2,7	10	1,5	1,5	1,5	10	10	10	10
1	3,5	1,5	10	1,6	10	10	10	10	10
1	2,7	1,7	1,5	1,6	10	10	1,5	1,5	10
1	2,7	2,7	1,5	2,6	1,8	1,8	10	1,6	10
1	3,7	1,5	1,5	1,5	10	10	10	10	10
1	2,6	1,7	1,5	1,6	1,5	1,5	10	10	10
1	3,7	2,6	1,5	2,5	1,5	1,5	10	1,5	10
1	1,7	1,6	1,5	1,5	1,5	10	1,5	1,5	10
1	2,7	1,6	1,5	1,5	1,8	10	10	10	10
1	2,7	2,5	10	1,5	1,5	1,5	1,5	10	10
1	4,7	3,7	1,5	1,6	10	10	10	10	10
1	1,7	10	10	1,5	10	1,5	10	10	10
1	3,6	1,5	1,5	3,5	10	1,5	10	1,6	10
1	3,6	1,5	1,5	2,5	1,5	1,5	10	1,6	10
1	4,7	3,8	1,7	1,6	10	10	10	10	10
1	3,7	2,7	10	2,5	2,5	2,5	1,8	10	10
1	2,7	1,7	1,6	1,5	10	10	10	10	10
1	3,7	1,7	10	1,5	1,5	1,5	10	10	10
1	2,6	1,6	1,5	2,5	1,5	1,5	10	1,5	10
1	1,7	10	1,5	1,5	1,5	10	10	1,5	10
1	2,7	1,5	1,8	1,5	1,5	1,5	10	1,5	10
1	2,8	1,8	10	1,5	10	1,8	10	10	10
1	3,6	1,7	1,5	1,6	1,5	1,5	10	1,5	10
1	3,6	1,7	1,5	1,6	1,5	1,5	10	1,5	10
1	3,6	1,7	1,5	1,6	1,5	1,5	10	1,5	10
1	3,7	1,5	10	3,5	10	10	10	4,5	10
1	2,7	1,7	1,5	1,5	1,5	1,5	10	1,5	10
1	2,7	10	1,7	1,5	1,5	10	10	10	10
1	2,7	10	10	1,5	1,5	10	10	10	10
1	2,6	1,6	1,5	1,5	1,5	1,5	1,5	1,5	10
1	2,7	2,7	1,5	2,6	1,6	1,6	1,7	1,5	10
1	2,7	1,6	3,5	1,5	2,5	2,5	1,5	1,5	1,5
1	2,7	1,6	2,5	2,6	2,5	2,5	10	1,9	10
1	2,7	1,5	1,5	1,5	1,7	1,7	1,5	1,5	1,5
1	2,7	1,6	1,7	1,7	1,7	1,7	10	1,6	10
1	2,7	1,5	1,5	1,5	1,6	1,6	10	10	10
1	2,7	1,6	2,6	2,6	1,7	1,7	1,7	1,7	1,7
1	2,7	1,7	10	2,5	1,5	1,5	2,5	1,6	1,5
1	2,7	1,5	1,5	2,8	2,8	2,8	10	10	10
1	2,7	1,6	10	1,6	10	10	10	10	10
1	2,7	1,7	10	2,5	1,6	1,6	1,7	1,7	1,7
1	2,7	1,5	1,5	1,5	1,5	1,5	10	1,5	1,5
1	3,7	1,6	1,8	1,5	10	10	10	1,6	10
1	2,7	1,6	1,5	1,5	2,5	2,5	10	10	10
1	3,7	1,6	1,5	2,5	2,5	2,5	10	1,6	10
1	3,7	2,6	10	1,5	1,5	1,5	10	1,5	10
1	3,7	1,5	1,8	2,6	2,5	2,5	10	1,5	10

1	3,7	10	10	1,5	1,5	10	10	10	10
1	2,7	10	2,8	2,8	10	1,6	10	10	10
1	2,6	1,5	1,5	2,5	2,5	1,5	10	1,5	1,5
1	2,6	1,5	1,5	2,5	2,5	1,5	10	1,5	1,5
1	3,7	2,7	1,5	1,6	1,5	1,5	10	10	10
1	1,5	3,7	1,5	1,5	1,5	1,5	1,5	1,5	10
1	3,6	1,5	1,5	2,5	2,5	2,5	10	1,5	10
1	2,7	1,6	1,6	1,5	1,5	1,5	1,5	1,6	10
1	2,7	1,5	1,8	1,5	1,5	1,5	10	1,7	10
1	1,7	1,7	1,5	1,5	1,5	10	10	1,5	10
1	3,7	1,5	10	3,5	10	10	3,5	1,8	10
1	3,7	1,5	10	3,5	10	2,5	10	1,8	10
1	3,7	10	1,5	1,5	1,5	10	10	1,8	10
1	1,6	3,7	1,5	2,5	1,5	1,5	1,5	1,6	10
1	2,6	1,6	1,5	1,5	1,5	1,5	1,5	1,6	1,6
1	2,6	1,6	1,5	1,5	1,5	1,5	1,5	1,5	10
1	3,6	1,6	1,6	1,5	1,6	1,6	10	1,6	10
1	3,6	1,6	1,6	1,5	1,6	1,6	10	1,6	10
1	3,7	1,6	1,5	2,6	2,5	2,5	10	10	10
1	2,7	1,6	1,5	1,5	1,5	1,5	10	10	10
1	2,6	1,7	1,5	1,5	3,5	3,5	1,6	1,6	1,6
1	2,7	1,7	1,5	1,5	10	10	10	10	10
1	2,7	1,7	1,5	1,5	10	10	10	10	10
1	2,6	1,5	1,5	1,5	1,7	10	1,5	1,8	10
1	2,6	1,5	1,5	1,5	1,7	10	1,5	1,8	10
1	3,6	1,6	1,6	1,6	1,6	1,6	10	1,5	10
1	2,7	1,6	1,5	1,5	1,5	10	1,5	1,5	10
1	2,7	2,7	2,6	2,6	1,5	1,5	10	1,6	10
1	3,7	10	1,5	1,5	1,5	10	10	1,5	10
1	3,7	2,6	2,5	2,5	1,6	1,6	10	1,6	10
1	2,6	10	1,8	2,5	2,6	2,6	1,5	10	10
1	4,7	10	1,5	1,5	1,5	10	10	1,5	10
1	2,7	1,7	1,5	1,5	1,6	1,6	1,5	1,5	10
1	3,7	2,6	1,5	2,6	2,5	2,5	10	1,5	10
1	2,6	1,6	1,5	2,5	1,5	1,5	10	10	10
1	2,7	10	1,5	1,5	1,5	1,5	10	1,5	10
1	2,7	10	1,5	1,5	1,5	10	10	1,6	10
1	4,8	10	1,5	1,5	1,5	10	10	1,6	10
1	3,6	1,6	1,5	1,5	2,5	2,5	10	2,5	10
1	3,6	1,6	1,5	1,5	2,5	2,5	10	2,5	10
1	2,6	10	1,5	1,5	1,5	10	10	10	10
1	2,6	1,5	1,5	2,5	1,5	1,5	10	10	10
1	3,7	2,7	2,6	1,6	1,5	1,5	10	10	10
1	2,7	1,5	10	1,5	1,6	1,6	1,5	1,5	1,5
1	2,6	1,5	1,5	1,5	1,5	1,5	10	1,5	10
1	2,7	10	10	1,5	1,6	1,6	10	1,6	10
1	3,7	2,6	1,5	1,5	1,5	1,5	10	1,7	10

1	2,7	1,7	1,6	1,6	10	10	10	10	10
1	2,7	10	1,5	1,5	1,5	10	10	1,6	10
1	2,7	10	1,5	1,5	1,5	10	10	10	10
1	3,7	2,6	2,6	2,6	2,6	2,6	1,5	1,5	10
1	3,7	2,6	2,6	2,6	2,6	2,6	1,5	1,5	10
1	2,7	10	1,5	1,5	1,5	10	10	10	10
1	3,7	10	1,5	1,5	1,6	1,6	10	1,6	10
1	3,7	1,8	10	3,5	2,5	2,5	10	2,6	10
1	2,7	1,7	1,5	1,5	1,8	1,8	10	10	10
1	3,7	1,6	1,5	1,5	1,5	1,5	10	10	10
1	2,6	1,5	1,5	1,5	10	10	10	10	10
1	2,6	1,7	1,5	1,5	10	10	10	1,6	10
1	3,7	10	1,5	1,5	1,5	10	10	1,5	10
1	2,7	10	1,5	1,5	1,5	10	10	10	10
1	2,7	10	1,5	1,5	10	10	10	10	10
1	2,7	10	1,5	1,5	10	10	10	10	10
1	3,7	1,7	1,5	1,5	1,5	10	1,5	1,5	10
1	2,7	10	1,5	1,5	1,5	10	10	1,6	10
1	3,7	10	1,5	1,5	1,5	10	10	10	10
1	2,7	2,7	1,5	2,5	1,5	1,5	10	10	10
1	2,7	1,7	1,6	1,6	1,5	1,5	10	10	10
1	2,7	1,5	1,5	1,5	2,5	2,5	10	10	10
1	2,7	1,5	1,5	1,5	2,5	2,5	10	10	10
1	3,7	1,7	10	1,5	10	10	10	1,8	10
1	2,5	1,5	1,5	1,5	1,5	1,5	10	10	10
1	3,7	1,6	1,5	1,5	1,5	1,5	10	1,5	10
1	3,7	2,5	2,5	2,6	1,8	1,8	10	1,5	10
1	2,7	10	1,5	1,5	1,5	10	10	10	10
1	3,7	1,7	10	1,5	10	10	10	1,5	10
1	3,6	2,7	2,5	1,6	1,5	1,5	1,6	1,6	10
1	2,6	1,5	1,5	1,5	10	10	10	1,9	10
1	3,7	1,7	1,5	1,5	1,5	1,5	10	1,5	10
1	2,7	1,5	1,5	1,5	1,5	1,5	10	1,5	10
1	2,6	1,5	1,5	2,5	1,5	1,5	10	1,5	10
1	3,7	10	1,5	1,5	1,5	10	10	10	10
1	2,7	10	1,5	1,5	1,5	10	10	10	10
1	2,6	1,6	1,5	1,5	1,5	1,5	10	1,5	10
1	3,7	2,7	2,5	2,5	1,5	1,5	10	10	10
1	2,7	10	1,5	1,5	1,6	10	10	10	10
1	2,6	1,6	2,5	2,5	2,5	2,5	10	10	10
1	2,7	1,7	1,5	1,5	1,8	1,8	10	1,6	10
1	2,7	1,5	1,5	1,5	1,6	1,6	10	10	10
1	3,7	2,7	1,5	2,6	10	10	10	10	10
1	4,7	1,7	10	1,5	1,5	1,5	10	10	10
1	3,7	2,7	1,5	2,6	10	10	10	10	10
1	2,7	1,7	1,5	1,5	10	10	10	1,5	10
1	3,7	2,6	2,5	2,6	2,6	2,6	1,5	1,6	10

1	3,7	2,6	2,5	2,6	2,6	2,6	1,5	1,6	10
1	1,7	1,5	1,5	1,5	1,5	1,5	10	10	10
1	2,6	1,5	10	1,5	1,5	1,5	10	1,6	10
1	3,7	10	1,5	1,5	1,5	1,5	10	10	10
1	2,7	10	1,5	1,5	1,5	10	10	1,5	10
1	1,7	10	1,5	1,5	1,5	10	10	10	10
1	1,7	10	1,5	1,5	1,5	10	10	1,6	10
1	2,7	10	1,5	1,5	1,5	1,5	10	10	10
1	1,7	10	1,5	1,5	1,5	10	10	1,6	10
1	1,7	10	1,5	1,5	1,5	1,5	10	1,6	10
1	2,6	2,6	1,5	2,6	1,5	1,5	10	2,5	10
1	3,7	1,6	10	1,5	1,5	1,5	10	10	10
1	1,7	1,5	10	1,5	1,5	1,5	10	1,5	10
1	2,7	1,5	10	1,5	1,5	1,5	10	1,5	10
1	1,6	10	1,5	1,5	10	10	1,6	1,5	10
1	2,7	1,6	1,5	1,5	1,7	1,7	10	10	10
1	3,7	1,6	1,8	1,5	1,6	1,6	10	10	10
1	3,7	1,6	1,8	1,5	1,6	1,6	10	10	10
1	1,7	1,5	10	2,5	1,8	1,8	10	10	10
1	3,7	2,7	1,5	2,6	1,5	1,5	10	10	10
1	3,7	1,7	1,5	10	3,5	3,5	10	10	10
1	2,6	1,5	1,5	1,5	1,5	1,5	10	10	10
1	2,7	2,7	1,5	2,6	1,6	1,6	10	10	10
1	2,7	1,6	10	1,5	2,5	2,5	10	10	10
1	2,6	1,6	10	1,5	2,5	2,5	10	10	10
1	3,7	1,7	1,5	1,5	1,8	1,5	1,5	10	10
1	1,6	10	1,5	1,5	10	10	1,6	1,5	10
1	3,7	1,7	10	1,5	10	10	10	10	10
1	3,7	1,6	10	1,5	2,6	2,6	1,6	1,6	1,6
1	2,6	1,6	10	1,5	1,5	1,5	10	1,5	1,5
1	2,6	10	1,5	1,5	1,5	1,5	1,5	1,6	1,6
1	2,7	1,6	10	1,5	1,5	1,5	10	1,8	10
1	2,7	10	1,5	1,5	1,5	10	10	1,7	10
1	2,7	1,5	10	1,5	1,5	10	10	1,7	10
1	2,6	2,6	10	2,6	2,6	2,6	10	10	10
1	2,7	1,5	10	1,5	2,7	2,7	10	10	10
1	1,7	1,5	10	3,5	10	10	10	10	10
1	2,7	10	1,5	1,5	1,5	10	10	1,6	10
1	2,6	1,7	1,5	1,6	1,5	1,5	10	1,5	10
1	2,6	1,5	10	1,5	1,5	1,5	10	10	10
1	2,7	1,5	10	1,5	1,5	1,5	10	10	10
1	2,6	1,5	10	1,5	1,6	1,6	1,5	1,5	10
1	2,6	2,6	10	1,5	10	10	10	1,5	1,5
1	1,6	1,6	10	1,5	10	10	10	1,5	1,5
1	1,7	2,7	3,6	3,6	1,5	1,5	1,8	1,6	10
1	2,7	1,7	1,5	1,5	1,5	1,5	10	1,8	10
1	1,5	1,4	1,6	1,6	1,4	1,4	10	1,4	10

1	2,6	1,5	1,5	2,5	2,6	2,6	1,5	1,7	10
1	1,5	1,5	1,4	1,6	1,4	1,5	10	10	10
1	2,7	1,7	10	1,5	2,5	2,5	10	10	10
1	2,6	2,6	10	1,5	1,5	1,5	1,5	1,5	1,5
1	2,7	10	10	2,6	10	10	10	10	10
1	2,7	10	10	1,5	1,8	1,8	10	10	10
1	2,7	1,5	1,5	1,5	1,5	1,5	10	10	10
1	1,6	2,6	1,6	1,5	1,5	2,6	1,5	1,5	2,5
1	1,5	1,5	1,5	1,4	1,4	1,4	1,6	2,6	10
1	1,5	1,5	1,5	1,4	1,4	1,4	1,6	2,6	10
1	1,5	1,5	1,5	1,5	1,5	2,6	10	2,6	10
1	2,7	1,5	10	3,5	1,5	1,5	1,5	1,5	10
1	2,7	10	10	1,5	1,8	1,8	10	10	10
1	1,5	1,5	1,6	1,5	1,5	1,5	1,6	1,6	1,5
1	1,7	1,7	1,8	1,5	1,5	1,5	10	1,6	10
1	2,5	1,6	10	2,5	1,6	1,6	10	1,6	10
1	2,6	2,6	10	1,5	1,5	1,5	10	1,6	1,5
1	3,7	1,5	1,5	1,5	1,5	1,5	10	1,7	10
1	1,6	1,6	1,6	1,6	1,6	1,6	1,6	1,6	1,6
1	2,6	1,7	1,5	1,5	1,5	1,5	10	1,5	10
1	2,7	2,5	2,5	2,5	10	10	10	10	10
1	3,7	1,7	1,5	1,6	10	10	10	10	10
1	2,7	1,7	1,5	2,6	1,8	1,8	10	10	10
1	2,7	1,7	1,5	2,6	10	10	10	10	10
1	2,6	1,5	1,5	1,5	1,5	1,5	1,5	1,5	10
1	2,7	1,7	10	1,5	10	10	10	1,5	10
1	3,7	2,7	1,5	1,5	10	10	10	10	10
1	2,7	1,5	1,5	1,5	1,5	10	10	10	10
1	2,7	2,7	1,5	2,6	1,6	1,6	10	1,7	10
1	2,7	1,6	1,5	1,5	1,5	1,5	1,5	10	10
1	2,7	2,5	2,5	1,5	1,5	1,5	10	10	10
1	2,7	1,5	1,5	1,5	1,5	1,5	1,5	1,5	1,5
1	2,7	2,5	2,5	1,5	1,5	1,5	10	10	10
1	2,7	1,5	10	1,5	1,5	1,5	10	10	10
1	3,7	2,7	1,5	2,6	10	10	10	10	10
1	2,5	2,5	1,5	1,5	10	10	10	10	10
1	2,7	1,7	1,5	2,5	1,5	1,5	10	1,6	10
1	3,7	1,7	1,5	1,5	10	10	10	1,6	10
1	2,7	1,7	1,5	2,6	10	10	10	10	10
1	2,7	2,7	1,5	1,6	1,5	1,5	10	10	10
1	2,7	1,5	1,5	2,5	1,5	1,5	1,5	1,5	1,7
1	2,5	2,5	1,5	1,5	10	10	10	10	10
1	2,7	1,7	1,5	1,5	1,5	1,5	10	1,5	10
1	2,5	2,5	2,5	2,5	2,5	10	10	10	10
1	2,7	1,6	10	1,5	1,5	1,5	10	10	10
1	2,6	1,6	1,8	2,6	1,5	1,5	10	1,8	10
1	2,6	1,6	10	1,5	1,5	1,5	10	1,5	1,5

1	2,6	1,6	10	1,5	1,5	1,5	10	1,6	1,6
1	2,6	1,7	1,5	1,5	1,5	1,5	1,5	1,5	10
1	3,7	1,5	1,5	1,5	1,5	1,5	10	10	10
1	2,6	1,5	1,5	2,6	1,5	10	10	10	10
1	2,7	1,7	1,5	1,5	1,5	1,5	10	10	10
1	1,5	1,5	1,5	1,5	10	10	10	10	10
1	2,6	1,6	10	1,5	10	10	10	1,5	10
1	2,6	1,6	10	1,5	10	10	10	1,5	1,6
1	2,7	1,6	1,5	1,5	1,5	1,5	1,6	1,6	1,5
1	3,6	2,5	2,6	2,6	2,5	10	10	10	10
1	3,7	1,7	2,5	2,6	10	10	10	10	10
1	2,7	1,5	1,5	1,5	1,5	1,5	10	10	10
1	2,6	2,6	10	1,5	1,5	1,5	10	1,6	10
1	2,6	1,6	10	1,5	1,5	1,5	10	1,5	1,5
1	2,6	1,6	10	1,5	10	10	10	1,5	1,6
1	2,6	1,6	10	1,5	10	10	10	1,5	10
1	2,6	1,6	10	1,5	10	10	10	1,5	10
1	2,6	1,6	10	1,5	1,5	1,5	10	1,5	10
1	2,7	1,7	1,5	1,5	1,5	1,5	10	10	10
1	2,7	1,7	1,5	1,5	1,5	1,5	1,5	1,5	10
1	1,5	2,6	1,5	1,5	1,5	1,5	10	1,5	10
1	3,7	2,7	2,5	2,6	1,6	1,6	10	10	10
1	2,6	1,6	10	1,5	10	10	10	10	10
1	3,7	1,6	1,5	1,5	1,5	1,5	1,5	1,5	1,5
1	2,6	1,6	10	1,5	1,5	1,5	10	1,5	10
1	3,7	2,7	2,5	1,5	1,6	1,6	10	10	10
1	2,6	2,6	10	1,5	1,5	1,5	10	1,5	10
1	2,6	1,6	10	1,5	10	10	10	1,5	10
1	2,6	2,6	10	1,5	10	10	10	1,5	10
1	2,7	1,7	10	1,5	10	10	10	1,5	1,5
1	3,7	1,6	1,8	1,5	10	10	10	10	10
1	2,7	1,5	10	2,5	1,8	1,8	1,7	1,7	10
1	2,5	2,5	1,7	2,5	10	1,6	10	1,5	10
1	2,7	1,6	2,5	1,5	1,5	10	10	1,7	10
1	2,7	2,7	1,5	2,6	1,5	1,5	10	1,6	10
1	2,7	1,6	1,5	1,5	1,5	10	10	1,5	10
1	3,7	1,5	1,5	3,6	2,6	2,6	10	10	10
1	2,7	1,6	2,5	2,5	1,5	10	10	1,5	10
1	2,7	1,5	1,5	1,5	1,5	10	10	1,5	10
1	2,7	1,5	1,5	1,5	1,5	10	10	10	10
1	2,6	1,5	1,7	1,7	1,5	1,7	10	10	10
1	3,6	3,6	1,5	1,5	1,5	1,5	10	10	1,6
1	2,6	1,6	1,8	2,6	1,6	1,5	10	10	10
1	2,5	2,5	1,7	1,7	1,7	1,7	10	10	10
1	2,7	2,6	1,7	1,7	10	10	10	10	10
1	2,7	2,5	2,5	2,5	2,5	2,5	10	10	10
1	2,7	1,7	1,5	1,5	1,5	10	10	10	10

1	2,7	2,7	1,5	1,6	1,6	1,6	10	10	10
1	1,7	1,7	1,5	1,5	1,5	1,5	10	1,7	10
1	2,5	1,5	1,5	1,5	10	10	10	10	10
1	2,7	2,7	1,5	1,6	10	10	10	10	1,6
1	2,6	2,7	1,5	1,5	10	10	10	10	10
1	2,7	2,7	1,5	1,6	10	10	10	10	10
1	3,7	3,7	1,5	2,5	10	10	10	1,6	10
1	2,7	2,7	1,5	1,5	1,5	1,5	1,6	1,5	10
1	2,7	1,6	1,5	1,5	1,5	1,5	10	10	10
1	2,7	1,5	10	2,8	1,8	1,8	10	10	10
1	2,6	1,5	1,5	2,5	3,6	3,6	10	1,5	1,5
1	2,7	2,5	1,5	1,5	1,5	1,5	1,5	1,5	1,5
1	3,6	3,6	10	2,5	2,5	1,5	10	1,5	10
1	2,7	1,5	1,8	1,5	1,5	1,5	10	1,7	10
1	2,6	2,6	10	2,5	1,5	1,5	10	1,5	10
1	2,7	1,5	1,5	1,5	1,5	1,5	10	10	10
1	3,7	1,6	10	1,5	1,6	1,6	1,5	1,6	1,5
1	2,7	1,5	1,5	1,5	1,5	1,5	1,5	1,5	1,5
1	2,6	1,6	10	1,6	10	10	10	10	10
1	3,7	1,6	10	1,5	1,6	1,6	1,5	1,6	1,5
1	2,7	2,7	1,5	2,6	1,5	1,5	10	10	10
1	2,7	1,5	1,5	1,5	1,5	1,5	10	10	10
1	2,6	1,5	10	1,5	1,6	1,6	10	1,5	10
1	2,7	1,7	1,5	1,5	1,5	1,5	1,6	1,6	1,6
1	2,5	2,5	2,5	2,5	1,5	1,5	1,5	1,5	1,5
1	2,6	1,6	1,8	1,5	1,5	1,5	10	10	10
1	1,6	1,6	1,6	1,6	1,6	1,6	1,6	1,6	1,5
1	2,7	1,7	1,8	1,5	1,7	1,7	10	1,8	10
1	2,6	1,5	1,5	1,5	1,5	1,5	10	10	10
1	2,6	1,5	1,5	1,5	1,5	1,5	10	10	10
1	1,6	1,6	1,6	1,6	1,6	1,6	1,5	1,6	1,6
1	2,7	1,5	1,5	1,5	1,5	1,5	1,5	1,5	1,6
1	3,6	1,5	1,5	1,5	1,5	1,5	10	1,5	10
1	2,7	2,7	2,5	2,6	1,5	1,5	1,5	1,5	10
1	2,7	1,5	1,5	1,5	1,5	1,5	1,5	1,5	1,5
1	3,7	1,7	2,5	2,6	1,5	1,5	10	10	10
1	2,5	1,7	10	1,6	3,7	3,7	10	10	10
1	2,5	1,7	10	1,6	3,7	3,7	10	10	10
1	2,6	1,5	10	1,5	1,5	1,5	10	1,5	10
1	2,7	1,6	10	1,6	1,6	1,6	10	1,6	10
1	2,7	1,7	10	1,5	10	10	10	10	10
1	1,6	1,6	1,6	1,6	1,6	1,6	1,6	1,6	1,6
1	2,6	1,5	10	1,5	1,5	1,5	10	1,5	10
1	2,6	1,5	1,5	1,5	1,5	1,5	10	1,5	10
1	2,6	1,5	1,6	1,5	2,6	2,6	10	10	10
1	3,7	3,7	1,8	2,5	1,8	1,8	10	1,8	1,7
1	1,7	1,7	1,6	2,5	2,6	2,6	1,5	1,5	10

1	2,6	2,5	1,5	1,5	1,5	1,5	10	10	10
1	2,6	1,5	1,5	1,5	1,5	1,5	1,5	10	10
1	1,7	1,5	1,5	1,5	2,5	2,5	10	1,6	10
1	1,7	1,5	1,5	1,5	2,5	2,5	10	1,6	10
1	2,7	1,7	1,5	1,5	1,5	1,5	10	1,8	1,8
1	3,7	2,7	2,5	2,6	10	10	10	10	10
1	1,5	1,7	1,5	1,5	1,5	10	10	1,7	10
1	2,7	4,7	1,5	1,6	1,6	1,6	10	1,6	1,5
1	2,7	4,7	1,5	1,6	1,6	1,6	10	1,6	1,5
1	2,7	1,6	1,5	1,5	1,5	1,5	10	1,6	10
1	2,7	1,6	1,8	1,5	1,5	1,5	1,5	1,5	1,5
1	2,5	1,5	1,5	1,5	1,5	1,5	1,5	1,5	1,5
1	2,7	2,7	1,5	1,5	1,6	1,6	10	1,6	1,6
1	2,7	1,5	1,5	1,5	1,5	1,5	10	1,5	10
1	2,7	1,7	1,5	2,5	1,5	10	10	10	10
1	2,7	1,5	1,5	1,5	1,5	1,5	10	1,5	10
1	2,7	1,5	1,5	1,5	1,5	1,5	10	2,5	10
1	2,7	1,5	1,5	1,5	1,5	1,5	10	1,5	1,5
1	3,7	1,7	2,5	2,5	10	10	10	10	10
1	2,7	2,6	1,6	1,6	1,6	1,5	1,6	1,6	10
1	2,5	1,6	1,6	1,6	1,6	1,6	10	10	10
1	2,7	1,5	1,5	1,5	1,5	1,5	10	10	10
1	2,7	1,5	1,5	1,5	1,6	1,6	10	10	10
1	2,7	1,5	1,8	1,5	1,6	1,6	10	10	10
1	2,7	2,7	1,5	1,5	1,5	1,5	10	10	10
1	3,7	3,7	1,6	2,6	2,5	1,6	10	3,5	1,6
1	2,7	2,7	10	1,5	10	10	10	10	1,7
1	2,6	1,6	1,5	1,5	1,5	1,5	1,5	1,5	10
1	2,7	1,5	10	1,5	1,5	1,5	10	1,5	10
1	3,7	1,5	1,8	1,5	1,6	1,6	1,5	1,5	10
1	2,7	1,5	1,5	1,5	1,5	1,5	1,5	10	10
1	2,7	2,7	1,5	1,5	1,5	1,5	10	10	1,5
1	3,6	1,7	10	3,5	1,6	1,6	10	10	10
1	2,6	1,6	1,5	1,5	1,5	1,5	1,5	1,5	10
1	3,6	1,7	10	3,5	1,6	1,6	10	10	10
1	2,7	1,5	1,5	1,5	1,5	1,5	1,5	1,5	10
1	2,6	1,5	1,5	1,5	1,5	1,5	10	10	10
1	2,7	2,7	1,5	1,5	10	10	10	10	1,7
1	3,7	1,7	1,7	2,5	1,5	10	10	1,5	10
1	2,7	1,6	10	1,5	1,5	1,5	1,5	1,5	10
1	2,7	2,7	1,5	1,5	1,5	1,5	1,5	1,5	1,5
1	2,7	1,7	1,5	2,6	1,5	1,5	10	10	10
1	3,7	2,5	1,5	1,5	1,5	1,5	10	1,5	10
1	1,6	1,5	1,5	1,5	1,5	1,5	10	10	10
1	2,7	2,7	1,5	1,5	1,5	10	10	10	10
1	3,7	1,6	2,6	2,6	2,6	10	1,5	4,6	10
1	3,7	2,7	1,5	1,6	1,7	1,7	10	10	10

1	2,7	1,5	1,5	1,5	1,5	1,5	10	10	10
1	2,7	1,6	2,5	2,5	2,5	2,5	3,5	2,5	10
1	3,7	2,7	2,5	2,5	10	10	10	10	10
1	3,7	1,5	2,5	2,5	1,6	1,6	1,5	1,7	1,7
1	3,7	1,5	1,5	10	10	10	10	10	10
1	3,7	2,7	2,5	2,5	1,6	1,6	10	10	10
1	2,7	1,5	1,5	1,5	3,6	3,6	10	1,7	10
1	2,7	1,7	1,7	1,7	1,7	1,7	10	10	10
1	3,7	2,7	1,5	2,6	1,5	1,5	10	10	10
1	2,7	1,5	1,5	1,5	1,5	1,5	10	10	10
1	3,7	1,5	1,5	1,5	1,5	1,5	10	10	10
1	2,7	1,7	1,5	1,5	1,5	1,5	1,5	1,5	10
1	2,7	1,7	2,5	2,6	1,5	1,5	10	10	10
1	2,7	1,7	2,5	2,6	1,5	1,5	10	10	10
1	3,7	3,7	1,5	1,5	1,5	1,5	10	1,5	1,5
1	1,7	1,7	1,5	2,6	1,5	1,5	10	10	10
1	2,7	2,7	10	2,5	1,7	1,7	10	1,5	10
1	3,7	3,7	10	2,5	1,7	1,7	10	1,5	10
1	1,7	1,7	1,5	1,5	1,5	1,5	10	1,5	10
1	2,7	2,7	1,5	1,5	1,6	1,6	1,6	1,6	1,5
1	2,6	1,5	1,5	1,5	1,7	1,7	1,5	1,7	10
1	2,7	2,7	1,5	2,6	1,5	1,5	10	2,7	1,5
	2,7	1,7	1,5	2,5	1,5	10	10	10	10
1	2,7	10	10	1,5	10	10	10	10	10
1	2,7	1,5	10	1,5	10	10	10	10	10
1	2,7	1,6	10	1,5	1,6	1,6	10	1,5	10
1	2,7	1,5	10	1,5	10	10	10	10	10
1	2,7	1,6	10	2,5	2,5	2,5	10	1,5	10
1	2,7	1,7	1,8	1,5	1,5	1,5	10	10	10
1	2,6	1,7	1,5	1,6	1,5	1,5	1,6	10	10
1	2,6	1,6	1,5	1,5	1,5	1,5	1,5	1,5	1,5
1	2,7	1,5	1,5	1,5	1,5	1,5	10	10	10
1	2,6	1,5	10	1,5	1,5	1,5	10	1,5	10
1	2,7	1,5	1,5	1,5	1,5	1,5	1,5	1,5	1,5
1	1,6	1,6	1,6	1,6	1,5	1,5	10	1,5	1,6
1	2,7	1,7	1,5	2,6	1,5	1,5	10	10	10
1	1,6	1,7	1,5	2,6	2,5	2,5	1,5	1,5	10
1	1,6	1,5	1,5	1,6	1,6	1,6	1,5	1,5	1,6
1	2,7	1,7	1,8	1,6	1,5	1,5	10	1,5	10
1	2,6	1,6	1,5	1,5	1,5	1,5	10	1,5	1,5
1	2,5	1,5	1,5	1,5	1,5	1,5	10	10	10
1	2,6	1,5	10	1,5	10	10	10	1,8	10
1	2,6	1,5	10	1,5	10	10	10	1,8	10
1	2,6	1,6	1,5	1,6	1,5	1,5	1,6	10	10
1	1,6	1,5	10	1,5	1,5	1,5	1,5	1,6	10
1	3,7	1,5	1,5	2,6	1,5	1,5	10	10	10

Brown bre	Whole wh	White brea	Fat-cakes	Scones	Cakes	Muffins	Biscuits	chicken dr	chicken thi
1,5	10	10	1,5	2,8	1,8	2,7	1,5	1,6	1,6
1,5	10	10	1,5	2,8	1,8	2,7	1,5	1,6	1,6
1,7	10	1,7	1,5	1,5	1,5	1,8	1,8	1,6	1,8
10	10	10	1,6	1,5	1,9	10	1,5	1,5	10
1,7	10	1,8	1,5	2,6	1,8	10	1,5	10	1,5
1,7	10	10	10	1,8	10	10	10	1,5	1,5
10	10	2,7	1,5	10	1,8	10	1,5	1,5	1,5
1,5	1,5	10	10	1,8	1,5	1,8	1,6	10	10
2,7	10	2,7	2,7	2,7	1,8	1,8	2,7	2,6	2,6
1,7	10	10	1,8	10	1,9	10	1,7	10	10
1,6	10	10	10	1,5	1,8	1,8	1,5	2,7	10
1,5	10	1,5	1,5	1,5	1,8	1,8	1,5	1,5	1,5
2,6	10	2,5	1,6	2,5	1,8	1,8	2,5	1,5	1,5
1,7	10	10	1,8	10	1,8	10	1,7	1,5	10
2,7	10	2,5	1,5	1,5	1,8	1,8	1,6	1,5	1,5
1,5	10	10	1,9	2,8	1,8	10	1,5	10	10
1,5	10	10	1,8	1,8	1,8	10	1,5	10	10
2,6	10	1,5	1,5	1,5	1,5	1,5	1,5	1,5	1,5
2,6	10	2,5	1,5	1,5	1,8	1,8	1,5	1,5	1,5
1,5	10	1,5	1,5	1,5	1,8	1,8	1,6	1,5	1,5
2,6	10	1,5	1,6	1,8	1,8	1,8	1,5	1,5	1,6
2,6	10	1,5	1,5	1,5	1,5	1,8	1,5	1,5	1,5
2,6	10	2,5	1,5	1,5	1,8	1,8	1,6	1,5	1,5
1,6	10	1,5	1,6	2,5	1,8	1,8	1,5	10	10
1,6	10	1,5	1,5	1,5	1,8	1,8	1,5	1,5	1,5
1,6	10	1,5	1,6	1,5	1,8	1,8	1,5	1,5	1,5
1,6	10	10	1,5	1,6	1,9	10	1,6	10	10
1,7	10	1,5	1,8	1,5	1,8	10	1,6	1,6	1,6
10	10	2,7	1,6	1,5	1,5	10	1,5	1,6	1,6
1,5	10	10	1,5	1,7	1,7	10	1,6	10	10
2,6	10	2,6	1,5	10	10	10	1,8	10	10
2,6	10	10	1,5	1,5	1,8	1,5	1,6	10	10
2,6	10	2,6	1,6	2,5	1,8	10	1,6	1,6	1,6
1,5	10	10	10	10	1,8	10	10	1,5	10
1,5	10	1,5	1,5	1,5	1,8	1,8	1,6	10	10
1,6	10	10	10	10	1,9	10	10	10	10
1,6	10	10	10	10	1,8	10	1,7	1,5	1,5
1,7	10	10	1,6	10	10	10	1,6	10	1,7
2,6	10	2,6	1,5	1,5	1,8	1,8	1,7	1,5	1,5
1,7	10	10	1,5	10	1,5	1,5	1,5	2,6	10
1,7	10	10	1,5	10	1,8	10	1,7	1,6	1,6
1,5	10	1,5	1,5	10	1,5	10	1,5	1,6	10
1,6	10	1,6	1,5	10	1,9	10	1,5	10	10
1,5	10	1,5	10	1,6	1,8	1,5	1,8	1,6	10
1,7	10	10	10	1,8	1,8	10	1,8	1,5	1,5
10	10	10	10	10	10	10	1,5	1,5	10

1,6	10	10	10	10	10	10	1,6	1,5	10
1,5	10	10	1,7	10	10	10	10	1,7	10
2,6	10	2,6	10	10	2,8	10	2,5	1,5	1,5
1,6	10	1,6	1,5	1,5	1,5	1,5	1,5	1,5	1,5
1,5	10	10	10	1,8	1,8	10	1,7	1,6	10
1,6	10	10	1,5	1,5	1,9	10	10	10	10
10	10	1,7	1,6	10	1,8	1,8	1,5	1,6	1,6
2,7	10	1,5	10	1,5	1,9	10	1,5	10	10
2,7	10	10	10	1,5	1,9	10	10	10	1,6
2,7	10	1,5	1,6	1,5	1,9	1,5	1,5	1,5	1,5
1,6	10	10	10	2,6	1,9	1,5	1,5	1,6	1,6
2,7	10	10	10	1,8	1,9	10	10	10	1,6
2,7	10	1,6	1,5	1,5	1,5	1,5	1,5	10	10
1,6	10	1,5	1,5	1,5	1,5	1,5	1,5	2,6	2,6
1,7	10	10	1,5	10	1,8	10	1,8	1,5	1,5
1,7	10	10	10	1,8	1,8	10	10	1,5	10
1,6	10	1,5	10	1,8	2,9	10	2,9	1,5	10
1,6	10	10	1,6	1,5	1,8	10	2,7	10	2,7
1,6	1,5	1,5	1,5	1,5	10	10	1,5	1,6	1,6
2,6	10	1,5	1,8	2,5	2,8	10	1,7	10	1,6
2,6	10	2,6	1,6	2,5	1,8	1,8	2,6	1,5	1,5
2,6	10	2,5	1,5	1,5	1,8	1,8	2,6	10	10
2,6	10	2,5	1,5	1,5	1,8	1,8	2,6	10	10
1,5	10	1,5	1,5	1,8	1,8	10	1,5	1,6	1,6
1,5	10	1,5	1,5	1,8	1,8	10	1,5	1,6	1,6
1,7	10	1,7	10	2,7	3,9	10	10	10	10
2,7	10	10	10	10	10	10	10	1,6	10
1,5	10	10	1,8	1,8	1,8	1,8	1,8	1,5	1,5
1,6	10	1,6	1,8	10	1,8	10	2,6	2,6	2,6
1,6	10	10	10	10	10	10	10	1,6	1,6
1,6	10	10	1,5	10	10	10	10	1,5	1,5
1,6	10	10	1,5	10	10	10	10	1,5	1,5
1,6	10	1,6	1,5	10	1,8	1,5	1,5	1,6	1,6
1,5	10	10	1,5	10	2,8	10	1,7	10	10
1,7	10	1,5	1,5	1,8	3,8	2,8	2,6	1,5	1,5
2,6	10	10	1,5	1,5	1,8	10	1,5	2,5	2,5
1,5	10	1,5	1,5	1,5	1,8	10	2,6	2,5	2,5
1,6	10	10	10	1,8	1,8	10	1,5	1,5	1,5
10	10	2,6	1,5	10	1,9	10	10	1,5	10
1,5	1,5	1,5	1,6	1,5	1,8	1,5	1,5	1,5	10
1,6	10	1,5	1,8	1,5	1,8	1,8	1,8	1,5	1,5
1,6	1,5	1,6	1,5	1,5	1,8	1,8	1,5	1,6	1,6
1,6	10	1,6	1,5	1,8	1,8	1,8	1,6	1,6	1,6
1,6	10	1,6	1,8	1,5	1,8	1,8	1,6	2,6	2,6
1,6	10	1,6	1,5	1,8	1,8	1,8	1,5	2,6	2,6
1,6	10	1,6	1,5	1,8	1,8	1,8	1,6	1,5	1,5
1,7	10	10	1,5	2,5	1,8	1,8	2,5	1,5	1,5

1,6	10	10	1,7	10	1,8	10	10	1,5	1,5
1,7	10	10	10	10	1,8	10	10	2,6	2,6
1,7	10	1,5	10	10	10	10	1,7	1,6	1,6
1,7	10	10	10	10	1,9	10	2,8	1,6	1,6
1,6	10	10	1,5	1,8	1,8	1,5	1,5	1,5	1,5
1,5	10	10	1,6	1,5	1,8	10	1,5	10	10
2,6	10	1,5	1,5	1,5	1,8	1,5	1,6	1,5	1,5
1,6	10	1,5	1,5	1,8	1,8	1,8	1,6	10	10
1,7	10	1,4	1,5	10	1,8	10	1,5	1,5	1,5
3,7	10	1,7	3,5	1,8	1,8	1,8	1,8	2,5	10
1,6	10	10	1,6	10	1,9	10	10	10	10
1,5	10	10	1,5	10	1,8	10	10	1,5	1,5
1,6	10	10	10	3,6	10	10	1,7	2,7	2,7
2,7	10	2,7	1,5	1,5	1,5	1,5	2,5	2,6	2,5
1,6	10	10	1,6	10	1,9	10	10	10	10
1,6	10	1,5	1,6	1,5	1,8	1,8	1,5	1,5	1,5
1,5	10	1,5	1,8	1,8	10	10	10	1,5	1,5
1,5	10	10	1,5	10	10	10	10	1,6	1,6
1,5	10	1,5	1,5	1,5	1,8	1,8	1,7	1,5	10
1,7	10	10	10	1,8	1,8	10	10	1,5	1,5
1,7	10	1,6	1,6	1,5	1,8	1,5	1,5	1,5	1,5
1,5	10	10	1,5	10	1,8	1,8	1,8	1,7	1,7
1,6	10	1,6	1,5	1,5	1,5	1,5	1,7	1,5	1,5
1,5	10	1,6	10	1,6	1,5	1,5	1,7	1,5	1,5
1,6	10	1,6	1,5	1,5	1,5	1,5	1,7	1,5	1,5
1,6	10	1,6	1,5	10	1,8	10	10	3,6	10
1,6	10	10	10	10	10	10	1,6	1,6	1,6
1,6	10	1,6	1,5	1,5	1,8	10	10	10	10
1,6	10	10	1,5	10	1,8	10	10	1,5	1,5
2,6	10	2,5	1,5	1,5	1,5	1,5	1,5	1,5	1,5
1,7	10	10	1,5	1,8	1,8	10	10	1,5	1,5
2,7	1,5	1,5	1,5	1,5	10	10	1,7	2,6	2,6
2,6	10	2,5	1,5	1,5	1,8	1,5	1,5	1,5	1,5
1,5	10	10	10	1,8	1,8	1,8	1,6	1,5	1,5
1,6	1,6	1,6	1,5	1,8	1,8	10	1,6	10	10
1,5	10	1,5	1,5	1,8	1,8	1,8	2,6	10	10
1,6	10	1,6	1,7	1,8	1,8	1,8	1,6	2,5	10
1,5	10	10	10	1,8	10	10	3,7	1,5	1,5
1,5	10	1,5	1,5	1,8	1,8	1,8	1,5	10	2,6
1,7	10	1,7	1,5	1,8	1,8	10	1,6	2,5	10
1,5	10	10	10	1,8	1,8	10	1,8	1,5	1,5
1,6	10	1,5	1,5	1,5	1,8	1,5	1,5	1,5	1,5
1,6	10	10	1,5	1,5	1,9	10	10	10	1,5
1,6	1,5	1,6	1,5	2,7	1,8	2,8	1,5	1,5	1,5
2,6	10	10	1,5	1,5	1,8	1,5	2,6	1,5	1,5
1,7	10	1,7	1,5	1,6	1,8	1,8	1,5	1,5	1,5
2,6	10	2,5	1,5	1,5	1,8	1,5	1,5	1,5	1,5

1,7	10	10	1,5	1,5	1,8	10	10	1,5	1,5
10	10	1,6	10	2,8	1,8	10	1,6	1,5	10
10	10	10	1,8	1,8	1,8	1,8	1,5	2,5	2,5
10	10	10	1,8	1,8	1,8	1,8	1,5	2,6	2,6
1,6	1,8	1,5	1,5	1,5	1,9	10	1,7	1,5	1,5
1,5	10	1,6	1,5	1,5	1,8	1,8	1,6	2,5	2,5
2,5	10	2,5	1,6	2,5	1,8	1,8	1,5	1,5	1,5
1,6	10	1,6	1,5	1,5	1,8	1,8	1,6	1,6	1,6
1,7	10	1,7	1,6	1,5	1,8	10	1,6	10	10
1,6	10	1,6	1,5	1,5	10	10	10	1,5	10
1,5	10	10	1,8	1,8	1,8	1,8	1,8	1,5	1,5
1,5	10	10	1,8	1,8	1,8	1,8	1,8	1,5	1,5
1,7	10	10	1,8	1,8	1,8	10	1,7	1,5	1,5
1,6	10	10	1,5	1,5	1,8	1,8	1,5	10	2,6
1,7	10	1,7	1,6	1,6	1,8	1,8	1,6	2,6	2,6
1,7	10	1,7	1,6	1,5	1,8	1,8	1,6	10	10
1,7	10	1,7	1,6	1,6	1,5	10	1,5	1,5	10
1,7	10	1,7	1,6	1,6	1,5	10	1,5	1,5	10
2,6	10	2,5	1,6	1,5	1,8	1,5	2,5	1,5	1,5
1,6	10	10	1,5	1,5	1,5	1,5	1,5	1,5	10
1,6	10	1,6	10	3,8	1,8	1,8	1,7	10	10
1,7	10	1,7	1,5	1,8	1,8	1,8	1,8	1,6	1,6
1,7	10	1,7	1,5	1,8	1,8	1,8	1,8	1,6	1,6
10	10	1,8	1,5	10	1,8	1,8	1,8	2,6	2,6
10	10	1,8	1,5	10	1,8	1,8	1,8	2,6	2,6
1,6	1,6	1,6	1,7	1,5	1,8	1,8	1,6	1,5	1,5
1,6	10	10	1,5	1,5	1,8	1,5	1,5	1,5	1,5
2,7	10	10	2,8	1,5	1,9	10	10	1,5	1,5
1,7	10	10	1,5	10	1,8	10	1,6	1,5	1,5
2,6	10	2,5	1,5	1,5	1,8	1,5	1,5	10	10
10	10	1,7	1,5	10	1,8	10	1,5	10	10
1,6	10	10	10	10	1,8	10	1,6	1,5	1,5
1,7	10	10	1,6	1,8	1,8	1,5	1,5	10	10
2,6	10	2,6	1,5	1,5	1,8	1,5	1,5	1,5	1,5
1,6	10	10	1,8	1,5	1,8	1,8	1,5	1,5	10
1,6	10	1,6	10	1,8	1,8	10	1,6	1,5	1,5
10	10	1,6	1,6	10	1,8	1,8	1,6	1,5	1,5
1,6	10	10	10	10	1,8	10	1,5	1,5	1,5
1,5	10	1,5	1,5	1,5	1,8	1,8	2,7	1,5	1,5
1,5	10	1,5	1,5	1,5	1,8	1,8	3,7	1,5	1,5
1,6	10	10	10	1,8	1,8	10	1,5	1,5	1,5
1,7	1,5	10	10	2,5	1,8	1,8	1,5	2,6	2,6
2,7	10	2,7	10	2,6	1,8	1,8	1,8	10	1,5
1,6	10	1,5	1,5	1,5	1,8	1,8	1,5	10	10
1,6	10	10	1,5	1,5	1,8	10	10	1,5	1,5
1,6	10	10	1,5	1,8	1,8	10	1,6	10	10
1,5	10	1,5	1,6	10	1,8	10	1,6	10	1,5

1,6	10	10	1,5	10	1,8	10	1,5	1,6	10
10	10	1,6	1,5	1,8	1,8	10	1,6	1,5	1,5
1,7	10	10	1,6	1,6	1,8	10	1,5	1,5	1,5
1,5	10	1,5	1,5	1,5	1,8	1,5	1,5	1,5	1,5
1,5	10	1,5	1,5	1,5	1,8	1,5	1,5	1,5	1,5
1,6	10	10	1,6	10	1,8	10	1,5	1,5	1,5
1,7	10	10	1,7	1,8	1,8	10	1,8	1,5	1,5
1,6	10	10	1,6	1,4	1,8	10	1,6	2,6	10
1,5	10	10	1,5	1,5	1,8	1,6	1,5	1,6	10
10	10	1,5	2,6	1,5	1,9	1,8	1,8	1,5	1,5
1,6	10	10	1,6	1,5	1,5	10	3,7	1,5	10
1,7	10	1,7	1,5	1,5	1,8	1,8	1,7	10	10
1,6	10	10	1,5	1,8	1,8	10	10	1,5	1,5
1,6	10	10	1,6	1,8	1,8	10	10	1,5	1,5
1,7	10	1,7	1,5	1,5	1,8	1,8	1,7	1,5	1,5
1,6	10	10	1,6	10	1,7	1,7	1,5	1,5	1,5
1,5	10	1,5	10	1,5	1,8	1,8	10	15	1,5
1,7	10	10	1,7	1,5	1,8	10	1,6	1,5	1,5
10	10	1,6	1,6	1,6	1,8	10	1,5	1,5	1,5
1,6	10	10	1,5	1,8	1,9	1,5	10	1,5	1,5
1,6	10	1,6	1,5	1,5	1,8	1,8	1,5	10	10
1,5	10	10	1,6	1,5	1,5	1,5	10	1,5	10
1,5	10	10	1,6	1,5	1,5	1,5	10	1,5	10
10	10	1,5	1,5	1,8	1,8	10	10	10	10
1,5	10	1,5	2,5	1,5	1,8	10	1,5	10	10
2,6	10	2,6	1,6	1,5	1,8	1,8	1,6	10	1,6
2,5	10	2,5	1,6	1,5	1,8	1,8	2,6	2,5	2,5
1,8	10	10	1,6	10	1,8	10	10	1,5	1,5
1,7	10	1,5	1,5	1,8	1,8	10	10	10	1,5
10	2,7	10	1,5	1,8	1,9	1,8	1,8	1,5	1,5
1,6	10	1,5	1,6	1,5	1,8	10	1,5	1,5	1,5
1,6	10	10	1,7	1,5	1,8	1,8	10	1,6	10
1,6	1,5	1,5	1,5	1,5	1,8	1,8	1,5	1,5	1,5
1,7	10	10	1,5	1,8	1,8	1,8	1,5	2,5	2,5
1,6	10	10	1,5	1,8	1,8	10	1,5	1,5	1,5
10	10	1,6	1,6	1,8	1,8	10	10	1,5	1,5
1,6	10	1,5	1,7	1,8	1,8	1,8	1,5	10	10
2,7	10	1,6	1,5	1,8	1,9	1,8	1,8	10	1,5
1,7	10	10	10	10	1,8	10	10	1,5	1,5
1,7	10	1,7	1,6	1,8	1,8	1,8	1,5	1,5	1,5
1,5	10	1,5	1,8	1,8	1,8	1,8	1,5	2,5	2,5
1,6	10	10	1,5	1,8	1,8	10	10	1,5	1,5
10	10	1,5	1,6	10	1,9	10	10	10	1,5
10	10	10	1,5	10	1,8	10	10	10	10
10	10	1,5	1,6	10	1,9	10	10	10	1,5
1,6	10	10	1,5	1,8	1,8	10	1,6	1,8	1,6
2,6	10	2,5	1,5	1,5	1,8	1,8	1,5	1,5	1,5

2,6	10	2,5	1,5	1,5	1,8	1,8	1,5	1,5	1,5
10	10	1,6	1,8	1,8	10	10	1,6	1,5	1,5
1,6	10	1,6	1,5	1,8	1,8	1,8	1,6	2,6	2,6
1,6	10	10	1,6	10	1,8	1,5	1,5	1,5	1,5
10	10	1,6	1,5	1,8	1,8	10	1,6	1,5	1,5
1,5	10	10	1,5	1,8	1,8	10	1,6	1,5	1,5
10	10	1,6	1,5	10	10	10	1,6	1,5	1,5
10	10	10	1,5	1,5	1,8	10	1,6	1,5	1,5
10	10	1,6	1,5	10	1,6	10	1,6	1,5	1,5
10	10	1,6	10	1,8	1,8	1,8	1,6	1,5	1,5
1,5	10	1,5	1,5	1,5	1,5	10	10	1,5	10
2,7	10	10	1,5	10	10	10	1,5	1,5	1,5
1,7	10	10	1,7	10	1,8	10	1,5	1,5	10
1,8	10	10	1,7	10	1,8	10	1,5	2,7	10
1,7	10	10	10	1,8	1,8	1,8	1,6	10	2,5
1,6	10	1,6	1,6	10	1,8	10	10	1,5	10
1,6	10	1,5	1,7	1,5	1,8	1,8	2,5	10	10
1,6	10	1,5	1,7	1,5	1,8	1,8	2,5	10	10
1,6	10	10	1,6	1,8	1,8	1,8	1,5	10	10
2,6	10	10	1,5	1,5	10	1,5	1,5	1,6	1,6
1,6	10	10	10	10	1,8	10	10	10	10
1,7	10	10	1,5	1,8	1,8	1,8	1,6	1,5	10
1,6	10	10	1,5	1,5	1,9	1,8	1,8	1,6	1,6
1,5	10	10	1,6	1,8	1,8	1,8	2,5	2,5	2,5
1,5	10	10	1,6	1,8	1,8	1,8	2,5	2,5	2,5
1,7	10	10	1,5	1,8	1,8	1,8	1,8	1,5	1,5
1,7	10	10	10	1,8	1,8	1,8	1,6	10	2,5
1,7	10	10	1,6	10	1,8	1,8	1,4	1,6	1,6
2,7	10	10	1,6	1,6	1,8	10	1,6	1,5	3,5
1,7	10	10	1,6	1,8	1,8	1,8	1,6	2,5	10
1,6	1,8	1,5	1,7	1,9	1,8	1,8	1,7	2,5	2,5
1,7	10	10	1,5	1,8	1,8	10	1,9	2,5	10
1,7	10	10	10	1,8	1,8	10	1,7	1,5	1,5
1,5	10	10	1,7	1,5	1,8	1,8	1,8	1,6	1,6
1,6	10	1,5	1,5	1,5	1,8	1,8	1,5	2,5	1,5
1,5	10	10	10	1,8	1,8	10	10	10	2,5
1,6	10	1,5	1,5	1,8	1,8	1,8	1,6	1,5	1,5
1,6	10	10	1,5	1,8	1,8	10	1,6	1,5	1,5
2,6	10	10	1,5	1,5	1,6	1,8	1,6	1,6	10
1,5	10	1,5	1,5	10	10	10	1,5	1,6	1,6
1,5	10	1,5	1,6	1,8	1,8	1,8	2,5	10	10
1,7	10	10	10	10	1,8	1,8	1,6	1,5	1,5
1,6	10	10	1,5	1,8	1,8	10	1,6	10	2,5
1,6	10	10	1,5	1,8	1,8	10	10	10	2,5
1,6	10	1,6	1,5	1,5	1,8	1,8	1,5	10	10
1,5	10	10	1,6	10	1,8	10	1,5	1,6	1,5
1,4	10	1,4	10	1,4	1,8	1,8	1,8	1,6	1,5

1,6	10	1,6	1,6	1,5	1,8	1,8	1,6	10	10
1,5	10	10	10	1,5	1,8	1,8	1,8	1,7	1,5
1,7	10	1,5	2,6	1,7	1,8	1,8	1,5	1,5	1,5
1,6	10	10	1,5	1,5	1,8	1,8	1,6	2,6	2,6
3,7	2,5	2,5	2,5	1,8	1,8	1,8	1,5	1,5	1,5
1,7	10	10	1,6	1,8	10	10	1,8	10	10
1,7	10	10	1,5	1,5	1,8	1,5	1,5	1,5	1,6
1,5	10	1,5	10	10	1,8	2,8	1,5	1,6	1,5
2,6	1,6	1,6	10	1,5	1,8	1,8	1,6	1,6	1,6
2,6	1,6	1,6	10	1,5	1,8	1,8	1,6	1,6	1,6
1,5	10	1,5	10	1,7	1,8	1,5	2,7	1,7	1,5
1,5	1,5	1,5	1,5	1,5	1,5	1,8	1,5	1,5	1,5
1,7	10	10	1,6	1,8	10	10	1,8	10	10
1,6	1,5	1,6	1,6	1,5	1,8	1,8	1,6	1,5	1,6
1,6	1,8	10	1,8	1,8	1,8	1,8	1,6	2,6	2,6
1,7	10	1,7	10	1,7	1,8	1,8	1,5	1,6	2,6
1,6	10	1,5	1,6	1,5	1,8	1,8	1,6	2,6	2,6
1,7	10	1,6	1,5	10	10	10	1,6	2,7	10
1,6	1,5	1,6	10	1,6	1,6	1,8	1,8	1,6	1,6
1,6	10	10	1,5	1,8	1,8	1,8	1,5	1,5	1,5
1,6	10	10	1,5	1,5	1,5	1,5	1,5	2,5	10
2,7	10	10	10	10	1,9	10	1,5	10	1,6
2,7	10	1,6	1,5	1,8	1,8	10	10	1,6	10
1,7	10	1,6	1,5	1,8	1,8	10	1,8	10	1,6
1,6	10	1,6	1,5	1,5	1,5	1,5	1,5	1,5	1,5
1,5	10	1,5	1,5	1,8	1,8	10	1,5	10	10
1,7	10	1,6	1,5	1,5	1,8	10	1,5	1,6	1,6
1,5	10	1,6	1,5	1,8	1,8	1,8	1,8	1,8	1,5
1,7	10	1,8	10	1,8	1,8	10	10	1,6	1,6
1,6	10	1,6	1,5	10	10	10	1,5	10	10
1,5	10	10	1,5	1,5	1,5	1,5	1,5	1,5	1,5
1,5	1,5	1,5	1,5	1,8	1,8	1,8	1,5	10	10
1,5	10	10	1,5	1,5	1,8	1,5	1,5	1,5	1,5
1,6	10	10	10	1,5	10	1,8	1,5	1,5	1,5
1,7	10	1,8	1,5	1,5	1,5	10	1,5	10	1,6
1,5	10	10	10	1,5	1,8	1,8	1,5	10	1,5
1,7	10	10	1,5	1,6	1,5	10	1,7	1,5	10
1,7	1,8	1,5	1,5	1,8	1,9	1,8	1,8	10	10
1,7	10	1,8	1,5	1,5	1,9	10	1,8	1,6	1,6
1,7	10	1,5	1,5	1,5	1,9	1,8	1,8	1,6	1,6
1,5	1,8	1,8	1,5	1,8	1,5	1,5	1,5	10	10
1,5	10	1,5	1,5	1,8	1,8	1,8	1,5	10	2,5
1,6	10	10	1,5	1,5	1,5	1,8	1,5	1,5	10
1,5	10	1,5	1,5	1,5	1,5	1,5	1,5	1,5	1,5
1,6	10	1,7	10	10	10	10	1,5	1,5	1,5
1,6	10	10	1,8	1,8	1,8	10	1,7	10	1,6
1,7	10	10	1,8	1,8	1,8	1,8	1,6	2,6	2,6

1,7	10	10	1,8	1,8	1,8	1,8	1,6	2,6	2,6
1,6	10	1,6	1,5	1,5	1,8	1,8	1,5	1,5	1,5
1,5	10	1,5	1,5	1,5	1,8	1,8	1,5	1,5	1,5
1,5	10	1,5	10	1,6	1,8	2,8	1,8	2,6	1,6
1,7	10	1,7	1,7	1,7	1,8	10	1,5	1,5	1,5
1,5	10	1,5	1,5	1,5	1,5	1,5	1,5	1,5	10
1,7	10	10	1,8	1,8	1,8	1,8	1,6	2,6	2,6
1,7	10	10	1,8	1,8	1,8	1,8	1,6	2,6	2,6
1,5	1,8	1,8	1,8	1,5	1,8	1,8	1,5	10	1,5
2,6	1,5	2,5	10	10	2,8	10	3,8	2,6	2,7
1,7	10	1,5	1,6	1,5	1,8	1,6	1,6	10	1,6
1,6	10	10	1,5	10	1,8	10	10	10	10
1,6	10	1,5	1,7	1,5	1,8	1,8	1,5	1,6	1,6
1,6	10	10	1,9	1,9	1,9	1,9	1,5	2,6	1,6
1,6	10	10	1,8	1,8	1,8	1,8	1,6	2,6	2,6
1,6	10	10	1,8	1,8	1,8	1,8	1,8	10	10
1,6	10	10	1,8	1,8	1,8	1,8	1,6	10	10
1,6	10	10	1,8	1,8	1,8	1,8	1,6	2,6	2,6
1,7	10	1,7	1,7	1,7	1,8	10	1,5	1,5	1,5
1,6	10	1,6	1,5	1,8	1,8	1,8	1,5	1,5	1,5
1,5	10	1,5	10	1,5	1,8	2,5	2,8	1,6	1,5
1,7	10	10	1,5	1,5	1,8	10	1,6	10	10
1,6	10	10	1,8	1,8	1,8	1,8	1,6	2,6	2,6
3,6	1,5	10	2,5	1,5	1,8	1,8	1,5	1,6	1,5
1,6	10	10	1,5	1,8	1,8	1,8	1,6	2,6	2,6
2,7	10	1,6	1,5	1,5	1,8	1,6	1,6	10	1,6
1,6	1,8	1,5	1,8	1,8	1,8	1,8	1,6	1,6	2,6
1,6	10	10	1,8	1,8	1,8	1,8	1,6	1,6	2,6
1,6	10	10	1,8	1,8	1,8	1,8	1,6	2,6	2,6
1,5	10	1,5	1,8	1,8	1,8	1,8	1,6	1,6	2,6
1,7	10	1,8	10	2,8	10	10	10	10	10
1,7	10	10	10	10	1,8	1,8	10	1,6	1,6
1,5	10	10	10	10	1,5	10	1,5	10	2,6
2,7	10	2,7	1,6	1,6	1,6	1,9	1,8	10	1,6
2,7	10	10	10	10	1,9	10	10	10	1,6
1,6	10	1,6	1,5	1,8	1,8	1,8	1,5	1,5	1,5
1,6	10	1,5	3,6	1,8	10	1,8	1,8	10	10
1,6	10	1,5	1,6	1,5	1,8	1,8	1,5	1,5	1,5
1,6	10	1,6	1,5	1,8	1,8	1,8	1,5	1,5	1,5
1,6	10	1,6	1,6	1,8	1,8	1,8	1,6	1,5	1,5
2,6	1,5	1,5	1,8	10	2,8	2,8	1,8	10	1,6
1,7	10	1,6	10	1,8	1,8	10	1,7	2,6	3,6
1,6	1,6	1,6	3,6	10	1,8	2,8	2,8	1,7	1,6
1,5	10	10	1,5	1,5	1,5	1,5	1,5	10	1,5
1,6	10	10	10	10	10	10	10	10	10
1,5	10	10	1,5	1,8	1,8	1,8	1,6	1,5	10
1,5	1,5	1,5	1,5	1,5	1,5	1,5	1,5	10	10

1,7	10	10	1,5	1,5	1,8	10	10	10	1,6
1,7	10	1,8	1,5	1,8	10	10	1,6	10	1,6
1,5	1,5	1,5	1,5	1,5	1,5	10	1,5	1,6	10
1,6	10	1,5	1,5	1,5	10	10	1,5	10	10
2,7	10	1,5	1,6	1,5	10	10	1,6	10	10
1,6	10	1,5	1,6	1,6	10	10	1,6	10	10
1,6	10	1,6	1,5	1,8	1,8	1,8	1,7	3,6	3,6
1,6	1,5	1,5	1,5	1,5	1,8	1,8	1,5	2,6	2,6
1,6	10	1,6	1,5	1,5	1,8	1,8	1,6	10	10
1,5	10	1,5	1,5	1,5	1,5	1,8	1,8	1,6	1,5
1,6	10	1,6	1,5	1,5	1,8	1,5	1,5	1,5	10
1,5	10	1,5	1,5	1,5	1,5	1,5	1,5	1,5	10
3,6	10	10	10	1,8	1,8	10	1,7	2,6	2,6
1,7	10	1,5	1,6	10	10	10	10	10	10
3,6	10	10	10	1,8	1,8	10	1,7	2,6	2,6
1,6	10	1,5	1,5	1,8	1,8	1,8	1,6	10	10
1,6	10	1,6	1,5	1,5	1,8	1,5	1,6	1,6	10
1,5	1,5	1,5	1,5	1,7	1,7	1,7	1,5	10	10
1,7	10	1,7	1,5	10	10	10	1,7	1,5	1,5
1,6	10	1,6	1,5	1,5	1,8	1,5	1,6	1,6	10
1,7	19	1,5	1,5	1,5	1,9	1,5	1,5	2,5	10
1,5	1,5	1,5	1,5	1,5	1,5	1,5	1,5	1,5	10
1,6	10	1,6	1,5	1,5	1,8	1,8	1,6	10	2,6
1,7	10	10	1,8	1,8	1,8	1,8	1,6	2,6	2,6
1,5	1,5	1,5	1,5	1,5	1,5	1,5	1,5	10	10
1,5	10	1,5	1,6	1,8	1,8	1,8	1,6	10	1,6
1,5	1,5	1,5	1,6	1,6	1,8	1,8	1,8	1,5	1,6
1,6	10	1,6	1,8	1,5	1,8	1,8	1,7	2,6	1,6
1,6	10	1,6	1,5	1,5	1,8	1,8	1,6	10	1,6
1,6	10	1,6	1,5	1,5	1,8	1,8	1,6	10	1,6
1,5	1,5	1,5	1,6	1,6	1,8	1,5	1,6	1,6	1,6
1,7	1,5	1,8	1,8	1,8	1,8	1,8	1,6	2,6	2,6
1,6	10	1,6	1,6	10	10	10	1,6	1,6	1,6
1,7	1,8	1,8	1,5	1,5	1,8	1,5	1,5	2,6	1,6
1,5	1,5	10	1,5	1,5	1,5	1,5	1,5	1,5	10
1,7	10	1,6	1,5	1,5	1,9	1,5	1,5	2,6	10
1,7	10	1,7	1,8	1,6	1,8	1,8	1,7	1,6	1,5
1,7	10	1,7	1,8	1,6	1,8	1,8	1,7	1,6	1,5
1,6	1,5	1,5	1,5	1,5	1,8	1,8	1,5	10	10
1,6	10	1,6	1,8	1,8	1,8	1,8	1,5	1,5	1,5
1,7	10	10	1,5	10	1,8	10	10	1,5	1,5
1,5	1,5	1,5	1,6	1,6	1,8	1,6	1,6	10	1,6
1,6	1,5	1,5	1,5	1,5	1,8	1,8	1,5	10	10
1,6	10	1,6	1,5	1,5	1,8	1,8	1,6	10	2,6
1,6	10	10	2,6	10	10	10	2,5	1,5	1,6
1,6	1,8	1,6	1,8	2,6	1,8	1,8	2,5	1,6	1,6
1,5	10	10	1,5	1,5	1,8	1,8	1,5	10	10

1,6	10	1,5	1,5	1,5	1,7	1,5	1,5	1,5	1,5
1,6	10	10	10	1,5	1,8	1,5	1,5	1,5	1,5
1,7	10	1,5	1,6	1,8	1,8	1,8	1,5	1,5	1,5
1,7	10	1,6	1,6	1,8	1,8	1,8	1,5	1,5	1,5
1,7	1,5	1,5	1,6	1,5	1,8	1,8	1,5	1,5	1,5
2,7	10	1,5	1,5	1,5	1,5	1,5	1,5	1,5	1,5
1,5	10	10	1,8	1,8	1,8	1,8	1,8	10	10
1,6	1,5	1,6	1,5	1,5	1,5	1,5	1,5	1,5	1,6
1,6	1,5	1,5	1,5	1,5	1,5	1,5	1,5	1,5	1,6
1,7	10	1,5	1,5	1,5	10	1,8	1,6	10	10
1,5	10	1,6	1,5	1,5	1,8	1,8	1,5	1,5	1,5
1,5	10	10	10	1,9	10	10	10	10	10
1,6	1,5	1,5	1,5	1,5	1,8	1,8	1,5	2,6	2,6
1,6	10	1,6	1,6	1,5	1,5	1,5	1,5	1,5	1,5
2,7	10	2,5	1,6	1,6	1,8	10	10	10	2,7
1,6	10	10	10	1,7	1,5	1,5	1,5	10	1,5
1,5	10	1,5	1,5	1,5	1,8	1,8	1,5	10	10
1,5	10	10	10	1,5	1,8	1,5	1,5	1,5	10
2,7	10	1,6	1,5	1,5	1,8	1,5	1,5	10	2,6
1,5	10	1,5	1,6	10	10	10	10	10	10
1,6	10	10	10	1,5	10	10	1,5	1,5	1,5
1,6	10	10	1,5	1,8	1,8	1,8	1,8	1,5	1,5
1,5	10	10	1,5	1,5	1,6	1,6	1,6	10	1,5
1,5	10	1,5	2,5	1,7	1,8	1,8	1,6	1,6	1,5
1,6	10	10	1,5	10	1,8	1,8	1,5	2,6	2,6
1,8	1,6	1,5	1,5	1,5	1,8	1,8	1,8	1,7	1,5
1,5	10	1,5	1,8	1,8	1,8	1,8	1,5	2,6	2,6
1,6	10	10	1,5	1,8	1,8	1,8	1,8	1,5	10
1,6	10	1,6	1,5	1,5	10	10	10	1,5	1,5
2,6	10	2,6	1,8	1,8	1,8	1,8	2,6	1,5	1,5
1,7	10	1,5	1,5	1,8	1,8	10	1,5	1,5	1,5
1,6	1,5	1,5	1,5	1,5	1,5	1,5	1,5	1,6	1,6
1,7	10	1,7	3,5	2,5	10	10	1,5	1,6	1,5
1,6	10	1,6	1,5	1,8	1,8	1,8	1,5	1,5	1,5
1,7	10	1,7	3,5	2,5	10	19	1,5	1,6	1,5
1,6	10	1,5	1,5	1,5	1,8	1,8	1,5	1,5	1,5
1,6	10	10	1,8	1,8	1,8	1,8	1,5	1,5	10
1,5	10	1,5	10	1,5	1,8	1,8	1,5	1,6	1,6
1,7	10	1,7	1,8	1,8	1,8	10	1,7	10	3,6
1,5	10	1,5	1,5	1,5	1,8	1,5	1,5	1,5	1,5
1,6	1,5	1,5	1,5	1,5	1,8	1,8	1,6	10	2,6
2,7	10	2,5	1,6	1,6	1,8	1,6	1,6	10	2,6
1,7	10	10	1,5	1,7	10	10	10	10	10
1,6	10	1,6	1,6	1,5	1,5	1,5	1,5	1,5	1,5
2,7	10	2,6	10	10	2,8	10	10	10	10
10	3,6	1,6	1,5	1,5	1,5	1,5	2,6	1,5	1,5
1,7	10	1,6	1,6	10	10	10	10	10	1,6

1,6	10	1,5	1,6	1,8	1,8	10	1,5	1,5	1,5
1,5	10	1,5	2,6	3,5	1,8	2,5	1,5	1,5	10
2,7	10	10	10	10	2,8	10	10	10	2,7
1,5	10	1,5	2,5	1,5	1,8	1,8	1,5	1,6	1,5
1,5	10	10	1,5	1,5	1,8	2,8	2,8	2,5	10
2,7	10	1,5	1,6	1,6	1,6	1,6	1,6	10	10
1,6	10	1,6	10	10	10	10	1,6	1,5	10
1,5	10	10	1,5	1,5	1,8	1,5	1,5	1,5	1,5
2,7	10	1,6	1,7	10	10	10	10	10	10
1,6	10	1,6	1,5	1,5	1,8	1,5	1,5	1,5	10
1,6	10	1,6	1,5	1,8	1,8	1,5	1,6	1,5	10
1,6	10	1,6	1,5	1,5	1,8	1,8	1,6	1,5	1,5
2,7	10	2,6	1,6	1,5	1,8	1,8	1,5	10	1,7
2,7	10	1,5	1,6	1,6	1,8	1,8	1,5	10	1,7
1,6	10	10	1,5	1,5	1,8	1,8	1,6	3,6	3,6
10	10	1,7	1,5	10	10	10	10	10	10
1,7	10	10	1,5	10	10	10	1,6	3,6	3,6
1,7	10	10	1,5	10	10	10	1,6	3,6	3,6
1,5	10	10	1,5	1,5	1,8	1,8	1,6	2,6	2,6
1,6	1,5	10	1,5	1,5	1,8	1,8	1,7	2,6	2,6
1,6	10	10	1,8	1,8	1,8	1,8	1,5	10	1,5
1,7	10	1,5	1,5	1,5	1,5	1,5	1,5	1,5	10
1,7	10	10	10	2,8	1,8	1,8	1,5	1,5	10
1,7	10	10	1,6	1,5	1,9	1,8	1,6	1,5	1,6
1,6	10	10	1,6	10	10	10	1,5	1,5	1,5
1,7	10	10	10	1,8	1,8	10	1,6	1,6	10
1,6	10	10	1,6	10	10	10	1,5	1,5	1,5
1,5	1,5	10	1,6	1,8	1,8	1,8	1,5	1,5	1,5
1,5	10	10	1,5	1,5	1,5	1,5	1,5	1,6	10
1,6	10	1,6	1,5	1,5	1,8	1,8	1,5	1,5	1,5
1,6	10	1,6	1,8	1,8	1,8	1,8	1,6	2,6	2,6
1,5	10	10	10	1,5	1,5	1,5	1,5	1,5	10
1,7	10	10	1,6	1,8	1,8	1,8	1,6	1,6	1,5
1,5	10	10	10	1,5	1,8	10	1,5	10	1,5
1,5	10	1,5	1,5	2,6	1,8	1,5	1,6	1,5	1,6
1,7	1,8	1,8	1,5	1,8	1,8	1,8	1,8	10	10
1,7	1,5	10	1,5	1,8	1,8	1,8	1,7	1,5	1,5
1,6	10	1,6	1,6	1,6	1,8	1,6	1,6	1,6	1,6
10	10	1,5	1,6	1,8	1,8	10	1,5	10	10
1,6	1,8	1,6	1,8	1,8	1,8	1,8	1,6	2,6	2,6
1,5	10	10	10	1,5	1,5	1,5	1,5	1,5	1,5
10	10	10	1,8	1,8	1,8	10	1,6	1,5	1,5
10	10	10	1,7	1,8	1,8	10	1,6	1,5	1,5
1,6	10	1,6	1,5	1,5	1,8	1,8	1,5	1,5	1,5
10	10	10	1,6	1,8	1,8	10	1,6	1,5	10
1,7	10	1,8	1,5	1,5	1,8	1,8	1,8	2,5	1,5

chicken br	chicken wi	stewedchi	chicken he	chicken liv	chicken giz	chicken ne	chicken gib	stewed chi	beef stew
10	10	1,5	10	1,6	10	10	10	2,5	1,6
10	10	1,5	10	1,6	10	10	10	2,5	1,6
3,8	1,5	1,8	10	1,5	1,5	1,5	1,8	1,6	1,8
10	1,5	1,5	10	1,5	1,8	1,8	10	1,5	10
1,5	1,5	1,5	10	1,5	1,5	2,5	2,5	2,5	2,5
10	10	1,5	10	1,5	10	1,5	1,5	10	10
1,5	10	10	10	1,5	1,5	10	1,5	1,5	1,5
1,5	1,6	2,5	10	1,5	1,5	10	10	1,5	2,5
1,5	2,5	2,5	10	2,5	2,5	2,6	2,6	2,6	2,5
1,5	1,5	1,5	10	1,5	1,5	1,5	1,5	1,5	1,8
10	2,6	2,6	10	1,6	10	1,5	10	1,6	1,5
1,5	1,5	1,5	10	2,5	2,5	1,8	1,5	1,5	1,5
2,5	2,6	2,6	10	2,6	2,5	2,5	2,5	2,6	2,5
10	10	1,5	10	10	1,5	1,5	10	10	2,6
1,5	1,5	1,5	10	1,5	1,5	1,5	1,5	1,6	1,8
10	1,6	1,5	10	10	10	10	1,5	1,6	1,5
10	1,6	1,5	10	10	10	10	1,5	1,6	1,5
1,5	1,5	2,6	2,5	1,6	2,5	1,5	2,6	2,6	2,5
1,5	2,5	2,6	10	2,6	2,6	2,6	2,5	2,5	2,5
2,6	2,5	2,5	10	2,5	2,5	2,5	2,5	2,5	2,5
2,6	2,6	2,6	10	2,6	2,6	2,5	2,5	1,6	2,8
2,5	2,6	2,6	10	2,6	2,5	2,5	1,5	2,5	1,5
1,5	2,6	2,6	10	2,6	2,6	2,5	2,5	2,6	2,5
10	10	10	10	10	10	10	10	10	10
2,5	2,6	2,6	10	10	2,5	2,5	2,6	2,6	2,5
1,5	2,6	2,5	10	2,5	2,5	2,5	2,5	2,5	2,5
10	1,5	1,5	10	1,5	1,5	10	2,5	10	10
1,6	10	10	10	10	1,5	10	1,5	1,5	1,5
1,6	1,6	1,5	1,5	1,5	1,5	1,5	1,5	1,6	1,5
2,7	10	1,6	10	10	10	10	10	2,6	2,5
10	10	1,5	10	1,5	10	10	10	1,5	1,5
10	2,5	2,5	10	2,6	2,6	2,5	2,5	2,6	1,5
10	1,6	1,5	10	10	10	10	1,5	1,5	10
10	10	1,5	10	1,5	10	1,5	10	10	10
1,6	1,6	1,5	10	1,8	10	1,5	1,5	1,5	1,8
2,5	2,5	2,6	10	1,5	1,5	10	2,5	2,5	1,8
1,5	1,5	2,7	1,7	1,5	10	1,6	10	1,7	10
1,7	10	1,5	10	1,5	10	10	10	10	10
10	2,5	2,6	10	10	10	2,5	10	1,5	1,8
1,6	1,6	1,5	1,5	1,5	10	10	10	2,6	1,5
1,6	1,6	10	10	1,6	10	10	1,5	1,6	1,6
1,6	1,6	1,6	10	1,5	1,5	1,5	1,5	1,6	1,6
10	2,6	1,6	10	1,8	1,8	1,5	1,6	1,5	1,8
10	1,5	1,6	10	1,5	1,6	1,5	1,5	1,6	1,5
1,5	1,5	1,5	10	1,8	10	1,5	1,5	1,5	1,8
10	10	1,5	10	1,5	10	10	1,8	10	10

10	10	1,5	10	1,5	10	1,5	10	10	1,8
1,7	10	10	10	10	1,5	10	10	10	1,5
1,5	2,6	2,6	10	2,6	2,6	2,6	2,5	2,5	2,6
1,5	1,5	1,5	10	1,5	1,5	10	1,5	1,6	1,5
10	10	2,5	1,5	1,5	1,5	1,5	1,8	1,7	1,5
1,6	1,5	1,6	10	1,8	2,8	10	1,5	1,5	1,8
1,6	1,6	10	10	10	2,5	2,5	10	1,6	1,5
1,6	1,6	1,6	10	1,5	10	10	10	1,6	1,5
1,6	1,6	1,5	10	1,5	10	10	10	1,6	10
1,5	1,5	1,6	10	10	10	10	10	1,5	1,8
1,6	1,6	1,5	10	10	10	1,5	10	1,6	1,5
1,6	1,6	1,5	10	10	10	10	10	1,6	10
10	1,6	1,6	10	1,5	10	10	10	1,6	10
2,6	10	2,6	10	2,6	2,6	2,5	2,5	2,7	2,5
10	1,5	1,8	10	1,5	10	10	1,5	1,6	1,5
10	10	1,5	10	1,5	10	1,5	1,5	1,6	1,8
10	10	1,5	10	1,8	10	1,5	10	2,5	10
10	10	1,5	10	2,6	1,6	10	1,7	2,6	1,6
1,6	1,6	1,5	10	1,8	1,5	1,5	1,5	1,6	1,8
10	10	1,5	10	1,5	1,5	1,6	10	1,5	1,8
1,5	1,6	1,6	10	2,6	2,6	10	2,6	2,6	2,5
2,5	2,5	2,5	10	10	10	10	2,5	2,5	2,5
2,5	2,5	2,5	10	10	10	10	2,5	2,5	2,5
1,6	1,6	1,5	10	10	1,8	10	1,5	1,5	1,8
1,6	1,6	1,5	10	10	1,8	10	1,5	1,8	1,8
10	1,6	1,5	10	10	10	10	10	1,6	1,8
10	1,5	1,5	10	1,5	10	10	1,5	1,5	1,5
10	10	1,5	10	1,8	10	10	1,8	1,8	1,8
2,6	1,5	10	10	2,6	1,5	10	10	2,6	1,5
2,7	2,7	10	10	10	10	10	10	3,6	1,5
1,5	1,5	3,5	10	2,5	2,5	2,5	2,5	10	10
1,5	1,5	3,5	10	2,5	2,5	2,5	2,5	10	10
1,6	1,6	1,6	1,6	1,6	1,6	1,6	1,6	1,5	1,5
10	1,5	1,6	10	1,6	1,5	1,7	1,7	2,6	1,6
1,5	1,5	1,5	10	1,5	1,5	1,5	1,5	1,5	1,5
2,5	2,6	2,6	1,5	2,6	2,5	2,5	1,5	2,5	1,8
2,5	2,5	10	10	2,6	2,5	10	2,5	2,5	2,5
10	10	1,5	10	10	10	10	1,5	10	1,5
10	1,5	2,6	10	2,8	1,5	10	10	1,5	10
1,6	1,6	2,6	10	2,6	3,6	10	1,5	2,7	2,5
1,5	1,5	1,5	10	1,5	1,8	1,8	1,8	1,5	1,8
1,6	1,6	1,5	10	1,5	1,5	1,5	2,5	2,6	1,8
1,6	1,6	1,5	10	1,5	1,5	1,5	1,5	2,7	1,5
2,6	2,6	1,5	10	1,5	10	1,5	1,5	2,6	2,6
2,6	2,6	1,5	10	1,5	1,5	1,5	1,5	2,5	1,8
1,5	1,5	1,5	10	1,5	10	1,5	1,5	1,5	1,5
1,5	2,6	2,6	10	2,5	1,5	1,5	1,5	1,5	2,8

10	10	1,6	10	1,5	10	10	1,6	10	1,8
10	1,6	3,5	10	10	10	1,5	10	10	1,5
10	10	1,7	10	1,5	2,6	2,6	10	2,6	2,6
1,6	10	1,5	10	1,8	10	10	10	1,6	1,8
1,5	1,5	1,5	10	1,5	10	1,5	1,5	1,5	1,8
1,5	1,5	1,6	10	1,5	10	1,5	1,5	1,6	10
2,5	2,6	2,6	10	2,5	2,5	2,5	2,5	2,6	2,6
10	1,5	1,5	10	1,5	1,5	1,5	1,5	1,5	1,5
1,5	1,5	1,5	1,5	1,5	1,5	1,5	1,5	1,5	1,5
1,6	2,5	2,5	1,5	2,5	2,5	2,5	2,5	1,5	1,5
1,5	1,5	1,6	10	1,5	10	10	10	1,5	1,5
10	10	1,5	10	1,5	10	1,5	10	10	1,5
10	2,7	2,7	10	2,7	2,7	2,7	2,7	2,7	2,7
2,7	2,6	3,5	10	1,5	1,5	1,5	1,5	2,6	2,5
1,5	1,5	1,6	10	1,5	10	10	10	1,5	1,5
1,5	1,5	1,6	10	1,6	1,5	10	1,5	1,5	1,8
2,5	1,5	2,6	1,6	1,5	10	1,5	10	2,5	1,8
10	1,6	1,5	10	1,5	10	1,5	1,5	1,5	1,5
10	10	3,6	10	1,5	10	10	1,5	1,5	1,5
10	10	1,5	10	1,5	1,5	10	1,5	10	1,5
1,5	1,5	1,5	10	1,6	1,6	10	1,6	1,6	1,5
10	1,7	1,5	10	1,7	10	1,7	1,7	1,7	1,7
1,5	10	1,5	10	10	10	10	1,6	1,5	1,5
1,5	10	1,5	10	10	10	10	10	1,6	1,5
1,5	10	1,5	10	10	10	10	10	1,6	1,5
3,6	3,6	3,5	3,5	3,6	10	1,5	10	2,5	2,5
1,5	1,5	2,6	1,5	1,5	1,5	1,6	1,5	2,5	1,5
10	10	1,5	10	10	10	10	10	1,6	10
10	10	10	10	1,5	10	1,5	10	10	1,8
1,5	1,5	1,5	10	2,6	2,6	1,5	2,6	1,5	2,6
1,5	1,5	2,6	10	2,9	10	10	10	1,6	1,6
2,6	2,6	1,5	10	1,5	1,5	10	1,5	1,5	1,5
1,5	1,5	1,6	10	1,6	1,5	1,6	1,5	1,5	2,5
1,5	1,5	1,5	1,6	1,7	1,8	1,8	10	1,5	1,5
10	1,6	1,7	1,7	1,6	1,7	1,7	10	1,5	1,5
1,5	1,5	1,7	1,7	2,5	1,5	1,5	1,5	1,6	10
10	2,5	1,8	1,8	1,5	1,8	1,8	1,8	1,6	1,6
1,5	1,6	10	10	2,5	10	10	10	2,5	2,5
10	2,6	1,5	10	1,5	1,5	10	10	1,5	1,5
10	10	1,5	10	1,6	1,5	1,5	10	1,6	1,6
1,5	1,5	1,5	10	1,5	10	10	1,5	1,5	1,5
10	1,5	1,5	10	1,5	10	1,5	1,5	1,5	1,5
1,5	1,5	10	10	1,8	10	1,5	10	1,5	1,5
10	1,6	1,5	10	1,5	1,5	1,5	1,5	2,5	1,6
1,5	1,6	2,6	10	2,6	2,6	10	2,6	2,6	2,5
1,5	1,5	1,5	10	1,5	1,5	1,5	1,6	1,6	1,5
1,5	2,6	10	10	2,6	2,5	2,5	2,6	2,6	2,8

10	10	10	10	1,5	1,5	10	10	10	1,8
1,5	2,6	2,5	10	10	2,6	10	10	2,5	2,5
10	2,5	2,5	10	2,5	10	10	10	2,5	10
10	2,6	2,6	10	2,6	10	10	10	2,6	10
1,5	1,5	10	10	1,8	1,8	10	1,8	1,5	1,8
2,5	2,5	2,5	10	2,5	2,5	2,5	1,5	1,5	1,5
1,5	1,5	2,6	2,6	2,6	2,6	2,6	1,5	1,6	2,5
1,6	10	1,5	10	1,5	1,5	10	1,5	1,6	1,6
10	1,5	2,5	10	10	10	10	10	10	10
10	1,5	1,5	10	1,5	10	10	1,5	1,5	1,5
3,7	3,7	3,7	10	3,7	10	10	3,5	2,5	2,5
3,7	3,7	3,7	10	3,7	10	10	3,5	2,5	2,5
10	10	1,5	10	1,5	10	10	1,5	10	1,8
2,6	2,6	2,6	10	1,5	1,5	3,5	2,5	2,6	2,6
1,5	1,6	1,5	10	2,5	2,5	1,5	1,5	2,6	1,5
2,5	2,5	10	10	1,6	1,5	10	1,5	2,6	1,6
10	2,6	1,6	10	10	10	10	10	2,7	1,6
10	2,6	1,6	10	10	10	10	10	2,7	1,6
1,5	2,6	2,6	10	2,6	10	2,8	2,5	2,5	2,8
10	10	1,5	10	1,5	10	1,5	10	1,6	1,7
1,6	1,5	10	10	2,5	10	10	10	2,5	2,5
1,6	1,6	1,5	10	10	1,5	1,5	1,5	10	1,5
1,6	1,6	1,5	10	10	1,5	1,5	1,5	10	1,5
2,6	2,6	1,5	10	2,6	2,5	1,8	2,5	10	10
2,6	2,6	1,5	10	2,6	2,8	1,8	2,5	10	10
10	1,5	1,5	10	1,5	1,6	1,5	1,5	1,5	1,5
10	1,5	1,5	10	10	1,5	10	1,5	1,5	1,5
1,5	10	1,5	10	1,5	10	10	10	1,5	1,5
10	10	1,5	10	1,5	10	10	1,5	10	1,8
2,5	2,5	10	2,5	2,6	2,5	10	2,5	2,6	2,5
1,5	10	1,5	10	2,5	1,5	2,5	10	10	10
10	10	1,5	10	1,5	10	1,5	1,6	1,6	1,8
10	1,6	1,6	10	1,5	10	10	10	1,5	1,5
1,5	1,5	2,6	2,5	2,6	2,6	2,6	2,6	2,5	2,6
10	10	1,5	10	1,5	1,5	1,5	1,6	1,5	1,5
10	10	1,5	10	1,5	10	1,5	1,5	1,6	1,8
10	10	1,5	10	10	1,5	10	10	1,5	10
10	10	1,5	10	10	10	1,5	1,5	10	1,8
1,5	1,5	2,5	1,5	1,5	2,5	10	2,5	2,5	1,5
1,5	1,5	2,5	1,5	1,5	2,5	10	2,5	2,5	10
10	10	1,5	10	1,5	10	10	10	10	1,5
10	2,6	2,5	10	1,5	1,5	2,5	1,5	10	1,5
1,5	1,5	1,5	10	1,8	1,8	1,6	10	1,6	1,8
10	1,5	1,5	10	1,5	10	10	1,5	1,6	1,5
10	1,5	10	10	1,5	10	10	1,5	1,5	1,8
2,5	10	10	10	1,5	1,5	10	10	10	1,5
1,5	1,5	1,6	10	1,5	10	1,5	10	1,5	1,8

10	1,6	1,5	10	1,5	1,5	1,5	1,5	2,7	2,7
10	10	1,5	10	1,5	10	10	1,5	10	1,8
10	10	1,5	10	1,5	10	10	1,5	10	1,8
1,5	1,5	2,5	10	2,6	2,5	2,5	2,6	2,5	2,5
1,5	1,5	2,5	10	2,6	2,5	2,5	2,6	2,5	2,5
10	10	1,5	10	1,5	10	10	10	10	1,8
10	10	1,5	10	1,5	10	10	1,5	1,6	10
10	2,6	10	10	10	10	10	10	3,5	2,5
10	1,6	1,5	10	1,5	2,7	10	10	1,5	1,5
1,5	1,5	1,6	10	1,8	1,8	1,5	1,8	1,5	1,8
1,6	10	1,5	10	2,5	10	1,5	10	2,6	1,5
2,6	2,6	1,5	10	10	10	10	1,5	10	1,8
10	10	1,5	10	1,5	10	10	1,5	1,6	10
10	10	1,5	10	1,5	10	1,5	1,5	10	1,8
10	1,5	1,5	10	1,5	1,5	1,5	1,5	1,5	1,6
10	10	1,5	10	1,5	10	1,6	10	1,6	10
10	1,5	1,5	10	1,5	1,5	1,5	1,5	1,5	10
10	10	1,5	10	1,5	10	10	1,5	10	10
10	10	1,5	10	1,5	10	1,5	10	1,5	1,8
1,5	1,5	1,6	10	1,5	1,5	1,5	10	1,5	1,8
10	1,5	1,5	10	1,5	10	10	10	1,5	1,5
1,5	1,5	1,5	10	10	10	1,5	1,6	1,5	1,5
1,5	1,5	1,5	10	10	10	1,5	1,6	1,5	1,5
3,6	10	3,5	10	10	2,5	10	10	10	10
1,5	1,5	1,5	10	1,5	1,5	1,5	2,5	1,5	2,5
10	1,5	1,5	10	1,5	1,5	1,5	1,5	1,6	1,6
10	2,6	10	10	2,6	10	2,6	2,6	2,5	1,8
10	10	1,5	10	1,5	10	1,5	1,5	10	10
10	10	2,5	10	2,5	2,5	10	10	3,6	2,8
1,5	1,5	1,6	10	1,8	1,8	1,5	1,8	1,5	1,8
1,5	1,5	1,5	10	2,5	1,8	1,5	2,5	10	1,5
10	10	1,5	1,5	1,5	1,5	10	10	1,6	1,6
1,5	1,5	1,5	1,5	1,5	1,5	1,5	1,5	1,5	1,5
2,5	2,5	1,5	10	10	10	10	1,5	2,5	10
10	10	10	10	1,5	10	10	1,5	1,6	1,8
10	10	10	10	1,5	1,5	10	1,5	1,5	1,8
1,5	1,5	1,6	10	10	10	10	10	1,5	1,5
1,5	1,5	1,6	10	2,5	1,8	1,5	10	1,5	1,8
10	10	1,5	10	1,5	10	10	10	1,5	1,8
1,5	1,5	1,5	10	1,5	1,5	1,5	1,5	1,5	2,5
2,5	10	10	10	10	1,5	10	1,8	10	2,5
1,5	1,5	1,5	1,5	1,6	1,6	1,6	10	10	1,5
1,5	1,5	1,7	10	10	10	1,5	10	1,5	1,5
1,5	10	2,5	10	1,5	10	10	10	10	10
1,5	1,5	1,6	10	10	10	1,5	10	1,5	1,8
1,6	1,6	1,6	1,5	1,5	1,5	1,5	10	1,6	1,6
1,5	10	10	10	2,6	10	10	1,5	1,5	2,5

1,5	10	10	10	2,6	10	10	1,5	1,5	2,5
10	10	1,5	10	1,5	10	10	1,5	1,6	1,8
2,6	2,6	1,5	10	1,5	1,5	1,5	1,5	2,6	1,8
10	10	10	10	1,5	10	10	10	1,6	1,8
10	10	10	10	1,5	1,5	10	1,5	1,6	10
10	10	1,6	10	1,5	10	10	1,5	10	1,8
10	10	1,5	10	1,5	10	1,5	1,5	1,5	1,8
10	10	1,5	10	1,5	10	1,5	1,5	1,6	1,8
10	10	1,5	1,5	1,5	1,5	1,5	1,5	1,5	1,5
10	10	1,5	10	1,5	10	10	1,5	1,6	1,8
2,6	1,5	1,5	10	2,6	10	10	1,5	1,5	10
10	1,5	1,5	10	1,5	10	1,5	1,5	1,5	10
10	1,5	2,5	10	2,5	2,5	2,5	1,5	2,5	2,5
10	1,5	2,5	10	2,5	2,5	2,5	1,5	2,5	2,5
10	10	2,5	2,5	2,5	10	10	1,5	1,8	10
10	10	1,5	10	1,5	10	10	10	1,5	1,5
1,5	2,5	2,6	10	2,5	2,5	2,5	2,5	2,5	2,8
1,5	2,5	2,5	10	2,5	2,5	2,5	2,5	2,5	10
1,6	10	1,5	10	1,5	1,5	1,5	10	2,5	10
1,6	1,6	1,5	2,5	1,5	10	1,5	10	1,6	1,5
2,5	10	2,5	10	10	10	10	10	10	10
10	10	1,8	10	1,5	1,5	1,5	10	1,8	1,8
1,6	1,6	1,5	10	10	10	10	10	1,6	1,9
2,5	1,5	1,5	2,5	1,5	1,5	1,5	10	2,8	10
2,5	1,5	1,5	2,5	1,5	1,5	1,5	10	2,8	10
10	1,5	1,5	10	1,5	1,5	10	1,5	1,5	1,5
10	10	2,5	10	2,5	2,5	10	10	1,5	1,8
1,6	1,6	3,5	10	10	10	3,5	3,5	10	2,8
10	10	2,5	10	10	10	3,5	10	1,5	1,8
2,5	10	1,5	10	2,5	10	10	10	2,5	2,8
10	1,5	1,5	10	2,5	1,5	1,5	10	1,5	1,5
2,5	10	1,5	10	1,5	1,5	10	10	10	10
10	10	1,5	10	1,5	1,5	10	10	1,5	1,6
1,6	10	1,6	10	1,5	10	10	1,5	1,6	10
10	2,5	2,5	10	2,5	2,5	2,5	2,5	2,5	1,5
10	10	1,5	10	10	1,5	1,5	10	1,5	1,8
10	1,5	1,5	10	1,8	1,8	2,8	10	1,5	1,8
10	10	1,6	10	1,5	10	1,5	1,5	1,6	1,8
10	1,5	1,5	10	1,5	10	10	10	1,6	1,5
1,6	1,6	2,5	10	1,5	10	10	1,5	1,5	1,8
2,5	10	10	10	2,8	10	10	10	10	10
1,5	1,5	1,5	10	1,5	1,5	10	10	1,5	1,8
2,5	2,5	1,5	10	10	10	10	10	1,6	1,8
2,5	2,5	1,5	10	10	10	10	10	1,6	1,8
1,6	10	1,6	10	1,5	1,5	1,5	1,5	1,5	1,6
1,5	1,5	2,5	10	1,5	1,5	1,5	1,6	1,5	1,5
1,5	10	1,5	10	1,5	1,5	2,6	10	1,6	1,5

1,6	10	1,5	10	1,5	1,5	1,6	1,5	1,5	1,5
10	1,5	1,6	10	1,5	1,5	1,5	10	1,4	1,5
1,5	1,5	1,5	10	1,5	1,5	1,5	1,5	1,5	1,5
2,6	2,6	1,5	10	1,5	1,5	1,5	1,5	1,6	2,5
1,5	1,5	1,5	10	1,5	1,5	1,5	1,5	1,5	1,5
10	2,6	10	10	10	10	10	2,6	1,5	1,8
10	1,6	1,6	10	1,6	1,6	1,6	10	1,6	10
1,6	1,6	2,6	10	1,6	1,5	1,5	2,5	1,6	1,5
1,5	1,5	1,6	10	1,6	2,7	2,7	2,7	2,7	1,6
1,5	1,5	1,6	10	1,6	2,7	2,7	2,7	2,7	1,6
1,6	1,5	1,5	10	2,6	1,5	10	10	1,5	2,5
10	1,5	1,5	1,5	1,5	1,5	10	10	1,5	1,5
10	2,6	10	10	10	10	10	2,6	1,5	1,8
1,6	1,6	1,5	10	1,5	1,6	1,5	10	1,5	1,5
2,6	2,6	1,5	10	1,5	1,5	1,5	1,5	1,6	1,5
1,6	1,6	2,5	10	1,5	10	2,5	2,5	2,5	1,5
2,6	2,6	2,5	10	1,5	1,5	1,5	10	1,6	1,5
10	10	2,6	10	10	10	10	10	2,6	1,8
1,6	1,6	1,6	10	1,6	1,6	1,6	1,6	1,6	1,6
10	1,5	1,5	10	1,5	1,5	1,5	1,5	1,5	1,5
10	10	2,5	10	10	10	10	10	2,5	2,5
1,6	1,6	1,5	10	1,5	10	10	10	1,6	1,5
10	1,6	1,6	10	1,6	1,6	1,5	10	1,6	1,5
1,6	1,6	1,6	10	1,5	1,5	1,6	1,5	1,6	1,5
1,5	1,5	1,5	10	1,5	1,5	1,5	1,5	1,5	1,5
10	1,5	1,5	10	1,5	10	1,5	1,5	1,5	1,5
1,6	1,6	1,5	10	1,5	1,5	1,6	10	1,5	1,5
10	1,5	1,5	10	1,5	1,5	1,5	1,5	1,5	1,5
1,6	1,6	1,5	10	1,5	1,5	1,5	10	1,6	1,8
10	1,5	1,5	10	1,5	10	1,5	1,5	1,5	10
1,5	1,5	1,5	1,5	1,5	1,5	1,5	1,5	1,5	1,5
10	1,5	1,5	10	10	10	10	10	1,5	1,5
10	10	1,5	1,5	1,5	1,5	1,5	1,5	1,5	1,5
10	1,5	1,5	10	1,5	1,5	1,5	1,5	1,5	1,5
1,6	1,6	1,7	10	1,5	1,5	1,5	10	1,6	1,5
1,5	1,5	1,5	1,5	1,5	1,5	10	10	1,5	1,5
10	1,5	1,5	10	10	10	1,5	1,5	1,5	1,5
10	1,6	1,6	10	1,5	1,5	10	10	1,6	10
1,6	1,6	1,5	10	1,5	1,8	1,5	10	1,6	10
1,6	1,6	1,5	10	1,5	1,5	1,6	1,5	1,6	1,6
10	1,5	1,5	1,5	1,5	1,5	1,5	1,5	1,5	1,5
10	2,5	2,5	10	1,5	1,5	10	10	1,5	1,5
1,5	10	1,5	10	1,5	10	1,5	1,5	1,5	1,5
10	10	1,5	1,5	1,5	1,5	10	10	1,5	1,5
1,5	1,5	1,5	1,5	1,5	1,5	1,5	1,5	1,5	10
1,6	1,6	1,5	10	1,5	1,5	1,8	1,5	1,6	2,5
2,6	2,6	1,6	10	1,5	1,5	1,5	1,5	2,6	1,5

2,6	2,6	1,5	10	1,5	1,5	1,5	1,5	1,6	1,8
1,5	1,5	1,5	1,5	1,5	10	1,5	1,5	1,5	1,5
10	1,5	1,5	10	1,5	1,5	1,5	1,5	1,5	1,5
2,5	1,6	2,6	10	1,5	10	2,6	10	2,5	1,6
1,5	1,5	1,6	10	1,7	1,6	1,5	1,6	1,6	1,7
10	10	10	10	1,5	1,5	1,5	1,5	1,5	1,5
2,6	2,6	1,6	10	1,5	1,5	1,5	1,5	2,6	1,8
2,6	2,6	1,5	10	1,5	1,5	1,5	1,5	2,6	1,5
10	10	1,5	10	10	10	10	10	1,8	1,8
10	2,6	2,6	10	1,6	2,6	2,5	10	2,6	2,6
1,6	1,6	1,6	10	10	10	1,5	10	1,5	1,8
10	10	1,5	10	1,5	1,6	1,6	1,6	1,6	1,7
1,6	1,6	1,5	10	1,5	10	1,5	1,5	1,6	1,5
1,6	1,6	1,7	10	1,9	1,9	1,9	1,9	1,9	1,9
2,6	2,6	1,5	10	1,5	1,5	1,5	1,5	1,6	1,8
1,5	1,5	1,6	10	1,5	10	1,5	1,5	1,5	1,8
2,6	2,6	1,5	10	1,5	10	1,5	1,5	1,5	1,8
2,6	2,6	1,5	10	1,5	1,5	1,5	1,5	1,6	1,8
1,5	1,5	1,6	10	1,7	1,6	1,5	1,6	1,6	1,7
1,5	1,5	1,5	1,5	1,5	10	10	1,5	1,5	1,5
1,5	1,5	1,5	10	1,5	10	2,5	10	1,5	1,5
10	2,6	2,6	10	1,5	1,5	10	1,5	1,5	1,5
2,6	2,6	1,5	10	1,5	1,5	1,5	1,5	1,6	1,8
1,5	1,5	1,5	10	1,5	1,5	10	1,5	1,5	1,5
2,6	2,6	1,5	1,5	1,5	1,5	1,5	1,5	2,6	1,8
2,6	2,6	2,6	10	1,5	1,5	1,5	1,5	1,5	1,5
2,6	2,6	1,5	10	1,5	1,5	1,5	1,5	1,5	1,8
2,6	2,6	1,5	10	1,5	1,5	1,5	1,5	1,6	1,5
2,6	2,6	1,5	10	1,5	1,5	1,5	1,5	1,6	1,8
2,6	2,6	1,5	10	1,5	1,5	1,5	1,5	2,6	2,8
2,6	10	1,8	10	10	10	10	10	1,8	1,5
1,6	1,6	1,6	10	1,5	10	2,5	1,5	2,6	2,8
10	2,6	10	10	1,5	10	10	10	10	10
1,6	1,6	1,6	10	1,6	1,6	1,6	1,5	1,6	1,5
1,6	10	1,6	10	1,5	1,5	10	10	1,5	1,5
1,5	1,5	1,5	1,5	1,5	1,5	1,5	1,5	1,5	1,5
1,8	10	3,6	2,6	1,8	10	1,8	1,6	3,6	10
1,5	1,5	1,5	10	1,5	1,5	1,5	1,5	1,5	1,5
1,5	1,5	1,5	1,5	1,5	1,5	1,5	1,5	1,5	1,5
1,5	1,5	1,5	1,5	1,5	1,5	1,5	1,5	1,5	1,5
10	1,6	2,6	1,5	10	10	10	10	2,5	10
3,6	3,6	1,5	10	2,6	2,6	2,5	10	2,6	1,5
10	10	3,6	10	10	2,6	10	10	10	10
10	10	1,5	10	1,5	10	10	10	1,5	1,5
10	10	2,6	10	10	10	1,5	10	2,7	1,8
10	10	1,5	10	1,5	1,5	1,5	1,5	1,5	1,5
10	10	1,5	10	1,5	1,5	10	1,5	1,5	1,5

1,6	10	1,6	10	1,5	1,5	10	1,5	1,5	1,5
10	10	1,5	10	10	10	10	10	10	1,5
10	1,5	1,5	1,5	1,5	1,5	1,5	1,5	1,5	1,5
10	1,6	1,6	10	10	1,6	10	10	1,6	10
10	1,5	1,6	10	1,6	10	10	10	1,6	1,6
10	1,6	1,6	10	1,6	1,6	10	10	1,6	10
3,6	3,6	1,5	10	3,5	2,5	2,5	2,5	3,6	2,5
2,6	2,6	1,5	10	1,5	1,5	1,5	10	1,6	1,5
1,5	1,5	1,5	10	1,5	1,5	1,5	1,5	1,5	10
10	1,5	2,5	10	1,5	1,5	1,5	1,5	1,5	2,5
10	10	1,5	10	10	10	10	1,5	1,5	1,5
10	10	1,5	1,5	1,5	1,5	1,5	1,5	1,5	1,5
2,6	2,6	1,5	10	1,5	1,5	1,5	1,5	1,6	1,5
1,5	10	1,5	10	10	10	10	2,6	2,6	2,5
2,6	2,6	1,5	10	1,5	1,5	1,5	1,5	1,6	1,5
10	10	1,5	1,5	1,5	1,5	1,5	1,5	1,5	1,5
10	1,6	1,5	10	1,6	10	10	1,5	1,6	1,6
10	1,5	1,5	1,5	1,5	1,5	1,5	1,5	1,5	1,5
10	1,5	1,5	10	1,5	1,5	1,5	1,5	1,5	1,5
10	1,6	1,5	10	1,6	10	10	1,5	1,6	1,6
10	10	1,5	10	1,8	10	10	10	1,6	1,5
10	1,5	1,5	10	2,5	2,5	10	10	1,5	2,5
2,6	10	1,5	10	1,5	1,5	10	1,6	1,6	1,5
2,6	2,6	10	10	10	1,5	10	10	1,6	1,5
10	1,5	1,5	1,5	1,5	1,5	1,5	1,5	1,5	1,5
1,6	10	2,6	10	1,5	1,5	10	2,5	1,6	2,6
1,6	1,6	1,6	1,6	1,6	1,5	1,6	1,5	1,5	1,6
10	10	1,6	10	1,8	1,8	10	1,6	1,6	1,6
1,6	10	1,7	10	1,5	1,5	10	1,5	1,5	1,6
1,6	10	1,7	10	1,5	1,5	10	1,5	1,5	1,6
1,6	1,6	1,5	1,5	1,5	1,5	1,6	1,5	1,5	1,6
2,6	2,6	1,5	10	1,5	1,5	1,5	10	1,5	10
1,6	1,6	1,6	10	10	10	1,6	1,6	1,6	1,5
10	10	1,6	10	1,5	1,5	10	10	1,5	1,5
10	1,5	1,5	1,5	1,5	1,5	1,5	1,5	1,5	1,5
10	10	1,6	10	10	10	10	10	1,6	1,5
1,6	1,6	1,5	1,5	1,6	1,6	1,5	1,5	1,6	1,6
1,6	1,6	1,5	1,5	1,6	1,6	1,5	1,5	1,6	1,6
1,5	1,5	1,5	1,5	1,5	1,5	1,5	1,5	1,5	1,5
1,5	1,5	1,5	10	1,5	1,5	1,5	1,5	1,5	1,5
1,5	1,5	1,5	10	1,5	10	1,5	1,5	1,5	10
1,6	1,6	10	10	1,6	1,6	10	10	1,6	1,6
1,5	1,5	1,5	1,5	1,5	1,5	1,5	1,5	1,5	1,5
2,6	10	1,5	10	1,5	1,5	10	1,5	1,6	1,5
10	1,6	2,5	10	10	10	10	10	10	10
1,6	1,6	1,5	10	2,5	2,5	2,5	10	1,6	2,6
1,6	10	1,6	10	1,6	10	10	1,5	1,5	1,5

1,5	1,5	1,5	10	1,5	1,5	1,5	1,5	1,5	1,5
1,5	1,5	1,5	10	1,5	1,5	10	10	1,5	1,5
10	1,5	1,5	10	1,5	1,5	1,5	10	10	10
10	1,5	1,5	10	1,5	1,5	1,5	1,5	1,5	1,5
1,5	1,5	1,6	1,5	1,5	1,5	1,5	1,5	1,5	1,5
1,5	1,5	1,5	10	1,5	1,5	1,5	10	1,5	1,5
10	10	10	10	10	10	10	10	1,5	1,8
1,6	1,6	1,6	1,6	1,6	1,6	1,6	1,6	1,6	1,5
1,6	1,6	1,6	1,6	1,6	1,6	1,6	1,6	1,6	1,5
10	10	1,5	10	10	10	10	10	1,5	1,8
1,5	1,5	1,5	1,5	1,5	1,5	1,5	1,5	1,5	1,5
10	10	1,5	10	1,5	1,5	1,5	1,5	1,5	1,8
2,6	2,6	1,5	10	1,5	1,5	1,5	1,5	1,5	2,5
1,5	1,5	1,5	1,5	1,5	1,5	1,5	1,5	1,5	1,5
2,7	2,7	2,7	10	2,5	10	2,5	10	2,7	1,7
10	10	1,5	10	1,6	10	1,5	10	1,5	1,5
10	10	10	10	10	10	10	10	2,7	2,5
10	10	1,5	1,5	1,5	1,6	10	10	1,5	1,6
2,6	2,6	2,6	10	2,6	10	10	10	2,6	2,5
10	10	10	10	1,6	1,5	1,5	10	1,6	1,5
1,5	1,5	1,6	10	10	10	10	10	1,5	1,5
1,5	1,5	10	10	1,5	10	10	1,5	1,5	1,5
10	1,5	1,5	10	1,5	1,5	1,5	1,5	1,5	1,5
1,5	1,5	1,6	1,5	1,8	1,8	1,7	2,7	2,6	1,8
2,6	2,6	10	10	1,5	10	10	1,5	2,6	1,5
10	1,6	3,7	10	2,7	1,6	2,8	10	1,7	2,6
2,6	2,6	1,5	10	1,5	1,5	1,5	10	2,6	2,5
10	1,5	1,5	10	1,5	10	10	10	1,5	2,6
1,5	1,5	1,5	1,5	1,5	1,5	1,5	1,5	1,5	1,5
1,5	1,5	1,5	10	1,5	1,5	1,5	1,5	1,5	1,6
10	1,5	10	10	1,5	1,5	1,5	1,5	1,5	10
1,6	1,6	1,5	10	1,5	10	1,5	10	1,6	1,5
1,5	10	2,5	10	10	10	1,5	10	1,5	1,5
1,5	1,5	1,5	10	1,5	1,5	1,5	1,5	1,5	10
1,5	10	2,5	10	10	10	1,5	10	1,5	1,5
1,5	1,5	1,5	10	10	10	1,5	10	1,5	1,5
10	10	1,5	1,5	1,5	1,5	1,5	1,5	1,5	1,8
1,6	1,6	2,5	10	1,5	1,5	1,5	1,5	1,6	1,8
10	10	1,7	10	10	10	10	10	3,6	10
1,5	1,5	1,5	1,5	1,5	1,5	1,5	1,5	1,5	1,5
2,6	2,6	1,5	10	1,5	1,5	1,5	1,5	2,6	2,6
2,6	2,6	1,5	10	1,5	1,5	1,5	1,5	2,6	2,6
10	10	1,5	10	10	10	10	10	1,6	1,5
10	1,5	1,5	10	1,5	1,5	1,5	1,5	1,5	1,5
10	2,6	2,6	10	2,5	10	10	10	10	1,8
1,5	1,5	2,6	10	2,5	2,5	2,5	10	2,6	2,5
1,6	1,6	1,6	10	1,6	1,6	1,6	10	1,6	1,5

10	1,5	10	10	1,5	1,5	1,5	1,5	1,5	1,5
1,5	1,5	1,5	10	1,5	1,5	1,5	1,5	1,5	1,5
2,7	2,7	2,7	10	2,7	10	10	10	2,7	10
1,5	1,5	1,6	10	1,5	1,5	1,6	1,6	1,5	10
10	10	1,5	10	1,5	1,5	1,5	1,5	1,5	1,5
10	2,6	2,6	10	2,6	10	10	10	2,6	10
10	10	1,6	10	1,7	10	10	1,6	2,6	1,5
1,5	1,5	1,6	10	1,7	1,7	10	10	10	10
2,6	2,6	2,6	10	1,6	1,6	1,6	10	1,6	10
10	1,5	1,5	10	1,5	1,5	10	1,5	1,5	1,5
10	1,5	1,5	10	1,5	1,5	10	1,6	1,5	1,8
1,5	1,5	1,5	1,5	1,5	1,5	1,5	1,5	1,5	1,5
1,7	1,7	1,7	10	1,7	1,5	1,5	10	1,6	1,5
1,7	1,7	1,7	10	1,8	1,5	1,5	10	1,6	1,5
3,6	3,6	3,6	10	1,5	1,5	1,5	1,5	1,6	2,5
10	1,5	1,5	1,5	1,5	1,5	1,5	1,5	1,5	1,5
3,6	3,6	10	10	10	10	10	10	3,6	1,8
3,6	3,6	10	10	10	10	10	10	3,6	2,8
2,6	2,6	1,5	10	1,5	1,5	1,5	1,5	1,5	1,8
2,6	2,6	1,5	10	1,5	1,5	1,5	1,5	2,5	1,5
1,5	10	10	10	10	1,8	10	10	1,6	1,8
10	1,5	1,5	1,5	1,5	1,5	1,5	1,5	1,5	1,5
10	10	10	10	1,5	10	1,5	10	10	1,5
1,6	1,6	1,7	10	1,6	1,6	1,6	10	10	10
1,5	1,5	1,5	10	1,8	1,8	1,5	10	2,5	10
1,5	10	1,5	10	1,6	1,5	10	1,5	1,5	1,5
1,5	1,5	1,5	10	1,8	1,8	1,5	1,8	2,5	10
1,5	1,5	1,5	10	1,5	10	10	10	1,5	10
10	10	1,5	1,5	1,5	1,5	10	1,5	1,5	1,5
1,5	1,5	1,5	10	1,5	1,5	10	1,5	1,5	1,5
2,6	2,6	1,5	10	1,5	1,5	1,5	1,5	1,6	1,6
10	10	1,5	1,5	1,5	1,5	1,5	1,5	1,5	1,5
1,5	10	1,6	10	2,5	10	10	10	1,5	10
10	10	1,5	10	1,5	1,5	1,5	1,5	1,5	1,5
1,6	1,5	1,6	10	1,5	10	2,5	1,6	1,5	10
10	1,6	1,6	1,5	1,6	1,6	1,6	10	1,6	1,6
1,5	1,5	1,5	10	1,5	1,5	1,5	1,5	1,5	1,5
1,6	1,6	1,5	1,6	1,6	1,6	1,6	1,6	1,5	1,5
1,6	10	10	10	1,5	10	10	1,5	1,6	1,5
2,6	2,6	1,5	10	1,5	10	1,5	10	2,6	10
1,5	1,5	1,5	1,5	1,5	1,5	1,5	1,5	1,5	1,5
1,5	1,5	1,5	10	1,5	1,5	1,5	1,7	1,8	1,7
1,5	1,5	1,5	10	1,5	1,5	1,5	1,8	1,5	1,8
1,5	1,5	1,5	10	1,5	1,5	10	1,5	1,5	1,5
10	10	1,5	10	1,5	1,5	1,5	1,5	1,5	1,5
1,5	1,5	1,6	10	1,8	1,8	10	10	1,6	1,8

minced me	Ox tripe	Ox liver	Ox tail	Canned be	Lamb	Pork stew	grilled por	goat stew	polony
10	10	2,5	10	10	2,5	2,5	10	1,5	1,5
10	10	2,5	10	10	2,5	2,5	10	1,5	1,5
10	10	1,5	1,5	1,8	10	10	10	2,7	10
10	10	10	10	10	10	10	10	1,8	1,6
2,5	2,5	1,5	10	1,5	10	1,5	10	2,5	3,6
10	10	10	10	1,5	10	10	10	10	1,7
10	10	1,8	10	1,8	10	10	10	1,8	1,5
10	10	10	10	10	10	10	10	1,5	1,5
2,6	2,6	2,5	1,5	2,5	10	10	10	10	1,6
10	10	1,5	10	10	10	10	10	10	1,5
10	10	2,8	10	10	10	10	10	1,5	1,8
2,5	1,8	1,8	10	2,5	1,8	10	10	2,5	1,5
2,5	2,8	2,5	10	2,5	2,8	2,5	2,8	2,5	1,5
10	10	10	10	1,8	10	10	10	10	1,8
1,5	1,5	1,5	10	1,5	10	10	10	10	1,5
10	10	10	10	10	10	1,8	1,8	10	1,6
10	10	10	10	10	10	1,8	1,8	10	1,6
2,5	2,5	2,5	10	2,6	2,6	2,6	2,5	2,5	1,5
2,5	2,8	1,8	10	2,5	10	10	10	2,5	1,5
2,5	2,5	2,5	10	2,5	10	2,5	2,8	2,5	1,5
2,5	10	2,5	10	2,5	10	10	10	2,5	1,5
1,5	1,5	1,5	10	2,5	2,5	2,5	2,5	2,5	1,5
2,5	10	2,8	10	2,5	2,5	10	10	2,5	1,5
10	10	1,8	10	2,5	10	1,8	1,8	2,5	1,5
2,5	2,8	2,8	10	2,5	10	2,5	2,5	2,5	1,5
2,5	2,8	2,5	10	2,5	10	2,8	2,8	2,8	1,5
2,5	10	2,5	10	10	10	10	10	10	2,6
1,5	10	10	10	1,5	10	10	10	10	1,6
1,5	2,5	1,5	10	1,5	2,5	2,5	1,5	1,5	2,6
10	10	10	10	10	10	10	10	1,8	1,6
1,5	1,5	10	10	10	10	1,5	10	1,5	1,5
2,8	2,8	1,5	10	2,5	1,5	10	10	10	1,5
2,5	2,5	2,5	10	2,6	2,5	1,8	1,8	2,5	1,5
10	10	10	10	10	10	10	10	10	1,8
10	1,8	10	10	1,8	10	10	10	10	1,8
10	2,8	2,8	10	2,5	10	10	10	10	1,6
10	1,8	1,8	10	10	10	10	10	1,8	1,6
10	10	10	10	1,5	10	10	10	10	1,6
10	10	10	10	10	10	10	10	2,8	1,5
10	1,5	10	10	10	10	10	10	1,5	2,7
10	1,7	10	10	10	10	10	10	1,6	1,7
10	1,6	10	10	1,5	1,5	10	10	1,5	1,5
10	1,8	1,8	10	1,5	1,8	1,8	10	1,8	1,5
10	1,5	1,5	10	1,5	1,5	10	10	10	1,8
10	1,8	10	10	10	10	10	10	10	10
10	10	10	10	1,8	10	1,8	10	1,8	1,8

10	10	10	10	1,8	10	1,8	10	1,8	1,8
10	10	10	10	1,5	10	10	10	10	2,5
1,5	1,5	1,8	10	1,6	10	1,8	1,8	10	1,5
1,5	10	10	10	1,5	1,5	10	10	1,5	1,5
10	1,8	1,8	10	1,7	10	1,5	10	1,5	1,6
10	10	10	10	10	10	10	10	10	1,6
1,5	1,5	1,5	10	1,5	10	10	10	1,5	1,5
1,5	10	1,5	1,8	1,6	10	10	10	10	1,6
10	10	10	10	1,5	10	10	10	10	2,6
1,5	10	10	10	1,5	10	10	10	1,8	2,6
10	10	10	10	1,5	10	10	10	10	2,6
10	10	10	10	10	10	10	10	10	2,7
10	10	10	10	10	10	10	10	1,6	10
2,5	1,5	1,5	10	2,5	10	2,6	10	1,5	1,5
10	10	10	10	10	10	10	10	1,8	1,6
10	10	10	10	1,8	1,8	1,8	10	1,8	1,5
10	10	10	10	10	10	10	10	10	1,5
10	4,7	10	10	10	10	10	10	1,6	1,5
10	1,8	1,8	10	10	10	1,5	1,5	1,8	1,8
10	10	1,5	10	10	10	10	10	10	1,6
2,6	2,5	1,5	10	2,6	10	2,5	2,5	2,6	1,5
2,6	2,5	10	10	2,5	2,5	2,5	2,5	2,5	1,5
2,6	2,5	10	10	2,5	2,5	2,5	2,5	2,5	1,5
10	1,8	10	10	10	10	10	10	1,8	10
10	1,8	10	10	10	10	10	10	1,8	10
10	10	2,5	10	1,8	10	1,8	10	1,8	2,8
10	10	10	10	1,8	10	10	10	1,8	1,5
10	10	10	10	1,8	10	10	10	10	1,8
1,5	1,5	10	10	2,5	10	1,5	10	1,5	1,6
1,5	1,5	10	10	10	10	10	10	10	10
10	10	2,8	10	1,5	10	10	10	10	1,5
10	10	2,8	10	1,5	10	10	10	10	1,5
10	10	10	10	10	10	10	10	1,8	10
10	10	2,6	10	10	10	10	10	1,8	1,7
1,5	1,5	1,5	10	1,5	10	10	10	1,5	1,6
1,5	1,8	2,5	10	2,5	10	10	10	2,5	1,5
1,5	10	2,5	10	2,5	10	10	10	2,5	1,5
1,8	10	10	10	1,8	1,8	1,8	10	10	1,8
1,5	10	2,9	10	1,5	10	10	10	10	10
10	10	1,5	10	1,6	10	10	10	2,5	1,5
10	1,8	10	10	1,5	10	10	10	1,8	1,5
1,5	1,8	1,5	10	10	2,8	2,8	2,8	2,8	1,5
10	1,5	1,8	10	10	10	10	10	1,8	1,6
10	1,5	10	10	1,5	1,5	10	10	10	1,6
10	1,5	10	10	1,5	1,8	10	10	10	1,5
10	1,5	10	10	1,5	1,8	10	10	10	1,5
10	1,8	1,8	10	2,5	2,5	2,5	2,5	2,5	1,5

10	10	10	10	1,5	10	10	10	10	10
2,5	10	10	10	10	10	10	1,5	1,5	1,5
10	10	1,7	10	10	10	10	10	1,5	1,7
10	10	10	10	10	10	10	10	1,8	10
10	1,8	1,8	10	10	10	10	10	10	10
10	10	10	10	10	10	10	10	10	10
2,5	2,5	2,5	10	2,6	10	10	10	2,5	1,5
10	10	1,8	10	1,8	10	10	10	10	1,5
10	10	1,8	10	10	10	10	10	10	1,5
1,8	10	1,8	10	2,5	10	1,8	10	2,5	1,5
10	10	1,8	10	10	10	10	10	10	1,5
10	10	10	10	10	10	10	10	1,8	1,8
10	1,5	1,5	10	10	2,5	10	10	10	1,7
10	1,5	2,5	1,5	10	10	1,5	1,5	10	3,6
10	10	10	10	10	10	10	10	10	1,5
1,5	10	1,5	10	2,6	1,5	10	10	2,5	1,5
10	1,8	10	1,8	1,8	10	10	10	1,8	1,8
10	10	10	10	10	10	10	10	10	1,6
10	10	10	10	1,5	10	10	10	1,5	10
10	10	10	10	10	1,8	1,8	10	1,8	1,5
2,6	1,5	2,5	1,5	10	10	10	10	2,5	1,5
1,5	10	1,7	10	10	10	10	10	1,5	1,5
10	10	10	10	1,5	1,5	1,5	10	1,6	1,6
1,5	10	10	10	10	1,5	1,5	1,5	10	1,6
1,5	10	10	10	10	1,5	1,5	1,5	10	1,6
10	10	10	10	1,5	10	10	1,6	2,5	1,6
1,5	1,5	1,5	1,5	2,6	1,5	1,6	1,5	1,5	1,5
10	10	10	10	10	10	10	10	10	1,5
10	10	10	10	10	10	10	10	1,8	1,8
1,5	2,5	2,5	10	2,6	2,5	2,5	2,5	2,6	1,5
1,5	1,5	1,5	10	1,8	10	10	10	1,8	2,6
1,5	1,5	1,5	1,5	1,5	10	10	10	1,5	2,5
2,6	2,5	2,5	10	1,5	1,8	10	10	2,5	1,5
1,8	1,8	1,8	1,8	1,8	1,8	1,8	1,8	1,8	1,8
1,7	1,7	10	10	10	1,6	10	10	1,8	1,5
10	10	10	10	10	10	10	10	10	10
1,6	10	10	10	10	1,7	1,7	1,7	10	1,6
10	10	10	10	2,8	10	10	10	10	1,5
10	1,5	1,8	10	1,8	1,8	1,8	1,8	1,8	1,8
10	10	10	10	10	1,5	1,5	10	1,5	1,6
10	1,5	1,5	10	1,5	10	10	10	1,5	1,5
10	10	1,5	10	10	10	10	10	1,5	1,5
10	10	10	10	10	10	10	10	1,8	1,5
10	10	1,5	10	1,5	1,6	2,5	1,5	1,5	1,5
2,5	2,5	1,5	10	2,5	2,5	2,5	2,5	2,6	1,5
1,5	1,5	1,5	1,8	1,5	1,8	1,8	1,8	10	1,5
10	2,5	2,5	10	2,6	10	10	10	2,5	1,5

1,8	10	10	10	1,8	10	10	10	1,8	1,8
10	10	10	10	1,5	1,5	10	10	1,8	1,6
2,5	10	10	10	10	10	10	10	2,8	10
2,6	10	10	10	10	10	10	10	2,8	10
1,8	10	1,8	10	1,8	10	10	10	1,8	1,5
10	1,5	1,5	1,5	1,5	1,8	1,8	1,8	1,8	2,5
10	2,5	1,5	10	10	10	2,5	2,5	2,5	1,5
1,5	10	1,5	10	10	1,5	10	10	10	1,6
10	10	10	10	1,8	10	10	10	10	1,6
1,5	1,5	1,5	10	1,5	10	1,5	10	1,5	1,5
10	10	3,5	10	10	10	10	10	10	1,5
10	10	3,5	10	10	10	10	10	10	1,5
10	10	10	10	10	10	10	10	10	1,5
2,6	10	1,6	10	2,5	1,5	10	10	1,5	1,5
1,5	10	1,5	10	1,5	1,5	10	10	10	1,5
10	10	1,5	10	1,5	2,5	10	10	1,6	1,6
1,6	10	10	10	10	10	10	10	10	1,5
1,6	10	10	10	10	10	10	10	10	1,6
10	10	10	10	2,5	10	1,8	1,8	2,5	1,5
10	1,8	1,5	10	10	10	10	10	1,7	1,6
1,7	10	10	10	2,5	10	10	2,5	10	1,6
10	2,8	2,5	10	10	10	10	10	10	1,8
10	2,8	2,5	10	10	10	10	10	10	1,8
10	2,5	2,5	10	10	10	2,5	2,5	10	2,6
10	2,6	2,5	10	10	10	2,5	2,5	10	2,6
1,5	1,5	10	10	10	10	1,5	10	1,5	1,5
10	1,5	10	10	1,5	10	10	10	1,5	1,6
10	10	1,8	10	10	10	10	10	10	2,7
1,8	10	10	10	1,5	1,8	1,5	10	1,5	10
2,5	2,5	1,6	10	1,5	10	2,6	2,6	2,5	1,5
10	10	10	10	10	10	1,5	10	10	1,5
10	10	10	10	1,8	10	10	10	10	1,6
10	10	10	10	10	10	10	10	10	1,5
2,6	2,6	2,6	10	2,6	1,8	10	10	2,5	1,5
10	10	1,5	10	10	10	1,8	1,8	1,5	1,5
1,8	10	10	10	1,8	1,8	10	10	1,8	1,5
10	10	10	10	10	1,9	10	10	1,8	1,6
10	10	10	10	10	1,8	10	10	1,8	1,6
10	10	1,5	10	1,5	10	2,5	1,5	1,5	1,5
10	10	1,5	10	1,5	10	2,5	1,5	1,5	1,5
10	10	10	10	1,8	1,8	10	10	1,8	1,5
10	1,5	1,5	10	10	10	10	10	10	1,5
1,5	10	1,8	10	1,5	10	10	10	1,8	1,6
1,5	1,5	1,5	10	1,5	1,5	1,5	1,5	1,5	1,5
1,5	1,5	1,5	10	10	10	10	10	10	1,5
10	10	10	10	1,5	10	10	10	10	1,5
10	10	10	10	10	10	10	10	1,8	1,6

10	10	10	10	10	1,6	1,6	10	1,5	1,7
1,8	10	10	10	1,8	1,8	10	10	1,8	1,5
1,8	10	10	10	10	1,8	1,8	10	1,8	1,6
2,5	2,5	2,5	10	2,5	2,5	10	10	2,5	1,5
2,5	2,5	2,5	10	2,5	2,5	10	10	2,5	1,5
1,8	10	10	10	1,8	1,8	1,8	10	10	1,5
10	10	10	10	10	10	10	10	1,8	1,6
10	10	10	10	2,5	10	10	10	2,8	1,6
10	10	1,5	10	10	1,7	1,7	10	1,5	1,8
10	10	10	10	1,5	10	10	10	1,8	1,6
10	2,5	10	10	10	10	10	10	2,5	1,5
10	2,8	10	10	10	1,5	10	10	10	10
1,8	10	10	10	1,8	10	10	10	10	1,5
10	10	10	10	1,8	1,8	10	10	10	1,6
10	1,5	1,5	10	1,5	10	10	10	1,5	1,5
10	10	10	1,9	1,8	1,8	10	10	1,8	1,6
10	1,5	1,5	10	1,5	10	1,5	1,5	1,5	1,6
10	10	10	10	1,8	10	10	10	1,8	1,6
10	10	10	10	1,8	1,8	10	10	1,8	1,5
1,5	1,6	1,5	1,8	1,5	10	10	10	10	1,7
1,5	10	1,5	10	10	10	10	10	10	1,5
10	10	10	10	10	1,5	10	10	1,5	1,6
10	10	10	10	10	1,5	10	10	1,5	1,6
10	10	10	10	10	10	10	10	10	1,5
10	10	10	10	10	10	10	10	10	1,5
10	1,5	1,5	10	10	10	1,5	1,5	1,5	1,5
10	1,8	10	10	10	10	10	10	10	1,5
10	10	10	10	10	10	10	10	10	1,5
10	10	10	10	2,5	10	10	10	10	1,7
1,5	10	10	10	1,5	10	10	10	1,8	1,6
10	1,5	10	10	10	1,5	10	10	2,8	1,8
10	1,7	10	10	10	10	10	10	10	1,8
10	1,5	1,5	10	10	10	1,5	1,5	1,5	1,5
2,5	2,5	10	10	10	10	10	10	1,9	1,5
10	10	10	10	10	10	10	10	1,8	1,5
10	10	10	10	10	1,8	10	10	1,8	1,6
10	1,5	1,5	1,9	1,9	1,9	1,9	1,9	2,5	1,5
10	10	1,8	10	1,5	10	10	10	1,8	2,7
10	10	10	10	1,5	1,8	1,5	10	1,8	1,6
1,8	1,5	1,5	10	1,8	1,8	1,8	1,8	1,5	1,5
10	10	1,8	10	1,8	2,8	10	10	1,8	1,5
10	10	1,5	10	10	10	10	10	10	1,8
10	10	10	10	10	10	10	10	10	1,6
10	10	10	10	10	10	10	10	10	1,5
10	10	10	10	10	10	10	10	10	1,6
10	10	1,5	10	10	10	1,5	1,5	2,7	1,7
2,5	2,8	1,5	10	2,5	1,8	10	10	2,5	1,5

2,5	2,8	1,5	10	2,5	1,8	10	10	2,5	1,5
10	10	10	10	1,8	10	10	10	1,8	1,5
10	2,8	1,5	10	1,5	2,9	10	10	1,9	1,6
10	10	10	10	10	1,8	10	10	1,8	1,8
10	10	10	10	10	1,8	10	10	1,8	1,5
10	10	10	10	1,8	1,8	10	10	1,8	1,8
10	10	10	10	1,8	1,8	10	10	1,8	1,5
10	10	10	10	1,8	1,8	10	10	1,8	1,6
10	10	10	10	1,8	1,8	10	10	1,8	1,5
1,8	1,8	10	10	1,8	1,8	10	10	1,8	1,7
10	10	10	10	10	10	10	10	10	1,8
10	10	10	10	10	10	10	10	1,7	10
10	10	10	10	10	10	10	10	10	10
10	10	10	10	10	10	10	10	10	1,6
10	10	10	10	2,5	10	10	1,5	10	1,6
10	10	10	10	10	10	10	10	10	1,8
2,8	2,8	1,8	10	1,5	10	10	10	1,8	1,5
10	10	10	10	10	10	10	10	1,8	1,5
10	10	10	10	10	10	1,5	1,5	1,6	1,7
10	1,8	1,5	10	10	10	10	10	1,8	2,7
10	10	10	10	10	10	10	10	10	10
10	10	10	10	10	10	10	1,5	10	1,6
1,5	10	10	10	10	1,8	10	10	10	2,7
10	10	10	10	10	10	10	10	10	1,5
10	10	10	10	10	10	10	10	10	1,5
10	1,9	1,5	10	10	10	10	10	1,5	1,5
10	10	10	10	1,5	10	10	1,5	10	1,6
10	10	10	10	10	10	10	10	10	1,7
2,8	10	10	10	10	10	10	2,8	1,8	1,6
10	10	10	10	2,8	1,8	10	1,8	10	1,6
10	10	10	10	1,8	1,8	10	10	1,8	1,6
10	10	10	10	1,5	10	2,8	10	10	1,7
1,5	10	10	10	1,5	1,8	10	10	1,8	1,7
10	10	10	10	10	10	10	10	10	1,5
2,5	2,8	2,6	10	1,5	2,5	2,5	2,5	2,5	1,5
1,8	1,8	10	10	10	10	10	10	10	10
10	10	10	10	1,5	1,8	10	10	1,8	1,5
10	10	10	10	10	1,8	10	10	1,8	1,6
1,5	10	10	10	10	10	10	10	1,5	1,5
10	10	1,5	10	10	10	1,8	1,8	10	10
10	10	10	10	10	10	10	1,5	10	1,5
10	10	10	10	2,5	10	10	1,5	10	1,6
10	10	10	10	1,5	1,8	1,8	1,8	1,8	1,6
10	10	10	10	1,5	1,8	1,8	1,8	1,8	1,6
1,6	1,5	1,5	1,5	1,5	1,5	10	10	1,5	1,5
1,5	1,5	10	10	1,8	10	10	10	1,5	1,6
10	10	10	10	10	10	10	1,4	1,5	1,5

10	10	1,5	1,8	1,5	10	10	10	10	1,5
10	10	10	10	10	10	1,5	10	1,5	1,4
10	1,5	1,5	10	10	10	10	10	10	1,6
2,5	2,5	2,5	10	1,5	10	1,8	1,8	1,8	1,6
1,5	10	1,5	10	1,5	10	10	10	10	1,5
10	1,8	1,8	10	10	10	10	10	1,8	1,6
10	10	10	10	10	10	10	10	10	1,5
10	10	10	10	10	10	10	10	1,5	1,5
10	10	10	10	10	10	10	10	10	1,5
10	10	10	10	10	10	10	10	10	1,5
1,5	10	10	10	10	10	10	10	1,5	1,5
10	10	10	10	10	10	10	10	1,5	1,6
10	1,8	1,8	1,9	1,9	1,9	1,9	1,9	1,8	1,6
1,6	10	1,6	10	10	10	10	10	1,5	10
1,5	1,5	1,5	10	1,5	1,8	10	10	1,8	1,6
10	1,8	1,8	10	10	10	10	10	1,8	3,7
10	1,6	1,5	10	10	10	10	10	1,8	1,6
2,8	10	10	10	10	10	1,9	10	10	1,6
1,7	10	10	10	10	1,6	10	10	1,5	1,5
1,5	1,5	1,5	10	1,5	1,5	1,5	1,5	1,5	1,5
10	10	10	10	10	10	10	10	10	10
10	10	10	10	1,5	10	10	10	10	1,5
1,5	1,8	10	10	1,5	10	1,8	10	1,8	1,6
1,6	1,8	1,8	10	1,5	10	10	10	1,8	1,6
1,5	1,5	1,5	10	1,5	1,5	10	10	1,5	1,5
1,5	1,5	1,5	10	1,5	1,5	10	10	1,5	1,5
1,5	10	10	10	1,5	10	10	10	10	1,6
1,5	1,5	1,5	10	1,5	10	10	10	1,5	1,5
1,5	1,8	1,8	10	1,5	10	10	10	1,8	1,6
10	10	10	10	10	10	10	10	10	1,6
1,5	10	10	10	10	10	10	10	10	1,5
1,5	10	10	10	10	10	10	10	10	10
1,5	10	10	10	10	10	10	10	1,5	1,5
1,5	1,5	1,5	10	1,5	1,5	1,5	1,5	1,5	1,5
1,6	1,8	10	10	1,5	10	10	10	1,8	1,5
10	10	1,5	10	1,5	1,5	1,5	1,5	1,5	1,5
10	1,5	10	10	1,5	10	10	10	10	1,6
10	10	10	10	10	10	10	10	10	1,6
10	10	10	10	10	10	10	10	10	10
1,5	1,8	1,5	10	1,5	1,8	10	10	1,8	1,7
10	10	2,5	10	10	10	10	10	1,7	1,5
10	10	10	10	10	10	10	10	10	10
10	10	1,5	10	10	1,5	10	10	1,8	1,5
1,5	10	1,5	10	10	10	10	1,5	1,5	1,5
10	10	10	10	10	10	10	10	1,5	10
10	1,8	1,8	10	10	10	10	10	1,8	1,5
10	1,8	10	10	10	10	10	10	10	1,5

10	1,8	10	10	10	10	10	10	10	1,5
1,5	1,5	1,5	10	1,5	10	10	10	1,5	1,5
1,5	1,5	1,5	10	1,5	10	10	10	10	1,5
10	10	10	10	10	10	10	10	10	1,5
10	1,8	1,5	10	1,6	1,6	10	10	1,6	1,5
1,5	1,8	10	10	10	10	10	10	10	10
10	1,8	10	10	10	10	10	10	1,8	1,6
10	1,5	10	10	10	10	10	10	1,8	1,6
1,8	10	10	10	10	10	10	10	10	10
10	10	10	10	10	10	10	10	2,5	2,6
1,5	10	10	10	10	10	10	10	1,8	1,6
1,7	10	1,5	10	10	10	1,8	10	1,8	1,8
10	1,8	10	10	1,8	10	10	10	10	1,6
10	1,8	10	10	10	10	10	10	10	1,5
10	10	10	10	10	10	10	10	10	1,5
10	10	10	10	10	10	10	10	10	10
10	10	10	10	10	10	10	10	10	10
10	1,8	10	10	10	10	10	10	10	1,5
10	1,8	1,5	10	1,6	1,6	10	10	1,6	1,5
1,5	10	1,5	10	1,5	10	10	10	1,5	1,6
10	10	10	10	10	10	10	10	1,7	1,5
10	10	10	10	1,5	10	10	10	1,8	1,6
10	1,8	10	10	10	10	10	10	10	1,5
1,5	1,5	1,5	10	1,5	1,5	1,5	1,5	1,5	1,5
10	1,8	10	10	10	10	10	10	1,8	1,5
1,5	10	10	10	1,5	10	10	10	10	1,6
10	1,8	1,8	10	10	10	1,8	1,8	1,8	1,6
10	1,8	10	10	10	10	10	10	10	1,5
10	1,8	1,8	10	10	10	10	10	1,8	1,5
10	2,8	10	10	10	10	10	10	1,8	1,6
10	1,8	10	10	1,8	10	10	10	1,9	10
10	10	2,8	10	10	10	10	10	10	10
10	10	1,5	10	10	10	1,5	1,5	10	10
1,5	1,5	1,5	10	1,5	1,5	1,5	1,5	1,5	1,6
10	10	10	10	10	10	10	10	10	1,8
1,5	1,5	1,8	10	1,8	1,8	1,8	1,8	1,8	1,6
10	10	10	10	10	10	10	10	10	10
1,5	1,5	1,5	10	1,5	1,5	10	10	1,5	1,5
1,5	1,5	1,8	10	1,8	1,8	10	10	1,8	1,5
1,5	1,5	1,5	10	1,5	1,5	10	10	10	1,5
10	10	2,5	10	10	10	10	10	10	2,5
10	1,5	1,5	10	10	10	10	10	10	1,6
10	10	10	10	10	10	10	10	10	2,6
10	10	1,5	10	10	1,5	10	10	1,5	10
10	10	2,7	10	10	10	10	10	10	10
1,5	10	10	10	10	10	10	10	10	10
10	10	1,5	10	10	1,8	10	10	1,5	10

10	1,5	1,5	10	1,5	1,5	1,5	10	1,5	1,6
10	10	10	10	10	10	10	10	10	1,7
1,5	10	10	10	10	1,8	1,8	10	10	10
10	10	10	10	10	10	10	10	10	10
1,5	10	10	10	1,6	10	10	10	10	1,6
10	10	10	10	1,6	10	10	10	10	1,6
1,5	10	10	10	1,5	10	1,5	1,5	1,8	1,6
1,8	2,5	1,5	10	1,5	1,8	1,8	1,8	1,8	1,5
10	10	10	10	10	10	10	10	10	1,5
1,5	1,5	2,5	10	10	2,5	10	10	1,8	1,5
10	10	10	10	1,5	10	10	10	10	1,5
1,5	10	1,5	10	10	1,5	10	10	1,5	1,5
10	1,5	1,5	10	10	10	10	10	10	1,5
1,5	10	10	10	10	10	10	10	1,8	1,8
10	1,5	1,5	10	10	10	10	10	10	1,6
10	10	1,8	10	10	10	10	10	1,5	1,8
10	10	10	10	1,5	10	10	10	10	10
1,5	1,5	10	10	10	10	10	10	10	10
10	1,5	1,5	10	1,5	10	10	10	1,5	1,5
10	10	10	10	1,5	10	10	10	10	10
1,5	10	10	10	1,5	10	10	10	1,8	1,5
10	10	2,5	10	10	10	10	10	2,5	1,5
10	10	10	10	10	1,5	10	10	1,5	1,5
10	1,5	1,8	10	1,5	1,8	1,8	1,8	1,8	1,5
1,5	10	1,5	10	1,5	1,5	1,5	1,5	1,5	1,5
10	10	10	10	10	10	10	10	1,8	1,5
1,6	1,6	1,6	1,6	1,6	1,6	1,6	1,6	1,6	1,5
1,5	1,5	1,5	10	1,5	1,5	1,5	1,5	1,6	1,5
10	10	10	10	10	1,6	10	10	1,5	1,5
10	10	10	10	10	1,6	10	10	1,5	1,5
1,6	10	1,5	1,5	1,5	1,5	10	10	1,6	1,5
1,5	1,8	10	1,8	1,8	1,8	1,8	1,8	1,8	1,6
10	1,5	10	10	10	1,5	1,5	1,5	1,5	1,5
1,5	1,8	10	10	1,5	1,8	10	10	10	1,6
1,5	10	10	10	10	10	10	10	10	10
1,5	10	1,8	10	1,8	1,8	10	10	1,8	1,6
1,5	1,5	1,5	10	10	10	1,5	15	1,6	1,6
1,5	1,5	1,5	10	10	10	1,5	1,5	1,6	1,6
10	1,5	1,5	10	1,5	1,5	1,5	1,5	10	1,5
1,5	1,5	1,5	10	1,5	1,5	10	10	10	1,5
10	10	10	10	10	1,8	10	10	1,8	1,5
1,6	1,6	1,6	10	1,6	1,6	10	10	10	1,5
10	1,5	1,5	10	1,5	1,5	1,5	1,5	1,5	1,5
10	10	10	10	10	10	10	10	1,5	1,5
10	10	10	10	10	10	10	10	10	2,5
10	2,8	1,5	10	2,6	10	10	10	1,8	1,6
10	10	10	10	1,5	10	1,5	10	10	3,6

10	10	10	10	10	10	10	10	10	10
10	10	10	10	10	10	10	10	10	10
10	10	1,5	10	10	1,8	10	10	10	1,5
10	10	10	10	10	10	10	10	10	10
1,5	1,8	1,5	10	10	10	1,8	1,8	1,8	1,5
10	10	1,8	10	1,5	10	10	10	10	1,5
10	10	10	10	10	10	10	10	10	1,9
1,5	1,5	1,5	1,5	1,5	1,5	1,5	1,5	1,5	1,6
1,5	1,5	1,5	1,5	1,5	1,5	1,5	1,5	1,5	1,6
10	10	10	10	10	10	10	10	1,9	1,5
1,5	1,5	1,5	10	1,5	1,5	10	10	1,5	1,5
1,8	10	10	10	10	10	10	10	10	10
1,8	1,8	1,8	10	1,5	1,8	1,8	1,8	1,8	1,6
1,5	10	1,5	10	10	10	1,5	10	1,5	1,5
10	10	10	10	10	10	10	10	10	1,5
10	10	10	10	10	10	1,5	1,5	1,5	1,5
1,5	10	1,5	10	10	10	1,5	10	10	1,8
10	10	1,6	10	10	10	10	10	10	10
2,8	2,8	1,8	10	10	10	1,8	10	10	2,5
10	10	1,5	10	10	10	10	10	10	10
1,5	10	1,5	10	1,5	10	10	10	1,5	1,5
1,5	1,5	1,5	10	1,5	1,5	10	10	1,8	1,6
10	10	10	10	10	1,8	10	10	1,8	1,5
10	10	10	10	10	10	10	10	10	1,8
10	1,8	10	10	10	10	10	10	1,8	1,5
10	10	1,6	2,6	10	10	10	10	3,6	2,7
10	10	10	10	10	10	10	10	10	10
10	10	10	10	10	10	10	10	1,8	1,5
1,5	1,5	1,5	10	1,5	1,5	1,5	1,5	1,5	1,5
1,5	1,8	1,5	1,8	1,5	10	10	10	1,5	1,6
10	10	10	10	10	10	10	10	10	1,5
1,8	1,8	10	10	10	10	1,8	1,8	10	1,5
10	1,8	1,8	10	10	10	10	10	1,5	1,8
10	10	10	10	10	10	10	10	10	1,5
10	1,8	1,8	10	10	10	10	10	1,5	1,9
1,5	1,5	1,5	10	10	10	10	10	10	1,5
10	10	1,5	10	10	10	1,5	10	1,8	1,8
1,8	1,5	1,8	10	1,5	1,8	1,8	1,8	1,8	1,5
10	10	10	10	10	10	10	10	10	1,7
1,5	1,5	1,5	10	1,5	1,5	10	10	1,5	1,5
1,5	2,5	1,5	10	1,5	1,5	1,5	1,5	1,8	1,6
1,5	2,6	1,5	10	1,5	1,5	1,5	1,5	1,5	1,6
10	10	10	10	10	10	10	10	10	10
1,5	10	10	10	10	10	10	10	10	10
10	10	10	10	10	10	10	10	10	1,6
3,5	3,6	1,5	10	2,5	10	10	10	10	1,5
1,5	10	1,5	10	1,5	1,5	10	10	1,5	1,6

10	10	10	10	10	10	10	10	10	1,5
1,5	10	10	10	10	10	10	10	1,5	2,5
10	10	10	10	2,7	10	10	10	10	2,5
10	10	10	10	10	10	10	10	10	1,5
10	10	10	10	10	1,5	10	10	1,8	1,8
10	10	10	10	10	10	10	10	10	2,5
10	10	10	10	10	10	10	10	1,6	10
10	10	10	10	10	1,5	1,8	1,8	1,5	1,6
10	10	1,5	10	1,5	10	10	10	10	1,6
1,5	1,5	1,5	10	1,5	1,5	1,5	1,5	1,5	1,5
10	10	10	10	10	10	10	10	1,8	1,6
1,5	1,5	1,8	10	1,8	1,8	10	10	1,8	1,6
10	1,5	1,8	10	10	10	10	10	1,8	1,6
10	1,8	1,8	10	10	10	10	10	1,8	1,6
10	2,8	10	10	1,8	10	10	10	1,8	1,6
1,5	1,5	1,5	10	10	10	10	10	10	1,5
10	10	10	10	10	10	10	10	2,5	1,6
10	10	10	10	10	10	10	10	2,5	1,6
10	10	2,8	10	1,5	10	1,8	1,8	1,8	1,5
1,8	1,5	2,8	10	1,5	1,8	2,5	2,5	1,8	1,6
10	10	10	10	10	1,8	10	10	1,8	2,6
1,5	1,5	1,5	10	10	10	10	10	10	1,5
10	10	10	10	10	10	10	10	1,8	1,7
10	10	10	10	10	10	10	10	10	10
10	10	10	10	10	10	10	10	10	10
10	10	10	10	10	10	1,5	1,8	10	1,6
10	10	10	10	10	10	10	10	10	10
10	10	10	10	10	10	10	10	10	1,5
10	10	10	10	10	10	10	10	1,8	1,6
10	1,5	1,8	10	1,5	10	1,5	1,5	1,8	1,5
1,5	2,8	1,5	10	1,5	1,8	1,5	1,5	1,8	1,6
10	10	1,5	10	10	1,5	1,8	1,8	1,5	1,5
10	10	10	10	10	10	10	10	10	1,6
10	10	1,5	10	10	1,5	1,5	1,5	1,5	1,5
10	10	10	10	10	10	10	10	10	1,6
1,6	1,5	1,5	10	10	10	1,5	10	1,9	1,6
1,5	1,8	1,8	10	1,5	10	10	10	1,8	1,5
1,6	10	1,6	10	10	10	10	10	10	1,5
10	10	10	10	1,5	10	1,5	1,5	10	1,5
10	10	10	10	10	10	10	10	1,8	1,6
10	10	1,5	10	10	10	10	10	10	10
1,7	10	10	10	10	10	10	10	1,8	1,5
1,5	10	10	10	10	10	10	10	1,5	1,6
10	1,5	1,8	10	1,5	10	1,5	1,5	1,8	1,9
10	10	10	10	10	10	10	10	10	1,5
1,6	10	1,8	10	1,5	10	10	10	10	1,9

beef russia	pork russia	vienna	bacon	wors	canned fis	fried fish	steamed fi	dried smol	locust
1,6	10	2,5	10	1,5	1,6	10	10	10	10
1,6	10	2,5	10	1,5	1,6	10	10	10	10
10	10	10	10	2,7	1,9	1,5	1,5	1,5	10
1,5	10	10	10	1,5	10	10	10	1,5	10
1,5	1,5	1,5	10	2,5	2,5	2,5	2,5	10	1,5
10	10	10	10	10	10	1,5	10	10	10
10	10	10	10	1,8	10	2,5	10	10	10
10	10	10	10	1,5	1,5	10	10	10	10
1,5	10	1,5	10	1,5	2,6	2,6	1,5	2,5	2,5
10	10	10	10	1,5	1,5	10	10	10	10
10	10	1,5	10	2,7	2,6	10	10	10	10
1,5	10	1,5	10	1,5	2,6	2,6	10	1,6	10
1,5	1,5	1,5	10	2,6	2,6	1,6	10	2,6	2,6
10	10	1,8	10	10	1,5	10	10	10	10
2,5	10	1,5	10	1,5	1,5	1,5	10	10	1,5
10	10	1,6	1,5	1,5	1,5	10	10	1,5	10
10	10	1,6	1,5	1,5	1,5	10	10	1,5	10
1,5	1,5	1,5	10	2,6	2,6	10	10	2,5	2,5
1,5	10	2,5	10	2,6	2,6	10	10	1,5	2,5
1,5	1,5	1,5	10	2,6	2,6	10	10	2,5	10
1,5	10	1,5	10	2,6	2,6	10	10	1,5	2,5
1,5	1,5	1,5	10	2,6	2,6	1,5	10	1,5	1,5
1,5	10	1,5	10	2,6	2,6	1,5	10	1,5	10
1,5	1,5	1,5	10	2,6	2,6	2,5	10	1,5	2,5
1,5	1,5	1,5	10	2,6	2,6	1,5	10	1,5	2,5
1,5	1,5	1,5	10	2,6	2,6	2,6	10	2,5	10
2,6	10	2,6	10	1,5	2,5	10	10	10	10
10	10	10	10	1,5	1,5	1,5	1,8	10	1,5
1,5	1,5	10	10	2,5	2,5	1,5	1,5	1,5	1,5
1,6	10	1,6	10	10	1,6	10	10	10	10
10	10	10	10	1,6	10	10	10	10	10
10	10	10	10	2,5	2,6	10	10	2,5	10
1,5	1,5	10	10	1,8	2,6	10	10	2,6	10
10	10	10	10	10	1,5	1,5	10	10	2,9
10	10	10	10	1,5	1,5	1,5	10	10	1,5
10	10	10	10	2,6	1,5	10	10	1,6	1,8
10	10	10	10	2,7	1,5	1,5	10	10	10
2,5	10	10	10	1,6	1,5	10	10	10	10
10	10	1,5	10	2,5	2,6	10	10	2,6	2,6
10	10	10	10	1,5	10	10	10	10	10
1,7	10	1,7	10	1,7	1,6	1,6	10	1,6	10
1,5	10	10	10	1,5	1,5	1,5	10	1,5	1,5
1,5	10	10	10	1,5	10	1,8	10	1,6	1,8
1,5	10	10	10	1,5	1,6	10	10	10	1,8
10	10	10	10	1,5	1,5	1,5	10	1,5	1,5
10	1,8	10	10	10	1,5	10	10	10	1,9

1,8	10	10	10	10	1,5	10	10	10	1,9
10	10	10	10	10	3,6	1,5	10	10	10
10	10	10	10	1,5	2,6	10	10	2,6	2,6
1,5	1,5	1,5	10	1,5	1,5	10	10	10	1,5
10	10	1,5	10	1,8	1,5	1,5	10	10	10
10	10	10	10	1,6	10	10	10	10	10
1,5	10	10	10	1,5	1,5	1,5	1,5	1,5	1,6
10	10	10	10	1,6	10	10	10	1,6	10
10	10	1,6	10	3,6	1,5	10	10	10	10
10	10	10	10	1,6	1,5	10	10	1,5	1,9
10	10	10	10	1,5	2,6	10	10	1,5	10
10	10	10	10	1,6	1,5	10	10	1,6	10
10	10	10	10	10	1,6	10	10	1,6	10
1,5	1,5	1,5	10	2,6	2,6	1,5	10	10	1,5
1,5	10	10	10	1,5	10	1,5	10	10	10
10	10	1,8	10	10	1,5	10	10	10	1,9
10	10	1,5	10	10	2,5	10	10	10	10
1,5	1,5	1,5	10	2,7	2,6	1,5	10	10	10
1,5	10	10	10	1,5	1,5	1,5	10	1,5	1,5
10	10	10	10	1,5	1,5	10	10	10	10
1,5	1,5	1,5	10	2,5	2,6	10	10	2,6	2,5
1,5	1,5	1,5	10	2,5	2,5	2,5	10	2,5	2,9
1,5	1,5	1,5	10	2,5	2,5	2,5	10	2,5	2,9
10	10	10	10	1,5	1,5	1,8	1,5	1,5	1,5
10	10	10	10	1,5	1,5	1,8	1,5	1,5	1,5
10	10	10	10	2,5	2,5	10	10	1,8	1,5
10	10	10	10	1,5	10	10	10	1,5	1,5
1,8	1,8	1,8	10	1,5	1,5	10	10	10	1,9
2,6	2,6	1,6	10	1,6	1,6	1,5	1,6	10	1,5
10	10	10	10	2,6	1,6	1,6	10	1,6	4,7
1,5	10	1,5	10	2,5	1,5	1,5	1,5	1,8	1,5
1,5	1,5	1,5	10	2,5	1,5	1,5	1,5	1,8	1,5
1,8	10	10	10	1,5	1,5	1,5	1,5	1,5	1,5
1,7	10	1,7	10	1,7	2,5	1,5	10	10	10
10	10	10	10	1,5	1,5	1,5	10	1,5	1,5
1,5	10	1,5	10	2,6	2,6	2,6	10	2,6	10
1,5	1,5	1,5	10	2,5	2,5	10	10	10	2,5
10	10	1,8	10	1,5	1,5	10	10	10	1,9
10	10	10	10	10	2,5	10	10	10	1,9
1,5	10	10	10	1,5	1,5	10	10	10	1,5
1,5	10	1,8	10	1,5	1,5	1,5	10	1,5	1,5
1,5	10	1,8	10	2,5	1,5	10	2,5	1,5	1,5
1,8	10	10	10	2,5	1,5	10	10	1,5	1,5
1,6	10	1,6	10	2,5	1,5	1,8	1,5	1,5	1,5
1,5	10	10	10	2,6	1,5	10	1,5	1,5	1,5
1,8	10	10	10	1,6	1,5	10	10	1,5	1,5
1,5	1,5	1,5	10	1,8	2,6	1,5	1,5	2,5	2,5

10	10	10	10	1,8	1,5	1,5	10	10	1,9
1,5	10	1,5	10	3,5	10	10	10	10	10
1,7	10	1,7	10	2,5	1,6	10	10	10	10
10	10	10	10	1,8	2,6	10	10	1,5	10
10	10	10	10	1,5	2,5	2,5	10	2,5	10
10	10	10	10	10	1,5	1,5	1,5	1,5	1,5
1,5	10	1,5	10	2,5	2,6	2,5	2,5	2,5	2,5
1,5	10	10	10	1,5	1,6	1,5	10	10	1,5
10	10	10	10	1,5	1,6	1,6	1,6	1,6	1,6
10	10	10	10	2,5	10	1,5	10	10	1,5
10	10	10	10	1,5	1,5	10	10	10	1,9
10	10	10	10	10	10	10	10	10	1,9
10	10	10	10	1,7	1,5	1,5	10	10	10
10	3,5	10	10	2,6	2,5	10	10	10	10
10	10	10	10	1,5	10	10	10	10	1,9
1,5	10	1,5	10	2,5	1,5	10	10	10	10
10	10	10	10	2,8	2,8	10	10	10	1,8
10	10	10	10	1,5	1,5	1,5	10	10	10
10	10	1,5	10	1,5	1,5	1,5	10	10	10
1,5	1,5	1,5	10	10	1,5	10	10	10	10
10	10	1,5	10	2,6	2,6	10	10	10	10
10	10	10	10	1,5	1,5	1,5	10	1,5	1,5
1,6	10	1,6	10	1,5	1,5	10	10	10	1,5
1,6	10	1,6	10	1,5	1,5	10	10	10	10
1,6	10	1,6	10	1,5	1,5	10	10	10	1,5
1,6	10	1,6	10	3,5	3,5	10	10	10	10
10	10	10	10	1,5	2,6	1,5	10	10	10
10	10	10	10	1,8	10	10	10	10	10
10	10	10	10	10	10	10	10	10	1,9
1,5	1,5	1,5	10	2,6	2,6	2,6	1,5	2,5	2,6
2,6	10	2,5	10	1,5	1,5	1,5	10	10	10
1,5	10	1,5	10	1,5	1,5	10	10	10	10
1,5	10	1,5	10	2,6	2,6	1,5	10	2,6	2,6
1,8	1,8	1,8	1,8	1,6	1,5	1,5	10	10	10
1,7	10	1,7	10	1,8	1,5	10	10	10	10
10	10	10	10	1,8	1,5	10	10	10	10
1,6	1,6	1,6	1,6	1,6	1,5	1,7	10	10	10
10	10	10	10	2,5	10	10	10	10	10
1,8	1,8	1,8	10	1,8	1,5	1,5	10	10	10
1,6	1,6	1,6	1,6	1,5	1,5	1,5	10	10	1,9
1,5	10	10	10	10	10	10	10	10	10
1,5	10	1,5	10	1,5	1,5	1,5	1,5	1,5	10
10	10	10	10	1,5	1,5	10	10	10	10
1,5	1,5	1,5	1,8	2,6	1,5	10	10	10	10
1,5	1,5	1,5	10	2,6	2,6	10	10	10	10
1,8	1,8	1,5	10	1,5	1,5	1,5	1,5	10	10
1,5	10	1,5	10	2,6	2,6	10	10	2,6	2,6

10	10	1,8	10	10	1,5	1,5	10	10	1,9
10	10	1,5	10	1,5	1,5	1,5	10	10	1,5
10	10	10	10	2,5	2,5	10	10	2,5	10
10	10	10	10	2,5	2,5	10	10	2,5	10
1,5	10	1,5	10	1,5	1,5	10	10	1,6	1,9
1,8	10	2,5	1,8	1,5	1,5	1,5	1,8	10	1,5
1,5	1,5	1,5	10	2,6	2,6	2,6	10	2,6	10
1,6	10	1,6	10	1,6	1,5	1,5	10	10	10
1,6	10	1,8	10	2,5	10	1,5	10	10	10
1,5	10	1,5	10	10	1,5	1,5	10	10	1,5
1,5	10	1,8	10	3,6	10	10	1,5	1,5	10
1,5	10	1,8	10	3,6	10	10	1,5	1,5	10
1,5	10	1,5	10	10	1,5	10	10	10	10
1,5	10	1,5	10	1,6	1,5	1,5	10	10	2,6
1,5	10	1,5	10	1,5	1,5	10	10	10	1,5
1,6	10	1,6	10	2,6	10	10	10	10	1,5
1,5	10	1,5	10	1,6	1,5	10	10	10	10
1,5	10	1,5	10	1,6	1,5	10	10	10	10
1,5	1,5	1,5	10	2,6	2,6	10	10	2,6	10
1,6	1,6	1,6	10	1,5	1,6	10	10	10	10
10	1,6	1,6	10	2,5	2,5	2,5	1,5	1,5	10
1,8	10	1,8	10	2,5	2,5	10	10	1,5	10
1,8	1,8	1,8	10	2,5	2,5	10	10	1,5	10
1,6	10	1,5	10	2,6	2,5	10	10	2,5	10
1,6	10	1,5	10	2,6	2,5	10	10	2,5	1,8
1,5	10	10	10	1,6	1,6	1,5	10	1,5	10
1,5	10	1,5	10	1,5	1,5	1,5	1,5	10	10
2,7	10	2,7	10	1,5	1,5	10	10	1,5	10
10	10	10	10	10	1,6	1,5	10	10	1,9
1,5	1,5	1,5	10	2,6	2,6	10	10	10	1,5
1,6	10	1,8	10	2,5	2,5	1,5	10	10	10
10	10	1,6	10	1,8	1,5	10	10	10	1,8
10	10	10	10	1,6	1,6	1,5	10	10	10
1,5	10	1,5	10	2,6	2,6	10	10	2,5	10
1,5	10	1,5	10	1,5	1,6	1,5	10	10	10
10	10	10	10	1,8	1,5	10	10	10	1,9
10	10	10	10	1,8	1,5	1,5	10	10	10
10	10	10	10	1,8	1,5	10	10	10	1,8
1,5	1,5	1,5	10	1,5	1,5	1,5	1,5	1,5	1,5
1,5	1,5	1,5	10	1,5	1,5	1,5	1,5	1,5	1,5
10	10	10	10	1,8	1,8	10	10	10	10
1,5	10	1,5	10	2,5	10	10	10	2,5	10
10	10	10	10	1,5	1,5	10	10	10	1,9
1,5	1,5	1,5	1,5	1,5	1,5	1,5	10	1,6	1,5
1,5	10	10	10	1,5	10	10	10	10	1,5
1,5	10	2,5	10	1,5	10	10	10	10	10
10	10	10	10	1,6	10	10	10	1,6	10

1,7	1,7	1,7	10	1,5	1,7	10	10	10	1,9
1,5	10	1,5	10	1,5	10	10	10	10	1,9
10	10	10	10	10	1,5	10	10	10	10
1,5	10	1,5	10	2,6	2,6	2,5	2,5	2,6	2,6
1,5	10	1,5	10	2,6	2,6	2,5	2,5	2,6	2,6
1,5	10	10	10	1,8	1,5	1,5	10	10	10
1,6	10	10	10	10	1,5	10	10	10	1,9
10	10	10	10	2,6	10	1,5	10	10	10
1,8	1,8	1,8	10	1,5	1,7	1,7	10	10	10
10	10	10	10	1,5	1,5	10	10	1,6	10
10	10	1,5	10	1,5	2,5	1,5	1,5	10	10
10	10	10	10	2,5	2,5	10	1,5	2,5	10
1,5	10	1,6	10	10	1,5	1,5	10	10	10
10	10	10	10	1,8	1,5	10	10	10	10
1,5	1,5	1,5	10	1,5	1,5	1,5	1,5	1,5	10
10	10	10	10	1,8	1,5	10	10	10	1,9
1,5	10	1,5	10	1,6	1,8	1,8	1,8	1,8	1,5
1,8	10	10	10	1,8	1,5	10	10	10	1,9
10	10	1,5	10	1,8	1,5	1,5	10	10	1,9
10	10	1,6	10	1,5	1,5	10	1,5	10	1,9
1,5	10	10	10	1,5	1,5	1,5	10	10	10
1,6	10	1,6	10	1,6	1,5	1,6	1,5	1,5	1,5
1,6	10	1,6	10	1,6	1,5	1,6	1,5	1,5	1,5
10	10	10	10	10	10	10	10	10	10
1,5	10	1,5	10	2,5	2,5	10	10	1,5	10
1,5	1,5	1,5	10	1,5	1,5	1,5	1,5	1,5	10
1,5	10	1,5	10	2,6	2,6	10	10	2,5	10
1,5	10	1,5	10	1,8	1,5	10	10	10	1,9
1,7	10	1,6	10	1,5	1,5	1,5	10	10	10
1,6	10	1,6	10	1,5	10	10	10	10	1,9
10	10	10	10	2,6	1,5	10	10	2,5	10
10	10	10	10	1,5	1,6	1,6	10	10	10
1,5	1,5	1,5	1,5	1,6	1,5	1,5	10	1,5	10
1,5	10	10	10	10	2,5	10	1,8	1,5	10
1,5	10	1,5	10	1,8	10	10	10	10	10
1,6	10	10	10	1,8	10	1,5	10	10	1,8
1,5	10	1,5	10	1,5	1,5	1,6	10	10	10
2,7	10	10	10	1,5	2,6	10	10	10	1,9
1,5	10	10	10	1,8	10	10	10	10	10
10	10	1,5	10	1,5	10	10	10	10	1,5
1,8	10	1,8	1,8	2,5	10	10	10	10	10
1,8	1,8	1,8	10	1,5	1,6	1,6	10	10	1,9
1,6	10	10	10	1,6	1,6	1,5	10	1,5	10
10	10	1,5	10	3,5	3,6	1,5	1,5	10	10
1,6	10	10	10	1,6	1,6	10	10	1,6	10
1,7	1,8	1,8	1,8	1,8	2,6	10	10	10	1,9
1,5	10	1,5	10	2,6	2,6	2,5	10	2,5	10

1,5	10	1,5	10	2,6	2,6	2,5	10	2,5	10
10	10	10	10	1,8	1,5	10	10	10	1,9
1,5	10	1,8	10	2,5	1,5	10	10	2,5	1,5
10	10	10	10	1,8	1,5	10	10	10	1,9
10	10	10	10	10	1,5	1,5	10	10	1,9
10	10	10	10	1,8	10	10	10	10	10
10	10	10	10	1,8	10	10	10	10	1,9
10	10	10	10	1,9	1,5	10	10	10	10
10	10	10	10	1,8	10	10	10	10	1,8
1,6	10	10	10	1,8	1,5	10	10	10	1,9
10	10	10	10	10	1,5	1,5	10	10	1,5
10	10	10	10	1,5	1,5	1,5	10	1,5	1,5
10	10	10	10	1,6	2,6	2,5	10	10	1,5
10	10	10	10	1,6	2,6	2,5	10	10	1,5
10	10	10	10	2,5	1,6	1,5	10	10	1,5
1,8	10	1,8	10	1,7	1,5	1,6	10	10	1,9
1,5	10	1,5	10	2,5	2,5	2,5	2,5	2,5	2,6
10	10	1,5	10	2,5	2,5	2,5	2,5	1,5	1,5
10	10	1,8	10	1,5	1,5	10	10	10	1,8
10	10	10	10	1,5	1,5	10	10	1,6	1,9
10	10	10	10	2,5	10	10	10	10	3,5
10	10	10	10	2,5	1,5	10	10	10	10
2,7	10	2,7	10	1,5	1,5	10	10	10	1,9
10	10	10	10	10	2,5	2,5	10	10	10
10	10	10	10	10	2,5	2,5	10	10	10
10	10	1,5	10	1,5	1,5	1,6	1,5	1,5	1,5
10	10	10	10	2,5	1,6	1,8	10	10	1,5
10	10	10	10	10	1,5	10	10	1,5	1,5
1,6	10	1,8	10	2,5	2,5	10	10	10	1,6
1,5	10	1,5	10	2,5	2,5	10	10	10	1,5
1,6	10	1,6	10	1,5	10	10	10	10	1,6
1,6	10	1,7		2,5	1,8	1,5	10	10	1,5
1,5	10	10	10	1,6	1,5	10	10	10	1,9
10	10	10	10	1,5	1,5	1,5	1,5	1,5	1,5
1,5	1,5	1,5	10	2,6	2,5	2,5	2,5	2,5	1,5
10	10	10	10	10	1,5	10	10	10	1,9
1,8	10	10	10	2,5	2,5	1,8	1,8	10	1,5
10	10	10	10	1,8	1,5	10	10	10	1,9
1,5	10	1,8	10	1,5	10	10	10	10	1,5
10	10	10	10	1,5	2,5	10	10	1,5	1,5
1,5	10	1,5	10	2,6	1,8	1,5	10	10	1,5
1,6	10	1,5	10	2,5	1,5	1,5	10	10	10
1,6	10	1,5	10	1,6	1,5	10	10	10	10
1,6	10	1,5	10	1,6	1,5	10	10	10	10
1,5	10	1,5	10	1,5	1,5	1,5	10	10	1,9
1,6	10	1,6	10	2,5	1,5	10	10	10	10
1,5	2,6	2,6	10	1,5	1,5	1,5	10	10	10

10	10	10	10	1,5	10	1,5	10	10	10
10	10	10	10	1,4	1,5	10	10	10	10
1,6	10	1,6	10	1,6	1,6	1,5	1,5	1,5	1,5
1,6	10	1,6	10	2,6	1,5	10	10	10	1,9
1,5	10	1,5	10	1,5	1,5	1,5	1,5	1,5	1,9
10	10	10	10	1,6	10	1,5	10	1,5	10
10	10	1,5	10	10	10	10	10	10	10
1,5	1,5	1,5	10	1,5	1,5	1,6	10	1,6	10
1,5	10	1,4	10	1,5	2,6	10	10	10	10
1,5	10	1,4	10	1,5	2,6	10	10	10	10
1,5	1,5	1,5	10	1,5	1,6	3,5	10	1,5	10
1,5	10	1,5	1,5	1,5	1,5	10	10	10	1,9
10	10	10	10	1,6	10	1,5	10	1,5	10
1,5	10	1,5	1,6	1,5	1,5	1,6	10	1,6	10
1,5	10	1,5	10	1,6	1,5	10	10	1,5	10
1,5	10	1,5	10	2,5	2,6	1,5	10	10	1,9
1,5	10	1,5	10	2,6	1,5	10	10	1,5	1,9
1,8	10	1,7	10	1,6	2,6	1,6	10	10	10
1,9	10	1,6	10	1,6	1,6	1,6	1,6	1,6	10
1,5	1,5	1,5	10	1,5	1,5	1,8	10	1,8	10
10	10	10	10	10	1,5	10	10	10	10
10	10	10	10	1,5	1,6	10	10	10	10
10	10	10	10	1,6	1,5	10	10	1,6	1,9
10	10	10	10	1,6	1,6	10	10	1,6	10
1,5	10	1,5	10	1,5	1,5	1,5	1,5	1,5	1,5
1,5	10	1,5	10	1,5	1,5	1,5	10	1,5	1,5
1,5	10	10	10	1,5	1,5	10	10	1,5	10
1,5	10	1,5	10	1,5	1,5	1,5	1,5	1,5	10
1,6	10	1,6	10	1,8	1,5	10	10	1,5	10
10	10	10	10	10	1,5	1,5	10	1,5	1,5
1,5	10	10	10	1,5	1,5	1,4	10	10	10
10	10	10	10	10	1,5	10	10	10	1,9
1,5	10	10	10	1,5	1,5	1,5	10	10	10
1,5	1,5	1,5	10	1,5	1,5	1,5	1,5	1,5	1,5
10	10	10	10	1,7	1,5	10	10	1,5	1,9
1,5	1,5	1,5	1,5	1,5	1,5	2,7	10	10	10
1,6	10	10	10	1,5	1,5	1,5	10	1,5	10
10	10	10	10	1,6	1,6	10	10	1,6	10
10	10	10	10	1,5	1,5	10	1,5	1,5	1,9
1,7	10	10	10	1,6	1,6	10	10	1,6	1,9
1,8	10	1,8	10	1,5	1,5	10	10	10	10
10	10	10	10	1,6	1,5	1,5	10	10	1,9
1,5	10	10	10	1,5	1,5	1,5	10	1,5	10
1,5	1,5	1,5	10	1,5	1,5	10	10	10	10
10	10	1,5	10	1,5	10	10	10	1,9	1,5
1,5	10	1,5	10	1,8	1,5	1,5	10	10	1,9
1,5	10	10	10	1,6	1,5	10	10	1,5	1,9

1,5	10	10	10	1,5	1,5	10	10	1,5	1,9
1,5	10	1,5	10	1,5	1,5	1,5	1,5	1,5	1,5
1,5	10	1,5	10	1,5	1,5	10	10	10	1,5
10	10	10	10	2,5	2,5	2,6	10	10	10
1,5	10	10	10	1,6	1,6	1,5	10	10	10
10	10	10	10	10	1,5	1,5	10	10	10
1,6	10	10	10	1,6	1,5	10	10	1,5	1,9
1,6	10	10	10	2,6	1,5	10	10	10	1,9
10	10	10	10	1,5	1,5	1,5	1,9	10	1,9
2,6	10	10	10	2,6	2,5	2,7	10	10	10
1,6	10	10	10	1,5	1,5	1,7	10	1,6	10
1,8	1,8	1,8	1,8	1,5	1,5	1,5	10	10	10
1,6	10	1,5	10	1,6	1,5	10	10	1,5	10
1,5	10	10	10	1,5	1,5	10	10	1,5	1,9
1,5	10	10	10	1,5	1,5	10	10	1,5	1,9
10	10	10	10	1,8	1,5	10	10	1,5	1,9
10	10	10	10	1,8	1,5	10	10	1,5	1,9
1,5	10	10	10	1,5	1,5	10	10	10	10
1,5	10	10	10	1,6	1,6	1,5	10	10	10
1,6	10	1,5	10	1,5	1,5	1,5	1,5	1,5	1,5
1,5	10	1,5	10	1,5	1,5	1,5	10	10	10
1,6	10	10	10	1,6	1,5	1,8	10	1,5	10
1,5	10	10	10	1,6	1,5	10	10	1,5	10
1,5	10	1,5	1,5	1,5	1,5	1,5	1,5	1,5	1,9
1,5	10	10	10	1,5	1,5	10	10	1,5	10
1,6	10	1,6	10	1,6	1,5	10	10	1,5	10
1,6	10	1,5	10	1,6	1,5	10	10	1,5	10
1,5	10	10	10	1,5	1,5	10	10	1,5	10
1,5	10	10	10	1,5	1,5	10	10	1,5	10
1,6	10	10	10	1,6	1,5	10	10	1,5	1,9
10	10	10	10	10	1,8	10	10	10	2,8
10	10	10	10	2,8	1,5	10	10	10	10
10	10	10	10	1,5	10	10	10	10	10
1,6	1,6	1,6	10	1,6	1,5	1,5	10	1,5	1,5
10	10	10	10	1,8	2,5	10	10	2,6	1,9
1,6	10	1,5	10	1,8	1,5	1,5	1,5	1,5	1,5
10	10	10	10	10	3,6	3,6	3,6	10	10
1,5	10	10	10	1,6	1,6	1,5	10	1,5	1,5
1,5	10	1,5	10	1,5	1,5	1,5	1,5	1,5	1,5
1,5	10	1,5	10	1,5	1,5	1,5	1,5	1,5	1,5
1,6	10	2,5	2,5	1,5	2,6	10	10	10	10
1,6	10	1,6	10	3,6	1,5	10	10	1,5	10
2,6	10	2,5	10	1,6	2,5	10	10	10	10
10	10	10	10	10	10	1,5	10	10	1,5
10	10	10	10	10	1,6	10	10	10	10
10	10	10	10	1,5	1,5	1,5	10	10	10
10	10	10	10	10	1,5	1,5	10	10	10

1,5	1,5	1,5	10	1,6	1,5	10	10	1,5	10
10	10	1,5	10	1,5	1,5	10	10	10	10
10	10	1,5	10	10	1,5	1,5	10	10	10
10	10	10	10	1,5	1,6	10	10	1,5	10
10	10	10	10	1,5	1,5	10	10	1,5	10
10	10	10	10	1,6	1,5	10	10	1,5	10
1,5	10	1,6	10	3,6	1,5	10	10	1,5	10
1,5	10	1,5	10	1,6	1,5	10	10	1,5	1,9
10	10	10	10	10	1,5	1,5	1,5	1,5	10
1,5	10	1,8	10	2,5	1,5	10	10	10	10
1,5	10	1,5	10	1,5	1,5	10	10	10	10
1,5	10	1,5	10	1,5	1,5	10	10	10	10
1,5	10	1,5	10	1,6	1,5	10	10	10	10
1,8	10	1,8	10	1,6	10	1,5	10	10	10
1,6	10	1,5	10	1,6	1,5	10	10	10	10
10	10	10	10	10	1,6	1,5	10	10	1,9
10	10	10	10	1,6	10	10	10	10	10
10	10	10	10	10	1,5	10	10	10	10
1,5	10	1,5	10	1,5	1,6	1,6	10	1,5	1,5
10	10	10	10	10	1,6	10	10	10	10
1,5	10	10	10	1,5	1,5	10	10	1,6	10
1,5	10	1,5	10	1,5	1,5	1,5	10	10	10
1,5	10	1,5	10	2,5	1,5	10	10	10	10
1,5	10	1,8	1,8	1,6	1,5	1,8	1,8	1,9	10
1,5	1,5	1,5	1,5	1,5	2,5	1,5	10	10	10
1,5	10	1,5	10	1,6	1,5	1,5	10	10	10
1,5	1,6	1,5	1,5	1,5	1,5	1,5	1,5	1,5	10
1,5	1,5	1,5	10	1,6	1,6	1,5	10	10	1,9
1,5	10	1,5	10	1,6	1,5	1,6	10	10	10
1,5	10	1,5	10	1,6	1,5	1,6	10	10	10
1,5	10	1,5	1,6	1,5	1,5	1,6	1,6	1,6	10
1,6	10	1,6	10	1,6	1,5	1,8	1,8	1,5	1,9
1,5	10	1,5	10	1,5	1,5	1,5	1,5	1,5	1,5
10	10	10	10	1,5	1,5	10	10	1,5	10
10	10	10	10	1,5	10	1,5	10	10	1,9
10	10	10	10	1,6	1,5	10	10	1,5	10
1,6	10	1,6	10	1,6	1,5	1,5	10	10	1,9
1,6	10	1,6	10	1,6	1,5	1,5	10	10	1,8
1,5	1,5	10	10	1,5	1,5	1,5	10	1,5	10
1,5	10	1,5	1,5	1,5	1,5	1,5	10	1,5	1,9
1,5	10	1,5	10	1,5	1,5	1,5	10	1,5	1,9
1,5	10	1,5	1,5	1,5	10	1,5	1,5	1,6	10
1,5	1,5	10	10	1,5	1,5	1,5	10	1,5	10
1,5	10	10	10	2,5	1,5	10	10	10	10
1,8	1,5	1,8	1,8	1,5	1,5	1,5	10	1,5	10
1,6	10	1,5	10	2,6	1,5	1,5	1,8	2,6	1,9
10	10	1,5	10	1,5	10	10	10	10	10

10	10	10	10	10	1,5	1,5	10	10	1,9
10	10	10	10	10	1,5	1,5	10	10	1,9
1,5	10	1,5	10	1,5	1,5	10	10	10	10
10	10	1,5	1,5	1,5	10	10	10	10	10
1,5	1,5	1,5	1,8	1,5	1,5	1,5	1,5	1,5	1,8
1,5	10	10	10	1,5	1,5	1,8	10	1,5	10
1,5	10	1,8	10	1,8	1,8	1,8	10	10	10
1,6	1,6	1,6	1,6	1,6	1,5	1,5	1,5	1,5	1,5
1,6	1,6	1,6	1,6	1,6	1,6	1,5	1,5	1,5	1,5
1,5	10	10	10	1,5	1,5	1,5	10	10	10
1,5	10	1,5	10	1,5	1,5	1,5	1,5	1,5	1,5
1,5	10	1,5	10	1,8	1,5	1,5	10	10	10
1,6	10	1,8	10	2,6	1,5	10	10	1,6	10
1,5	1,5	1,5	10	1,5	1,5	1,5	1,5	1,5	10
1,5	10	1,5	10	2,5	10	1,8	1,5	10	10
1,5	1,5	1,5	1,5	1,7	1,6	1,6	10	10	10
1,8	1,8	1,5	10	1,5	1,5	1,5	10	10	10
1,8	1,8	1,8	1,8	1,8	1,5	1,5	10	10	1,9
1,8	10	1,8	10	2,5	2,5	10	10	1,6	10
10	10	10	10	10	10	1,5	10	1,6	10
1,5	10	1,5	10	1,5	1,5	10	10	10	1,9
1,6	10	1,6	10	1,5	1,5	1,6	10	10	10
1,5	1,5	1,5	10	1,5	1,6	1,5	10	10	10
10	10	10	10	1,5	1,5	1,5	1,5	10	1,9
1,5	10	1,5	10	1,8	1,5	10	10	10	10
2,7	10	2,7	2,6	3,7	2,7	2,6	10	10	10
10	10	10	10	2,5	1,5	1,5	1,5	1,5	10
1,5	10	1,5	10	1,6	1,5	10	10	10	10
1,6	1,6	1,6	10	1,5	1,5	1,5	1,5	1,5	10
1,6	10	1,6	10	1,5	10	10	10	10	10
10	10	1,5	10	1,5	1,5	10	10	10	10
1,5	10	1,5	10	1,5	1,5	10	10	1,5	10
3,6	10	1,8	10	1,5	2,6	1,5	1,5	10	1,9
1,5	10	1,5	10	1,5	10	10	10	10	10
3,6	10	1,8	10	1,5	2,6	2,5	1,5	10	1,9
1,5	10	10	10	1,5	1,5	1,5	1,5	1,5	1,5
1,8	1,8	1,8	1,8	1,5	1,5	1,5	10	10	1,9
1,5	10	1,5	10	1,5	1,5	1,8	1,8	1,5	1,9
1	10	1,7	10	10	1,5	1,8	10	10	10
1,5	1,5	1,5	10	1,5	10	10	10	1,5	10
1,6	10	1,5	10	1,6	1,5	10	10	1,5	1,9
1,6	10	1,6	10	1,5	1,5	10	10	1,6	10
10	10	10	10	10	1,5	1,5	10	10	10
10	10	1,5	10	1,5	10	10	10	10	10
10	10	10	10	1,5	1,5	10	10	1,5	10
2,5	10	3,5	10	2,6	10	1,5	2,5	2,5	10
1,6	10	1,6	10	1,5	1,5	10	10	1,6	10

1,5	10	1,5	10	1,5	1,5	10	10	10	10
2,5	10	2,5	10	2,5	1,5	10	10	10	10
10	10	10	10	2,5	2,5	10	10	1,5	1,8
10	10	10	10	1,5	1,5	10	10	10	10
1,8	10	1,7	10	1,7	1,7	1,5	10	10	10
10	10	10	10	2,6	10	10	10	1,6	10
10	10	10	10	1,6	10	1,5	10	10	10
1,5	1,5	1,5	1,5	1,5	1,5	1,5	10	10	10
10	10	10	10	1,5	10	10	10	1,5	10
1,5	1,5	1,5	10	1,5	1,6	1,6	1,6	1,6	1,5
1,6	10	10	10	1,5	2,6	10	10	10	1,9
1,6	10	1,6	10	1,6	1,5	1,5	1,5	1,5	1,5
1,6	10	10	10	1,5	1,5	10	10	1,5	10
1,6	10	10	10	1,5	1,5	10	10	1,5	10
1,6	10	1,5	10	2,6	1,5	1,5	1,5	1,5	10
1,5	1,5	1,5	10	1,5	1,5	1,5	10	10	10
1,6	10	10	10	3,6	1,5	10	10	1,5	10
1,6	10	10	10	3,6	1,5	10	10	1,5	10
1,5	10	1,5	10	2,6	1,5	1,5	1,5	1,5	10
1,6	10	1,8	10	2,5	1,5	10	10	1,5	10
1,5	10	1,5	10	1,5	10	1,5	10	10	10
1,5	1,5	1,5	10	1,5	1,5	1,5	10	10	10
1,5	10	10	10	1,5	1,5	10	10	10	10
10	10	10	10	1,5	1,6	1,5	10	10	1,9
10	10	10	10	10	1,5	1,5	10	10	1,9
1,5	10	1,8	10	2,5	1,5	1,8	1,8	1,8	1,9
10	10	10	10	10	1,5	1,5	10	10	1,8
10	10	10	10	10	1,5	1,5	1,5	1,5	1,9
1,5	10	10	10	1,5	1,5	1,5	10	10	2,9
10	10	10	10	1,5	1,5	1,5	10	1,5	1,5
1,6	10	1,6	1,5	1,6	1,5	1,8	1,8	1,5	1,9
1,5	1,5	1,5	1,5	1,5	1,5	1,5	10	10	1,9
10	10	10	10	10	1,5	1,5	10	10	1,9
1,5	1,5	1,5	1,5	1,5	1,5	1,5	10	10	10
1,6	10	1,6	10	1,6	1,6	10	10	10	10
1,6	1,6	1,6	1,6	1,5	1,5	10	10	1,6	1,9
1,5	10	1,5	1,8	1,5	1,5	1,5	1,5	1,5	1,9
1,5	10	1,5	10	1,6	1,6	1,6	1,6	1,6	10
1,5	10	1,5	10	1,5	1,5	1,5	10	10	1,9
1,6	10	1,5	10	10	1,5	1,5	1,5	1,5	1,9
10	10	10	10	1,5	1,5	1,5	10	10	1,9
1,6	1,7	1,8	10	1,5	2,5	1,8	10	1,8	10
1,8	10	1,8	10	1,5	2,5	1,8	10	1,8	10
10	10	10	10	1,5	1,5	1,5	10	1,5	1,5
1,5	10	1,8	10	1,5	1,5	1,5	10	10	1,9
10	10	10	10	1,6	1,6	10	10	1,6	1,9

mopani wo	termites	nthwamak	jengelenge	full cream	sour milk	shop full cr	shop full cr	powdered	condensed
1,5	10	10	10	1,5	10	1,6	1,6	10	10
1,5	10	10	10	1,5	10	1,6	1,6	10	10
10	10	10	10	2,7	2,7	2,7	2,7	1,8	10
10	10	10	10	10	10	10	1,5	1,5	10
1,5	10	10	10	1,5	1,5	1,5	1,5	1,5	10
1,9	10	10	10	4,5	1,6	10	1,6	10	10
1,8	10	10	10	1,5	10	1,5	1,5	10	10
10	10	10	10	10	10	1,6	1,6	10	10
2,5	10	10	10	10	10	2,7	2,6	10	10
1,5	10	10	10	10	10	10	3,7	10	10
10	10	10	10	1,6	10	1,7	1,7	1,6	10
2,5	10	10	10	10	10	2,7	2,7	10	10
2,5	10	10	10	1,5	2,5	2,6	2,5	10	10
10	10	10	10	1,7	10	10	10	1,7	1,8
1,5	10	10	10	10	10	10	1,6	10	10
10	10	10	10	10	10	10	1,7	1,7	10
10	10	10	10	10	10	1,7	1,7	10	10
2,5	1,8	10	10	10	1,5	1,6	1,6	10	10
10	10	10	10	1,5	2,5	2,5	1,5	10	10
2,5	10	10	10	10	2,5	2,5	1,5	10	10
2,5	10	10	10	10	2,5	2,6	1,5	10	10
1,5	10	10	10	10	2,5	2,5	1,5	10	10
10	10	10	10	10	2,6	2,5	1,5	10	10
2,6	10	10	10	1,5	2,6	2,6	1,5	10	10
2,5	10	10	10	1,5	2,5	2,6	2,5	10	10
2,5	10	10	10	10	2,5	2,5	2,5	10	10
10	10	10	10	10	10	1,6	1,6	10	10
10	10	10	10	10	10	1,5	1,5	10	10
1,5	10	10	10	10	10	10	10	1,5	10
10	10	10	10	1,6	2,7	2,6	1,6	10	10
1,9	10	10	10	10	10	10	10	10	10
10	10	10	10	10	10	2,6	1,5	10	10
2,6	10	10	10	10	10	2,6	1,5	10	10
10	1,9	10	10	2,8	10	10	10	10	10
1,5	10	10	10	10	10	1,8	1,8	10	10
2,5	10	10	10	10	10	1,6	1,6	10	10
1,5	10	10	10	10	1,5	10	10	10	10
1,8	10	10	10	10	10	1,6	1,6	10	10
2,6	10	10	10	10	10	2,6	2,5	20	20
10	10	10	10	10	1,5	10	10	10	10
1,6	10	10	10	10	10	1,7	1,7	10	10
1,5	10	10	10	2,6	10	2,6	2,6	1,5	1,5
1,5	10	10	10	10	10	1,6	1,6	10	10
1,5	10	10	10	10	10	1,5	1,5	10	10
10	10	10	10	10	10	1,6	1,6	1,6	10
10	10	10	10	2,5	2,5	10	10	1,5	10

10	10	10	10	2,5	1,5	10	10	10	1,8
2,6	10	10	10	10	3,5	1,5	10	10	10
10	10	10	10	10	2,6	1,7	1,5	10	10
10	10	10	10	1,5	1,5	2,5	1,5	1,5	10
1,5	1,8	10	10	10	10	1,6	1,6	10	10
1,5	1,9	10	10	10	10	1,6	1,6	10	10
10	10	10	10	1,5	1,5	2,5	2,5	10	1,5
10	10	10	10	10	10	1,7	1,7	10	10
10	10	10	10	10	10	2,7	2,7	10	10
1,8	10	10	10	10	10	2,7	2,7	1,7	10
10	10	10	10	10	10	2,7	2,7	10	10
10	10	10	10	10	10	1,7	1,7	10	10
10	10	10	10	10	10	2,6	2,6	10	10
2,6	10	10	10	1,5	2,6	1,6	1,5	10	10
1,8	10	10	10	10	10	2,6	2,6	10	10
1,9	10	10	10	1,5	1,5	10	10	1,8	10
1,8	10	10	10	1,6	1,5	1,6	1,6	10	10
10	1,9	10	10	10	10	2,7	2,7	10	10
1,5	10	10	10	10	10	1,5	1,5	10	10
10	10	10	10	10	10	1,8	1,8	10	10
2,6	1,5	10	10	10	10	2,6	1,5	10	10
2,6	10	10	10	10	2,9	2,6	1,5	10	10
10	10	10	10	10	2,6	2,6	1,5	10	10
1,5	10	10	10	10	10	1,5	1,5	10	10
1,5	10	10	10	10	10	1,5	1,5	10	10
10	10	10	10	10	10	1,7	1,7	10	10
1,5	10	10	10	10	10	10	1,6	10	10
1,9	10	10	10	1,6	1,6	10	10	10	10
2,6	10	10	10	2,7	1,5	2,6	2,6	10	10
1,6	10	10	10	10	10	1,5	1,5	10	10
1,5	10	10	10	10	10	1,5	1,5	10	10
1,5	10	10	10	10	10	1,5	1,5	10	10
1,5	10	10	10	1,5	10	1,5	1,5	1,5	10
1,8	10	10	10	10	10	1,6	1,6	10	10
10	10	10	10	10	10	1,6	1,6	10	10
2,6	10	10	10	10	2,6	2,5	1,5	10	10
10	10	10	10	10	10	2,6	1,5	10	10
10	10	10	10	1,5	1,5	10	10	10	10
1,9	10	10	10	10	10	3,7	3,7	2,8	10
1,5	1,5	10	10	1,5	10	1,5	1,5	1,5	10
1,8	10	10	10	10	10	1,5	1,5	10	1,5
1,5	10	10	10	10	10	1,5	1,5	10	10
1,5	10	10	10	10	10	1,5	1,5	10	10
1,8	10	10	10	10	10	1,5	1,5	1,5	10
1,5	10	10	10	10	10	1,5	1,5	10	10
1,5	10	10	10	10	10	1,5	1,5	10	10
1,5	1,5	10	10	10	2,5	2,6	1,5	10	10

10	10	10	10	1,6	1,6	1,6	10	1,5	10
1,5	10	10	10	10	10	1,5	1,5	10	10
1,5	10	10	10	10	10	1,6	1,6	10	10
10	10	10	10	10	10	2,7	2,7	10	10
2,5	1,5	10	10	10	10	1,5	1,5	10	10
10	10	10	10	10	10	10	10	10	10
10	10	10	10	10	2,5	2,6	1,5	1,5	10
10	10	10	10	10	10	1,5	1,6	10	10
1,6	10	10	10	10	10	1,6	1,6	10	10
1,8	10	10	10	1,5	1,5	3,5	3,5	10	10
1,8	10	10	10	1,5	1,6	1,6	1,6	10	10
10	10	10	10	1,5	1,5	10	10	1,5	10
10	10	10	10	10	10	1,7	1,7	10	10
10	10	10	10	1,5	1,5	1,5	1,5	10	1,5
1,8	10	10	10	1,5	1,6	1,6	1,6	10	10
10	10	10	10	2,5	2,6	2,6	1,5	10	10
1,8	10	10	10	10	10	1,8	1,8	10	10
10	10	10	10	10	10	1,6	1,6	10	10
1,5	10	10	10	10	10	1,6	1,6	10	10
1,9	10	10	10	1,6	1,6	10	10	1,5	1,8
2,5	10	10	10	1,5	2,6	2,6	1,5	10	10
1,5	10	10	10	10	10	1,5	1,5	10	10
1,5	10	10	10	1,7	1,7	1,7	1,7	1,5	10
1,5	10	10	10	1,7	1,7	1,7	1,7	1,5	10
1,5	10	10	10	1,7	1,7	1,7	1,7	1,5	10
3,5	10	10	10	3,8	3,8	1,7	1,7	10	10
10	10	10	10	2,5	2,5	1,5	1,5	10	10
1,5	10	10	10	10	10	10	10	10	10
10	10	10	10	1,5	1,5	10	10	1,5	10
2,5	10	10	10	10	2,9	2,6	2,5	10	10
10	10	10	10	1,5	1,5	1,5	1,6	10	10
10	10	10	10	2,6	10	2,6	2,6	1,5	1,5
2,6	10	10	10	10	2,9	1,5	1,5	10	10
10	10	10	10	1,5	1,5	1,6	1,6	10	10
1,8	10	10	10	10	10	1,6	1,6	10	10
1,9	10	10	10	1,9	1,9	1,5	1,5	10	10
1,7	10	10	10	10	10	1,5	1,5	10	10
1,8	10	10	10	2,8	2,8	1,5	1,5	10	10
1,5	10	10	10	10	10	1,5	1,5	1,5	10
1,5	10	10	10	1,5	1,5	1,5	1,5	1,8	10
10	10	10	10	1,5	1,5	1,6	1,6	10	10
10	10	10	10	1,5	10	1,6	1,6	1,5	10
10	10	10	10	10	10	1,7	1,7	10	10
10	10	10	10	2,6	2,6	2,6	2,6	1,5	10
2,5	10	10	10	2,5	2,6	2,6	1,5	10	10
10	10	10	10	2,6	2,6	2,6	2,6	1,8	1,8
2,6	2,5	10	10	2,5	2,6	2,6	2,5	10	10

10	10	10	10	1,5	1,5	1,5	1,5	10	10
1,5	10	10	10	10	10	1,6	1,6	10	10
10	10	10	10	1,5	1,5	2,5	2,5	10	10
10	10	10	10	1,5	1,5	2,5	2,5	10	10
1,5	10	10	10	1,5	1,5	1,6	1,6	10	10
1,5	10	10	10	2,4	2,4	2,4	2,4	1,5	10
2,6	10	10	10	1,5	2,6	2,6	2,5	10	10
1,5	10	10	10	1,6	1,6	1,6	1,6	1,5	1,5
10	10	10	10	2,9	2,9	1,7	1,7	10	10
1,5	10	10	10	1,5	1,5	1,5	1,5	1,5	10
10	10	10	10	1,7	1,7	10	1,6	10	10
10	10	10	10	1,7	1,7	10	1,6	10	10
10	10	10	10	1,6	1,6	10	1,5	10	10
10	10	10	10	1,6	1,6	1,6	1,6	1,5	10
1,5	10	10	10	2,6	2,6	2,6	2,6	2,7	10
10	10	10	10	2,5	2,5	2,5	2,5	10	1,5
10	10	10	10	2,7	2,7	2,7	2,7	10	10
10	10	10	10	2,7	2,6	2,6	2,6	10	10
2,6	10	10	10	10	2,5	2,6	2,5	10	10
10	10	10	10	10	10	1,6	1,6	1,5	10
10	10	10	10	10	10	1,7	1,7	10	10
10	10	10	10	10	10	2,6	2,6	10	10
10	10	10	10	10	10	1,6	1,6	10	10
2,5	10	10	10	10	10	1,5	1,5	10	10
2,5	10	10	10	10	10	1,5	1,5	10	10
10	10	10	10	1,5	1,5	1,5	1,5	10	10
1,5	10	10	10	1,5	10	1,6	1,6	10	1,5
10	10	10	10	10	10	1,6	2,7	1,6	1,6
1,9	10	10	10	1,5	1,5	1,5	1,5	10	10
2,6	10	10	10	1,5	2,5	2,5	2,5	10	10
1,8	10	10	10	10	10	1,5	1,5	10	10
10	10	10	10	10	2,6	10	1,5	10	10
1,6	10	10	10	10	10	1,5	1,5	10	10
2,5	10	10	10	2,5	2,6	2,6	2,6	10	10
10	10	10	10	1,5	1,5	1,5	1,5	10	10
10	10	10	10	10	1,5	1,5	1,5	10	10
1,9	10	10	10	10	1,8	1,5	1,6	10	10
10	10	10	10	1,6	1,6	1,6	1,6	1,5	10
1,5	10	10	10	2,6	2,6	2,6	2,6	10	10
1,5	10	10	10	2,6	2,6	2,6	2,6	10	10
10	10	10	10	10	1,5	1,5	1,6	10	10
1,5	10	10	10	1,5	1,5	10	3,5	10	10
1,8	10	10	10	10	10	1,6	1,6	10	10
1,5	10	10	10	1,5	1,5	1,5	1,5	1,5	1,5
1,5	1,8	10	10	1,5	1,5	1,5	1,5	10	1,5
10	10	10	10	10	10	1,7	1,7	10	10
1,5	10	10	10	10	10	2,7	2,7	10	10

1,8	10	10	10	10	10	1,5	1,5	10	10
10	10	10	10	1,6	1,6	1,6	1,6	1,5	10
1,9	10	10	10	10	1,5	1,5	1,5	10	10
2,6	10	10	10	2,5	2,6	2,6	2,6	10	10
2,6	10	10	10	2,5	2,6	10	10	10	10
10	10	10	10	1,5	1,5	1,5	1,5	10	10
10	10	10	10	1,6	2,6	1,6	1,6	10	10
1,8	10	10	10	10	10	1,6	1,6	10	10
10	10	10	10	10	10	1,5	1,5	10	10
10	10	10	10	10	10	1,6	1,6	10	10
1,5	10	10	10	1,5	1,5	1,5	1,5	10	10
10	10	10	10	1,5	1,5	1,5	1,5	10	10
10	10	10	10	1,5	1,6	1,6	1,5	10	1,8
10	10	10	10	10	1,9	10	1,5	10	10
1,5	10	10	10	1,5	1,5	1,5	1,5	10	10
1,9	10	10	10	10	1,5	1,5	1,6	10	10
1,5	10	10	10	1,5	1,5	1,5	1,5	10	10
10	10	10	10	1,5	1,6	1,5	1,5	10	10
10	10	10	10	10	1,5	1,5	1,5	10	10
1,8	10	10	10	1,5	1,5	1,7	1,7	10	10
1,6	10	10	10	10	10	1,6	1,6	10	10
1,5	10	10	10	1,5	1,5	1,5	1,5	1,5	10
1,5	10	10	10	1,5	1,5	1,5	1,5	1,5	10
2,5	10	10	10	10	10	10	1,6	10	10
1,8	10	10	10	10	10	10	2,5	10	10
10	10	10	10	1,5	1,5	1,5	1,5	10	10
10	10	10	10	10	2,6	2,5	1,5	10	10
10	10	10	10	10	10	1,5	1,6	10	10
10	10	10	10	2,5	2,5	1,6	1,6	10	10
1,8	10	10	10	1,5	1,5	1,7	1,7	10	10
1,5	10	10	10	10	10	1,5	1,5	10	10
1,5	10	10	10	10	10	1,6	1,6	10	10
10	10	10	10	1,5	1,5	1,5	1,5	10	10
10	10	10	10	10	10	2,5	2,5	10	10
10	10	10	10	1,8	1,8	10	1,6	10	10
10	10	10	10	10	1,5	1,6	1,6	10	10
10	10	10	10	2,6	2,6	2,6	2,6	10	10
1,8	10	10	10	2,6	1,5	2,7	2,7	10	10
10	10	10	10	10	10	1,5	1,5	10	10
1,5	10	10	10	1,5	1,5	1,5	1,5	10	10
10	10	10	10	10	10	1,6	1,6	10	10
10	10	10	10	10	10	1,5	1,5	10	10
1,8	10	10	10	10	10	1,7	1,7	10	10
2,8	10	10	10	10	10	10	3,5	10	10
1,8	10	10	10	10	10	2,7	2,7	10	10
1,6	10	10	10	10	10	2,7	2,7	10	10
10	10	10	10	2,6	2,5	2,5	2,5	10	10

10	10	10	10	2,6	2,5	2,5	2,5	10	10
10	10	10	10	10	10	1,5	1,6	10	10
1,5	10	10	10	10	10	1,6	1,6	10	10
10	10	10	10	10	10	1,5	1,6	1,6	10
10	10	10	10	10	1,5	1,5	1,5	10	10
10	10	10	10	10	1,5	1,6	1,6	10	10
10	10	10	10	10	10	1,6	1,6	10	10
10	10	10	10	10	1,5	1,5	1,5	10	10
10	10	10	10	10	10	1,6	1,6	10	10
10	10	10	10	10	1,5	1,5	1,6	10	10
1,5	10	10	10	2,5	2,5	2,5	2,5	1,5	1,5
10	10	10	10	1,5	1,5	1,6	1,6	10	10
1,8	10	10	10	10	10	1,7	1,7	10	10
1,8	10	10	10	10	10	1,7	1,7	10	10
1,5	10	10	10	10	10	2,6	2,6	10	10
10	1,8	10	10	10	10	1,5	1,5	10	10
10	2,5	10	10	1,5	2,5	1,5	1,5	10	10
10	2,5	10	10	1,5	2,5	1,5	1,5	10	10
1,8	10	10	10	10	10	1,6	1,6	10	10
1,8	1,8	10	10	10	10	1,7	1,7	10	10
10	1,8	10	10	10	10	10	10	10	10
10	10	10	10	10	10	1,6	1,6	10	10
10	10	10	10	10	10	2,7	2,7	2,7	10
10	10	10	10	10	10	10	10	10	10
10	10	10	10	10	10	10	10	10	10
10	1,5	10	10	10	10	1,5	1,5	1,5	10
1,5	10	10	10	10	10	2,6	2,6	10	10
1,5	10	10	10	10	10	1,6	1,6	10	10
2,5	10	10	10	10	10	1,6	1,6	10	10
2,5	10	10	10	10	10	1,6	1,6	10	10
1,8	1,9	10	10	10	10	1,6	1,6	1,5	1,5
1,8	10	10	10	10	10	1,6	1,6	10	10
1,9	10	10	10	10	1,5	10	10	1,6	1,5
1,5	10	10	10	1,5	1,5	1,5	1,5	10	10
1,5	1,8	10	10	1,5	1,5	1,5	1,5	10	10
10	10	10	10	10	10	10	10	1,5	10
1,5	10	10	10	10	10	1,5	1,5	10	10
1,9	10	10	10	10	1,5	1,6	1,6	1,6	10
1,5	10	10	10	1,6	1,6	1,6	1,6	10	10
1,5	1,5	10	10	10	10	1,5	1,5	10	10
10	10	10	10	10	10	1,6	1,6	10	10
1,5	10	10	10	10	10	1,5	2,5	10	10
10	10	10	10	1,8	1,8	1,5	1,5	10	10
10	10	10	10	1,8	1,8	1,5	1,5	10	10
10	10	10	10	1,6	1,6	1,6	1,6	10	10
1,5	10	10	10	10	2,6	2,6	2,6	1,5	10
1,5	10	10	10	1,4	1,5	1,5	1,5	10	10

10	10	10	10	2,6	2,6	2,6	2,6	1,6	1,5
1,5	10	10	10	1,6	1,5	1,5	10	1,5	10
1,8	10	10	10	1,5	1,5	1,5	1,5	1,5	10
1,5	10	10	10	10	10	1,5	1,5	10	10
1,9	10	10	10	1,5	1,5	1,5	1,5	1,5	1,5
10	10	10	10	10	10	10	10	10	10
10	10	10	10	1,5	1,5	1,5	1,5	1,5	10
1,5	10	10	10	1,5	1,5	1,5	1,5	1,6	10
1,5	10	10	10	1,5	1,5	1,5	1,5	10	10
1,5	10	10	10	1,5	1,5	1,5	1,5	10	10
1,5	10	10	10	10	10	10	1,7	10	10
10	10	10	10	1,5	1,5	1,5	1,5	1,5	1,5
10	10	10	10	10	10	10	10	10	10
1,7	10	10	10	1,6	1,6	1,5	1,6	10	10
10	10	10	10	1,5	1,5	1,5	1,5	1,5	10
1,5	10	10	10	1,5	1,5	1,5	1,5	10	10
1,5	10	10	10	10	10	1,5	1,5	10	10
1,8	10	10	10	10	10	10	1,7	10	10
1,6	10	10	10	1,5	1,5	1,5	1,5	1,5	10
10	10	10	10	10	10	1,6	1,6	1,6	10
10	10	10	10	10	10	1,5	1,5	10	10
10	10	10	10	10	10	2,7	2,7	10	10
1,8	10	10	10	10	10	1,7	1,7	10	10
1,6	10	10	10	10	10	1,7	1,7	10	10
1,5	10	10	10	1,5	1,5	1,5	1,5	10	1,5
1,5	1,5	10	10	1,5	1,5	1,5	1,5	1,5	1,5
1,6	10	10	10	10	10	1,7	1,7	10	10
1,5	10	10	10	1,5	1,5	1,5	1,5	10	10
10	10	10	10	10	10	2,7	2,7	10	10
1,5	10	10	10	10	10	1,6	1,6	10	10
2,5	10	10	10	10	10	1,5	1,5	10	10
1,5	10	10	10	10	10	1,5	1,5	10	1,8
1,5	10	10	10	10	10	1,5	1,5	10	10
1,5	10	10	10	10	10	1,5	1,5	10	10
1,8	10	10	10	10	10	1,7	1,7	10	10
2,5	10	10	10	10	10	1,5	1,5	10	10
10	10	10	10	1,6	10	1,6	1,6	10	10
10	10	10	10	10	10	2,7	2,7	10	10
1,8	10	10	10	10	10	1,7	1,7	10	10
1,8	10	10	10	10	10	1,7	1,7	10	10
10	10	10	10	10	10	1,5	1,5	10	10
1,5	10	10	10	10	10	1,5	1,5	10	10
10	10	10	10	1,5	1,5	1,5	10	10	10
10	10	10	10	10	10	1,5	1,5	1,6	1,6
1,5	1,5	10	10	1,5	1,5	1,5	1,5	10	10
1,6	10	10	10	1,6	1,6	1,6	1,6	10	10
1,5	10	10	10	10	10	1,5	1,5	10	10

1,5	10	10	10	10	10	1,5	1,5	10	10
1,5	10	10	10	10	10	1,5	1,5	1,5	1,5
1,5	10	10	10	1,5	1,5	1,5	1,5	10	10
2,6	10	10	10	2,6	10	2,5	2,6	10	10
1,5	10	10	10	1,6	1,6	1,6	1,6	10	10
1,5	10	10	10	1,9	1,9	1,9	1,7	1,6	1,9
1,5	10	10	10	10	10	10	1,5	10	10
1,5	10	10	10	10	10	1,5	1,5	10	10
1,6	10	10	10	10	10	1,5	1,5	10	10
3,6	10	10	10	3,6	2,6	2,6	10	10	10
1,8	10	10	10	10	10	1,7	1,7	10	10
10	10	10	10	10	10	1,5	1,5	10	10
1,5	10	10	10	10	10	1,5	1,5	10	10
1,5	10	10	10	10	10	1,5	1,5	10	10
1,5	10	10	10	10	10	1,5	1,5	10	10
1,8	10	10	10	10	10	10	1,5	10	10
1,8	10	10	10	10	10	10	1,5	10	10
10	10	10	10	10	10	1,5	1,5	10	10
1,5	10	10	10	1,6	1,6	1,6	1,6	10	10
1,5	10	10	10	10	10	1,5	1,5	1,5	10
1,5	10	10	10	1,5	1,5	2,6	1,5	10	10
10	10	10	10	10	10	1,7	1,7	10	10
1,5	10	10	10	10	10	10	1,5	10	10
1,5	10	1,5	10	1,5	1,5	1,5	1,5	1,5	1,5
1,8	10	10	10	10	10	10	1,5	10	10
1,6	10	10	10	10	10	1,7	1,7	10	10
1,5	10	10	10	10	10	1,5	1,5	10	10
1,5	10	10	10	10	10	10	2,5	10	10
10	10	10	10	10	10	1,5	1,5	10	10
1,5	10	10	10	10	10	1,5	1,5	10	10
1,8	10	10	10	10	10	10	1,6	10	10
1,6	10	10	10	10	10	2,5	2,5	10	10
1,5	10	10	10	10	10	1,6	1,6	10	10
1,9	1,5	10	10	10	10	1,6	1,6	10	10
1,8	10	10	10	10	10	2,6	2,6	10	10
1,5	10	10	1,5	1,5	1,5	1,5	1,5	10	10
10	10	10	10	10	10	10	10	10	10
1,5	10	10	10	10	10	1,6	1,6	10	10
10	10	10	10	1,5	1,5	1,5	1,5	10	10
1,5	10	10	10	1,5	1,5	1,5	1,5	1,5	1,5
10	10	10	10	1,6	10	2,6	2,5	10	10
1,5	10	10	10	10	10	1,5	1,5	1,6	10
10	10	10	10	2,6	10	2,5	2,5	10	10
10	10	10	10	10	10	1,5	1,5	1,6	10
1,6	10	10	10	10	10	2,6	3,6	10	10
1,5	10	10	10	10	10	1,6	1,6	10	10
1,5	10	10	10	10	10	1,5	1,5	10	10

10	10	10	10	10	10	1,7	1,7	10	10
1,6	10	10	10	10	10	10	1,7	10	10
10	10	10	10	10	10	1,6	1,6	10	10
10	10	10	10	10	10	1,7	1,7	10	10
10	10	10	10	10	10	1,7	1,7	10	10
10	10	10	10	10	10	1,7	1,7	10	10
2,5	10	10	10	10	10	1,5	1,5	10	10
1,5	10	10	10	2,5	2,5	2,5	2,5	10	10
1,5	10	10	10	10	10	1,5	1,5	10	10
10	10	10	10	2,7	2,7	2,7	2,7	10	10
1,5	10	10	10	1,5	1,5	1,5	1,5	10	10
10	10	10	10	10	10	1,5	1,5	1,5	1,6
1,6	10	10	10	10	10	1,5	1,5	1,5	10
10	10	10	10	10	10	10	1,7	10	10
1,6	10	10	10	10	10	1,5	1,5	1,5	10
1,5	10	10	10	10	10	1,5	1,5	10	10
10	10	10	10	1,6	1,6	1,6	1,6	1,6	1,5
10	10	10	10	10	10	1,5	1,5	1,8	1,5
1,5	10	10	10	1,5	1,5	1,5	1,5	10	10
10	10	10	10	1,6	1,6	1,6	1,6	1,6	1,6
10	10	10	10	10	10	1,7	1,7	10	10
10	10	10	10	10	10	2,6	2,6	10	10
10	10	10	10	1,5	1,5	1,5	1,5	10	10
10	10	10	10	1,8	1,8	1,5	1,5	1,6	1,8
1,5	10	10	10	1,9	1,9	1,5	1,5	10	10
10	10	10	10	1,5	1,5	1,5	1,5	10	10
1,6	10	10	10	1,5	1,5	1,5	1,5	1,5	1,5
1,9	10	10	10	2,5	2,5	2,5	2,5	1,5	10
10	10	10	10	1,6	1,6	1,6	1,6	10	10
10	10	10	10	1,6	1,6	1,6	1,6	10	10
1,6	10	10	10	1,5	1,5	1,5	1,5	1,5	1,5
1,5	10	10	10	1,8	1,8	1,5	1,5	10	10
1,5	10	10	10	1,5	1,5	1,5	1,5	10	10
1,8	10	10	10	10	10	1,7	1,7	10	10
1,5	10	10	10	10	10	1,5	1,5	10	10
10	10	10	10	10	10	1,7	1,7	10	10
1,9	10	10	10	2,6	2,6	2,6	2,6	10	10
1,9	10	10	10	2,6	2,6	2,6	2,6	10	10
1,5	10	10	10	1,5	1,5	1,5	1,5	1,5	10
1,9	10	10	10	1,5	1,5	1,5	1,5	10	10
10	10	10	10	1,5	1,5	1,5	1,5	1,5	10
10	10	10	10	1,5	1,5	1,5	1,5	1,5	1,5
1,5	10	10	10	1,5	1,5	1,5	1,5	1,5	10
10	10	10	10	2,5	2,5	2,5	2,5	1,5	10
10	10	10	10	2,5	1,5	10	10	10	10
1,5	1,5	10	10	1,5	1,5	1,5	1,5	1,5	1,5
10	10	10	10	2,6	2,6	2,6	2,6	10	10

1,5	10	10	10	10	10	1,5	1,5	10	10
1,5	10	10	10	10	10	1,5	1,5	10	10
10	10	10	10	1,5	1,5	1,5	1,5	10	10
1,5	10	10	10	1,5	1,5	1,5	1,5	10	1,6
1,5	10	10	10	1,5	1,5	1,5	1,5	10	10
1,5	10	10	10	10	10	1,7	1,7	10	10
10	10	10	10	10	10	10	1,7	10	10
1,5	10	10	10	1,5	1,5	1,6	1,6	1,5	1,5
1,5	10	10	10	1,5	1,5	1,6	1,6	1,5	1,5
1,6	10	10	10	10	1,6	1,6	10	10	10
1,5	10	10	10	1,5	1,5	1,5	1,5	10	10
1,5	10	10	10	10	10	1,5	1,5	10	10
2,5	10	10	10	2,5	2,5	2,5	2,5	2,5	2,5
10	10	10	10	1,5	1,5	1,5	1,5	10	10
10	10	10	10	10	10	1,7	1,7	10	10
10	10	10	10	10	10	10	1,5	1,5	10
10	10	10	10	3,7	1,9	1,9	1,9	1,9	1,9
1,5	10	10	10	1,9	1,9	1,5	1,5	10	10
1,8	10	10	10	2,8	10	2,5	2,7	10	10
10	10	10	10	1,6	10	1,7	1,5	1,7	2,6
1,5	10	10	10	10	10	1,5	1,5	10	10
10	10	10	10	10	10	1,5	1,5	1,5	10
1,5	10	10	10	10	1,9	1,5	1,5	10	10
1,9	10	10	10	1,6	1,6	1,6	1,5	1,5	1,5
10	10	10	10	10	10	10	1,5	1,5	10
10	10	10	10	2,6	10	2,6	2,6	1,7	10
10	10	10	10	1,5	1,5	1,5	1,5	10	10
10	10	10	10	2,6	2,6	2,6	2,5	10	10
1,5	10	10	10	1,5	1,5	1,5	1,5	1,5	1,5
10	10	10	10	1,6	1,5	1,5	1,5	10	1,5
10	10	10	10	1,5	1,5	1,5	1,5	10	10
1,5	10	10	10	10	10	10	10	10	10
1,9	10	10	10	1,5	1,5	1,5	1,5	10	1,8
1,8	10	10	10	1,5	1,5	1,5	1,5	10	10
1,9	10	10	10	1,5	1,5	1,5	1,5	10	1,8
1,5	10	10	10	1,5	1,5	1,5	10	10	1,5
1,5	10	10	10	10	10	1,5	1,5	10	10
1,5	10	10	10	10	10	1,5	1,5	10	10
10	10	10	10	10	10	10	1,5	10	10
1,5	10	10	10	1,5	1,5	1,5	1,5	1,5	1,5
1,5	10	10	10	1,5	1,5	1,5	1,5	1,5	1,5
10	10	10	10	10	10	1,7	1,7	10	10
1,5	10	10	10	10	10	10	10	10	10
10	10	10	10	1,5	1,5	1,5	1,5	10	10
1,8	10	10	10	10	10	1,7	1,7	10	10
1,5	10	10	10	3,5	3,5	2,5	10	10	10
10	10	10	10	10	10	1,7	1,7	10	10

10	10	10	10	1,5	1,5	1,5	1,5	10	10
2,5	10	10	10	2,5	2,5	2,5	2,5	10	2,5
1,5	10	10	10	10	10	2,7	2,7	10	10
1,5	10	10	10	1,6	1,6	1,6	1,6	10	10
1,5	10	10	10	1,9	1,9	1,5	1,5	10	10
10	10	10	10	10	10	2,7	2,7	10	10
10	10	10	10	2,6	2,6	2,6	2,6	1,5	2,5
1,5	10	10	10	10	10	10	1,5	10	10
1,5	10	10	10	10	10	1,7	1,7	10	10
1,5	10	10	10	1,5	1,5	1,5	1,5	1,5	10
1,9	10	10	10	2,5	2,5	2,5	2,5	10	10
1,5	10	10	10	1,5	1,5	1,5	1,5	10	10
10	10	10	10	10	10	1,7	1,7	10	10
10	10	10	10	10	10	1,7	1,7	10	10
1,5	10	10	10	1,5	1,5	1,5	1,5	10	10
1,5	10	10	10	1,7	1,7	1,7	1,7	2,7	10
10	10	10	10	10	10	2,5	2,5	10	10
10	10	10	10	10	10	2,5	2,5	10	10
1,5	10	10	10	10	10	1,5	1,5	1,5	10
2,5	1,5	10	10	1,5	1,5	1,5	1,5	10	10
10	10	10	10	1,6	1,6	1,6	1,6	10	10
1,5	10	10	10	1,7	1,7	1,7	1,7	2,7	10
10	10	10	10	10	10	10	10	10	10
1,8	1,9	10	10	10	10	10	10	10	10
1,9	10	10	10	10	10	10	10	10	10
1,9	10	10	10	10	10	1,6	1,6	10	10
1,8	10	10	10	10	10	10	10	10	10
10	10	10	10	1,5	1,5	1,5	1,5	1,5	10
10	10	10	10	10	10	10	10	10	10
1,5	10	10	10	10	10	1,5	1,5	10	10
1,5	1,5	10	10	10	10	1,5	1,5	10	1,5
1,5	1,9	10	10	1,9	1,9	1,5	1,5	10	10
1,9	10	10	10	10	10	1,6	10	10	10
1,5	10	10	10	10	10	1,5	1,5	10	10
1,5	1,6	10	10	1,5	1,5	1,5	1,6	10	10
1,5	10	10	10	10	10	1,7	1,7	10	10
1,9	10	10	10	1,5	1,5	1,5	1,5	10	1,5
1,6	1,6	10	10	1,6	1,6	1,6	1,6	1,6	1,5
10	10	10	10	1,6	1,6	1,6	1,6	10	10
1,5	10	10	10	10	10	1,5	1,5	1,5	1,5
1,5	10	10	10	10	10	1,5	1,5	10	1,5
10	10	10	10	10	1,5	1,5	10	10	10
10	10	10	10	10	10	1,5	1,5	10	10
1,5	10	10	10	10	10	1,5	1,5	10	10
10	10	10	10	10	10	1,6	1,6	10	10
1,8	10	10	10	10	10	1,7	1,7	10	10

danone	coffee cre	ice-cream	cheese	boiled egg	fried eggs	scrambele	mayo	inkomazi	baked bea
1,7	1,7	1,8	1,5	1,5	1,6	10	1,5	1,5	1,5
1,7	1,7	1,8	1,5	1,5	1,6	10	1,5	1,5	1,5
1,7	1,7	1,8	10	1,7	1,7	10	1,8	2,7	1,7
1,5	1,5	1,5	10	1,6	1,6	10	1,8	1,6	10
1,5	2,6	1,5	10	1,5	2,5	10	1,5	2,5	10
2,7	10	1,9	1,6	10	1,6	10	10	1,6	1,5
1,5	1,6	10	10	1,5	1,5	10	1,5	10	10
1,5	1,6	10	10	1,5	1,5	10	10	1,6	1,6
1,6	1,7	1,6	1,6	10	10	10	1,5	2,6	2,5
1,7	1,6	1,5	10	10	1,5	10	1,8	10	10
1,7	1,7	1,8	1,6	1,7	1,5	10	1,5	1,6	1,5
1,5	1,6	1,5	1,5	1,6	2,6	2,6	2,6	2,6	2,6
1,6	1,6	1,8	1,5	1,5	1,6	2,6	10	2,6	2,5
1,5	10	1,8	1,8	1,8	1,5	10	1,8	1,5	10
1,7	1,7	1,7	1,8	1,5	1,5	10	10	1,6	1,5
1,5	1,5	1,5	1,6	10	2,5	10	1,6	1,5	1,5
1,5	1,5	1,5	1,6	10	1,5	10	1,6	1,5	1,5
1,6	1,5	1,8	1,8	1,5	1,5	2,6	1,5	2,5	2,5
10	1,5	1,8	1,8	1,5	2,5	2,6	1,8	2,6	2,5
1,5	1,5	1,8	1,8	1,5	2,5	2,5	1,8	2,6	1,5
1,6	1,5	1,8	1,5	1,5	2,5	2,5	1,8	2,6	1,5
1,7	1,6	1,8	1,5	2,5	2,5	2,6	1,5	2,5	2,5
1,6	2,6	1,8	1,5	1,5	2,6	2,6	1,8	2,6	2,5
1,5	1,6	1,8	1,8	1,5	2,6	2,6	1,8	2,5	2,6
1,7	1,6	1,5	1,5	1,5	2,6	2,6	1,8	2,6	1,5
1,6	1,5	1,8	1,5	1,5	2,5	2,6	1,8	2,6	2,5
2,7	1,6	1,5	10	1,6	1,6	10	1,5	1,6	1,5
2,7	1,7	1,6	1,5	1,5	1,7	10	1,5	1,5	1,5
1,6	1,5	1,5	10	1,5	1,5	1,5	10	10	2,5
3,6	1,5	1,8	10	10	1,5	10	1,7	2,7	1,5
1,5	1,6	10	10	10	1,5	10	10	1,7	1,5
1,6	1,6	1,5	1,5	1,5	1,6	1,6	1,5	2,5	2,5
1,5	1,5	1,5	10	2,5	2,5	2,5	1,5	1,6	2,6
2,8	1,7	1,8	1,8	10	1,5	10	1,8	2,8	10
1,8	1,5	10	10	10	1,5	10	1,8	1,8	1,5
2,6	1,6	1,8	10	10	2,6	10	1,8	2,6	2,8
10	10	1,8	10	1,5	1,6	1,5	1,8	1,5	10
1,7	10	10	10	1,5	1,5	10	10	1,5	1,5
1,5	1,6	10	10	1,6	1,6	1,6	1,5	2,6	1,5
1,7	1,7	1,5	10	1,6	1,6	10	1,5	1,5	10
3,7	1,6	1,5	10	1,7	1,6	1,6	1,6	1,7	1,7
1,6	1,5	1,5	10	1,5	1,5	10	1,5	2,6	1,5
2,7	10	1,8	10	2,5	1,6	10	1,8	10	1,5
1,6	1,5	1,5	10	10	1,6	10	1,5	1,5	1,5
1,6	1,6	1,8	10	10	1,5	10	1,8	1,5	1,8
2,6	10	10	10	10	1,5	10	1,8	1,5	10

2,6	1,6	10	10	10	1,6	10	1,8	10	1,5
1,8	1,7	10	1,5	10	1,7	10	10	1,5	1,5
1,6	1,5	1,8	10	1,5	2,5	2,6	1,5	2,6	1,6
1,7	1,7	1,5	1,6	1,5	1,5	10	1,5	1,6	1,5
1,6	1,7	1,8	1,5	2,6	1,7	10	1,5	1,6	1,5
1,7	1,6	1,8	10	10	1,6	10	1,8	1,6	10
1,6	10	1,5	10	1,5	1,5	10	10	2,5	1,5
1,8	1,8	1,5	10	1,6	1,6	10	1,8	1,6	1,6
2,7	1,7	2,8	10	10	1,6	10	1,5	1,6	1,5
2,7	1,7	2,8	10	1,6	1,6	10	10	1,7	1,8
2,7	1,7	1,8	10	10	1,6	10	1,8	1,6	1,5
2,7	1,7	1,8	10	10	1,7	10	1,8	2,7	1,8
2,7	1,7	2,8	10	1,5	1,6	10	1,8	2,7	1,5
1,6	1,5	1,5	1,5	1,5	2,6	10	1,5	1,6	1,6
1,7	1,5	1,8	10	1,8	1,5	10	1,8	10	10
2,8	1,6	1,8	10	1,8	1,5	10	1,8	1,5	10
3,7	1,6	1,5	10	2,6	1,6	10	1,8	1,5	10
3,7	2,7	1,7	10	1,7	2,7	10	1,6	1,7	1,6
1,5	1,7	10	10	10	1,6	10	1,8	1,5	1,5
2,8	10	1,9	10	10	1,5	10	1,8	1,5	1,5
1,6	1,5	1,8	10	2,5	2,6	2,6	1,5	2,6	2,5
1,6	1,6	1,5	1,8	1,5	1,5	1,8	10	1,5	2,5
1,6	1,6	1,5	1,8	1,5	2,6	2,6	1,5	10	2,5
1,5	1,5	1,8	10	1,8	1,5	10	1,8	1,5	1,5
1,5	1,5	1,8	10	1,8	1,5	10	10	1,5	1,5
2,7	1,6	10	10	1,6	1,6	10	1,8	2,6	1,5
1,5	1,6	1,8	10	10	1,5	10	1,5	1,5	1,5
2,6	1,7	1,8	10	10	1,5	10	1,8	10	10
2,7	1,7	1,5	1,5	1,6	1,5	10	1,6	1,5	1,6
1,7	1,7	10	10	1,6	2,6	10	1,8	1,8	10
1,8	10	1,8	10	1,8	1,5	10	2,8	2,5	10
1,8	10	1,8	10	1,8	1,5	10	1,8	2,5	10
2,6	10	10	10	1,5	2,5	10	1,5	1,8	1,5
1,5	1,6	1,7	1,8	2,6	1,6	10	1,5	1,6	1,5
2,7	1,7	1,5	1,5	10	1,5	10	1,5	1,6	1,5
1,6	2,6	1,5	10	1,5	1,5	1,5	1,5	2,6	2,5
1,6	1,6	1,5	10	1,5	2,6	2,6	1,5	2,5	2,5
2,6	1,6	1,8	10	10	1,5	10	1,8	10	10
2,6	1,6	10	10	10	2,6	10	10	2,5	1,5
1,5	1,5	1,5	1,5	1,6	1,6	10	1,5	2,6	1,5
1,5	1,6	10	10	1,5	1,5	10	1,8	1,5	1,5
1,5	1,6	1,8	10	1,5	1,5	10	1,8	1,6	2,5
1,6	1,6	1,8	10	1,6	1,6	10	1,8	2,6	1,5
1,6	1,6	1,8	1,5	1,6	1,6	10	1,8	2,6	1,5
1,5	1,6	1,8	10	1,6	1,6	10	1,8	2,6	1,5
1,6	1,6	1,8	10	1,5	1,5	10	1,8	2,6	1,5
1,5	1,5	1,5	1,5	1,5	2,6	2,6	1,5	1,6	1,5

1,7	1,7	10	1,7	10	1,6	10	1,8	1,6	10
1,7	2,5	10	1,5	2,5	1,5	10	10	1,5	1,5
1,6	1,7	1,8	10	1,8	1,6	10	1,8	1,6	2,5
1,7	1,6	1,5	10	10	1,5	10	1,8	1,5	1,5
1,5	1,6	10	10	1,5	1,5	10	10	2,5	1,5
1,5	1,7	1,5	10	1,5	1,5	10	10	10	2,5
1,7	1,6	1,5	10	1,5	2,5	2,6	1,5	1,6	2,5
1,6	1,7	1,9	10	10	1,6	10	1,8	1,5	1,5
1,6	1,6	1,8	10	10	1,6	10	1,5	1,5	1,5
1,7	1,7	1,7	1,8	1,5	1,5	10	1,5	1,5	2,5
1,6	1,6	1,8	10	10	1,5	10	1,8	1,6	1,8
1,6	10	1,8	10	1,5	1,5	10	1,8	1,5	10
1,7	10	1,5	3,7	1,7	1,7	10	1,8	1,7	1,7
1,6	1,6	10	1,6	1,5	2,5	10	10	1,5	10
1,6	1,6	1,7	10	10	1,5	10	1,8	1,6	1,8
2,6	1,5	1,5	10	1,5	2,6	2,6	1,5	2,6	1,5
1,8	10	10	10	1,8	2,8	10	1,8	1,8	1,8
1,7	1,5	10	10	10	1,6	10	10	1,5	10
1,7	1,6	1,5	10	1,5	1,5	10	1,5	2,5	1,5
1,6	1,6	1,8	1,8	10	1,8	10	1,8	10	10
1,6	1,7	1,6	10	1,5	2,6	2,6	1,5	2,6	1,5
1,7	1,5	10	1,5	1,5	1,5	10	1,8	1,5	1,5
1,5	1,6	1,5	1,5	1,5	1,5	10	1,5	1,7	1,5
1,7	1,6	1,5	1,5	1,5	1,5	10	1,5	1,5	1,5
1,7	1,6	1,5	1,5	1,5	1,5	10	1,5	1,7	1,5
3,7	1,7	1,8	10	3,5	3,5	10	1,5	3,6	3,5
1,7	1,7	1,5	10	1,6	1,6	10	10	1,6	10
1,7	1,7	10	10	10	1,5	10	10	1,5	1,5
1,6	10	1,8	10	1,5	1,5	10	1,8	1,5	10
1,5	2,5	1,5	1,8	1,5	2,6	2,6	1,5	2,6	2,5
2,7	1,7	1,8	1,5	1,8	2,5	10	1,8	1,5	1,8
1,7	1,7	1,5	1,5	1,5	1,5	10	1,5	2,6	1,5
2,6	1,5	1,5	1,5	1,5	2,6	2,6	1,5	2,6	2,5
1,7	1,5	1,8	1,5	1,6	1,6	1,6	1,8	2,5	1,5
1,7	1,5	1,8	1,7	1,6	1,7	10	10	1,6	1,6
1,5	1,5	1,8	1,5	10	10	10	1,8	1,5	1,5
2,5	1,6	1,6	1,7	1,7	1,5	1,5	10	1,5	1,7
2,7	1,5	2,6	10	2,5	10	10	10	1,5	1,5
2,8	1,5	2,8	1,8	2,6	1,6	10	1,8	1,5	1,5
2,6	1,7	1,8	1,8	1,5	1,5	10	10	1,5	1,5
1,7	1,5	1,5	10	1,5	1,5	10	10	10	1,5
1,6	1,6	1,5	10	1,5	1,5	10	1,5	1,5	1,5
2,7	10	1,9	10	1,5	1,6	10	1,8	1,6	1,6
2,6	1,5	1,5	1,5	2,6	1,6	10	1,5	2,6	1,5
1,7	1,6	1,5	1,5	1,5	2,6	2,6	1,5	2,6	1,5
1,6	1,7	1,8	1,8	1,8	1,6	10	1,8	2,6	1,5
1,6	1,5	1,5	1,5	2,5	2,5	2,5	1,5	2,6	2,5

1,6	1,7	1,8	10	10	1,5	10	1,8	1,5	10
1,7	1,7	1,6	1,6	1,6	1,6	10	1,5	2,6	1,5
1,8	1,5	1,8	10	1,5	2,5	10	1,8	2,8	1,6
1,8	1,5	1,8	10	1,5	2,5	10	1,8	2,8	1,6
1,6	1,6	1,8	10	1,5	1,6	10	1,8	1,6	1,5
1,8	1,5	1,5	1,8	1,5	1,5	10	1,8	2,5	1,5
1,5	1,5	1,8	10	2,5	2,6	2,6	1,8	2,6	2,5
1,7	1,5	1,8	1,5	1,5	1,5	10	1,5	1,6	1,5
1,7	1,7	1,5	10	1,5	1,6	10	1,8	1,5	1,5
1,6	1,6	1,5	1,5	1,5	1,5	10	10	1,5	1,5
1,5	1,5	1,5	10	1,5	1,5	10	1,5	1,6	1,5
1,5	1,5	1,5	10	1,5	1,5	10	1,5	1,6	1,5
1,6	10	1,8	1,8	10	1,5	10	1,8	1,5	10
1,6	1,6	1,8	1,8	1,6	1,5	10	1,5	1,6	10
1,7	2,6	1,5	1,8	1,5	2,6	10	1,5	2,6	1,5
2,6	1,6	1,5	2,6	1,5	1,5	10	1,5	1,6	1,5
1,6	1,6	1,8	10	10	1,5	10	1,5	2,6	10
1,6	2,6	1,6	10	10	1,5	10	1,5	1,6	10
1,7	1,6	1,8	10	2,6	10	10	1,8	2,6	2,5
1,5	1,5	1,5	10	1,5	1,5	1,5	1,5	1,6	1,5
3,7	10	1,8	1,6	1,6	1,6	1,5	10	2,5	1,5
1,8	10	10	10	10	2,6	10	10	2,6	10
1,8	10	10	10	10	2,6	10	10	2,6	10
1,6	1,6	1,8	1,5	1,7	1,6	10	1,8	2,6	2,5
1,6	1,6	1,8	1,5	1,7	1,6	10	1,8	2,6	2,5
1,6	1,6	10	10	1,5	10	10	1,5	1,5	1,5
1,6	1,6	1,5	1,5	1,5	1,6	10	1,5	1,6	1,5
2,7	1,7	1,8	10	1,5	1,5	10	1,8	1,5	1,5
1,6	10	1,8	1,6	10	1,6	10	1,8	1,5	10
1,6	1,6	1,8	1,5	1,5	2,5	2,6	1,5	2,6	2,5
2,8	1,6	1,8	10	1,5	1,5	10	1,5	2,5	1,5
2,6	1,6	10	10	10	1,5	10	1,5	10	1,5
2,6	1,5	10	1,8	1,5	1,5	10	1,6	1,6	1,5
1,7	1,6	1,8	1,5	2,5	2,5	2,5	1,5	2,6	2,6
1,6	1,6	1,8	10	1,5	1,6	10	1,8	1,5	1,5
1,6	1,6	1,8	1,8	10	1,5	10	1,8	1,6	1,5
1,6	1,6	10	10	10	1,5	10	1,8	1,6	10
1,5	1,6	10	1,8	10	1,5	10	1,8	1,6	1,5
3,5	1,5	1,5	1,5	1,5	1,5	10	1,5	2,5	1,5
3,5	1,5	1,5	1,5	1,5	1,5	10	1,5	2,5	1,5
1,5	1,6	10	10	10	1,5	10	1,8	1,5	1,5
1,7	1,7	1,8	10	1,5	2,7	10	1,5	2,5	1,4
1,7	1,7	10	1,7	1,6	1,5	10	1,8	1,5	1,5
1,6	1,6	1,5	1,5	1,5	1,5	10	1,8	1,5	1,5
1,6	1,6	10	10	1,5	1,5	10	1,5	1,5	1,5
2,6	10	1,8	1,6	1,5	1,5	10	10	1,5	1,5
2,7	1,7	10	1,8	10	1,6	10	1,8	1,7	1,5

2,6	1,7	1,8	1,8	1,6	1,7	10	1,8	1,6	1,5
1,6	10	10	1,5	10	1,6	10	1,8	1,5	1,5
1,6	1,7	10	1,5	1,5	1,5	10	1,8	1,5	1,5
2,6	3,5	1,8	1,5	2,5	2,6	2,6	1,8	2,5	2,5
2,6	3,5	1,8	1,5	2,5	2,6	2,6	1,8	2,5	2,5
1,6	10	10	1,8	10	1,6	10	1,5	1,6	1,5
2,7	1,7	10	10	10	1,5	10	1,8	10	1,5
1,7	1,5	1,8	10	10	1,7	10	1,8	2,6	10
1,6	1,5	1,8	10	1,7	1,6	10	1,8	1,6	1,5
2,7	1,7	1,8	10	1,6	1,6	10	1,8	1,6	1,5
1,7	1,7	10	10	1,5	1,5	10	1,5	1,5	1,5
1,5	1,7	1,8	10	1,5	1,5	10	1,8	2,6	2,5
1,6	10	1,8	1,8	1,5	1,5	10	1,8	1,6	1,5
1,6	1,6	10	10	10	1,5	10	1,7	1,6	1,5
1,5	1,7	10	10	1,5	1,5	10	1,5	1,7	1,5
1,6	1,6	1,8	1,5	1,5	1,5	10	1,8	1,6	1,5
1,5	1,5	10	10	1,5	1,5	10	1,5	1,6	1,5
1,6	10	10	10	10	1,5	10	1,8	1,5	1,5
1,6	1,7	10	10	10	1,5	10	1,8	1,6	1,5
2,7	1,6	1,8	1,6	1,5	1,8	1,6	10	1,6	1,5
1,7	1,6	1,8	10	1,5	1,5	10	1,5	1,6	1,5
1,6	1,5	10	10	1,8	1,5	10	1,5	1,5	1,5
1,6	1,6	10	10	1,7	1,5	10	1,5	1,5	1,5
3,8	10	10	1,8	2,6	3,6	10	1,8	3,5	3,5
1,8	1,8	1,8	1,8	1,5	2,6	10	1,8	2,8	1,8
1,6	1,6	1,5	1,5	1,5	1,5	1,5	1,5	1,6	1,5
1,5	1,6	1,8	1,5	1,5	2,6	2,6	1,8	1,6	1,5
1,6	10	1,8	10	10	1,5	10	1,8	1,6	1,5
10	1,6	1,5	1,8	3,7	3,7	10	1,8	2,6	2,5
1,7	1,7	1,8	10	10	1,6	10	10	1,6	1,5
1,6	1,6	1,8	10	1,6	1,6	10	1,8	2,7	2,5
1,6	1,5	1,7	1,8	1,6	1,5	10	1,5	1,5	1,5
1,5	1,5	1,5	1,5	1,5	1,5	1,5	1,5	1,5	1,5
1,6	1,7	1,6	1,5	1,6	2,6	10	1,8	2,6	1,5
1,6	1,6	1,8	10	10	1,5	10	1,5	1,6	1,5
1,6	1,6	10	1,8	10	1,5	10	1,8	1,6	1,5
2,6	1,5	1,6	10	3,6	1,5	10	1,5	1,5	10
2,6	1,7	1,8	10	2,6	1,6	10	10	2,6	1,5
1,7	1,6	10	10	10	1,5	10	10	1,6	1,5
1,7	1,6	1,5	1,8	1,5	1,7	10	1,5	1,5	1,5
1,6	1,7	10	1,8	1,5	1,6	10	10	2,5	10
1,6	1,5	1,5	10	1,5	1,5	10	10	1,6	1,5
1,7	1,7	1,8	10	1,5	1,6	10	1,8	10	1,5
1,7	1,5	10	10	2,5	2,5	10	10	2,8	1,5
2,7	1,7	2,8	10	1,5	1,6	10	1,8	10	1,5
1,6	1,7	1,8	1,8	1,6	1,5	10	1,5	2,6	1,6
1,6	1,5	1,5	1,6	2,5	2,6	2,6	1,5	2,6	2,5

1,6	1,5	1,5	1,6	2,5	2,6	2,6	1,5	2,6	2,5
1,6	10	1,8	10	10	1,5	10	1,8	1,6	1,5
1,5	1,5	1,7	10	1,6	1,6	10	1,8	2,6	1,5
1,6	1,6	1,8	1,8	10	1,5	10	1,8	1,6	1,5
1,6	1,6	1,8	10	10	1,5	10	1,8	1,6	1,5
1,6	1,5	10	10	1,5	1,5	10	1,8	1,6	1,5
1,6	1,6	10	10	10	1,5	10	10	1,5	1,5
1,6	1,6	10	1,5	10	1,5	10	1,8	1,6	1,5
1,6	1,6	1,9	10	10	1,5	10	10	1,5	1,5
1,5	1,5	1,7	1,8	10	1,5	10	10	1,6	10
2,6	1,6	1,5	10	1,5	1,5	1,5	10	1,5	1,5
2,7	3,7	1,8	10	1,5	1,6	10	1,5	1,5	1,5
1,5	1,7	10	10	3,5	3,5	10	10	1,5	1,5
1,5	1,6	10	10	1,5	2,5	10	10	1,5	1,5
1,8	1,6	10	10	1,5	2,6	10	10	2,5	1,5
1,7	1,5	1,8	1,6	1,5	1,5	10	1,8	1,5	1,5
1,6	1,6	1,8	1,8	1,5	2,5	2,5	1,8	2,5	1,5
1,6	1,5	1,8	1,8	1,5	2,5	2,5	1,8	2,5	1,5
1,6	1,6	1,9	10	10	1,6	10	10	10	10
2,7	1,7	1,8	10	1,5	1,6	10	1,8	2,7	1,5
3,8	10	10	10	10	2,5	10	10	2,6	2,5
1,6	1,5	1,8	10	1,5	1,6	10	10	2,6	1,5
2,7	1,7	1,7	1,7	1,5	1,7	10	1,8	2,7	1,5
1,6	10	1,8	1,6	1,5	2,5	10	10	10	1,5
1,6	10	1,8	1,6	1,5	2,5	10	10	10	1,5
1,7	1,7	10	1,5	1,5	1,5	1,5	1,8	1,7	1,5
1,8	1,6	10	10	1,5	2,6	10	10	2,5	1,5
1,6	1,6	10	10	10	1,6	10	10	1,6	10
1,5	1,7	1,8	1,8	2,5	1,8	10	10	1,5	2,5
3,7	1,6	1,8	1,8	1,5	2,6	10	10	2,6	1,5
1,6	1,6	1,8	1,5	1,6	1,5	10	1,8	1,5	1,5
1,7	1,6	10	10	10	2,6	10	10	2,5	1,5
1,6	1,7	1,5	10	1,5	10	10	1,5	1,5	1,5
1,7	1,7	1,5	1,5	1,5	1,5	10	1,5	1,5	1,5
1,7	1,6	1,8	1,8	1,5	1,5	1,5	1,8	2,5	2,5
2,8	1,5	10	10	10	1,5	10	10	10	1,5
1,8	1,6	10	10	1,5	1,6	10	1,8	2,5	2,5
1,6	1,6	10	1,5	10	1,5	10	1,8	1,5	1,5
1,6	1,5	1,8	1,8	1,6	1,8	10	1,6	1,6	1,5
1,5	1,5	1,8	1,8	1,6	1,6	10	10	2,6	1,5
1,6	1,6	1,8	10	1,5	1,6	10	1,5	2,6	1,5
1,8	10	10	1,7	1,6	1,6	10	10	1,6	2,5
1,6	1,6	10	10	10	1,5	10	10	1,6	1,5
1,6	1,6	10	10	10	1,5	10	10	1,6	1,5
1,6	1,5	1,5	1,5	1,5	1,5	10	1,6	2,6	1,5
1,6	1,6	1,5	1,5	1,5	1,5	10	1,5	2,6	1,5
1,6	1,5	1,5	10	1,5	1,5	10	1,4	1,6	1,5

2,7	1,5	1,5	1,5	1,5	1,5	1,5	1,5	2,6	1,5
2,8	1,5	1,5	10	1,5	1,5	10	1,4	1,5	1,5
1,5	1,5	1,5	1,5	1,5	1,5	1,5	10	10	1,5
1,7	1,6	1,8	1,6	1,5	1,5	10	10	1,6	1,5
1,5	1,5	1,5	1,5	1,5	1,5	1,5	1,5	1,5	1,5
1,6	10	1,6	10	2,6	1,6	10	1,7	1,5	1,5
1,5	1,5	1,5	1,5	1,5	1,5	10	1,5	1,5	10
1,6	1,5	1,5	1,5	1,5	1,5	10	1,5	1,5	1,6
1,6	1,6	1,7	10	1,5	1,6	10	1,7	1,6	1,6
1,6	1,6	1,7	10	1,5	1,6	10	1,7	1,6	1,6
1,7	10	10	10	10	1,5	10	10	1,5	1,5
1,5	1,5	1,5	1,5	1,5	1,5	10	1,5	1,5	1,5
1,6	10	1,6	10	2,6	1,6	10	1,8	1,7	1,5
1,5	1,5	1,6	1,6	1,5	1,5	10	1,5	1,6	1,6
1,7	1,6	1,8	1,5	1,5	1,5	10	1,8	1,6	1,6
2,7	1,7	1,5	10	1,5	1,5	10	1,5	2,6	1,5
1,6	1,6	1,8	1,5	1,5	1,5	10	1,8	1,6	1,5
10	10	1,8	10	1,5	1,7	10	10	10	1,5
1,6	1,5	1,5	1,6	1,6	1,6	10	1,6	1,6	1,6
1,7	1,7	1,8	1,8	1,6	1,6	10	1,8	1,6	1,5
1,6	2,5	1,8	10	10	1,5	10	1,5	1,6	1,5
1,6	1,7	1,8	10	10	1,5	10	1,8	1,6	1,5
1,6	1,7	1,5	10	1,8	1,6	10	1,5	1,6	1,5
1,7	1,7	1,8	10	1,5	1,6	10	1,8	1,6	1,5
1,6	1,6	1,8	1,8	1,5	1,5	10	1,8	1,5	1,5
1,7	1,6	1,8	1,5	1,5	1,5	10	1,5	1,5	1,5
1,6	1,7	1,8	10	10	1,6	10	1,8	1,5	1,5
1,7	1,7	1,5	10	1,5	1,5	10	1,8	1,5	1,5
1,6	1,7	1,8	10	1,5	1,6	10	1,5	1,6	1,5
1,6	1,6	1,8	10	10	1,5	10	1,8	1,5	1,5
1,6	1,5	1,8	10	10	1,5	10	1,5	1,5	1,5
1,5	1,5	1,5	1,6	1,5	1,5	10	1,5	1,5	1,5
1,6	1,5	1,8	10	10	1,5	10	1,5	1,5	1,5
1,7	1,7	1,8	10	1,5	1,5	10	1,8	1,5	1,5
1,6	1,7	1,5	1,5	10	1,6	10	1,5	1,6	10
1,7	1,5	1,5	10	10	10	1,6	1,5	1,5	1,5
1,7	1,6	10	10	1,5	1,5	10	1,5	1,5	1,5
1,7	1,7	1,8	10	10	1,6	10	1,5	1,6	1,5
1,7	1,7	1,8	1,8	10	1,5	10	1,8	1,6	1,5
1,7	1,7	1,5	10	1,8	1,6	10	1,5	1,6	1,5
2,7	1,5	1,8	1,8	1,5	1,5	10	10	1,6	1,5
1,6	1,5	1,8	10	1,5	1,5	10	10	1,5	1,5
1,6	1,5	1,8	10	10	1,5	10	1,5	1,5	1,5
1,7	1,5	1,8	1,5	1,5	1,5	10	10	1,5	1,5
1,6	1,6	10	10	1,5	1,5	10	1,8	1,5	1,5
1,6	1,6	1,8	10	1,5	1,5	10	10	1,6	1,5
1,6	1,6	1,8	1,5	1,5	1,5	10	1,8	1,6	1,5

1,7	1,6	1,8	1,8	1,5	1,5	10	1,8	1,6	1,5
1,6	1,6	1,8	1,8	1,5	1,5	10	1,5	1,5	1,5
1,5	1,5	1,5	1,5	1,5	1,5	1,5	1,5	1,5	1,5
1,6	1,6	10	10	1,5	1,5	10	10	1,6	2,6
1,5	1,7	1,5	1,5	1,5	10	10	1,5	1,6	1,5
1,7	1,6	1,8	1,5	1,5	1,5	10	1,6	1,5	1,5
1,6	1,7	1,8	1,8	1,5	1,5	10	1,8	2,6	1,5
1,6	1,7	1,8	1,6	1,5	1,5	10	1,8	2,6	1,5
1,7	1,5	1,5	10	10	1,5	10	1,5	1,5	1,5
3,7	2,7	10	10	2,5	1,6	10	10	2,5	2,6
1,7	1,7	1,8	10	1,8	1,6	10	1,5	1,7	1,5
1,7	1,5	1,8	1,8	1,5	1,5	10	1,6	1,5	1,5
1,6	1,6	1,8	10	1,5	1,5	10	1,8	1,6	1,5
1,5	1,6	1,8	10	1,5	1,6	10	1,8	2,6	1,5
1,6	1,6	1,8	10	1,5	1,5	10	1,8	1,6	1,5
1,5	1,5	1,8	10	10	1,5	10	1,8	2,5	1,5
1,5	1,5	1,8	10	10	1,5	10	1,5	1,6	1,5
1,6	1,6	1,8	10	1,5	1,5	10	1,8	3,6	1,5
1,5	1,7	1,5	1,5	1,5	10	10	1,5	2,6	1,5
1,7	1,6	1,8	1,8	1,5	1,5	10	1,5	1,5	1,5
1,5	1,5	10	10	1,5	1,5	10	1,5	1,5	1,5
1,6	1,7	1,8	10	1,5	1,6	10	1,5	1,7	1,5
1,6	1,7	1,8	1,5	1,5	1,5	10	1,8	2,6	1,5
1,5	1,5	1,5	1,5	1,5	1,5	1,5	1,5	1,5	1,5
1,6	1,6	1,8	10	1,5	1,5	10	1,8	1,6	1,5
1,7	1,7	1,6	1,6	1,6	1,6	10	1,5	1,6	1,8
1,6	1,6	1,8	10	1,5	1,5	10	1,8	1,6	1,5
1,6	1,6	1,8	10	1,5	1,5	10	1,8	2,6	1,2
1,6	1,6	1,8	10	1,5	1,5	10	1,8	1,6	1,5
1,6	1,6	1,8	1,5	1,5	1,5	10	1,8	1,6	1,5
10	10	10	10	10	1,5	10	10	1,8	10
1,7	1,7	1,8	10	1,5	1,5	10	10	2,6	10
10	1,8	10	1,5	10	1,5	10	10	10	1,5
2,8	2,6	1,8	10	1,8	2,6	10	1,8	1,5	1,5
2,6	2,7	1,8	10	10	2,6	10	1,8	2,6	1,5
1,6	1,6	1,6	10	1,5	1,5	10	1,8	1,5	1,5
10	1,6	10	10	10	2,7	10	10	10	10
1,7	1,5	1,8	10	1,5	1,6	10	1,8	1,5	1,5
1,6	1,6	1,8	10	1,5	1,5	10	1,6	1,6	1,5
1,5	1,5	1,5	10	1,5	1,6	10	1,5	1,5	1,5
3,7	19	10	10	1,6	2,6	10	10	2,6	1,6
1,6	1,6	1,8	1,5	1,5	1,5	10	1,8	1,6	1,5
3,7	2,5	10	10	1,5	1,6	10	10	2,6	2,6
1,5	1,5	1,5	1,5	1,5	1,5	10	1,5	1,5	1,5
10	10	10	10	1,5	1,5	10	10	1,6	10
1,7	10	1,8	10	1,5	1,5	10	1,6	1,5	1,5
2,7	1,5	1,5	10	1,55	1,5	10	1,5	1,5	10

1,7	1,7	1,8	10	1,5	1,6	10	1,5	1,6	1,5
1,6	10	1,5	1,6	1,7	1,7	10	1,5	10	1,5
1,5	1,5	1,5	10	10	1,5	10	1,5	1,5	1,5
1,6	1,7	1,5	1,6	10	1,6	10	1,5	1,6	1,5
1,6	1,7	1,7	1,6	10	1,6	10	1,5	1,5	1,5
1,6	1,7	1,6	1,5	10	1,6	10	1,5	1,6	1,5
1,6	1,6	1,8	1,6	1,5	1,5	10	1,8	1,6	1,5
2,5	1,5	1,8	10	1,5	1,5	10	1,8	1,6	1,5
1,6	1,6	1,6	10	10	1,5	10	1,5	1,5	1,5
1,7	2,7	1,6	1,8	1,8	2,5	1,5	1,8	2,6	1,5
2,7	1,5	1,5	10	1,7	1,5	10	1,5	1,6	1,5
1,7	1,5	1,7	1,6	10	1,5	10	1,5	1,5	1,5
1,6	1,6	1,8	1,5	10	1,5	10	10	1,6	1,5
1,7	10	1,8	1,8	1,5	1,6	10	1,5	1,7	1,5
1,6	1,6	1,8	1,5	10	1,5	10	10	1,6	1,5
1,6	1,5	1,8	1,5	1,6	1,6	10	1,5	1,5	1,5
1,6	1,6	1,6	1,6	1,5	1,6	10	1,6	1,6	1,6
1,5	1,5	1,8	1,5	10	1,5	10	1,5	1,5	1,5
1,7	1,7	1,5	1,5	1,5	1,6	10	1,5	1,6	1,6
1,6	1,6	1,6	1,6	1,5	1,6	10	1,6	1,6	1,6
1,7	1,7	1,8	1,6	10	1,6	10	1,8	1,6	1,5
1,7	1,5	1,5	10	1,6	1,5	10	1,5	1,5	1,5
2,5	1,5	2,5	10	1,5	1,6	10	2,5	2,6	1,5
1,6	1,7	1,8	1,5	1,6	1,6	10	1,8	1,6	1,5
1,5	1,5	1,5	1,5	1,5	1,5	1,5	10	2,5	1,5
2,7	1,5	1,5	10	1,5	1,6	10	1,5	2,6	1,5
1,5	1,6	1,5	1,6	1,5	1,5	1,5	1,5	1,6	1,5
2,7	1,5	2,5	1,5	1,6	1,6	10	1,6	2,6	1,5
2,6	1,5	1,6	10	1,5	1,6	10	1,5	1,6	1,5
2,6	1,5	1,6	10	1,5	1,6	10	1,5	1,6	1,5
1,6	1,5	1,5	1,6	1,5	1,5	1,6	1,6	1,5	1,5
1,6	1,7	1,8	1,6	1,6	1,6	10	1,8	2,6	1,5
1,6	1,6	1,8	10	1,5	1,5	10	1,5	1,6	1,5
1,7	1,7	1,8	1,7	1,8	1,7	10	1,5	1,7	1,5
1,5	1,5	1,5	1,5	1,5	1,5	1,5	1,5	1,5	10
1,7	1,7	1,8	10	1,8	1,6	10	1,8	1,6	1,5
2,6	1,5	2,6	1,5	1,5	1,5	10	1,6	2,6	1,5
2,6	1,5	2,6	1,5	1,5	1,5	10	1,6	2,6	1,5
1,5	1,5	1,5	10	1,5	1,5	10	1,5	1,5	1,5
1,5	1,5	1,5	10	10	1,5	10	1,5	1,5	1,5
1,5	10	1,5	1,5	10	1,5	10	1,5	1,5	1,5
1,5	1,5	1,6	1,6	1,5	1,5	1,5	1,6	1,5	1,6
1,5	1,5	1,5	10	1,5	1,5	10	1,5	1,5	1,5
1,6	1,5	1,5	10	1,5	1,5	10	1,5	2,6	1,5
3,5	1,7	1,5	10	1,5	1,5	10	1,5	1,5	10
1,6	1,6	1,8	1,6	1,5	1,5	10	1,8	2,6	1,5
1,6	1,5	1,8	10	1,5	1,5	10	10	2,6	1,5

1,7	1,5	1,5	1,5	1,5	1,5	10	1,5	1,5	1,8
1,7	1,5	1,8	1,5	1,5	1,5	10	1,5	1,5	1,8
1,5	1,5	1,5	1,5	1,5	1,5	10	1,5	1,5	10
1,5	1,5	1,5	10	1,5	1,5	10	1,5	1,8	10
1,6	1,5	1,5	10	1,5	1,5	1,5	1,5	1,5	10
1,5	1,7	1,5	10	10	1,6	10	1,5	1,6	10
1,5	10	10	1,8	1,7	10	10	10	10	1,5
1,6	1,6	1,6	1,6	1,6	1,6	1,6	1,6	1,7	1,5
1,6	1,6	1,6	1,6	1,6	1,6	10	1,6	1,7	1,5
1,7	1,7	10	10	1,6	1,5	10	10	1,5	1,5
2,6	1,7	1,8	1,5	1,5	1,6	1,5	1,5	1,5	1,5
1,6	1,5	1,5	1,5	1,5	1,6	1,8	1,8	1,5	1,5
1,6	1,7	1,8	1,5	1,5	1,5	10	1,8	1,6	1,5
1,6	1,6	1,6	1,5	1,5	1,5	10	1,5	1,5	10
1,5	1,7	1,5	10	10	1,5	10	1,5	1,6	1,8
1,7	1,5	1,8	1,8	1,5	1,6	10	1,5	1,5	1,5
3,5	10	1,5	10	1,5	1,5	10	1,5	10	1,5
1,7	1,6	1,8	10	1,5	1,6	1,5	1,5	1,5	1,6
1,5	1,5	10	10	10	2,6	10	1,5	2,7	2,5
2,7	2,7	1,7	10	1,5	1,7	1,6	1,7	1,5	2,6
1,5	1,5	1,8	1,5	1,5	1,5	10	1,8	1,5	1,5
1,6	1,6	1,6	10	1,5	1,5	10	1,5	1,5	1,5
1,7	1,6	1,6	10	1,5	1,5	10	1,6	1,5	1,5
1,8	2,6	10	10	1,5	1,5	10	1,9	2,6	1,5
1,7	1,5	1,8	10	10	1,5	10	1,8	2,5	1,5
2,8	2,7	1,8	10	2,6	1,7	10	1,7	3,7	2,6
1,7	1,7	1,8	1,5	1,5	1,5	10	1,8	1,6	1,5
1,6	1,5	2,7	1,5	1,5	1,5	10	10	1,5	1,5
1,5	1,5	1,5	1,5	1,5	1,5	10	1,5	1,5	1,5
1,6	1,5	1,8	1,5	1,5	1,6	1,5	1,5	1,6	1,5
1,5	1,5	1,5	10	1,5	1,5	10	1,5	1,5	1,5
1,6	1,5	1,8	1,8	1,5	1,5	10	1,8	1,6	1,5
1,6	1,6	1,5	10	1,5	1,5	10	1,5	10	1,5
1,5	1,5	1,5	10	10	10	10	1,5	1,5	10
1,6	1,6	1,5	10	1,5	1,5	10	1,5	10	1,5
1,5	1,6	1,5	1,8	1,5	1,5	10	1,8	1,6	1,5
1,7	1,5	1,8	1,5	1,5	1,5	1,8	1,8	1,5	1,5
1,6	1,6	1,8	10	1,5	1,5	10	1,8	1,6	1,5
1,7	10	1,8	10	3,5	3,5	10	1,5	1,5	1,5
1,7	1,5	1,5	1,5	1,5	1,5	10	1,5	1,5	10
1,6	1,7	1,8	1,6	1,5	1,5	10	1,8	2,6	1,5
1,7	1,7	1,5	10	10	1,6	10	1,6	1,6	1,6
1,5	10	10	10	10	1,5	10	10	1,5	1,5
1,6	1,5	1,5	10	1,5	1,5	1,5	1,5	1,5	10
1,5	1,7	1,5	10	1,5	1,6	10	1,5	1,6	1,5
2,5	2,6	2,5	1,5	2,5	1,6	1,5	1,5	2,5	3,5
1,7	1,7	1,7	10	1,5	1,6	10	1,6	1,6	1,5

1,5	1,5	1,5	10	1,5	1,5	10	1,5	1,5	1,5
2,5	2,5	2,5	2,5	10	2,5	10	2,5	2,5	1,5
2,6	2,7	2,8	10	10	2,7	10	2,5	2,6	1,5
1,7	1,6	1,5	1,5	10	1,6	10	1,5	1,6	1,5
1,7	1,5	1,8	10	1,5	1,5	10	1,5	1,5	1,5
2,6	2,7	1,6	1,5	10	1,6	10	1,5	1,6	1,5
1,6	1,6	2,6	1,6	1,5	2,6	10	10	2,6	1,5
1,7	1,5	1,9	1,5	1,5	1,5	10	1,8	1,5	1,5
1,5	1,7	1,5	10	10	1,6	10	1,5	1,6	1,5
1,6	1,6	1,5	10	1,6	1,6	10	1,8	1,5	1,5
2,6	1,7	1,8	1,8	10	2,6	10	1,6	2,6	1,5
2,6	3,6	1,8	1,8	1,5	1,5	10	1,8	1,5	1,5
1,5	1,7	1,5	10	1,5	1,6	10	1,5	1,6	1,8
1,5	1,7	1,5	10	1,5	1,6	10	1,5	1,6	1,8
1,7	1,6	1,8	1,5	1,5	1,5	10	1,8	1,6	1,5
2,7	1,7	1,7	1,5	1,5	1,6	10	1,5	1,7	10
1,7	1,7	1,8	10	1,5	1,5	10	1,8	1,6	1,5
1,7	1,7	1,8	10	1,5	1,5	10	1,8	1,6	1,5
1,7	1,5	1,8	1,5	1,5	1,5	10	1,8	1,6	1,5
1,6	1,7	1,8	1,5	1,5	1,5	10	1,8	1,6	1,5
1,7	1,6	1,8	1,5	1,8	1,5	10	1,8	2,6	1,6
2,7	1,7	1,7	1,5	1,5	1,6	10	1,5	1,7	10
10	10	10	10	10	10	10	1,5	1,6	1,5
1,8	1,5	1,8	10	10	1,6	10	10	1,5	1,5
1,6	1,7	10	10	1,5	1,6	10	10	10	1,5
1,5	1,7	10	1,8	1,5	3,6	10	1,8	2,5	1,5
1,6	1,8	10	10	1,5	1,6	10	10	10	1,5
1,5	1,5	1,5	1,5	1,5	1,6	10	1,5	1,5	1,5
2,5	1,7	1,5	10	1,5	1,5	10	1,8	2,5	1,5
1,5	1,5	1,5	10	1,5	1,5	10	10	1,5	1,5
1,6	1,6	1,8	1,6	1,5	1,5	10	1,8	2,6	1,5
1,6	1,5	1,5	1,5	1,5	1,5	10	1,5	1,5	1,5
10	1,6	1,8	1,5	1,5	2,6	10	10	2,6	1,5
1,5	1,5	1,5	1,5	1,5	1,5	10	1,5	1,5	1,5
1,5	1,5	1,6	10	1,5	1,5	10	1,6	1,5	1,5
1,7	1,7	1,5	1,7	1,5	1,6	10	1,5	1,6	1,5
1,6	1,6	1,5	1,8	1,5	1,5	1,5	1,5	1,5	1,5
1,6	1,6	1,6	1,6	1,6	1,6	10	1,6	1,5	1,6
1,6	1,7	1,6	10	1,5	1,5	10	1,5	2,6	1,5
1,6	1,7	1,8	1,6	1,5	1,5	10	1,8	3,6	1,5
1,5	1,5	1,5	10	10	1,5	10	1,5	1,5	1,5
1,8	1,7	10	10	10	1,6	10	10	1,5	1,5
1,8	1,7	10	10	10	1,6	10	10	1,5	1,5
1,5	1,5	1,5	10	1,5	1,5	10	10	1,5	1,5
1,5	1,7	1,8	1,8	10	1,6	10	10	1,5	1,5
1,8	1,7	1,8	10	10	1,6	10	1,8	1,6	1,5

sugar bear	kidney bea	boiled jug	boiled drie	butter bea	boiled fres	roasted gr	processed	marula nu	roasted bu
1,6	10	10	10	10	10	10	1,5	10	10
1,6	10	10	10	10	10	10	1,5	10	10
1,7	10	1,7	10	1,7	1,7	1,7	1,7	1,7	10
1,8	10	10	10	10	10	1,5	10	10	10
1,5	10	10	10	10	2,5	1,5	1,5	1,5	10
1,5	10	10	10	10	10	10	10	10	10
1,5	10	10	10	10	1,5	10	10	10	10
10	10	10	10	1,5	1,9	1,9	1,8	10	10
2,6	10	2,9	2,9	10	2,6	2,9	1,5	2,9	10
10	10	10	10	10	1,8	10	1,8	10	10
1,5	10	10	10	10	10	2,6	1,5	10	10
2,6	10	10	2,6	10	2,6	1,5	1,8	1,5	10
2,6	10	2,5	10	10	2,9	2,5	1,5	1,5	10
1,8	10	10	10	10	10	10	10	1,9	10
1,5	10	10	10	10	1,9	10	1,9	1,9	10
10	10	10	10	10	10	1,8	1,8	10	10
10	10	10	10	10	10	1,8	1,8	10	10
2,6	10	1,5	10	10	2,5	2,5	1,5	1,5	10
2,6	10	2,5	10	10	2,6	2,6	1,5	1,5	10
2,5	10	10	2,5	10	2,5	2,5	1,8	2,5	10
2,6	10	1,5	10	10	1,6	1,5	1,8	1,5	10
2,6	10	10	10	10	2,6	1,5	1,5	2,5	10
2,6	10	2,5	10	10	2,6	2,5	1,5	1,5	10
2,6	10	10	10	10	2,6	2,5	1,8	1,5	10
2,6	10	2,5	10	10	2,9	2,5	2,5	1,5	10
2,6	10	10	10	10	2,9	2,5	1,8	10	10
10	10	10	10	10	10	10	10	10	10
1,5	10	10	1,9	10	1,9	1,9	10	1,9	10
2,5	2,5	1,5	1,5	1,5	1,5	1,5	1,5	1,5	10
1,6	10	10	10	10	1,6	10	10	10	10
1,5	10	10	1,5	10	1,5	1,5	1,5	10	10
2,5	10	1,9	10	10	1,9	2,5	1,5	10	10
2,6	1,5	1,5	1,6	1,5	2,5	1,8	1,5	2,6	10
1,5	10	10	10	10	10	10	1,8	10	10
1,5	10	1,5	1,9	10	1,9	1,9	10	1,5	10
1,6	10	10	10	10	1,8	1,8	10	10	10
1,6	10	10	10	10	10	10	10	10	10
10	10	1,9	1,9	10	1,9	10	10	10	10
1,5	1,5	10	10	10	2,6	10	10	10	10
1,5	10	10	1,5	10	10	1,5	1,5	10	1,5
1,6	10	1,6	1,6	10	1,6	1,6	10	10	10
10	10	1,5	1,5	1,5	1,5	1,5	1,5	1,5	10
1,5	10	10	1,8	10	2,9	1,9	10	10	10
1,5	10	1,8	1,5	10	1,5	10	10	10	10
1,5	10	10	1,9	10	1,9	1,9	10	1,5	10
1,5	10	10	10	10	10	10	1,9	10	10

10	10	10	10	10	10	10	1,8	10	10
2,6	10	10	10	10	10	1,8	10	1,7	10
2,6	1,8	2,5	10	10	2,6	2,6	1,5	2,5	10
1,5	10	1,5	1,5	10	1,5	1,5	1,5	10	10
1,5	10	10	10	10	1,8	1,8	1,8	1,9	10
1,6	10	10	10	10	10	10	10	10	10
1,5	10	1,8	1,9	10	1,9	1,9	1,8	10	10
1,8	10	1,9	1,9	10	1,5	1,5	1,8	10	10
1,8	10	10	10	10	1,8	1,8	1,8	10	10
10	10	10	10	10	2,9	1,9	10	10	10
1,8	10	2,9	10	10	1,8	1,8	10	10	10
10	10	10	10	10	1,8	1,8	10	10	10
1,8	10	1,8	10	10	1,8	1,5	10	10	10
1,6	1,6	1,6	1,6	10	1,5	1,6	1,5	1,5	10
1,5	10	10	10	10	1,9	10	10	1,9	10
1,5	10	10	1,5	10	10	10	1,9	10	10
1,5	10	10	10	10	10	10	10	10	10
1,7	10	10	10	10	1,8	1,5	10	10	10
1,5	10	10	1,9	10	1,9	10	10	10	1,5
1,5	10	10	10	10	1,5	10	10	10	10
2,6	10	2,9	2,6	10	2,9	2,5	1,5	2,6	10
2,5	10	2,5	2,5	10	2,9	2,5	10	2,6	10
2,5	10	2,5	2,5	10	2,9	2,5	10	2,6	10
1,5	10	1,5	1,9	10	1,9	1,9	10	10	1,5
1,5	10	1,5	1,9	10	1,9	1,9	10	10	1,5
1,5	10	10	1,9	10	1,5	10	1,5	10	10
1,5	10	10	1,5	10	1,5	10	10	10	10
1,5	10	1,5	1,5	10	10	10	1,9	10	10
1,6	2,6	10	3,6	1,5	1,5	2,6	1,5	10	1,5
1,5	10	10	1,8	10	1,8	10	10	10	10
10	10	10	10	10	10	1,8	1,8	10	10
10	10	10	10	10	10	1,8	1,8	10	10
1,5	1,5	10	1,5	10	1,8	1,5	1,5	10	10
1,5	10	10	10	2,7	1,8	1,5	10	10	10
10	10	10	1,5	10	1,5	10	10	10	10
2,5	10	2,5	2,5	10	2,5	2,5	2,5	1,5	10
2,6	10	10	2,5	10	2,5	1,5	1,5	10	10
10	10	10	1,5	10	10	10	1,9	10	10
1,5	10	1,8	1,8	10	2,5	1,5	10	10	10
1,5	10	1,5	1,5	10	1,6	1,5	10	10	10
1,5	10	1,9	1,9	10	1,9	1,5	10	10	1,5
1,5	10	1,5	1,9	10	1,9	1,5	10	1,5	10
1,5	10	10	1,9	10	1,9	1,5	10	10	10
1,5	10	10	1,9	10	1,9	1,5	1,5	10	10
1,5	10	10	1,9	10	1,9	1,5	10	10	10
1,5	10	10	1,9	10	1,9	1,5	1,8	10	10
2,5	10	2,5	2,5	10	2,9	2,5	1,5	1,5	10

1,5	10	10	10	10	10	10	1,9	10	10
10	10	10	10	10	10	10	10	10	10
10	10	10	10	10	1,8	1,9	1,7	10	10
1,5	10	1,9	2,9	10	2,9	1,9	10	10	10
2,5	10	10	1,9	10	1,9	1,5	10	1,5	10
2,5	1,5	1,5	1,5	10	1,5	1,5	1,5	10	10
2,5	10	2,9	2,5	2,5	2,9	2,5	1,5	1,5	1,6
1,5	10	1,9	1,5	10	1,9	1,8	1,8	10	10
1,5	10	1,5	10	10	1,5	1,5	10	10	10
10	10	10	1,9	10	1,9	1,9	1,8	1,9	10
1,8	10	10	10	10	10	10	10	1,9	10
1,5	10	10	10	10	10	10	1,9	10	10
1,5	10	10	1,9	10	1,9	1,7	10	10	10
1,5	10	10	1,5	10	10	10	10	10	10
1,8	10	10	10	10	10	10	10	1,9	10
2,6	10	10	10	10	2,9	2,5	1,8	1,5	10
1,8	10	10	1,8	10	1,8	10	10	10	10
10	10	10	10	10	10	10	10	10	10
10	10	10	1,5	10	1,5	1,5	1,8	2,7	10
1,5	10	10	10	10	10	10	1,9	10	10
1,6	10	1,5	10	10	2,6	2,6	1,5	1,6	10
1,5	10	10	10	10	1,9	1,9	1,8	1,5	10
10	1,5	10	1,5	10	10	10	10	1,5	10
10	1,5	10	1,5	10	10	10	10	1,5	10
10	1,5	10	1,5	10	10	10	10	1,5	10
3,5	3,5	2,9	10	2,7	1,5	1,9	10	10	10
10	10	10	1,5	10	1,5	1,5	10	10	10
1,5	10	10	1,5	10	10	10	10	1,5	10
1,5	10	10	10	10	10	10	1,9	10	10
10	10	1,5	1,5	10	1,9	2,5	1,5	1,5	10
1,8	10	1,9	10	1,8	1,5	10	1,8	10	10
1,5	1,5	10	1,5	1,5	1,5	1,5	1,5	10	10
2,6	10	10	10	10	2,9	2,5	2,5	10	10
1,5	1,8	10	10	10	1,7	1,7	1,5	10	10
1,5	10	10	10	10	1,8	10	1,8	10	10
1,5	1,8	10	10	10	1,6	10	10	10	10
17	1,7	1,7	10	10	1,7	1,6	1,6	10	10
10	10	10	10	1,5	10	10	1,8	10	10
1,5	10	10	10	10	1,8	1,8	10	10	10
1,7	10	10	10	1,8	1,8	1,8	1,8	10	10
1,5	10	10	1,9	10	10	1,5	1,5	10	10
1,5	10	10	10	10	10	10	10	10	10
10	10	10	10	10	1,5	10	10	10	10
1,5	10	1,5	10	10	1,5	1,5	1,5	10	10
2,6	10	10	10	10	2,9	2,6	10	2,6	10
1,8	1,8	10	1,6	10	1,5	1,5	1,5	1,5	10
2,6	10	10	10	10	1,6	2,6	2,5	2,5	10

1,5	10	10	10	10	10	10	1,9	10	10
1,5	10	10	1,9	1,5	1,9	1,9	1,8	1,9	1,9
2,5	10	10	10	10	1,9	10	10	1,5	10
2,5	10	10	10	10	1,9	10	10	1,5	10
1,5	10	10	1,9	10	1,8	1,8	1,8	10	10
1,5	10	1,8	1,8	10	1,8	10	1,8	1,8	10
2,5	10	10	10	10	2,5	2,5	1,5	1,6	10
1,5	1,5	1,5	1,5	10	1,5	1,5	1,5	1,5	10
1,6	10	10	10	10	1,9	10	10	3,9	10
1,5	10	10	1,5	10	10	10	10	1,5	10
1,5	10	10	1,5	10	1,8	10	10	10	10
1,5	10	10	1,5	10	1,8	10	10	10	10
1,5	10	10	10	10	10	10	10	10	10
1,6	1,8	10	1,6	10	2,6	1,5	10	1,5	10
1,5	10	10	1,5	10	1,5	1,5	1,5	1,5	10
10	10	10	1,5	10	1,5	1,5	10	10	10
10	10	10	1,5	10	1,5	1,5	10	1,5	1,5
10	10	10	1,5	10	1,5	1,5	10	1,5	10
2,6	10	10	10	10	2,5	10	10	2,5	10
1,5	10	10	10	10	1,8	10	1,6	10	10
10	10	10	10	10	1,9	1,8	10	10	10
2,5	10	10	10	10	10	10	10	10	10
2,5	10	10	10	10	10	10	10	10	10
1,5	10	10	10	10	1,9	1,9	10	10	10
1,5	10	10	10	10	1,9	1,9	10	10	10
1,5	10	10	1,5	10	1,8	1,8	1,8	1,5	10
1,5	10	1,5	1,5	10	1,5	1,5	1,5	10	10
1,8	10	10	1,9	10	1,5	1,5	1,8	10	10
1,5	10	10	10	10	10	10	1,8	10	10
2,5	10	10	10	10	1,6	2,5	1,5	1,6	10
1,5	10	10	10	10	10	10	10	10	10
1,5	10	10	10	10	10	10	1,8	10	10
1,5	10	10	10	10	10	10	10	10	10
2,5	10	10	10	10	2,6	2,5	2,5	2,5	10
1,5	10	10	1,5	10	1,5	1,5	1,5	1,5	10
1,5	1,5	10	10	10	1,9	10	1,9	10	10
1,5	10	10	10	10	10	10	1,9	10	10
1,5	10	10	10	10	10	10	10	10	10
10	10	10	1,5	10	1,5	1,5	1,5	1,5	10
10	10	10	1,5	10	1,5	1,5	1,5	1,5	10
1,5	10	10	10	10	10	10	1,9	10	10
1,4	10	10	10	10	1,9	10	1,8	10	10
1,5	10	10	1,9	10	1,8	1,5	10	10	10
1,5	10	1,5	1,5	10	1,5	1,5	1,5	1,6	10
1,5	10	1,5	1,5	10	1,5	1,5	1,5	1,5	10
2,5	10	10	10	10	10	10	10	10	10
1,5	10	10	10	1,5	1,5	1,5	10	10	10

1,5	10	10	10	1,5	1,5	1,7	10	10	10
1,5	10	10	10	10	10	10	1,9	10	10
1,5	10	10	10	10	10	10	1,9	10	10
2,6	10	10	10	10	2,6	2,5	2,5	2,5	10
2,6	10	10	10	10	2,6	2,5	2,5	2,5	10
1,5	10	10	10	10	10	10	1,9	10	10
1,5	10	10	10	10	10	10	10	10	10
10	10	10	10	10	1,9	1,8	1,8	2,6	10
1,5	10	10	10	10	10	1,6	10	10	10
1,5	10	1,9	1,9	10	2,8	1,5	10	10	10
10	10	10	1,5	10	1,5	10	1,5	10	10
1,5	10	1,5	1,9	10	1,9	1,5	1,8	10	10
1,5	10	10	10	10	10	10	1,9	10	10
1,5	10	10	10	10	10	10	1,9	10	10
1,5	10	10	10	10	10	10	10	10	10
1,5	10	10	10	10	10	10	10	10	10
1,5	10	10	10	10	10	10	10	10	10
1,5	10	10	10	10	10	10	1,9	10	10
1,5	10	10	10	10	10	10	1,9	10	10
1,5	10	10	1,9	10	1,8	1,5	10	10	10
1,5	10	10	10	10	1,5	10	10	10	10
1,5	1,5	10	10	10	1,5	10	1,5	1,5	10
1,5	1,5	10	10	10	1,5	10	1,5	1,5	10
3,6	10	10	10	10	10	1,9	1,8	2,9	10
1,5	10	1,5	1,8	10	10	10	10	10	10
1,5	10	10	1,5	10	1,5	1,5	1,5	1,5	10
2,5	10	10	10	10	2,5	1,5	1,8	2,5	10
1,5	10	10	10	10	10	10	1,9	10	10
1,5	10	10	10	10	1,9	1,5	1,5	2,5	10
1,5	10	10	10	10	1,6	1,5	10	10	10
1,5	10	10	10	10	1,9	1,5	10	10	10
1,6	10	10	10	10	1,5	1,6	10	10	10
1,5	10	10	10	10	1,5	1,5	1,5	1,5	10
1,5	10	1,5	10	10	1,8	1,5	1,9	10	10
1,5	10	10	10	10	10	10	10	10	10
1,5	10	10	10	10	10	10	1,9	10	10
10	10	10	1,5	10	1,5	10	10	10	10
1,5	10	1,9	1,9	1,5	1,9	1,8	10	10	10
1,5	10	10	10	10	10	10	1,9	10	10
1,5	10	1,8	1,9	10	1,5	1,5	1,5	10	10
2,5	10	10	10	10	10	10	10	10	10
1,5	10	10	10	10	10	10	10	10	10
1,5	10	10	10	10	1,8	10	10	10	10
1,5	10	10	10	10	10	1,9	10	10	10
1,5	10	10	10	10	1,8	10	10	10	10
1,6	10	10	10	10	1,6	1,6	1,6	10	10
2,6	10	2,8	10	10	2,5	2,5	1,5	1,5	10

2,6	10	10	10	10	2,5	2,5	1,5	1,5	10
1,5	10	10	10	10	10	10	1,9	10	10
1,5	10	10	1,9	10	1,9	1,5	10	10	10
1,5	1,5	10	10	10	10	10	1,9	10	10
1,5	10	10	10	10	10	10	10	10	10
1,5	10	10	10	10	10	10	10	10	10
10	10	10	10	10	10	10	1,9	10	10
1,5	10	10	10	10	10	10	1,8	10	10
10	10	10	10	10	10	10	1,9	10	10
1,5	10	10	10	10	10	10	1,9	10	10
1,5	10	10	1,5	10	10	10	10	10	10
1,5	10	1,5	10	10	1,5	1,5	1,5	10	10
2,5	10	10	10	10	10	10	10	10	10
2,5	10	10	10	10	10	10	10	10	10
1,5	10	10	10	10	1,9	1,8	10	10	10
10	10	10	10	10	1,7	1,7	1,7	10	10
2,5	10	1,9	10	10	1,9	1,5	1,8	1,5	10
2,5	10	1,9	10	10	1,9	1,5	1,8	10	10
10	10	1,9	1,9	10	1,9	10	10	10	10
1,8	10	1,8	10	10	1,8	1,8	10	10	10
2,6	1,9	10	10	10	10	10	10	10	10
1,5	10	10	10	10	10	10	10	10	10
1,5	10	10	1,9	10	1,5	1,5	10	10	10
2,5	10	1,9	1,9	10	1,9	1,9	10	10	10
2,5	10	1,9	1,9	10	1,9	1,9	10	10	10
1,5	10	10	10	10	1,5	1,5	1,5	1,5	10
1,5	10	10	10	10	1,9	1,8	10	10	10
10	1,5	10	1,9	10	10	10	1,8	10	10
2,5	10	1,9	10	10	10	10	10	10	10
2,5	10	1,9	1,9	10	1,9	10	10	10	10
1,5	10	1,9	1,9	1,5	1,9	1,8	1,8	10	10
1,5	10	10	10	10	1,9	10	10	10	10
1,5	10	10	10	10	10	10	10	10	10
1,5	1,5	1,5	1,5	10	1,5	1,5	10	10	10
10	10	1,5	10	10	1,5	2,6	1,8	10	10
1,5	10	10	10	1,6	1,5	1,5	1,5	10	10
2,5	10	1,5	1,9	10	1,9	10	10	10	10
1,5	10	10	10	10	10	10	10	10	10
1,5	10	1,9	1,6	10	1,6	1,5	10	10	10
1,5	10	1,5	1,9	10	1,9	1,5	1,8	10	10
2,6	10	10	10	10	1,9	10	10	10	10
2,5	10	1,9	1,9	10	1,9	1,9	1,8	10	10
1,5	10	1,9	1,9	10	1,9	1,5	1,8	10	10
1,5	10	1,9	1,9	10	1,9	1,5	1,8	10	10
10	10	1,6	1,5	10	1,5	1,5	1,5	10	1,5
10	10	10	1,5	10	1,5	1,5	1,5	1,5	1,5
1,5	10	1,5	10	10	10	10	1,6	10	10

1,5	1,5	10	1,5	10	1,5	1,5	1,5	10	1,5
1,4	10	10	1,5	10	10	10	1,6	1,5	10
1,5	1,5	10	1,5	1,5	1,5	1,8	1,5	10	10
1,5	10	10	10	1,5	1,9	1,9	1,8	10	10
1,5	1,5	1,5	1,5	1,5	1,5	10	10	10	10
10	10	10	10	10	10	10	10	10	10
10	10	10	10	10	10	10	10	10	10
1,6	10	1,5	1,6	1,6	1,6	1,6	1,6	10	10
1,5	10	10	1,5	1,5	1,9	10	1,6	10	1,9
1,5	10	10	1,5	1,5	1,9	10	1,6	10	1,9
1,5	10	10	10	10	10	10	10	10	10
1,5	1,5	1,8	1,5	1,5	1,8	1,8	10	10	10
1,5	10	10	10	10	10	10	10	10	10
1,5	1,6	1,6	1,5	1,5	1,6	10	1,6	10	10
1,6	10	1,9	1,9	10	1,9	1,5	1,8	10	10
1,5	1,5	1,8	1,6	10	1,5	1,5	1,5	1,5	1,5
1,5	10	10	1,9	10	1,9	1,5	1,8	10	10
1,5	10	10	10	10	10	1,8	10	10	10
1,6	10	1,6	1,6	1,5	10	1,6	1,5	10	10
1,5	10	1,9	10	10	10	10	10	10	10
1,5	10	10	10	10	10	10	10	10	1,5
1,5	10	1,9	10	10	1,9	1,8	10	10	10
1,5	10	1,9	10	10	1,9	1,8	10	10	10
1,5	10	10	1,9	10	1,9	1,8	10	10	10
1,5	10	1,5	1,5	10	1,5	1,5	1,5	10	10
1,5	10	1,5	1,5	10	1,5	1,5	1,5	1,5	10
1,5	10	1,9	10	10	1,9	1,8	1,8	10	10
1,5	10	1,5	1,5	10	1,5	1,5	1,5	1,5	10
1,5	10	10	10	10	1,9	10	10	10	10
1,5	10	1,5	10	10	1,5	10	10	1,5	10
1,5	1,5	10	10	10	10	10	10	10	10
1,5	10	1,5	1,5	10	1,5	1,5	1,5	10	10
1,5	10	1,5	10	10	10	10	10	10	10
1,5	10	1,5	1,5	10	1,5	1,5	1,5	1,5	10
10	10	10	10	10	1,9	1,8	10	10	10
1,5	1,5	1,5	10	10	10	10	1,5	1,5	1,5
10	10	1,9	1,5	10	1,9	1,5	1,5	10	10
1,5	10	1,9	1,8	10	1,9	1,8	10	10	10
1,5	10	1,9	10	10	1,9	1,8	10	10	10
1,5	10	1,9	10	10	1,9	1,8	10	10	10
1,5	10	1,5	10	10	10	10	1,5	10	10
10	10	1,5	10	10	10	10	1,5	10	10
10	10	1,9	1,9	10	1,9	10	1,8	10	10
10	10	10	1,5	10	10	10	1,5	10	10
1,5	10	1,5	1,5	10	1,5	1,5	1,5	1,5	10
10	10	1,5	1,5	10	1,5	1,5	1,5	1,5	1,5
1,5	10	10	10	10	1,9	1,8	1,8	10	1,5

1,5	10	10	10	10	1,9	1,8	1,9	10	1,5
1,5	10	10	1,5	10	1,5	1,5	1,5	10	10
1,5	1,5	1,5	1,5	1,5	1,5	1,5	10	10	10
10	1,5	10	10	2,5	10	10	10	10	10
10	10	10	1,6	10	10	1,5	1,5	1,5	1,5
1,5	10	1,5	1,5	1,5	1,5	1,5	1,5	10	10
1,5	10	10	10	10	1,9	1,8	10	10	1,5
1,5	10	10	10	10	1,9	1,8	1,8	10	1,5
1,8	10	1,5	1,5	10	10	1,5	1,5	10	10
2,5	10	10	10	10	10	10	2,7	1,6	10
1,5	10	10	10	10	1,9	1,8	1,8	10	10
1,5	10	10	10	10	10	1,6	1,5	10	10
1,5	10	10	1,9	10	1,9	1,5	1,8	10	1,5
1,5	10	10	10	10	1,9	1,8	10	10	1,5
1,5	10	10	10	10	1,9	1,8	10	10	1,5
1,5	10	10	10	10	1,9	10	10	10	1,5
1,5	10	10	10	10	1,9	10	10	10	1,5
1,5	10	10	10	10	1,9	1,8	10	10	1,5
10	10	10	1,6	10	10	1,5	1,5	1,5	1,5
1,5	10	10	1,5	10	1,5	1,5	1,5	10	10
1,5	10	10	1,5	10	10	10	1,6	1,5	10
1,5	10	10	10	10	1,9	1,5	10	10	10
1,5	10	10	10	10	1,9	1,8	10	10	1,5
1,5	1,5	1,5	1,5	1,5	1,5	1,5	1,5	10	10
1,5	10	10	10	10	1,9	1,8	10	10	10
1,5	10	10	10	10	1,9	1,8	1,8	10	1,5
1,5	10	10	1,9	10	1,9	1,8	1,8	10	1,5
1,2	10	10	10	10	1,8	1,8	10	10	1,5
1,5	10	10	10	10	1,9	1,8	10	10	10
1,5	10	10	10	10	1,9	1,8	1,8	10	1,5
1,5	10	10	10	10	10	1,8	10	10	10
10	10	10	10	10	1,9	1,9	10	10	10
10	10	10	10	10	10	10	10	10	10
1,5	10	10	1,9	10	1,9	10	1,8	10	10
10	10	10	10	10	1,8	1,8	10	10	10
1,5	10	10	10	10	1,5	1,5	1,8	1,6	10
10	10	10	10	10	1,6	3,6	1,6	10	1,5
1,5	1,5	1,5	1,5	1,5	1,5	1,5	1,5	10	10
1,5	10	1,5	1,5	10	1,5	1,5	1,5	1,5	10
1,5	10	1,5	1,5	10	1,5	1,5	1,5	1,5	10
1,6	10	10	1,5	10	10	10	1,5	10	10
1,5	10	10	1,9	10	1,9	1,8	1,8	1,8	10
2,6	10	10	10	10	10	10	1,5	10	10
1,5	10	1,5	1,5	10	10	10	1,5	10	10
10	10	10	10	10	10	10	10	10	10
1,5	10	10	10	10	10	1,6	10	10	10
10	10	10	10	10	10	10	10	10	10

1,5	10	10	10	10	1,8	10	10	1,9	10
1,5	10	10	10	10	10	10	10	10	10
1,5	1,5	1,5	1,5	1,5	1,5	10	1,5	10	10
10	10	10	10	10	10	1,5	10	10	10
10	10	10	10	10	10	1,8	10	10	10
10	10	10	10	10	10	10	10	10	10
1,5	10	10	1,9	10	1,9	1,5	1,8	1,5	10
1,5	10	1,9	1,9	10	1,9	1,9	10	1,8	10
1,5	10	10	10	10	10	10	10	10	10
10	10	10	1,5	10	1,8	1,8	1,8	1,8	10
10	10	1,5	1,6	10	1,5	1,5	1,5	10	10
1,5	1,5	10	10	10	10	1,5	1,5	10	10
1,5	10	10	10	10	1,9	1,8	1,8	10	10
1,5	10	10	10	10	10	10	10	10	10
1,5	10	10	10	10	1,9	1,8	1,8	10	10
1,5	10	10	10	10	10	1,5	1,5	10	10
10	10	10	1,6	10	1,5	1,5	1,5	10	10
10	10	1,5	1,5	10	10	10	1,5	10	10
1,6	10	10	1,5	10	1,5	1,5	1,5	1,5	10
10	10	10	1,6	10	1,5	1,5	1,5	10	10
1,5	10	10	10	10	10	10	10	10	10
1,5	10	1,5	1,5	1,5	1,5	1,9	10	10	10
10	10	10	1,5	10	10	10	10	10	1,5
1,5	10	1,9	10	10	1,9	1,8	1,5	10	1,5
1,5	1,5	1,5	10	10	10	10	10	10	1,5
10	10	10	1,5	10	10	10	10	10	1,5
1,5	1,5	1,6	1,6	1,6	1,6	10	1,7	1,7	1,6
10	10	1,5	1,5	10	10	10	1,5	1,5	1,5
10	10	10	1,6	10	10	10	10	10	1,5
10	10	10	1,6	10	10	10	10	1,5	10
1,5	1,5	1,5	1,5	1,5	1,6	1,6	1,6	1,5	10
1,5	10	1,9	1,9	10	1,9	1,9	1,8	10	1,5
1,5	10	1,5	1,5	10	1,5	1,5	1,5	1,5	10
1,5	10	1,9	10	10	1,9	1,8	1,8	10	1,5
10	10	10	10	10	10	10	10	10	10
1,5	10	10	10	10	1,9	10	10	10	10
10	10	10	1,5	10	1,5	1,5	1,5	1,5	1,5
10	10	10	1,5	10	1,5	1,5	1,5	1,5	1,5
10	1,5	1,5	1,5	10	1,5	1,5	1,5	1,5	10
1,5	1,5	1,5	1,5	10	1,5	1,5	1,5	1,5	1,5
1,5	10	10	10	10	1,5	1,5	1,5	1,5	1,5
1,6	1,6	10	1,6	1,6	1,6	1,6	1,7	10	1,5
1,5	10	1,5	1,5	10	1,5	1,5	1,6	1,6	10
10	10	10	1,5	10	10	10	1,5	10	1,5
1,5	10	10	10	10	1,5	1,8	10	10	1,5
1,5	10	1,5	1,9	10	1,9	1,5	1,8	1,5	1,5
1,5	1,5	10	1,5	10	10	10	10	10	1,5

1,5	10	10	10	10	10	1,8	1,5	10	10
1,5	10	10	10	10	10	1,8	1,5	10	10
10	10	10	10	10	10	10	10	10	10
10	10	10	10	10	10	10	10	10	10
10	10	10	10	10	10	10	10	10	10
10	10	10	10	10	1,8	1,8	10	10	10
1,5	10	10	10	10	10	10	1,9	10	10
1,6	10	10	10	10	1,5	1,6	1,5	1,5	10
1,6	10	10	10	10	1,5	1,5	1,5	1,5	10
1,5	10	10	10	10	10	10	1,5	10	1,5
1,5	10	1,5	1,5	10	1,5	1,5	1,6	1,6	10
1,5	10	10	10	10	10	10	1,6	10	1,9
1,5	10	1,9	1,9	10	1,9	1,5	1,8	10	1,5
1,5	10	1,5	10	10	1,5	1,5	1,6	10	10
10	10	10	10	10	1,8	10	10	10	10
1,6	10	10	10	10	10	10	1,5	10	10
1,5	10	10	10	10	10	10	1,5	10	1,5
1,5	10	10	10	10	10	1,5	1,5	10	10
10	10	10	10	10	1,8	1,8	10	10	10
2,8	2,8	1,6	1,9	1,9	10	10	10	10	10
1,5	10	10	10	10	10	1,8	10	10	10
1,5	10	10	10	10	10	10	1,5	10	1,5
1,5	10	10	10	10	10	10	10	10	10
10	10	10	1,6	10	1,8	1,5	1,5	1,5	1,5
1,5	10	10	10	10	10	10	10	10	1,5
2,7	2,6	1,8	1,7	10	1,7	10	10	1,9	10
1,5	10	10	1,9	10	1,9	1,5	10	1,5	1,5
1,5	10	1,5	1,5	10	1,5	10	10	10	1,5
1,5	10	1,5	10	10	1,5	1,5	1,5	1,5	1,5
1,5	1,5	1,5	1,5	10	1,5	1,5	1,5	10	1,5
10	10	10	10	10	10	10	10	10	10
1,5	10	10	1,5	10	1,8	1,8	10	10	1,5
1,5	1,5	10	2,6	10	1,5	1,5	1,8	1,5	1,5
10	10	10	10	10	10	10	10	10	10
1,5	1,5	10	2,6	10	1,5	1,5	1,8	1,5	1,5
1,5	10	1,5	1,5	10	1,5	1,5	1,6	10	10
1,5	10	10	10	10	10	10	10	10	10
1,5	10	10	10	10	1,9	1,9	1,8	10	1,5
1,5	10	10	10	10	10	10	1,8	10	10
10	10	1,5	10	10	1,5	1,5	1,5	1,5	1,5
1,5	10	10	1,9	10	1,9	1,5	1,6	10	1,5
1,6	10	10	10	10	1,5	10	1,8	10	10
1,5	10	10	10	10	10	10	10	10	10
10	10	10	10	10	10	10	10	10	10
10	10	10	10	10	1,5	1,5	10	10	10
1,5	10	1,9	10	10	1,9	1,8	10	2,5	10
1,5	10	10	10	10	1,6	1,5	1,8	10	10

1,5	10	10	10	10	10	10	10	10	10
1,5	1,5	10	1,5	10	1,5	1,5	1,5	2,5	1,5
1,5	10	10	10	10	1,8	10	10	10	10
10	10	10	1,5	10	10	10	10	10	1,5
1,5	1,5	10	10	10	10	1,6	1,6	10	10
10	10	10	10	10	1,8	1,8	10	10	10
1,5	1,5	10	1,5	10	1,5	10	10	1,5	1,5
1,5	10	10	10	10	10	1,5	1,5	10	10
1,5	10	10	10	10	1,8	1,8	10	10	10
1,5	10	10	10	10	1,5	1,5	1,5	1,5	10
10	10	1,5	1,6	10	10	10	10	10	1,5
1,5	10	1,5	1,5	10	1,5	1,5	1,5	10	1,5
10	10	10	10	10	1,8	1,8	10	1,9	10
10	10	10	10	10	1,8	1,5	10	1,9	10
1,5	10	1,5	1,9	10	1,9	1,5	1,8	1,8	1,5
10	10	10	10	10	10	10	10	10	10
1,5	10	10	10	10	1,9	1,5	1,8	10	1,5
1,5	10	10	10	10	1,9	1,5	1,8	10	1,5
1,5	10	1,8	1,9	10	1,9	1,5	10	1,8	1,5
10	10	1,9	1,9	10	1,9	1,5	10	10	1,5
10	10	1,8	1,6	10	10	10	10	10	1,5
10	10	10	10	10	10	10	10	10	10
1,5	10	10	10	10	10	10	10	10	1,5
1,5	10	10	10	10	10	10	10	10	10
1,5	10	10	10	10	10	1,9	1,8	10	10
10	1,6	10	10	10	10	10	10	10	10
1,5	10	10	10	10	10	10	1,7	10	10
1,5	1,5	1,9	1,5	1,5	1,5	1,8	1,5	1,5	10
10	10	1,9	1,5	10	1,5	1,5	1,5	1,9	1,5
1,5	10	1,5	1,5	10	1,5	1,5	1,5	1,5	10
1,5	10	1,9	1,9	10	1,9	1,5	1,8	10	1,5
1,5	10	1,5	1,5	10	10	10	10	10	10
1,5	10	10	1,9	10	1,9	10	1,8	10	10
1,5	1,5	1,5	1,5	1,5	1,5	1,5	10	10	10
1,5	1,6	1,6	10	10	1,6	1,6	1,6	1,5	10
1,5	10	1,9	10	10	1,9	10	10	10	1,5
1,5	10	1,9	1,9	10	1,8	10	10	10	10
1,6	1,5	1,5	1,6	1,6	1,6	1,6	1,6	10	10
1,5	1,5	10	1,5	10	1,5	1,5	1,5	10	1,5
1,5	10	1,9	1,9	10	1,9	1,5	1,8	10	1,5
1,5	1,5	1,5	1,5	1,5	1,5	10	1,5	10	10
1,5	10	1,9	10	10	1,9	1,9	10	10	10
1,5	10	1,9	10	10	1,9	1,9	10	10	10
1,5	10	1,5	1,5	10	1,5	1,5	1,5	1,5	10
1,5	10	1,9	10	10	1,9	10	10	10	10
1,5	10	10	10	10	1,9	1,8	1,8	10	1,5

canned pe	apple	banana	orange	avocado	granadilla	lemon	litchi	plums	pawpaw
10	1,5	1,5	1,9	2,9	1,6	1,4	2,6	1,5	1,6
10	1,5	1,5	1,9	2,9	1,6	1,4	2,6	1,5	1,6
10	1,6	1,5	1,5	1,5	1,5	1,5	1,6	1,6	1,9
10	2,5	2,5	2,5	1,9	10	10	1,9	1,8	1,8
10	1,6	1,5	1,5	1,9	10	10	1,9	10	1,5
10	1,6	1,6	1,6	1,9	10	10	10	1,6	10
10	1,5	1,8	1,9	10	1,9	1,5	1,9	10	1,5
10	1,6	1,5	1,9	1,5	10	10	10	10	1,9
10	1,5	1,9	1,9	1,9	1,9	1,9	1,9	1,8	1,9
10	2,5	2,5	2,5	10	10	1,5	1,9	10	10
10	1,7	1,7	1,9	1,9	10	10	2,7	10	10
1,8	1,5	1,5	1,5	10	1,5	1,5	1,5	1,5	1,5
10	1,5	1,5	2,9	2,9	1,5	2,5	2,9	1,5	2,9
10	1,5	1,5	1,5	1,9	1,9	1,8	10	10	1,8
10	1,7	1,7	1,5	1,9	10	10	1,9	10	1,9
10	1,6	1,6	1,6	1,9	10	10	10	10	10
10	2,6	2,6	2,6	1,9	10	10	10	10	10
10	1,5	1,5	1,5	2,5	1,5	1,6	1,6	1,5	2,6
10	1,5	1,5	1,6	1,6	1,5	1,6	1,5	1,5	2,9
10	1,5	1,5	2,6	3,6	1,5	1,5	2,5	1,5	2,6
10	1,5	1,5	1,5	2,5	1,5	1,5	1,5	1,5	2,5
10	2,6	1,5	2,6	2,6	1,5	1,5	2,9	1,5	2,6
10	1,5	1,5	1,5	2,9	1,5	1,5	1,9	1,5	2,6
10	1,5	1,5	1,6	2,9	1,5	1,5	1,9	1,5	1,9
10	2,5	1,5	2,8	2,8	1,8	1,5	2,5	1,5	2,9
10	1,6	10	1,6	10	1,5	1,6	1,8	1,5	10
10	1,6	2,6	1,6	10	10	10	10	10	1,6
10	1,6	1,6	1,9	1,9	10	10	10	10	1,9
1,5	1,7	1,7	1,7	1,5	1,5	1,5	1,9	10	1,5
10	2,6	1,5	2,9	1,9	2,7	10	10	10	10
10	1,5	1,5	1,5	10	10	1,5	1,9	10	10
10	1,9	1,9	1,9	1,9	10	10	1,9	10	2,5
10	1,5	1,9	1,9	1,9	1,5	1,5	1,9	1,8	1,8
10	1,5	1,8	1,9	1,9	1,9	1,9	1,9	10	1,9
10	1,5	1,5	10	1,9	10	1,5	1,9	10	1,9
10	2,7	2,7	2,7	1,7	10	10	1,5	10	1,5
10	1,5	1,5	1,9	1,9	10	10	1,9	10	10
10	2,8	2,8	1,9	1,9	10	10	10	10	10
10	1,5	1,5	1,5	1,5	1,5	1,5	1,5	1,9	1,9
10	1,8	1,8	1,8	1,7	1,9	1,8	10	10	10
1,6	1,6	1,6	1,6	1,6	1,6	1,6	1,6	10	1,6
10	1,5	1,6	1,6	1,5	1,5	1,5	1,5	1,5	1,5
10	1,6	1,6	1,6	1,9	10	10	10	10	10
10	1,6	1,6	1,6	1,6	10	1,6	10	10	1,6
1,8	1,8	1,8	1,8	10	1,9	10	1,9	1,8	1,9
10	1,5	1,5	1,9	1,9	1,9	1,9	10	10	1,9

10	1,5	1,5	1,9	1,9	1,9	1,9	10	10	1,9
10	1,8	1,8	1,8	1,9	1,9	1,9	1,9	10	1,9
10	1,5	1,5	1,5	10	1,5	1,5	1,9	10	10
10	1,7	1,7	1,7	1,7	1,7	1,7	1,5	10	1,5
10	1,6	1,6	1,9	2,9	10	1,9	1,5	10	1,5
10	2,7	1,6	2,7	1,9	10	10	10	10	1,8
10	1,5	1,5	1,5	1,9	10	10	1,9	10	1,5
10	2,7	2,7	2,7	10	10	10	10	10	10
10	3,7	3,7	3,7	10	10	10	10	10	10
10	1,7	10	1,7	1,9	10	10	1,9	10	10
10	2,7	2,7	2,7	1,5	10	10	10	10	10
10	1,7	1,7	1,7	10	10	10	10	10	10
10	2,7	2,7	2,7	1,9	10	1,5	1,9	10	10
1,5	1,6	1,5	1,5	1,5	1,5	1,5	1,5	1,6	1,5
10	1,6	1,6	1,9	1,9	10	10	1,9	1,9	1,9
10	1,7	1,7	1,7	1,9	10	1,7	10	2,8	1,8
10	1,6	1,6	1,6	1,9	10	10	10	10	1,8
10	1,5	1,5	1,4	1,4	1,5	10	1,9	10	10
10	1,5	1,8	1,8	1,9	10	10	10	10	10
10	1,8	1,8	1,8	10	10	10	1,5	10	10
10	1,6	1,6	1,6	1,9	1,5	1,5	1,9	1,5	2,6
10	1,5	1,5	1,5	1,5	1,9	10	1,5	1,8	1,6
10	1,5	1,5	1,5	1,9	1,9	10	1,5	1,8	1,6
10	1,5	1,8	1,9	1,9	10	10	1,9	10	1,5
10	1,5	1,8	1,9	1,9	10	10	1,9	10	1,5
10	1,6	1,6	2,6	1,9	10	10	10	10	10
10	1,6	1,6	1,6	1,9	10	1,5	1,9	10	10
10	1,5	1,5	1,9	1,9	10	1,9	10	10	1,9
10	1,6	1,6	1,9	1,9	1,5	10	1,9	10	1,5
10	1,5	1,5	2,9	2,9	10	10	10	10	10
10	1,8	1,8	1,9	1,9	10	10	10	10	1,9
10	1,8	1,8	1,9	1,9	10	10	10	10	1,9
10	1,5	1,5	1,5	10	10	10	10	10	10
2,8	1,6	1,6	1,9	1,9	10	10	1,5	10	1,5
10	2,7	1,5	1,5	1,5	10	10	2,9	1,5	10
10	1,5	1,5	1,5	1,9	1,5	10	1,5	1,5	1,5
10	1,5	1,9	1,5	1,9	1,5	1,5	1,9	1,5	1,9
10	1,5	1,5	1,9	1,9	10	10	10	10	10
10	2,7	2,7	2,7	1,9	10	1,8	1,8	10	10
10	1,5	1,6	2,5	1,5	10	1,5	1,5	10	1,5
1,8	1,5	1,8	1,9	1,9	10	10	1,9	10	1,9
10	1,5	1,5	1,5	1,9	1,9	1,9	1,9	10	1,9
10	1,6	1,6	1,6	1,9	10	1,8	1,9	10	1,9
1,5	1,6	1,5	1,5	1,8	1,8	1,5	1,9	10	1,9
10	1,5	1,5	1,5	1,9	10	1,8	1,9	10	1,9
10	1,6	1,8	1,8	1,9	10	1,8	1,9	10	1,9
10	1,5	1,5	1,5	1,6	1,9	1,6	1,6	1,5	1,9

10	1,7	1,7	1,9	1,9	10	10	10	1,9	1,9
10	1,6	10	1,8	1,7	10	1,8	10	10	10
10	1,7	1,7	1,9	2,9	1,9	10	10	10	1,6
10	1,6	1,6	1,6	1,9	10	10	10	10	1,8
10	1,5	1,6	1,9	1,9	10	1,5	1,9	10	10
10	1,5	2,5	2,5	2,5	10	10	2,5	10	1,5
1,5	1,5	1,5	1,5	1,9	1,5	1,5	1,5	1,5	1,9
10	1,5	1,5	1,5	1,9	10	1,9	1,9	10	1,9
10	1,5	1,5	1,5	1,9	10	1,8	1,8	10	1,8
10	1,4	1,6	1,6	1,9	10	1,5	1,9	1,8	3,9
10	2,8	2,8	2,8	2,9	10	1,5	10	10	1,8
10	1,5	1,5	1,9	1,9	1,9	1,9	1,9	1,9	1,9
10	2,7	2,7	2,7	1,9	1,9	1,7	1,9	10	1,9
10	2,6	2,6	2,6	10	10	10	2,5	10	10
10	1,7	1,7	1,7	1,8	10	1,5	1,5	10	1,8
10	1,5	1,5	1,5	1,9	1,5	1,5	1,9	1,9	1,9
10	1,8	1,8	1,9	1,9	10	10	10	10	10
10	1,7	1,6	1,6	10	10	10	10	10	10
10	1,5	3,5	1,5	10	10	10	1,9	10	1,5
10	1,5	1,5	1,9	1,9	1,9	1,9	1,9	10	1,9
10	1,5	1,5	1,5	1,5	1,5	1,5	1,5	1,5	1,5
1,8	1,8	1,8	1,8	1,9	1,9	1,5	1,9	1,8	1,9
10	1,5	1,5	1,5	1,5	1,5	1,5	1,5	10	1,5
10	1,5	1,5	1,5	1,5	1,5	1,5	1,5	10	1,5
10	1,5	1,5	1,5	1,5	1,5	1,5	1,5	10	1,5
1,5	1,6	1,6	1,5	1,9	1,9	1,9	1,9	1,8	1,5
10	1,6	1,6	2,7	1,7	10	1,5	2,6	10	1,6
10	1,5	1,5	1,5	10	10	1,8	10	10	1,5
10	1,5	1,5	1,9	1,9	1,9	10	1,9	10	1,9
10	1,5	1,5	1,5	1,9	1,5	1,5	1,5	1,5	1,9
10	2,7	2,7	2,7	2,9	2,7	2,5	10	10	1,5
10	1,7	1,7	1,7	1,6	10	1,7	1,5	1,5	1,5
10	1,5	1,5	1,5	1,9	1,5	1,5	1,5	1,5	1,9
10	1,5	1,5	2,9	2,9	10	1,7	10	1,8	1,5
10	1,5	1,5	1,9	1,9	10	1,9	1,9	10	1,8
10	1,5	1,5	2,9	2,9	10	1,7	1,7	10	1,6
1,7	2,6	2,6	2,6	1,9	2,6	1,6	1,6	1,7	1,7
1,5	1,5	1,5	1,5	1,9	1,9	10	1,9	10	1,9
10	1,6	1,6	2,9	2,9	1,8	1,8	1,8	10	1,9
1,5	1,6	1,6	2,9	2,9	10	10	10	10	1,9
1,5	1,5	1,5	1,5	1,9	1,9	10	1,9	10	10
1,5	1,5	1,5	1,5	1,5	1,5	1,8	1,8	10	1,5
10	2,6	2,6	2,6	1,9	10	10	10	10	1,8
10	1,5	1,5	1,5	1,6	1,5	10	10	10	10
10	1,5	1,5	1,5	2,9	1,5	1,5	1,9	1,9	2,9
10	1,6	1,6	1,6	1,6	1,5	1,9	1,6	10	1,9
10	1,6	1,6	1,5	1,9	1,5	1,6	1,6	1,5	2,5

10	1,5	1,5	1,9	1,9	1,9	1,9	1,9	10	1,9
1,5	1,6	1,5	1,9	1,9	1,9	1,9	1,9	1,9	1,9
10	1,6	1,6	1,6	1,9	10	1,9	1,9	10	1,9
10	1,6	1,6	1,6	1,9	10	1,9	1,9	10	1,9
10	1,7	1,7	1,7	1,9	10	10	10	10	1,8
1,8	1,5	1,5	1,9	1,9	1,8	1,8	1,9	1,5	1,8
10	1,5	1,5	1,5	1,9	1,5	1,6	1,6	1,5	2,9
10	1,6	1,5	1,6	1,5	10	1,5	1,9	10	1,5
10	1,6	1,5	1,9	1,9	10	10	1,9	10	1,9
10	1,7	1,5	1,5	1,9	10	1,9	1,9	10	1,6
10	1,5	1,5	1,5	1,9	1,9	1,9	1,9	10	1,8
10	1,5	1,5	1,5	1,9	1,9	1,9	1,9	10	1,8
10	1,6	1,6	1,9	1,9	1,9	1,9	1,9	10	1,9
10	1,6	1,6	1,6	1,6	10	1,5	1,9	10	1,5
10	1,5	1,5	1,5	1,6	10	1,5	1,5	10	1,9
10	1,5	1,5	1,5	1,5	10	10	1,5	10	10
10	1,5	1,5	1,5	1,5	10	10	1,5	10	10
10	1,7	1,6	1,6	1,6	10	10	1,5	10	10
10	1,5	1,5	1,5	2,5	1,5	1,5	1,5	1,5	10
10	1,5	1,5	1,9	10	10	1,5	1,5	10	1,8
10	1,6	1,5	1,9	1,9	1,9	10	10	10	1,9
10	1,8	1,8	1,8	1,9	10	10	1,9	10	10
10	1,8	1,8	1,8	1,9	10	10	1,9	10	10
10	1,6	1,6	1,6	1,9	10	1,8	1,9	10	1,9
10	1,6	1,6	1,6	1,9	10	1,8	1,9	10	1,9
10	1,6	1,5	1,5	1,5	10	10	1,5	1,5	1,5
10	1,7	1,5	1,8	1,9	10	10	1,9	10	1,8
10	1,7	1,7	1,7	1,7	10	1,7	10	10	1,8
10	1,6	1,6	1,9	1,9	1,9	1,9	1,9	10	1,9
10	1,5	1,5	1,5	2,6	10	1,5	1,5	1,5	2,6
10	1,8	1,8	1,9	1,9	1,9	10	10	10	2,9
10	1,5	1,5	1,9	1,9	1,9	1,9	1,9	10	1,9
10	1,5	1,5	1,9	1,9	10	1,9	1,9	10	10
10	1,5	1,5	1,5	1,9	1,5	1,5	1,5	1,5	2,5
10	1,5	1,5	1,5	1,5	10	10	1,5	10	1,5
10	1,5	1,5	1,9	1,9	1,9	1,9	1,9	10	1,9
10	1,5	1,5	1,9	1,9	1,9	1,9	1,9	10	10
10	1,5	1,9	1,9	1,9	1,9	1,9	1,9	10	1,9
10	1,5	1,5	1,5	1,5	10	10	1,9	10	1,9
10	1,5	1,5	1,5	1,5	10	10	1,9	10	1,9
10	1,5	1,5	1,9	1,9	10	1,9	1,9	10	1,8
10	1,5	1,5	1,5	1,9	10	1,8	1,9	10	1,9
10	1,7	1,7	1,7	10	10	10	1,9	10	1,8
1,6	1,6	1,6	1,6	1,9	1,9	1,8	1,9	1,5	1,5
10	1,6	1,6	1,6	1,9	10	1,8	1,9	10	1,8
10	1,5	1,5	1,9	10	10	10	1,9	1,8	1,8
10	1,7	1,7	1,7	1,9	10	1,5	10	10	10

1,8	1,7	1,7	1,7	1,9	10	10	10	10	1,6
10	1,5	1,5	1,9	1,9	1,9	1,9	1,9	10	1,9
10	1,5	1,5	1,9	1,9	1,9	10	1,9	10	1,9
10	1,5	1,5	1,5	1,9	1,5	1,5	1,5	1,5	1,6
10	1,5	1,5	1,5	1,9	1,5	1,5	1,5	1,5	1,6
10	1,5	1,5	1,9	1,9	1,9	1,9	1,9	10	1,9
10	1,6	1,6	1,9	1,9	1,9	1,9	1,9	10	1,9
10	1,7	1,7	1,8	1,9	10	10	1,9	10	2,9
10	1,5	1,5	1,9	1,9	10	10	10	10	10
10	2,7	2,7	2,7	10	10	10	10	10	10
10	1,5	1,5	1,5	1,5	10	10	1,6	10	1,5
10	1,6	1,6	1,6	1,9	10	10	1,9	10	1,9
10	1,5	1,5	1,9	1,9	10	1,9	10	10	1,9
10	1,5	1,5	1,9	1,9	10	1,9	1,9	10	10
10	1,5	1,5	1,5	1,5	10	1,5	1,5	10	1,5
10	1,5	1,5	1,9	1,9	10	1,9	10	10	1,9
10	1,5	1,5	1,5	1,5	10	10	1,9	10	1,5
10	1,5	1,5	1,9	1,9	10	1,9	10	10	1,9
10	1,5	1,5	1,9	1,9	10	1,9	1,9	10	10
10	3,7	3,7	3,7	10	10	10	10	1,8	1,8
10	1,5	1,5	1,9	1,9	10	1,9	1,9	1,9	1,9
10	1,5	1,5	1,5	1,6	10	10	1,5	10	1,5
10	1,5	1,5	1,5	1,6	10	10	1,5	10	1,5
10	1,8	1,8	10	10	10	10	10	10	10
10	1,8	1,8	1,8	1,9	10	10	10	10	1,9
10	1,6	1,6	1,6	1,9	10	10	1,9	10	1,6
10	1,5	1,5	1,5	10	10	1,8	1,5	1,8	2,6
10	1,5	1,5	1,9	1,9	1,9	1,9	1,9	10	1,9
10	1,7	1,7	1,7	1,9	10	10	10	10	1,9
10	2,7	2,7	2,7	1,9	10	10	10	10	1,9
10	1,5	1,5	1,5	1,9	10	10	1,9	10	1,9
10	1,6	1,6	1,8	1,8	10	1,9	1,9	10	1,9
10	1,5	1,5	1,5	1,9	10	10	1,9	10	1,5
1,5	1,5	1,5	1,5	1,9	10	1,8	1,8	10	1,9
10	1,5	1,5	1,9	1,9	10	10	10	10	10
10	1,5	1,5	1,9	1,9	1,9	1,9	1,9	10	1,9
10	1,5	1,5	2,5	1,5	10	10	10	10	1,5
10	1,7	1,7	1,7	1,9	10	10	10	10	10
10	1,5	1,5	1,9	1,9	10	10	1,9	10	1,9
10	1,5	1,5	1,5	1,5	10	1,5	1,5	1,8	1,9
10	1,5	1,5	1,5	1,9	10	10	1,9	1,8	1,9
10	1,5	1,5	2,9	1,9	10	10	1,9	10	1,9
10	3,9	3,9	3,9	1,9	10	10	10	10	1,9
10	1,7	1,5	1,9	1,9	10	10	10	10	10
10	3,7	3,7	3,7	1,9	10	10	10	10	1,9
10	1,6	1,6	2,9	2,9	10	1,8	1,8	10	1,9
10	1,5	1,5	1,5	1,6	1,5	10	1,5	1,5	1,6

10	1,5	1,5	1,5	1,6	1,5	10	1,5	1,5	1,6
10	1,5	1,5	1,9	1,9	1,9	1,9	1,9	10	10
1,5	1,6	1,6	1,6	1,9	10	1,8	1,9	10	1,9
10	1,5	1,5	1,9	1,9	10	10	10	10	1,9
10	1,5	1,5	1,9	1,9	1,9	1,9	1,9	10	1,9
10	1,5	1,5	1,8	1,8	10	10	1,8	10	1,9
10	1,5	1,5	1,9	1,9	1,9	1,9	1,9	10	1,9
10	1,5	1,5	1,9	1,9	1,9	1,9	1,9	10	1,9
10	1,6	1,6	1,9	1,9	1,9	1,9	1,9	1,9	10
10	1,5	1,5	1,9	1,9	1,9	1,9	1,9	10	1,9
10	1,5	1,5	1,5	1,5	10	1,5	1,5	10	1,9
10	1,6	1,6	1,6	1,9	1,9	1,8	1,9	10	1,6
10	1,6	1,6	1,9	1,9	10	10	10	10	10
10	1,6	1,6	1,9	1,9	10	10	10	10	10
10	1,5	1,5	1,8	1,9	1,5	1,5	1,9	10	10
10	1,5	1,5	2,9	2,9	10	1,8	1,8	10	1,7
10	1,5	1,6	1,6	2,6	1,9	1,5	2,9	1,5	2,9
10	1,5	1,5	1,6	2,6	1,9	1,5	2,9	1,9	2,9
10	1,6	1,5	1,9	1,9	10	1,9	1,9	10	1,9
10	1,7	1,7	2,9	2,9	10	2,7	1,9	10	1,9
10	1,8	1,8	1,8	1,9	10	10	10	10	10
10	1,6	1,5	1,8	10	10	1,6	1,9	10	10
10	2,7	2,7	3,8	1,9	10	1,9	1,9	10	3,9
10	1,6	10	1,8	1,9	10	1,5	1,9	10	10
10	1,6	10	1,8	1,9	10	1,5	1,9	10	10
10	1,7	1,7	1,7	1,7	1,9	1,8	1,8	1,9	1,9
10	1,5	1,5	1,8	1,9	10	1,5	1,9	10	10
10	1,7	1,5	1,5	1,9	10	1,8	1,9	10	1,9
10	1,7	1,7	1,9	1,9	1,9	1,9	1,9	10	10
10	1,6	1,6	2,8	1,9	10	1,9	1,9	1,5	1,9
10	1,5	1,6	1,5	1,9	10	1,5	1,9	1,5	1,9
10	1,7	1,7	1,8	1,9	1,9	1,9	1,9	10	1,5
10	1,6	1,6	1,9	1,9	1,9	1,9	1,9	10	1,9
10	1,5	1,5	1,5	1,9	1,9	1,8	1,9	10	1,5
10	1,5	1,5	2,5	3,6	2,5	1,9	2,9	1,5	2,6
10	1,5	1,5	1,9	2,9	10	1,8	1,8	10	1,8
10	1,8	1,8	1,9	1,9	10	1,9	1,9	10	1,9
10	1,5	1,5	1,9	1,9	1,9	1,9	1,9	10	1,9
10	1,6	1,6	1,6	1,6	10	1,6	10	1,9	1,9
10	1,5	1,5	1,5	1,9	1,9	1,8	1,9	10	1,9
10	1,5	1,5	1,6	1,9	1,9	1,6	1,9	10	1,9
10	2,7	1,9	1,9	1,9	10	1,5	1,9	1,8	1,9
10	1,6	1,6	1,9	1,9	1,9	1,5	1,9	10	1,8
10	1,6	1,6	1,9	1,9	1,9	1,5	1,9	10	1,8
10	1,9	1,9	1,9	1,9	1,9	1,9	1,9	10	1,9
10	2,9	1,9	1,9	1,9	10	1,9	1,9	10	1,9
10	1,5	1,5	1,5	1,5	10	10	10	10	1,6

10	1,9	1,9	1,9	1,9	1,9	1,9	1,9	1,9	1,9
10	1,5	1,5	1,5	1,9	1,9	1,9	1,9	10	1,9
10	1,5	1,5	1,5	1,9	1,5	10	10	10	1,5
1,8	1,7	1,7	1,9	1,9	10	1,9	1,9	1,5	1,9
10	1,5	1,5	1,5	1,5	1,5	1,5	10	10	1,5
10	1,6	1,5	1,5	1,9	1,9	1,5	10	10	10
10	1,5	1,5	1,5	1,9	10	10	10	10	10
10	1,5	1,5	1,5	1,5	1,5	1,5	1,5	1,5	1,5
1,9	1,5	1,5	2,6	1,9	10	10	10	10	1,6
1,9	1,5	1,5	2,6	1,9	10	10	10	10	1,6
10	1,7	10	1,5	10	10	10	10	10	10
10	1,5	1,5	1,5	1,9	1,8	1,5	1,5	10	1,5
10	1,6	1,5	1,5	1,9	1,9	1,9	1,9	10	1,9
10	1,6	1,6	1,6	1,6	10	10	10	1,5	1,5
1,8	1,7	1,7	1,9	1,9	10	1,5	1,9	10	1,8
10	2,9	2,9	1,9	1,9	1,9	1,9	1,9	10	1,9
1,8	1,7	1,7	1,9	1,9	10	1,9	1,9	10	10
10	1,7	1,7	1,5	1,9	1,9	1,9	1,9	10	1,5
10	1,6	1,6	1,6	1,7	1,6	1,6	1,6	10	1,6
10	1,5	1,5	1,5	1,9	1,9	1,9	1,9	1,9	1,9
1	1,5	1,5	1	10	10	1,9	1,9	1,8	1,8
10	1,7	1,7	1,9	10	10	10	1,9	10	10
10	1,7	1,7	1,9	10	10	1,9	1,9	1,8	1,8
10	1,7	1,7	1,9	1,9	1,9	1,9	1,9	10	1,8
1,5	1,5	1,5	1,5	1,9	1,9	1,9	1,9	1,9	1,9
10	1,5	1,5	1,5	1,9	1,9	1,9	1,9	1,9	1,9
10	1,8	1,8	1,9	10	10	10	1,9	10	1,8
1,5	1,5	1,5	1,5	1,9	1,9	1,9	1,9	10	1,9
10	1,7	1,7	1,9	1,9	1,9	1,9	1,9	10	1,8
1,5	1,5	1,5	1,5	1,9	1,9	1,9	1,9	10	1,8
10	1,5	1,5	1,9	1,9	1,9	1,9	1,9	1,5	1,5
10	1,5	1,5	1,5	10	10	10	10	10	1,5
10	1,5	10	1,9	10	10	10	10	10	10
1,5	1,5	1,5	1,5	1,9	1,9	1,9	1,9	10	1,9
10	1,7	1,7	1,9	1,9	10	10	10	10	1,5
1,5	1,5	1,5	1,5	1,9	1,9	1,9	1,9	10	1,9
1,8	1,7	1,6	1,7	1,9	10	10	1,9	1,8	1,9
10	1,7	1,7	1,9	1,9	10	10	1,9	10	10
10	1,7	1,7	1,9	1,9	1,9	1,9	1,9	1,8	1,8
10	1,7	1,7	1,9	1,9	10	10	10	10	1,8
1,5	1,5	1,5	1,9	1,9	1,9	1,9	1,9	10	10
10	1,5	1,5	10	10	10	10	10	10	10
10	1,6	1,6	1,6	1,9	10	10	10	10	1,9
10	1,5	1,5	1,9	1,9	1,9	1,9	1,9	1,5	1,5
10	1,5	1,5	1,9	1,9	1,9	1,9	1,9	1,9	1,9
10	1,9	1,9	1,9	1,9	1,9	1,9	1,9	1,9	1,9
1,8	1,6	1,6	1,9	1,9	10	1,8	1,9	10	1,9

10	1,6	1,6	1,9	1,9	10	1,8	1,9	10	1,8
1,5	1,5	1,5	1,5	1,9	1,9	1,9	1,9	1,9	1,9
10	1,5	1,5	1,5	1,5	1,5	10	10	10	10
10	1,5	1,5	1,5	1,9	10	10	10	10	1,5
10	1,9	1,9	1,9	1,9	10	1,9	1,9	10	1,9
1,5	1,5	1,5	1,5	10	10	10	10	1,9	1,5
10	1,6	1,6	1,9	1,9	10	1,8	1,9	10	1,8
1,8	1,6	1,6	1,9	1,9	10	1,8	1,9	10	1,8
1,5	1,5	1,5	1,9	1,9	1,9	1,9	1,9	1,9	1,5
10	1,6	1,6	2,5	1,6	10	10	2,9	10	2,9
10	1,8	1,8	1,9	1,9	10	10	10	10	10
10	1,5	1,5	1,9	1,9	1,9	1,9	1,9	10	1,5
1,8	1,6	1,6	1,9	1,9	10	1,9	1,9	10	1,8
10	1,6	1,6	1,9	1,9	10	1,8	1,9	10	1,8
1,5	1,6	1,6	1,9	1,9	10	1,8	1,9	10	1,8
10	1,5	1,5	1,9	1,9	10	1,8	1,9	10	10
10	1,5	1,5	1,9	1,9	10	1,8	1,9	10	10
10	1,6	1,6	1,9	1,9	10	1,8	1,9	10	1,8
10	1,9	1,9	1,9	1,9	10	1,9	1,9	10	1,9
1,5	1,5	1,5	1,5	1,9	1,9	1,9	1,9	1,9	1,9
10	1,6	1,5	1,5	1,9	10	10	10	10	1,5
10	1,7	1,7	1,9	1,9	10	10	1,9	10	1,8
10	1,6	1,6	1,9	1,9	10	1,8	1,9	10	1,8
10	1,5	1,5	1,5	1,9	1,9	1,5	1,5	10	1,5
10	1,6	1,6	1,9	1,9	10	10	1,9	10	10
10	1,7	1,7	1,9	1,9	10	1,8	1,9	10	10
1,9	1,6	1,6	1,9	1,9	10	1,9	1,9	10	1,8
10	1,6	1,6	1,9	1,9	10	1,8	1,9	10	1,8
10	1,5	1,5	1,9	1,9	10	10	1,9	10	1,8
1,5	1,6	1,6	1,9	1,9	10	1,8	1,9	10	10
10	1,5	10	1,9	1,9	10	10	10	10	2,9
10	1,7	1,7	1,9	1,9	10	1,9	1,9	10	1,9
10	1,5	1,5	10	10	10	10	10	10	10
1,5	2,6	2,6	2,6	2,9	2,9	2,9	2,9	1,9	1,9
10	2,7	2,7	2,7	1,9	10	10	10	10	10
1,8	1,5	1,5	1,5	1,9	1,9	1,9	1,9	1,9	1,9
10	1,8	1,6	1,9	2,9	10	10	2,9	10	10
1,5	1,5	1,5	1,5	1,9	1,9	1,9	1,9	1,9	1,9
1,8	1,5	1,5	1,5	1,9	1,9	1,9	1,9	10	1,9
10	1,5	1,5	1,5	1,9	1,9	1,9	1,9	10	1,9
10	2,5	2,5	2,5	1,8	10	10	10	10	1,9
1,5	1,7	1,7	1,9	1,9	1,9	1,9	1,9	1,5	1,9
10	2,6	2,6	2,6	1,8	10	10	10	10	1,5
10	1,9	1,9	1,9	1,9	1,9	10	1,9	10	1,5
10	10	1,9	1,9	2,9	1,9	1,9	1,9	10	1,9
10	1,6	1,6	1,9	1,9	1,9	10	1,9	1,5	1,9
10	1,5	1,5	1,9	1,9	10	1,9	1,9	10	10

10	1,7	1,7	1,9	1,9	10	10	10	10	1,9
10	1,6	1,6	1,9	1,9	1,9	1,9	1,9	10	1,9
1,5	1,5	1,5	1,9	10	10	1,9	1,9	1,5	1,5
10	1,6	1,6	1,9	1,9	10	10	10	10	1,9
10	1,6	1,6	1,8	1,8	10	10	10	10	10
10	1,6	1,6	1,9	1,9	10	10	10	1,9	10
1,5	1,6	1,6	1,9	1,9	10	1,9	1,9	1,8	10
10	1,7	1,7	1,9	1,9	10	1,9	1,9	10	1,9
10	1,5	1,5	1,5	1,9	1,9	1,9	1,9	1,9	1,9
10	1,9	1,9	1,9	1,9	1,9	1,9	1,9	1,9	10
10	1,9	1,9	1,9	1,9	1,9	1,9	1,9	1,9	1,9
1,5	1,9	1,9	1,9	1,9	1,9	1,9	1,9	1,9	1,9
10	1,6	1,6	1,9	10	10	10	1,9	10	1,9
10	1,5	1,5	1,9	1,9	10	10	10	10	1,6
10	1,6	1,6	1,9	10	10	10	1,9	10	1,9
10	1,6	1,9	1,9	1,9	1,9	1,9	1,9	1,9	1,9
10	1,6	1,6	1,6	1,9	10	1,9	1,9	10	1,9
10	1,5	1,5	1,9	1,9	1,9	1,9	1,9	1,9	1,9
1,5	1,5	1,5	1,5	1,9	1,9	1,9	1,9	1,9	1,9
10	1,6	1,6	1,6	1,9	10	1,9	1,9	10	1,9
10	1,7	1,7	1,9	1,9	1,9	1,9	1,9	10	1,8
10	1,5	1,5	1,9	1,9	1,9	1,9	1,9	10	1,9
10	1,9	1,9	1,9	1,9	10	1,9	1,9	10	1,9
1,8	1,6	1,6	1,9	1,9	10	1,8	1,9	10	10
1,5	1,5	1,5	1,9	1,9	1,9	1,9	1,9	10	1,9
10	1,9	1,9	1,9	1,9	1,9	1,9	1,9	10	1,9
1,7	1,7	1,7	1,7	1,7	1,7	1,7	1,7	1,6	1,6
1,9	1,9	1,9	1,9	1,9	1,9	1,9	1,9	10	1,9
10	1,9	1,9	1,9	1,9	1,9	1,9	1,9	10	1,9
1,9	1,9	1,9	1,9	1,9	1,9	1,9	1,9	10	1,5
10	1,6	1,6	1,6	1,6	1,6	1,6	1,6	1,6	1,6
1,8	1,6	1,6	1,9	1,9	10	1,8	1,9	1,8	1,8
1,5	1,5	1,5	1,5	1,9	1,9	1,9	1,9	10	1,9
1,5	1,7	1,7	1,9	1,9	10	1,9	1,9	1,8	1,5
10	1,5	1,5	1,9	1,9	1,9	1,9	1,9	1,9	1,9
10	1,7	1,7	1,9	1,9	1,9	1,9	1,9	1,8	1,8
10	1,9	1,9	1,9	1,9	1,9	1,9	1,9	10	1,9
10	1,9	1,9	1,9	1,9	1,9	1,9	1,9	10	1,9
10	1,5	1,5	1,5	1,9	1,9	1,9	1,9	10	1,9
1,5	1,5	1,5	1,5	1,5	1,5	1,5	1,5	10	1,5
10	1,5	1,5	1,5	1,9	10	1,5	1,9	10	1,8
10	1,6	1,6	1,6	1,6	1,6	1,6	1,6	1,5	1,5
10	1,5	1,5	1,5	1,9	1,9	1,9	1,9	10	1,9
10	1,9	1,9	1,9	1,9	1,9	1,9	1,9	10	1,9
10	1,5	1,5	1,5	1,8	1,8	10	10	10	10
10	1,6	1,6	1,9	1,9	1,9	1,9	1,9	1,8	1,9
1,8	1,9	1,9	1,9	1,9	10	10	1,9	1,9	1,9

10	1,5	1,5	1,9	1,9	10	10	10	10	10
10	1,9	1,9	1,9	1,9	10	10	10	1,8	1,8
10	1,5	1,5	1,5	1,9	1,8	10	10	10	1,5
10	1,5	1,5	1,5	10	10	10	10	10	1,5
10	1,5	1,5	1,5	1,8	1,8	10	10	10	1,5
10	2,6	2,6	2,9	1,9	10	10	10	10	10
10	1,6	1,5	1,9	10	10	10	10	10	10
1,5	1,6	1,6	1,6	1,9	1,9	1,9	1,9	1,9	1,9
1,5	1,5	1,5	1,5	1,9	1,9	1,9	1,9	1,9	1,9
10	1,6	1,6	10	10	10	10	10	10	1,8
1,6	1,6	1,6	1,6	1,9	1,9	1,9	1,9	10	1,8
1,6	1,6	1,6	1,6	10	10	10	10	10	1,6
10	1,7	1,7	1,9	1,9	10	1,9	1,9	1,9	1,9
1,6	1,6	1,6	1,6	1,9	1,9	1,9	1,9	10	1,9
10	1,8	1,8	1,9	1,9	10	1,8	1,9	10	10
10	1,5	1,5	1,9	1,9	1,9	1,9	1,9	10	1,9
10	1,6	1,5	10	10	10	10	10	10	10
10	1,6	1,6	1,9	1,9	1,9	1,9	1,9	10	1,9
10	2,7	2,7	2,7	1,9	10	10	1,9	10	1,9
10	2,9	2,9	2,9	2,9	2,9	2,9	10	10	10
10	1,9	1,9	10	10	10	10	10	10	10
1,8	1,5	1,5	1,5	1,9	1,9	1,9	1,9	10	1,9
10	1,5	1,5	1,9	1,9	1,9	1,9	1,9	1,9	1,9
10	2,9	2,9	1,9	1,9	2,9	1,9	2,9	10	1,9
10	1,5	1,5	1,9	1,9	10	10	1,9	10	1,9
1,6	1,5	1,5	1,5	1,9	1,9	10	1,9	10	1,9
10	1,7	1,7	1,9	1,9	1,9	1,9	1,9	10	1,9
10	2,9	2,9	2,9	1,9	10	10	1,9	10	1,9
1,5	1,5	1,5	1,5	1,9	1,9	1,9	1,9	1,9	1,9
10	1,9	1,9	2,9	1,9	10	1,9	1,9	1,9	1,9
10	1,5	1,5	1,5	10	10	10	10	10	10
10	1,6	1,6	1,9	1,9	10	1,9	1,9	10	1,9
1,8	1,9	3,9	3,9	1,9	3,9	10	1,9	10	2,9
10	1,5	1,5	1,5	1,8	1,5	10	10	10	1,8
1,8	1,9	2,9	2,9	1,9	2,9	10	1,9	10	2,9
1,5	1,5	1,5	1,5	1,8	1,8	1,8	1,8	1,8	1,9
1,8	1,5	1,5	1,8	1,8	1,8	1,8	1,8	1,9	1,9
10	1,6	1,6	1,9	1,9	1,9	1,9	1,9	10	1,9
10	1,6	1,7	1,9	1,9	10	10	10	10	1,5
10	1,5	1,5	1,5	1,9	1,9	1,9	1,9	1,9	1,9
1,8	1,7	1,7	1,9	1,9	10	1,9	1,9	1,8	1,9
10	1,7	1,7	1,9	1,9	10	1,9	1,9	1,8	1,9
10	1,5	1,5	10	10	10	10	10	10	10
10	1,5	1,5	1,5	1,8	1,8	1,8	1,8	10	10
10	1,7	1,7	1,9	1,9	10	10	10	10	10
10	1,5	1,5	1,9	1,9	10	10	1,9	1,9	1,9
10	1,7	1,7	1,7	1,9	10	1,9	1,9	1,8	1,9

10	1,5	1,5	1,5	1,9	10	10	10	10	10
10	1,9	1,9	1,9	1,9	10	10	10	10	2,9
10	2,7	2,7	2,7	1,9	10	10	10	10	10
10	1,9	1,9	1,9	1,9	10	10	1,9	10	1,9
10	1,7	10	10	10	10	10	10	1,9	10
10	1,7	1,7	1,9	1,9	10	1,9	1,9	10	10
10	1,9	1,9	1,9	1,9	10	1,9	10	10	10
10	1,5	1,5	1,9	1,9	1,9	1,9	1,9	10	10
10	1,7	1,7	1,7	10	10	10	10	10	10
1,5	1,5	1,5	1,5	1,9	1,9	1,9	1,9	1,9	1,9
10	1,9	2,9	2,9	1,9	1,9	1,9	1,9	10	1,9
1,5	1,5	1,5	1,5	1,9	1,9	1,9	1,9	1,5	1,9
10	2,7	2,7	2,9	1,9	10	1,5	1,9	1,5	1,9
10	2,6	2,6	2,6	1,9	10	1,5	10	10	1,9
10	1,7	1,7	1,9	1,9	10	1,9	1,9	10	1,9
10	1,5	1,5	1,5	1,8	10	10	10	10	10
10	1,7	1,7	1,9	1,9	1,9	10	1,9	10	1,9
10	1,7	1,7	1,9	1,9	1,9	10	1,9	10	1,9
10	1,6	1,6	1,9	1,9	10	10	1,9	10	1,9
10	1,6	1,6	1,9	1,9	10	1,9	1,9	10	1,9
10	2,9	2,9	1,9	1,9	10	1,9	1,9	10	1,5
10	1,5	1,5	1,5	1,8	10	10	10	10	10
10	1,5	1,5	1,5	1,9	10	10	10	10	10
10	1,8	1,8	1,8	1,9	10	1,9	1,9	10	1,9
10	1,5	1,5	1,5	1,9	1,9	1,9	1,9	10	10
10	1,5	1,5	1,9	10	1,9	1,9	1,9	10	1,9
10	1,5	1,5	1,5	1,9	1,8	1,8	1,8	10	10
10	1,5	1,5	1,5	1,9	10	1,5	1,9	10	1,5
10	1,9	1,9	1,9	1,9	1,9	1,9	1,9	1,9	1,9
1,5	1,5	1,5	1,5	1,9	1,9	1,9	1,9	1,9	1,9
10	1,6	1,6	1,9	1,9	1,9	1,8	1,9	1,8	1,8
10	1,5	1,5	1,9	1,9	1,9	1,9	1,9	1,5	1,5
10	1,5	1,5	1,8	1,9	1,9	1,9	1,9	10	1,9
10	1,5	1,5	1,9	1,9	1,5	1,9	1,9	1,5	1,5
10	1,6	1,6	1,6	1,6	1,6	1,5	1,9	10	1,6
1,8	1,7	1,7	1,9	1,9	10	10	1,9	10	10
10	1,5	1,5	1,5	1,9	10	1,5	1,9	1,8	1,5
10	1,6	1,6	1,6	1,6	1,6	1,6	1,6	10	1,6
10	1,9	1,9	1,9	1,9	1,9	1,9	1,9	10	1,9
10	1,6	1,6	1,9	1,9	1,9	1,8	1,9	10	1,8
10	1,5	1,5	1,9	1,9	1,9	1,9	1,9	10	10
10	1,5	1,5	1,9	1,9	10	1,9	1,9	10	10
10	1,5	1,5	1,9	1,9	10	1,9	1,9	10	10
1,5	1,5	1,5	1,5	1,9	1,9	1,9	1,9	1,9	1,9
10	1,6	1,5	1,5	1,9	1,9	1,9	1,9	10	1,9
10	1,7	1,7	1,9	1,9	10	1,9	1,9	10	1,8

grapes	guava	mango	naartjie	peach	pear	pineapple	apricots	watermelc	madoro
1,6	2,7	1,9	1,5	10	1,5	1,5	10	1,7	10
1,6	2,7	1,9	1,5	10	1,5	1,5	10	1,7	10
1,6	1,7	1,5	1,7	1,5	1,6	1,5	1,6	1,5	10
1,8	2,9	2,9	2,9	2,9	1,8	10	10	1,9	10
1,5	1,5	1,5	1,5	1,5	1,5	10	10	2,5	10
1,5	1,5	2,9	1,9	10	10	10	10	1,9	10
10	1,5	1,9	1,9	10	10	10	10	1,9	10
1,8	3,9	1,9	1,9	1,9	1,5	1,5	10	1,9	10
1,8	1,9	1,9	1,9	1,8	1,8	1,8	1,8	1,9	10
1,8	10	1,9	1,9	10	10	10	10	1,8	10
1,6	1,7	1,9	10	10	2,5	2,5	10	1,6	10
1,5	1,5	1,5	1,5	1,5	1,5	1,5	1,5	1,5	10
2,5	2,9	3,9	2,9	1,5	1,5	1,5	1,5	2,9	10
1,8	1,9	1,9	10	10	10	1,5	10	1,9	10
1,8	1,9	1,9	1,9	1,9	1,8	10	1,9	1,9	10
1,5	1,9	1,9	10	1,6	1,5	10	10	1,9	10
1,5	2,9	2,9	10	1,5	1,5	1,5	10	2,9	10
1,5	1,5	2,7	2,6	1,5	1,5	10	10	2,6	10
1,5	1,5	3,9	1,5	1,5	1,5	1,8	1,8	2,7	10
1,5	1,5	2,7	2,5	1,5	1,5	10	10	2,6	10
1,5	1,5	2,6	1,6	2,6	10	10	10	2,6	10
1,5	1,5	2,6	2,6	1,5	1,5	1,5	1,5	2,6	10
1,5	2,9	2,5	2,5	1,5	1,5	1,5	1,5	3,7	10
1,5	1,9	3,9	2,9	1,5	1,5	1,8	1,5	2,6	10
2,5	1,9	3,9	2,9	1,5	2,5	1,8	1,5	2,9	10
1,5	10	10	2,6	10	1,6	10	1,5	2,6	10
2,5	2,9	3,9	10	2,9	10	10	10	2,6	10
1,9	1,9	1,9	1,9	1,9	1,8	10	10	1,9	10
1,5	10	10	10	10	1,5	1,5	1,5	1,5	10
10	1,8	2,9	10	10	1,7	10	2,9	1,7	10
10	10	1,9	10	10	10	10	10	1,6	10
10	2,9	2,9	1,6	1,5	1,5	1,5	10	2,6	10
1,8	1,8	1,8	1,8	1,8	1,8	1,8	1,8	2,9	10
10	1,9	1,9	10	10	10	10	10	1,9	10
1,9	1,9	1,9	1,9	10	10	10	10	1,9	10
10	2,9	2,9	10	2,9	10	1,5	10	2,9	10
10	10	3,9	10	10	1,5	10	10	1,9	10
10	10	2,9	10	10	10	10	10	2,9	10
1,9	1,9	1,9	1,9	1,9	10	10	10	1,9	10
10	10	1,9	10	10	10	10	10	10	10
1,6	1,6	1,6	1,6	10	10	10	1,6	1,5	10
1,5	1,5	10	10	10	1,5	1,5	1,5	1,9	10
10	1,9	1,9	1,9	10	10	10	10	1,9	10
1,6	1,6	1,6	1,6	10	1,6	10	10	1,6	10
1,8	10	1,9	1,9	1,9	10	10	10	1,9	10
10	1,9	2,9	10	10	10	10	10	1,9	10

10	1,9	1,9	10	10	10	10	10	1,9	10
10	1,9	2,9	1,9	1,9	1,8	1,8	10	3,9	10
1,5	1,5	1,5	1,5	1,5	1,5	10	10	2,6	10
1,5	1,7	1,7	1,7	1,5	1,5	1,5	10	1,7	1,5
1,5	1,5	1,9	10	10	1,8	10	1,7	1,9	10
1,9	1,9	1,9	1,9	10	10	10	10	2,8	10
1,5	10	1,9	1,9	1,9	10	10	1,9	1,9	10
1,8	10	2,9	2,9	10	1,7	1,7	10	1,9	10
10	10	2,9	2,9	10	10	10	10	2,8	10
1,8	1,9	1,9	1,9	10	2,7	10	10	2,9	10
10	10	1,9	1,9	10	10	10	10	10	10
10	1,9	1,9	1,9	10	10	10	10	2,9	10
10	10	1,9	1,9	10	10	10	10	1,5	10
1,6	1,5	1,5	1,5	1,5	1,6	1,5	1,5	1,5	10
1,8	2,9	3,9	2,9	10	1,8	10	10	1,9	10
1,9	1,9	1,9	1,9	10	10	10	10	1,9	10
1,5	1,9	1,9	1,9	1,9	1,6	10	10	2,9	10
2,8	1,8	3,9	2,9	10	10	10	10	2,8	10
10	10	1,9	10	10	10	10	10	1,9	10
10	10	1,9	1,5	10	10	10	10	1,7	10
2,5	1,5	2,6	1,5	1,5	1,5	1,5	1,5	1,5	10
1,9	1,9	2,9	2,9	1,5	1,5	1,5	1,5	2,5	10
1,9	1,5	2,9	2,9	1,5	1,5	1,5	1,5	2,9	10
10	1,5	1,9	1,9	10	10	10	10	1,9	10
10	1,5	1,9	1,9	10	10	10	10	1,9	10
2,8	20	2,9	2,8	10	10	10	10	2,9	10
10	10	1,9	1,9	10	10	10	10	1,7	10
10	1,9	1,9	10	10	10	10	10	1,9	10
1,5	2,6	10	1,6	10	1,5	10	10	2,6	10
10	10	1,9	10	10	10	10	10	2,6	10
10	1,8	1,8	10	10	10	10	10	1,9	10
10	1,9	1,9	10	10	10	10	10	1,9	10
10	10	1,9	10	10	10	10	10	1,5	10
10	1,5	3,9	1,9	1,8	1,8	1,8	2,9	1,5	10
1,5	10	1,9	1,9	1,5	1,5	10	10	1,6	10
1,5	2,6	2,6	2,6	1,5	1,5	1,5	1,5	2,6	10
2,9	2,9	2,9	2,6	1,5	1,5	1,5	1,5	2,6	10
10	1,9	1,9	1,9	10	10	10	10	1,9	10
10	10	10	10	10	10	10	10	2,9	10
1,5	1,5	1,6	1,5	1,5	1,5	1,5	10	1,6	10
1,9	1,9	1,9	1,9	1,9	1,8	10	10	1,9	10
1,8	1,9	1,9	1,9	1,8	1,8	1,8	10	1,9	10
1,8	1,9	1,9	1,9	1,8	1,8	10	10	1,9	10
1,8	1,9	1,9	1,9	1,9	1,5	1,8	10	1,9	10
1,8	1,9	1,9	1,9	1,8	1,8	10	10	1,9	10
1,8	1,9	1,9	1,9	1,8	1,5	10	10	1,9	10
1,5	1,9	1,9	1,5	1,5	1,5	10	1,5	1,9	1,5

1,9	1,9	1,9	1,9	1,9	10	10	10	10	10
1,5	10	10	10	10	10	10	10	1,9	10
1,7	1,6	1,9	1,9	1,8	1,7	1,7	10	1,8	10
1,8	1,9	2,9	1,9	1,9	10	10	10	10	10
10	1,5	1,9	1,9	1,9	10	10	10	1,9	1,9
10	3,7	1,9	1,9	2,6	10	10	10	2,5	10
1,5	1,5	1,6	1,5	1,5	1,5	1,5	1,5	1,6	10
1,9	1,9	1,9	1,9	1,9	10	10	10	1,9	10
1,9	1,8	1,9	1,8	1,8	1,5	10	10	1,9	10
1,8	1,9	1,9	2,9	2,9	2,9	1,8	10	3,9	10
1,8	1,9	1,9	1,9	10	10	10	10	1,9	10
10	1,9	1,9	1,9	1,9	10	10	10	1,9	10
1,7	1,7	1,9	1,7	10	1,5	10	10	1,9	10
1,5	2,7	3,6	3,7	10	10	10	10	1,5	10
1,8	1,9	1,9	1,9	10	10	10	10	1,9	10
1,5	1,9	1,9	1,5	1,5	1,5	1,5	1,5	2,9	10
1,9	10	1,9	10	10	10	10	10	2,9	10
10	1,6	1,9	1,9	10	10	10	10	1,6	10
1,8	3,6	2,9	1,5	3,6	10	10	10	2,5	10
1,9	1,9	1,9	1,9	1,9	10	1,9	10	1,9	10
1,5	1,5	1,6	1,5	1,5	1,5	1,5	1,5	1,9	10
1,8	1,9	1,9	1,9	1,9	1,8	1,8	1,8	1,9	10
1,5	1,7	1,5	1,5	1,5	1,5	10	1,5	1,5	10
1,5	1,7	1,5	1,5	1,5	1,5	10	1,5	1,5	10
1,5	1,7	1,5	1,5	1,5	1,5	10	1,5	1,5	10
1,9	1,9	1,9	2,9	2,9	10	2,9	10	2,9	10
1,9	1,9	2,9	1,9	2,9	10	10	10	2,9	10
10	1,5	1,9	1,9	1,9	10	10	10	1,5	10
10	1,9	1,9	1,9	1,9	10	10	10	1,9	10
1,5	1,9	1,9	1,5	1,5	1,5	1,5	10	1,9	10
1,5	2,9	2,9	2,8	1,8	10	10	10	2,8	10
2,5	3,6	1,5	1,5	1,5	1,5	1,5	1,5	1,6	10
1,5	1,9	1,9	1,5	1,5	1,5	1,5	1,5	1,9	10
1,5	1,5	2,9	10	10	10	1,8	10	1,7	10
1,8	1,9	1,9	10	10	10	10	10	1,9	10
1,6	1,6	10	10	10	1,5	1,5	10	10	10
1,7	1,7	1,7	10	10	1,6	1,6	1,6	1,9	10
1,9	1,9	1,9	1,9	1,9	10	10	10	1,9	10
1,8	1,9	1,9	10	10	1,8	10	10	1,9	10
1,8	10	2,9	10	10	10	10	10	1,9	1,9
1,5	1,9	1,9	1,9	1,9	1,5	1,9	10	1,9	10
1,5	10	10	1,5	1,5	1,5	10	10	1,5	10
1,5	1,9	1,9	1,9	1,9	1,5	10	10	1,8	10
1,9	1,9	1,9	1,5	10	10	10	10	1,5	10
1,9	2,9	3,9	2,9	1,5	1,5	1,5	1,5	3,9	10
1,9	1,9	1,9	1,9	1,9	1,9	1,9	1,9	1,9	10
1,6	2,5	2,9	1,5	1,5	1,6	1,5	1,5	2,9	10

1,9	1,9	1,9	1,9	1,9	10	10	10	1,9	10
1,9	1,9	1,9	1,9	1,9	1,9	1,8	1,8	3,9	10
1,8	1,9	1,9	1,9	1,8	1,8	10	10	1,9	10
1,8	1,9	1,9	1,9	1,8	1,8	10	10	1,8	10
1,8	2,9	2,9	2,9	2,8	10	10	10	2,8	10
1,8	1,9	1,5	1,5	1,8	1,8	1,8	1,8	2,5	10
1,5	2,9	4,9	1,5	1,5	1,5	1,5	1,5	2,9	1,5
1,8	1,9	1,5	1,9	1,9	10	10	10	1,9	10
1,5	1,9	1,9	1,9	10	1,5	10	1,5	3,9	10
10	1,6	1,9	1,8	10	1,5	10	1,5	1,5	10
1,8	1,9	1,9	1,9	1,9	10	10	10	1,9	10
1,8	1,9	1,9	1,9	1,9	10	10	10	1,9	10
1,9	1,9	1,9	1,9	10	10	10	10	1,5	10
1,8	3,6	1,9	2,7	1,9	1,9	1,5	1,9	1,5	10
1,9	1,9	1,9	1,5	10	10	10	10	1,5	10
1,5	1,5	1,5	1,5	10	10	10	10	1,6	10
1,5	1,6	1,9	1,9	10	10	10	10	1,5	10
10	1,6	1,9	1,9	10	1,5	10	10	1,5	10
10	1,5	2,6	2,6	10	10	10	10	10	10
1,6	1,9	1,9	10	10	1,5	10	10	1,5	10
10	2,9	1,9	1,9	1,9	10	10	10	3,9	10
1,8	1,9	1,9	1,9	10	10	10	10	1,9	10
1,8	1,9	1,9	1,9	10	10	10	10	1,9	10
1,8	1,9	1,9	1,9	1,8	1,7	1,8	10	1,9	10
1,8	1,9	1,9	1,9	1,8	1,7	1,8	10	1,9	10
1,5	1,6	1,9	1,5	10	10	10	10	1,5	10
1,8	1,8	1,8	1,8	1,8	1,8	10	10	1,6	10
1,8	1,9	1,9	1,8	10	1,8	10	10	1,8	10
1,5	1,9	1,9	1,9	1,9	10	10	10	10	10
1,5	1,6	3,6	1,5	1,5	1,5	1,5	1,5	2,6	10
10	1,9	1,9	1,9	1,9	10	10	3,9	10	10
1,9	1,9	1,9	1,9	1,9	10	10	10	1,9	10
1,9	10	1,9	10	10	10	10	10	1,9	10
1,5	1,5	3,7	10	1,5	1,5	1,5	1,5	2,6	10
1,5	1,9	1,5	1,6	1,5	1,5	1,5	1,5	1,5	10
1,9	1,9	1,9	1,9	1,9	10	1,9	1,9	1,9	10
10	1,9	1,9	1,9	1,9	10	10	10	1,9	10
10	1,9	1,9	1,9	1,9	10	10	10	1,9	10
1,9	2,5	1,9	1,5	10	10	10	10	1,9	10
1,9	2,5	1,9	1,5	10	10	10	10	1,9	10
10	1,8	1,9	1,9	1,9	10	10	10	10	10
1,8	1,9	1,9	1,9	1,8	1,8	10	10	1,9	10
1,8	1,9	1,9	1,9	1,9	1,5	1,5	10	1,5	10
1,5	1,5	1,9	1,5	1,9	1,5	1,5	10	1,6	1,9
1,5	1,9	1,9	1,9	1,9	1,8	1,8	1,8	1,5	10
1,8	10	2,9	10	10	1,8	10	10	1,9	10
1,8	1,9	1,9	1,9	1,9	1,5	10	10	1,5	1,5

10	1,9	1,9	10	10	10	10	10	2,9	10
1,8	1,9	1,9	1,9	1,9	10	10	10	10	10
10	1,9	1,9	1,9	1,9	10	10	10	1,9	10
2,5	1,5	2,7	1,5	1,5	1,5	1,5	1,5	2,6	10
2,5	1,5	2,7	1,5	1,5	1,5	1,5	1,5	2,6	10
1,9	1,9	1,9	1,9	1,9	10	10	10	1,9	10
10	1,9	1,9	1,9	1,9	10	10	10	1,9	10
10	1,9	1,9	1,9	10	10	10	10	3,9	10
10	1,9	10	10	10	10	10	10	10	10
1,8	1,9	1,9	1,9	1,9	10	10	10	2,8	10
10	1,5	1,5	1,5	10	10	10	1,5	1,5	10
1,8	1,9	1,9	1,9	1,8	1,5	10	10	1,9	10
10	1,9	1,9	10	1,9	10	10	10	1,9	10
1,8	1,9	1,9	1,9	1,9	10	10	10	1,9	10
1,9	1,9	1,9	1,9	1,9	1,5	1,5	1,5	1,9	10
10	1,9	1,9	1,9	1,9	10	10	10	1,9	10
1,9	1,9	1,9	1,9	1,9	1,5	1,5	1,5	1,9	10
10	1,9	1,9	1,9	1,9	10	10	10	1,9	10
1,9	1,9	1,9	1,9	1,9	10	10	10	1,9	10
1,8	1,9	2,9	2,9	2,9	2,7	1,8	10	1,5	10
1,9	1,9	1,9	10	10	10	10	10	1,9	10
1,5	10	1,5	10	10	10	1,5	1,5	10	10
1,5	10	1,5	10	10	10	1,5	1,5	10	10
10	10	10	10	10	10	10	10	1,9	10
10	1,9	1,9	1,9	1,8	10	10	10	1,9	10
1,9	1,6	1,9	1,6	1,9	1,6	1,5	1,5	1,6	10
1,5	2,5	2,6	1,5	1,5	1,5	10	10	2,6	10
10	1,9	1,9	1,9	1,9	10	10	10	1,9	10
10	1,9	1,9	1,9	1,9	1,8	1,9	10	1,9	10
1,8	1,9	2,9	2,9	10	10	10	10	1,5	10
10	1,9	1,9	1,9	1,8	1,8	10	10	1,9	10
1,9	1,9	1,9	10	10	10	10	10	1,9	10
1,9	1,5	1,9	1,5	1,9	1,5	1,9	1,5	1,5	10
1,8	1,9	1,9	1,9	1,8	1,8	10	10	1,9	10
10	1,9	1,9	1,9	1,9	10	10	10	1,9	10
1,9	1,9	1,9	1,9	10	10	10	10	1,9	10
10	10	1,9	1,5	10	10	1,5	10	1,5	10
1,8	1,9	1,9	1,9	1,9	10	10	10	1,8	10
10	1,9	1,9	1,9	1,9	10	10	10	1,9	10
1,5	1,5	1,9	1,5	1,5	1,5	1,5	1,5	1,5	10
1,8	1,9	1,9	1,9	1,8	1,8	1,8	1,8	1,9	10
1,8	1,9	1,9	10	10	10	10	10	1,9	10
1,8	1,9	1,9	1,9	1,9	10	10	10	10	10
10	1,9	2,9	2,9	10	10	10	10	1,9	10
1,8	1,9	1,9	1,9	1,9	10	10	10	1,8	10
1,8	1,9	1,9	10	10	10	10	10	1,9	10
1,5	1,5	2,9	1,5	1,5	1,5	10	10	1,5	10

1,5	1,5	2,6	1,5	1,5	1,5	10	10	2,5	10
1,9	1,9	1,9	1,9	1,9	10	10	10	1,9	10
1,9	1,9	1,9	1,9	1,8	1,8	10	10	1,9	10
10	1,9	1,9	1,9	1,9	10	10	10	1,9	10
1,9	1,9	1,9	1,9	1,9	10	10	10	1,9	10
10	1,9	1,9	1,9	1,9	10	10	10	10	10
10	1,9	1,9	1,9	10	10	10	10	1,9	10
1,9	1,9	1,9	1,9	1,9	10	10	10	1,9	10
1,9	1,9	1,9	1,9	10	10	10	10	1,9	10
1,9	1,9	1,9	1,9	1,9	10	10	10	1,9	10
1,5	1,9	2,9	2,6	10	1,5	10	10	1,5	10
1,9	1,7	1,9	1,9	1,9	1,8	10	10	1,9	10
1,8	1,8	1,9	1,9	1,9	10	10	10	10	10
1,8	1,8	1,9	1,9	1,9	10	10	10	10	10
1,8	1,9	2,9	2,9	3,9	1,5	10	10	1,9	10
1,7	1,7	3,9	10	10	10	10	10	10	10
1,9	2,9	4,9	2,9	1,5	1,5	1,5	1,5	2,6	10
1,9	2,9	4,9	2,9	1,5	1,5	1,5	1,5	2,6	10
2,9	2,9	2,9	2,9	2,9	10	10	10	1,9	10
10	2,9	2,9	2,9	2,9	10	10	10	1,5	10
10	2,9	3,9	2,9	3,9	10	10	10	1,9	10
1,8	2,9	3,9	2,9	2,9	1,8	10	10	10	10
10	3,9	3,9	2,9	10	10	10	10	1,8	10
1,8	2,9	3,9	2,9	1,9	1,8	10	10	1,9	10
1,8	2,9	3,9	2,9	10	10	10	10	1,9	10
1,9	1,9	1,9	1,9	1,9	1,9	10	1,5	1,9	10
1,8	1,9	2,9	2,9	3,9	1,8	10	10	1,9	10
10	1,9	1,9	1,9	1,9	1,6	10	10	1,9	10
1,6	1,8	1,8	2,8	2,8	1,7	1,7	10	2,9	10
1,8	2,9	2,9	2,9	3,8	1,8	10	10	1,9	10
1,8	2,9	2,9	2,9	2,9	2,5	10	10	1,9	10
10	3,9	2,9	2,9	3,9	10	10	10	1,9	10
1,9	1,9	1,9	1,9	1,9	10	1,9	1,9	1,9	10
1,9	1,9	1,9	1,9	1,9	1,5	1,9	10	1,9	1,9
1,8	2,8	3,7	2,6	1,5	1,5	1,5	1,5	1,5	10
2,9	2,9	2,9	10	10	10	10	10	1,9	10
1,8	2,5	3,9	2,9	2,9	10	10	10	1,9	10
1,9	1,9	1,9	1,9	1,9	10	10	10	1,9	10
1,9	1,9	1,9	1,9	1,9	1,9	10	10	1,9	10
1,8	1,9	1,9	1,9	1,8	10	10	10	1,9	10
1,8	2,9	2,9	3,9	3,9	1,8	1,8	10	1,9	10
1,8	1,9	3,9	3,9	3,9	1,8	1,8	10	1,9	10
1,8	1,9	1,9	1,9	1,9	1,8	10	10	1,9	10
1,8	1,9	1,9	1,9	1,9	1,8	10	10	1,9	10
1,9	1,9	1,9	1,9	1,9	1,9	10	10	1,9	10
1,9	1,9	1,9	1,9	1,9	1,9	1,9	10	1,9	10
10	10	1,5	10	10	10	10	10	1,5	10

1,9	1,9	1,9	1,9	1,9	1,9	1,9	1,9	1,9	10
10	10	1,9	10	10	10	10	10	1,5	10
1,5	1,5	1,5	1,5	10	10	10	10	10	10
1,9	1,9	1,9	1,9	1,9	1,8	1,9	1,9	1,9	10
1,5	1,5	1,5	1,5	1,5	1,5	10	10	1,9	10
1,9	1,6	3,9	10	10	10	1,8	1,8	1,9	10
10	1,5	1,9	1,9	10	10	10	10	10	10
1,5	1,5	1,6	10	10	10	10	10	1,6	10
1,4	10	1,5	10	10	10	10	10	1,6	10
1,4	10	1,5	10	10	10	10	10	1,6	10
1,5	10	1,7	10	10	10	10	10	10	10
1,5	1,5	1,9	1,9	10	10	10	10	10	10
1,9	3,6	1,9	1,9	10	10	1,9	1,9	2,5	10
1,5	1,5	1,5	10	10	10	1,6	10	1,5	10
1,8	1,9	1,9	1,9	1,9	1,8	1,8	10	1,9	10
1,9	1,9	1,9	1,9	1,9	1,9	1,9	1,9	1,9	10
1,8	1,9	1,9	1,9	1,9	1,8	10	10	1,9	10
1,6	1,9	1,6	10	10	10	10	10	10	10
1,5	1,5	1,5	1,5	1,5	1,5	1,6	1,6	1,6	10
1,9	1,9	1,9	1,9	1,9	1,9	1,9	1,9	1,9	10
1,8	1,9	1,5	10	10	10	10	10	10	10
10	10	1,9	1,9	10	1,8	10	10	1,9	10
1,8	1,9	1,9	1,9	10	10	1,8	10	1,9	10
1,8	1,9	1,9	1,9	1,9	10	10	10	1,9	10
1,9	1,9	1,9	1,9	1,9	1,9	1,9	1,9	1,9	10
1,9	1,9	1,9	1,9	1,9	1,9	1,9	1,9	1,9	10
1,8	10	1,9	1,9	10	1,8	10	10	1,9	10
1,9	1,9	1,9	1,9	1,9	1,9	10	10	1,5	10
1,8	1,9	1,9	1,9	1,9	1,8	10	10	1,9	10
1,8	1,8	1,8	1,8	1,8	1,8	10	10	1,9	10
1,5	1,9	1,9	10	10	10	10	10	1,9	10
10	10	1,9	10	10	1,5	1,9	1,9	1,9	10
10	10	10	10	10	10	10	10	1,5	10
1,9	1,9	1,9	1,9	1,9	1,9	1,9	1,9	1,9	10
10	10	1,9	1,9	10	10	10	10	1,9	10
1,9	1,9	1,9	10	10	10	1,9	1,9	1,9	1,9
1,9	1,9	1,9	1,9	1,9	1,8	10	1,9	1,9	10
10	10	1,9	1,9	10	10	10	10	1,9	10
1,8	1,9	1,9	1,9	1,9	1,8	1,9	10	1,9	10
1,8	1,9	1,9	1,9	10	1,8	10	10	1,9	10
1,8	10	1,9	10	10	10	10	10	1,9	10
1,9	1,9	10	10	10	10	10	10	1,9	10
10	10	1,9	1,9	10	10	10	10	1,9	10
1,5	1,9	1,9	1,5	1,5	1,5	1,5	10	1,5	10
1,9	1,9	1,9	1,9	1,9	1,9	1,9	1,9	1,9	1,9
1,9	1,9	1,9	1,9	1,9	1,9	1,9	10	1,9	10
1,9	1,9	1,9	1,9	1,9	1,8	10	10	1,9	10

1,8	1,9	1,9	1,9	1,9	1,8	10	10	1,9	10
1,9	1,9	1,9	1,9	1,9	1,9	1,9	1,9	1,9	10
10	10	10	10	10	10	10	10	1,8	10
10	10	1,9	10	10	10	10	10	1,9	10
10	1,9	1,9	1,9	1,9	1,9	10	10	1,9	10
1,9	1,9	1,9	10	10	10	1,9	10	1,9	10
1,8	1,9	1,9	1,9	1,9	1,8	10	10	1,9	10
1,8	1,9	1,9	1,9	1,9	1,8	10	10	1,9	10
10	10	1,9	10	10	10	1,9	1,9	1,9	10
10	10	1,9	10	10	10	10	10	1,9	10
10	10	1,9	1,9	10	10	10	10	1,9	10
1,9	1,9	1,9	1,9	10	10	1,8	10	1,8	10
1,8	1,9	1,9	1,9	1,9	1,5	10	10	1,9	10
1,8	10	10	10	10	1,8	10	10	1,9	10
1,8	1,9	1,9	1,9	1,9	1,8	10	10	1,9	10
10	1,9	1,9	1,9	1,9	10	10	10	1,9	10
10	1,9	1,9	1,9	1,9	10	10	10	1,9	10
1,8	1,9	1,9	1,9	1,9	1,8	10	10	1,9	10
10	1,9	1,9	1,9	1,9	1,9	10	10	1,9	10
1,9	1,9	1,9	1,9	1,9	1,9	1,8	1,8	1,8	10
10	10	1,9	10	10	10	10	10	1,6	10
1,8	1,9	1,9	1,9	10	10	10	10	1,9	10
1,8	1,9	1,9	1,9	1,9	1,8	10	10	1,9	10
1,5	1,5	1,9	1,9	1,9	1,9	1,9	1,5	1,8	10
1,8	1,9	1,9	1,9	1,9	1,8	10	10	1,9	10
1,8	1,9	1,9	1,9	10	10	10	10	1,9	10
1,8	1,9	1,9	1,9	1,9	1,5	10	10	1,9	10
1,8	1,9	1,9	1,9	1,9	1,8	10	10	1,9	10
1,8	1,9	1,9	1,9	1,9	1,8	10	10	1,9	10
1,8	1,9	1,9	1,9	1,9	1,8	10	10	1,9	10
10	10	10	10	10	10	10	10	1,9	10
1,8	1,9	1,9	1,9	1,9	1,8	10	10	1,9	1,9
10	10	10	10	10	10	10	10	1,5	10
1,9	1,9	1,9	1,9	1,9	1,9	1,9	1,9	1,9	10
10	2,9	2,9	2,9	10	10	10	10	10	10
1,9	1,9	1,9	1,9	1,9	1,9	1,9	1,9	1,5	10
10	2,9	3,9	2,9	2,9	10	10	10	10	10
1,9	1,9	1,9	1,9	1,9	1,9	10	10	1,5	10
1,9	1,9	1,9	1,9	1,9	1,9	1,9	1,9	1,9	10
1,9	1,9	1,9	1,9	1,9	1,9	1,9	1,9	1,9	10
10	10	2,9	10	10	10	10	10	2,8	10
1,9	1,9	1,9	1,9	1,9	1,8	10	10	1,9	10
10	10	2,9	10	10	10	1,6	10	2,8	10
10	10	1,9	10	10	10	10	10	1,9	1,9
10	10	10	10	10	10	10	10	10	10
1,9	1,9	1,9	1,9	1,9	1,9	1,9	10	10	10
1,5	10	1,9	10	10	1,5	10	10	1,8	10

1,8	1,9	1,9	1,9	1,9	1,8	10	10	1,9	10
10	10	10	10	10	10	1,9	10	10	10
1,9	1,9	10	10	10	10	1,9	10	1,9	10
1,8	1,9	1,9	1,9	10	10	10	10	1,9	10
1,8	1,9	1,9	1,9	10	10	10	10	1,9	10
1,8	1,9	1,9	1,9	10	10	10	10	1,9	10
1,9	1,9	1,9	1,9	1,9	1,8	10	10	1,9	10
1,9	1,9	1,9	1,9	1,9	1,8	10	10	1,9	10
1,9	1,9	1,9	1,9	1,9	1,9	10	10	10	10
1,9	1,9	1,9	1,9	1,9	1,9	1,9	1,9	1,9	10
1,9	1,9	1,9	1,9	1,9	1,9	1,9	1,9	1,9	10
1,9	1,9	1,9	1,9	1,9	1,9	1,5	1,5	1,5	10
1,9	10	1,9	10	10	1,5	10	10	1,9	10
10	10	1,9	10	10	10	10	10	10	10
1,9	10	1,9	1,9	1,9	1,5	10	10	1,9	10
1,9	1,9	1,9	10	10	1,8	1,6	10	1,6	10
1,9	1,9	1,9	1,9	1,9	1,9	1,9	1,9	1,9	10
1,9	1,9	1,9	10	10	10	10	10	10	10
1,9	1,9	1,9	1,9	1,9	1,9	1,9	1,9	1,9	10
1,9	1,9	1,9	1,9	1,9	1,9	1,9	1,9	1,9	10
1,9	1,9	1,9	1,9	1,9	1,8	1,8	10	1,8	1,9
1,9	1,9	1,9	1,9	10	10	10	10	10	10
1,9	1,9	1,9	1,9	1,9	1,9	10	10	1,9	10
1,8	1,9	1,9	1,9	1,9	1,8	10	1,9	10	10
1,9	1,9	1,9	1,9	1,9	1,5	1,5	1,5	1,5	10
1,9	1,9	1,9	1,9	1,9	1,9	10	10	1,9	10
1,6	1,6	1,6	1,6	1,6	1,6	1,6	1,5	1,6	10
1,9	1,9	1,9	1,9	1,9	1,9	1,9	10	1,9	10
1,9	1,9	1,9	1,9	1,9	1,9	10	10	1,9	10
1,5	1,5	1,5	1,5	1,5	1,5	10	10	1,9	10
1,6	1,6	1,6	1,6	1,6	1,6	1,5	1,5	1,5	10
1,8	1,9	1,9	1,9	1,9	1,8	1,8	1,8	1,9	10
1,9	1,9	1,9	1,9	1,9	1,9	1,5	1,5	1,5	1,9
1,9	1,9	1,9	1,9	1,9	1,8	1,8	10	1,9	10
1,9	1,9	1,9	1,9	1,9	1,9	1,5	1,5	1,9	10
1,9	1,9	1,9	1,9	10	1,8	1,8	10	10	10
1,9	1,9	1,9	1,9	1,9	1,9	1,9	1,9	1,9	10
1,9	1,9	1,9	1,9	1,9	1,9	1,9	1,9	1,9	10
1,9	1,9	1,9	1,9	1,9	1,9	1,9	1,9	1,9	10
1,5	1,5	1,5	1,5	1,5	1,5	10	10	10	10
1,9	1,8	1,9	1,9	1,9	1,9	1,6	1,6	1,9	10
1,5	1,6	1,6	1,5	1,5	1,5	1,5	1,6	1,6	1,6
1,9	1,9	1,9	1,9	1,9	1,9	1,9	1,9	1,9	10
1,9	10	1,9	1,9	1,9	10	10	10	1,9	10
1,5	1,5	2,5	1,5	10	10	1,9	10	10	10
1,9	1,9	1,9	1,9	1,9	1,8	1,8	1,9	1,9	10
1,9	1,9	1,9	1,9	1,9	1,9	10	1,9	1,9	10

10	10	10	10	10	10	10	10	1,5	10
1,8	1,8	1,8	10	10	10	10	10	1,5	10
1,5	1,9	1,9	1,5	10	10	1,9	1,9	1,9	10
1,5	1,5	1,9	10	10	10	10	10	10	10
1,5	1,5	1,9	1,9	10	10	10	10	10	10
10	10	1,9	1,9	10	10	10	10	1,9	10
10	10	1,9	10	10	10	10	10	10	10
1,9	1,9	1,9	1,9	1,9	1,9	1,9	1,8	1,6	10
1,9	1,9	1,9	1,9	1,9	1,9	1,7	1,7	1,5	10
1,9	1,9	1,9	1,9	10	10	10	10	10	10
1,9	1,9	1,9	1,9	1,9	1,9	1,9	1,9	1,6	1,6
1,6	1,8	1,8	10	10	1,8	1,9	10	10	10
1,9	1,9	1,9	1,9	1,9	1,8	1,8	1,8	1,9	10
1,9	1,9	1,9	1,9	1,9	1,9	1,9	1,9	1,6	10
10	1,9	1,9	1,9	10	1,9	10	10	1,9	10
1,9	1,9	1,9	1,9	10	10	1,9	1,9	1,9	10
10	10	1,9	10	10	10	10	10	10	10
1,9	1,9	1,9	10	10	10	10	10	10	10
1,9	10	1,9	1,9	10	10	10	10	1,9	10
10	10	10	10	10	10	1,6	10	1,9	10
10	10	10	10	10	10	10	10	1,9	10
1,9	1,9	1,9	1,9	1,9	1,9	10	10	1,5	10
1,9	1,9	1,9	1,9	1,9	1,9	1,9	1,5	10	10
1,9	1,9	1,9	1,9	10	10	10	10	1,5	10
1,9	1,9	1,9	1,9	1,9	1,8	10	10	1,9	10
10	10	1,9	1,9	1,9	1,9	10	10	1,9	10
1,9	1,9	1,9	1,9	1,9	1,5	1,9	10	1,9	10
10	1,9	1,9	10	10	1,9	1,9	10	1,9	10
1,9	1,9	1,9	1,9	1,9	1,9	1,9	1,9	1,5	10
1,9	1,9	1,9	1,9	1,9	1,9	1,9	1,9	1,9	10
1,5	1,9	1,9	1,9	1,9	10	10	10	10	10
1,9	1,9	1,9	1,9	1,9	1,8	1,8	10	1,9	10
2,9	2,9	1,9	10	1,9	1,9	10	10	1,9	1,9
1,8	1,5	1,9	1,9	10	10	1,5	1,5	1,5	10
2,9	2,9	1,9	10	1,9	1,9	10	10	1,9	1,9
1,9	1,9	1,9	1,9	1,9	1,9	1,9	1,9	1,9	10
1,9	1,9	1,9	10	1,9	1,9	1,9	1,9	10	10
1,9	1,9	1,9	1,9	1,9	1,8	1,9	10	1,9	10
10	10	10	10	10	10	10	10	1,9	10
1,9	1,9	1,9	1,9	1,9	1,9	1,9	1,9	1,5	10
1,9	1,9	1,9	1,9	1,9	1,8	1,9	10	1,9	10
1,9	1,9	1,9	1,9	10	10	1,5	10	1,9	10
10	10	10	10	10	10	10	10	10	10
1,5	1,5	1,9	1,9	10	10	10	10	10	10
10	10	1,9	1,9	10	10	10	10	1,9	10
1,9	1,9	1,9	1,9	1,9	1,9	10	1,9	1,9	10
1,9	1,9	1,9	1,9	10	10	10	1,9	10	10

10	1,5	1,9	1,9	10	10	10	10	10	10
10	2,9	2,9	2,9	2,9	10	2,5	10	1,9	10
10	1,9	1,9	1,9	10	10	10	10	1,9	10
10	1,9	1,9	1,9	10	1,9	10	1,9	1,9	10
1,9	1,9	1,9	10	10	10	10	10	10	10
1,9	1,9	1,9	1,9	10	1,5	10	10	1,9	10
10	10	1,9	10	10	10	10	1,9	1,9	10
10	10	1,9	1,9	10	10	1,5	10	1,5	10
10	10	1,9	1,9	10	10	1,8	10	1,9	10
1,9	1,9	1,9	1,9	1,9	1,9	1,9	1,9	1,5	10
1,9	1,9	1,9	1,9	1,9	1,9	10	10	1,9	10
1,9	1,9	1,9	1,9	1,9	1,9	1,9	1,9	1,6	10
1,9	1,9	1,9	1,9	1,9	1,6	10	10	1,9	10
1,9	1,9	1,9	1,9	1,9	1,6	10	10	1,9	10
1,9	1,9	1,9	1,9	1,9	1,8	1,8	10	1,9	10
10	1,5	1,5	1,5	10	10	10	10	10	10
1,9	1,9	1,9	1,9	1,9	10	1,8	10	1,9	10
1,9	1,9	1,9	1,9	1,9	10	1,8	10	1,9	10
1,9	1,9	1,9	1,9	1,9	1,8	10	10	1,9	10
1,9	1,9	1,9	1,9	1,9	1,8	10	10	1,9	10
1,9	1,9	1,9	1,9	1,9	1,9	1,9	1,9	1,9	10
1,5	1,5	1,5	1,5	10	10	10	10	10	10
10	1,8	10	10	10	10	10	10	1,5	10
10	1,9	2,9	2,9	2,9	10	10	10	1,9	10
10	10	3,9	2,9	1,9	10	10	10	1,9	10
1,8	1,8	1,8	2,9	2,9	10	1,9	1,9	1,9	10
10	10	3,9	2,9	1,9	1,9	10	10	1,9	10
1,5	1,5	1,9	1,9	1,5	1,5	1,5	1,5	1,9	10
1,9	1,9	1,9	1,9	1,9	1,9	1,9	1,9	1,9	10
1,9	1,9	1,9	1,9	1,9	1,9	1,5	1,5	1,5	10
1,9	1,9	1,9	1,9	1,9	1,8	1,8	10	1,9	10
1,9	1,9	1,9	10	10	10	10	10	1,9	10
1,9	1,9	3,9	3,9	1,9	10	10	10	1,9	10
1,9	1,9	1,9	1,5	1,5	1,5	10	10	1,9	10
1,5	1,5	1,6	10	1,6	1,6	1,6	1,6	1,6	10
1,9	1,9	1,9	1,9	1,9	1,8	10	10	10	10
1,5	1,5	1,9	1,9	1,5	1,5	1,9	10	1,9	10
1,6	1,6	1,6	10	1,5	1,5	1,6	10	1,6	10
1,9	1,9	1,9	1,9	1,9	1,9	1,9	1,9	1,9	10
1,8	1,9	1,9	1,9	1,9	1,8	1,9	10	1,9	10
10	1,9	1,9	10	10	10	1,5	1,5	1,5	1,5
10	10	1,9	1,9	1,9	1,9	10	10	1,9	10
10	10	3,9	2,9	2,9	1,9	10	10	1,9	10
1,9	1,9	1,9	1,9	1,9	1,9	1,5	1,5	1,5	10
1,9	1,9	1,9	1,9	1,9	10	10	10	10	10
1,8	1,9	1,9	1,9	1,9	1,8	10	10	1,9	10

thanzwa	mazwilu	makwakw	masala	baobab fru	fig	tintoma	nombelo	marula fru	Nii
10	10	10	10	10	10	10	10	10	1,5
10	10	10	10	10	10	10	10	10	1,5
10	1,9	10	10	1,7	10	10	1,5	10	1,7
10	10	10	10	2,9	10	10	1,9	10	1,9
10	1,5	10	10	1,6	10	10	10	10	1,5
10	10	10	10	1,9	10	10	10	10	10
10	1,9	10	10	1,9	10	1,9	10	10	1,9
10	10	10	10	10	10	10	10	10	4,9
10	2,9	10	10	2,9	10	10	2,6	2,6	2,6
10	1,9	10	10	1,9	10	10	10	10	2,9
10	10	10	10	1,5	10	10	10	10	1,7
10	1,5	10	10	2,5	10	10	2,5	10	2,5
10	10	10	10	2,5	10	10	2,9	1,9	2,5
10	10	10	10	1,9	1,9	10	10	10	1,9
1,9	1,9	10	10	1,9	10	10	10	10	1,9
10	10	10	10	1,9	10	10	1,9	10	2,9
10	10	10	10	2,9	10	10	2,9	10	2,9
1,5	10	10	10	2,6	10	10	1,5	2,5	2,6
1,5	1,5	1,5	10	2,6	1,5	10	2,5	1,5	2,6
10	1,5	10	10	2,6	10	10	2,5	10	10
1,5	1,5	10	10	2,6	10	10	2,5	10	2,6
10	1,5	10	10	2,5	10	10	1,5	10	2,5
10	2,5	10	10	2,6	10	10	1,5	10	2,6
10	10	10	10	10	10	10	1,9	10	1,9
1,9	1,5	10	10	2,5	10	10	1,9	10	1,9
10	10	10	10	10	10	10	1,9	10	1,9
10	10	10	10	2,9	10	10	10	10	1,9
1,9	10	10	10	4,9	10	10	10	10	4,9
10	10	10	10	1,5	10	10	1,9	10	1,5
10	10	10	10	1,9	10	10	10	10	1,7
10	10	10	10	1,6	10	10	10	10	1,5
10	10	10	10	2,9	10	10	1,6	10	2,6
10	10	1,5	1,8	1,6	10	10	2,8	2,6	2,6
1,9	1,9	10	10	1,9	10	10	1,9	10	10
10	10	10	10	1,9	10	10	10	10	1,9
10	10	10	10	2,9	10	10	10	10	2,9
10	10	10	10	1,9	10	10	1,8	10	1,8
10	10	10	10	1,6	10	10	10	1,9	1,9
10	10	10	10	2,6	10	10	2,6	2,6	2,6
10	10	10	10	10	10	10	10	10	10
10	10	10	10	1,5	10	10	10	10	1,6
10	10	10	10	1,5	10	10	1,5	10	1,6
10	10	10	10	2,9	10	10	1,9	10	2,9
10	1,9	10	10	1,5	10	10	1,9	10	1,5
10	10	10	10	1,9	10	10	10	10	1,9
10	10	10	10	1,9	10	10	1,9	10	10

10	10	10	10	1,9	10	10	1,9	10	10
1,9	10	2,6	10	3,4	10	10	1,9	10	3,6
1,5	10	10	10	2,6	10	10	2,6	2,6	2,6
10	1,5	10	10	1,6	10	10	10	10	1,7
1,9	10	2,9	10	1,7	10	10	1,5	1,9	1,9
10	10	10	10	2,9	10	10	1,9	10	1,9
10	10	10	10	10	10	10	10	10	1,5
10	10	10	10	10	10	10	10	10	10
10	10	10	10	1,9	10	10	10	10	10
10	10	10	10	2,9	10	10	10	10	2,9
10	10	10	10	2,9	10	10	10	10	2,9
10	10	10	10	1,9	10	10	10	10	10
10	10	10	10	1,9	10	10	10	10	10
1,5	1,6	10	10	1,6	10	10	1,5	10	1,6
10	4,9	10	10	2,5	10	10	10	10	2,6
10	1,9	10	10	1,9	10	10	10	10	1,9
10	10	10	10	2,9	10	10	10	10	10
3,9	10	1,5	10	10	10	10	10	1,7	10
1,9	10	10	10	1,9	10	10	10	10	1,9
1,9	10	10	10	1,5	10	10	10	10	10
1,6	1,6	10	10	2,6	10	10	2,6	2,6	2,6
10	2,9	10	10	2,6	10	10	2,6	10	2,6
10	2,9	10	10	2,6	10	10	2,6	10	2,6
10	10	10	10	1,9	10	10	10	10	1,5
10	10	10	10	1,9	10	10	10	10	1,5
10	10	10	10	2,9	10	10	10	10	2,9
10	10	10	10	1,6	10	10	10	10	1,6
10	10	10	10	1,9	10	10	1,9	10	1,9
10	1,8	10	10	1,5	1,5	10	1,5	10	1,5
1,5	1,5	10	10	2,6	10	10	10	10	10
10	10	10	10	1,5	10	10	10	10	1,9
10	10	10	10	1,5	10	10	10	10	1,9
10	10	10	10	1,5	10	10	10	10	10
10	10	10	10	10	10	10	10	1,9	1,8
10	10	10	10	1,5	10	10	10	10	1,5
10	10	10	10	2,5	10	10	2,5	10	2,5
10	2,9	10	10	2,6	10	10	2,6	10	2,6
10	1,9	10	10	10	1,9	10	10	10	10
10	2,9	10	10	2,9	10	10	10	10	2,9
10	10	10	10	1,5	10	10	1,7	10	1,5
10	1,9	10	10	1,9	10	10	1,9	10	1,9
1,9	1,9	1,9	1,9	1,9	10	10	1,9	10	1,9
10	10	10	10	1,9	10	10	10	10	1,9
10	1,9	10	10	1,9	10	10	1,9	10	1,9
10	1,9	10	10	1,9	10	10	1,9	10	1,9
10	1,9	10	10	1,9	10	10	1,9	10	1,9
1,5	10	10	10	1,8	1,6	10	1,6	10	10

1,9	10	10	1,9	10	10	10	1,9	10	10
10	10	10	10	10	10	10	10	1,9	1,9
1,9	10	10	10	1,9	10	10	1,9	10	1,8
1,9	1,9	1,9	1,9	10	10	10	1,9	10	10
1,9	1,9	1,9	1,9	1,9	10	10	1,9	10	1,9
3,6	3,6	10	2,5	10	2,5	10	2,7	10	10
10	10	10	10	1,5	1,5	10	1,9	10	1,9
10	1,9	10	10	10	10	10	1,9	10	10
10	1,5	10	1,9	1,9	10	10	1,9	10	10
1,9	1,9	10	10	1,9	10	10	10	1,9	3,9
10	10	10	10	2,9	10	10	10	10	2,9
10	10	10	10	1,9	10	10	10	10	1,9
10	10	10	10	1,9	10	10	10	10	1,9
10	10	10	10	10	10	10	10	10	10
10	10	10	10	2,9	10	10	10	10	2,9
1,8	1,9	1,5	1,5	2,6	1,9	10	1,6	10	2,6
10	2,8	10	10	2,9	10	10	10	1,9	10
10	10	10	10	1,5	10	10	10	1,5	1,7
10	10	10	10	2,6	10	10	10	10	1,5
10	10	10	1,9	1,9	10	10	1,9	10	1,9
10	1,5	10	10	2,6	10	10	2,6	10	2,6
10	10	10	10	1,9	10	10	10	1,9	1,9
10	10	10	10	10	10	10	1,5	10	10
10	10	10	10	10	10	10	1,5	10	10
10	10	10	10	1,5	10	10	1,5	10	10
10	10	10	10	10	2,5	10	10	10	3,9
10	1,9	10	10	10	10	10	10	10	1,9
10	10	10	10	1,6	10	10	1,9	10	1,6
10	10	10	10	10	1,9	10	10	10	1,9
10	1,9	10	10	1,6	1,5	10	2,6	10	10
10	10	10	10	1,9	1,9	10	10	10	10
10	10	10	10	10	10	10	10	10	10
10	10	10	10	2,6	10	10	2,6	10	2,6
1,9	1,9	10	10	1,9	10	10	1,9	1,9	1,9
10	10	10	10	2,5	10	10	10	10	10
10	10	10	10	10	10	10	10	10	10
10	10	10	10	10	10	10	10	10	10
10	10	10	10	2,6	10	10	10	10	10
1,9	1,9	10	10	1,9	10	10	10	1,9	1,9
1,9	1,9	10	10	10	10	10	10	1,9	1,9
10	10	10	10	1,9	10	10	1,9	10	10
10	10	10	10	1,5	10	10	10	10	1,5
10	10	10	10	1,9	10	10	10	10	10
10	10	10	10	1,5	10	10	10	10	10
10	10	10	10	2,6	10	10	2,6	10	1,5
10	10	10	10	1,9	10	10	1,5	10	1,5
10	10	10	10	2,5	10	10	2,6	10	2,6

10	10	10	10	1,9	10	10	10	10	1,9
1,9	10	10	10	2,9	10	10	2,9	10	3,9
10	10	10	10	1,9	10	10	10	10	1,9
10	10	10	10	1,9	10	10	10	10	1,9
2,9	10	10	10	1,9	10	10	10	10	2,9
10	10	10	10	1,5	10	10	10	1,9	1,5
1,5	2,9	1,5	1,5	2,6	1,5	10	1,5	2,5	2,6
10	10	10	10	1,5	10	10	10	10	1,5
10	10	10	10	3,9	10	10	10	10	1,9
10	10	10	10	1,6	10	10	10	1,5	1,5
10	10	10	10	1,9	10	10	10	1,9	1,9
10	10	10	10	1,9	10	10	10	1,9	1,9
10	10	10	10	1,9	10	10	10	1,9	1,9
10	10	10	10	1,5	10	10	10	10	1,5
10	10	10	10	1,5	10	10	10	10	1,5
10	10	10	10	10	10	10	10	10	1,5
10	10	10	10	1,5	10	10	10	10	10
10	10	10	10	10	10	10	10	10	10
10	10	10	10	2,5	10	10	2,5	10	2,5
10	10	10	10	1,9	10	10	10	1,5	10
10	10	10	10	10	10	10	10	10	10
10	10	10	10	1,9	10	10	10	10	1,9
10	10	10	10	1,9	10	10	10	10	1,9
10	10	10	10	1,9	10	10	10	10	1,9
10	10	10	10	1,9	10	10	10	10	1,9
10	10	10	10	1,5	10	10	10	10	1,6
10	10	10	10	1,7	10	10	10	10	1,6
10	10	10	10	1,9	10	10	10	10	1,9
10	10	10	10	1,9	10	10	10	10	1,9
10	10	10	10	2,6	10	10	2,5	10	2,5
10	10	10	10	2,9	10	10	10	10	10
10	10	10	10	1,9	10	10	10	10	1,9
10	10	10	10	1,5	10	10	10	10	10
10	10	10	10	2,6	10	10	2,6	10	1,5
10	10	10	10	1,5	10	10	1,5	10	1,5
10	10	10	10	10	10	10	1,9	10	1,9
10	10	10	10	1,9	10	10	10	10	1,9
10	10	10	10	1,9	10	10	10	10	1,9
10	10	10	10	1,5	10	10	10	10	10
10	10	10	10	1,5	10	10	10	10	10
10	1,9	10	10	10	10	10	10	1,9	1,9
10	10	10	10	1,9	10	10	10	10	1,9
10	10	10	10	10	10	10	10	10	1,9
1,9	1,9	1,5	10	1,6	1,6	10	1,9	10	1,5
10	10	10	10	1,5	10	10	1,9	10	10
10	10	10	10	10	10	10	10	10	1,9
1,5	1,5	1,5	1,5	1,9	1,9	1,9	10	10	2,9

10	10	10	10	1,9	10	10	10	10	1,9
10	10	10	10	1,9	10	10	1,9	10	1,9
1,9	10	10	10	1,9	10	10	1,9	10	1,9
1,5	10	10	10	2,6	10	10	2,6	1,5	2,6
1,5	10	10	10	2,6	10	10	2,6	1,5	2,6
10	10	10	10	1,9	10	10	1,9	10	1,9
10	10	10	10	10	10	10	10	10	1,9
10	10	10	10	2,9	10	10	10	10	1,9
10	10	10	10	1,5	10	10	10	10	10
10	10	10	10	1,9	10	10	10	10	10
10	10	10	10	10	10	10	10	10	1,5
10	10	10	10	1,9	10	10	10	10	1,9
10	10	10	10	1,9	10	10	1,9	10	1,9
10	10	10	10	1,9	10	10	10	10	1,9
10	10	10	10	1,5	10	10	10	10	1,5
10	10	10	10	1,9	10	10	10	10	1,9
10	10	10	10	1,5	10	10	10	10	1,5
10	10	10	10	1,9	10	10	10	10	1,9
10	10	10	10	1,9	10	10	1,9	10	1,9
10	10	10	10	2,9	10	10	10	10	1,9
10	10	10	10	10	10	10	10	10	1,8
10	10	10	10	1,5	10	10	10	10	1,5
10	10	10	10	1,5	10	10	10	10	1,5
10	10	10	10	1,9	10	10	10	10	10
10	10	10	10	1,9	10	10	10	10	1,9
10	10	10	10	1,6	10	10	10	1,5	1,5
1,5	1,5	10	10	1,5	10	10	2,5	1,5	2,6
10	10	10	10	1,9	1,9	10	10	10	1,9
10	10	10	10	10	10	10	10	10	10
10	10	10	10	2,9	10	10	10	10	10
10	10	10	10	1,9	10	10	10	10	1,9
10	10	10	10	10	10	10	10	10	1,8
10	10	10	10	1,5	10	10	10	10	1,5
10	10	10	10	1,9	10	10	1,8	10	1,9
10	10	10	10	1,9	10	10	10	10	1,9
10	10	10	10	1,9	10	10	10	10	10
10	10	10	10	1,5	10	10	10	10	10
10	10	10	10	1,9	10	10	10	10	10
10	10	10	10	1,9	10	10	10	10	1,9
10	10	10	10	1,5	10	10	1,5	10	1,5
10	10	10	10	1,9	10	10	10	10	1,9
10	10	10	10	1,9	10	10	1,9	10	1,9
10	10	10	10	1,9	10	10	10	10	1,9
10	10	10	10	2,5	10	10	10	10	10
10	10	10	10	1,9	10	10	10	10	1,9
10	10	10	10	10	10	10	10	10	10
10	10	10	10	1,5	10	10	1,5	2,5	2,5

10	10	10	10	1,5	10	10	1,5	2,5	2,5
10	10	10	10	10	10	10	10	10	10
10	10	10	10	1,9	10	10	1,9	10	1,9
10	10	10	10	1,9	10	10	1,9	10	10
1,9	10	10	10	1,9	10	10	10	10	10
10	10	10	10	1,9	10	10	10	10	10
10	10	10	10	1,9	10	10	10	10	1,9
1,9	10	10	10	1,9	10	10	1,9	10	1,9
10	10	10	10	1,9	10	10	10	10	1,9
10	10	10	10	1,9	10	10	1,9	10	1,9
1,9	1,9	10	1,9	1,9	1,9	10	2,9	10	1,5
1,8	1,5	10	1,5	10	10	1,9	1,9	10	1,5
10	10	10	10	10	10	10	10	10	1,9
10	10	10	10	10	10	10	10	10	1,9
10	10	10	10	10	10	10	2,9	10	3,9
10	10	10	10	1,9	10	10	1,8	10	1,8
2,6	2,6	1,5	1,5	1,5	1,5	10	2,6	1,5	1,5
2,6	2,6	1,5	1,5	1,5	1,5	10	2,6	1,5	1,5
10	10	10	10	10	10	10	2,9	10	10
10	1,9	10	1,9	1,9	10	10	1,9	10	1,9
2,9	10	10	2,9	1,8	10	10	2,9	10	10
10	10	10	10	1,8	10	10	2,9	10	1,9
10	2,9	10	10	10	10	10	1,9	10	1,9
10	10	10	10	10	10	10	3,9	10	10
10	10	10	10	10	10	10	3,9	10	10
10	10	10	10	10	10	10	1,9	10	10
10	10	10	10	10	10	10	2,9	10	3,9
10	1,9	10	10	1,8	10	10	1,9	10	1,9
1,9	2,9	10	1,9	1,9	10	10	2,8	10	1,9
10	10	10	10	10	10	10	2,9	10	10
10	10	10	10	10	10	10	10	10	10
10	10	10	10	1,9	10	10	2,9	10	10
10	10	10	10	10	10	10	1,9	10	1,9
1,9	1,9	10	1,9	1,9	1,9	1,9	1,9	10	1,5
1,6	2,6	10	1,5	1,5	1,5	10	2,7	10	1,5
10	10	10	10	1,6	10	10	1,9	10	1,9
1,9	10	10	10	1,9	10	10	1,9	1,9	2,9
10	10	10	10	1,9	10	10	10	10	1,9
10	1,9	10	10	1,9	10	10	1,9	10	1,9
10	1,9	10	10	1,9	10	10	1,9	10	1,9
10	1,9	10	1,9	10	10	10	3,9	10	2,9
10	10	10	10	10	10	10	10	10	10
10	10	10	10	1,9	10	10	10	10	1,9
10	10	10	10	1,9	10	10	10	10	10
10	10	10	10	10	10	10	10	10	10
10	10	10	10	1,9	10	10	10	10	1,9
10	10	10	10	1,8	10	10	1,6	10	10

10	10	10	10	1,9	10	10	1,9	10	10
10	10	10	10	1,4	10	10	10	10	10
10	10	10	10	10	10	10	10	10	10
10	1,9	10	10	1,9	10	10	10	10	1,9
10	10	10	10	10	10	10	10	10	10
10	10	10	10	10	10	10	10	10	1,9
10	10	10	10	10	10	10	10	10	10
10	10	10	10	10	10	10	10	10	10
10	10	10	10	1,6	10	10	10	10	10
10	10	10	10	1,6	10	10	10	10	10
10	10	10	10	10	10	10	10	10	10
10	10	10	10	10	10	10	10	10	10
10	10	10	10	10	10	10	10	10	1,9
10	10	10	10	10	10	10	10	10	10
1,9	1,9	10	10	1,9	10	10	1,9	10	1,9
10	10	10	10	1,5	10	10	10	10	1,9
10	10	10	10	1,9	10	10	10	10	1,9
10	10	10	10	10	10	10	1,9	1,9	1,9
10	10	10	10	10	10	10	10	10	10
10	10	10	10	1,9	10	10	10	1,9	1,9
10	10	10	10	10	10	10	1,9	1,9	1,9
10	10	10	10	1,9	10	10	10	10	1,9
10	10	10	10	1,9	10	10	10	10	1,9
1,9	10	10	10	1,9	10	10	10	10	1,9
10	10	10	10	1,9	10	10	10	1,9	1,9
10	10	10	10	1,9	10	10	1,5	1,5	1,5
1,9	10	10	10	1,9	10	10	10	10	1,9
10	10	10	10	1,5	10	10	1,5	1,5	1,5
10	10	10	10	1,9	10	10	10	10	1,9
10	10	10	10	1,9	10	10	10	10	1,5
10	10	10	10	10	10	10	10	10	10
10	10	10	10	10	10	10	1,9	10	10
10	10	10	10	10	10	10	1,9	10	10
10	10	10	10	1,9	10	10	1,9	1,9	1,9
10	10	10	10	1,9	10	10	10	10	1,9
1,9	1,9	10	10	10	10	10	1,9	10	10
10	10	10	10	1,9	10	10	10	1,9	1,9
10	10	10	10	1,9	10	10	10	10	1,9
10	10	10	10	1,9	10	10	1,9	10	1,9
10	10	10	10	1,9	10	10	10	10	1,9
10	10	10	10	1,9	10	10	1,9	10	10
10	10	10	10	10	10	10	1,9	1,9	1,9
10	10	10	10	1,9	10	10	10	10	1,9
10	10	10	10	10	10	10	1,9	10	10
1,9	1,9	1,9	10	1,9	10	10	1,9	1,9	1,9
10	10	10	10	1,9	10	10	10	1,9	10
10	10	10	10	1,9	10	10	10	10	1,9

10	10	10	10	1,9	10	10	10	10	1,9
10	1,9	10	10	1,9	10	10	10	1,9	1,9
10	10	10	10	10	10	10	10	10	10
10	10	10	10	10	10	10	10	10	10
10	10	10	10	1,9	10	10	10	1,9	10
10	10	10	10	10	10	10	1,9	1,9	1,9
10	10	10	10	1,9	10	10	10	10	1,9
10	10	10	10	1,9	10	10	10	10	1,9
10	10	10	10	10	10	10	10	1,9	1,9
10	10	10	10	1,5	10	10	10	1,6	10
1,9	10	10	10	1,9	10	10	10	10	1,9
10	10	10	10	10	10	10	1,9	1,9	1,9
10	10	10	10	1,9	10	10	10	10	1,9
10	10	10	10	1,9	10	10	10	10	1,9
10	10	10	10	1,9	10	10	10	10	1,9
10	10	10	10	1,9	10	10	10	10	1,9
10	10	10	10	1,9	10	10	10	10	1,9
10	10	10	10	1,9	10	10	10	10	1,9
10	10	10	10	1,9	10	10	10	1,9	10
10	1,8	1,8	10	1,5	10	10	10	1,9	1,9
10	10	10	10	1,7	10	10	10	10	10
10	10	10	10	1,9	10	10	10	10	1,9
10	10	10	10	1,9	10	10	10	10	1,9
10	10	10	10	10	10	10	10	10	10
10	10	10	10	1,9	10	10	10	10	1,9
10	10	10	10	1,9	10	10	10	10	1,9
10	10	10	10	1,9	10	10	1,9	10	1,9
10	10	10	10	1,9	10	10	10	10	1,9
10	10	10	10	1,9	10	10	10	10	1,9
10	10	10	10	1,9	10	10	10	10	1,9
2,9	10	10	10	10	10	10	10	10	10
1,9	1,9	10	1,9	1,9	10	10	1,9	10	10
10	10	10	1,5	10	10	10	10	10	10
1,9	10	10	10	1,9	10	10	1,9	10	1,9
2,9	2,9	10	2,9	10	10	10	2,9	2,9	10
10	1,5	10	1,5	1,9	1,9	1,9	1,9	1,9	1,9
1,9	10	10	10	10	10	10	4,9	10	10
10	10	10	10	1,5	10	10	1,9	10	10
1,9	1,9	10	1,9	1,9	1,9	1,9	1,9	10	1,8
10	10	10	1,9	1,9	1,9	1,9	1,9	10	1,9
10	10	10	10	10	10	10	10	10	10
10	10	10	10	1,9	10	10	10	10	1,9
10	10	10	10	3,6	10	10	10	10	10
1,9	10	10	10	10	10	10	1,9	10	1,9
10	10	10	10	10	10	10	10	10	10
10	10	10	10	1,9	10	10	10	10	10
10	10	10	10	10	10	10	10	10	1,9

10	10	10	10	1,9	10	10	10	10	1,9
10	10	10	10	10	10	10	10	10	10
10	10	10	10	1,9	10	10	1,9	1,9	1,9
10	10	10	10	1,9	10	10	10	10	1,9
10	10	10	10	1,9	10	10	10	10	1,9
10	10	10	10	1,9	10	10	10	10	1,9
10	10	10	10	1,9	10	10	10	10	1,9
10	10	10	10	1,9	10	10	10	10	1,9
10	10	10	10	1,5	10	10	10	10	1,5
10	10	10	10	1,9	10	10	10	10	10
10	10	10	10	1,9	10	10	1,9	10	1,9
10	10	10	10	10	10	10	10	10	10
10	10	10	10	1,9	10	10	10	10	10
10	10	10	10	10	10	10	10	10	10
10	10	10	10	1,9	10	10	10	10	10
10	10	10	10	1,9	10	10	10	10	1,9
10	10	10	10	10	10	10	10	10	10
10	10	10	1,9	1,9	10	10	10	10	10
10	10	10	10	1,5	10	10	10	1,9	1,9
10	10	10	10	10	10	10	10	10	10
10	10	10	10	1,9	10	10	10	10	1,9
10	10	10	10	1,5	10	10	1,5	1,5	1,5
10	10	10	10	10	10	10	10	10	10
10	10	10	10	1,9	10	10	10	10	1,9
10	10	10	10	10	10	10	10	10	10
10	10	10	10	10	10	10	10	10	10
10	10	10	10	1,6	10	10	10	1,6	10
10	10	10	10	1,9	10	10	10	10	10
10	10	10	10	1,9	10	10	10	10	10
10	10	10	10	1,5	10	10	10	10	10
10	10	10	10	1,6	10	10	10	10	10
10	10	10	10	1,9	10	10	10	1,8	1,8
1,9	1,9	1,9	10	1,9	1,9	10	1,9	1,9	1,9
10	10	10	10	1,9	10	10	10	10	1,9
10	10	10	10	10	10	10	10	10	10
10	10	10	10	1,9	10	10	10	10	10
10	10	10	10	1,9	10	10	10	1,5	10
10	10	10	10	1,9	10	10	10	1,5	10
10	10	10	10	10	10	10	10	1,9	1,9
10	10	10	10	10	10	10	10	10	10
1,9	10	10	10	1,9	10	10	1,9	1,9	1,9
10	10	10	10	1,6	10	10	10	10	10
10	10	10	10	10	10	10	10	1,9	10
10	10	10	10	10	10	10	10	10	10
10	10	10	10	1,9	10	10	10	10	10
1,9	1,9	1,9	1,9	1,9	1,9	10	1,9	1,9	1,9
10	10	10	10	10	10	10	1,9	10	1,9

10	10	10	10	1,9	10	10	10	10	10
10	10	10	10	1,9	10	10	10	10	10
10	10	10	10	10	10	10	10	10	10
10	10	10	10	10	10	10	10	10	10
10	10	10	10	10	10	10	10	10	10
10	10	10	10	10	10	10	10	10	1,9
10	10	10	10	10	10	10	10	10	10
10	1,6	10	10	1,9	10	10	1,9	10	1,5
10	1,9	10	10	1,5	10	10	1,8	10	1,5
10	10	10	10	10	10	10	10	10	10
10	10	10	10	1,6	1,6	10	10	1,5	1,5
10	10	10	10	10	10	10	10	10	10
10	10	10	10	1,9	1,9	10	10	10	1,9
10	10	10	10	1,5	10	10	10	10	1,5
10	10	10	10	1,9	10	10	10	10	1,9
10	10	10	10	10	10	10	10	10	1,9
10	10	10	10	10	10	10	10	10	10
10	10	10	10	10	10	10	10	10	10
10	10	10	10	1,9	10	10	10	10	1,9
10	10	10	10	10	10	10	10	10	10
10	10	10	10	10	10	10	10	10	10
10	10	10	10	1,5	10	10	10	10	1,9
10	10	10	10	10	10	10	10	10	10
10	10	10	10	1,9	10	10	10	10	1,9
10	10	10	10	1,9	10	10	10	10	10
10	10	10	10	1,9	10	10	10	1,9	10
10	10	10	10	1,9	10	10	1,9	10	1,9
10	10	10	10	10	10	10	10	10	10
10	10	10	10	1,5	10	10	1,9	1,9	1,9
10	10	10	10	1,9	10	10	1,9	10	1,9
10	10	10	10	10	10	10	10	10	10
10	10	10	10	1,9	10	10	1,9	10	1,9
10	10	10	10	2,9	1,9	10	2,9	10	3,7
10	10	10	10	10	10	10	10	10	10
10	10	10	10	1,9	1,9	10	1,9	10	3,7
10	10	10	10	1,5	1,5	1,5	1,5	1,5	1,5
10	10	10	10	10	10	10	1,9	10	1,8
10	10	10	10	1,9	10	10	10	10	1,9
10	10	10	10	10	10	10	10	10	10
1,9	1,5	1,9	10	1,9	1,9	10	10	1,9	1,9
10	10	10	10	1,9	10	10	10	10	1,9
10	10	10	10	1,9	10	10	10	10	1,9
10	10	10	10	10	10	10	10	10	10
10	10	10	10	10	10	10	10	10	10
10	10	10	10	10	10	10	10	10	1,9
10	1,9	10	10	1,9	10	10	1,9	10	1,9
10	10	10	10	1,9	10	10	10	10	1,9

10	10	10	10	10	10	10	10	10	10
10	10	10	10	1,9	10	10	1,9	10	1,9
10	10	10	10	1,8	10	10	10	10	1,9
10	10	10	10	1,9	10	10	10	10	1,9
10	10	10	10	10	10	10	10	10	10
10	10	10	10	1,9	10	10	10	10	1,9
10	10	10	10	1,9	10	10	10	10	10
10	10	10	10	10	10	10	1,9	10	10
10	10	10	10	1,9	10	10	10	10	1,9
10	10	10	10	1,5	10	10	10	10	1,5
10	10	10	10	1,9	10	10	10	1,9	10
1,9	1,9	10	10	10	10	10	1,9	10	1,9
10	10	10	10	1,9	10	10	10	10	1,9
10	10	10	10	1,9	10	10	10	10	1,9
1,9	1,9	10	10	1,9	1,9	10	10	1,9	1,9
10	10	10	10	10	10	10	10	10	10
10	10	10	10	1,9	10	10	10	10	1,9
10	10	10	10	1,9	10	10	10	10	1,9
10	10	10	10	1,9	10	10	10	10	1,9
10	10	10	10	1,9	10	10	1,9	10	1,9
10	10	10	10	1,9	10	10	10	10	10
10	10	10	10	10	10	10	10	10	10
10	10	10	10	10	10	10	10	10	1,9
10	10	10	10	10	10	10	2,9	10	10
10	10	10	10	10	10	10	1,9	10	10
10	10	10	10	1,9	10	10	1,9	10	10
10	10	10	10	10	10	10	1,8	10	10
10	10	10	10	10	10	10	10	10	10
10	10	10	10	1,9	10	10	10	10	10
1,5	1,5	10	1,5	1,5	1,5	1,5	1,9	10	1,9
1,9	1,9	10	10	1,9	10	10	1,9	10	1,9
1,9	10	10	10	1,9	10	10	1,5	1,5	1,5
10	10	10	10	10	10	10	2,9	10	10
10	10	10	10	10	10	10	10	10	10
10	10	10	10	1,6	10	10	1,6	10	10
10	1,9	10	1,9	1,9	10	10	1,9	10	1,9
10	1,9	10	1,9	1,9	10	10	1,9	10	1,9
1,6	1,6	10	1,6	1,7	1,6	10	1,6	10	10
10	10	10	10	10	10	10	1,9	10	10
10	1,9	10	1,9	1,9	10	10	1,9	10	1,9
1,5	1,5	1,5	10	10	10	10	10	10	10
10	10	10	10	10	10	10	1,9	10	10
10	10	10	10	10	10	10	1,9	10	10
1,5	1,5	10	1,5	1,5	1,5	1,5	1,9	10	1,9
10	10	10	10	10	10	10	2,9	10	10
1,9	1,9	10	1,9	1,9	10	10	1,9	10	1,9

xirhomber	mobola plı	cooked spi	stewed spi	stewed spi	stewed spi	stewed spi	boiled pota	shallow fri	stewed po
10	10	10	10	10	1,8	10	10	1,5	1,6
10	10	10	10	10	1,8	10	10	1,5	1,6
10	1,9	1,8	1,6	1,6	10	1,6	10	1,6	1,5
10	10	10	1,5	10	10	10	10	10	1,5
10	10	1,5	1,5	10	10	10	10	10	1,5
10	10	10	10	10	10	10	1,5	10	10
10	10	10	1,5	10	10	10	10	10	1,5
10	10	10	10	1,5	10	10	10	10	10
10	10	2,5	10	10	10	10	10	1,6	2,6
10	10	10	10	10	10	10	10	10	1,5
10	10	1,6	10	10	10	10	1,6	10	1,5
10	10	2,5	2,5	2,5	10	10	2,5	2,5	2,5
10	10	2,5	2,5	2,5	10	10	10	1,5	2,5
10	10	1,9	10	10	10	10	1,9	10	10
10	10	10	10	10	10	10	10	1,5	1,5
10	10	10	1,5	10	10	10	10	1,6	1,5
10	10	10	1,5	10	10	10	10	1,6	1,5
10	10	10	2,5	2,5	10	10	10	10	2,5
10	10	1,5	2,5	2,5	10	10	1,5	10	2,5
10	10	1,5	2,5	2,5	10	10	1,5	10	2,5
10	10	1,5	2,5	10	10	10	10	10	2,5
10	10	1,5	1,5	2,5	2,5	10	10	10	2,5
10	10	2,5	2,6	2,6	2,5	10	1,5	1,5	2,6
10	10	1,5	2,5	2,5	10	10	1,5	1,5	2,5
10	10	1,5	2,5	2,5	10	10	1,5	1,5	2,5
10	10	1,5	2,5	2,5	10	10	1,5	1,5	2,5
10	10	10	10	10	10	10	10	10	10
10	10	10	1,5	1,5	10	10	10	1,5	1,5
10	10	1,5	1,5	1,5	10	10	1,5	1,5	1,5
10	10	10	2,6	10	10	10	1,8	1,6	1,7
10	10	1,5	1,5	10	10	1,5	10	10	1,5
10	10	10	2,6	2,6	10	10	10	10	2,5
10	10	10	2,6	2,6	10	10	10	10	2,6
10	1,9	10	1,9	10	10	10	1,5	10	10
10	10	10	1,5	10	10	10	10	1,5	1,5
10	10	10	2,5	1,5	10	10	10	1,5	2,5
10	10	10	1,5	10	10	10	10	1,5	1,5
10	10	10	10	10	10	10	10	10	1,5
10	10	10	10	10	10	10	10	10	2,6
10	10	1,5	10	10	10	10	10	1,5	1,5
10	10	10	1,6	1,6	10	10	10	1,6	1,6
10	10	1,6	1,6	1,6	1,6	10	1,5	1,5	1,5
10	10	10	1,5	1,5	10	10	10	1,5	1,5
10	10	1,5	1,5	10	10	10	10	10	10
10	10	1,5	1,5	1,5	10	10	10	1,5	1,5
10	10	1,9	10	10	10	10	10	1,5	1,5

10	10	1,9	1,9	10	10	10	10	10	1,5
10	10	10	2,5	10	10	10	10	1,8	4,5
10	10	10	2,6	10	2,6	10	10	10	2,6
10	10	1,5	1,5	1,5	10	10	1,5	1,5	1,5
10	10	10	1,5	1,5	10	10	10	1,5	1,6
10	10	1,5	1,5	10	10	10	10	10	1,5
10	10	10	10	1,5	10	10	10	1,5	1,5
10	10	10	10	10	10	10	10	1,5	1,5
10	10	10	1,8	1,8	10	10	10	1,5	1,5
10	10	1,8	1,8	1,8	1,8	1,8	10	1,5	1,5
10	10	10	1,5	1,5	10	10	10	2,5	1,5
10	10	10	1,8	1,8	10	10	10	10	1,5
10	10	10	1,8	1,8	10	10	10	1,5	1,5
10	10	1,6	10	2,6	10	1,5	2,6	1,4	10
10	10	10	1,5	10	10	10	10	10	1,5
10	10	1,9	1,9	10	10	1,9	10	10	10
10	10	10	1,8	10	10	10	10	2,7	1,8
10	10	10	1,6	10	10	2,6	10	1,6	1,5
10	10	1,5	1,5	10	10	10	10	1,5	1,5
10	10	1,5	1,5	10	10	10	10	10	1,5
10	10	10	2,6	1,5	1,5	10	10	10	2,6
10	10	10	2,6	2,6	10	10	10	10	2,5
10	10	10	2,6	2,6	10	10	10	10	2,5
10	10	1,5	1,5	10	10	10	10	1,8	1,5
10	10	1,5	1,5	10	10	10	10	1,8	1,5
10	10	10	10	10	10	10	10	10	2,8
10	10	1,5	1,5	1,5	1,5	10	10	1,5	1,5
10	10	1,9	1,9	10	10	10	1,5	10	10
10	10	1,5	1,5	1,5	10	1,5	10	1,6	2,5
10	10	1,5	1,5	1,5	10	10	10	10	1,5
10	10	10	1,5	1,5	10	10	10	1,5	1,5
10	10	10	1,5	1,5	10	10	10	1,5	1,5
10	10	1,5	10	1,5	10	10	10	1,5	1,5
10	10	10	1,7	2,6	1,6	1,5	1,5	2,7	1,6
10	10	10	10	10	10	10	10	1,5	1,5
10	10	10	10	2,6	2,5	10	10	10	1,5
10	10	2,5	10	10	10	10	10	10	2,5
10	10	1,9	10	1,9	10	10	10	10	1,9
10	10	10	2,5	2,5	10	10	10	10	2,5
10	10	1,5	1,5	2,6	10	10	10	10	2,6
10	10	1,5	1,5	1,5	10	10	10	1,5	1,5
10	10	10	1,5	10	10	1,5	10	1,5	1,5
10	10	1,5	1,5	1,5	10	10	10	1,5	1,5
10	10	1,5	1,5	1,5	10	10	1,5	1,5	1,5
10	10	1,9	1,9	1,9	10	10	10	1,5	1,5
10	10	1,9	1,9	1,9	10	10	10	1,8	1,5
10	10	2,5	2,5	2,5	10	10	10	10	10

10	10	10	10	1,9	10	10	10	10	1,9
10	10	10	10	10	10	10	10	10	1,5
10	10	10	1,7	1,7	10	10	1,6	1,5	1,5
10	10	10	10	1,5	10	10	10	10	1,5
10	10	10	10	10	10	10	10	1,5	1,5
10	10	2,5	10	10	10	10	10	2,5	10
10	10	2,5	2,5	2,5	2,5	2,5	10	10	2,5
10	10	1,5	1,5	1,5	10	10	10	1,5	1,5
10	10	1,5	1,5	1,5	1,5	10	10	1,5	1,5
10	10	10	1,5	1,5	10	10	10	10	1,5
10	10	10	10	2,8	10	10	10	1,8	1,8
10	10	10	1,9	1,9	10	10	10	1,9	10
10	10	2,6	2,6	2,6	10	10	10	10	3,6
10	10	1,5	1,5	10	10	10	10	1,5	1,5
10	10	10	10	1,8	10	10	10	1,8	1,8
10	10	1,5	2,5	2,5	2,5	10	1,5	1,5	1,6
10	10	1,8	10	10	10	10	10	1,8	2,8
10	10	1,5	10	10	10	10	10	1,5	1,5
10	10	10	10	10	10	10	10	10	10
10	10	10	1,9	1,9	10	10	10	10	10
10	10	2,6	2,6	2,6	10	10	1,5	1,5	2,6
10	10	10	1,5	10	10	10	10	1,5	1,5
10	10	1,5	1,5	1,5	1,5	1,5	1,5	1,5	1,5
10	10	1,5	1,5	1,5	1,5	1,5	1,5	1,5	1,5
10	10	1,5	1,5	1,5	1,5	1,5	1,5	1,5	1,5
10	10	10	3,5	3,5	10	10	10	3,5	3,5
10	10	1,8	1,8	2,8	1,8	1,8	10	1,8	1,8
10	10	1,5	1,5	1,5	10	10	10	1,5	1,5
10	10	10	1,9	1,9	10	10	10	1,9	10
10	10	2,6	2,6	2,6	2,6	10	1,5	1,5	2,6
10	10	10	1,5	1,5	10	1,5	2,5	2,5	1,8
10	10	1,5	1,5	1,5	10	10	1,5	1,5	1,5
10	10	2,5	2,6	2,6	10	10	10	10	2,6
10	10	10	1,5	1,5	10	1,8	1,8	1,6	1,7
10	10	10	2,7	2,7	10	10	10	1,5	10
10	10	1,7	1,7	10	10	1,8	1,8	1,6	1,8
10	10	1,8	1,8	1,8	1,8	1,8	1,8	1,5	10
10	10	10	10	1,5	10	10	10	1,5	10
10	10	1,5	1,5	1,5	10	10	10	1,6	1,5
10	10	10	1,6	1,5	10	10	10	1,7	1,5
10	10	10	1,5	1,5	10	10	10	1,5	1,5
10	10	1,5	10	1,5	10	1,5	10	1,5	1,5
10	10	1,5	1,5	1,5	10	10	10	1,5	1,5
10	10	10	10	10	10	10	10	10	10
10	10	10	2,6	2,5	10	10	10	10	2,6
10	10	1,5	1,5	1,5	10	10	1,5	1,5	1,5
10	10	10	10	10	10	10	10	10	2,6

10	10	10	1,8	10	1,8	10	10	10	1,8
10	10	10	2,5	10	10	10	1,5	10	1,5
10	10	10	10	10	10	10	1,5	10	2,5
10	10	10	10	10	10	10	1,5	10	2,5
10	10	10	1,8	1,8	10	10	10	1,5	1,5
10	10	1,5	1,5	1,5	1,5	1,5	1,5	1,5	1,5
10	10	2,5	2,5	2,6	10	10	10	10	2,6
10	10	1,5	1,5	1,5	10	10	10	1,5	1,6
10	10	10	1,8	10	10	10	1,5	10	1,5
10	10	1,5	1,5	1,5	10	10	10	1,5	1,5
10	10	10	10	2,5	10	10	2,8	1,5	2,5
10	10	10	10	2,5	10	10	2,8	1,5	2,5
10	10	1,9	1,9	1,9	10	1,9	10	1,5	1,5
10	10	1,5	2,5	2,5	1,5	10	10	1,5	1,5
10	10	1,5	1,5	1,5	1,5	1,5	10	1,5	1,6
10	10	1,5	1,5	10	10	10	10	1,5	1,5
10	10	1,5	1,6	10	10	10	10	1,5	1,6
10	10	10	10	10	10	10	1,5	1,5	1,6
10	10	10	2,6	10	10	10	10	10	2,5
10	10	10	1,5	10	10	10	1,5	1,5	1,5
10	10	10	2,5	2,5	10	1,5	10	10	10
10	10	1,9	1,9	1,9	10	10	10	1,5	1,5
10	10	1,9	1,9	1,9	10	10	10	1,6	1,6
10	10	10	10	10	10	10	10	1,5	10
10	10	10	10	10	10	10	10	1,5	10
10	10	1,6	10	10	10	10	10	1,5	1,6
10	10	1,7	1,7	1,7	10	10	10	1,5	1,5
10	10	10	1,8	1,8	10	1,8	10	1,5	1,5
10	10	1,9	1,9	1,9	10	10	10	1,5	10
10	10	2,5	2,5	2,5	10	10	10	10	2,5
10	10	10	10	2,9	10	10	10	10	2,5
10	10	1,9	1,9	1,9	1,9	1,9	10	10	10
10	10	10	10	10	10	1,5	1,5	1,5	1,6
10	10	2,5	2,5	2,5	10	10	10	10	2,6
10	10	1,5	1,5	1,5	10	10	10	1,5	1,5
10	10	10	1,9	1,9	1,9	1,9	1,5	1,5	10
10	10	10	1,9	1,9	10	10	10	1,5	10
10	10	10	1,9	1,9	10	1,9	10	10	10
10	10	1,5	1,5	1,5	10	1,5	10	1,5	10
10	10	1,5	1,5	1,5	10	1,5	10	1,5	10
10	10	1,9	1,9	1,9	10	10	10	1,5	10
10	10	10	10	1,5	10	10	10	1,8	2,5
10	10	10	1,8	1,8	10	10	10	1,5	1,5
10	10	1,5	1,5	1,5	10	1,5	10	1,5	1,5
10	10	1,5	1,5	1,5	1,5	1,5	10	1,5	1,5
10	10	10	10	2,9	10	10	10	10	10
10	10	10	1,8	10	10	1,8	10	1,5	1,5

10	10	10	10	10	10	10	1,6	1,5	1,7
10	10	10	1,9	1,9	10	1,9	10	10	10
10	10	1,9	1,9	1,9	1,9	10	10	10	1,9
10	10	10	2,6	2,6	10	10	10	10	2,5
10	10	10	10	2,6	2,6	10	10	10	1,5
10	10	10	1,9	1,9	1,9	10	10	10	10
10	10	10	1,9	10	10	10	10	10	10
10	10	10	2,8	10	10	10	10	10	3,5
10	10	1,5	1,5	10	10	10	10	1,6	1,5
10	10	10	10	10	10	10	10	1,6	1,5
10	10	1,5	1,5	10	10	10	1,5	1,5	1,5
10	10	10	10	10	10	10	10	1,8	1,5
10	10	1,9	1,9	1,9	10	10	10	1,5	1,5
10	10	1,8	1,8	1,8	10	10	10	1,5	10
10	10	1,5	1,5	1,5	1,5	1,5	10	1,5	1,5
10	10	1,9	1,9	10	10	10	1,5	10	10
10	10	1,5	1,5	1,5	1,5	1,5	10	1,5	1,5
10	10	10	1,9	1,9	1,9	10	10	1,5	10
10	10	10	1,9	1,9	10	10	1,5	10	10
10	10	1,6	1,6	1,6	10	10	10	1,6	1,5
10	10	10	1,5	10	10	10	10	1,5	1,5
10	10	10	10	10	10	10	1,5	1,5	1,5
10	10	10	10	10	10	10	1,5	1,5	1,5
10	10	10	10	3,9	10	10	10	10	2,5
10	10	10	10	10	10	10	10	1,5	2,5
10	10	1,5	1,5	1,5	10	1,5	10	1,5	1,5
10	10	2,5	2,5	2,5	10	10	2,5	2,5	2,6
10	10	1,9	1,9	1,9	10	1,9	10	10	10
10	10	10	3,9	10	10	10	10	10	10
10	10	1,8	1,8	1,8	10	10	10	1,6	1,5
10	10	1,9	1,9	10	10	10	10	1,5	1,5
10	10	10	1,6	10	10	10	1,5	1,6	1,5
10	10	1,5	1,5	1,5	1,5	1,5	10	1,5	1,5
10	10	1,5	1,5	1,5	10	10	1,5	1,5	1,5
10	10	10	1,9	1,9	1,9	1,9	10	10	10
10	10	1,9	1,9	1,9	1,9	10	1,5	10	10
10	10	1,5	1,5	10	10	10	1,5	1,5	2,5
10	10	1,8	1,8	1,8	10	1,8	10	2,6	1,5
10	10	1,9	10	1,9	10	10	1,5	10	10
10	10	1,5	1,5	1,5	10	10	1,5	1,5	1,5
10	10	10	10	10	10	10	1,5	1,5	1,5
10	10	10	1,6	1,6	1,6	1,6	10	1,6	1,5
10	10	10	10	10	10	10	10	1,6	1,5
10	10	10	10	10	10	10	10	10	3,5
10	10	10	10	10	10	10	10	1,5	1,5
10	10	10	2,7	2,7	10	10	1,5	2,6	1,5
10	10	10	2,5	2,5	2,5	10	10	10	2,5

10	10	10	2,5	2,5	2,5	10	10	10	2,5
10	10	10	1,9	1,9	1,9	10	10	1,5	10
10	10	1,5	1,5	1,5	10	10	1,5	1,5	1,5
10	10	1,9	1,9	1,9	1,9	10	10	1,5	10
10	10	10	1,9	1,9	1,9	1,9	1,5	1,5	10
10	10	10	1,9	1,9	1,9	10	10	1,5	10
10	10	10	1,9	1,9	1,9	10	10	1,5	10
10	10	10	1,9	1,9	1,9	1,9	1,5	10	10
10	10	10	1,9	1,9	1,9	10	10	1,5	10
10	10	1,9	1,9	1,9	10	10	10	1,5	10
10	10	1,5	1,5	1,5	10	10	1,5	1,5	1,5
10	10	10	1,5	1,5	1,5	1,5	10	1,5	1,5
10	10	10	1,9	10	10	10	10	10	10
10	10	10	10	2,9	10	10	10	10	10
10	10	10	1,9	10	10	10	10	10	1,5
10	10	10	1,5	10	10	10	1,7	1,5	1,7
10	10	2,5	2,5	2,5	10	10	1,5	1,5	1,5
10	10	2,5	2,5	2,5	10	10	2,5	2,5	2,5
10	10	10	1,8	10	10	10	10	10	1,5
10	10	10	10	10	10	10	10	2,5	1,5
10	10	10	2,8	10	10	10	10	10	3,5
10	10	10	1,5	1,5	10	10	10	10	10
10	10	10	1,9	1,9	10	10	10	1,6	1,6
10	10	10	10	10	10	10	1,8	10	2,5
10	10	10	10	10	10	10	1,8	10	2,5
10	10	1,5	1,5	1,5	1,5	1,5	1,5	1,5	1,5
10	10	10	10	1,8	10	10	10	10	1,5
10	10	10	1,5	10	10	10	10	1,8	1,5
10	10	10	1,9	10	10	10	1,5	10	10
10	10	10	2,5	10	10	10	10	10	2,5
10	10	10	1,5	1,5	10	10	1,5	1,5	10
10	10	10	1,8	1,8	10	10	10	10	10
10	10	10	1,9	1,9	10	10	1,5	10	10
10	10	1,5	1,5	10	1,5	10	10	1,5	1,5
10	10	10	2,5	2,5	10	10	1,5	1,5	2,5
10	10	1,5	10	10	10	10	1,5	1,5	1,5
10	10	10	1,5	1,8	10	10	10	10	2,5
10	10	1,9	1,9	1,9	1,9	10	10	1,5	10
10	10	1,6	10	1,6	10	1,6	10	1,6	1,6
10	10	1,9	1,9	1,9	1,9	10	1,5	1,5	1,5
10	10	10	10	10	10	10	10	10	2,5
10	10	10	1,8	1,8	10	10	10	10	2,5
10	10	10	10	10	10	10	10	10	1,5
10	10	10	10	10	10	10	10	10	1,5
10	10	1,9	1,9	10	10	10	10	1,5	1,5
10	10	1,9	1,9	10	10	10	10	1,5	1,5
10	10	10	1,5	1,6	1,6	10	10	10	1,5

10	10	1,9	1,9	10	10	10	1,5	1,5	1,5
10	10	1,6	1,5	10	10	10	10	10	1,5
10	10	1,5	1,5	10	10	10	10	10	1,5
10	10	10	10	10	10	1,5	10	10	1,5
10	10	1,5	1,5	1,5	10	10	10	1,5	1,5
10	10	10	1,9	10	10	10	10	1,5	1,5
10	10	1,5	1,5	10	10	10	10	1,5	1,5
10	10	1,5	1,6	10	1,6	10	1,6	1,6	1,6
10	10	1,6	1,6	10	1,6	1,5	10	10	1,6
10	10	1,6	1,6	10	1,6	1,5	10	10	1,6
10	10	10	1,5	10	10	10	10	10	10
10	10	1,6	1,6	10	10	10	10	10	1,5
10	10	10	1,9	10	10	10	10	1,5	1,5
10	10	10	1,6	1,6	1,5	1,6	10	10	1,6
10	10	10	1,5	1,5	10	10	10	1,5	1,5
10	10	1,9	1,9	10	10	10	10	1,5	1,5
10	10	10	2,5	10	10	10	10	1,5	1,5
10	10	10	1,6	10	10	10	10	10	10
10	10	10	1,6	1,6	10	10	10	1,6	1,6
10	10	1,5	1,5	1,5	10	10	10	1,5	1,5
10	10	10	1,9	10	10	10	10	1,5	1,5
10	10	10	10	10	10	10	10	1,5	1,8
10	10	10	1,5	10	10	10	10	1,5	1,5
10	10	10	10	1,8	10	10	10	1,5	1,5
10	10	1,5	1,5	1,5	1,5	10	10	1,5	1,5
10	10	1,5	1,5	1,6	10	10	10	1,6	1,5
10	10	10	10	10	10	10	10	1,8	1,5
10	10	1,5	1,5	10	10	10	10	1,5	1,5
10	10	10	1,8	10	10	10	10	1,5	1,6
10	10	1,5	1,5	10	10	10	10	1,5	1,5
10	10	1,9	1,9	1,5	10	10	10	1,5	1,5
10	10	10	1,9	10	10	1,5	1,5	1,5	1,5
10	10	10	1,5	10	10	10	10	1,5	1,5
10	10	1,5	1,5	1,5	10	10	10	1,5	1,5
10	10	10	1,8	1,5	10	10	10	1,5	1,8
10	10	10	10	10	10	10	1,5	1,5	1,5
10	10	10	10	10	10	10	10	10	1,8
10	10	10	1,5	10	10	10	10	1,5	1,8
10	10	10	1,8	10	1,8	10	10	1,5	1,5
10	10	10	10	1,8	10	10	10	1,6	1,5
10	10	10	1,5	10	10	10	1,5	1,5	1,5
10	10	10	10	1,5	1,5	1,5	1,5	1,5	1,5
10	10	10	1,5	1,5	10	10	10	10	1,5
10	10	10	1,5	1,5	10	10	10	1,5	1,5
10	10	1,5	1,5	1,5	10	10	10	10	1,5
10	10	1,9	1,9	10	10	10	1,5	1,5	1,5
10	10	10	1,5	1,5	10	10	10	10	1,5

10	10	10	1,8	1,5	10	10	10	10	1,5
10	10	1,5	1,5	1,5	1,5	10	10	1,5	1,5
10	10	1,5	1,5	1,5	10	1,5	10	10	1,5
10	10	10	1,6	1,6	1,6	10	10	10	1,5
10	10	1,9	1,9	10	10	10	10	1,6	1,6
10	10	10	10	10	10	10	1,5	1,5	1,5
10	10	10	1,5	1,5	10	10	10	10	1,5
10	10	10	1,5	1,5	10	10	10	10	1,5
10	10	10	1,5	1,5	1,5	1,5	1,5	1,5	1,5
10	10	10	1,6	1,6	1,5	10	10	10	1,5
10	10	10	1,8	10	10	10	10	1,5	1,5
1,9	1,9	1,9	1,9	10	10	10	10	10	10
10	10	10	1,5	10	10	10	10	1,5	1,5
10	10	10	10	10	10	10	10	10	1,5
10	10	10	10	10	10	10	10	10	1,5
10	10	10	1,5	1,5	10	10	10	10	1,5
10	10	10	1,5	1,5	10	10	10	10	1,5
10	10	10	1,8	10	10	10	10	10	1,5
10	10	1,9	1,9	10	10	10	10	1,6	1,6
10	10	1,5	1,5	1,5	10	10	10	1,5	1,5
10	10	10	1,6	1,6	10	10	1,5	10	1,5
10	10	10	10	1,5	10	10	10	1,5	1,5
10	10	10	1,8	1,8	10	10	10	10	1,5
10	10	1,5	1,5	1,5	10	1,5	10	1,5	1,5
10	10	10	10	10	10	10	10	10	1,5
10	10	10	1,8	1,8	10	10	10	1,5	1,6
10	10	10	1,5	1,5	10	10	10	1,5	1,5
10	10	10	1,8	1,8	10	10	10	10	1,5
10	10	10	1,8	1,8	10	10	10	10	1,5
10	10	10	1,5	1,5	10	10	10	10	1,5
10	10	10	1,8	10	10	10	10	10	10
10	10	10	1,5	10	1,5	10	10	10	1,5
10	10	1,5	10	1,5	10	10	10	10	10
10	10	1,5	10	1,5	10	10	10	1,5	1,5
10	10	10	2,8	2,8	10	10	10	2,5	2,5
10	1,9	1,5	1,5	1,5	1,5	10	10	1,5	1,5
10	10	10	10	10	10	10	10	1,6	1,5
10	10	1,5	1,5	1,5	10	10	10	1,5	1,5
10	10	1,5	1,5	1,5	1,5	10	10	1,5	1,5
10	10	1,5	1,5	1,5	1,5	10	10	1,6	1,5
10	10	2,7	1,6	2,6	10	10	10	10	10
10	10	10	2,5	1,5	10	10	10	10	2,5
10	10	10	1,6	1,5	1,6	10	10	10	2,5
10	10	1,5	1,5	1,5	10	10	10	1,5	1,5
10	10	10	1,7	1,6	1,5	10	10	10	1,6
10	10	10	10	1,5	1,5	1,5	1,5	1,5	1,5
10	10	10	1,5	1,5	10	10	10	10	10

10	10	10	1,5	1,5	10	10	10	1,5	1,5
10	10	10	1,9	10	10	10	10	10	10
10	10	1,5	1,5	1,5	10	10	1,5	1,5	1,5
10	10	10	10	10	10	10	10	1,6	1,6
10	10	10	10	10	10	10	10	1,6	1,6
10	10	10	10	10	10	10	10	1,6	1,6
10	10	10	1,5	10	10	1,5	2,5	2,5	10
10	10	1,5	1,5	1,5	1,5	10	10	10	1,5
10	10	1,5	1,5	1,5	10	10	10	1,5	1,5
10	10	1,9	1,9	10	10	10	1,5	1,5	1,5
10	10	1,9	1,9	10	10	10	1,5	1,5	1,5
10	10	10	10	10	10	10	1,5	1,5	1,5
10	10	10	10	10	10	1,5	10	1,5	10
10	10	1,5	1,5	1,5	10	10	10	10	10
10	10	10	10	10	10	1,5	10	1,5	10
10	10	10	10	1,5	10	10	1,8	1,8	1,8
10	10	1,9	1,9	1,9	10	10	10	1,6	1,6
10	10	1,5	1,5	1,5	10	10	1,5	1,5	1,5
10	10	1,5	1,5	1,5	1,5	10	10	1,6	1,6
10	10	1,9	1,9	1,9	10	10	10	1,6	1,6
10	10	10	10	1,5	10	10	10	1,5	1,5
10	10	10	10	1,5	10	10	1,5	1,5	10
10	10	1,9	1,9	10	10	10	10	1,9	1,5
10	10	10	1,5	1,5	10	10	10	10	1,5
10	10	10	1,5	1,5	10	10	1,5	1,5	1,5
10	10	1,9	1,9	10	10	10	10	1,6	1,6
10	10	10	1,5	1,6	10	10	10	1,6	1,6
10	10	1,9	1,9	10	10	10	10	1,6	1,6
10	10	1,9	1,9	10	10	10	10	1,5	1,6
10	10	1,9	1,9	10	10	10	10	1,5	1,6
10	10	1,8	1,6	1,5	10	10	10	1,6	1,6
10	10	10	1,5	1,5	1,5	1,5	1,5	1,5	1,5
10	10	1,5	1,5	1,5	10	10	10	1,5	1,5
10	10	10	1,5	1,5	10	10	10	1,6	1,5
10	10	10	1,9	1,5	10	10	10	10	10
10	10	10	1,5	10	10	10	10	1,5	1,5
10	10	1,9	1,9	1,9	10	10	10	1,5	1,6
10	10	1,9	1,9	1,9	10	10	10	1,5	1,6
10	10	10	10	10	10	10	10	1,5	1,5
10	10	1,5	1,5	1,5	1,5	1,5	10	1,5	1,5
10	10	1,5	1,5	1,5	10	1,5	10	1,5	1,5
10	10	10	1,5	1,6	10	10	10	1,6	1,5
10	1,9	10	10	10	10	10	10	1,9	1,9
10	10	1,9	1,9	10	10	10	10	1,5	1,6
10	10	1,5	1,5	10	10	10	10	10	1,5
10	10	1,9	1,9	10	10	10	10	10	1,5
10	10	1,5	1,5	1,5	10	1,5	1,5	1,6	1,5

10	10	1,5	1,5	1,5	1,5	1,5	1,5	1,5	10
10	10	1,5	1,5	1,5	1,5	1,5	1,5	1,5	1,5
10	10	1,5	1,5	1,5	10	10	10	10	1,5
10	10	1,5	1,5	1,5	10	10	10	10	1,5
10	10	1,5	1,5	10	10	10	10	10	10
10	10	10	10	10	10	10	10	1,5	1,5
10	10	10	10	1,8	10	10	10	10	10
10	10	1,6	1,6	1,5	10	10	10	1,6	1,6
10	10	1,5	1,5	1,5	10	10	10	1,6	1,6
10	10	1,8	10	10	10	10	10	10	1,5
10	10	1,5	1,5	1,5	1,5	1,5	10	1,5	1,5
10	10	10	10	10	10	10	10	10	10
10	10	10	2,5	10	10	10	10	10	1,5
10	10	10	10	10	10	10	10	10	10
10	10	10	1,5	10	10	10	10	1,8	1,5
10	10	10	1,5	10	10	10	10	10	1,5
10	10	1,5	1,5	1,5	10	10	10	10	10
10	10	1,9	1,9	1,8	10	10	1,5	1,5	1,5
10	10	10	1,5	10	10	10	10	1,6	1,5
10	10	10	1,5	10	10	10	10	10	1,5
10	10	1,8	1,8	1,9	1,9	1,9	1,9	1,9	1,9
10	10	1,5	1,5	1,5	10	10	10	1,5	1,5
10	10	10	10	10	10	10	10	10	10
10	10	1,9	1,9	10	10	10	1,5	1,5	1,5
10	10	10	1,5	10	10	10	10	1,5	1,5
10	10	10	10	10	10	10	10	10	1,5
10	10	10	1,5	1,5	10	1,5	10	10	1,5
10	10	1,9	1,9	10	10	10	10	1,5	1,5
10	1,9	10	1,5	1,5	1,5	10	10	1,5	1,5
10	10	1,5	1,5	1,5	10	1,5	10	1,5	1,5
10	10	10	1,5	10	10	10	10	1,5	1,5
10	10	1,5	1,5	10	1,5	10	10	1,8	1,5
10	10	1,9	1,9	10	10	10	1,5	1,5	1,5
10	10	10	10	1,5	10	10	10	10	1,5
10	10	1,9	1,9	10	10	10	1,5	1,5	1,5
10	10	1,5	1,5	1,5	1,5	10	10	10	1,5
1,9	10	10	10	10	10	10	1,5	1,5	1,5
10	10	10	10	10	10	10	10	10	10
10	10	10	1,8	10	10	10	10	1,8	10
10	10	1,5	1,5	1,5	1,5	1,5	10	1,5	1,5
10	10	10	10	10	10	10	2,6	3,7	1,5
10	10	10	10	10	10	10	2,6	3,7	1,5
10	10	10	10	1,5	10	10	10	10	10
10	10	1,5	1,5	1,5	1,5	1,5	1,5	1,5	1,5
10	10	10	1,8	10	10	10	10	1,5	1,5
10	10	1,5	1,5	10	10	10	10	2,5	2,5
10	10	10	10	10	10	10	2,6	3,7	1,5

10	10	10	1,5	10	10	10	10	1,5	1,5
10	10	1,9	1,9	1,5	10	10	2,5	2,5	2,5
10	10	10	2,8	10	10	10	10	1,8	2,5
10	10	1,9	1,9	10	10	10	1,5	1,6	1,5
10	10	10	10	1,9	10	10	10	10	1,9
10	10	10	10	1,5	10	10	10	10	1,5
10	10	1,9	1,9	1,9	10	10	1,5	1,5	1,5
10	10	1,5	1,5	1,5	10	10	10	1,5	1,5
10	10	10	1,5	10	10	10	10	1,6	1,5
10	10	1,5	1,5	1,5	1,5	1,5	10	1,5	1,5
10	10	1,9	1,9	1,9	10	10	10	1,6	1,5
10	10	1,5	1,5	1,5	1,5	1,5	10	1,5	1,5
10	10	10	10	1,8	10	10	10	1,6	1,5
10	10	10	10	1,8	10	10	10	1,6	1,5
10	10	10	2,5	10	10	10	10	1,5	1,5
10	10	10	10	10	10	10	10	10	10
10	10	10	1,5	1,5	1,5	1,5	10	1,5	2,5
10	10	10	1,5	10	1,5	1,5	10	1,5	2,5
10	10	10	10	10	1,5	10	10	10	1,5
10	10	10	10	10	1,5	10	10	10	1,5
10	10	1,9	1,9	10	10	10	1,5	1,5	1,5
10	10	10	10	10	10	10	10	10	10
10	10	10	10	10	10	10	10	10	1,5
10	10	10	1,9	10	10	10	10	10	10
10	10	10	10	1,8	10	10	10	10	10
10	10	1,9	1,9	10	10	10	10	10	10
10	10	10	10	1,8	10	10	10	10	10
10	10	10	1,5	1,6	10	10	10	1,5	1,5
10	10	10	1,9	10	10	10	1,5	1,5	1,5
10	10	1,5	1,5	1,5	1,5	10	10	1,5	1,5
10	10	10	2,5	2,5	10	10	1,5	10	10
10	10	1,5	1,5	10	10	10	10	1,5	1,5
10	10	1,9	1,9	10	10	10	10	1,5	1,5
10	10	1,5	1,5	1,5	10	10	1,5	1,5	1,5
10	10	10	1,6	10	10	10	1,6	1,6	10
10	10	10	1,5	1,5	10	10	10	1,6	1,5
10	10	10	1,5	1,5	10	10	10	1,5	1,5
10	10	10	1,6	1,5	1,6	10	10	1,6	1,6
10	10	1,9	1,9	10	10	10	1,5	1,5	1,5
10	10	10	1,5	1,5	10	10	10	10	1,5
10	10	10	1,5	1,5	10	10	1,5	1,5	1,5
10	10	1,8	1,8	10	10	10	10	10	1,5
10	10	1,9	1,9	10	10	10	10	10	1,5
10	10	1,5	1,5	1,5	1,5	10	10	1,5	1,5
10	10	10	10	10	10	10	10	10	10
10	10	10	1,5	10	10	10	10	1,5	1,6

mashed pc	potato sala	boiled whi	boiled yell	boiled orai	boiled carr	stewed cai	carrotsalac	boiled butt	butternut
10	1,6	10	10	10	10	1,6	10	10	1,6
10	1,6	10	10	10	10	1,6	10	10	1,6
1,5	1,5	1,5	1,5	1,5	10	1,5	1,5	1,5	1,5
1,5	1,5	10	2,8	2,8	10	10	10	1,8	1,5
1,5	1,5	1,5	1,5	10	1,5	1,5	1,5	1,5	1,5
10	10	10	10	10	10	10	10	1,5	10
10	10	1,8	10	10	10	10	10	1,5	1,5
1,5	1,5	10	1,8	10	10	10	1,5	10	1,5
2,5	2,5	1,6	1,6	1,6	10	10	10	2,6	2,6
10	1,5	1,8	1,8	1,8	10	10	10	1,5	10
2,6	2,6	10	10	10	10	1,5	10	1,4	1,6
10	2,5	1,5	1,5	1,5	10	10	10	2,5	2,5
2,5	2,5	1,5	1,5	1,5	10	10	10	1,5	2,6
10	1,5	10	10	10	1,5	10	10	1,5	10
10	1,5	1,8	1,8	1,8	10	1,5	1,5	10	1,5
10	1,6	10	10	1,8	10	10	10	1,7	1,7
10	1,6	10	10	1,8	10	10	10	1,7	1,7
2,5	2,5	1,5	1,5	1,5	10	10	10	2,5	2,6
1,5	1,5	1,5	1,5	1,5	10	10	1,5	1,5	2,6
2,5	2,5	1,5	1,5	1,5	10	10	1,5	1,5	2,5
1,5	1,5	1,5	1,5	1,5	10	10	10	10	1,5
2,5	2,5	1,5	1,5	1,5	10	10	10	10	2,5
2,5	2,5	1,5	1,5	1,5	10	10	10	10	2,5
1,8	1,5	1,5	1,5	1,5	1,8	10	1,5	1,5	2,5
2,5	2,5	1,5	1,5	1,5	10	1,5	1,5	2,5	2,6
2,5	2,5	1,5	1,5	1,5	10	10	10	2,5	2,6
10	1,5	10	10	10	10	10	10	10	10
1,5	1,5	1,9	1,9	1,9	10	10	1,5	1,5	1,5
1,5	1,5	1,5	1,5	1,5	1,5	1,5	1,5	1,5	1,5
1,6	1,8	1,7	1,7	1,7	10	10	1,8	1,7	2,7
10	10	1,5	1,5	1,5	1,5	10	10	1,5	1,5
1,5	2,5	1,8	1,8	1,8	10	10	10	1,5	2,9
10	2,6	1,6	1,6	1,6	10	10	10	2,5	2,5
1,8	10	10	10	10	1,8	10	1,8	10	1,5
10	1,5	10	1,8	10	10	1,5	1,5	1,5	1,5
10	1,8	1,8	1,8	1,8	10	10	10	1,5	1,5
10	1,5	1,7	10	10	10	1,5	1,7	10	2,6
10	10	10	10	10	10	10	10	1,4	10
10	10	1,5	1,5	1,5	10	10	10	10	10
10	1,8	1,8	1,8	1,8	10	1,8	1,8	1,8	1,8
1,6	1,5	1,6	1,6	1,6	10	1,5	1,6	1,6	1,6
1,5	1,5	1,5	1,5	1,5	1,5	1,5	1,5	1,5	1,5
10	1,5	2,8	2,8	2,8	10	10	10	1,8	10
10	10	1,5	1,5	1,5	10	10	1,5	1,9	10
10	1,5	1,8	1,8	1,8	10	1,5	1,8	10	1,5
10	1,8	10	10	10	10	10	1,8	10	1,5

10	1,8	10	1,9	10	1,5	10	1,8	1,5	10
10	3,8	2,8	2,8	2,8	10	1,5	1,5	10	1,5
1,5	1,5	1,5	1,5	1,5	10	10	10	1,9	1,9
1,5	1,5	1,5	1,5	1,5	1,5	1,5	1,5	1,5	1,5
10	1,5	10	1,9	10	10	1,6	1,7	1,6	1,5
10	10	1,8	1,8	1,8	10	10	10	1,8	10
10	1,5	1,8	1,8	1,8	1,5	1,5	1,5	10	1,5
10	1,5	10	10	10	10	10	10	1,8	1,8
10	1,5	10	10	10	10	10	10	10	1,5
10	1,8	2,8	2,8	2,8	10	10	10	1,8	1,5
10	1,5	10	10	10	10	1,5	10	10	1,5
10	1,8	10	10	10	10	1,6	10	10	1,5
10	1,5	1,8	1,8	1,8	10	10	10	1,5	1,5
1,5	1,5	1,5	1,5	1,5	1,5	2,5	2,5	1,5	1,5
10	1,5	2,9	2,9	2,9	10	10	1,5	10	1,5
10	1,8	1,9	10	10	10	10	1,8	1,5	10
10	1,9	2,8	2,8	2,8	10	10	10	1,5	1,5
1,7	1,8	1,8	1,8	1,8	10	2,7	1,6	2,7	1,7
10	1,5	10	1,8	10	10	1,5	1,5	1,5	1,5
10	1,5	1,8	1,8	1,8	10	10	1,5	10	10
2,5	2,6	1,8	1,8	1,8	10	10	10	2,6	2,6
2,5	2,5	1,5	1,5	1,5	10	10	10	1,5	2,5
2,5	2,5	1,5	1,5	1,5	10	10	10	1,5	2,5
10	10	10	10	10	10	1,8	1,5	1,5	1,5
10	10	10	10	10	10	1,8	1,5	1,5	1,5
10	1,8	1,8	1,8	1,8	10	10	1,8	10	1,5
10	10	1,5	10	10	10	10	10	1,5	1,5
10	1,5	1,9	10	1,9	10	10	1,5	1,5	1,5
1,6	1,6	1,6	1,6	1,6	10	10	1,6	10	2,6
10	10	10	10	10	10	10	10	1,5	1,5
10	1,5	10	1,9	10	10	10	10	10	2,5
10	1,5	10	1,9	10	10	10	10	10	2,5
10	10	1,5	1,5	1,5	10	10	10	10	10
1,5	1,5	1,5	1,5	1,5	10	10	1,8	2,7	1,7
10	1,5	10	10	1,5	10	1,5	1,5	1,5	10
10	2,5	1,5	1,5	1,5	10	10	10	2,5	2,6
10	10	1,5	1,5	1,5	10	10	10	1,5	2,6
10	1,5	10	10	10	10	10	1,5	10	1,5
10	1,8	2,8	2,8	2,8	10	1,8	10	2,8	10
10	1,5	1,5	1,5	1,5	10	10	1,5	2,6	2,6
10	1,5	1,5	1,5	10	10	1,5	1,5	1,5	1,5
10	1,5	1,8	1,8	1,8	10	1,5	1,5	1,8	1,5
10	1,5	1,8	1,8	10	10	1,8	1,5	1,8	1,5
10	1,6	1,8	1,8	10	10	1,5	1,5	10	1,5
1,5	1,8	1,8	1,8	1,9	1,9	1,5	1,5	10	1,5
10	1,5	1,8	1,8	10	10	1,5	1,5	10	1,5
2,5	10	2,5	2,5	2,5	10	10	10	2,5	2,5

10	1,8	1,9	10	10	10	10	1,8	10	1,8
10	1,5	10	10	10	10	10	10	10	10
1,6	1,5	1,5	1,5	1,5	10	10	1,6	10	1,7
10	1,5	1,8	1,8	1,8	10	1,5	10	10	1,5
10	1,5	1,5	1,5	1,5	10	10	1,5	1,5	1,5
2,5	2,5	2,6	2,6	2,6	10	1,5	10	10	10
2,5	2,5	2,5	2,5	2,5	1,5	1,5	1,5	2,6	2,6
10	10	1,8	1,8	1,8	10	10	1,5	1,9	1,5
10	10	1,8	1,8	1,8	10	10	1,5	1,8	1,5
10	1,5	2,9	2,9	2,9	1,5	1,5	1,5	3,5	3,5
10	1,8	1,8	1,8	1,8	10	10	10	10	2,8
10	1,5	10	1,5	10	1,8	10	10	1,9	10
10	1,5	10	10	10	10	1,7	1,5	1,5	1,5
10	10	10	10	10	10	10	10	1,5	1,5
10	1,8	1,8	1,8	1,8	10	10	10	10	2,8
2,6	1,5	1,5	1,5	1,5	10	10	1,5	2,5	2,5
10	1,8	10	10	10	1,8	1,8	1,8	1,8	1,8
10	10	1,5	10	10	10	10	10	1,5	10
10	10	1,5	1,5	1,5	10	1,5	10	10	1,5
10	1,8	1,9	10	10	1,8	10	1,8	1,9	1,9
2,6	2,5	1,5	1,5	1,5	1,5	1,5	1,5	1,5	1,5
10	1,5	1,8	10	10	10	10	1,5	10	1,5
1,5	1,5	1,5	1,5	1,5	1,5	1,5	1,5	1,5	1,5
1,5	1,5	1,5	1,5	1,5	1,5	1,5	1,5	1,5	1,5
1,5	1,5	1,5	1,5	1,5	1,5	1,5	1,5	1,5	1,5
10	2,6	1,9	1,9	1,9	10	10	10	10	2,5
10	1,8	1,8	1,8	1,8	2,8	2,8	10	1,8	1,8
1,5	1,5	1,5	1,5	1,5	10	10	1,5	1,5	1,5
10	1,5	10	1,5	10	1,8	10	1,8	1,9	10
2,6	1,5	1,5	1,5	1,5	10	1,6	10	2,5	2,5
10	1,9	2,9	2,9	2,9	1,9	1,9	10	10	2,9
1,5	1,5	10	10	10	1,5	1,5	1,5	1,6	1,6
2,6	2,6	1,5	1,5	1,5	10	10	10	1,5	1,5
1,6	1,7	1,8	1,8	1,8	10	1,7	1,8	1,5	1,5
1,6	1,5	1,8	1,8	1,8	10	1,7	1,7	10	1,7
1,8	1,8	10	10	10	10	1,6	1,8	10	1,5
1,6	1,5	1,7	10	10	1,7	1,7	1,7	1,8	1,6
10	1,5	10	10	10	10	10	1,5	10	1,5
1,6	1,8	1,8	1,8	1,8	1,8	1,8	1,8	1,5	1,5
2,8	1,8	10	2,7	2,7	10	10	1,5	1,8	1,5
10	1,5	10	10	1,8	10	1,5	1,5	10	1,5
1,5	1,5	1,5	1,5	1,5	10	1,5	1,5	10	1,5
10	1,5	10	1,8	10	10	1,8	10	1,8	1,5
10	10	1,5	1,5	1,5	10	1,5	1,5	1,5	1,5
2,5	2,5	1,5	1,5	1,5	10	10	2,5	2,5	2,6
1,5	1,5	1,5	1,5	1,5	10	1,5	1,5	1,5	1,5
10	10	1,5	1,5	1,5	10	10	2,5	2,5	2,6

10	10	1,9	10	10	10	10	10	1,5	1,5
10	2,5	1,9	1,9	1,9	1,5	10	1,5	10	1,5
10	2,5	1,5	1,5	1,5	10	1,5	1,5	10	1,5
10	2,5	1,5	1,5	1,5	10	1,5	1,5	10	1,5
1,8	10	2,8	10	10	1,5	10	1,5	1,5	1,5
1,5	1,5	1,5	1,5	1,5	10	1,5	1,5	1,5	1,5
10	2,6	1,5	1,5	1,5	10	10	10	2,5	2,5
1,5	1,5	1,5	1,5	1,5	10	1,5	1,5	1,5	1,5
10	1,5	10	10	1,9	10	10	10	10	1,5
1,5	1,5	1,5	1,5	10	10	1,5	1,5	1,5	1,5
10	10	10	10	1,8	10	10	1,5	1,5	10
10	10	10	10	1,8	10	10	1,5	1,5	10
10	1,5	1,9	10	10	10	10	10	1,5	1,5
10	10	10	10	10	10	1,5	1,5	1,5	1,5
1,5	10	10	1,5	10	10	1,5	1,5	1,5	1,5
10	1,5	1,6	1,6	1,6	10	1,5	10	1,5	1,5
10	1,5	1,5	1,5	1,5	10	1,5	1,5	1,5	1,5
10	1,5	10	10	10	1,5	1,5	1,5	1,5	1,5
10	10	2,6	10	10	10	10	10	2,5	1,5
1,6	1,5	1,5	1,5	1,5	10	1,5	1,5	1,5	1,5
1,5	10	10	10	10	10	10	1,5	10	1,5
10	1,5	10	10	10	10	1,8	1,5	10	1,5
10	1,5	10	10	10	10	1,8	1,5	10	1,5
10	1,5	1,8	10	10	10	1,5	1,5	10	1,5
10	1,5	1,8	10	10	10	1,5	1,5	10	1,5
1,6	10	1,5	1,5	1,5	10	10	1,5	10	10
1,5	1,5	1,5	1,5	1,5	10	1,5	1,5	1,5	1,5
10	1,8	1,8	1,8	1,8	10	1,5	10	1,8	1,5
10	1,8	1,8	10	10	10	10	1,8	10	1,5
10	2,5	1,5	1,5	1,5	1,5	10	1,5	10	1,5
10	2,5	1,9	1,9	1,9	10	10	10	10	1,5
10	1,5	10	1,9	10	10	10	10	1,5	10
1,6	1,6	1,5	1,5	1,5	10	10	1,5	10	1,5
10	2,6	1,5	1,5	1,5	1,5	10	1,5	2,5	2,5
1,5	1,5	1,5	1,5	1,5	1,5	1,5	1,5	1,5	1,5
1,5	10	1,9	10	10	10	10	1,5	1,5	1,5
1,5	10	1,9	10	10	10	10	10	1,5	1,5
10	1,5	1,5	10	10	10	10	1,5	1,5	1,5
1,5	1,5	1,5	1,5	1,5	10	1,5	1,5	1,5	1,5
1,5	1,5	1,5	1,5	1,5	10	1,5	1,5	1,5	1,5
1,5	10	1,9	10	10	10	10	1,5	1,5	1,5
10	10	10	10	10	1,5	1,5	1,5	1,5	1,5
1,5	1,8	1,8	1,8	1,8	10	1,5	10	10	1,5
10	1,5	1,5	1,5	1,5	1,5	1,5	1,5	1,5	1,5
1,5	1,5	1,5	1,5	1,5	1,5	1,5	1,5	1,5	1,5
10	1,5	2,9	2,9	2,9	10	10	1,5	10	1,5
10	1,8	1,8	1,8	1,8	10	10	10	10	1,5

10	1,7	1,9	1,9	1,9	10	1,6	10	1,6	1,6
10	1,8	1,8	10	10	10	10	1,8	10	1,5
1,5	1,5	10	10	10	10	10	1,5	1,5	1,5
10	2,5	1,5	1,5	1,5	10	10	10	2,5	2,5
10	2,5	1,5	1,5	1,5	10	10	10	2,5	2,5
10	1,5	1,9	10	10	10	10	1,5	1,5	10
10	1,5	1,9	10	10	10	10	1,5	10	1,5
10	2,5	1,9	1,9	1,9	10	10	1,5	10	1,5
10	10	1,5	1,5	1,5	10	10	10	10	1,5
10	1,5	2,8	2,8	2,8	10	10	10	10	10
10	1,5	1,5	1,5	10	10	10	1,5	1,5	1,5
10	10	10	1,8	10	10	1,5	1,5	10	1,5
10	1,5	1,5	10	10	10	10	1,5	1,5	10
1,5	1,9	10	10	10	10	10	1,5	1,5	10
1,5	1,5	1,5	1,5	1,5	10	10	1,5	1,5	1,5
10	1,5	1,9	10	10	10	10	1,5	1,9	1,9
1,5	1,5	1,5	1,5	1,5	10	1,5	1,5	10	1,5
10	1,5	10	10	1,9	10	10	1,5	1,5	1,5
10	1,5	10	10	10	10	10	1,5	1,5	10
1,6	1,8	1,8	1,8	1,8	10	10	10	1,5	1,6
1,5	1,5	1,5	10	10	10	1,5	1,5	10	1,5
1,5	1,5	10	10	10	1,5	1,5	1,5	1,5	1,5
1,5	1,5	10	10	10	1,5	1,5	1,5	1,5	1,5
10	1,5	10	1,9	10	10	10	10	10	10
10	10	10	1,5	1,5	10	2,5	10	10	1,5
1,5	1,5	1,5	1,5	1,5	1,5	1,5	1,5	1,5	1,5
2,5	2,5	1,5	1,5	1,5	10	10	10	1,5	1,5
10	1,5	10	1,9	10	10	10	1,5	10	1,5
10	1,5	1,9	1,9	1,9	10	10	10	10	1,5
10	1,8	10	10	10	10	10	10	1,8	1,8
10	10	1,8	10	10	10	1,5	10	1,5	1,5
10	1,5	1,5	1,5	1,5	10	1,5	1,5	10	1,6
10	10	1,5	1,5	1,5	10	1,5	1,5	1,5	1,5
10	1,5	1,8	1,8	1,8	1,5	1,5	1,5	1,5	1,5
10	1,5	1,5	10	10	10	10	1,5	1,5	1,5
10	1,5	10	10	10	10	10	1,5	10	1,5
10	1,5	1,5	1,5	1,5	1,5	1,5	1,5	10	10
10	1,9	10	10	10	10	10	10	1,8	1,5
10	1,5	10	1,9	10	10	10	1,5	1,5	10
1,5	1,5	1,5	1,5	1,5	10	1,5	1,5	1,5	1,5
10	1,5	1,8	1,8	1,8	10	1,8	1,5	10	1,5
1,5	1,6	1,8	1,8	1,8	10	1,6	1,5	1,6	1,6
10	10	1,8	1,8	1,8	10	10	10	10	1,5
10	10	2,8	10	10	10	10	10	10	2,5
10	10	1,8	1,8	1,8	10	10	10	10	1,5
1,5	1,5	1,8	10	10	10	1,5	1,8	10	1,6
2,5	2,5	1,5	1,5	1,5	1,5	10	10	10	10

2,5	2,5	1,5	1,5	1,5	1,5	10	10	10	10
10	1,5	10	1,9	10	10	10	1,5	1,5	1,5
10	1,5	1,8	1,8	10	10	1,5	1,5	1,8	1,5
10	1,5	10	1,9	10	10	10	10	1,5	1,5
10	1,5	10	10	10	1,5	10	1,5	1,5	1,5
10	1,5	10	1,9	10	10	10	10	1,5	10
10	1,5	10	1,8	10	10	10	1,5	1,5	10
10	1,5	10	1,9	10	10	10	1,5	1,5	1,5
10	1,5	10	1,5	10	10	10	10	1,5	1,5
10	1,5	10	10	10	10	10	1,5	1,5	1,5
1,5	1,5	1,5	1,5	1,5	10	1,5	1,5	1,5	1,5
1,5	1,5	1,5	1,5	1,5	10	1,5	1,5	10	1,5
10	2,5	10	10	10	10	10	2,5	10	10
2,5	10	10	10	10	10	10	2,5	10	2,5
10	1,5	1,9	1,9	1,9	10	10	1,5	10	1,5
1,6	1,8	1,8	1,8	1,8	1,7	1,7	1,7	1,6	1,5
1,5	1,5	1,5	1,5	1,5	1,5	10	1,5	1,5	2,5
1,5	1,5	1,5	1,5	1,5	1,5	10	1,5	1,5	2,5
10	1,5	1,9	1,9	1,9	10	10	1,5	10	1,5
10	1,8	1,8	1,8	1,8	10	1,5	10	1,8	1,5
10	10	1,9	1,9	1,9	10	10	1,5	10	1,8
10	1,5	1,9	1,9	1,9	10	10	1,5	1,5	10
10	1,5	1,8	1,8	1,8	10	10	10	1,5	1,5
10	1,5	1,9	1,9	1,9	10	10	1,5	10	1,5
10	1,5	1,9	1,9	1,9	10	10	1,5	10	1,5
1,5	1,5	1,5	1,5	1,5	10	1,5	1,5	10	1,5
10	1,5	1,9	1,9	1,9	10	10	1,5	10	1,5
10	10	1,8	1,8	1,8	10	1,5	1,5	10	1,5
10	1,5	1,9	1,9	1,9	10	10	1,5	10	1,5
10	1,5	1,9	1,9	1,9	10	10	1,5	10	1,5
1,5	1,5	1,5	1,5	1,5	1,5	10	1,5	1,6	1,5
10	1,5	1,9	1,9	1,9	10	10	1,5	10	1,5
10	1,5	10	1,5	10	10	10	1,5	1,5	1,5
10	1,5	1,5	1,5	1,5	10	1,5	1,5	1,9	1,5
1,5	1,5	1,5	1,5	1,5	10	10	2,5	1,5	2,5
1,5	1,5	1,5	1,6	1,5	10	1,5	1,5	1,6	1,6
10	1,5	1,9	1,9	1,9	10	10	1,8	10	1,8
10	1,5	10	1,9	10	10	10	1,5	1,5	1,5
1,6	1,7	10	10	10	10	1,6	10	1,5	1,5
10	1,5	10	10	1,8	10	1,5	1,5	1,8	1,5
10	1,5	1,9	1,9	1,9	10	10	1,5	10	1,5
10	1,5	1,8	1,8	1,8	10	10	1,5	10	1,5
1,5	1,5	1,8	1,8	1,8	10	10	1,5	10	1,5
1,5	1,5	1,8	1,8	1,8	10	10	1,5	10	1,5
10	10	1,9	1,9	1,9	10	10	1,5	1,5	1,5
10	1,5	1,9	1,9	1,9	10	10	1,5	1,5	1,5
1,6	10	10	10	10	10	10	10	1,5	1,5

1,5	1,5	1,9	1,9	1,9	1,5	1,5	1,5	1,5	1,5
10	1,5	10	10	10	10	10	10	1,5	1,5
1,5	1,5	1,8	1,8	1,8	10	10	10	10	1,5
1,5	1,5	1,8	1,8	1,8	10	1,6	1,5	1,5	1,5
1,5	1,8	1,8	1,8	1,5	1,5	1,5	1,5	1,5	1,5
10	10	1,8	1,8	1,8	10	1,5	10	1,5	1,5
1,5	1,8	1,8	1,8	10	10	10	10	10	1,5
1,6	1,6	10	10	10	1,6	10	10	1,6	1,6
10	1,5	10	10	10	10	10	10	1,5	1,6
10	1,5	10	10	10	10	10	10	1,5	1,6
1,5	1,5	1,5	10	10	10	10	1,6	10	1,5
10	1,5	1,8	1,8	1,8	10	10	10	10	1,5
10	10	1,8	1,8	1,8	10	1,5	10	1,8	1,8
1,6	1,6	10	10	10	10	10	10	10	1,5
1,5	1,5	1,8	1,8	1,8	10	1,5	1,5	1,8	1,5
1,5	1,5	1,9	1,9	1,9	10	10	1,5	1,5	1,5
1,5	1,5	1,8	1,8	1,8	10	1,5	1,5	10	1,5
1,5	1,5	1,6	10	10	10	10	1,5	10	1,5
10	1,6	10	1,6	10	10	10	10	1,5	1,6
1,5	1,5	1,5	1,5	1,5	10	1,5	1,5	10	10
10	1,5	1,5	10	10	10	10	10	1,5	1,5
1,8	1,5	10	10	10	10	10	10	10	1,5
1,8	1,8	1,8	1,8	1,8	10	10	10	1,8	1,5
10	1,5	1,8	1,8	1,8	10	10	10	10	1,8
1,5	1,5	1,5	1,5	1,5	1,5	1,5	1,5	1,5	1,5
1,5	1,5	1,5	1,5	1,5	10	10	1,5	1,5	1,5
10	1,5	10	10	10	10	10	10	1,8	1,5
10	1,5	1,5	1,5	1,5	10	10	1,5	1,5	1,5
1,8	1,8	1,8	1,8	10	10	10	10	10	1,5
1,5	1,5	1,5	1,5	1,5	10	10	1,5	1,5	1,5
1,5	1,5	1,5	1,5	1,5	10	10	10	1,5	1,5
1,5	1,5	1,5	1,5	1,5	10	10	10	10	1,5
10	1,8	1,5	10	10	1,5	1,5	1,5	1,5	1,5
1,5	1,5	1,5	1,5	1,5	10	10	1,5	1,5	1,5
1,8	1,5	1,8	1,8	1,8	10	1,5	10	1,5	1,5
1,5	1,8	1,8	1,8	1,8	1,5	1,5	1,5	1,5	1,5
10	1,8	1,9	1,9	1,9	10	10	1,8	1,5	10
10	1,5	10	1,8	10	10	10	10	10	1,5
1,5	1,5	1,8	1,8	1,8	10	1,5	10	10	1,5
1,5	1,5	0,8	1,8	1,8	10	10	10	1,8	1,5
1,5	1,5	1,5	1,5	1,5	1,6	1,6	1,6	1,9	1,5
1,5	1,5	1,5	1,5	1,5	10	10	10	1,5	1,5
10	1,5	1,9	1,9	1,9	10	10	10	10	1,5
1,5	1,5	1,5	1,5	1,5	10	10	1,5	10	1,5
1,5	10	1,5	1,5	1,5	10	10	1,5	1,5	1,5
10	1,5	1,9	1,9	1,9	10	10	1,5	1,5	1,5
1,5	1,5	1,8	1,8	1,8	10	1,5	1,5	10	1,5

1,5	1,5	1,8	1,8	1,8	10	1,5	1,5	10	1,5
1,5	1,5	1,5	1,5	1,5	10	1,5	1,5	1,5	1,5
1,5	1,5	1,5	1,5	10	10	10	10	10	10
1,6	10	10	10	10	10	10	10	10	10
10	1,5	1,9	1,9	1,9	10	10	1,5	1,5	1,5
1,5	1,8	1,5	1,5	1,5	1,8	1,8	1,8	10	1,5
1,5	1,5	1,8	1,8	1,8	10	10	10	1,8	1,5
1,5	1,5	1,8	1,8	1,8	10	1,5	1,5	1,8	1,6
1,5	1,5	1,5	1,5	1,5	1,8	1,8	1,8	10	1,5
10	2,6	10	10	10	10	10	10	10	10
10	1,5	10	1,8	10	10	10	10	10	1,5
1,8	1,5	1,5	1,5	1,5	1,8	1,8	1,8	10	1,5
1,5	1,5	1,8	1,8	1,8	10	1,5	1,5	10	1,5
1,5	1,9	1,8	1,8	1,8	10	10	1,5	10	1,5
1,5	1,5	1,8	1,8	1,8	10	10	1,5	10	1,5
10	10	1,8	1,8	1,8	10	10	10	1,8	1,5
10	10	1,8	1,8	1,8	10	10	10	1,8	1,5
1,5	1,5	1,8	1,8	1,8	10	10	10	10	1,5
10	1,5	1,9	1,9	1,9	10	10	1,5	1,5	1,5
1,5	1,5	1,5	1,5	1,5	10	10	1,5	1,5	1,5
1,6	1,6	10	10	10	10	10	10	10	1,6
10	1,5	1,8	1,8	1,8	10	10	10	1,5	1,5
1,5	1,5	1,8	1,8	1,8	10	10	10	1,8	1,5
10	1,8	1,8	1,8	1,5	1,5	1,5	1,5	1,5	1,5
1,5	1,5	1,8	1,8	1,8	1,8	1,8	1,5	10	1,5
10	1,5	1,8	1,8	1,8	10	10	10	1,8	1,5
1,5	1,5	1,8	1,8	1,8	10	1,5	1,5	10	1,5
10	1,5	1,8	1,8	1,8	10	10	10	1,8	1,5
10	1,5	1,8	1,8	1,8	10	1,5	1,5	10	1,5
1,5	1,5	1,8	1,8	1,8	10	1,5	10	10	1,5
10	1,5	1,8	10	10	1,8	10	10	10	1,5
10	10	1,8	1,8	1,8	10	1,5	1,5	1,5	1,5
10	10	1,8	1,8	10	10	10	10	1,7	1,5
1,8	1,5	1,8	1,8	1,8	10	10	2,5	1,8	1,5
10	1,8	2,8	10	10	10	10	10	10	2,8
1,5	1,5	1,5	1,5	1,5	10	10	1,5	1,5	1,5
10	3,9	3,9	3,9	3,9	10	10	1,5	2,7	2,7
1,5	1,5	1,5	1,5	1,5	10	10	1,5	1,5	1,5
1,5	1,5	1,5	1,5	1,5	1,5	1,5	1,5	1,5	1,5
1,5	1,5	1,5	1,5	1,5	10	10	1,5	1,5	1,5
10	2,6	10	10	10	10	10	10	10	2,6
1,5	1,5	1,8	1,8	1,8	1,5	1,5	1,5	1,5	1,5
10	2,6	10	10	10	10	10	10	2,5	2,6
1,5	1,8	1,5	1,5	1,5	1,5	1,5	1,5	1,5	1,5
10	1,7	10	10	10	10	10	10	10	10
1,5	1,5	1,5	1,5	1,5	10	10	10	1,5	1,5
1,5	1,5	1,5	10	10	10	10	1,5	10	1,5

10	1,5	1,8	1,8	1,8	10	10	10	1,8	1,8
1,5	1,5	1,9	10	10	10	10	1,5	10	1,5
1,5	1,5	1,5	1,5	1,5	10	10	10	10	1,5
10	1,5	10	10	10	10	10	10	1,8	1,5
10	1,5	10	10	10	10	10	10	1,8	1,5
10	1,5	10	10	10	10	10	10	1,8	1,5
1,5	1,5	1,8	1,8	1,8	10	1,5	1,5	1,5	1,5
10	10	1,8	1,8	1,8	10	1,5	10	1,5	1,5
1,5	10	1,5	1,5	1,5	10	10	1,5	1,5	1,5
10	1,5	1,9	1,9	1,9	10	1,5	1,5	1,5	1,5
10	1,5	1,9	1,9	1,9	10	1,6	1,6	1,5	1,5
1,5	1,5	1,5	1,5	1,5	1,5	1,5	1,6	1,5	1,5
1,5	1,5	1,8	1,8	1,8	10	1,5	1,5	10	1,5
10	10	10	10	10	10	10	1,5	10	1,5
1,5	1,5	1,8	1,8	1,8	10	1,5	1,5	10	1,5
1,8	1,5	1,9	1,9	1,9	10	10	10	1,6	1,6
10	1,5	1,9	1,9	1,9	10	10	10	1,5	1,5
1,5	1,8	1,5	1,5	1,5	1,5	1,5	1,5	1,5	1,5
1,5	1,5	1,5	1,5	1,5	10	1,5	1,5	10	1,5
10	1,5	1,9	1,9	1,9	10	10	10	1,5	1,5
1,5	1,5	1,8	1,8	1,8	10	10	10	10	1,5
10	1,5	1,5	1,5	1,5	1,5	10	10	10	1,5
10	10	1,8	1,8	1,8	10	10	1,5	1,5	1,5
1,5	1,5	1,8	1,8	1,8	1,5	1,5	1,5	1,8	1,5
1,5	1,5	1,5	1,5	1,5	1,5	1,5	1,5	1,5	1,5
10	10	1,5	1,5	1,5	10	10	1,5	1,5	1,5
1,6	1,6	1,6	10	10	10	10	1,6	1,5	1,5
10	1,5	1,8	1,8	1,8	10	10	1,5	1,5	1,5
10	1,9	1,9	1,9	10	10	1,5	1,5	1,5	1,5
10	10	1,9	1,9	1,9	10	10	1,5	1,5	1,5
10	1,6	1,5	10	10	1,5	10	10	1,5	1,5
1,5	1,5	1,8	1,8	1,8	10	1,6	1,5	10	1,5
1,5	1,5	1,5	1,5	1,5	10	10	1,5	1,5	1,5
1,8	1,5	10	10	10	10	10	10	1,5	1,5
1,5	1,5	1,5	1,5	1,5	1,5	1,5	1,5	1,5	1,5
1,5	1,5	10	10	10	10	10	10	10	1,5
10	1,5	1,9	1,9	1,9	10	10	1,5	1,5	1,5
10	1,5	1,9	1,9	1,9	10	10	1,5	1,5	1,5
10	1,5	1,5	1,5	1,5	10	10	1,5	1,5	1,5
1,5	1,5	1,5	1,5	1,5	1,5	1,5	1,5	1,5	1,5
1,5	1,5	1,8	1,8	1,8	10	1,5	1,5	1,5	1,5
1,5	1,5	1,5	10	10	10	10	10	1,6	1,5
10	1,5	1,5	1,5	1,5	10	10	1,5	1,5	1,5
10	10	1,9	1,9	1,9	10	10	1,5	1,5	1,5
1,5	1,5	10	10	10	10	10	10	1,5	1,8
1,5	1,5	1,8	1,8	1,8	1,5	1,5	1,5	1,5	1,5
1,5	1,5	1,9	1,9	1,9	10	1,5	1,5	1,5	1,5

1,5	1,6	1,5	1,5	1,5	1,5	1,5	1,5	1,5	1,5
1,5	1,6	1,5	1,5	1,5	1,5	1,5	1,5	1,5	1,5
1,5	1,5	1,8	1,8	1,8	10	10	10	1,5	1,5
1,5	1,5	1,8	1,8	1,8	1,5	10	10	1,5	1,5
10	10	1,8	1,8	1,8	10	10	10	1,5	1,5
10	1,5	1,8	1,8	1,8	10	10	10	1,8	1,5
1,8	10	1,9	10	10	10	10	1,9	10	1,5
10	1,5	1,5	1,5	1,5	10	1,5	1,9	1,5	1,5
1,5	1,5	1,5	1,5	1,5	10	1,5	1,6	1,5	1,5
10	1,8	1,9	10	10	1,6	10	10	1,5	1,5
1,5	1,5	1,5	1,5	1,5	1,5	1,5	1,5	1,5	1,5
10	10	10	1,5	10	10	10	10	1,5	1,5
1,5	1,5	1,8	1,8	1,8	10	1,5	1,5	1,8	1,5
10	1,5	1,5	1,5	1,5	1,5	1,5	1,5	1,5	1,5
10	1,8	10	10	10	10	10	10	10	1,8
1,5	1,5	1,5	1,5	1,5	10	10	10	1,5	1,5
1,5	10	1,9	1,9	1,9	1,5	10	10	10	1,5
1,7	1,5	1,5	1,5	1,5	1,6	1,6	1,6	1,6	1,6
1,5	1,5	1,8	10	10	10	10	10	10	1,5
1,6	10	10	10	10	10	10	10	10	10
10	1,5	1,5	1,5	1,5	1,8	1,8	1,8	1,8	1,5
1,5	1,5	1,5	1,5	1,5	10	10	1,5	10	1,5
1,8	1,5	1,5	1,5	1,5	10	10	1,8	10	1,5
10	10	1,9	1,9	1,9	10	10	10	1,5	1,5
1,5	1,5	1,8	1,8	1,8	10	10	1,5	10	1,5
1,5	1,9	10	10	10	10	10	10	10	10
1,5	1,5	1,8	1,8	1,8	10	1,5	1,5	1,5	1,5
1,5	1,5	1,5	1,5	1,5	10	1,5	1,5	1,5	1,5
1,5	1,5	1,5	1,5	1,5	10	1,5	1,5	1,5	1,5
1,5	1,5	1,9	1,9	1,9	1,5	1,5	1,5	1,5	1,5
1,5	1,5	1,8	1,8	1,8	10	10	10	10	1,5
1,5	1,5	1,8	1,8	1,8	10	1,5	1,5	10	1,5
1,5	1,5	2,8	2,8	2,8	10	1,5	1,5	1,5	1,5
1,5	1,5	1,5	1,5	1,5	10	10	10	10	1,5
1,5	1,5	2,8	2,8	2,8	10	1,5	1,5	1,5	1,5
1,5	1,5	1,5	1,5	1,5	1,5	1,5	1,5	1,5	1,5
1,5	10	1,5	10	10	10	10	10	1,5	1,5
10	10	1,8	1,8	1,8	10	10	1,5	1,5	1,5
10	10	1,9	10	10	10	10	1,5	10	1,5
1,5	1,5	1,5	1,5	1,5	10	10	1,5	1,5	1,5
1,5	1,5	1,8	1,8	1,8	10	1,5	10	1,8	1,5
10	1,5	1,5	1,5	1,5	10	10	10	1,8	1,5
1,5	10	10	10	10	10	1,5	10	10	1,5
10	1,5	1,7	1,7	1,7	10	10	10	1,5	1,5
10	1,8	10	10	10	10	10	10	1,9	1,5
10	2,5	10	10	3,9	10	10	10	10	2,5
1,5	1,5	1,5	1,5	1,5	10	10	1,5	1,5	1,5

10	1,5	1,8	1,8	1,8	10	10	10	10	1,5
10	1,5	1,9	1,9	1,9	10	10	10	1,5	1,5
10	10	10	10	10	10	10	10	1,9	1,9
10	10	1,5	1,5	1,5	10	10	10	1,5	1,5
1,9	1,8	10	10	10	10	10	10	1,5	1,5
10	1,5	10	1,5	10	10	10	10	10	1,5
1,5	1,5	1,9	1,9	1,9	10	1,5	1,5	1,5	1,5
1,5	1,5	1,5	1,5	1,5	1,8	1,8	1,8	10	1,5
10	1,5	10	10	10	10	10	10	10	1,5
1,5	1,5	1,5	1,5	1,5	10	1,5	1,5	1,5	1,5
10	10	1,5	1,5	1,5	10	10	10	1,5	1,5
1,5	1,5	1,5	1,5	1,5	1,5	1,5	1,5	1,5	1,5
10	1,5	10	10	10	10	10	10	1,8	1,5
10	1,5	10	10	10	10	10	10	1,8	1,5
10	1,5	1,8	1,8	1,8	10	1,5	10	1,5	1,5
10	10	10	10	10	10	10	10	10	1,5
1,5	1,5	1,8	1,8	1,8	10	1,5	2,5	1,5	1,5
1,5	1,5	1,8	1,8	1,8	10	1,5	2,5	1,5	1,5
10	10	10	10	10	10	10	10	10	1,5
1,5	1,5	1,8	1,8	1,8	10	1,5	10	1,8	1,5
10	1,5	1,9	1,9	1,9	10	10	10	1,5	1,5
10	10	10	10	10	10	10	10	10	1,5
10	10	10	10	10	10	10	10	10	10
10	1,5	1,9	1,9	1,9	10	10	10	10	1,5
10	1,5	1,9	1,9	1,9	10	10	10	10	1,5
10	1,5	10	10	10	10	1,5	1,5	1,5	10
10	1,5	1,9	1,9	1,9	10	10	10	10	1,5
10	1,5	1,8	1,8	1,8	10	1,5	1,5	10	1,5
10	1,5	1,9	1,9	1,9	1,6	1,6	1,6	1,5	1,5
1,5	1,5	1,5	1,5	1,5	1,5	1,5	1,5	1,5	1,5
1,5	1,5	1,8	1,8	1,8	1,5	1,6	1,5	10	1,5
1,5	1,8	1,5	1,5	1,5	10	10	10	1,5	1,5
1,5	1,5	1,9	1,9	1,9	10	10	1,5	1,5	1,5
1,5	1,5	1,5	1,5	1,5	1,5	1,5	1,5	1,5	1,5
1,5	1,6	10	10	10	1,5	1,5	1,5	1,5	1,5
1,5	1,8	1,8	1,8	1,8	1,8	1,8	1,8	1,5	1,5
1,5	1,5	1,8	1,8	1,8	10	1,5	1,5	1,5	1,5
1,5	1,6	1,5	10	10	10	10	10	1,6	1,6
1,5	1,5	1,9	1,9	1,9	10	10	1,5	1,5	1,5
10	10	1,8	1,8	10	10	1,6	1,6	1,8	1,5
1,5	1,5	1,5	1,5	1,5	10	10	10	10	1,5
1,5	1,5	10	10	10	10	10	1,5	10	1,5
1,5	1,5	10	10	10	10	10	1,5	10	1,5
1,5	1,5	1,5	1,5	1,5	1,5	1,5	1,5	1,5	1,5
10	1,5	10	10	10	10	10	1,5	10	1,5
10	1,5	10	10	10	10	1,5	10	10	1,5

boiled rha	tomato gra	raw tomat	tomato sal	beetroot s	bottled be	stewed be	boiled cab	fried cabba	stewed cal
10	1,5	10	10	10	1,5	1,5	10	10	1,5
10	1,5	10	10	10	1,5	1,5	10	10	1,5
10	1,5	10	1,5	1,5	1,5	10	10	10	1,5
10	1,6	10	10	1,9	10	10	10	10	1,5
1,5	1,5	1,5	1,5	1,5	1,5	1,5	1,5	1,5	1,5
10	1,5	10	10	1,5	10	10	10	1,5	10
10	1,5	10	10	10	10	10	10	10	1,5
10	1,5	10	10	1,5	10	10	10	1,5	1,5
10	2,6	10	2,5	2,6	2,6	10	2,5	2,5	2,5
10	1,5	10	10	1,8	1,8	10	10	1,5	1,5
10	1,6	10	10	1,5	1,5	10	10	1,6	1,6
10	2,6	10	10	2,6	2,6	10	10	2,6	2,6
10	2,6	1,5	1,5	2,5	2,5	10	2,5	2,5	2,5
10	1,5	10	1,8	1,8	10	10	10	1,8	10
10	1,5	1,5	10	1,5	1,5	10	10	1,5	1,5
10	1,5	10	10	1,6	1,5	10	10	10	1,5
10	1,5	10	10	1,6	1,5	10	10	10	1,5
10	2,6	1,5	1,5	2,6	1,5	10	1,5	1,5	2,5
2,5	2,5	1,5	2,5	2,5	2,5	10	1,5	1,5	2,5
10	2,6	1,5	2,5	2,5	2,5	10	1,5	1,5	1,5
10	2,6	1,5	1,5	1,5	1,5	10	1,5	1,5	2,6
10	2,5	1,5	1,5	2,5	2,5	10	10	10	2,5
10	2,6	1,5	1,5	2,5	2,5	10	2,5	2,5	2,5
10	2,5	1,5	1,8	2,6	2,6	10	1,5	1,5	2,5
10	2,6	1,5	2,5	2,5	2,5	10	2,5	2,5	2,5
10	2,6	1,5	1,8	2,5	2,5	10	1,5	1,5	1,5
10	1,5	1,5	10	1,8	1,5	10	10	10	1,5
10	1,5	10	10	10	1,6	10	1,5	1,5	1,5
10	1,5	1,5	1,5	1,5	1,5	10	1,5	1,5	1,5
10	1,6	10	10	1,8	2,8	1,6	2,7	1,5	1,7
10	1,5	10	10	10	1,5	10	1,5	10	1,5
10	2,6	10	10	2,6	2,5	1,5	1,5	1,5	1,5
1,5	2,5	10	2,5	2,6	2,6	10	10	10	2,6
10	1,5	10	10	1,8	10	10	10	1,5	10
1,9	1,5	10	10	10	1,5	10	10	1,5	1,5
10	2,5	10	10	1,8	1,5	10	10	1,5	1,5
10	1,6	10	10	1,5	10	10	1,5	2,6	1,5
10	1,5	10	10	10	1,5	10	10	10	1,5
10	10	10	10	1,5	1,5	10	10	10	2,6
10	1,8	10	10	1,8	1,8	10	1,8	1,8	1,8
10	1,6	10	10	1,5	1,6	10	10	10	1,6
10	1,5	1,5	1,5	1,5	1,5	10	1,5	1,5	1,5
10	1,5	10	1,5	1,8	1,9	10	10	10	1,5
10	1,5	10	10	1,5	1,5	10	10	1,5	1,5
10	1,5	10	10	1,5	1,5	1,5	10	1,5	1,5
10	1,5	10	10	1,6	10	10	10	1,5	10

10	1,5	10	10	1,5	10	10	10	1,5	1,5
10	2,5	10	10	1,6	10	10	10	2,8	2,8
10	2,6	10	10	1,6	1,5	10	10	10	2,6
10	1,5	10	1,5	1,5	1,5	1,5	1,5	1,5	10
1,5	2,5	10	10	1,5	1,5	10	10	1,5	1,6
1,9	10	10	10	10	1,5	10	1,8	1,8	1,8
10	10	10	1,5	1,5	1,5	10	1,5	1,5	1,5
1,9	1,5	10	1,8	1,8	1,5	10	10	10	10
1,9	1,5	10	10	10	10	10	10	1,8	1,8
10	1,6	10	10	1,8	1,8	10	10	1,5	1,5
10	1,6	10	10	1,8	1,5	10	10	1,5	1,5
10	1,6	10	10	10	10	10	10	1,5	1,5
10	1,6	10	10	1,5	10	10	10	10	10
1,5	2,6	10	1,5	2,5	2,5	10	10	2,5	2,6
10	1,5	10	10	1,5	10	10	10	10	1,5
10	1,5	10	1,8	1,8	10	10	1,8	1,5	1,5
10	1,5	10	10	1,8	1,5	10	1,6	10	1,8
1,6	2,5	10	10	10	1,7	2,6	1,5	1,6	1,7
10	2,5	10	10	1,5	1,5	10	10	1,5	1,5
10	1,5	10	10	1,8	1,5	10	1,8	1,8	1,8
10	2,6	10	10	2,5	2,5	10	2,5	2,5	2,6
2,5	2,6	10	2,5	2,5	2,6	10	2,5	2,5	2,6
2,5	2,6	10	2,5	2,5	2,6	10	2,5	2,5	2,6
10	1,5	1,5	1,5	1,5	1,5	10	1,5	1,5	1,5
10	1,5	1,5	1,5	1,5	1,5	10	1,5	1,5	1,5
10	1,5	1,5	10	1,8	1,5	10	10	1,5	1,5
10	1,6	10	10	1,5	1,5	10	10	1,5	1,5
10	1,5	10	1,8	1,7	10	10	10	1,5	1,5
10	1,5	10	1,5	2,5	1,5	2,5	2,6	2,5	2,6
10	1,6	10	10	10	1,5	10	10	1,5	1,5
10	1,5	10	10	1,8	10	10	10	10	2,5
10	1,5	10	10	1,8	10	10	10	10	2,5
10	1,5	1,5	10	10	1,5	10	10	1,5	10
10	1,6	10	10	1,8	1,8	1,7	2,7	1,6	1,5
10	1,6	10	10	10	1,5	10	10	10	1,5
10	2,6	1,8	10	2,5	2,6	1,5	1,5	1,5	2,5
10	10	10	10	10	10	10	1,5	1,5	2,5
10	2,6	10	1,5	10	1,5	10	10	1,5	1,5
10	2,5	10	10	1,8	1,5	10	10	10	10
10	1,6	10	10	1,5	1,5	10	10	1,5	1,5
10	1,5	10	10	1,5	1,5	10	10	1,5	1,5
10	2,6	10	1,5	1,5	1,5	10	10	10	1,5
10	2,5	10	10	1,5	1,5	10	10	10	1,5
10	1,5	10	10	1,5	1,5	10	10	1,5	1,5
10	1,5	10	10	1,5	1,5	10	1,5	1,5	1,5
10	1,5	10	10	1,5	1,5	10	10	10	1,5
10	2,6	1,8	10	2,5	1,5	10	1,5	1,5	2,6

1,8	1,6	10	10	1,8	10	10	10	1,5	1,5
2,9	1,5	10	10	10	1,5	10	10	1,5	10
1,9	1,6	10	10	10	1,7	10	10	1,5	1,6
1,9	1,6	10	10	1,5	1,6	10	10	1,5	1,8
1,9	1,5	10	10	1,5	1,5	10	1,5	1,8	1,5
2,6	4,7	10	10	10	10	10	10	10	10
2,5	2,5	2,5	2,5	2,5	2,6	10	1,5	2,5	2,6
1,8	1,5	10	10	10	1,5	10	1,5	1,5	1,5
1,8	1,6	10	10	10	1,5	10	1,5	1,5	1,5
10	2,6	10	10	1,5	10	1,9	10	1,5	1,5
10	1,5	10	1,8	1,8	1,8	10	10	1,8	1,8
10	1,5	10	10	1,5	10	10	10	1,5	1,5
10	1,7	10	10	1,5	10	10	10	1,7	1,7
10	1,5	10	10	1,5	1,5	10	10	10	10
10	1,5	10	1,8	1,8	1,8	10	10	1,8	1,8
1,5	3,6	1,5	1,5	2,5	1,5	1,5	2,6	2,6	2,6
10	1,8	10	10	1,8	1,8	10	2,8	2,8	1,8
10	1,5	10	10	1,5	1,5	10	10	1,5	10
10	1,5	10	10	10	1,5	10	10	10	2,5
1,9	1,5	10	1,5	1,5	10	10	10	1,5	1,5
10	2,6	1,5	10	1,5	1,5	1,6	2,6	2,6	2,6
10	1,5	10	10	10	10	10	10	10	1,5
10	1,6	10	10	1,6	1,6	10	1,6	1,6	1,6
10	1,5	10	10	1,6	1,6	10	1,6	1,6	1,6
10	1,6	10	10	1,6	1,6	10	1,6	1,6	1,6
10	3,5	10	10	2,5	2,5	10	10	10	2,5
1,8	1,8	10	10	1,8	1,8	10	1,8	1,8	10
10	1,6	10	10	10	1,5	10	10	1,5	1,5
10	1,5	10	10	1,5	10	10	10	1,5	1,5
10	2,6	1,5	1,5	2,5	2,5	10	2,6	2,6	2,6
10	1,5	10	10	1,5	1,5	1,5	10	1,8	1,8
10	1,6	10	10	1,5	1,5	10	1,6	1,6	1,5
10	2,6	1,5	10	2,5	2,6	1,5	1,5	2,5	2,5
10	1,5	10	10	1,8	1,5	10	1,6	1,5	2,5
10	1,5	10	10	10	1,6	10	10	1,6	1,6
10	2,5	10	10	10	1,6	1,6	1,5	1,5	1,8
10	1,5	10	10	1,7	1,7	10	10	1,6	1,5
10	2,5	10	10	2,5	10	10	10	10	10
10	1,5	10	1,5	1,5	1,5	10	10	1,5	1,5
10	1,5	10	1,8	10	1,5	10	10	1,6	1,5
10	1,5	10	10	1,5	10	10	10	1,5	1,5
10	1,5	10	10	1,5	1,5	10	1,5	1,5	10
10	1,8	10	10	1,8	1,5	10	1,5	1,5	1,5
10	1,6	10	10	10	10	10	10	1,6	1,6
10	2,6	10	1,5	2,6	2,5	10	2,5	2,5	2,6
1,5	1,5	10	10	1,5	10	10	1,5	1,5	1,5
10	2,6	10	1,5	2,5	2,5	10	2,6	2,6	2,6

10	1,5	10	10	1,5	10	10	10	1,5	1,5
10	1,6	10	10	10	1,5	10	10	10	1,5
10	2,5	10	1,5	2,5	2,5	10	10	10	2,5
10	2,5	10	1,5	2,5	2,5	10	10	10	2,5
10	1,5	10	1,8	1,5	1,6	10	10	1,8	1,8
1,5	1,5	1,5	1,5	1,5	1,5	10	1,5	1,5	1,5
2,6	2,6	10	1,5	2,5	2,5	10	1,5	1,5	2,6
10	1,6	10	10	1,5	1,5	10	10	1,5	1,6
10	1,8	10	10	1,5	1,5	10	10	10	1,5
10	1,5	10	1,5	1,5	1,5	10	10	1,5	1,5
10	1,5	10	3,5	3,5	3,5	10	10	10	3,5
10	1,5	10	3,5	3,5	3,5	10	10	10	3,5
10	1,5	10	1,5	1,5	10	1,9	10	1,5	1,5
10	1,5	10	10	10	10	1,5	1,6	1,6	1,6
10	10	10	10	1,5	1,5	10	10	1,5	1,6
10	1,5	10	10	1,5	1,5	10	10	1,5	1,5
10	1,5	10	10	1,5	1,5	10	10	1,5	1,6
10	1,5	10	10	1,5	1,5	10	10	1,5	1,5
10	2,6	1,5	10	2,5	2,5	10	10	10	2,6
10	1,5	10	10	10	1,5	1,6	10	1,5	1,5
10	2,5	10	10	10	1,5	10	10	1,5	10
10	1,5	10	1,5	1,5	10	10	10	10	1,5
10	1,5	10	1,6	1,6	10	10	10	10	1,5
10	2,6	10	1,5	2,5	10	10	10	10	2,5
10	2,6	10	1,5	2,5	10	10	10	10	2,5
10	1,6	10	10	1,6	1,6	1,6	10	1,5	1,6
10	1,5	10	1,5	1,5	1,5	1,5	1,5	1,5	10
10	1,5	10	10	1,8	10	1,8	10	1,5	1,5
10	1,7	1,6	1,5	1,5	10	10	10	1,5	1,5
10	2,6	1,5	1,5	2,6	2,5	10	10	2,5	2,5
10	10	10	10	10	1,5	10	10	10	1,5
10	1,5	10	10	10	1,5	10	10	1,5	1,5
10	1,5	10	10	10	1,5	10	10	1,5	10
10	2,6	2,5	2,5	2,6	2,6	10	2,5	2,5	2,5
10	1,5	1,5	1,5	1,5	1,5	10	10	10	1,5
10	1,5	10	10	1,5	1,5	10	1,5	1,5	10
10	1,5	10	10	10	1,5	10	10	1,5	1,5
10	1,5	10	10	10	1,5	10	10	1,5	10
10	1,5	10	1,5	2,5	1,5	10	10	10	10
10	1,5	10	1,5	2,5	1,5	10	10	10	10
10	1,5	10	10	1,5	1,5	10	1,5	1,5	1,5
10	1,5	1,8	2,5	1,5	1,5	10	10	10	1,5
10	1,5	10	10	10	1,5	10	10	1,5	1,5
1,5	1,5	1,5	1,5	1,5	1,5	1,5	1,5	1,5	1,5
1,5	1,5	1,5	1,5	1,5	1,5	1,5	1,5	1,5	1,5
10	1,5	10	10	10	1,5	1,8	10	10	1,5
10	1,5	10	10	1,8	1,5	10	10	1,5	1,5

10	1,5	10	10	10	1,6	1,5	10	10	1,5
10	1,5	10	10	1,5	10	10	1,5	1,8	1,5
10	1,5	10	1,5	1,5	10	10	1,5	1,5	1,5
10	2,6	10	2,5	1,5	1,5	1,5	2,6	2,6	2,6
10	2,6	10	2,5	1,5	1,5	1,5	2,6	2,6	2,6
10	1,5	10	1,5	1,5	1,5	10	10	1,5	1,5
10	10	10	10	1,5	10	10	10	1,5	10
10	2,5	10	10	1,5	10	10	10	10	1,5
10	1,5	10	10	10	1,5	10	1,5	1,5	1,5
10	1,8	10	10	1,5	1,5	10	10	1,5	1,5
10	1,5	10	10	10	1,5	10	10	1,5	1,5
10	2,5	10	10	1,5	1,5	10	10	10	1,5
10	1,6	10	1,5	1,5	1,5	10	10	1,5	1,5
10	1,5	10	10	1,5	10	10	10	1,5	1,5
10	1,5	1,5	1,5	1,5	1,5	1,5	10	2,5	2,5
10	1,6	10	10	1,5	1,5	10	10	1,5	10
10	1,5	1,5	1,5	1,5	1,5	1,5	10	1,5	1,5
10	1,5	10	10	1,5	1,5	10	10	1,5	1,5
10	1,6	10	1,5	10	1,5	10	1,5	1,5	10
10	1,7	10	10	1,8	1,5	1,8	1,5	1,5	1,5
1,9	1,5	10	10	1,5	1,5	10	10	1,5	1,5
10	1,5	10	10	1,5	1,4	10	1,5	1,5	1,5
10	1,5	10	10	1,6	1,5	10	1,5	1,5	1,5
10	2,5	10	10	10	1,5	10	10	10	3,5
10	1,5	10	10	1,5	1,5	10	10	2,5	2,5
10	1,5	10	10	1,5	1,5	10	1,5	1,5	1,5
10	1,5	10	2,5	1,5	2,5	10	2,5	2,5	2,5
10	1,5	10	10	1,5	1,5	10	1,5	1,5	1,5
10	1,5	10	10	10	1,5	10	10	10	1,5
10	1,5	10	1,5	10	1,6	10	1,5	1,5	1,5
10	2,6	1,5	10	1,5	1,5	10	10	10	1,5
10	1,5	10	10	10	1,5	10	10	1,7	1,5
10	1,5	1,5	1,5	1,5	1,5	1,5	1,5	1,5	1,5
10	2,6	10	10	1,5	1,5	10	1,5	10	1,5
10	1,5	10	10	10	10	10	1,5	10	1,5
10	1,5	10	10	1,5	1,5	10	1,5	1,5	1,5
10	1,5	10	1,5	1,5	1,5	1,5	10	10	1,5
10	1,5	10	10	10	1,5	10	10	1,5	1,5
10	1,5	10	10	1,5	10	10	10	1,6	1,6
10	1,5	1,5	10	1,5	1,5	10	10	1,5	1,5
10	1,5	10	10	1,9	1,9	10	10	10	1,5
10	1,5	10	10	10	1,5	10	1,5	1,5	1,5
10	10	1,5	10	1,8	1,5	1,8	10	1,5	1,5
10	2,5	10	10	10	1,5	10	10	10	2,5
10	10	1,5	10	1,8	1,5	1,8	10	1,5	1,5
10	2,6	10	10	10	1,6	10	10	2,5	2,5
10	1,5	2,5	10	2,5	2,5	10	2,5	2,5	2,5

10	1,5	2,5	10	2,5	2,5	10	2,5	2,5	2,5
10	1,6	10	10	1,5	1,5	10	1,5	1,5	1,5
2,5	10	10	10	1,5	1,5	10	10	10	2,5
10	1,5	10	1,5	10	1,5	1,5	1,5	1,5	10
10	1,5	10	10	1,5	1,5	10	10	1,5	10
10	1,5	10	10	10	1,5	10	1,5	1,5	1,5
10	1,5	10	10	1,5	10	10	10	1,5	1,5
10	1,5	10	10	1,5	1,5	10	10	1,5	1,5
10	10	10	10	1,5	10	10	1,5	1,5	10
10	1,5	10	10	10	1,5	10	10	1,5	1,5
1,5	1,5	1,5	1,5	1,5	1,5	1,5	1,5	1,5	10
10	1,5	10	10	1,5	1,5	10	1,5	1,5	1,5
2,5	2,5	10	10	10	1,5	10	10	2,5	10
10	2,5	10	10	1,5	10	10	10	2,5	10
10	1,5	10	10	1,9	10	1,8	10	1,5	10
10	2,5	10	10	10	1,5	10	10	1,5	1,7
1,5	2,6	1,5	1,5	1,5	1,5	10	2,5	2,5	2,6
1,5	2,6	1,5	1,5	1,5	1,5	10	2,5	2,5	2,6
10	1,5	10	10	10	10	10	10	10	1,5
1,9	1,5	10	1,8	1,7	1,6	10	1,5	1,5	1,5
10	2,6	10	10	10	1,5	10	10	10	2,5
10	2,5	10	10	1,5	1,5	10	10	10	1,5
10	1,5	10	10	1,8	1,6	10	10	1,5	1,5
10	2,5	10	10	1,5	1,5	10	10	10	1,5
10	2,5	10	10	1,5	1,5	10	10	10	1,5
10	1,5	1,5	1,5	1,5	1,5	10	10	1,5	10
10	1,5	10	10	1,9	10	1,8	10	1,5	10
10	1,5	10	10	1,5	1,5	10	10	1,5	1,5
10	1,6	10	10	1,5	10	10	10	10	1,5
10	2,5	10	10	1,5	10	10	10	2,5	1,7
10	1,5	10	10	1,5	10	10	1,5	10	10
1,9	1,5	10	10	10	1,5	10	10	10	1,8
10	1,6	10	10	1,5	1,5	10	1,5	1,5	1,5
1,8	1,5	1,5	1,5	1,5	10	10	10	1,5	1,5
10	2,6	1,5	1,5	1,5	1,5	2,5	2,5	2,5	2,5
10	1,5	10	10	1,6	1,6	10	1,5	1,5	1,5
10	10	10	10	1,8	10	10	10	10	2,5
10	1,5	10	10	1,5	1,5	10	1,5	1,5	10
1,9	1,6	10	10	10	1,6	10	10	1,6	1,6
10	1,5	1,5	1,5	1,5	1,5	10	1,5	1,5	1,5
10	2,5	10	10	1,5	10	10	10	10	1,5
10	1,5	10	10	1,5	10	10	10	10	1,5
10	1,6	10	10	1,5	1,5	10	10	1,5	1,5
10	1,6	10	10	1,5	1,5	10	10	1,5	1,5
10	1,5	10	10	1,5	1,5	10	10	10	1,5
10	1,5	10	10	1,5	1,5	10	10	10	1,5
10	1,5	1,6	10	1,6	1,5	10	10	1,5	1,5

10	1,5	1,5	1,5	1,5	1,5	10	1,5	1,5	1,5
10	1,5	10	10	10	10	10	10	1,5	1,4
10	1,5	10	10	1,5	1,5	1,5	1,5	1,5	1,5
10	1,6	10	1,5	1,5	1,5	10	10	10	1,5
10	1,5	1,5	1,5	1,5	1,5	1,5	1,5	1,5	1,5
10	10	10	10	10	1,5	10	10	1,5	1,5
10	1,5	10	10	1,5	1,5	1,5	10	1,8	1,5
10	1,5	1,6	10	1,5	1,5	10	10	1,5	1,5
10	1,5	1,6	10	1,5	1,5	10	10	1,5	1,5
10	1,5	1,6	10	1,5	1,5	10	10	1,5	1,5
10	1,6	10	10	1,6	10	10	10	10	10
10	1,5	10	10	1,5	1,5	1,5	1,5	1,5	1,5
10	10	10	10	10	1,5	10	10	10	1,5
10	1,5	1,5	10	1,5	10	10	10	1,5	1,5
10	1,6	10	1,5	1,5	1,5	10	10	1,5	1,5
10	1,5	10	10	1,5	1,5	10	10	10	1,5
10	1,5	10	10	1,5	1,5	10	10	1,5	1,5
10	1,6	10	10	1,5	10	10	10	1,5	10
10	1,6	1,5	10	1,6	10	10	1,5	1,5	1,5
10	1,5	10	1,5	1,5	1,5	1,5	1,5	1,5	1,5
10	1,5	10	10	10	10	10	1,5	1,5	1,5
10	1,6	10	10	1,5	1,6	10	10	1,5	1,5
10	1,6	10	10	1,8	1,6	10	10	1,5	1,5
10	1,5	10	10	1,8	1,5	10	10	1,5	1,8
10	1,5	10	1,5	1,8	1,8	10	1,5	1,5	1,5
1,8	1,5	10	1,5	1,5	1,5	10	1,5	1,5	1,5
10	1,5	10	10	1,5	1,6	10	10	1,5	1,5
1,5	1,5	10	1,5	1,5	1,5	10	1,5	1,5	1,5
10	1,5	1,8	1,5	1,8	1,6	10	10	1,8	1,8
1,5	1,5	10	10	1,5	1,5	10	1,5	1,5	1,5
10	1,5	10	10	10	10	10	1,5	1,5	10
10	1,5	10	10	10	10	10	10	10	1,5
10	1,5	10	10	2,5	2,5	10	10	1,5	10
10	1,5	1,5	1,5	1,5	1,5	1,5	1,5	1,5	1,5
10	1,6	10	10	1,8	1,6	10	10	1,5	1,5
10	1,5	10	2,6	1,5	1,5	1,5	1,5	1,5	10
10	1,5	10	10	1,5	10	10	10	10	1,5
10	1,5	10	10	10	1,6	10	10	1,5	1,5
10	1,6	10	10	1,5	1,6	10	10	1,5	1,5
10	1,5	10	10	1,5	1,6	10	10	1,5	1,5
1,9	1,5	10	10	10	1,5	10	10	1,5	1,6
10	1,5	10	10	10	10	10	1,5	1,5	1,5
10	1,5	10	10	1,5	1,5	1,5	10	1,5	1,5
10	1,5	10	10	10	10	10	10	1,5	10
1,5	1,5	10	10	10	1,5	1,5	1,5	1,5	1,5
10	1,5	10	10	1,5	1,5	10	10	10	1,5
10	1,7	10	10	1,5	1,5	10	10	10	1,5

10	1,6	10	10	1,5	1,5	10	10	10	1,5
10	1,5	10	1,5	1,5	1,5	10	1,5	1,5	1,5
10	1,5	10	10	10	10	10	1,5	1,5	1,5
10	1,6	10	1,6	1,6	10	10	10	1,5	1,6
10	1,5	1,5	10	1,5	1,5	10	10	10	1,5
10	1,5	10	10	10	1,5	1,5	1,5	1,5	1,6
10	1,6	10	10	1,5	1,5	10	10	10	1,5
10	1,6	10	10	1,5	1,5	10	10	10	1,5
10	1,5	10	10	1,5	1,5	1,5	1,5	1,5	1,5
10	2,6	1,6	10	2,5	10	10	10	10	1,6
10	1,5	10	10	1,8	1,5	10	10	10	1,5
10	1,6	10	10	10	1,5	1,5	1,5	1,5	10
10	1,6	10	10	1,5	1,5	10	10	1,5	1,5
10	1,6	10	10	1,5	1,5	10	10	10	1,5
10	1,6	10	10	1,5	1,5	10	10	10	1,5
10	1,6	10	10	1,5	10	10	10	10	1,5
10	1,6	10	10	1,5	10	10	10	10	1,5
10	1,6	10	10	1,5	1,5	10	10	10	1,5
10	1,5	1,5	10	1,5	1,5	10	10	10	1,5
10	1,5	10	1,5	1,5	1,5	10	1,5	1,5	1,5
10	1,6	10	10	1,5	1,5	10	10	1,5	1,5
10	1,5	10	10	1,8	1,5	1,9	10	10	1,5
10	1,6	10	10	1,5	1,5	10	10	10	1,5
10	1,5	1,5	1,5	1,5	1,5	1,5	1,5	1,5	1,5
10	1,6	10	10	1,5	1,5	10	10	10	1,5
10	1,5	10	10	1,8	1,5	10	10	1,5	1,5
10	1,6	10	10	1,5	1,5	10	10	10	1,5
10	1,6	10	10	1,5	1,5	10	10	10	1,5
10	1,6	10	10	1,5	1,5	10	10	10	1,5
10	1,6	10	10	1,5	1,5	10	10	10	1,5
10	10	10	10	2,8	10	10	10	1,8	1,8
10	2,6	1,5	10	2,5	2,5	10	10	10	2,5
10	10	10	10	1,7	1,7	10	10	1,5	1,5
10	1,6	10	10	1,5	1,5	10	1,5	1,5	10
2,9	2,6	10	10	2,8	2,7	10	10	2,5	2,5
10	1,5	10	1,5	1,5	1,5	1,5	1,5	1,5	1,5
3,6	10	10	10	1,5	1,5	10	10	10	10
10	1,5	10	10	1,5	1,5	10	10	1,5	1,5
10	1,5	10	1,5	1,5	1,5	10	1,5	1,5	1,5
1,5	1,5	10	1,5	1,5	1,5	10	1,5	1,5	1,5
10	2,7	1,7	10	10	10	10	10	2,5	3,6
10	1,6	10	1,5	1,5	1,5	10	10	10	1,5
10	10	10	10	10	10	10	10	10	2,6
1,9	1,5	10	10	10	1,5	10	1,5	1,5	1,5
10	1,6	10	10	10	10	10	10	10	10
10	1,5	10	1,5	1,5	1,5	1,5	1,5	1,5	10
10	1,5	10	1,5	1,5	10	10	1,5	1,5	10

10	1,5	10	10	1,8	1,5	10	10	10	1,5
10	1,5	10	10	10	1,5	10	10	1,6	10
10	1,5	10	10	1,5	1,5	1,5	1,5	1,5	10
10	1,5	10	10	1,8	1,5	10	10	10	1,5
10	1,5	10	10	1,8	1,5	10	10	10	1,5
10	1,5	10	10	1,8	1,5	10	10	10	1,5
10	1,6	10	1,5	1,5	1,5	10	10	1,5	10
10	1,6	1,5	1,5	1,5	1,5	10	2,5	2,5	2,6
10	1,5	10	10	1,5	1,5	10	1,5	1,5	1,5
10	1,5	10	10	1,5	1,5	10	10	10	1,5
10	1,5	1,5	10	1,6	1,6	10	10	19	1,5
1,9	1,5	10	10	1,5	1,5	1,5	1,5	1,5	10
10	1,6	10	10	1,5	1,5	10	10	10	1,5
10	1,5	10	10	1,5	10	10	1,5	1,5	10
10	1,6	10	10	1,5	1,5	10	10	10	1,5
10	1,5	10	10	1,5	1,5	1,5	10	1,5	1,7
10	1,5	10	10	1,5	1,5	10	10	10	1,5
10	1,5	10	10	10	10	10	10	10	1,5
10	1,6	1,5	1,5	1,5	10	10	1,5	1,5	1,5
10	1,5	10	10	1,5	1,5	10	10	10	1,5
10	1,5	10	10	1,5	1,6	10	10	1,5	1,5
1,9	1,5	10	1,5	1,5	1,5	1,5	1,5	2,5	10
10	1,5	10	10	1,5	1,5	10	10	10	1,5
10	1,6	10	10	1,5	1,5	10	10	10	1,5
10	1,5	10	10	10	10	10	1,5	1,5	1,5
10	1,5	10	10	1,5	1,5	10	10	10	2,5
10	1,5	1,6	10	1,5	1,5	1,6	10	1,5	1,6
10	1,5	10	10	1,5	1,5	10	10	10	1,5
10	1,5	10	10	1,5	1,5	10	10	10	2,6
10	1,5	10	10	1,5	1,5	10	10	10	2,6
10	1,5	10	10	1,5	1,6	1,6	10	1,6	1,5
10	1,6	1,5	1,8	1,5	1,5	10	10	1,5	1,5
1,8	1,5	10	1,5	1,5	1,5	10	1,5	1,5	1,5
10	1,5	10	10	1,8	1,5	10	10	1,5	10
1,5	1,5	10	10	1,5	1,5	1,5	1,5	1,5	1,5
10	1,5	1,8	1,8	1,5	1,5	10	10	10	1,5
10	1,5	10	10	1,5	1,5	10	10	10	1,5
10	1,5	10	10	1,5	1,5	10	10	10	1,5
10	1,5	10	1,5	1,5	1,5	1,5	1,5	1,5	1,5
10	1,5	1,5	1,5	1,5	1,5	1,5	10	10	1,5
10	1,5	1,5	10	1,5	1,5	1,5	1,5	1,5	1,5
10	1,5	10	10	1,5	1,5	10	10	1,5	1,5
10	1,5	10	1,5	1,5	1,5	1,5	1,5	1,5	1,5
10	1,5	10	10	1,5	1,5	10	10	10	1,5
10	1,6	10	10	10	10	10	10	10	1,5
1,9	1,5	10	1,5	1,5	1,5	10	10	10	1,5
10	1,5	1,5	1,5	1,5	1,5	1,5	10	1,6	10

1,9	1,5	10	10	1,8	1,8	1,8	1,5	1,5	1,6
1,5	1,5	10	10	1,8	1,8	1,5	1,8	1,8	1,6
10	1,5	10	10	10	1,5	10	10	1,5	1,5
10	1,5	10	10	10	10	10	10	1,5	1,5
10	1,5	10	10	10	10	10	10	1,5	1,5
10	1,5	1,5	1,5	1,5	1,5	10	10	1,5	1,5
10	10	10	10	1,5	10	10	1,5	10	10
10	1,6	1,5	1,5	1,6	1,6	10	1,5	1,5	1,9
10	1,6	1,5	1,5	1,6	1,6	10	1,5	1,5	1,5
10	1,5	10	10	1,5	10	10	1,5	10	10
10	1,5	1,5	1,5	1,5	1,5	1,5	1,5	1,5	1,5
10	1,5	10	10	10	10	10	1,5	1,5	1,5
10	1,6	10	1,5	1,5	1,5	10	10	1,5	1,5
10	1,5	1,5	1,5	1,5	1,5	10	10	10	10
10	1,5	10	10	1,5	1,5	1,5	10	1,5	1,5
10	1,5	10	10	1,6	1,6	10	10	10	10
10	1,5	1,5	10	1,5	10	10	10	1,5	10
10	1,6	10	1,5	1,5	1,5	10	10	1,5	1,5
10	1,5	10	10	1,5	1,5	10	10	1,5	1,5
10	10	1,8	10	1,7	10	10	10	10	10
10	1,6	10	10	1,5	1,5	1,8	1,8	1,8	1,8
10	1,5	10	10	1,5	15	10	1,5	1,5	1,5
10	1,5	10	10	1,5	1,5	10	10	1,5	1,5
10	1,5	10	10	1,5	1,5	10	10	10	1,6
10	1,5	10	10	1,5	1,5	10	10	1,5	1,5
10	1,5	10	1,5	1,5	1,5	10	10	10	1,5
10	2,5	1,5	1,5	1,5	1,5	10	10	1,5	1,5
1,5	1,5	10	10	1,5	1,5	10	10	10	1,6
10	1,5	10	1,5	1,5	1,5	1,5	1,5	1,5	1,5
10	1,5	10	1,5	1,5	1,5	10	1,5	1,5	1,5
10	1,5	10	10	1,5	1,5	10	10	1,5	1,5
10	1,5	1,5	1,5	1,5	1,5	10	10	1,5	1,5
10	1,5	1,5	1,8	10	10	10	10	1,5	1,5
10	1,5	10	10	10	10	10	10	1,5	1,5
10	1,5	1,5	1,8	10	10	10	10	1,5	1,5
1,5	10	10	10	1,5	1,5	1,5	1,5	1,5	1,5
10	1,5	10	10	1,5	1,5	10	1,6	10	10
10	1,5	10	1,5	1,5	1,5	10	10	1,5	1,5
10	2,5	10	10	10	10	10	3,6	3,6	19
10	1,5	10	1,5	1,5	1,5	10	1,5	1,5	1,5
10	1,5	10	1,5	1,5	1,5	10	10	10	1,5
10	1,5	10	1,5	1,5	1,5	10	10	10	1,5
10	1,5	10	10	1,5	10	10	1,5	10	10
10	1,5	10	10	1,5	1,5	10	10	1,5	1,5
10	1,5	1,5	10	1,8	1,5	10	10	10	1,5
10	10	10	10	2,5	2,5	10	10	1,5	1,5
10	1,5	10	1,5	1,5	1,5	10	10	10	1,5

10	1,5	10	10	10	1,5	10	10	1,5	1,5
10	1,5	10	10	2,5	2,5	10	10	1,5	1,5
10	2,5	10	10	1,8	1,5	10	10	1,5	10
10	1,6	10	10	1,5	1,5	10	10	10	1,6
1,5	1,5	10	10	10	1,5	1,5	1,8	1,8	10
10	1,5	10	10	1,5	1,5	10	10	10	1,5
10	1,5	1,5	1,5	1,5	1,5	1,5	10	1,5	1,5
10	1,6	10	10	10	1,5	1,8	1,5	1,5	1,5
10	1,5	10	1,5	10	1,5	10	10	10	1,5
10	1,7	1,5	1,5	1,5	1,5	1,5	1,5	1,5	1,5
10	1,5	10	10	1,5	1,5	10	10	10	1,6
10	1,5	1,5	1,5	1,5	1,5	1,5	1,5	1,5	1,5
10	1,5	10	10	1,5	1,6	10	10	10	1,5
10	1,5	10	10	1,5	1,6	10	10	10	1,5
10	1,5	1,5	1,5	1,5	1,5	10	10	10	1,5
10	1,5	10	10	10	1,5	10	10	10	10
10	1,6	1,5	1,5	1,5	1,5	10	10	1,5	1,5
10	1,6	1,5	1,5	1,5	1,5	10	10	1,5	1,5
10	1,5	10	10	1,5	1,5	10	10	10	1,5
10	1,6	10	10	1,5	1,5	10	10	10	1,5
10	1,5	10	10	10	10	10	10	10	1,5
10	1,5	10	10	10	1,5	10	10	10	10
10	10	10	10	10	10	10	10	10	10
10	1,6	10	10	10	10	10	10	10	1,5
10	1,5	10	10	1,5	1,5	10	10	1,5	1,5
10	1,5	10	10	1,5	1,5	10	1,5	10	10
10	1,5	10	10	1,5	1,5	10	10	1,5	1,5
10	1,5	10	10	1,5	1,5	10	10	10	1,5
10	1,5	1,5	1,5	1,5	1,5	10	10	10	1,6
1,5	1,5	1,5	1,5	1,5	1,5	1,5	1,5	1,5	1,5
1,9	1,6	10	1,8	1,5	1,5	10	10	10	1,5
10	1,5	10	1,5	1,5	1,5	1,5	1,5	1,5	1,5
10	1,6	10	10	1,5	1,5	10	10	10	1,5
10	1,5	10	10	1,5	1,5	1,5	1,5	1,5	10
10	1,5	10	10	1,6	10	10	1,6	1,8	1,5
10	1,5	1,5	1,5	1,5	1,6	10	10	1,5	1,5
1,9	1,5	10	1,5	1,5	1,5	10	10	1,5	1,5
1,6	1,6	10	10	1,5	10	10	10	1,5	1,5
10	1,5	10	10	1,5	1,5	10	10	10	1,5
10	1,6	1,5	1,5	1,5	1,5	10	10	10	1,5
10	1,5	10	10	10	10	10	1,5	1,5	10
10	1,5	10	10	10	10	10	10	1,5	1,5
10	1,5	10	10	10	10	10	10	1,5	1,5
1,5	1,5	1,5	1,5	1,5	1,5	1,5	1,5	1,5	1,5
10	1,5	10	10	1,5	10	10	1,5	1,5	1,5
10	1,6	10	10	1,8	1,6	10	10	10	1,5

cabbage s	stewed co	stewed co	dried cowp	dried cowp	stewed pu	stewed pu	stewed pu	stewed pu	stewed pu
1,6	10	10	10	10	10	10	10	1,5	10
1,6	10	10	10	10	10	10	10	1,5	10
1,5	1,5	1,5	1,5	1,5	1,5	1,5	1,5	1,5	1,5
1,8	1,5	1,5	1,8	10	10	1,9	1,9	1,9	1,8
1,5	1,5	1,5	1,5	1,5	1,5	1,5	1,5	1,5	1,5
1,5	10	10	10	10	1,5	10	10	10	1,8
10	1,9	10	1,5	10	1,5	1,5	10	10	1,5
1,5	10	10	10	10	10	1,5	10	10	10
10	2,9	2,9	2,6	2,6	2,9	2,9	2,9	2,9	2,9
1,5	1,8	10	1,8	10	10	1,9	1,9	1,9	1,9
1,5	10	10	10	10	10	10	10	1,5	1,6
2,6	2,6	10	2,6	10	2,6	2,6	2,6	2,6	2,6
2,5	2,5	10	2,5	2,5	2,5	2,5	2,5	2,5	2,5
10	10	10	1,5	1,9	1,5	10	10	10	1,5
1,5	1,9	10	1,5	10	1,9	1,9	10	1,9	1,9
1,5	10	10	10	10	1,5	1,5	1,6	1,6	1,6
1,5	10	10	10	10	1,5	1,5	1,6	1,6	1,6
2,5	2,5	10	2,6	10	2,5	2,5	2,5	2,5	2,5
2,5	2,5	2,5	2,5	2,5	2,5	2,5	2,5	2,5	2,5
1,5	2,5	10	2,5	2,5	2,5	2,5	2,5	2,5	2,5
2,5	2,5	2,5	2,5	10	2,5	10	10	2,5	10
2,5	2,5	10	2,5	10	2,5	2,5	2,5	2,5	2,5
2,5	2,6	2,5	2,5	2,5	2,6	2,5	2,5	2,5	2,5
2,5	2,5	10	2,5	10	2,5	2,5	2,5	2,5	2,5
2,5	2,5	10	2,5	10	2,5	2,5	2,5	2,5	2,5
1,5	1,5	10	2,5	2,5	2,5	2,5	2,5	2,5	2,5
2,5	1,5	10	10	10	10	10	10	10	10
1,6	1,9	10	1,9	10	10	1,9	10	10	10
1,5	1,5	1,5	1,5	1,5	1,5	1,5	1,5	1,5	1,5
1,8	10	1,7	1,6	1,7	10	1,7	1,6	1,7	10
1,5	1,6	10	1,6	1,6	1,6	1,5	1,5	1,5	1,5
2,5	2,9	2,6	2,5	2,5	2,5	2,5	2,5	2,5	2,5
2,6	2,6	2,6	2,6	10	2,6	2,6	2,6	2,6	2,6
10	10	1,9	1,8	10	1,9	10	10	10	1,9
1,5	1,5	10	1,5	10	1,5	1,5	10	10	10
1,5	2,5	1,8	1,8	1,8	10	2,9	2,8	1,9	2,8
1,6	1,7	10	10	10	10	10	1,5	1,7	1,5
10	1,9	10	1,8	1,9	1,9	10	10	1,9	10
2,5	2,6	10	2,6	10	2,6	10	10	2,6	10
1,8	1,8	10	1,5	1,5	1,5	1,5	1,5	1,5	1,5
1,7	1,5	10	10	10	1,6	1,6	10	1,5	1,6
1,5	1,5	1,5	1,5	1,5	1,5	10	1,5	1,5	1,5
1,5	1,5	10	1,5	10	2,5	1,5	1,5	1,5	1,5
1,5	1,5	10	1,5	10	10	10	10	1,5	10
1,5	1,5	10	1,5	10	1,5	1,5	1,5	10	10
1,8	1,9	10	1,9	10	1,9	10	1,9	10	10

10	1,9	1,5	10	1,5	10	10	1,9	10	10
2,5	1,9	10	2,8	10	10	10	10	10	10
2,6	2,6	10	2,6	10	2,6	3,6	2,6	2,6	2,6
1,5	1,5	10	1,5	10	10	1,5	1,5	1,5	1,5
1,7	10	10	10	2,9	1,7	1,5	1,5	1,5	1,5
10	1,9	1,9	1,5	1,5	10	1,9	1,5	1,5	1,5
1,5	1,9	10	1,5	10	1,9	10	10	1,5	10
1,8	10	10	10	1,5	1,9	1,9	1,9	1,9	1,5
1,5	10	10	10	10	1,5	1,5	1,5	1,5	1,8
1,8	1,9	10	1,5	10	10	1,9	1,9	1,9	10
1,5	10	10	1,9	1,9	1,9	10	10	1,9	10
1,5	1,9	10	1,8	10	1,9	1,9	10	1,9	10
1,5	1,9	1,9	1,5	10	1,9	1,9	1,9	1,9	10
1,5	1,5	10	2,5	10	2,5	1,5	10	10	10
10	1,9	10	1,9	1,9	1,9	1,9	10	10	10
1,8	1,9	1,9	1,5	10	1,9	1,9	10	1,8	1,8
1,5	10	10	10	10	10	1,9	1,9	1,9	1,8
1,7	2,7	1,5	1,6	2,7	1,7	1,6	1,5	1,5	2,7
1,5	1,9	10	1,5	10	1,5	1,5	1,5	1,5	10
1,5	10	10	10	10	10	10	10	10	10
2,5	2,6	1,9	2,6	10	2,6	2,6	2,6	2,5	2,5
2,5	2,6	10	2,5	2,5	2,9	2,9	2,5	2,5	2,5
2,5	2,9	10	2,5	2,5	2,9	2,9	2,5	2,5	2,5
1,5	1,9	10	1,5	1,5	1,5	1,5	1,5	1,5	1,5
1,5	1,9	10	1,5	1,5	1,5	1,5	1,5	1,5	1,5
1,5	1,5	10	1,5	10	10	1,9	1,9	1,9	10
1,5	1,5	10	1,5	10	1,5	1,5	10	10	10
10	10	10	1,8	10	10	1,9	1,9	1,9	10
3,6	3,6	2,6	2,6	2,6	2,6	4,6	2,6	2,5	2,6
1,5	10	10	1,6	10	10	10	10	10	10
1,5	1,5	1,5	1,9	10	1,9	1,9	10	10	10
1,5	1,5	1,5	1,9	10	1,9	1,9	10	10	10
1,5	10	10	1,5	10	10	10	10	1,5	10
1,7	10	10	1,5	1,5	1,6	1,6	1,7	1,6	1,6
1,5	10	10	10	10	10	10	10	10	10
1,5	2,9	2,9	10	10	2,6	2,6	2,5	2,5	2,5
2,5	1,9	2,5	10	10	10	2,6	2,9	10	10
1,5	1,9	10	1,9	10	10	1,9	10	1,9	1,9
10	10	1,5	1,5	1,8	10	2,9	1,9	1,9	1,5
1,5	1,5	10	1,5	10	10	1,5	1,5	10	10
1,5	1,9	10	1,5	1,5	1,5	1,5	1,5	1,5	1,5
1,5	1,9	10	1,5	1,5	2,9	2,9	1,9	1,9	1,5
1,5	1,9	10	1,5	1,5	1,9	1,9	1,9	1,9	1,5
1,5	1,9	10	1,5	10	1,9	1,9	1,9	1,9	1,9
1,5	1,9	10	1,5	10	1,9	1,9	1,9	1,9	1,5
1,5	1,9	10	1,5	10	1,9	1,9	1,9	1,9	1,5
2,5	10	10	1,5	1,5	2,6	2,5	2,5	2,5	10

1,8	1,9	10	1,5	10	10	10	10	10	10
1,5	10	10	10	10	1,9	10	10	10	10
1,5	10	10	10	10	1,6	1,6	1,7	1,7	1,6
1,8	10	10	10	10	10	1,9	1,9	1,9	1,8
1,5	1,5	1,9	1,5	10	1,5	1,5	1,5	1,5	1,5
10	1,6	10	2,5	10	2,6	2,5	2,5	10	2,5
2,6	2,5	2,5	2,5	2,5	2,6	2,5	2,5	2,6	2,5
1,5	1,5	1,5	1,5	1,5	1,5	1,5	1,5	1,5	1,5
1,5	1,5	10	1,5	1,5	1,5	1,5	1,5	1,5	1,5
1,5	1,9	1,9	1,9	19	2,9	2,9	10	10	10
1,8	10	10	1,8	10	10	1,9	1,9	1,9	10
1,5	1,9	10	10	1,9	10	10	1,9	10	1,9
1,5	1,9	10	1,9	10	10	10	10	10	10
10	10	10	10	10	10	10	10	10	10
1,8	10	10	1,8	10	10	1,9	1,9	1,9	10
2,5	2,6	2,6	2,6	2,6	2,6	2,6	2,6	2,6	2,6
1,8	1,8	10	1,8	10	1,8	1,8	1,8	1,8	1,8
1,5	1,5	10	1,5	10	10	10	10	1,5	10
1,5	10	10	10	10	10	10	10	10	10
1,5	1,9	10	1,5	10	1,9	10	10	1,9	10
2,6	2,6	2,6	2,6	2,6	2,6	2,6	2,6	2,6	2,6
1,5	1,9	1,9	10	10	1,5	1,5	10	1,5	10
1,6	1,5	1,5	1,5	1,5	1,5	1,5	1,6	1,6	1,6
1,6	1,5	1,5	1,5	1,5	1,5	1,5	1,6	1,6	1,6
1,6	1,5	1,5	1,5	1,5	1,5	1,5	1,6	1,6	1,5
2,5	1,9	10	2,9	10	2,9	2,9	10	10	10
1,8	1,8	1,8	1,8	1,8	1,8	1,8	1,8	1,8	1,8
1,5	1,5	10	1,5	10	10	10	10	10	10
1,5	1,9	10	10	1,9	10	10	1,9	10	1,9
2,5	2,6	2,6	2,6	2,6	2,6	2,6	2,6	2,6	2,6
1,5	10	10	10	10	1,7	1,7	1,7	1,7	1,7
1,5	10	10	10	10	10	10	10	10	10
2,5	2,6	10	2,6	10	2,5	2,5	2,5	2,6	2,6
1,5	1,9	10	1,9	1,9	1,9	1,9	10	1,8	1,8
1,5	10	10	1,5	1,5	1,5	1,5	1,5	1,5	1,5
1,8	10	10	2,6	1,8	1,8	1,8	1,7	1,7	1,7
1,6	10	10	1,8	1,8	1,8	1,8	1,8	1,8	1,8
2,5	10	10	10	10	10	1,9	10	10	10
1,6	1,9	1,9	1,9	1,9	1,9	1,9	1,9	1,9	1,9
1,7	1,8	10	1,8	10	2,8	2,8	2,8	2,8	2,8
1,5	10	10	10	10	1,5	1,5	1,5	1,5	1,5
1,5	10	10	10	10	10	10	10	10	10
1,8	2,8	10	1,5	10	10	1,8	1,8	1,8	10
1,6	10	10	10	10	10	10	10	10	10
2,5	2,6	2,6	2,5	2,5	2,6	2,5	2,6	2,5	2,6
1,5	1,5	10	1,5	10	10	10	10	10	10
2,5	2,6	10	2,6	10	2,6	10	10	2,6	10

10	1,9	10	1,9	10	1,9	1,9	10	1,9	10
1,5	1,9	10	10	10	1,9	10	2,9	10	10
2,5	2,5	10	2,5	10	10	10	10	10	10
2,5	2,5	10	2,5	10	10	10	10	10	10
1,8	2,5	2,5	1,5	10	1,5	1,5	1,5	1,5	1,5
1,5	1,5	10	1,5	10	1,5	1,5	1,5	1,5	1,5
2,5	2,6	10	2,6	10	2,6	2,5	2,5	2,5	10
1,5	1,5	10	1,5	10	1,5	1,5	1,5	1,5	10
1,5	10	10	10	10	10	10	10	10	10
1,5	1,5	10	1,5	1,5	1,5	1,5	1,5	1,5	1,5
3,5	2,9	10	2,5	10	2,9	2,9	2,9	2,9	10
3,5	2,9	10	2,5	10	2,9	2,9	2,9	2,9	10
1,5	1,9	1,9	1,5	1,9	1,9	10	10	1,9	10
10	1,5	1,5	1,5	1,5	1,6	1,5	1,5	1,5	1,5
1,5	10	10	10	1,5	1,5	1,5	1,5	10	1,4
1,5	10	10	10	10	10	10	10	10	10
1,5	1,5	1,5	1,5	1,5	10	10	1,5	1,5	1,5
1,5	1,5	10	1,5	1,5	10	10	10	10	10
1,5	2,6	10	2,6	10	2,6	2,6	2,5	2,5	2,5
1,6	10	10	1,6	1,6	1,6	1,4	10	10	10
1,5	1,5	1,9	10	1,9	10	10	10	10	1,9
1,5	1,9	1,9	1,5	1,5	2,9	2,9	1,9	1,9	1,9
1,5	2,9	1,9	1,5	1,5	2,9	2,9	2,9	1,9	1,9
1,5	10	10	10	10	1,9	1,9	1,9	1,9	1,9
1,9	10	10	10	10	1,9	1,9	1,9	1,9	1,9
1,6	1,6	10	1,6	1,5	1,5	1,5	10	10	10
1,5	1,5	1,5	1,5	1,5	1,5	1,5	1,5	1,5	1,5
1,5	1,8	10	1,5	10	10	1,9	1,9	1,9	1,9
1,5	1,5	10	1,5	1,5	10	10	10	10	1,9
2,5	2,5	10	2,5	10	2,5	2,5	2,5	2,6	2,5
1,5	1,9	10	10	10	2,9	10	10	10	10
1,5	10	10	1,5	1,9	1,9	10	1,9	1,9	10
1,5	10	10	1,6	10	1,5	1,5	10	10	10
2,5	2,5	2,5	2,5	2,5	2,5	2,5	2,6	2,5	2,5
1,5	1,5	10	1,5	10	10	10	10	10	10
1,5	1,9	10	1,5	1,9	1,9	10	10	10	10
10	1,9	10	1,5	1,9	1,9	10	10	1,9	10
1,5	1,9	1,9	1,9	10	1,9	10	1,9	10	10
1,5	1,5	10	1,5	10	10	10	10	10	10
1,5	1,5	10	1,5	10	10	10	10	10	10
1,5	10	1,9	1,5	10	1,9	10	10	1,9	10
1,5	1,9	10	1,5	10	1,5	1,5	1,5	1,5	1,5
1,5	1,5	10	1,5	10	1,8	10	10	1,5	10
1,5	1,5	10	1,5	1,5	1,6	1,6	1,5	1,5	1,5
1,5	1,5	1,5	1,5	1,5	1,5	1,5	1,5	1,5	1,5
1,5	1,9	10	1,9	10	10	10	1,9	10	10
1,8	1,5	10	1,5	10	10	10	10	10	10

1,5	1,5	10	1,5	10	10	10	10	10	10
10	10	10	1,5	1,9	10	10	1,9	1,9	10
1,5	1,9	1,9	1,5	10	1,9	10	1,9	1,9	10
2,5	2,5	2,5	2,6	2,5	2,6	2,5	2,5	2,5	2,5
2,5	2,5	2,5	2,6	2,5	2,6	2,5	2,5	2,5	2,5
1,8	10	10	1,5	1,9	1,9	10	10	10	10
1,5	10	10	10	10	1,9	1,9	10	10	10
1,5	1,9	10	10	10	1,9	1,9	10	10	10
1,5	10	1,5	1,6	1,5	1,5	1,5	10	10	10
1,8	1,5	10	1,5	1,5	10	10	10	1,5	10
10	1,5	10	1,5	10	10	10	10	10	10
1,5	1,9	1,9	1,5	1,5	1,9	1,9	1,9	1,9	1,9
10	1,5	10	1,5	10	10	10	1,9	1,9	10
10	10	10	1,5	1,9	10	10	10	1,9	1,9
1,5	10	10	10	10	10	10	10	10	10
1,5	10	1,9	1,5	1,5	1,9	10	10	1,9	10
1,5	10	10	10	10	10	10	10	10	10
1,5	10	10	1,5	1,9	1,9	10	10	10	10
1,5	10	10	1,5	1,9	10	10	1,9	1,9	10
1,5	10	10	10	10	10	1,5	1,9	1,9	1,5
1,6	1,9	10	1,5	1,5	1,5	1,5	10	1,5	1,5
1,5	10	10	10	10	10	10	10	10	10
1,5	10	10	10	10	10	10	10	10	10
1,5	2,9	10	2,9	10	10	10	10	10	10
10	1,9	1,9	1,5	1,5	1,9	1,9	10	1,9	10
1,5	1,5	10	1,5	10	1,5	1,5	1,5	1,5	1,5
2,5	2,5	10	2,5	10	2,5	2,5	2,5	2,5	10
1,5	10	10	1,5	1,9	10	10	1,9	1,9	10
1,5	1,9	1,9	1,9	1,9	1,9	10	1,9	10	10
1,8	1,9	10	1,5	10	1,9	1,9	10	1,9	10
10	1,9	10	2,5	2,5	1,9	1,9	1,9	1,9	1,9
1,5	1,9	1,6	1,6	1,6	1,5	1,5	10	10	10
1,5	1,5	10	1,5	1,5	1,5	1,5	1,5	1,5	1,5
1,5	1,9	1,9	1,5	10	1,9	1,9	1,9	1,9	1,9
1,5	1,5	10	1,5	1,5	1,9	1,9	10	1,9	10
10	1,9	1,9	1,5	10	1,9	10	1,9	1,9	10
1,5	10	10	10	10	10	10	10	10	10
1,5	1,9	10	1,5	10	1,9	10	10	1,9	10
10	1,5	10	1,5	1,9	1,9	10	1,9	10	10
1,5	1,5	10	1,5	10	10	1,5	10	10	10
1,5	10	10	10	10	1,9	1,9	1,9	1,9	1,9
1,8	1,9	1,9	2,5	2,5	1,6	1,6	1,5	1,5	1,5
1,5	1,9	10	1,5	10	1,9	1,9	1,5	1,5	1,5
10	1,9	10	2,9	10	10	2,9	10	10	10
1,5	1,9	10	1,8	10	1,9	1,8	1,8	1,8	1,8
1,8	10	10	1,8	1,8	1,8	1,8	1,5	1,5	1,5
2,5	2,5	10	2,6	2,5	10	10	10	10	10

2,5	2,5	10	2,6	2,5	10	10	10	10	10
10	1,9	10	1,5	10	1,9	10	10	10	10
1,5	1,9	1,9	1,5	1,5	1,9	1,9	2,9	2,9	1,5
1,5	10	10	1,5	1,9	1,9	10	10	1,9	10
1,5	1,9	10	1,5	10	1,9	10	10	1,9	1,9
1,5	1,9	10	1,5	1,9	1,9	10	1,9	1,9	10
1,5	10	10	1,5	1,5	10	10	10	1,9	10
10	10	10	1,5	1,9	1,9	10	10	10	10
1,5	1,9	10	1,5	1,9	10	10	1,9	1,9	10
10	1,9	10	1,5	1,9	1,9	10	10	10	10
1,5	1,5	10	1,5	1,5	10	10	10	10	10
1,5	1,5	1,5	1,5	1,5	1,5	1,5	1,5	1,5	1,5
1,5	1,8	10	1,9	10	10	1,9	10	10	1,9
2,5	2,8	10	1,9	10	10	1,9	10	10	1,9
1,5	1,9	10	1,9	10	1,9	1,9	10	10	10
1,7	1,7	10	1,6	1,6	1,9	1,9	1,9	1,9	1,9
2,5	2,5	10	1,5	2,5	1,5	2,5	2,5	2,5	2,5
2,5	2,5	10	2,5	2,5	2,5	2,5	2,5	2,5	2,5
1,5	1,9	10	1,9	1,9	1,9	1,9	10	10	10
1,5	1,9	1,9	1,8	1,8	1,9	1,9	1,9	1,9	1,8
1,5	2,9	2,9	2,9	2,9	1,9	2,9	2,9	10	10
1,5	10	10	10	10	10	10	10	10	10
1,5	10	10	10	10	10	1,9	1,8	1,8	1,5
1,5	1,9	1,9	1,9	1,9	1,9	1,9	1,9	1,9	1,9
1,5	1,9	1,9	1,9	1,9	1,9	1,9	10	10	10
1,5	1,5	10	1,5	1,5	1,5	10	1,5	1,5	1,5
1,5	1,9	10	1,9	10	1,9	1,9	10	10	1,9
1,5	1,9	10	1,5	10	1,9	1,9	1,9	1,9	1,9
1,5	1,9	10	1,9	1,9	1,9	1,9	10	10	10
1,5	1,9	10	1,9	10	1,9	1,9	10	1,9	10
1,5	1,5	10	10	10	10	10	10	10	10
2,8	1,9	1,9	1,9	10	10	1,9	10	1,9	10
1,5	10	1,9	1,5	10	1,9	10	1,9	10	10
1,5	1,5	1,5	1,5	1,5	1,5	1,5	1,5	1,5	1,5
2,5	2,5	10	2,5	10	2,5	2,5	2,5	2,5	2,5
1,5	10	10	1,5	1,5	10	10	10	10	10
1,8	1,9	1,9	1,9	1,9	1,9	1,9	1,9	1,9	10
10	1,9	1,9	1,5	10	1,9	10	1,9	10	10
1,6	1,6	10	10	10	1,7	10	10	10	10
1,5	1,9	1,8	1,5	1,5	1,9	1,9	1,9	1,9	1,5
1,5	1,5	10	1,9	10	1,9	1,9	1,9	10	10
1,5	1,9	1,9	10	1,9	1,9	1,9	1,9	1,9	10
1,5	1,9	10	10	10	1,9	1,9	10	10	10
1,5	1,9	10	10	10	1,9	1,9	10	10	10
10	10	10	10	10	10	10	10	10	10
1,5	10	10	10	10	10	10	10	1,5	10
1,5	1,5	1,4	1,5	1,5	1,5	10	10	10	10

1,5	1,5	10	1,5	10	10	10	10	1,5	1,5
10	1,4	10	1,6	1,5	1,5	10	10	10	10
10	1,5	10	1,5	10	1,5	1,5	1,5	1,6	1,6
1,5	1,9	1,9	1,8	1,8	1,9	1,9	1,9	1,9	1,8
1,5	1,5	1,5	1,5	1,5	1,5	1,5	10	10	10
1,5	10	10	10	10	10	10	10	10	10
1,5	10	10	10	10	10	10	10	10	10
1,6	1,5	1,5	1,6	1,7	1,6	10	10	10	10
10	1,5	1,5	1,5	1,5	1,5	1,5	10	10	10
10	1,5	1,5	1,5	1,5	1,5	1,5	10	10	10
1,5	10	10	10	10	10	10	10	10	10
1,5	1,5	1,5	1,5	1,5	1,5	1,5	10	10	10
1,5	10	10	10	10	10	10	10	10	10
10	1,5	1,5	1,6	1,6	1,7	1,6	10	10	10
1,5	1,9	1,9	1,8	1,8	1,9	1,9	1,9	1,9	1,8
1,5	1,5	1,5	10	10	10	10	10	10	10
1,5	1,9	10	1,8	10	1,9	1,9	1,9	1,9	10
1,5	1,9	1,9	1,9	10	1,9	1,9	1,9	10	10
1,5	1,6	1,6	1,6	1,6	1,6	1,6	10	10	10
1,5	1,5	1,5	1,6	1,6	10	10	10	10	10
1,5	1,9	10	2,9	2,9	2,9	2,9	10	10	1,9
1,5	1,5	10	1,8	10	10	10	10	1,9	10
1,8	1,9	10	1,8	1,8	1,9	1,9	1,9	1,9	1,8
1,5	10	10	1,8	1,8	1,9	1,9	10	1,9	10
1,5	1,5	1,5	1,5	1,5	1,5	1,5	1,5	1,5	1,5
1,5	1,5	1,5	1,5	1,5	1,5	1,5	1,5	1,5	1,5
1,5	1,9	1,9	1,9	10	10	10	10	1,9	10
10	1,5	1,5	1,5	1,5	1,5	10	10	1,5	10
1,8	1,9	1,9	1,8	1,8	1,9	1,9	1,9	1,9	1,9
1,5	1,5	1,5	1,5	10	1,5	10	10	1,5	10
10	10	1,5	1,5	10	10	10	10	1,9	1,9
1,5	10	10	1,5	1,6	1,5	10	10	10	10
1,5	10	10	1,5	10	10	10	10	1,9	1,9
10	1,5	1,5	1,5	1,5	1,5	1,5	1,5	1,5	1,5
1,5	10	10	1,8	10	1,9	1,9	10	1,9	10
10	10	10	10	10	1,9	10	10	10	1,9
1,5	1,9	10	1,9	10	1,9	1,9	1,9	1,9	10
1,5	10	10	10	10	1,9	10	10	1,9	10
1,5	1,9	1,9	1,8	1,8	1,9	1,9	1,9	1,9	1,8
1,5	1,9	1,9	1,8	1,8	1,8	1,8	1,9	1,9	1,9
1,5	1,9	1,9	1,5	1,9	10	10	10	10	1,9
1,5	1,9	10	1,5	1,9	1,9	10	10	10	10
1,5	1,5	1,5	1,5	1,5	1,5	1,5	1,5	1,5	1,5
1,5	1,9	10	1,5	10	10	10	10	10	1,9
1,5	1,5	1,5	1,5	1,5	1,5	1,5	1,5	1,5	1,5
1,5	1,9	10	10	10	10	10	10	1,9	10
1,5	1,9	10	1,8	1,8	1,9	1,9	1,9	1,9	1,8

10	1,9	10	1,8	10	1,9	1,9	1,9	1,9	1,8
1,5	1,5	1,5	1,5	1,5	1,5	1,5	1,5	1,5	10
1,5	10	10	10	10	10	10	10	1,5	1,5
1,6	1,5	10	1,6	10	1,5	1,5	10	10	10
1,5	1,9	10	10	10	10	10	1,9	10	10
1,7	10	10	10	10	1,9	1,9	1,9	1,9	1,9
10	1,9	1,9	1,8	1,8	1,9	1,9	1,9	1,9	1,8
10	1,9	10	1,8	1,8	1,9	1,9	1,9	1,9	1,5
10	10	10	1,5	1,5	10	10	10	1,9	1,9
2,6	1,9	1,6	10	10	1,6	1,6	10	10	10
1,5	1,9	10	1,8	10	10	1,9	1,9	1,9	10
10	1,9	10	10	10	1,9	1,9	10	10	1,9
1,5	1,9	1,9	1,5	1,5	1,9	1,9	1,9	1,9	1,5
10	1,9	10	1,8	1,8	1,9	1,9	1,9	1,9	1,8
10	1,9	10	1,8	1,8	1,9	1,9	1,9	1,9	1,8
10	1,9	1,9	1,5	1,5	1,9	1,9	1,9	1,9	1,5
10	1,9	1,9	1,5	1,5	1,9	1,9	1,9	1,9	1,5
1,5	1,9	10	1,8	10	1,9	1,9	1,9	1,9	1,8
1,5	1,9	10	10	10	10	10	1,9	10	10
10	1,5	1,5	1,6	1,6	1,6	1,6	1,6	1,5	1,5
1,6	1,6	1,5	10	10	1,6	1,6	10	10	10
1,5	1,9	10	1,8	10	1,9	1,9	1,9	1,9	10
10	1,9	1,9	1,8	1,8	1,9	1,9	1,9	1,9	1,8
10	1,5	1,5	1,5	1,5	1,5	1,5	1,5	1,5	1,5
1,5	10	10	1,8	10	1,9	1,9	1,9	1,9	1,5
1,5	10	10	1,8	10	1,9	1,9	10	1,9	10
1,5	1,9	1,9	1,9	1,5	1,9	1,9	1,9	1,9	1,5
1,5	1,9	10	1,8	1,8	1,9	1,9	1,9	1,9	1,8
1,5	1,9	1,9	1,8	1,8	1,9	1,9	1,9	1,9	1,5
1,5	1,9	10	1,5	1,5	1,9	1,9	1,9	1,9	1,8
10	1,8	10	1,8	1,8	1,9	10	10	10	10
1,5	1,9	1,9	1,5	1,5	1,9	1,9	1,9	1,9	1,9
1,6	10	10	10	10	10	10	10	10	10
1,5	1,9	1,9	1,9	1,9	1,9	1,9	1,9	1,9	10
2,5	1,8	1,8	10	10	2,8	10	10	2,9	2,9
1,5	1,5	1,5	1,5	1,5	1,5	1,5	1,5	1,5	1,5
10	3,6	10	2,6	10	4,6	4,6	10	10	10
1,5	1,5	1,5	1,9	1,9	1,9	1,9	1,9	1,9	10
1,5	1,5	1,5	1,5	1,5	1,5	1,5	1,5	1,9	1,9
1,5	1,5	1,5	1,5	1,5	1,5	1,5	1,5	1,5	1,5
2,5	2,5	2,5	2,7	2,7	2,6	10	10	10	10
1,5	1,9	1,9	1,8	1,8	1,9	1,9	1,9	1,9	1,8
2,6	2,6	3,7	2,6	10	2,6	10	10	10	10
1,5	1,9	10	10	10	10	10	10	10	1,9
10	10	1,6	1,7	10	1,6	10	10	10	10
10	1,9	10	10	10	1,9	10	10	10	10
1,5	10	10	1,5	10	1,9	1,9	10	1,9	1,9

1,5	1,9	10	10	10	10	1,9	1,9	1,9	1,9
1,5	10	10	1,8	10	10	10	10	1,9	10
1,5	10	1,9	10	10	10	10	10	10	10
1,5	10	10	10	10	1,9	10	10	1,9	10
1,5	10	10	10	10	1,9	10	10	1,9	10
1,5	10	10	10	10	1,9	10	10	1,9	10
1,5	1,9	10	1,5	1,5	1,9	1,9	1,9	1,9	1,8
1,5	1,9	1,9	1,5	1,5	1,9	1,9	1,9	1,9	1,5
1,5	1,9	10	1,5	10	1,5	1,5	1,5	1,5	1,5
1,5	1,9	10	10	10	10	10	10	10	10
1,5	1,9	10	10	10	10	1,9	1,9	10	10
1,5	10	10	10	10	1,9	1,9	1,9	10	1,9
10	10	10	1,5	1,5	1,9	1,9	1,9	1,9	10
1,5	10	10	10	10	10	10	10	10	10
10	10	10	1,5	1,5	1,9	1,9	1,9	1,9	10
10	10	10	10	10	1,9	1,9	10	10	10
1,5	10	10	10	10	10	10	10	10	10
1,5	1,9	10	10	10	10	10	10	10	1,9
1,5	1,5	10	1,5	1,5	1,5	1,5	1,5	1,5	1,5
1,5	10	10	10	10	10	10	10	10	10
1,5	10	10	10	10	1,9	1,9	1,9	1,9	10
10	1,9	10	1,9	1,5	10	10	10	10	10
1,5	1,9	10	10	10	1,9	10	10	1,9	10
1,5	1,9	10	1,8	1,8	1,9	1,9	1,9	1,9	1,8
1,5	10	10	1,5	10	1,9	10	10	10	1,9
1,5	1,9	10	1,5	10	1,9	10	10	1,9	10
1,5	1,6	1,6	1,6	1,6	1,6	1,6	1,6	1,6	1,6
1,5	1,9	10	10	10	1,9	10	10	1,9	10
2,5	1,9	10	1,9	10	1,9	10	10	1,9	10
2,5	1,9	10	1,9	10	1,9	10	10	1,9	10
1,5	1,6	1,6	1,6	1,6	1,6	1,6	1,6	1,6	1,6
1,5	1,9	1,9	1,8	1,8	1,9	1,9	1,9	1,9	1,8
1,5	1,5	1,5	1,5	1,5	1,5	1,5	1,5	1,5	10
1,5	1,9	1,9	1,8	1,8	1,9	1,9	1,9	1,9	1,8
1,5	10	10	10	10	1,9	10	10	10	10
1,5	1,8	10	1,8	10	1,9	1,9	1,9	1,9	1,9
10	1,9	10	10	10	10	10	10	1,9	10
10	1,9	10	10	10	10	10	10	1,9	10
1,5	1,5	1,5	1,5	1,5	1,5	1,5	1,5	1,5	1,5
1,5	1,5	1,5	10	10	10	10	10	10	10
1,5	1,5	1,5	1,5	1,5	1,5	1,5	1,5	1,5	1,5
1,5	1,5	1,5	1,6	1,6	1,6	1,6	1,6	10	10
1,5	1,5	1,5	1,5	1,5	1,5	1,5	1,5	1,5	1,5
1,5	1,9	10	1,9	10	1,9	10	10	1,9	10
1,8	10	10	10	10	10	10	10	10	10
1,5	1,9	1,9	1,5	1,5	1,9	1,9	1,9	1,9	1,9
1,5	1,9	10	1,6	10	10	10	10	10	10

1,5	1,9	1,9	1,5	1,5	1,5	1,5	1,5	1,9	1,9
1,5	1,9	1,9	1,5	1,5	1,5	1,5	1,5	1,9	1,9
10	1,5	10	10	10	10	10	10	10	10
10	1,5	10	10	10	10	10	10	10	10
1,5	1,5	1,5	10	10	10	10	10	10	10
1,5	10	10	1,8	10	1,9	1,9	1,9	10	10
1,5	10	10	10	10	10	10	10	10	10
1,6	1,5	1,5	1,5	1,5	1,5	1,5	1,5	1,5	1,5
1,6	1,5	1,5	1,5	1,5	1,5	1,5	1,5	1,5	1,5
1,5	1,9	1,9	1,9	1,9	10	10	10	10	10
10	1,5	1,5	1,5	1,5	1,5	1,5	1,5	1,5	1,5
1,8	10	10	10	10	10	10	10	10	10
1,5	1,9	1,9	1,5	1,5	1,9	1,9	1,9	1,9	1,9
10	10	10	10	10	10	10	10	10	10
10	10	10	1,8	10	10	1,9	1,9	1,9	10
10	10	10	1,5	10	10	10	10	1,9	1,9
1,5	1,9	10	10	10	10	10	10	10	1,9
1,5	1,9	1,9	10	10	10	10	10	10	10
1,5	10	10	10	10	10	10	10	10	10
1,7	10	10	10	10	10	10	10	10	10
10	1,9	1,9	10	10	10	10	10	10	10
1,5	10	10	10	10	1,5	1,5	10	10	10
10	10	10	10	10	10	10	10	10	10
10	1,9	10	10	10	10	10	10	10	10
1,5	10	10	1,5	10	10	10	10	10	10
1,9	1,9	1,9	1,9	1,9	1,9	1,9	1,9	1,9	1,9
1,5	1,9	1,9	1,5	1,5	1,9	1,9	1,9	1,9	1,5
1,5	1,9	10	10	10	10	10	10	10	10
10	1,5	1,5	1,5	1,5	1,5	1,5	1,5	1,5	1,5
1,5	1,5	10	1,5	10	1,5	10	10	10	10
1,5	10	10	10	10	10	10	10	10	10
1,5	1,9	1,9	1,5	1,5	1,9	1,9	1,9	1,9	1,9
1,8	1,5	1,5	1,5	10	1,9	10	10	1,9	1,9
1,5	10	10	10	10	10	10	10	10	10
1,8	1,5	1,5	1,5	10	1,9	10	10	1,9	1,9
1,5	1,5	1,5	1,5	1,5	10	10	10	10	10
10	10	10	1,8	10	10	10	10	10	10
1,5	1,9	10	1,5	1,5	1,9	1,9	1,9	1,9	1,5
2,8	10	10	10	10	10	10	10	10	10
1,5	1,5	10	1,5	10	1,5	1,5	10	1,5	10
1,5	10	10	1,8	1,8	1,9	1,9	1,9	10	10
1,5	10	10	1,8	1,8	1,9	1,9	1,9	10	10
1,5	1,5	10	1,5	10	1,9	10	10	1,5	10
1,5	1,5	10	10	10	10	10	10	10	10
1,5	1,9	10	10	10	10	10	10	1,9	10
1,5	2,9	10	1,5	10	1,9	10	10	1,9	10
1,5	10	10	1,8	1,8	1,9	1,9	1,9	10	10

10	1,5	10	1,5	10	10	10	10	10	10
1,5	1,9	10	1,5	1,5	10	1,5	10	1,5	10
1,5	1,9	1,9	1,8	1,8	10	1,9	1,9	1,9	1,8
1,5	1,9	10	10	10	10	10	1,9	10	10
10	10	1,9	10	1,9	1,9	10	10	10	10
10	10	10	1,5	10	10	10	10	10	10
1,5	1,9	10	1,9	10	1,5	10	10	1,9	10
1,8	10	10	1,5	1,5	1,5	1,9	1,9	1,9	1,9
1,5	1,9	10	1,8	10	10	10	10	10	10
10	1,5	1,5	1,5	1,5	1,5	1,5	1,5	1,5	1,5
1,6	1,9	10	10	10	10	10	10	10	10
1,5	1,5	1,5	1,5	1,5	1,5	1,5	1,5	1,5	1,5
1,5	1,9	10	1,8	10	10	10	10	1,9	10
1,5	1,9	10	1,8	10	10	10	10	10	10
1,5	1,9	1,9	1,5	10	1,9	1,9	1,9	1,9	1,5
10	10	10	10	10	10	10	10	10	10
2,5	1,9	1,9	1,5	1,5	1,9	1,9	1,9	1,9	10
2,5	1,9	1,9	1,5	1,5	1,9	1,9	1,9	1,9	10
10	10	10	1,5	1,5	1,9	1,9	1,9	1,9	1,9
1,5	1,9	1,9	1,5	1,5	1,9	1,9	1,9	2,9	1,5
1,5	10	10	10	10	10	10	10	10	10
10	10	10	10	10	10	10	10	10	10
1,5	10	10	10	10	10	10	10	10	10
1,5	1,9	1,9	1,9	10	1,9	1,9	1,9	10	10
1,5	1,9	1,9	10	10	1,9	1,9	1,9	1,9	1,9
1,5	10	10	10	10	10	10	10	10	10
1,5	1,9	1,9	10	10	1,9	1,9	1,9	1,9	1,9
10	1,5	1,5	1,5	1,5	1,5	1,5	1,5	1,5	1,5
1,5	1,9	10	10	10	1,9	1,9	1,9	1,9	10
1,5	1,5	1,5	1,5	1,5	1,5	1,5	1,5	1,5	1,5
1,5	1,9	1,9	1,8	1,8	1,9	1,9	1,9	1,9	1,8
1,5	1,9	10	1,5	10	10	1,9	10	1,9	1,9
1,5	1,9	10	10	10	1,9	1,9	10	10	10
1,5	1,9	10	10	10	10	10	10	10	1,9
1,6	1,6	1,6	1,7	1,7	1,6	1,6	1,5	10	10
1,5	1,9	10	1,8	1,8	1,8	1,8	1,9	1,9	1,8
1,5	1,5	10	1,5	10	1,5	1,5	10	1,5	10
1,6	1,6	1,6	1,6	1,7	1,7	1,6	10	10	10
10	1,5	10	1,9	10	1,9	10	10	10	10
1,5	1,9	1,9	1,8	1,8	1,9	1,9	1,9	1,9	1,5
1,5	1,9	10	1,5	10	10	10	10	10	1,9
10	1,9	1,9	1,9	1,9	1,9	1,9	1,9	1,9	1,9
1,9	2,9	2,9	1,9	1,9	1,9	1,9	1,9	1,9	1,9
1,5	1,5	1,5	1,5	1,5	1,5	1,5	1,5	1,5	1,5
1,5	10	10	1,9	1,9	1,9	1,9	10	1,9	1,9
1,5	1,9	10	1,8	10	1,9	1,9	10	1,9	10

stewed mu	stewed mu	stewed mu	boiled mu	stewed mu	stewed mu	boiled mu	stewed dri	boiled vow	stewed vo
1,8	10	10	10	10	10	10	10	10	10
1,8	10	10	10	10	10	10	10	10	10
1,5	1,5	1,5	10	10	10	10	10	1,5	1,5
10	10	10	1,9	10	10	10	10	10	10
1,5	1,5	1,5	1,5	1,5	1,5	1,5	1,5	1,5	1,5
10	10	1,8	10	1,8	10	1,8	10	10	10
10	10	10	1,9	1,9	1,9	1,9	1,9	1,9	1,5
10	10	10	1,9	10	10	10	10	10	1,5
10	10	10	10	2,9	10	10	10	10	10
10	10	1,8	1,8	10	10	10	10	1,8	10
10	10	10	10	10	10	10	10	10	2,6
2,6	2,6	2,6	10	2,6	2,6	2,6	2,6	10	10
2,5	10	2,5	2,5	2,5	2,5	10	10	2,5	2,5
1,8	10	10	1,8	10	10	10	10	1,9	10
1,9	1,9	1,9	1,9	1,9	1,9	10	10	10	1,9
10	10	10	10	10	10	10	10	10	10
10	10	10	10	10	10	10	10	10	10
2,5	10	2,5	2,5	2,5	2,5	10	10	2,5	2,5
2,5	2,5	2,5	2,5	2,5	2,5	10	10	2,5	2,5
2,5	10	2,5	2,5	2,5	2,5	10	10	2,5	2,5
2,5	10	2,5	2,5	2,5	2,5	10	10	2,5	2,5
1,5	10	1,5	1,5	1,5	1,5	10	10	10	10
2,5	2,5	2,5	2,5	2,5	2,5	2,5	2,5	2,5	2,5
2,5	10	2,5	2,5	2,5	2,5	10	10	2,5	2,5
2,5	10	2,5	2,5	2,5	2,5	10	10	1,5	1,5
2,5	10	2,5	2,5	2,5	2,5	10	10	1,5	2,5
10	10	10	10	10	10	10	10	10	10
1,9	10	10	10	10	10	10	10	10	10
1,5	1,5	1,5	1,5	1,5	1,5	1,5	1,5	1,5	1,5
10	10	10	2,7	2,8	10	10	10	1,7	1,6
10	10	10	1,9	1,9	10	10	10	1,9	10
10	10	10	10	2,5	2,5	10	10	10	10
2,6	2,6	2,6	2,6	2,6	2,6	2,6	2,6	2,6	2,6
1,9	10	1,9	10	10	10	1,9	10	10	1,9
10	10	10	10	10	10	10	10	1,9	1,9
10	10	1,5	10	1,8	10	10	10	1,5	10
10	10	10	10	10	10	10	10	10	10
10	10	10	10	10	10	10	10	10	10
10	10	10	10	10	10	10	10	10	10
10	10	10	10	10	10	10	10	10	10
1,5	10	10	10	10	10	10	10	10	10
1,5	1,5	1,5	1,5	1,5	1,5	1,5	1,5	1,5	1,5
10	10	10	10	10	10	10	10	10	10
10	10	10	10	10	10	10	10	10	10
1,9	10	1,9	1,9	1,9	1,9	10	10	1,9	1,9
10	10	10	1,9	1,9	10	10	1,9	1,9	10

10	10	1,5	1,5	10	10	1,5	10	1,5	10
1,9	10	1,9	1,9	10	10	10	10	10	2,9
2,5	10	2,5	10	2,5	2,6	10	10	10	2,6
1,5	10	1,5	1,5	1,5	1,5	1,5	1,5	1,5	1,5
1,5	1,5	1,6	1,6	1,5	10	1,5	1,5	1,5	2,7
1,9	1,9	1,9	1,5	10	1,5	1,5	1,5	1,5	10
1,9	10	1,9	1,9	1,9	1,9	10	10	1,9	1,9
10	10	10	10	10	10	10	10	1,9	10
10	10	10	10	10	10	10	10	1,9	10
10	10	10	1,8	10	10	10	10	1,9	10
1,9	10	10	1,9	10	10	10	10	1,9	10
10	10	10	10	10	10	10	10	1,9	10
10	10	10	10	10	10	10	10	10	10
2,5	2,5	2,5	2,5	2,5	1,5	1,5	1,5	1,5	1,5
1,9	10	1,9	1,9	10	10	1,9	10	10	10
1,5	10	1,8	10	10	10	1,9	1,9	1,9	10
10	10	10	10	10	10	10	10	1,8	10
1,8	1,5	1,7	1,9	1,9	1,9	10	10	1,7	1,8
10	10	10	10	10	10	10	10	1,9	1,9
10	10	10	10	10	10	10	10	10	10
2,6	10	10	2,5	2,5	2,5	10	10	10	10
2,9	10	2,5	2,6	2,5	2,5	10	10	2,5	2,6
2,9	10	2,5	2,6	2,5	2,5	10	10	2,5	2,6
10	10	10	10	10	10	10	10	1,9	1,9
10	10	10	10	10	10	10	10	1,9	1,9
10	10	10	10	10	10	10	10	1,9	10
10	10	10	10	10	10	10	10	1,8	1,8
10	10	10	10	10	10	10	10	1,9	1,9
1,5	10	2,5	2,6	2,6	2,5	10	10	1,5	1,5
10	10	10	10	10	10	10	10	10	10
1,5	10	2,9	1,9	2,9	10	10	10	10	10
1,5	10	2,9	1,9	2,9	10	10	10	10	10
1,5	10	1,5	10	10	10	10	10	1,5	1,5
1,5	1,5	1,5	1,5	1,5	10	10	10	1,7	1,6
10	10	1,5	10	10	10	10	10	10	1,5
10	10	10	10	2,6	2,6	2,5	2,5	2,5	2,6
10	2,5	10	10	10	10	2,5	10	10	10
1,9	10	1,9	10	10	10	1,9	10	1,9	10
10	10	1,8	10	10	10	10	10	1,8	10
10	10	1,5	1,5	10	10	10	10	1,5	1,5
10	10	10	10	10	10	10	10	1,9	1,9
1,9	1,9	1,9	1,9	1,9	1,9	1,9	1,5	2,9	2,9
10	10	10	10	10	10	10	10	1,9	1,9
1,9	10	1,9	10	10	10	10	10	1,9	1,9
1,9	10	1,9	10	10	10	10	10	1,9	1,9
10	10	10	10	10	10	10	10	1,9	1,9
2,5	2,6	2,5	2,5	2,6	2,6	2,5	10	2,5	2,5

10	10	1,9	1,9	1,9	10	10	10	1,9	1,9
10	10	10	10	10	10	10	10	10	1,9
10	10	1,7	1,7	1,7	1,7	10	10	1,5	10
10	10	10	1,8	10	10	10	10	10	10
10	10	10	1,9	1,9	1,9	10	1,5	1,9	1,9
2,5	10	2,5	2,5	2,5	1,5	10	10	2,5	1,5
2,5	2,5	2,5	2,6	2,6	2,6	2,5	2,5	2,5	2,5
10	10	10	1,9	1,9	1,5	1,6	1,5	1,5	1,5
10	10	10	1,9	1,9	1,9	10	10	1,5	1,5
2,9	10	2,9	1,9	10	10	1,9	1,9	10	10
10	10	10	10	10	10	10	10	1,9	10
10	10	1,9	10	10	10	10	10	10	1,9
10	10	10	10	10	10	10	10	10	10
10	10	10	10	10	10	10	10	10	10
10	10	10	10	10	10	10	10	1,9	10
2,6	2,6	2,6	2,6	2,6	2,6	2,6	2,6	2,6	2,6
10	10	10	10	10	10	10	10	1,9	1,9
10	10	10	10	10	10	10	10	1,5	1,5
10	10	10	10	10	10	10	10	10	10
1,9	10	1,9	1,9	10	10	10	10	1,9	10
2,6	10	2,6	2,6	2,6	2,6	2,6	2,6	2,6	2,6
1,9	10	1,9	1,9	1,9	1,9	10	10	10	10
10	10	10	10	10	10	10	10	10	10
10	10	10	10	10	10	10	10	10	10
10	10	10	10	10	10	10	10	10	10
2,9	10	2,9	10	10	10	10	2,9	10	10
10	10	10	1,8	1,8	1,8	1,8	1,8	2,8	1,8
10	10	10	1,8	1,8	1,8	10	10	10	10
10	10	1,9	10	10	10	10	10	10	1,9
2,5	1,6	2,5	2,5	3,5	2,5	2,5	2,6	2,5	2,5
1,9	1,7	1,7	1,5	10	10	1,5	1,5	10	10
10	10	10	10	10	10	10	10	10	10
2,5	10	2,6	2,5	2,5	2,5	10	10	2,5	2,5
1,8	1,9	1,9	10	10	10	2,9	2,9	2,9	2,9
10	10	10	10	10	10	10	10	2,8	2,8
10	10	10	10	10	10	10	10	10	10
1,9	10	10	10	10	10	10	10	10	10
10	10	10	10	10	10	10	10	10	10
1,9	1,9	1,9	1,9	1,9	10	1,9	1,9	1,8	1,8
1,8	1,8	10	10	1,8	1,8	10	10	1,8	1,8
10	10	10	10	10	10	10	10	10	10
10	10	10	10	10	10	10	10	10	10
10	10	10	10	10	10	10	10	2,9	10
10	10	10	10	10	10	10	10	10	10
10	10	10	10	10	10	10	10	2,5	2,6
10	10	10	10	10	10	10	10	10	10
10	10	10	10	10	10	10	10	10	10

1,9	10	1,9	10	10	10	10	10	1,9	1,9
2,9	10	10	10	10	10	10	10	10	1,9
10	10	10	10	10	10	10	10	10	10
10	10	10	10	10	10	10	10	10	10
1,9	1,9	1,9	10	10	10	10	10	1,5	10
1,8	10	10	10	10	10	10	10	10	10
2,6	10	10	10	2,6	2,6	10	10	10	10
1,5	10	1,5	10	10	10	10	10	1,5	1,5
10	10	10	10	10	10	10	10	10	10
10	10	10	10	10	10	10	10	1,5	10
10	10	10	10	10	10	10	10	10	10
10	10	10	10	10	10	10	10	10	10
1,9	1,9	1,9	10	10	10	10	10	10	10
1,5	1,5	1,5	1,5	1,5	1,5	1,5	1,5	1,5	1,6
10	10	10	10	10	10	10	10	1,5	1,5
10	10	10	10	10	10	10	10	10	10
10	10	10	10	10	10	10	10	10	10
10	10	10	10	10	10	10	10	10	10
10	10	10	10	10	10	10	10	10	10
1,6	1,6	1,6	1,6	1,6	10	10	10	10	10
10	10	10	10	10	10	10	10	10	10
10	10	10	10	10	10	10	10	1,9	1,9
10	10	10	10	10	10	10	10	1,9	1,9
10	10	10	10	10	10	10	10	10	10
10	10	10	10	10	10	10	10	10	10
10	1,5	10	10	10	10	10	10	1,5	1,5
10	10	10	10	10	10	10	10	1,5	1,5
10	10	10	10	10	10	10	10	1,8	10
1,9	10	10	1,9	10	10	1,9	1,9	10	10
2,5	10	2,5	2,5	2,5	2,6	10	10	2,5	2,5
10	10	10	10	10	10	10	10	10	10
10	10	10	1,9	1,9	10	10	10	1,9	1,9
10	10	10	10	10	10	10	10	1,5	1,5
2,5	10	2,5	2,6	2,5	2,5	10	10	2,5	2,6
10	10	10	1,5	1,5	1,5	10	10	1,6	1,5
1,9	10	1,9	10	10	10	1,9	10	1,9	10
1,9	1,9	10	10	10	10	1,9	10	1,9	10
1,9	10	1,9	1,9	10	10	10	10	1,9	10
10	10	10	10	10	10	10	10	1,5	1,5
10	10	10	10	10	10	10	10	1,5	1,5
1,9	10	10	10	1,9	1,9	10	10	1,9	10
10	10	10	10	10	10	10	10	10	10
1,9	10	1,9	10	10	10	10	10	1,5	10
1,5	10	1,5	1,5	1,5	1,5	10	10	1,5	1,5
1,5	1,5	1,5	1,5	1,5	1,5	1,5	1,5	1,5	1,5
1,9	10	1,9	10	10	10	10	10	1,9	10
10	10	10	10	10	10	10	10	10	10

10	10	1,5	1,5	10	10	10	10	1,5	1,5
1,9	10	1,9	10	10	10	10	10	1,9	1,9
1,9	1,9	1,9	1,9	10	10	10	10	10	1,9
2,5	2,5	2,6	2,6	2,6	2,5	2,5	2,6	2,5	2,6
2,5	2,5	2,6	2,6	2,5	2,5	2,5	2,6	2,5	2,5
1,9	1,9	10	10	10	10	1,9	1,9	10	10
10	10	10	1,9	10	10	1,9	1,9	1,9	1,9
10	10	10	10	10	10	10	10	10	1,9
10	10	10	10	10	10	10	10	1,5	1,5
10	10	10	10	10	10	10	10	10	10
1,5	10	10	10	10	10	10	10	1,5	1,5
1,9	1,9	1,9	10	10	10	10	10	1,9	1,9
1,9	10	1,9	10	1,9	10	10	10	1,9	1,9
10	1,9	1,9	10	10	10	10	1,9	1,9	10
10	10	10	10	10	10	10	10	1,9	1,9
1,9	1,9	10	10	10	10	1,9	10	10	10
10	10	10	10	10	10	10	10	1,9	1,9
1,9	10	1,9	10	10	10	10	10	1,9	1,9
1,9	10	1,9	10	10	10	1,9	1,9	10	10
10	10	10	1,9	10	10	10	10	1,9	10
10	10	10	10	10	10	10	10	10	10
10	10	10	10	10	10	10	10	10	10
10	10	10	10	10	10	10	10	10	10
10	10	10	10	10	10	10	10	10	10
10	10	10	10	10	10	10	10	10	10
10	10	10	10	10	10	10	10	1,5	1,5
2,5	10	1,9	1,9	1,9	2,8	10	10	10	2,8
1,9	1,9	10	10	10	10	10	10	10	1,9
1,9	10	1,9	10	1,9	10	10	10	10	10
10	10	10	1,9	10	10	10	10	10	10
1,9	1,9	1,9	1,9	1,9	1,9	1,9	1,5	1,9	1,9
10	10	10	10	10	10	10	10	10	10
10	10	10	10	10	10	10	10	1,5	1,5
1,9	10	10	1,9	1,9	1,9	10	10	2,9	2,9
10	10	10	1,9	10	1,9	1,9	10	1,9	10
10	10	10	1,9	10	10	10	10	1,9	1,9
10	10	10	10	10	10	10	10	1,5	1,5
1,9	10	10	10	10	10	10	10	1,9	10
1,9	1,9	10	1,9	10	10	1,9	10	1,9	1,9
1,5	10	10	1,5	1,5	10	10	10	1,5	10
10	10	10	10	10	10	10	10	1,9	1,9
10	10	10	10	10	10	10	10	10	10
10	10	1,9	10	10	10	10	10	1,9	10
2,9	10	10	10	10	10	10	10	10	10
10	10	1,9	10	10	10	10	10	1,8	10
10	10	10	10	10	10	10	10	10	10
10	10	10	10	10	10	10	10	10	10

10	10	10	10	10	10	10	10	10	10
10	10	10	1,9	10	10	10	10	1,9	10
10	10	10	10	10	10	10	10	1,9	1,9
1,9	10	10	10	10	1,9	1,9	10	1,9	10
1,9	10	10	10	10	10	10	10	1,9	1,9
10	10	10	10	10	10	1,9	1,9	1,9	10
1,9	10	1,9	10	1,9	10	10	10	1,9	1,9
1,9	10	10	10	10	10	10	10	1,9	1,9
1,9	10	1,9	10	10	10	1,9	10	1,9	10
1,9	10	10	10	1,9	10	10	10	1,9	1,9
1,5	10	1,5	10	10	10	10	10	10	10
1,5	1,5	1,5	1,5	1,5	1,5	1,5	1,5	1,9	1,9
2,5	10	10	10	10	10	1,9	10	10	1,8
2,5	10	10	10	10	10	1,9	10	10	1,8
1,9	1,9	1,9	10	10	10	1,9	1,9	10	1,9
10	10	10	10	1,6	1,5	10	10	1,5	1,5
2,5	2,5	2,5	1,5	1,5	1,5	2,5	2,5	2,5	2,5
2,5	2,5	2,5	1,5	2,5	2,5	2,5	2,5	2,5	2,5
1,9	10	1,9	10	10	1,9	10	10	10	10
1,9	1,9	1,9	10	10	10	10	10	1,9	10
2,9	10	2,9	10	10	10	2,9	10	10	10
10	10	10	10	10	10	10	10	10	10
10	10	1,9	1,9	10	10	10	10	1,9	10
1,9	10	10	10	10	10	1,9	1,9	1,9	10
1,9	10	10	10	10	10	1,9	1,9	1,9	10
1,5	1,5	1,5	10	10	10	10	10	1,5	1,5
1,9	1,9	1,9	10	10	10	1,9	1,9	10	1,9
1,9	10	1,9	1,9	1,9	1,9	10	10	1,9	1,9
1,9	1,9	1,9	10	10	10	10	10	10	10
10	10	1,9	10	1,9	10	10	10	10	10
10	10	10	10	10	10	10	10	10	10
1,8	10	10	10	10	10	1,9	10	1,9	1,9
10	10	10	1,9	10	10	10	10	1,9	1,9
10	10	10	10	10	10	10	10	10	10
10	10	10	2,5	2,5	2,5	10	10	2,5	2,5
1,5	10	1,5	10	10	10	10	10	1,5	10
1,9	1,9	1,9	10	10	10	10	10	10	1,9
1,9	1,9	10	10	10	10	10	10	1,9	10
10	10	10	1,5	1,5	1,5	10	10	10	1,5
1,9	1,9	1,9	1,9	1,9	1,9	10	10	1,9	1,9
1,9	10	1,9	10	2,9	10	10	10	10	2,9
10	1,9	1,9	1,9	10	10	10	10	1,9	10
10	10	10	10	10	10	10	10	10	10
10	10	10	10	10	10	10	10	10	10
10	10	10	10	10	10	10	10	10	10
10	10	10	10	10	10	10	10	10	10
1,6	1,6	1,5	1,5	10	10	10	10	1,5	10

1,9	10	10	1,9	1,9	1,9	10	10	1,9	1,9
10	10	10	1,5	1,5	1,4	10	10	10	1,5
10	10	10	1,5	1,5	1,5	10	10	10	10
1,9	1,9	1,9	1,9	1,9	1,9	1,9	10	1,9	1,9
10	10	10	10	10	10	10	10	10	10
10	10	10	10	10	10	10	10	10	10
1,5	10	1,5	10	10	10	10	10	10	10
10	10	10	10	10	10	10	10	10	10
1,6	1,5	1,5	10	10	10	10	10	1,6	10
1,6	1,5	1,5	10	10	10	10	10	1,6	10
10	10	10	10	10	10	10	10	10	10
1,5	1,5	1,5	1,5	1,5	1,5	10	10	10	10
10	10	10	10	10	10	10	10	10	10
1,6	1,5	1,6	10	10	10	10	10	10	10
1,9	1,9	1,9	1,9	1,9	1,9	1,9	1,8	1,9	1,9
1,9	10	10	1,9	1,9	1,9	10	10	10	1,9
10	10	10	1,9	1,9	10	10	10	10	10
1,9	10	10	10	1,9	10	10	10	10	1,9
10	10	10	10	10	10	10	10	10	10
10	10	10	10	10	10	10	10	10	10
1,9	1,9	10	1,9	1,9	10	10	10	10	10
10	10	10	10	10	10	10	10	10	10
10	10	10	10	10	10	10	10	10	10
1,9	1,9	10	10	10	10	10	10	10	10
10	10	10	10	10	10	10	10	10	10
1,5	1,5	1,5	1,9	1,9	1,9	1,5	1,5	1,5	1,5
10	10	10	10	10	10	10	10	10	10
10	10	10	1,9	1,9	1,9	10	10	1,5	1,5
10	10	10	10	10	10	10	10	1,9	1,9
1,5	10	1,5	1,5	1,5	1,5	10	10	1,5	1,5
1,9	10	10	10	10	10	10	10	10	10
1,9	10	10	10	10	10	10	10	10	10
1,9	10	10	10	10	10	10	10	10	10
1,5	1,5	1,5	1,9	1,9	1,9	1,5	1,5	1,5	1,5
10	10	10	10	10	10	10	10	1,9	10
1,9	10	10	10	10	1,9	10	10	10	10
10	10	10	1,9	10	10	10	10	1,9	10
10	10	10	10	10	10	10	10	10	10
10	10	10	10	10	10	10	10	10	10
10	10	10	1,8	10	10	10	10	1,9	10
1,9	10	10	1,9	1,9	10	10	10	10	10
1,9	10	10	10	1,9	10	10	10	10	10
10	10	10	10	10	10	10	10	1,9	1,9
10	10	10	10	10	10	1,9	10	10	10
1,5	1,5	1,5	1,5	1,5	1,5	1,5	1,5	1,5	1,5
1,9	10	10	1,9	1,9	10	10	10	10	10
10	10	10	10	10	10	10	10	10	10

10	10	10	10	10	10	10	10	10	1,9
10	10	10	10	10	10	10	10	1,5	1,5
10	10	10	10	10	10	10	10	10	10
10	10	10	10	10	10	10	10	1,5	10
1,9	10	10	10	10	10	10	10	1,9	1,9
10	10	1,9	10	10	10	10	10	10	1,9
1,9	1,9	1,9	10	10	10	10	10	10	1,9
1,9	1,9	1,9	10	10	10	10	10	10	1,9
10	10	10	10	10	1,9	10	10	10	10
10	10	10	10	10	10	10	10	10	10
10	10	10	10	10	10	10	10	10	10
1,9	10	10	10	1,9	1,9	1,9	10	10	10
10	10	10	1,9	1,9	10	10	10	1,9	1,9
10	10	10	10	10	10	10	10	10	1,9
10	10	10	10	10	10	10	10	10	1,9
1,9	1,9	1,9	10	10	10	10	10	10	1,9
1,9	1,9	1,9	10	10	10	10	10	10	1,9
10	10	10	10	10	10	10	10	10	10
1,9	10	10	10	10	10	10	10	1,9	1,9
10	10	10	10	10	10	10	10	10	10
10	10	10	1,7	1,5	10	10	10	1,7	10
10	10	10	10	10	10	10	10	1,9	10
1,9	1,9	1,9	10	10	10	10	10	10	1,9
10	1,5	1,5	1,5	1,5	1,5	10	10	1,5	10
10	10	10	10	10	10	10	10	10	10
10	10	10	10	10	10	10	10	1,9	10
1,9	1,9	1,9	1,9	1,9	1,9	1,9	10	1,9	1,9
1,9	1,9	1,9	10	10	10	10	10	1,9	1,9
1,9	1,9	1,9	10	10	10	10	10	10	1,9
1,9	1,9	1,9	10	10	10	10	10	1,9	1,9
10	10	10	10	10	10	10	10	10	1,9
1,9	1,9	1,9	1,9	1,9	1,9	10	10	1,9	1,9
10	10	10	10	10	10	10	10	10	10
1,5	10	1,5	1,9	1,9	1,9	1,5	1,5	10	10
10	10	10	2,9	10	10	2,9	10	2,9	10
1,8	1,8	1,8	1,5	1,5	1,5	1,5	1,5	1,5	1,5
4,9	10	4,9	4,9	4,9	4,9	10	10	2,9	2,9
10	10	10	1,9	1,9	1,9	1,9	1,9	1,9	10
1,9	1,9	1,9	1,9	1,9	1,9	1,9	1,9	1,9	1,9
10	10	10	1,9	1,9	1,9	1,9	1,9	1,9	1,9
10	10	10	10	10	10	10	10	10	10
1,9	1,9	1,9	10	10	10	10	10	10	10
10	10	10	10	10	10	10	10	10	10
10	10	10	10	1,9	10	10	10	10	10
10	10	10	10	10	10	10	10	10	10
1,9	10	10	10	10	10	10	1,9	10	1,9
10	10	10	10	10	10	10	1,9	10	10

10	10	10	10	10	10	10	10	1,9	10
1,9	10	10	10	10	1,6	10	10	10	10
10	10	10	10	10	10	10	10	10	10
10	10	10	10	10	10	10	1,9	10	10
10	10	10	10	10	10	10	1,9	10	10
10	10	10	10	10	10	10	1,9	10	10
1,9	1,9	1,9	1,9	1,9	1,9	1,9	1,8	1,9	1,9
1,9	1,9	1,9	1,9	1,9	1,9	1,9	1,5	1,9	1,9
10	10	10	1,5	1,5	1,5	10	10	1,5	1,5
1,9	10	1,9	1,9	1,9	1,9	10	10	10	10
1,9	10	10	10	10	10	10	10	10	10
1,9	10	10	1,9	10	10	10	1,9	10	10
10	10	10	10	10	10	10	10	10	10
1,5	10	10	10	10	10	10	10	10	10
10	10	10	10	10	10	10	10	10	10
1,9	10	1,9	10	10	10	10	10	1,9	1,9
1,9	10	10	10	10	10	10	10	10	10
1,9	10	10	10	1,9	10	10	10	10	10
1,9	1,9	1,9	1,9	1,9	1,9	1,5	1,5	1,5	1,5
1,9	10	10	10	10	10	10	10	10	10
10	10	10	10	10	10	10	10	10	10
1,9	10	10	10	1,9	10	10	10	10	10
10	10	10	10	10	10	10	10	1,9	1,9
1,9	10	1,9	1,9	1,9	1,9	10	10	10	1,9
10	10	10	10	10	10	10	1,9	10	10
10	10	10	1,9	1,9	1,9	10	10	1,9	1,9
10	10	10	10	10	10	10	10	1,6	1,6
1,9	10	1,9	1,9	1,9	1,9	10	10	1,9	1,9
1,9	10	1,9	1,9	1,9	1,9	10	10	1,9	1,9
1,9	10	1,9	1,9	1,9	1,9	10	10	1,9	1,9
1,6	1,6	1,6	1,6	1,6	1,6	1,6	1,6	1,6	1,6
1,9	1,9	10	10	10	10	10	10	10	1,9
1,9	10	1,9	1,9	1,9	10	10	10	10	10
1,9	10	10	1,9	10	10	10	10	1,9	10
10	10	10	10	10	10	10	10	10	10
10	10	10	10	10	10	10	10	1,9	1,9
1,9	10	1,9	1,9	1,9	1,9	10	10	1,9	1,9
1,9	10	1,9	1,9	1,9	1,9	10	10	1,9	1,9
10	10	10	10	10	10	10	10	10	10
10	10	10	1,5	1,5	1,5	1,5	1,5	1,5	1,5
1,5	1,5	1,5	10	10	10	10	10	1,5	1,5
10	10	10	10	10	10	10	10	1,6	1,6
10	10	10	10	10	10	10	10	10	10
1,9	10	10	10	10	10	10	10	10	10
1,8	10	10	10	10	10	10	10	10	10
1,9	1,9	10	10	10	10	10	10	1,9	1,9
1,9	10	10	10	10	10	10	10	10	10

10	10	10	10	10	10	10	10	1,9	1,9
10	10	10	10	10	10	10	10	1,9	1,9
10	10	10	10	10	10	10	10	10	10
10	10	10	10	10	10	10	10	10	10
10	10	10	10	10	10	10	10	10	10
10	10	10	10	10	10	10	10	1,9	10
10	10	10	10	10	10	10	10	10	10
1,5	1,5	1,5	1,9	1,9	1,9	1,5	1,5	1,5	1,5
1,5	1,5	1,5	1,9	1,9	1,9	1,5	1,5	1,5	1,5
10	10	10	10	10	10	10	10	10	10
1,5	1,5	1,9	1,9	1,9	1,9	1,9	1,9	1,9	1,9
1,9	10	10	10	10	10	10	10	10	10
1,9	1,9	1,9	1,9	1,9	1,9	1,9	1,5	1,9	1,9
10	10	10	10	10	10	10	10	10	10
10	10	10	10	10	10	10	10	1,9	10
10	10	10	1,9	10	10	10	10	10	10
10	10	10	10	10	10	10	10	10	10
10	10	10	1,9	1,9	1,9	1,9	1,9	1,9	1,9
10	10	10	10	10	10	10	10	1,9	10
10	10	10	10	10	10	10	10	10	10
10	10	10	10	10	10	10	10	10	10
1,5	10	10	10	10	10	10	10	1,5	1,5
10	10	10	10	10	10	10	10	10	10
10	10	10	10	10	10	10	10	10	10
1,9	1,9	1,9	10	10	10	10	10	1,9	1,9
1,9	1,9	1,9	1,9	1,9	1,9	1,9	1,9	1,9	1,9
10	10	10	10	10	10	10	10	1,9	1,9
10	10	10	10	10	10	10	10	10	10
1,5	1,5	1,5	1,5	1,5	1,5	1,5	10	1,5	1,5
1,5	10	1,5	1,5	1,5	1,5	10	10	1,5	1,5
10	10	10	10	10	10	10	10	10	10
10	10	10	1,9	1,9	1,9	10	1,5	1,9	1,9
10	10	10	10	10	10	10	10	10	10
10	10	10	10	10	10	10	10	10	10
10	10	10	10	10	10	10	10	10	10
1,5	1,5	1,5	1,9	1,9	1,9	1,5	1,5	1,5	1,5
10	10	10	10	1,5	10	10	10	10	10
1,9	1,9	1,9	10	10	10	10	10	1,9	1,9
10	10	10	10	10	10	10	10	1,8	10
10	10	10	10	10	10	10	10	10	10
10	10	10	10	10	10	10	10	10	10
10	10	10	10	10	10	10	10	10	10
10	10	10	10	10	10	10	10	10	10
10	10	10	10	10	10	10	10	10	10
10	10	10	10	10	10	10	10	1,9	10
10	10	10	10	1,9	10	10	10	1,9	1,9
10	10	10	10	10	10	10	10	10	10

10	10	10	10	10	10	10	10	10	10
1,9	10	1,9	1,9	1,9	1,9	10	10	1,9	1,9
10	1,9	1,9	1,9	1,9	10	1,9	1,9	1,9	1,8
1,9	10	10	10	10	10	10	10	10	1,9
10	10	10	1,9	10	10	10	10	10	10
1,9	10	10	10	10	10	10	10	10	1,9
10	10	10	10	10	10	10	10	10	10
10	10	10	10	10	10	10	10	10	10
10	10	10	10	10	10	10	10	1,9	10
1,5	1,5	1,5	1,5	1,5	1,5	10	1,5	1,5	1,5
1,9	10	10	10	10	10	10	10	1,9	10
1,5	1,5	1,5	1,5	1,5	1,5	1,5	1,5	1,5	1,5
10	10	10	10	10	10	10	10	10	10
10	10	10	10	10	10	10	10	10	10
1,9	10	1,9	10	10	10	10	10	1,9	1,9
10	10	10	10	10	10	10	10	10	10
10	10	10	10	10	10	10	10	10	10
10	10	10	10	10	10	10	10	10	10
1,9	1,9	1,9	10	10	10	10	10	1,9	1,9
1,9	1,9	1,9	1,9	1,9	1,9	1,9	1,5	1,9	1,9
1,5	10	10	10	10	10	10	10	10	10
10	10	10	10	10	10	10	10	10	10
10	10	10	10	10	10	10	10	10	10
1,9	1,9	10	10	10	10	10	10	10	10
10	10	10	10	10	10	10	10	10	10
10	10	10	10	10	10	10	10	10	10
10	10	10	10	10	10	10	10	10	10
1,5	10	1,5	1,5	1,5	1,5	10	10	10	1,5
1,9	10	1,9	1,9	1,9	1,9	1,9	1,9	1,9	1,9
1,9	1,9	1,9	1,9	1,9	1,9	1,9	1,9	1,9	1,9
10	10	10	1,9	1,9	1,9	1,9	1,8	10	10
1,9	10	10	10	10	1,9	10	10	10	10
10	10	10	10	10	10	10	10	10	10
1,9	10	10	10	10	10	10	10	10	10
1,5	1,5	1,6	1,5	10	10	10	10	1,6	10
10	10	10	1,9	1,9	10	10	10	1,9	10
1,9	10	10	10	1,5	1,5	10	10	10	1,5
1,6	1,6	1,7	1,7	1,6	1,6	10	10	10	10
1,9	1,9	1,9	1,9	1,9	1,9	10	10	10	1,9
1,9	1,9	1,9	1,9	1,9	1,9	10	10	10	1,9
1,9	10	10	10	10	10	1,9	10	10	10
10	10	10	1,9	1,9	1,9	10	10	10	1,9
10	10	10	1,9	1,9	1,9	10	10	10	1,9
1,9	1,9	1,9	1,9	1,9	1,9	1,9	1,9	1,9	1,9
10	10	10	10	10	10	10	10	10	10
10	10	10	1,9	1,9	10	10	10	1,9	10

stewed vo	boiled vow	stewed dri	boiled fres	boiled drie	boiled drie	boiled okra	boiled okra	boiled okra	boiled okra
10	10	10	1,5	10	10	10	10	1,5	10
10	10	10	1,5	10	10	10	10	1,5	10
1,5	1,5	1,5	1,5	10	1,5	10	10	10	10
10	10	10	10	1,9	10	10	1,9	10	1,9
1,5	1,5	1,5	1,5	1,5	1,5	10	1,5	10	1,5
10	10	1,9	1,8	10	10	10	1,9	10	10
1,9	10	10	1,5	10	1,5	1,5	10	10	1,5
10	10	10	10	1,5	10	10	10	10	10
10	10	10	2,9	10	10	10	2,9	10	2,9
10	10	10	1,5	1,5	10	10	10	10	1,8
1,6	1,6	1,6	1,6	1,9	1,6	10	10	10	10
10	2,6	10	2,6	2,6	2,6	2,6	2,6	2,6	2,6
2,5	10	10	2,5	2,5	2,5	2,5	2,5	2,5	2,5
10	10	10	1,9	10	10	1,5	10	10	10
1,9	1,9	1,9	1,9	1,9	1,9	1,8	1,8	10	1,8
10	10	10	1,9	1,9	10	1,9	10	10	1,9
10	10	10	1,9	1,9	10	1,9	10	1,9	10
2,5	10	10	2,5	2,5	2,6	2,5	1,5	1,5	2,5
2,5	10	10	2,5	2,5	2,5	2,5	2,5	2,5	2,5
2,6	10	10	2,5	2,5	2,5	2,5	2,5	2,5	2,5
2,5	2,5	10	2,5	2,5	2,5	1,5	2,5	2,5	2,5
1,5	10	10	1,5	1,5	10	1,5	1,5	1,5	1,5
2,5	2,5	2,5	2,5	2,5	1,5	1,5	1,5	1,5	2,5
2,5	10	10	2,5	2,5	2,5	2,5	2,5	2,5	2,5
1,5	10	10	2,5	2,5	2,5	2,5	2,5	2,5	2,5
2,5	10	10	2,5	2,5	2,5	2,5	2,5	2,5	2,5
10	10	10	2,9	10	10	10	10	10	10
10	10	10	1,9	1,9	1,9	10	1,9	10	10
1,5	1,5	1,5	1,5	1,5	1,5	1,5	1,5	1,5	1,5
1,7	1,7	1,7	10	10	10	10	10	10	10
10	1,5	1,5	1,5	1,5	1,6	1,9	1,9	1,9	1,9
10	10	10	2,6	1,8	1,8	10	2,5	2,5	2,5
2,6	2,6	2,6	2,5	10	10	10	10	10	10
10	10	10	1,9	10	1,9	1,9	1,9	10	10
1,9	10	10	1,5	1,5	1,5	1,5	1,5	10	1,5
10	10	10	2,9	2,9	1,9	10	10	10	2,8
10	10	10	1,9	1,9	10	10	1,7	1,5	1,7
10	10	10	1,9	1,9	1,9	1,9	10	10	10
10	10	10	2,6	10	10	10	10	10	2,6
10	10	10	1,5	10	1,5	10	10	10	10
10	10	10	1,6	1,5	1,6	10	10	10	10
1,5	1,5	1,5	1,5	1,5	1,5	1,5	1,5	10	1,5
10	10	10	1,5	1,5	1,5	1,5	1,5	1,8	1,5
10	10	10	1,5	1,5	10	10	10	10	10
1,9	10	10	1,5	1,5	1,5	1,5	1,5	1,6	1,5
1,9	10	1,9	10	10	10	10	10	10	1,9

10	10	10	1,9	10	10	10	1,9	10	10
10	10	10	1,9	10	10	10	1,9	10	10
2,6	10	10	2,6	1,5	2,6	10	10	10	10
1,5	1,5	1,5	1,5	1,5	1,5	1,5	1,5	10	10
10	10	10	1,5	1,5	1,5	10	1,5	10	2,5
10	1,9	1,9	1,5	1,6	1,8	1,9	1,9	1,8	1,8
1,9	10	10	1,5	1,5	1,5	1,5	1,5	10	1,5
10	10	10	1,9	1,5	1,5	1,8	1,8	1,8	1,8
10	10	10	1,9	1,5	1,5	10	10	10	1,9
10	10	10	1,9	1,8	1,8	10	10	10	1,9
10	10	10	1,9	1,9	1,9	10	10	10	1,8
10	10	10	1,9	1,9	1,8	10	10	10	1,9
10	10	10	1,9	10	10	10	10	10	1,9
10	10	10	2,6	2,6	2,6	10	10	10	10
10	10	10	1,9	1,9	10	10	1,9	10	10
10	10	10	1,9	1,9	1,9	1,9	1,9	1,9	1,9
10	1,8	10	1,8	1,8	1,5	1,9	1,9	1,8	1,8
10	1,8	1,8	1,6	10	1,8	1,7	1,6	10	10
1,9	10	10	1,5	1,5	1,5	10	10	10	10
10	10	10	1,5	10	10	10	10	10	10
2,6	10	10	2,6	2,6	1,5	1,5	1,6	1,5	1,6
2,5	10	10	2,6	2,6	2,5	2,5	2,6	2,5	2,5
2,5	10	10	2,6	2,6	2,9	2,5	2,6	2,5	2,6
1,9	10	10	1,5	1,5	1,5	1,5	1,5	10	1,5
1,9	10	10	1,5	1,5	1,5	1,5	1,5	10	1,5
10	10	10	1,5	1,5	1,5	10	10	10	10
1,5	10	10	10	10	10	10	10	10	10
10	10	10	1,9	1,9	1,9	1,9	1,9	10	1,9
2,5	10	10	2,6	10	2,6	1,5	10	10	2,8
10	10	10	10	10	10	10	10	10	10
10	1,9	10	1,9	1,9	10	1,9	10	10	10
10	1,9	10	1,9	1,9	10	1,9	10	10	10
1,5	10	10	1,5	10	1,5	1,5	1,5	10	1,5
1,6	1,5	1,5	1,5	10	1,5	10	10	10	10
1,5	10	10	1,5	10	1,5	10	10	10	1,5
2,6	2,6	10	2,6	2,6	2,5	10	2,5	2,5	2,5
10	10	10	2,6	2,6	10	2,5	2,5	2,5	2,5
10	10	10	1,9	1,9	1,9	1,9	1,9	10	1,9
10	10	10	2,5	2,5	1,5	1,8	1,8	1,8	1,8
2,6	10	10	2,6	10	2,6	10	10	10	10
1,5	10	10	1,9	1,9	1,5	10	10	10	1,5
2,9	10	10	1,5	1,5	1,5	10	10	10	1,5
1,9	10	10	1,9	1,9	1,5	10	10	10	1,9
1,9	10	10	1,9	1,9	1,5	10	10	10	1,9
1,9	10	10	1,9	1,9	1,5	10	10	10	1,9
1,9	10	10	1,9	1,9	1,5	10	10	10	1,9
2,5	10	10	2,5	2,5	2,5	10	10	10	10

10	10	10	1,9	1,9	1,9	10	10	10	10
10	10	10	1,5	10	10	1,9	1,9	10	10
10	10	10	1,7	1,7	1,7	10	10	10	10
10	10	10	1,8	1,8	1,8	10	10	10	10
1,9	1,9	1,9	10	1,9	1,9	10	10	10	10
1,5	2,5	1,5	2,6	2,6	1,5	2,5	2,5	10	2,5
2,6	2,5	2,5	2,5	2,5	2,5	2,5	2,6	2,5	2,5
1,5	1,5	10	1,6	1,5	1,5	10	10	10	10
1,5	10	10	1,5	1,5	1,5	10	10	10	10
10	10	10	10	2,9	10	10	2,9	10	10
10	1,9	10	1,9	10	10	10	10	10	10
10	10	1,9	1,9	1,9	1,9	10	10	10	1,9
10	10	10	1,5	1,5	1,5	10	10	10	10
10	10	10	2,5	2,5	2,5	2,5	2,5	10	2,5
10	1,9	10	1,9	10	10	10	10	10	1,8
2,5	2,5	2,5	2,6	2,6	2,6	2,5	2,5	2,6	2,6
1,8	1,8	1,8	2,8	2,8	1,8	10	10	10	10
1,5	10	10	1,5	1,5	1,5	10	10	1,5	1,5
10	10	10	10	10	1,5	10	1,6	1,6	10
10	10	10	1,9	1,9	10	1,9	10	10	1,9
2,5	2,5	2,6	2,5	2,6	2,5	2,5	2,5	2,6	2,6
10	10	10	1,5	1,5	1,5	10	10	10	1,5
10	10	10	1,5	1,5	1,5	1,5	1,5	10	1,5
10	10	10	1,5	1,5	1,5	1,5	1,5	10	1,5
10	10	10	1,5	1,5	1,5	1,5	1,5	10	10
10	10	10	10	2,9	10	10	10	10	10
1,8	2,8	2,8	10	10	10	10	10	10	1,8
10	10	10	1,5	1,5	1,5	10	10	10	10
10	10	10	1,9	1,9	1,9	10	10	10	1,9
2,5	2,6	2,5	2,5	2,6	2,5	2,5	3,5	2,5	3,5
10	10	10	1,8	1,8	10	1,5	1,5	1,5	1,5
10	10	10	1,5	10	1,5	1,5	1,5	10	1,5
2,6	10	10	2,5	2,6	2,6	2,5	2,6	2,5	2,6
2,9	1,5	1,5	1,9	1,9	1,9	10	10	10	10
1,6	10	10	10	1,5	1,5	10	10	10	10
10	1,8	1,8	1,8	1,8	10	10	10	10	10
10	10	10	10	10	10	10	10	10	10
10	10	10	10	10	10	10	10	10	10
1,8	1,5	1,5	1,5	10	1,5	10	10	10	10
1,8	10	10	1,5	1,5	1,5	10	10	10	10
10	10	10	1,5	1,5	1,5	10	10	10	10
10	1,5	10	1,5	1,5	10	1,5	1,5	1,5	1,5
10	10	10	10	10	10	10	10	10	10
10	10	10	10	10	10	10	10	10	10
2,6	10	10	2,5	2,6	2,6	10	10	2,5	2,6
10	10	10	10	10	10	10	10	10	10
10	10	10	10	10	2,6	10	10	10	10

10	10	10	1,9	1,9	1,9	1,9	1,9	10	10
10	10	10	10	1,9	10	10	10	10	10
10	10	10	2,5	2,5	2,5	10	10	10	10
10	10	10	2,5	2,5	2,5	10	10	10	10
10	2,5	2,5	2,5	10	10	10	10	10	1,5
10	10	10	1,5	1,5	1,5	1,5	1,5	1,5	1,5
10	10	10	2,6	3,6	10	10	10	2,6	2,6
1,5	1,5	1,5	1,5	1,5	1,5	10	10	10	10
10	10	10	10	1,5	10	10	1,9	10	10
10	1,5	10	1,5	1,5	10	10	10	10	10
10	10	10	2,9	2,9	2,9	10	10	10	10
10	10	10	2,9	2,9	2,9	10	10	10	10
10	10	10	1,9	1,9	1,9	1,9	1,9	10	10
1,6	1,5	1,5	1,6	1,6	1,5	10	10	10	10
10	1,5	1,5	1,5	1,5	1,5	10	10	10	10
10	10	10	1,5	1,5	1,5	10	10	10	10
10	10	10	1,5	1,5	1,5	10	10	10	10
10	10	10	10	10	1,5	10	10	10	10
10	10	10	2,5	2,5	2,5	10	2,5	2,5	2,5
10	1,5	1,5	1,4	1,4	1,5	10	10	10	10
10	10	10	1,5	10	10	10	1,9	10	10
1,9	10	10	1,5	1,5	1,5	10	10	10	10
1,9	10	10	1,5	1,5	1,5	10	10	10	10
10	10	10	1,5	1,5	1,5	10	10	10	1,5
10	10	10	1,5	1,5	1,5	10	10	10	1,5
1,5	10	10	1,5	1,5	1,5	1,5	1,5	10	10
1,5	1,5	1,5	1,5	1,5	1,5	10	10	10	1,5
10	10	10	1,8	1,8	1,8	10	10	10	1,5
10	1,9	1,9	10	10	1,9	1,9	10	10	10
2,6	2,5	2,5	2,6	2,5	2,5	2,5	2,5	2,5	2,5
10	10	10	10	1,9	10	1,9	10	10	10
10	10	10	1,9	1,9	10	10	10	1,9	1,9
1,6	10	10	1,5	1,5	1,6	1,8	10	10	1,6
2,5	10	10	10	2,6	2,6	2,5	2,5	2,6	2,5
1,5	10	10	1,5	1,5	1,5	1,5	1,5	10	10
10	1,9	10	1,9	1,9	1,9	10	10	1,9	1,9
10	1,9	10	10	10	1,9	10	10	1,9	10
10	10	10	1,9	1,9	1,9	10	10	10	10
1,5	10	10	1,5	10	1,5	1,5	1,5	10	1,5
1,5	10	10	1,5	10	1,5	1,5	1,5	10	1,5
10	10	10	1,9	1,9	10	10	10	1,9	1,9
10	10	10	1,5	1,5	1,5	10	10	10	1,5
10	10	10	1,5	1,8	1,8	10	10	10	1,5
1,5	10	10	1,5	1,5	1,5	1,5	1,5	1,5	1,5
1,5	1,5	1,5	1,5	1,5	1,5	1,5	1,5	1,5	1,5
10	10	10	10	10	10	10	10	10	10
10	10	10	1,5	1,5	1,5	10	10	10	10

1,6	1,9	1,9	1,5	1,5	1,6	10	10	10	10
10	1,9	10	1,9	1,9	1,9	1,9	1,9	10	1,9
10	1,9	10	1,9	1,9	1,9	1,9	1,9	10	10
2,6	2,6	2,6	2,6	2,5	2,5	2,5	2,5	2,6	2,5
2,6	2,6	2,6	2,6	2,5	2,5	2,5	2,5	2,6	2,5
10	1,9	1,9	10	10	10	1,9	1,9	10	10
10	10	10	1,9	10	10	10	10	1,9	10
10	10	10	1,9	1,9	10	10	3,9	10	10
1,7	10	10	10	1,6	1,5	1,5	1,5	1,5	1,5
10	10	10	10	10	10	10	10	10	10
1,5	10	10	1,5	1,5	1,5	10	10	10	10
1,9	1,9	1,5	1,5	1,5	1,5	10	10	10	1,9
10	10	10	1,9	1,9	1,9	1,9	1,9	10	10
10	10	1,9	1,9	10	1,9	10	10	1,9	10
1,9	1,9	10	1,5	1,5	1,5	10	10	10	1,5
10	10	10	1,9	1,9	1,9	10	10	1,9	10
1,9	1,9	1,9	1,5	1,5	1,5	10	10	10	1,9
10	10	10	1,9	1,9	1,9	10	1,9	10	10
10	1,9	10	1,9	10	1,9	1,9	10	10	10
10	10	10	1,9	1,9	1,5	10	10	10	1,9
10	10	10	1,5	1,5	10	10	10	1,5	1,5
10	10	10	1,5	1,5	1,5	1,5	1,5	10	1,5
10	10	10	1,5	1,5	1,5	1,5	1,5	10	1,5
10	10	10	2,9	10	10	10	10	10	10
10	10	10	1,9	1,9	1,5	10	10	10	10
1,5	1,5	1,5	1,5	10	1,5	10	10	10	10
2,8	10	10	1,5	1,5	2,5	2,8	2,8	2,8	2,8
10	10	10	1,9	1,9	1,9	1,9	1,9	10	10
1,9	10	10	10	2,5	1,9	10	1,9	10	10
10	10	10	1,9	1,5	1,5	10	10	10	1,5
1,9	1,9	10	1,9	1,9	1,5	10	10	10	1,5
10	10	10	10	10	10	10	1,5	1,5	1,5
1,5	10	10	1,5	1,5	1,5	1,5	1,5	1,5	1,5
1,9	10	10	1,5	1,5	1,5	10	10	10	2,5
10	1,9	10	1,9	10	1,9	10	10	1,9	10
10	10	10	1,9	1,9	10	10	10	10	1,9
1,5	10	10	1,5	1,5	1,5	1,5	1,5	10	10
10	10	10	1,9	1,8	1,8	10	10	10	1,9
10	10	10	1,9	10	1,9	1,9	10	10	10
10	10	10	1,5	1,5	1,5	1,5	1,5	10	10
10	1,9	1,5	1,5	1,5	1,5	10	10	10	1,9
10	1,5	1,5	1,9	1,9	1,9	1,6	1,6	1,5	1,5
10	10	10	1,9	1,9	10	1,9	1,9	1,9	1,9
10	10	10	2,9	10	10	10	1,9	1,9	10
10	10	10	1,9	1,9	10	1,8	1,8	1,8	1,8
10	10	10	2,9	2,9	2,9	1,5	1,6	1,6	1,6
10	10	10	2,6	2,6	2,5	10	10	2,5	2,6

10	10	10	2,6	2,6	2,5	10	10	2,5	2,6
10	10	10	1,9	1,9	10	10	10	1,9	1,9
1,9	10	10	1,8	1,8	1,5	10	10	10	1,9
10	1,9	10	1,9	1,9	10	10	10	1,9	10
10	10	1,9	1,9	10	10	10	10	1,9	1,9
10	10	10	1,9	1,9	10	10	1,9	1,9	10
10	10	10	1,9	1,9	1,9	10	10	1,9	1,9
10	10	10	1,9	1,9	1,9	10	10	1,9	10
10	10	10	1,9	1,9	10	10	10	1,9	1,9
10	10	10	1,9	1,9	1,9	10	10	1,9	10
1,5	10	10	1,5	1,5	1,5	1,5	1,5	10	10
1,9	1,9	1,9	1,5	1,5	1,5	10	10	10	1,9
10	10	10	10	1,9	10	10	10	10	10
10	10	10	10	1,9	10	10	10	10	10
10	10	10	1,9	1,9	1,9	10	1,9	1,9	10
1,5	1,6	1,6	1,8	1,8	1,5	10	10	10	10
2,5	2,5	10	2,5	2,5	2,5	2,5	2,5	2,5	2,5
2,5	2,5	10	2,5	2,5	2,5	1,5	2,5	2,5	2,5
10	10	10	1,9	1,9	1,9	10	10	10	10
10	10	10	1,9	1,9	10	10	10	10	1,9
10	10	10	10	2,9	10	10	1,9	10	10
10	10	10	1,9	1,9	10	10	10	10	10
10	10	10	1,9	1,9	10	10	10	10	1,9
10	10	10	10	10	2,9	1,9	10	1,9	10
10	10	10	10	1,9	2,9	1,9	10	1,9	10
1,5	10	10	1,5	1,5	1,5	1,5	1,5	1,5	10
10	10	10	1,9	1,9	1,9	10	1,9	1,9	10
1,9	10	10	1,9	1,9	1,5	10	10	10	1,9
10	1,9	10	10	1,9	1,9	10	1,9	10	10
10	10	10	1,9	1,9	1,9	1,9	1,9	10	10
10	10	10	1,9	1,9	1,9	10	10	10	10
1,9	1,9	10	10	10	10	10	1,9	10	10
10	1,9	10	1,9	10	1,9	10	10	10	10
10	10	10	1,5	1,5	1,5	10	10	10	10
2,5	2,5	2,5	2,5	2,5	2,5	10	10	2,5	2,5
10	10	10	1,5	1,5	1,5	10	10	10	10
10	1,9	10	1,9	1,9	1,9	10	1,9	10	10
10	10	10	1,9	1,9	1,9	10	10	1,9	1,9
1,5	10	10	1,6	10	10	10	10	10	10
1,9	1,9	10	1,9	1,9	1,9	10	10	10	1,8
10	10	10	2,9	2,9	1,9	10	1,9	1,9	10
1,9	10	10	1,9	1,9	10	1,9	10	10	10
10	10	10	1,9	1,9	10	10	10	10	1,5
10	10	10	1,9	1,9	10	10	10	10	1,5
10	10	10	10	10	10	10	10	10	10
10	10	10	1,9	1,9	1,9	1,5	1,5	10	1,5
10	10	10	1,5	1,5	1,6	10	1,5	10	1,5

1,9	10	10	1,9	1,9	1,9	1,9	1,9	10	1,9
10	10	10	1,6	1,5	1,5	10	10	10	1,5
1,5	1,5	1,5	1,5	1,5	1,5	10	10	10	1,5
1,9	1,9	10	1,9	1,9	10	10	10	1,5	10
10	10	10	1,5	1,5	1,5	1,5	1,5	1,5	1,5
10	10	10	10	10	10	10	10	10	10
1,5	10	10	10	10	10	10	10	10	10
10	10	10	10	10	10	10	10	10	1,6
10	10	10	1,6	1,6	1,6	10	1,6	10	1,5
10	10	10	1,6	1,6	1,6	10	1,6	10	1,5
10	10	10	10	10	10	10	1,5	10	10
10	10	10	1,5	1,5	1,5	1,5	1,5	1,5	1,5
10	10	10	10	10	10	10	10	10	10
10	10	10	1,6	1,6	1,5	10	10	10	1,5
1,9	1,9	1,5	10	10	10	10	10	10	10
1,9	10	10	1,9	1,9	1,9	1,9	1,9	10	1,9
10	10	10	1,9	1,9	10	10	10	10	1,5
10	10	10	1,6	10	10	10	1,5	10	10
10	10	10	1,6	1,6	1,6	10	10	10	1,6
10	10	10	1,5	1,5	1,5	1,5	1,5	1,5	1,5
10	1,9	1,9	1,9	1,9	10	10	10	1,9	10
10	10	10	1,9	10	10	10	10	10	1,9
1,9	10	10	1,9	1,9	10	10	10	10	1,9
10	10	10	1,9	1,9	1,8	10	10	10	1,9
10	10	10	1,9	1,9	1,9	10	10	10	10
1,5	1,5	1,5	1,9	1,9	1,9	10	10	10	1,9
10	10	10	1,9	1,8	1,8	10	10	10	1,8
1,5	1,5	10	1,5	1,5	1,5	10	10	10	1,5
10	1,9	10	1,9	1,8	1,8	10	10	10	1,9
1,5	10	10	1,5	1,5	1,5	10	10	10	1,5
10	10	10	10	10	10	10	1,9	10	10
10	10	10	10	10	10	1,9	1,9	10	10
10	10	10	10	10	10	10	1,9	10	10
1,5	10	10	1,5	1,5	1,5	10	10	10	1,5
10	10	10	1,9	1,9	1,8	10	10	10	1,9
10	10	10	10	10	10	10	10	10	10
10	10	10	1,9	1,9	1,9	10	1,9	10	1,9
10	10	10	1,9	1,8	10	10	10	10	1,9
10	10	10	1,9	1,8	1,8	10	10	10	1,9
10	10	1,9	1,9	1,9	1,8	10	10	10	1,9
10	1,9	10	10	10	1,9	1,9	1,9	1,9	1,9
10	10	10	1,9	10	10	10	1,9	10	10
1,5	10	10	1,9	1,9	1,9	1,5	1,5	1,5	1,5
10	1,9	10	10	10	10	10	1,5	10	10
1,5	1,5	1,5	1,5	1,5	1,5	1,5	1,5	1,5	1,5
1,9	10	10	1,9	1,9	1,9	10	10	10	10
10	10	10	1,9	1,8	1,8	10	10	10	1,8

1,9	10	10	1,9	1,9	10	10	10	10	1,8
1,5	10	10	1,5	1,5	1,5	10	10	10	1,5
10	10	10	10	10	10	10	10	10	10
1,6	10	10	1,6	10	1,6	1,6	10	10	1,5
1,9	10	10	1,9	1,9	1,9	1,5	1,5	10	1,5
10	1,9	1,9	1,9	1,9	1,9	10	10	1,5	10
10	10	10	1,9	1,8	1,8	10	10	10	1,8
10	10	10	1,9	1,8	1,8	10	10	10	1,5
1,9	1,9	1,9	10	10	10	10	10	10	10
10	10	10	1,6	10	1,5	1,5	1,5	10	2,5
10	10	10	1,9	1,8	1,8	10	10	10	10
10	10	1,9	10	10	10	1,9	10	10	10
10	10	10	1,9	1,9	1,5	10	10	10	1,5
1,9	10	10	1,9	1,8	1,8	10	10	10	1,8
10	10	10	10	1,9	1,9	10	10	10	1,5
10	10	10	1,9	1,8	1,8	10	10	10	1,5
10	10	10	1,9	1,8	1,8	10	10	10	1,5
10	10	10	1,9	1,8	1,8	10	10	10	1,8
1,9	10	10	1,9	1,9	1,9	1,5	1,5	10	1,5
10	10	10	1,9	1,9	1,9	10	10	10	1,8
10	10	10	1,6	1,6	1,5	10	10	10	10
10	1,9	10	1,9	1,8	1,8	10	10	10	10
10	10	10	1,9	1,8	1,8	10	10	10	1,8
1,5	1,5	1,5	1,5	1,5	1,5	1,5	1,5	1,5	1,5
10	10	10	1,9	1,9	10	10	10	10	1,8
10	10	10	1,9	1,9	10	10	10	10	10
1,5	1,9	10	1,9	1,5	1,5	10	10	10	1,5
10	1,9	10	1,9	1,8	1,8	10	10	10	1,8
10	10	10	10	1,8	1,8	10	10	10	1,8
10	10	10	1,9	1,9	1,8	10	10	10	1,5
10	10	10	10	10	10	10	1,8	10	10
1,9	10	10	1,9	1,9	1,9	10	1,5	10	1,5
10	10	10	10	10	10	10	10	10	10
10	10	10	1,8	1,8	1,8	10	10	10	10
10	10	10	2,8	2,8	10	10	10	10	10
1,5	1,5	1,5	1,5	1,5	1,5	10	10	10	10
2,9	10	10	2,9	2,9	2,9	4,9	4,9	10	4,9
1,9	10	10	1,9	1,9	1,9	10	10	10	10
1,9	1,9	1,9	1,9	1,9	1,9	10	10	10	10
1,9	1,9	1,9	1,9	1,9	1,9	10	10	10	10
10	10	10	2,6	1,6	1,6	10	10	10	10
10	10	10	1,9	1,5	1,5	10	10	10	1,5
10	10	10	2,6	2,7	1,5	2,6	1,5	10	10
10	10	1,9	10	10	10	10	10	10	1,9
10	10	10	1,6	1,5	1,5	1,6	1,5	10	2,7
10	10	10	1,9	10	1,9	10	1,5	10	10
10	10	10	10	10	10	10	1,9	10	10

10	10	10	1,9	1,9	1,8	1,9	1,9	1,9	1,9
1,6	10	10	1,6	10	10	10	1,6	10	10
10	10	10	10	10	10	10	1,9	10	10
10	10	1,9	1,9	1,9	1,9	10	10	10	1,9
10	10	1,9	1,9	1,9	1,9	10	10	10	1,9
10	10	1,9	1,9	1,9	1,9	10	10	10	1,9
1,9	1,9	1,5	1,9	1,5	1,5	10	10	10	1,5
1,9	1,9	1,5	1,9	1,5	1,5	10	10	10	1,5
1,5	10	10	1,5	1,5	1,5	10	10	10	1,5
10	10	10	1,9	1,9	1,9	10	10	10	10
10	10	10	1,9	1,9	1,9	10	1,9	10	1,9
10	10	10	1,9	10	10	1,9	10	10	10
10	10	10	1,9	1,9	10	10	10	10	1,5
10	10	10	10	10	1,5	10	1,5	10	10
10	10	10	1,9	1,9	10	10	10	10	1,5
10	1,9	10	1,9	10	1,9	1,9	10	10	10
10	10	10	1,9	1,9	1,9	10	10	10	10
10	10	10	10	10	10	1,9	1,9	10	10
1,5	1,5	1,5	1,5	1,5	1,5	1,5	1,5	1,5	1,5
10	10	10	1,9	1,9	1,9	10	10	10	10
10	10	1,9	1,9	10	10	10	10	10	1,9
10	10	10	1,9	1,9	1,9	10	10	10	10
1,9	10	10	1,9	1,9	1,9	10	10	10	10
10	10	10	1,9	1,8	1,8	10	10	10	1,5
10	10	10	10	10	10	10	1,9	10	10
1,9	10	10	1,9	1,9	1,9	10	10	10	10
1,6	1,6	1,6	1,5	1,5	1,6	1,6	1,6	10	1,6
1,9	10	10	1,9	1,9	1,9	1,9	1,9	10	1,9
1,9	10	10	1,9	1,9	1,9	10	10	10	10
1,5	10	10	1,9	1,9	1,9	10	10	10	10
1,6	1,6	1,6	1,6	1,6	1,6	1,6	1,6	1,6	1,6
10	10	10	1,9	1,8	1,8	10	10	10	1,5
10	10	10	1,9	10	10	1,5	1,5	1,5	1,5
10	10	10	1,9	1,9	1,8	10	10	10	1,9
10	10	10	1,9	10	10	10	1,9	10	10
1,9	10	10	1,9	10	10	10	10	10	1,9
1,9	10	10	1,9	1,9	1,9	1,9	1,9	10	1,9
1,9	10	10	1,9	1,9	1,9	1,9	1,9	10	1,9
10	10	10	1,5	1,5	1,5	1,5	1,5	1,5	1,5
1,5	1,5	1,5	10	10	1,5	10	1,5	1,5	1,5
1,5	1,5	1,5	1,5	1,5	1,5	10	1,5	1,5	1,5
1,6	10	10	1,5	1,6	1,6	10	1,6	10	1,6
10	10	10	1,5	1,5	1,5	1,5	1,5	1,5	1,5
10	10	10	1,9	1,9	1,9	10	10	10	10
10	10	10	10	10	10	10	10	10	10
1,9	1,9	1,5	1,9	1,9	1,5	10	1,9	1,9	1,9
10	10	10	1,9	10	1,9	10	10	10	1,5

10	10	10	10	10	1,9	1,9	1,9	10	1,9
10	10	10	10	10	1,9	1,9	1,9	10	1,9
10	10	10	10	10	10	10	10	10	10
10	10	10	10	10	10	10	10	10	10
10	10	10	10	10	10	10	1,5	10	1,5
10	1,9	10	1,9	1,9	1,8	10	10	10	1,9
10	10	10	10	10	10	10	10	10	10
1,5	1,5	1,5	1,5	1,5	1,5	1,5	1,5	1,5	1,5
1,5	1,5	1,5	1,5	1,5	1,5	1,5	1,5	1,5	1,5
10	10	10	10	1,9	1,9	10	10	10	10
1,5	1,5	1,5	1,9	1,9	1,9	1,5	1,5	1,5	1,5
10	10	10	10	10	10	1,9	10	10	10
1,9	1,9	1,5	1,9	1,9	1,5	10	10	10	1,5
10	10	10	10	10	10	10	10	10	10
10	10	10	1,9	1,9	1,8	10	10	10	1,9
10	10	10	10	10	10	10	1,9	10	10
10	10	10	1,9	10	1,9	10	10	10	10
10	10	1,9	1,9	1,9	1,5	10	10	10	10
10	10	10	1,9	10	1,8	1,9	10	10	1,9
10	10	10	10	10	10	10	10	10	10
10	10	10	10	10	10	10	10	10	10
10	10	1,5	10	10	10	10	10	10	10
10	10	10	1,5	10	1,9	10	10	10	10
10	10	10	1,9	10	1,9	10	10	10	10
1,9	10	10	10	10	10	10	10	1,5	1,5
1,9	1,9	1,9	1,9	1,9	1,9	1,9	1,9	1,9	1,9
1,9	10	10	10	1,9	1,5	10	10	1,5	1,5
10	10	10	1,9	10	1,9	1,5	1,5	10	1,5
1,5	1,5	1,5	1,5	1,6	1,6	10	10	10	1,5
1,5	10	10	1,5	1,5	1,5	1,5	1,5	10	10
10	10	10	10	10	10	10	10	10	10
1,9	1,9	1,5	1,9	1,9	1,9	1,5	1,5	1,5	1,5
10	10	10	1,9	10	1,9	1,5	1,5	10	10
10	10	10	10	10	10	10	10	10	10
10	10	10	1,9	10	1,9	1,5	1,5	10	10
1,5	10	10	1,9	1,9	1,9	10	10	10	1,5
10	10	10	10	10	10	1,9	10	10	10
1,9	10	10	1,9	1,9	1,5	1,5	1,5	1,5	1,5
10	10	10	3,5	10	10	10	2,6	10	10
10	10	10	1,5	10	1,5	10	10	10	10
10	10	10	10	10	10	1,5	1,5	1,5	1,5
10	10	10	10	10	10	1,5	1,5	1,5	1,5
10	10	10	10	10	10	10	10	10	10
10	10	10	10	10	10	10	10	10	10
10	10	10	10	10	1,9	10	10	10	1,9
1,9	10	10	1,9	1,9	1,9	10	10	1,9	1,9
10	10	10	10	10	10	1,5	1,5	1,5	1,5

10	10	10	10	10	10	10	10	10	10
1,9	10	10	1,9	10	10	1,5	1,5	10	10
10	1,9	10	1,9	1,9	1,9	1,9	1,9	10	1,9
1,9	10	10	1,9	10	10	1,5	1,5	10	10
10	1,9	10	10	10	1,9	1,9	10	10	10
10	10	10	10	10	1,9	10	10	10	1,9
10	10	10	1,9	1,9	1,9	1,5	1,5	10	10
10	10	1,9	1,9	10	1,9	1,9	1,9	1,9	1,9
10	10	10	1,9	10	10	10	10	10	1,9
1,5	1,5	1,5	1,5	1,5	1,5	1,5	1,5	1,5	1,5
10	10	10	1,9	1,9	1,9	1,5	1,5	10	1,5
1,5	1,5	1,5	1,5	1,5	1,5	1,5	1,5	1,5	1,5
10	10	10	1,9	1,9	1,8	10	10	10	1,9
10	10	10	1,9	1,9	1,8	10	10	10	1,9
1,9	10	10	1,9	1,9	10	10	10	1,5	1,5
10	10	10	10	10	10	10	10	10	10
10	10	10	1,9	1,9	10	10	10	1,5	1,5
10	10	10	1,9	1,9	10	10	10	1,5	1,5
10	1,9	10	1,9	1,9	1,9	10	10	10	1,5
1,9	1,9	1,5	1,9	1,9	1,9	10	10	10	1,5
10	10	10	1,9	1,9	1,9	1,5	1,5	10	10
10	10	10	10	10	10	10	10	10	10
10	10	10	10	10	10	1,5	1,5	10	10
10	10	10	1,9	1,9	1,9	10	1,9	10	10
10	10	10	10	1,9	1,9	10	10	1,8	1,8
10	10	10	10	1,8	10	10	10	10	10
10	10	10	10	1,9	1,9	10	10	1,8	1,8
1,5	10	10	10	1,5	1,5	10	10	10	1,5
1,9	1,9	1,9	1,9	1,9	1,9	1,9	1,9	10	1,9
1,9	1,9	1,9	1,9	1,9	1,9	10	10	10	1,9
10	10	10	1,9	10	10	10	10	10	1,8
10	10	10	10	10	1,5	10	1,9	10	10
10	10	10	10	1,9	10	10	10	10	10
10	10	10	10	10	10	10	10	10	10
10	10	10	1,6	1,6	1,6	10	10	10	10
10	10	10	1,9	1,9	1,8	10	10	10	1,9
1,9	10	10	10	10	10	10	1,5	10	1,5
1,6	1,6	10	1,6	1,6	1,6	1,6	1,6	1,6	1,7
1,9	10	10	1,9	1,9	1,9	10	1,9	10	1,9
10	1,8	10	1,9	1,8	1,8	10	10	10	1,5
10	10	10	1,9	10	10	1,9	10	10	10
1,9	10	10	1,9	1,9	1,9	10	10	10	10
1,9	10	10	1,9	1,9	1,9	10	10	10	10
1,9	1,9	1,9	1,9	1,9	1,9	10	10	10	1,9
10	10	10	1,9	1,9	1,9	10	10	10	10
10	10	10	1,9	1,8	1,8	10	10	10	1,9

stewed mc	stewed mc	boiled chir	stewed chi	stewed chi	green pepp	yellow pep	onion	kale	fried onior
10	10	10	1,5	10	1,6	1,6	1,7	10	10
10	10	10	1,5	10	1,6	1,6	1,7	10	10
10	10	1,5	1,5	1,5	1,5	1,5	1,7	1,6	1,5
10	1,9	10	10	1,9	1,5	1,5	10	10	10
10	10	1,5	1,5	1,5	1,5	1,5	1,5	10	1,5
10	1,9	10	10	10	1,7	10	1,7	10	10
10	10	10	1,5	10	1,8	10	1,5	10	1,5
10	10	10	10	1,5	1,5	10	1,5	1,9	10
10	10	2,9	2,9	10	1,5	1,5	1,5	2,9	10
1,9	10	10	10	10	1,5	1,5	1,5	10	10
1,5	10	1,9	10	10	1,6	1,6	1,6	10	1,6
10	10	2,9	2,9	10	1,6	1,6	1,6	2,6	10
2,5	10	2,5	2,5	10	1,5	10	1,5	2,5	1,5
1,9	1,9	10	10	10	1,5	10	1,7	10	10
1,9	1,9	10	10	10	1,6	10	1,6	10	1,7
10	10	10	10	1,8	10	10	1,8	10	10
10	10	10	10	1,8	10	10	1,9	10	10
2,5	10	2,5	2,5	10	1,5	1,5	1,5	2,5	1,6
1,5	10	2,5	2,5	10	1,5	10	10	2,5	1,5
2,5	10	2,5	2,5	10	10	10	10	10	1,6
2,5	10	2,5	2,5	10	1,5	10	1,6	2,5	1,5
10	10	1,5	2,5	10	1,5	10	1,5	2,5	1,5
10	10	2,5	2,5	10	1,5	10	1,6	2,5	10
10	10	2,5	2,5	10	1,5	10	1,5	2,5	1,5
1,5	10	2,5	2,5	10	1,5	1,5	1,5	2,6	1,5
2,5	10	2,5	2,5	10	1,5	10	1,5	2,5	1,5
10	10	10	10	1,5	2,6	2,6	2,6	10	10
10	10	1,9	10	10	1,5	10	1,5	10	10
1,5	1,5	1,5	1,5	1,5	1,5	1,5	1,6	1,5	1,5
10	10	2,7	10	1,7	10	10	2,7	10	10
1,5	1,5	1,5	1,5	1,5	1,5	1,5	10	1,8	10
2,9	2,9	10	2,9	10	1,5	10	10	2,9	1,5
2,6	2,6	2,6	2,6	10	1,5	1,5	1,5	2,6	1,5
10	1,9	10	10	1,9	10	10	1,7	10	10
1,5	10	1,9	10	10	1,5	10	1,5	10	1,5
10	10	10	10	1,5	10	10	1,5	10	10
1,5	10	10	2,7	10	1,5	10	2,8	10	1,5
10	10	1,9	10	10	10	10	10	10	10
10	10	10	2,6	10	10	10	10	10	10
10	10	1,5	1,5	10	10	10	1,6	10	1,5
1,5	10	10	1,6	10	1,6	1,6	10	10	10
10	10	1,5	1,5	1,5	1,5	1,5	3,7	10	10
10	1,5	1,6	10	10	1,6	10	1,6	10	10
1,5	10	1,5	10	10	1,6	1,6	1,6	10	10
1,5	1,5	1,5	1,5	10	1,5	1,5	1,5	1,9	1,5
10	10	1,9	10	10	10	10	1,5	10	10

10	10	1,9	10	1,9	10	10	1,6	10	10
10	10	10	10	10	2,6	10	2,6	10	10
1,5	1,5	2,5	2,5	2,6	10	10	10	2,6	10
1,5	1,5	1,5	1,5	10	1,5	1,5	3,7	10	1,5
1,6	1,5	10	10	10	1,6	1,6	1,7	10	10
10	1,9	1,6	10	10	10	10	1,6	1,5	10
1,5	10	1,9	1,9	10	1,5	10	1,5	1,9	1,5
10	1,9	1,5	10	1,8	1,5	10	10	10	10
10	10	1,5	10	10	1,6	1,6	10	10	10
10	10	1,5	1,5	10	10	10	10	10	10
10	10	1,5	10	10	1,6	10	10	10	10
10	10	1,5	10	10	1,6	10	10	10	10
1,9	1,9	1,5	10	1,5	10	10	10	10	10
10	10	2,6	2,6	10	1,6	1,6	2,6	10	10
10	10	10	10	10	1,6	1,6	1,6	10	10
1,9	10	1,9	10	10	1,5	10	10	1,9	10
10	10	10	10	1,5	1,8	1,8	1,8	10	10
10	10	1,6	1,6	1,6	10	10	1,7	10	1,7
1,5	10	1,9	1,9	10	1,5	10	1,5	10	1,5
10	10	1,5	1,5	10	1,5	10	1,5	10	1,5
10	10	1,9	1,9	10	10	10	10	10	10
10	10	2,5	2,6	2,6	2,5	2,5	10	2,9	10
10	10	2,5	2,6	2,6	1,5	1,5	10	2,5	10
1,5	10	1,9	1,9	1,8	1,5	10	1,5	10	1,5
1,5	10	1,9	1,9	1,8	1,5	10	1,5	10	1,5
1,9	10	1,8	10	10	1,8	10	1,6	10	10
10	10	1,5	1,5	10	10	10	1,5	10	1,5
1,9	1,9	1,9	10	1,9	1,5	10	10	1,9	10
10	10	2,6	2,6	10	2,5	10	2,5	10	2,5
10	10	1,6	1,6	2,5	10	10	2,7	10	2,7
1,9	10	10	1,9	10	10	10	1,5	10	10
1,9	10	10	1,9	10	10	10	1,5	10	10
10	10	1,9	1,5	10	1,5	1,5	4,7	10	10
10	10	1,5	1,5	1,5	1,5	1,5	10	10	10
10	10	1,5	10	10	10	10	1,5	10	1,5
10	10	2,5	2,6	10	10	10	10	1,5	1,5
10	10	2,5	2,5	1,5	1,5	1,5	10	2,5	1,5
1,9	10	1,9	10	1,9	1,5	10	10	1,9	10
10	10	10	10	10	10	10	10	10	10
10	10	1,5	1,6	10	1,5	1,5	3,7	10	10
1,9	10	1,9	1,9	10	1,5	10	1,5	10	1,5
2,9	1,9	1,9	1,9	1,9	1,5	1,5	1,6	2,9	1,6
10	10	1,9	1,9	10	1,5	10	1,6	10	1,6
10	10	1,9	1,9	10	1,5	1,5	1,6	10	1,6
10	10	2,9	2,9	2,9	1,5	10	1,6	10	1,6
10	10	1,9	1,9	10	1,5	10	1,5	10	1,5
2,5	2,6	2,5	2,6	2,5	1,5	1,5	1,5	10	1,5

1,9	1,9	1,9	10	1,9	1,5	10	1,5	10	10
10	10	1,9	10	10	1,5	1,5	1,5	10	10
1,6	1,6	1,6	1,6	10	1,8	1,8	10	10	10
1,9	1,9	10	1,5	1,5	1,8	1,8	10	10	10
1,9	1,9	1,9	1,9	10	1,5	10	1,5	1,9	1,5
2,5	10	2,5	2,5	10	10	10	1,6	10	10
2,5	2,5	2,5	2,5	1,5	1,5	1,5	10	2,5	1,5
10	10	1,5	1,5	10	10	10	1,5	10	1,5
1,5	1,5	1,5	1,5	1,5	1,5	10	10	10	1,5
2,9	10	2,9	10	10	2,5	10	2,5	10	10
1,9	1,9	1,5	10	10	10	10	10	10	10
10	10	1,9	1,9	10	1,6	10	1,6	10	10
10	10	1,9	1,9	10	10	10	10	10	10
1,5	10	1,5	1,5	10	1,5	10	1,5	10	10
1,9	1,9	1,5	10	10	10	10	10	10	10
2,6	2,6	2,5	2,5	2,5	1,5	1,5	1,5	2,5	1,5
10	10	2,8	1,9	10	10	10	2,8	10	2,8
10	10	1,5	1,5	10	1,6	10	1,7	10	1,7
10	10	1,5	10	10	1,7	1,7	1,7	10	1,7
1,9	10	1,9	10	1,9	10	10	1,5	10	10
10	10	2,6	2,6	10	1,5	1,5	1,5	2,6	1,5
1,5	10	1,9	1,9	10	1,6	10	1,7	10	1,6
10	10	1,5	1,5	1,5	1,6	1,6	1,6	10	10
10	10	1,5	1,5	1,5	1,6	1,6	1,6	10	10
10	10	1,5	1,5	1,5	1,6	1,6	1,6	10	10
2,9	10	10	2,9	10	2,6	2,6	2,6	1,9	10
1,8	1,8	2,8	2,8	1,8	10	10	2,7	10	2,7
10	10	1,5	1,5	10	10	10	1,6	10	1,5
10	10	1,9	1,9	10	1,6	10	1,6	10	10
2,5	2,6	2,5	3,6	3,5	1,5	1,5	1,5	2,6	1,5
10	10	1,5	10	1,5	1,8	1,8	10	1,5	10
10	10	1,5	1,5	1,5	1,6	1,6	1,6	10	10
10	10	2,9	2,9	10	1,5	10	1,5	2,6	1,5
10	10	1,5	1,5	10	1,8	1,8	1,5	10	1,8
10	10	1,7	1,7	1,5	1,6	1,6	1,5	10	10
10	10	10	1,7	1,6	1,8	1,8	1,5	10	10
1,8	1,8	1,8	1,8	1,8	1,6	1,6	1,5	10	10
10	10	10	1,9	10	1,5	10	1,5	10	10
1,5	1,5	1,5	1,5	1,5	1,8	1,8	1,5	10	1,8
10	10	1,8	1,8	1,5	1,8	1,8	1,5	10	1,8
10	10	1,9	1,9	10	1,6	10	1,5	10	1,5
10	10	1,5	1,5	10	1,5	1,5	1,5	1,5	1,5
10	10	1,5	10	10	10	10	10	10	10
10	10	10	10	10	1,5	1,5	1,6	10	10
10	10	2,5	2,6	10	1,5	1,5	1,5	2,6	1,5
10	10	1,5	1,5	1,5	1,5	1,5	1,6	1,5	10
10	10	2,6	2,6	10	1,5	10	1,5	10	1,5

10	10	1,9	1,9	10	1,5	10	1,5	1,9	10
10	10	1,9	10	10	2,7	2,7	2,7	10	10
10	10	2,5	2,5	10	1,5	1,5	1,5	10	1,5
10	10	2,5	2,5	10	1,5	1,5	1,5	10	1,5
10	10	1,5	10	1,5	10	10	10	1,5	10
10	10	1,5	1,5	10	1,5	10	2,7	10	10
10	10	2,6	2,6	10	1,6	1,5	1,6	2,6	1,6
10	10	1,5	1,5	1,5	1,5	1,5	1,5	10	10
10	10	1,9	10	10	1,5	1,5	1,7	10	10
1,5	10	1,5	1,5	10	1,5	1,5	1,5	10	1,5
10	10	2,9	2,9	10	1,8	10	3,7	10	3,7
10	10	2,9	2,9	10	1,8	10	3,7	10	3,7
1,9	1,9	1,9	10	10	1,5	10	1,7	10	10
10	10	1,5	1,5	1,5	10	10	3,6	10	10
10	10	1,5	1,5	10	1,6	10	2,6	1,6	10
10	10	1,5	1,5	1,5	1,5	10	1,6	10	10
10	10	1,5	1,5	1,6	10	10	1,6	10	10
10	10	1,6	1,6	1,6	10	10	1,6	10	10
10	10	1,5	2,6	10	10	10	1,5	10	1,5
10	10	1,5	1,5	1,6	10	10	1,5	10	10
10	10	10	10	10	1,6	1,6	1,6	10	10
10	10	1,9	1,9	10	1,8	10	1,6	10	1,6
10	10	2,9	2,9	10	1,8	10	1,6	10	1,6
1,9	10	2,9	2,9	10	1,6	10	2,7	10	2,7
1,9	10	2,9	2,9	10	1,6	10	2,7	10	2,7
10	10	1,5	1,5	10	10	10	1,6	10	10
10	10	1,7	1,7	10	1,6	10	1,6	10	1,5
1,8	10	1,5	10	10	1,6	1,6	10	10	10
1,9	10	10	10	1,9	1,5	10	1,5	10	10
2,6	2,5	2,5	2,5	10	1,5	1,5	1,5	2,5	10
10	10	10	1,9	10	1,6	1,6	1,6	10	10
1,9	10	1,9	1,9	10	1,5	10	1,7	10	10
10	1,5	1,5	1,5	10	1,5	1,5	1,5	10	10
2,6	10	2,6	2,5	2,5	10	10	1,5	10	1,5
10	10	1,5	1,5	10	1,7	10	2,6	10	10
10	10	1,9	1,9	1,9	1,6	10	1,5	10	10
1,9	10	1,9	1,9	10	1,6	10	1,5	10	10
1,9	10	1,9	10	10	1,5	10	1,7	10	10
10	10	1,5	1,5	10	1,5	1,5	1,5	10	10
10	10	1,5	1,5	10	1,5	1,5	1,5	10	10
10	10	1,9	1,9	10	1,5	10	1,6	10	10
10	10	1,9	1,9	10	1,5	1,5	1,6	10	1,6
10	10	1,5	1,5	10	10	10	10	10	10
1,5	10	1,5	1,5	10	1,5	1,5	1,5	1,5	1,5
1,5	1,5	1,5	1,5	1,5	1,5	1,5	1,5	1,5	10
10	10	10	1,9	10	1,5	1,5	1,5	10	10
10	10	1,5	10	10	1,5	10	10	10	10

10	10	1,9	1,9	1,5	10	10	10	10	10
1,9	1,9	10	10	1,9	1,5	1,9	1,6	10	10
1,9	1,9	10	10	1,9	1,6	10	1,6	10	10
2,6	2,6	2,5	2,6	2,6	1,5	10	1,5	1,5	1,5
2,6	2,6	2,5	2,6	2,6	1,5	10	1,5	1,5	1,5
1,9	10	1,9	1,9	10	1,5	10	1,6	10	10
10	10	10	1,9	10	1,5	10	1,7	10	10
10	10	10	2,9	10	10	10	2,7	10	10
10	10	1,5	1,5	1,6	10	10	10	10	10
10	10	1,5	10	10	1,6	10	10	10	10
10	10	1,5	1,5	10	2,5	2,5	2,6	10	10
10	10	2,9	2,9	10	1,5	1,5	1,6	2,9	1,6
1,9	1,9	10	10	1,9	1,5	10	1,5	10	10
1,9	1,9	10	10	1,9	1,5	10	1,7	10	10
10	10	1,9	1,9	1,9	10	10	1,6	10	1,6
1,9	10	1,9	10	10	1,5	10	1,8	10	10
1,5	1,5	10	1,5	1,5	1,5	1,5	1,5	10	10
1,9	10	1,9	10	10	1,5	10	1,7	10	10
1,9	1,9	10	10	1,9	1,5	10	1,6	10	10
10	10	10	1,5	10	10	10	10	10	10
10	10	1,9	1,9	10	1,8	1,8	10	10	10
1,5	10	1,5	1,5	10	1,6	10	1,5	10	10
1,5	10	1,5	1,5	10	1,6	10	1,5	10	10
10	10	10	10	10	10	10	1,5	10	10
1,9	10	2,9	2,9	10	1,5	10	1,6	10	1,6
10	10	1,5	1,5	1,5	1,5	1,5	10	10	1,5
2,5	2,5	2,5	2,6	10	1,5	1,5	1,5	2,5	1,5
1,9	10	1,9	1,9	10	1,5	10	1,7	10	10
1,9	10	10	2,9	10	1,5	1,5	1,7	10	10
10	10	1,5	10	10	1,5	10	10	10	10
10	10	1,9	1,9	2,9	1,6	1,6	1,6	2,9	1,6
10	10	1,9	1,9	10	1,8	1,8	1,5	10	10
10	10	1,5	1,5	1,5	1,5	1,5	1,5	10	1,5
1,9	10	2,9	2,9	10	1,6	1,6	1,6	2,9	1,6
1,9	10	10	10	1,9	1,5	10	1,5	1,9	10
10	10	1,9	1,9	10	1,6	10	1,6	10	10
10	10	1,5	1,5	10	10	10	10	10	10
10	10	1,5	1,5	10	1,6	1,6	10	10	10
10	10	1,9	1,9	10	1,6	10	1,7	10	10
10	10	1,5	10	10	1,5	1,5	1,5	10	10
10	10	2,9	2,9	2,9	1,5	1,5	1,6	10	1,6
1,5	1,5	1,9	1,9	1,5	1,8	1,8	1,5	10	1,5
10	10	1,5	10	10	1,6	10	10	10	10
10	10	10	2,9	10	10	10	2,6	10	10
10	10	1,5	10	10	1,6	10	10	10	10
10	10	2,9	2,9	10	1,8	1,8	1,5	10	10
10	10	2,6	2,6	2,5	1,5	1,5	1,5	1,5	1,5

10	10	2,6	2,6	2,5	1,5	1,5	1,5	1,5	1,5
1,8	10	1,9	10	10	1,5	10	1,6	10	10
10	10	1,9	1,9	10	1,5	1,5	1,6	10	1,6
1,9	10	1,9	1,9	10	1,6	10	1,6	10	10
1,9	10	1,9	1,9	1,9	1,5	10	1,6	10	10
10	10	1,9	1,9	1,9	1,6	10	1,7	10	10
1,9	10	10	1,9	10	10	10	1,7	10	10
1,9	10	1,9	10	10	1,6	10	1,6	10	10
1,9	1,9	1,9	10	10	1,5	10	1,6	10	10
1,9	1,9	1,9	1,9	10	1,6	10	1,6	10	10
1,5	10	1,5	1,5	10	10	10	2,6	1,5	1,5
1,5	1,5	1,5	1,5	1,5	1,5	10	10	1,5	10
10	10	10	1,9	10	1,6	1,6	1,7	10	10
10	10	10	1,9	10	1,6	1,6	1,7	10	10
1,9	10	10	1,9	10	1,6	1,6	1,6	10	10
1,5	1,5	1,8	1,8	1,9	1,8	10	1,5	10	10
2,5	2,5	2,5	2,5	10	1,5	1,5	1,5	2,5	1,5
2,5	2,5	2,5	2,5	10	1,5	1,5	1,5	2,5	1,5
10	10	10	1,9	10	10	1,8	1,8	1,7	1,9
10	10	1,5	10	10	1,6	10	10	10	10
10	10	10	1,9	10	1,8	10	1,6	10	10
10	10	1,9	1,9	10	1,6	1,5	1,6	10	10
1,9	1,9	1,5	10	10	1,6	1,6	10	10	10
10	10	1,9	1,9	10	1,5	1,5	1,7	10	1,9
10	10	1,9	1,9	10	1,5	1,5	1,5	1,9	10
10	10	1,5	1,5	10	1,5	1,5	1,5	10	1,5
1,9	10	10	1,9	10	1,5	1,5	1,5	10	10
1,9	10	1,9	1,9	10	10	10	1,6	10	1,6
10	10	10	1,9	1,5	1,7	1,6	1,7	10	10
10	10	1,9	1,9	10	2,6	1,5	2,7	10	10
10	10	1,9	1,9	10	1,6	1,5	1,7	10	10
10	10	10	1,9	10	1,7	1,5	1,7	10	10
10	10	1,9	1,9	10	1,6	10	1,7	10	10
10	10	1,5	1,5	10	1,5	1,5	1,5	10	1,5
2,5	2,5	2,5	2,5	10	1,5	1,5	1,6	10	1,5
10	10	1,5	1,5	10	10	10	1,5	10	1,5
1,9	10	10	1,9	10	1,5	10	1,6	10	10
1,9	10	1,9	10	1,9	1,6	10	1,7	10	10
10	10	1,6	1,6	10	1,5	1,5	1,6	10	10
10	10	1,9	1,9	1,9	1,6	10	1,6	10	1,6
2,9	10	10	2,9	10	1,5	1,8	1,7	1,9	10
10	10	10	10	10	1,5	1,5	1,6	10	1,5
10	10	1,9	1,9	10	1,5	1,5	1,5	1,9	1,5
10	10	1,9	1,9	10	1,5	1,5	1,5	1,9	1,5
10	10	1,9	1,9	10	1,5	1,5	1,5	10	10
10	10	10	10	10	10	10	1,5	10	10
10	10	1,6	1,5	1,6	1,5	1,6	1,6	10	1,6

10	10	1,9	1,9	10	1,5	1,5	1,5	10	10
10	10	1,5	1,5	1,6	10	10	10	10	10
10	1,9	1,9	10	10	10	10	10	10	10
10	10	1,9	1,9	10	1,5	1,5	1,5	1,9	1,5
1,5	1,5	1,9	1,9	10	10	10	1,5	1,9	1,5
10	10	10	1,9	10	10	10	1,5	10	10
10	10	1,9	1,5	10	10	10	1,5	10	1,5
1,6	10	1,6	1,6	1,6	1,6	1,6	1,6	10	1,6
1,6	10	1,6	1,5	1,6	10	10	1,5	10	1,6
1,6	10	1,6	1,5	1,6	10	10	1,5	10	1,6
1,5	10	10	1,5	10	1,5	10	10	10	10
1,5	1,5	1,9	1,9	1,5	10	10	1,5	1,5	1,5
10	10	10	1,9	10	10	10	1,5	10	10
1,6	1,6	1,5	1,5	1,5	1,6	10	1,6	10	10
1,9	1,9	1,9	1,9	10	1,5	1,5	1,5	1,9	1,5
10	10	10	1,9	10	1,5	1,5	1,5	1,9	10
10	10	1,9	1,9	10	1,5	10	1,5	10	1,5
1,9	10	10	1,9	10	1,5	10	10	10	1,5
1,6	1,6	1,6	1,6	1,6	1,6	1,6	1,6	10	1,6
1,5	1,5	1,5	1,5	1,5	1,5	1,5	10	10	1,5
10	10	1,9	1,9	1,9	10	10	1,5	10	10
10	10	1,9	1,9	10	10	10	10	10	10
10	10	1,9	1,9	10	10	10	10	10	10
10	10	1,9	1,9	10	1,5	10	10	10	10
10	10	1,5	1,5	10	1,5	1,5	1,5	10	1,5
10	10	1,5	1,5	1,5	1,5	1,5	1,5	1,5	1,5
10	10	1,9	1,9	10	10	10	10	10	10
1,5	1,5	1,5	1,5	1,5	1,5	1,5	1,5	1,5	1,5
1,9	1,9	1,9	10	10	10	10	10	1,8	10
10	10	1,5	1,5	1,5	1,5	1,5	1,5	1,5	10
10	10	10	10	1,9	2,5	10	10	10	10
10	10	10	1,5	10	10	10	10	10	1,5
10	10	10	10	1,9	2,5	10	10	1,5	10
1,5	1,5	1,5	1,5	1,5	1,5	1,5	1,5	1,5	1,5
1,9	1,9	1,9	1,9	10	10	10	10	10	10
10	10	10	1,9	10	1,5	1,5	1,5	10	1,5
10	10	10	10	1,9	10	10	1,6	1,9	1,6
10	10	1,9	1,9	10	10	10	10	10	10
10	1,9	1,9	1,9	10	10	10	10	10	10
10	10	1	1,9	10	10	10	10	10	10
10	10	10	10	1,9	1,5	1,5	1,5	10	10
10	10	10	1,9	10	1,5	1,5	1,5	10	10
1,5	1,5	1,5	1,5	1,5	1,5	1,5	1,5	10	1,5
10	10	10	10	10	1,6	1,6	1,6	10	1,5
1,5	1,5	1,5	1,5	1,5	1,5	1,5	1,5	1,5	1,5
1,9	10	1,9	1,9	10	1,5	1,5	1,5	1,9	10
1,9	1,9	1,9	1,9	10	1,5	1,5	1,5	10	1,5

1,9	1,9	1,9	1,9	10	1,5	1,5	1,5	10	1,5
1,5	1,5	1,5	1,5	1,5	1,5	1,5	1,5	10	1,5
10	10	1,5	1,5	10	10	10	10	1,5	10
1,6	10	1,5	1,5	1,6	1,5	10	10	10	10
1,9	1,9	1,9	1,9	10	1,5	1,5	1,5	10	10
10	10	1,9	1,9	10	10	10	1,5	10	10
1,9	1,9	1,9	1,9	10	10	10	1,5	1,9	1,5
1,9	1,9	1,9	1,9	10	1,5	1,5	1,5	1,9	1,5
10	10	10	1,9	10	10	10	1,5	10	10
1,6	10	1,6	1,5	1,6	10	10	10	10	10
10	10	1,9	1,9	10	10	10	10	10	10
10	10	1,9	10	10	1,5	1,5	1,5	10	1,9
1,9	1,9	1,9	1,9	10	10	10	10	10	1,5
1,9	1,9	1,9	1,9	10	10	10	1,5	10	1,5
10	10	1,9	1,9	10	10	10	1,5	10	1,5
1,9	1,9	1,9	1,9	10	10	10	1,5	1,9	1,5
1,9	1,9	1,9	1,9	10	10	10	1,5	1,9	1,5
1,9	1,9	1,9	1,9	10	10	10	1,5	10	1,5
1,9	1,9	1,9	1,9	10	1,5	1,5	1,5	10	10
1,5	1,5	1,5	1,5	10	1,5	1,5	10	10	1,5
10	10	1,6	1,6	1,5	1,5	10	10	10	1,5
10	10	1,9	1,9	10	10	10	10	10	10
1,9	1,9	1,9	1,9	10	10	10	1,5	1,9	1,5
1,5	1,5	1,5	1,5	10	1,5	1,5	1,5	1,5	1,5
10	10	1,9	1,9	10	1,5	10	10	10	1,5
10	10	1,9	1,9	10	10	10	10	10	10
1,9	1,9	1,9	1,9	10	1,5	1,5	1,5	1,9	1,5
1,9	1,9	1,9	1,9	10	10	10	1,5	1,9	1,5
10	10	1,9	1,9	10	10	10	1,5	10	1,5
1,9	1,9	1,9	1,9	10	1,5	1,5	1,5	10	1,5
10	10	10	1,9	10	1,6	10	10	10	1,6
1,9	1,9	2,9	2,9	1,9	1,8	1,8	1,5	10	10
10	10	10	10	10	2,8	2,8	2,5	10	10
1,5	10	1,5	1,5	10	1,5	1,5	10	10	10
10	2,8	2,9	2,8	2,8	10	10	10	10	10
1,5	1,5	1,5	1,5	1,5	1,5	1,5	1,5	1,5	1,5
10	10	4,9	4,9	10	10	10	2,7	10	10
10	10	1,5	1,5	1,5	1,5	1,5	1,5	1,5	10
1,9	1,9	1,5	1,5	1,5	1,5	1,5	1,5	1,5	1,5
1,9	1,9	1,9	1,9	1,9	1,5	1,5	1,5	1,9	1,5
10	10	10	10	10	10	10	10	10	10
1,9	1,9	1,9	1,9	1,9	1,5	1,5	1,5	10	1,5
10	10	2,6	2,5	2,6	10	10	10	10	10
10	10	10	1,9	1,9	1,8	1,8	1,5	10	1,8
10	10	1,9	1,6	1,5	10	10	10	10	10
10	10	1,9	10	10	1,5	1,5	1,5	10	10
10	10	2,5	2,5	10	10	10	1,5	10	1,5

10	10	1,9	10	1,9	10	10	10	10	10
10	1,8	10	1,6	10	1,5	10	1,5	10	10
10	10	1,5	10	1,9	10	10	1,5	10	10
10	10	1,9	10	10	10	10	10	10	10
10	10	10	1,9	10	10	10	10	10	10
10	10	1,9	10	10	10	10	10	10	10
1,9	1,9	1,9	1,9	10	1,5	1,5	1,5	10	1,5
1,9	1,9	1,9	1,9	1,9	1,5	1,5	1,5	2,9	1,5
1,5	1,5	1,5	1,5	1,5	1,5	1,5	1,5	10	1,5
1,9	1,9	1,9	1,9	10	1,5	1,5	1,5	10	10
10	10	10	10	10	10	10	1,5	10	10
10	10	10	1,9	10	1,5	1,5	1,5	10	1,5
10	10	1,9	1,9	10	1,5	1,5	1,5	10	1,5
10	10	10	1,5	10	10	10	1,5	10	10
10	10	1,9	1,9	10	1,5	1,5	1,5	10	1,5
10	10	10	1,9	10	1,5	1,5	1,5	10	1,5
1,9	1,9	1,9	10	1,5	1,5	1,5	1,5	10	10
10	1,9	10	1,5	10	1,5	1,5	1,5	10	10
1,5	1,5	1,5	1,5	10	10	10	10	10	10
1,9	1,9	1,9	1,9	10	1,5	1,5	1,5	10	10
10	10	1,9	1,9	10	1,5	10	10	10	10
10	10	10	1,9	10	1,5	1,5	1,5	10	1,5
1,9	10	1,9	1,9	10	1,5	1,5	1,5	10	10
10	10	1,9	1,9	10	1,5	1,5	1,5	1,9	1,5
10	10	10	1,9	10	1,5	1,5	1,5	10	1,5
1,9	10	1,9	1,9	10	1,5	1,5	1,5	10	10
1,6	1,6	1,6	1,6	1,6	1,6	1,6	1,6	10	1,6
1,9	10	1,9	1,9	10	1,5	1,5	1,5	10	10
1,9	10	1,9	1,9	10	1,5	1,5	1,5	10	10
1,9	1,9	1,9	10	1,5	1,5	1,5	1,5	10	10
1,6	1,6	1,6	1,6	1,6	1,5	1,5	1,5	10	1,5
10	10	1,9	1,9	10	1,5	1,5	1,5	10	1,5
10	10	1,5	1,5	1,5	10	10	10	1,9	10
10	10	1,9	10	1,9	10	10	10	10	10
10	10	10	1,9	10	1,5	1,5	1,5	10	1,5
1,9	10	1,9	1,9	10	10	10	10	10	10
1,9	1,9	1,9	1,9	10	1,5	1,5	1,5	10	10
1,9	1,9	1,9	1,9	10	1,5	1,5	1,5	10	10
1,5	1,5	1,5	1,5	1,5	1,5	1,5	1,5	1,5	1,5
10	10	10	10	10	10	10	10	10	10
10	10	1,9	1,9	10	1,5	10	10	1,9	19
1,6	1,6	1,5	1,5	1,6	1,6	1,6	1,6	10	1,6
1,5	1,5	1,5	1,5	1,5	1,5	1,5	1,5	1,5	1,5
1,9	10	1,9	1,9	10	1,5	1,5	1,5	10	10
10	10	10	1,5	10	10	10	1,5	10	1,8
1,9	10	1,9	1,9	10	1,5	1,5	1,5	10	1,5
10	10	1,9	1,9	10	1,6	1,6	1,6	10	10

10	10	10	10	10	10	10	1,5	10	10
10	10	10	10	10	1,5	1,5	1,7	10	10
10	10	10	10	10	10	10	10	1,5	10
10	10	10	10	10	10	10	10	1,5	10
10	10	1,8	1,9	1,9	10	10	10	1,9	10
10	10	1,9	10	10	10	10	10	10	10
10	10	10	1,5	10	10	10	10	10	1,5
1,5	1,5	1,5	1,5	1,6	1,5	1,5	1,5	1,5	1,5
1,5	1,5	1,6	1,6	1,5	1,5	1,5	1,5	1,5	1,5
10	10	10	1,6	10	1,5	10	1,6	10	10
1,5	1,5	1,5	1,5	1,5	1,5	1,5	10	1,5	1,5
10	10	10	1,9	10	1,5	1,5	1,5	10	1,5
1,9	1,9	1,9	1,9	1,9	10	10	1,5	10	1,5
10	10	10	10	10	1,5	1,5	1,5	10	1,5
10	10	1,8	10	10	10	10	10	10	10
1,9	10	10	10	1,9	1,8	1,8	1,5	10	10
10	10	10	1,9	10	10	10	10	10	1,5
10	10	1,9	1,9	1,9	1,8	1,8	1,5	10	10
1,9	10	1,9	1,9	10	10	10	10	10	1,8
10	10	10	10	10	10	10	10	10	10
10	10	1,9	1,9	1,9	1,5	1,5	1,5	10	1,5
10	1,5	1,5	10	1,5	1,5	1,5	10	1,5	10
10	10	10	10	10	1,6	10	10	10	10
10	10	1,9	1,9	10	10	10	1,6	10	10
1,9	1,9	1,9	1,9	10	1,5	1,5	1,5	1,9	1,5
1,9	1,9	1,9	1,9	1,9	1,5	1,5	1,5	10	10
10	10	1,9	1,9	10	1,5	1,5	1,6	10	1,6
10	10	1,9	1,9	10	1,5	1,5	1,5	10	10
10	10	1,5	1,5	1,5	1,5	1,5	1,5	10	1,5
10	10	1,5	1,5	10	1,5	1,5	1,5	10	10
10	10	10	1,9	10	10	10	1,5	10	1,5
1,9	1,9	1,9	1,9	1,9	10	10	1,5	10	1,5
10	10	1,9	1,9	1,9	1,5	1,5	1,5	10	10
10	10	10	10	10	10	10	10	10	10
10	10	1,9	1,9	1,9	1,5	1,5	1,5	10	10
1,5	1,5	1,5	1,5	1,5	1,5	1,5	1,5	1,5	10
10	10	10	10	10	10	10	1,5	10	1,8
1,9	10	1,9	1,9	10	1,5	1,5	1,5	2,9	1,5
1,6	10	10	10	10	10	10	10	10	10
10	10	10	10	10	10	10	10	10	10
10	10	10	10	10	10	10	10	10	10
10	10	10	10	10	10	10	10	10	10
10	10	10	1,5	10	10	10	10	10	1,5
10	10	1,5	1,5	10	10	10	1,5	1,5	10
10	10	10	1,9	10	10	10	10	10	10
1,9	10	1,9	1,9	10	10	10	10	2,5	10
10	10	10	10	10	10	10	10	10	10

10	10	10	1,9	10	10	10	10	10	10
10	10	1,9	1,9	10	1,6	1,6	1,6	10	10
10	10	1,9	1,9	10	10	10	10	10	10
10	10	1,9	10	10	1,5	1,5	1,5	10	10
10	10	10	1,9	10	1,8	1,8	1,5	10	10
10	10	10	1,9	10	10	10	10	10	10
1,5	10	1,9	1,9	10	1,6	1,6	1,6	1,9	10
10	10	1,9	1,9	1,9	1,8	1,8	1,5	10	10
10	10	1,9	1,9	10	10	10	10	10	10
10	10	1,5	1,5	1,5	1,5	1,5	1,5	10	1,5
10	10	1,9	1,9	10	1,5	1,5	1,5	10	10
10	10	1,5	1,5	1,5	1,5	1,5	10	1,5	1,5
10	10	1,9	1,9	10	10	10	10	10	10
10	10	1,9	1,9	10	10	10	10	10	10
10	10	1,9	1,9	10	1,5	1,5	1,5	10	1,5
10	10	10	10	10	10	10	10	10	10
10	10	1,9	1,9	1,9	10	10	1,5	10	1,5
10	10	1,9	1,9	1,9	10	10	1,5	10	1,5
10	10	1,9	1,9	10	1,5	1,5	1,5	10	1,5
1,9	1,9	1,9	1,9	1,9	10	10	1,5	1,9	1,5
10	10	1,9	1,9	10	1,5	1,5	1,5	10	10
10	10	10	10	10	10	10	10	10	10
10	10	10	10	10	10	10	10	10	10
1,9	10	10	1,9	10	1,8	10	1,6	1,9	10
1,9	1,9	1,9	1,9	10	1,6	10	1,6	10	10
10	10	10	1,6	10	1,6	1,6	1,6	10	10
1,9	1,9	1,9	1,9	10	1,6	10	1,6	10	10
10	10	10	1,9	10	1,5	1,5	1,5	10	10
10	10	1,9	1,9	10	1,5	1,5	1,5	10	10
1,5	1,5	1,5	1,5	1,5	1,5	1,5	1,5	10	1,5
10	10	1,9	1,9	10	1,5	1,5	1,5	10	1,5
10	10	10	1,9	10	1,5	1,5	1,5	10	10
10	10	10	1,9	10	1,6	1,8	1,7	10	10
10	10	10	1,9	10	1,5	1,5	1,5	10	10
1,6	10	1,5	1,5	1,6	1,6	1,6	1,6	10	1,6
1,9	10	1,9	1,9	10	10	10	10	10	10
1,5	10	10	1,9	10	1,5	1,5	1,5	10	1,5
1,6	1,6	1,6	1,6	1,6	1,6	1,6	1,6	10	1,5
1,9	1,9	1,9	1,9	10	1,5	1,5	1,5	10	10
1,9	1,9	1,9	1,9	10	1,5	10	1,5	1,9	1,5
10	10	10	1,9	1,9	10	10	1,5	10	10
1,9	10	1,9	1,9	10	1,5	1,5	1,7	1,9	10
1,9	10	1,9	1,9	10	1,5	1,5	1,7	1,9	10
1,5	1,5	1,5	1,5	1,5	1,5	1,5	1,5	10	1,5
10	10	10	1,9	10	1,5	1,5	1,7	10	10
10	10	1,9	1,9	10	10	10	10	10	10

cucumber	lettuce	water	tea	rooibos	coffee	fruit juice	squashes	cold drinks	flavoured r
10	1,6	4,7	1,5	1,5	10	1,6	1,6	1,5	1,6
10	1,6	4,7	1,5	1,5	10	1,6	1,6	1,5	1,6
10	10	3,7	1,6	1,6	10	1,6	1,6	1,5	1,8
10	10	4,7	1,5	1,5	10	1,8	1,5	1,5	1,8
10	1,5	3,7	10	3,6	10	1,6	1,6	1,5	1,5
10	1,8	3,7	1,7	1,7	10	2,7	1,5	1,6	1,5
10	10	4,7	1,7	10	10	10	1,8	1,5	1,5
10	10	4,7	10	1,6	10	1,5	2,5	1,5	10
10	10	4,7	2,7	2,7	10	2,6	2,6	2,5	2,5
10	10	4,7	1,5	1,5	10	10	1,5	1,5	1,8
10	10	4,6	1,5	1,5	10	1,5	1,6	1,7	1,5
10	10	4,7	2,6	2,6	10	1,6	2,6	2,6	2,6
10	1,8	2,7	1,6	1,5	10	2,6	1,5	1,5	1,5
10	1,8	1,7	1,7	1,7	10	1,6	10	1,5	1,6
10	10	4,7	1,6	1,6	10	1,6	1,5	1,5	1,5
10	10	4,7	1,5	1,5	1,5	1,5	1,6	1,5	1,8
10	10	4,7	1,5	1,5	1,5	1,5	1,6	1,5	1,8
10	1,8	2,7	1,6	1,5	10	1,6	1,5	1,6	1,5
10	1,8	2,6	1,5	1,5	10	1,5	1,5	2,6	1,5
10	10	2,7	2,5	1,5	10	2,6	1,5	1,6	1,5
10	10	2,7	1,5	1,5	10	1,6	1,5	1,8	1,8
10	10	2,6	1,6	1,5	10	2,6	1,5	1,5	1,5
10	10	3,7	2,6	2,6	10	2,6	1,5	1,5	1,5
10	10	3,7	1,6	1,5	10	2,6	2,5	1,5	1,5
1,8	1,5	3,7	1,5	1,5	10	2,6	1,6	1,5	1,5
10	1,8	3,7	1,6	1,5	10	1,6	1,5	1,5	1,5
10	10	4,7	1,7	1,7	1,7	1,6	10	1,6	1,8
10	10	4,7	1,7	10	10	1,8	1,7	1,6	1,8
10	1,5	4,7	1,5	1,5	1,5	1,5	1,6	1,6	1,5
10	10	3,7	1,6	1,6	10	2,6	2,7	1,6	10
10	10	3,7	3,6	10	10	1,6	1,5	1,8	10
10	10	4,7	2,6	1,5	10	2,6	1,5	1,5	1,5
10	10	4,7	1,6	1,6	10	2,6	2,6	1,5	1,5
10	10	3,7	1,6	10	10	1,5	10	1,5	1,5
10	10	4,7	10	1,6	10	10	1,6	1,5	1,5
10	10	4,7	1,6	1,6	10	10	1,5	1,6	1,5
10	10	2,7	2,5	10	10	1,7	1,5	1,6	1,7
10	10	3,7	10	1,7	10	1,8	1,7	1,5	10
10	10	4,7	2,6	2,6	10	2,6	2,5	1,5	1,5
1,8	10	3,7	1,7	1,7	10	1,4	1,5	1,5	1,5
10	10	4,7	1,7	1,7	10	1,7	1,7	1,6	1,5
10	10	4,7	1,5	1,5	1,5	1,7	1,7	1,7	1,5
10	10	3,7	1,6	1,6	10	10	1,7	1,6	10
10	10	3,7	10	1,7	10	1,7	1,7	1,6	1,8
10	10	4,7	10	1,6	10	1,8	1,8	1,5	10
10	10	3,7	1,6	10	10	1,6	10	1,5	10

10	10	3,7	1,7	10	10	1,7	1,5	1,5	1,8
10	10	3,7	10	1,7	10	10	2,6	10	1,8
10	10	4,7	2,7	2,7	10	1,5	1,5	1,8	1,8
1,5	1,5	4,7	1,5	1,5	10	1,5	1,5	1,6	1,5
1,5	10	4,7	1,7	1,7	10	1,7	1,7	1,5	1,6
10	10	4,7	1,6	1,6	10	1,8	1,5	1,5	1,8
10	10	4,7	1,7	10	1,5	1,5	1,5	1,5	2,5
1,5	10	4,7	1,7	1,7	10	1,8	1,6	1,5	1,8
1,6	10	4,7	1,7	1,7	10	1,5	1,6	1,5	1,8
10	10	4,7	1,7	1,7	1,7	1,5	1,7	1,5	1,8
10	10	3,7	1,7	1,7	10	1,5	1,6	1,5	1,8
1,5	10	4,7	1,7	1,7	1,5	1,5	1,6	1,5	1,8
10	10	3,7	1,7	1,7	10	1,5	1,6	1,5	1,8
10	1,5	4,7	1,5	1,5	10	1,6	1,6	1,5	1,5
10	10	4,7	10	1,5	10	1,7	2,6	3,7	10
10	10	3,7	1,6	1,6	10	1,7	10	1,5	1,8
10	10	3,7	1,6	1,6	10	1,8	1,7	1,6	1,6
10	10	3,7	1,6	1,6	10	1,6	1,7	1,7	1,6
10	10	4,7	1,6	1,6	10	10	1,5	1,5	1,5
10	10	4,7	10	1,6	10	1,5	1,5	1,5	1,5
10	10	4,7	2,6	2,6	10	2,6	2,6	1,6	1,6
10	1,5	4,7	1,6	1,6	10	2,6	1,6	1,5	1,5
10	1,5	4,7	1,6	1,6	10	2,6	1,6	1,5	1,5
10	10	4,7	1,5	10	10	10	1,8	1,5	1,8
10	10	4,7	1,5	10	10	10	1,8	1,5	1,8
10	10	3,6	1,6	1,6	10	1,8	1,6	1,6	1,8
10	10	2,7	10	2,7	10	10	1,6	1,6	1,5
10	10	3,7	1,6	10	10	1,7	10	1,6	10
1,5	2,6	4,7	1,5	1,5	1,5	1,5	1,7	1,5	1,5
10	10	4,7	1,7	1,5	10	1,5	2,6	1,5	1,6
10	10	3,7	1,7	1,7	10	1,8	1,5	1,5	1,8
10	10	3,7	1,7	1,7	10	1,8	1,5	1,5	1,8
10	10	4,8	1,5	1,5	1,5	10	1,5	2,5	1,5
10	10	3,7	1,6	1,6	10	1,5	1,5	1,5	10
10	10	3,7	1,7	10	10	1,5	2,7	1,6	10
10	10	4,7	1,5	1,5	10	1,5	1,5	1,5	1,5
10	10	4,7	1,6	1,6	10	1,5	2,6	1,5	1,5
10	10	4,7	1,7	10	10	1,6	10	2,7	1,6
10	10	3,7	1,6	1,6	10	1,8	1,7	1,5	1,8
10	10	2,8	1,6	1,6	10	1,6	1,7	1,5	1,6
10	10	4,7	1,5	1,5	1,5	10	1,5	1,5	1,8
10	10	4,7	1,6	1,6	10	1,6	1,6	1,5	1,8
10	10	4,7	1,6	1,6	10	1,9	1,5	1,6	1,8
10	10	4,7	1,6	1,6	10	1,6	1,5	1,6	1,5
10	10	4,7	1,6	1,6	10	10	1,5	1,6	1,5
10	10	4,7	1,6	1,6	10	1,8	1,5	1,6	1,8
10	1,5	3,7	1,5	1,5	10	1,7	1,7	1,5	1,8

10	10	2,7	1,7	10	10	1,6	10	1,5	1,8
10	10	4,7	1,7	1,7	1,5	1,5	1,5	1,5	10
10	10	3,7	1,7	1,7	10	1,7	1,7	1,7	1,6
10	10	3,7	1,6	1,6	1,8	1,8	1,5	1,8	1,8
10	10	4,7	1,7	1,7	10	10	3,7	1,5	10
10	10	4,7	1,7	1,7	1,5	1,8	2,7	1,5	1,5
10	10	3,7	1,6	1,6	10	1,5	2,6	1,5	1,5
10	10	4,7	10	1,6	1,5	10	1,6	1,5	1,8
10	10	3,7	10	3,5	10	1,5	1,6	1,5	1,8
10	10	4,7	10	2,7	10	3,8	3,7	2,6	1,8
10	10	3,7	1,6	1,6	10	1,8	1,6	1,5	1,8
10	10	4,7	1,6	10	10	1,7	10	1,5	1,6
10	10	4,7	1,7	1,7	10	1,7	1,7	10	10
10	10	4,7	1,5	1,5	10	1,5	1,6	2,6	10
10	10	3,7	1,6	1,6	10	1,8	1,6	1,5	1,8
10	10	3,6	1,5	1,5	10	1,6	1,5	1,5	1,5
10	10	3,7	1,7	1,7	10	10	1,9	2,8	1,8
10	10	3,7	1,5	10	10	1,6	2,6	1,5	10
10	10	4,7	1,6	1,6	10	1,6	1,5	1,6	1,5
10	10	3,7	1,5	10	10	1,5	10	1,5	1,5
10	10	3,7	1,5	1,6	10	2,6	2,5	1,5	2,6
10	10	4,7	1,5	1,5	10	1,8	1,5	1,5	1,8
10	10	3,7	1,7	1,7	10	1,6	1,5	1,6	1,5
10	10	3,7	1,7	1,7	1,9	1,6	1,5	1,6	1,5
10	10	3,7	1,7	1,7	10	1,6	1,5	1,6	1,5
10	10	4,7	10	1,7	10	2,8	2,6	1,6	2,8
10	10	4,7	1,6	1,6	10	1,7	2,6	1,5	1,6
10	10	3,7	10	3,7	10	1,6	1,6	1,5	1,5
10	10	4,7	1,6	10	10	1,6	10	1,6	1,5
10	1,5	3,7	1,6	1,5	10	1,5	2,5	1,5	1,5
1,8	1,8	4,7	1,7	1,7	1,7	1,7	1,7	1,7	1,8
10	10	4,7	1,7	1,7	10	1,6	1,6	1,6	1,5
10	10	2,7	1,5	1,5	10	1,6	1,5	1,5	1,5
1,8	1,8	2,7	1,5	1,5	10	1,5	1,5	2,7	1,6
10	1,8	3,6	1,5	1,5	10	1,5	1,5	1,5	1,6
10	10	3,7	1,4	1,4	1,8	2,6	1,5	2,6	1,5
1,6	1,6	2,7	1,5	1,5	1,5	1,5	1,5	2,6	1,5
10	10	4,7	10	10	1,5	1,5	1,6	1,6	10
1,8	1,8	3,7	1,5	1,5	10	1,7	1,5	1,6	1,8
1,8	1,8	4,7	1,5	1,5	1,9	1,5	1,7	1,5	10
10	10	4,8	1,5	1,5	10	1,6	1,5	1,5	1,5
1,5	1,5	3,7	1,6	1,6	1,6	1,6	1,6	1,6	1,5
10	10	4,7	1,6	1,6	10	2,5	1,5	1,5	1,5
10	10	4,7	1,6	1,6	1,6	1,5	1,6	1,6	1,5
10	10	3,7	1,6	1,6	10	2,6	2,5	1,5	1,5
10	10	4,7	2,6	2,6	10	2,6	1,5	10	1,5
10	10	2,6	1,6	1,6	10	1,6	1,5	1,5	1,5

10	10	1,7	1,5	10	10	1,5	10	1,5	10
1,5	1,5	4,7	10	1,7	10	1,6	1,6	1,6	10
10	10	4,7	10	10	1,6	1,7	1,5	10	10
10	10	4,7	10	10	1,6	1,7	1,5	10	10
10	10	3,7	1,7	1,7	10	1,8	1,5	1,5	1,8
1,8	1,8	3,7	1,7	1,7	10	1,5	1,5	1,6	1,8
10	10	2,7	1,5	1,6	10	1,5	1,5	1,5	1,5
10	10	4,7	1,6	1,6	1,6	1,7	1,5	1,6	1,5
1,5	1,5	4,7	1,5	1,5	1,5	1,7	1,7	1,7	1,8
1,5	1,5	3,7	1,6	1,6	1,6	1,6	1,6	10	1,5
10	10	4,7	1,5	1,5	10	1,8	1,8	1,5	10
10	10	4,7	1,5	1,5	10	1,8	1,8	1,5	10
10	10	4,7	1,7	10	10	1,5	1,5	1,5	1,5
10	10	4,7	1,6	1,6	10	1,6	1,6	1,6	1,5
10	10	3,7	1,6	1,6	10	1,7	1,5	1,6	1,5
10	10	4,7	1,7	1,7	10	1,7	1,5	1,5	1,5
10	10	4,7	1,5	1,5	10	1,6	1,6	1,6	1,5
10	10	3,7	1,6	1,6	10	1,6	1,5	1,6	1,5
10	10	2,6	1,6	1,6	10	1,6	1,5	1,5	1,5
10	10	1,7	1,6	1,6	10	1,5	2,7	2,7	10
10	10	4,7	10	1,4	10	1,5	1,6	2,6	1,8
10	10	4,7	1,6	1,6	10	10	1,8	1,8	10
10	10	3,7	1,6	1,6	10	10	1,8	1,8	10
10	10	4,7	1,5	1,5	10	1,5	1,5	1,6	1,8
10	10	4,7	1,5	1,5	10	1,5	1,5	1,6	1,8
10	10	3,7	1,7	1,7	10	1,6	1,6	1,6	1,5
10	1,5	3,7	1,6	1,6	10	1,6	1,5	1,5	1,5
10	10	4,7	1,7	1,7	10	1,7	10	10	1,5
10	10	3,7	1,6	10	10	1,5	10	1,6	1,5
10	10	3,6	1,6	1,6	10	2,6	2,6	1,5	1,5
1,8	1,8	4,7	10	10	1,6	10	2,7	1,6	1,8
10	10	4,7	1,6	10	10	1,6	10	1,5	1,8
1,6	1,6	3,7	1,5	1,5	10	1,5	1,5	1,5	1,5
10	10	4,7	1,6	1,6	10	2,6	2,5	1,5	2,5
10	10	4,7	1,6	1,6	1,6	1,6	2,6	1,5	1,5
10	10	4,7	1,6	1,6	10	1,5	1,5	1,6	1,8
10	10	4,7	1,6	10	10	1,5	10	1,6	10
10	10	4,7	1,7	10	10	1,5	10	1,5	1,8
1,5	10	3,7	1,7	1,6	10	1,5	1,5	1,5	1,5
1,5	10	3,7	1,7	1,6	10	1,5	1,5	1,5	1,5
10	10	3,7	1,6	10	10	1,6	10	1,6	10
10	10	4,7	1,7	1,7	10	10	1,7	1,7	1,5
10	10	4,7	1,7	1,7	10	1,8	1,6	1,5	1,8
1,5	1,5	3,6	1,5	1,5	1,5	1,5	1,6	1,5	1,5
10	10	3,7	10	3,7	10	1,5	1,6	1,5	1,5
10	10	3,7	10	1,5	10	1,8	1,6	1,5	1,8
10	1,8	4,7	1,7	1,7	1,7	1,7	1,6	1,5	10

10	10	1,5	1,7	1,5	10	1,5	1,5	1,5	1,5
10	10	4,7	1,6	10	10	1,6	10	1,5	1,5
10	10	4,7	1,6	10	10	1,6	1,5	1,6	1,5
10	10	4,7	2,6	2,6	10	2,6	2,6	1,5	1,5
10	10	3,7	2,6	2,6	10	2,6	2,6	1,5	1,5
10	10	4,8	1,6	10	10	1,6	10	1,6	1,5
10	10	4,7	10	1,6	10	1,7	10	1,6	1,8
10	10	4,7	10	1,5	10	1,8	1,7	1,5	2,8
10	10	4,7	1,5	1,5	10	1,6	1,5	1,5	10
10	10	4,7	1,5	1,5	1,5	1,5	1,6	1,5	1,8
10	10	4,7	1,6	1,6	10	1,5	1,7	1,5	1,5
10	10	3,7	1,5	1,5	10	10	1,6	1,6	1,8
10	10	4,7	1,6	10	10	1,6	1,6	1,6	1,8
10	10	4,7	1,6	10	10	1,6	10	1,5	1,5
10	10	4,7	1,6	1,6	10	1,5	2,6	1,5	10
10	10	4,7	1,6	10	10	1,6	10	1,6	1,8
10	10	4,7	1,7	1,7	10	1,8	1,6	1,5	10
10	10	3,7	1,6	10	10	1,5	1,6	1,6	10
10	10	4,7	1,6	10	10	1,6	10	1,6	1,8
10	10	4,7	1,7	1,7	1,7	1,8	1,7	1,5	1,5
10	10	3,7	1,6	1,6	10	1,5	1,5	1,5	1,5
10	10	3,7	1,7	1,7	1,7	1,7	1,6	1,7	1,5
10	10	3,7	1,7	1,7	1,7	1,7	1,6	1,7	1,5
10	10	4,7	10	1,5	10	10	10	1,5	1,8
10	10	4,7	2,6	2,6	10	1,5	1,5	1,5	1,6
1,5	1,5	3,7	10	3,7	10	1,5	1,6	1,7	1,5
10	10	2,7	1,5	1,5	10	2,6	2,5	1,5	1,5
10	10	4,7	1,6	10	10	1,5	10	1,7	10
10	10	4,7	10	1,5	10	1,8	1,7	1,7	1,5
10	10	4,7	1,7	1,7	1,5	1,8	2,7	1,5	1,8
10	10	4,7	1,6	1,6	10	10	1,5	1,5	1,8
10	10	3,6	1,5	1,5	10	1,5	1,5	1,5	1,5
1,5	1,5	3,8	10	1,5	10	1,5	1,6	1,5	1,5
10	10	4,7	1,6	1,6	10	1,6	1,5	1,8	1,8
10	10	3,7	1,6	10	10	1,5	10	1,6	1,8
10	10	4,7	1,6	10	10	1,5	1,5	1,5	1,8
10	10	4,7	1,5	1,5	1,5	1,5	1,5	1,6	1,5
10	10	4,7	1,7	1,7	1,5	1,5	1,6	1,6	1,5
10	10	4,7	1,6	10	10	1,6	1,6	1,6	1,8
10	1,5	2,7	1,6	1,6	10	1,5	1,6	1,5	1,5
1,8	1,8	4,7	1,7	1,7	10	1,8	1,8	1,8	1,8
10	10	4,7	1,6	1,6	10	1,6	1,7	1,6	1,7
10	10	3,8	1,6	1,6	1,6	1,6	1,7	1,5	1,8
10	10	4,7	1,6	10	10	10	1,6	1,6	10
10	10	4,7	1,7	1,7	1,7	1,5	1,7	1,5	1,8
10	10	3,7	1,8	1,8	1,8	2,7	2,7	1,6	1,5
10	1,5	2,7	2,6	2,6	10	1,6	1,5	1,6	1,5

10	1,5	2,7	2,6	2,6	10	1,6	1,5	1,6	1,5
10	10	4,7	1,6	10	10	1,5	10	1,5	10
10	10	4,7	1,6	1,6	10	1,5	1,5	1,5	1,8
10	10	3,7	1,7	10	10	1,6	1,5	1,6	1,8
10	10	4,7	1,6	10	10	1,6	10	1,6	10
10	10	4,7	1,6	10	10	1,6	10	1,6	1,8
10	10	3,7	1,6	10	10	1,6	10	1,6	1,8
10	10	3,7	1,6	10	10	1,6	10	1,6	10
10	10	4,7	1,6	10	10	1,5	10	1,6	10
10	10	4,7	1,5	10	10	1,5	10	1,6	1,8
10	10	2,7	1,5	1,5	1,5	1,6	1,6	1,6	1,5
10	10	4,7	10	1,7	10	1,5	1,7	1,5	1,8
10	10	4,7	10	1,7	10	10	1,7	1,5	10
10	10	4,7	10	1,7	10	10	1,7	1,5	10
10	10	4,7	10	1,6	10	1,8	2,6	1,5	1,8
10	10	3,7	1,5	1,5	10	1,5	1,7	1,6	10
10	1,8	3,7	1,6	1,5	10	2,7	2,5	1,5	1,5
10	1,8	3,7	1,6	1,5	10	2,7	2,5	1,5	1,5
10	10	4,7	10	1,6	10	1,8	1,6	1,5	1,8
10	10	4,7	1,7	1,7	10	1,6	1,6	1,5	1,5
10	10	4,7	1,7	10	10	2,8	1,5	1,5	1,8
10	10	3,7	10	1,6	10	1,5	1,6	1,5	1,8
10	10	4,7	1,7	1,7	1,5	1,5	1,6	1,5	1,8
1,9	1,9	4,7	10	1,5	10	1,5	1,7	1,5	1,8
10	10	4,7	10	1,5	10	1,5	1,7	1,5	1,8
10	10	4,7	10	1,7	10	1,6	1,6	1,5	1,8
10	10	4,7	10	1,6	10	1,8	2,6	1,5	1,8
10	10	4,7	1,7	1,7	10	10	1,6	1,6	10
10	10	4,7	1,7	10	10	1,8	1,6	1,5	1,8
1,8	1,8	4,7	1,6	1,6	10	1,8	1,6	1,6	1,8
10	10	4,6	10	1,5	1,5	1,6	1,6	1,5	1,8
10	10	4,7	10	1,7	1,5	10	1,7	1,5	1,9
10	10	4,7	10	1,7	10	1,5	10	1,6	1,5
1,5	1,5	4,7	10	1,6	10	1,5	1,5	1,5	1,5
10	1,8	2,7	1,5	1,5	10	2,6	2,6	1,6	1,5
10	10	3,7	1,5	1,5	10	1,5	1,5	1,5	10
10	10	4,7	1,5	1,5	10	10	1,6	1,5	1,8
10	10	4,7	10	1,6	10	1,5	10	1,6	1,8
1,5	10	4,7	1,6	1,6	10	1,7	1,6	1,6	1,5
10	10	4,7	1,6	1,6	10	10	1,5	1,5	1,8
1,8	1,8	4,7	10	1,6	10	1,6	10	1,5	1,8
1,5	1,5	4,7	10	1,6	10	1,5	2,7	1,5	1,5
10	10	4,7	1,6	1,6	10	10	1,6	10	1,8
10	10	4,7	1,6	1,6	10	10	1,6	10	1,8
10	10	4,7	1,5	1,5	1,5	1,6	1,6	1,5	1,5
10	10	4,7	1,5	1,5	1,5	1,6	1,6	1,5	1,5
10	10	1,7	1,6	1,6	10	1,8	10	1,6	1,6

10	10	4,7	1,6	1,6	1,6	1,7	1,6	1,5	1,5
10	10	1,7	1,6	10	10	1,8	10	1,5	1,5
10	10	4,7	1,7	1,7	10	1,5	1,5	1,5	1,5
1,5	1,5	4,7	1,6	1,6	10	1,6	1,5	1,6	1,8
10	10	4,7	1,7	1,5	10	1,5	1,5	1,5	1,5
10	10	4,7	1,6	1,6	10	1,6	1,6	1,5	1,5
10	10	4,7	1,7	1,7	10	10	1,5	1,5	1,5
10	10	1,7	1,5	1,5	1,5	1,5	1,6	1,5	1,6
10	10	1,7	1,5	1,5	1,6	1,6	10	1,5	1,5
10	10	1,7	1,5	1,5	1,6	1,6	10	1,5	1,5
1,5	10	3,7	1,5	1,5	10	1,7	1,5	10	10
1,5	1,5	4,7	1,5	1,5	10	1,5	1,5	1,5	1,5
10	10	4,7	1,5	1,5	10	1,6	1,6	1,5	1,5
10	10	1,7	1,5	1,5	1,6	1,6	1,6	1,6	1,5
10	10	4,7	1,6	1,6	10	1,6	1,5	1,6	1,8
10	10	4,7	1,6	1,6	1,6	1,5	1,7	1,5	1,5
10	10	4,7	1,6	1,6	10	1,5	1,5	1,6	1,8
1,5	1,5	3,7	1,7	1,7	10	1,6	1,7	1,8	10
10	10	1,7	1,6	1,5	1,5	1,5	1,6	1,6	1,6
1,5	1,5	4,7	1,6	1,6	1,6	1,5	1,5	1,5	1,5
10	10	4,7	1,5	1,5	10	10	1,6	1,5	1,8
10	10	4,7	1,7	1,7	10	10	2,7	1,5	1,8
10	10	2,7	1,7	1,7	10	1,8	1,6	1,5	1,8
10	10	2,7	1,7	1,7	10	1,8	1,6	1,6	1,8
1,5	1,5	4,7	1,7	1,7	1,7	1,8	1,5	1,5	1,5
1,5	1,5	1,7	1,6	1,6	1,6	1,5	1,5	1,5	1,5
10	10	2,7	1,7	1,7	10	10	2,7	1,6	1,5
10	10	4,7	1,7	1,7	1,7	1,5	1,5	1,5	1,5
10	10	4,7	2,7	2,7	10	2,8	2,7	1,8	1,8
10	10	4,7	1,5	1,5	1,5	1,5	1,5	1,5	1,5
10	10	3,7	1,5	1,5	10	1,5	1,5	1,5	1,5
1,5	1,5	4,7	1,5	1,5	1,5	1,5	1,5	1,5	1,5
10	10	3,7	1,5	1,5	10	1,5	1,5	1,5	1,5
10	10	4,7	1,6	1,6	1,6	1,5	1,5	1,5	1,5
10	10	4,7	2,7	2,7	10	10	1,7	1,5	1,8
1,5	1,5	4,7	1,5	1,5	10	10	1,6	1,6	1,5
10	10	4,7	1,6	1,6	10	1,8	1,6	1,5	1,5
10	10	4,7	1,7	1,7	10	10	1,7	1,5	1,5
10	10	4,7	1,7	1,7	1,8	10	1,7	1,5	1,8
10	10	4,7	1,7	1,7	10	1,8	1,7	1,5	1,8
1,5	1,5	4,7	1,5	1,5	10	1,5	2,6	1,5	1,6
10	10	4,7	1,5	1,5	10	10	1,5	1,5	1,5
10	10	4,7	1,5	1,5	10	1,5	1,5	1,5	1,5
1,5	1,5	3,7	1,5	1,5	1,5	1,5	1,5	1,5	1,5
10	10	4,7	1,5	1,5	1,5	1,5	1,5	1,5	1,5
10	10	4,7	1,7	1,7	10	1,7	1,6	1,5	1,5
10	10	4,7	1,6	1,6	10	10	1,8	1,6	1,8

10	10	4,7	1,6	1,6	10	10	1,6	1,5	1,8
1,5	1,5	4,7	1,6	1,6	1,6	1,5	1,5	1,5	1,5
10	10	4,7	1,7	1,7	1,7	1,7	1,6	1,6	1,6
10	10	1,7	1,6	10	1,5	1,8	10	1,6	1,6
10	10	4,7	1,6	1,6	1,5	1,7	1,6	1,5	1,5
10	10	1,6	1,5	1,5	10	1,5	1,5	1,7	1,5
10	10	4,7	1,6	1,6	10	10	1,6	1,5	1,8
10	10	4,7	1,6	1,6	10	10	1,6	1,5	1,8
10	10	4,7	1,5	1,5	1,5	1,5	1,5	1,5	10
10	10	1,6	1,6	10	10	2,7	10	1,6	1,6
10	10	2,7	1,7	1,7	10	1,8	2,7	1,5	1,5
1,9	1,9	4,7	1,5	1,5	1,5	1,6	1,5	1,5	1,8
10	10	4,7	1,6	1,6	10	1,6	1,5	1,6	10
10	10	4,7	1,6	1,6	10	10	1,6	1,5	1,8
10	10	4,7	1,6	1,6	10	10	1,6	1,6	1,8
10	10	4,7	1,6	1,6	10	10	1,5	1,5	10
10	10	4,7	1,6	1,6	10	10	1,5	1,5	10
10	10	4,7	1,6	1,6	10	10	1,6	1,6	1,8
10	10	4,7	1,6	1,6	1,5	1,7	1,6	1,5	1,5
1,5	1,5	4,7	1,6	1,6	1,6	1,5	1,5	1,5	1,5
10	10	1,7	1,6	1,5	1,5	1,5	10	1,5	1,5
10	10	2,7	1,7	1,7	10	10	2,6	2,5	2,5
10	10	4,7	1,6	1,6	10	10	1,6	1,6	1,8
1,5	1,5	4,7	1,6	1,6	1,6	1,6	1,5	1,5	1,5
10	10	4,7	1,6	1,6	10	10	1,6	1,6	1,8
1,8	1,8	2,7	1,7	1,7	10	10	1,7	1,5	1,6
10	10	4,7	1,6	1,6	10	10	1,5	1,5	1,8
10	10	4,7	1,6	1,6	10	10	1,5	1,6	1,8
10	10	4,7	1,6	1,6	10	10	1,6	1,5	1,8
10	10	4,7	1,6	1,6	10	10	1,6	1,5	1,8
10	10	3,7	2,7	10	10	10	10	2,5	10
10	10	4,7	1,7	1,7	1,7	10	1,7	1,6	1,8
10	10	1,7	1,5	1,5	10	10	1,6	1,5	1,5
10	1,5	4,8	10	1,6	10	1,5	1,5	1,5	1,5
10	10	3,7	2,7	2,7	2,7	2,7	2,7	2,5	1,8
10	10	4,7	1,6	1,6	1,6	1,5	1,5	1,5	1,5
10	10	4,7	2,7	2,7	2,7	10	2,7	1,6	1,5
10	1,5	3,7	10	1,7	10	1,5	1,5	1,5	1,5
1,5	1,5	4,7	1,7	1,7	1,5	1,5	1,5	1,5	1,5
10	10	4,7	1,6	1,6	1,6	1,5	1,5	1,5	1,5
10	10	1,7	1,5	1,5	1,5	1,5	10	2,6	1,6
10	10	4,7	1,6	1,6	1,6	1,5	1,5	1,5	1,8
10	10	1,7	1,5	1,5	1,5	2,8	10	1,5	1,5
1,8	1,8	4,7	1,5	1,5	10	10	1,7	1,6	1,5
10	10	3,7	1,5	1,6	1,5	10	10	1,6	10
1,5	1,5	4,7	1,5	1,5	1,5	1,5	1,6	1,5	1,8
10	10	4,7	1,6	1,6	1,5	1,5	1,5	1,5	1,5

10	10	2,7	1,7	1,7	10	10	1,6	1,6	1,5
10	10	3,7	1,7	1,7	10	1,5	1,5	1,6	1,7
10	10	4,7	1,5	1,5	1,5	1,5	1,5	1,5	1,5
10	10	2,7	1,7	1,7	10	10	1,6	1,5	1,5
10	10	2,7	1,7	1,7	10	10	1,6	1,6	1,5
10	10	2,7	1,7	1,7	10	10	1,6	1,5	1,5
10	10	4,7	1,6	1,6	1,6	1,6	1,5	1,5	1,8
10	10	4,7	1,6	1,6	1,6	1,5	1,5	1,6	1,8
10	10	5,7	1,6	1,6	1,6	1,5	1,5	1,5	1,5
10	10	4,7	1,5	1,5	1,5	1,6	1,6	1,5	1,5
10	10	4,7	1,7	1,7	1,7	1,6	1,7	1,6	1,5
1,5	1,5	4,7	1,5	1,5	10	1,5	1,5	2,6	1,5
1,5	10	4,7	1,6	1,6	10	1,8	1,7	1,5	10
10	10	3,7	1,7	1,7	10	10	2,7	2,6	1,8
1,5	10	4,7	1,6	1,6	10	1,8	1,7	1,5	10
1,8	1,8	2,7	1,5	1,5	10	1,5	1,6	1,7	10
10	10	4,7	10	10	10	2,7	2,7	1,6	1,5
1,8	1,8	3,7	1,5	1,5	10	1,5	2,5	1,5	10
10	10	4,7	1,7	1,7	1,7	1,5	1,5	1,5	1,5
10	10	4,7	10	10	10	1,7	2,7	1,6	1,5
1,8	10	4,7	1,7	1,7	1,8	1,5	2,7	1,6	1,5
1,5	1,5	4,7	1,5	1,5	10	10	1,5	2,6	1,5
10	10	4,7	1,5	1,5	10	1,5	2,5	1,5	1,5
1,8	1,8	4,7	1,6	1,6	10	1,5	1,5	1,6	1,8
1,5	1,5	4,7	1,5	1,5	1,5	1,5	1,5	1,5	1,5
10	10	4,7	1,5	1,5	10	1,5	2,6	1,5	1,5
10	10	1,7	1,5	1,5	1,5	1,5	1,5	1,5	1,5
10	10	4,7	1,5	1,5	1,6	1,5	1,6	1,5	1,5
10	10	4,7	1,5	1,5	10	1,6	2,6	1,5	1,5
10	10	4,7	1,5	1,5	10	1,6	2,6	1,5	1,5
1,6	1,6	1,7	1,5	1,5	1,5	1,5	1,6	1,5	1,5
1,5	1,5	4,7	1,6	1,6	1,6	1,6	1,6	1,6	1,8
10	10	4,7	1,7	1,7	10	1,5	1,5	1,5	1,5
10	10	4,7	1,7	1,7	10	1,8	1,5	1,5	1,5
1,5	1,5	4,7	1,5	1,5	1,5	1,5	1,5	1,5	1,5
10	10	4,7	1,7	1,7	1,7	1,5	1,7	1,5	1,8
10	10	4,7	1,6	1,6	1,6	1,5	1,5	1,5	1,5
10	10	4,7	1,6	1,6	1,6	1,5	1,5	1,5	1,5
10	10	4,7	1,6	1,6	1,6	1,5	1,5	1,5	1,5
10	10	1,7	1,5	1,5	10	1,5	1,5	1,5	1,5
19	19	2,7	1,5	1,5	10	1,5	1,5	1,5	1,5
10	10	1,7	1,5	1,5	1,5	1,6	1,6	1,5	1,6
10	10	4,7	1,6	1,6	1,6	1,5	1,5	1,5	1,5
10	10	4,7	1,5	1,5	10	1,5	1,6	1,5	1,5
10	19	4,7	1,7	1,7	10	1,5	1,6	1,5	2,5
1,5	1,5	4,7	1,7	1,7	1,7	1,6	2,6	1,6	1,5
10	1,8	4,7	1,6	1,6	10	1,6	1,6	1,5	1,5

10	10	1,6	1,6	1,6	10	1,5	1,5	1,6	1,5
10	10	1,6	1,6	1,6	10	1,5	1,5	1,6	1,5
10	10	4,7	1,7	1,7	10	1,5	1,5	1,5	1,5
10	10	4,7	1,7	1,7	10	1,5	1,5	1,8	1,5
10	10	4,7	1,7	1,7	10	1,5	1,5	1,5	1,5
10	1,8	2,7	2,7	2,7	10	1,6	2,7	1,5	1,8
10	10	3,7	1,7	1,7	10	10	1,6	1,8	1,9
1,5	1,5	4,7	1,7	1,7	1,7	1,6	1,6	1,6	1,6
1,5	1,5	4,7	1,7	1,7	1,7	1,6	1,6	1,6	1,6
10	10	3,7	1,7	1,7	10	10	3,7	1,5	1,8
10	10	4,7	10	1,7	10	1,6	1,6	1,5	1,5
2,5	2,5	1,7	1,6	1,5	1,5	1,5	1,5	1,5	10
10	10	4,7	1,7	1,7	1,7	1,5	1,5	1,6	1,8
10	10	4,7	10	1,6	10	1,6	1,5	1,5	1,5
10	10	2,7	1,7	1,7	10	2,6	1,6	1,5	1,8
10	10	1,7	1,5	1,5	1,5	10	1,6	1,5	1,7
10	10	3,7	1,7	1,7	10	1,8	1,5	1,6	10
10	10	1,7	1,5	1,5	10	1,5	1,5	1,6	1,5
10	10	2,7	2,7	2,7	1,5	2,7	2,7	2,7	1,8
10	10	3,7	1,5	2,5	10	10	10	1,6	2,6
1,5	1,5	1,7	1,7	1,7	10	10	10	1,7	1,6
1,5	1,5	3,7	10	1,5	10	1,5	1,5	1,5	1,5
1,8	1,8	1,7	1,5	1,5	10	10	1,6	1,6	1,5
10	10	4,7	1,6	1,6	10	1,5	1,6	1,5	1,5
10	10	4,7	1,5	1,5	10	10	1,5	1,5	1,8
10	10	4,7	1,6	1,6	1,5	1,6	1,6	1,6	1,6
10	10	4,7	1,7	1,7	1,7	1,5	1,5	1,5	1,8
10	10	4,7	1,7	10	10	10	1,7	1,5	1,5
1,5	1,5	3,7	10	1,7	10	1,6	1,6	1,5	1,5
1,5	1,5	4,7	1,6	1,6	10	1,6	1,6	1,5	10
10	10	4,7	1,7	1,5	10	1,5	1,5	1,5	1,5
10	10	4,7	1,6	1,6	10	10	1,5	1,6	1,8
10	10	3,7	1,7	1,7	10	1,5	2,6	3,5	1,8
10	10	1,7	1,7	1,7	10	1,6	1,5	1,5	1,5
10	10	3,7	1,7	1,7	10	1,5	2,6	3,5	1,8
1,5	1,5	3,7	10	1,5	10	1,5	1,5	1,5	1,5
10	1,8	1,7	1,5	1,5	10	1,6	1,5	1,5	1,5
10	10	4,7	1,6	1,6	10	10	1,6	1,5	1,8
10	10	3,7	1,7	1,7	10	1,8	1,7	1,7	10
10	10	4,7	1,5	1,5	1,5	1,5	1,5	1,5	1,5
10	10	2,7	1,7	1,7	10	10	1,5	1,5	1,5
10	10	2,7	1,7	1,7	10	10	1,6	1,6	1,5
10	10	3,7	2,6	2,6	10	1,5	1,5	1,5	10
10	10	4,7	1,7	1,7	1,6	1,5	1,5	1,5	1,5
10	10	2,7	1,7	1,7	1,6	10	1,6	1,5	1,5
10	10	3,6	1,6	2,6	10	2,6	2,6	1,5	1,5
10	10	2,7	1,7	1,7	10	10	1,6	1,5	1,5

10	10	4,7	1,7	1,5	10	1,5	1,5	1,5	1,5
10	10	3,7	1,7	1,7	1,7	1,6	1,6	1,6	2,5
10	10	2,7	2,7	2,7	2,7	2,5	2,7	1,5	1,8
10	10	4,7	1,6	1,6	10	10	1,6	1,5	1,5
1,8	1,8	1,7	1,5	1,5	1,5	1,5	1,6	1,6	1,6
10	10	2,7	1,7	1,7	10	10	2,7	2,5	2,5
10	10	4,7	1,6	1,6	10	1,6	1,6	1,5	1,5
1,5	1,5	1,6	1,5	1,5	1,5	1,7	1,5	1,6	10
10	10	2,7	1,7	1,7	10	10	1,5	1,5	1,5
1,5	1,5	4,7	1,6	1,6	1,6	1,5	1,5	1,5	1,5
10	10	4,7	1,5	1,5	1,5	10	1,6	1,5	1,6
10	1,5	4,7	10	1,7	10	1,5	1,5	1,5	1,5
10	10	2,7	1,7	1,7	10	2,7	2,7	1,5	1,5
10	10	2,7	1,7	1,7	10	2,7	2,7	1,5	1,5
10	10	4,7	1,6	1,6	1,6	1,5	1,5	1,5	1,8
10	10	4,7	1,5	1,5	1,5	1,5	1,6	1,5	1,5
10	10	4,7	1,7	1,7	1,7	1,6	1,6	1,6	1,5
10	10	4,7	1,7	1,7	1,7	1,6	1,6	1,6	1,5
10	10	4,7	1,6	1,6	10	1,5	1,5	1,5	1,8
10	10	4,7	1,7	1,7	1,7	10	1,5	1,6	1,8
10	10	4,7	1,7	1,7	1,5	1,6	1,6	1,5	1,5
10	10	4,7	1,5	1,5	1,5	1,5	1,5	1,5	1,5
10	10	4,7	1,7	1,7	10	1,5	10	1,6	1,5
10	10	3,7	1,7	10	10	10	1,5	1,8	10
10	10	4,7	1,7	1,7	10	1,8	1,6	1,5	1,9
10	10	4,7	1,6	1,6	10	1,5	1,7	1,5	10
10	10	4,7	10	1,7	10	1,8	1,6	1,5	1,9
10	10	4,7	1,6	1,6	10	1,6	1,5	1,5	1,5
10	10	4,7	1,5	10	10	10	1,6	1,5	1,5
10	1,5	4,7	10	1,6	10	1,5	1,5	1,5	1,5
1,5	1,5	4,7	1,6	1,6	1,6	1,6	1,5	1,5	1,8
1,5	1,5	3,7	1,5	1,5	1,5	1,5	1,5	1,5	1,5
10	10	3,7	10	1,7	10	1,5	1,6	1,6	1,8
1,5	1,5	3,7	1,5	1,5	1,5	1,5	1,5	1,5	10
10	10	1,7	1,5	1,5	10	1,6	10	1,5	1,6
10	10	4,7	1,7	1,7	1,8	1,5	1,7	1,6	1,5
1,5	1,5	4,7	1,5	1,5	1,5	1,5	1,5	1,5	1,5
10	10	1,7	1,5	1,5	10	1,6	1,5	1,5	1,6
10	10	4,7	1,7	1,7	10	1,6	1,6	1,5	1,6
1,5	1,5	4,7	1,6	1,6	1,6	1,5	1,6	1,6	1,8
10	10	4,7	1,5	1,5	10	1,5	1,5	2,5	1,5
10	10	3,7	1,7	1,7	10	1,8	1,6	1,5	10
10	10	3,7	1,7	1,7	10	1,8	1,6	1,5	10
10	1,5	4,7	10	1,6	10	10	1,5	1,5	1,5
10	10	4,7	1,7	1,7	10	1,8	1,6	1,5	1,8
10	10	4,7	1,8	1,8	10	10	1,7	1,6	1,9

Moisture (	Energy (kJ)	Nitrogen (g	Total prote	Plant prote	Animal prc	Total fat (g	Carbohydr	Ca (mg)	Fe (mg)
845	3943	0	33	11	22	22	110	232	2
983	4529	0	37	9	28	27	117	246	4
505	3115	0	22	13	9	10	99	84	3
769	3957	2	27	9	17	19	123	402	4
783	3398	0	31	9	22	17	90	185	3
771	2850	0	19	8	11	9	85	200	2
531	1930	0	17	5	12	3	51	70	1
680	2997	0	24	7	17	14	83	197	2
761	4392	2	39	17	22	16	139	206	10
617	3045	0	24	8	16	12	89	175	4
901	3991	1	38	8	30	22	110	629	5
1083	3749	0	24	11	13	17	107	258	4
858	5257	0	44	21	23	49	113	214	3
594	3077	0	24	8	16	15	91	217	2
784	4160	0	28	8	19	21	127	236	4
959	3714	1	23	6	16	14	114	124	2
876	3435	1	28	6	22	15	99	194	2
942	4873	2	53	16	37	27	120	414	13
931	4688	1	36	10	26	26	132	214	4
733	3074	0	23	6	17	15	85	160	2
838	4365	0	24	11	12	18	144	182	5
664	3960	1	35	13	22	24	93	179	4
878	4518	1	45	12	33	29	111	290	5
1144	5212	2	46	10	36	34	124	471	11
920	5889	2	51	19	32	58	112	282	6
1055	4909	0	37	9	28	30	128	452	3
593	2235	0	8	4	4	7	78	59	1
1090	5061	3	40	17	23	16	174	346	7
932	3327	1	27	6	21	21	85	168	2
863	5099	1	43	24	19	20	159	272	8
1075	4326	2	28	13	15	22	137	264	5
933	3791	0	25	13	12	30	92	245	3
1042	3743	0	31	15	16	19	107	169	6
1281	4107	1	23	17	6	17	139	233	6
885	2478	0	16	7	9	11	77	61	4
792	1524	0	12	2	10	6	39	61	0
1272	3943	0	30	9	21	24	111	416	2
859	4669	2	33	15	18	18	155	134	5
1076	3718	0	22	9	13	23	109	98	3
1577	3873	0	27	11	16	24	113	263	4
1160	4406	2	28	16	11	15	151	124	5
926	4437	2	35	13	22	27	115	192	7
785	4277	1	33	14	19	28	107	330	3
838	3201	0	22	9	13	14	91	96	3
1611	4820	2	44	15	29	16	156	255	6
811	2809	2	23	9	14	14	71	393	3

741	2834	0	16	9	7	7	97	202	6
1033	4621	2	32	17	15	14	161	164	6
879	4228	4	43	12	31	17	116	246	14
1426	4962	2	52	18	34	27	122	568	7
1571	5715	3	44	16	28	26	170	299	7
995	4090	0	27	11	16	21	119	263	5
972	3758	1	35	5	30	23	81	258	2
617	4929	3	44	18	26	39	121	219	5
724	5011	1	41	12	29	36	133	289	4
786	5154	3	39	8	31	43	124	854	7
590	2966	1	21	7	14	16	77	82	2
876	4204	2	29	11	17	22	121	207	3
1240	5603	2	35	20	14	29	184	100	4
692	4402	5	58	10	48	27	103	167	15
652	3573	1	21	10	10	13	121	71	3
712	5507	2	38	8	30	24	188	608	3
1005	5444	1	51	10	41	23	175	764	6
569	3960	1	30	9	20	28	99	187	3
773	3226	1	31	7	24	17	80	179	3
775	4206	2	43	15	28	19	115	206	6
441	5075	1	47	24	23	44	109	135	5
622	4446	3	49	15	34	25	115	323	13
938	5528	2	54	18	36	40	126	410	13
508	1829	0	15	2	13	10	34	99	2
486	1559	0	12	0	12	9	26	88	2
1029	4112	1	30	12	18	19	125	276	4
729	3911	0	19	15	4	10	148	132	4
691	2719	0	10	7	3	6	99	140	3
436	3631	1	42	11	31	26	75	120	7
895	3977	1	41	11	30	11	129	295	9
553	2868	1	27	8	19	11	80	192	4
751	3007	2	31	7	24	15	80	148	4
641	4385	0	40	20	19	28	117	150	4
1102	4879	2	47	11	35	31	117	375	4
867	4021	1	29	13	16	12	139	170	4
565	5523	0	41	27	13	39	147	177	4
923	3587	0	26	8	18	18	109	316	2
738	4040	1	24	10	14	15	146	429	3
573	4708	1	37	18	18	34	120	197	4
821	4998	2	52	16	36	33	124	379	8
490	2031	0	13	6	7	6	57	77	1
731	3118	0	24	9	15	16	85	178	3
1021	3716	1	19	9	10	12	117	151	3
884	3669	2	39	6	33	25	83	285	1
620	2954	0	31	6	25	26	51	197	1
582	2681	0	25	7	18	16	60	231	1
619	4514	1	29	26	2	33	117	298	7

554	2733	0	17	5	12	15	74	115	2
1468	3486	0	22	5	17	25	79	126	2
1469	5567	2	47	8	37	35	136	414	5
1021	3716	1	19	9	10	12	117	151	3
1342	4858	1	37	12	25	24	141	470	4
753	5084	5	48	6	41	55	96	272	30
494	5696	5	49	11	38	73	88	157	32
993	4904	3	49	5	44	51	68	368	19
1013	4425	1	42	8	33	23	107	326	8
918	4616	2	42	18	24	20	130	178	6
753	2797	0	29	7	22	12	78	259	3
626	3116	0	19	9	9	15	98	122	4
738	4182	2	35	7	28	38	88	219	5
626	3774	0	23	14	9	10	140	174	5
751	3108	0	19	7	12	17	93	91	2
525	4363	0	26	18	8	40	104	194	2
851	4525	2	56	7	49	37	96	701	6
972	5873	0	38	13	24	32	182	172	4
831	3325	0	19	6	13	21	89	210	4
648	3109	0	22	11	11	6	105	211	4
854	4201	1	36	11	25	18	131	391	5
681	4127	4	51	7	42	31	83	98	3
895	4157	1	30	14	15	15	132	338	4
930	4661	1	32	14	18	23	135	370	4
1003	4670	1	32	14	18	23	135	421	4
1148	5021	2	52	11	40	27	130	419	9
903	4252	0	27	10	17	23	117	171	3
732	4282	2	32	12	20	22	125	138	4
611	3594	0	25	12	12	18	107	141	5
1096	5343	2	34	21	13	37	140	324	12
822	4567	3	41	18	23	22	131	156	7
859	5865	2	38	23	14	35	174	460	7
971	5814	1	41	27	13	47	142	419	6
813	3873	0	29	11	18	20	96	233	2
532	3986	1	32	12	20	20	109	206	4
598	2926	2	30	8	22	9	82	171	5
834	4789	1	37	17	19	22	131	200	5
620	3159	1	27	8	19	14	91	92	5
504	3068	0	31	7	24	15	77	123	4
759	4277	1	32	15	16	15	136	165	6
424	2057	0	15	4	11	8	51	112	2
153	1126	0	11	0	11	6	19	77	0
301	2515	0	19	6	12	11	87	196	3
362	3929	0	35	14	21	29	97	245	4
293	2825	3	33	3	29	19	61	61	1
297	2826	1	24	10	14	6	100	217	3
358	3189	1	32	5	27	26	58	314	2

145	1358	0	12	0	11	10	17	124	3
285	3895	2	35	7	28	38	85	147	3
426	2907	0	22	6	16	20	70	86	1
440	2773	4	48	5	43	13	53	264	9
445	4386	1	31	11	19	23	135	263	6
300	4571	1	29	13	15	18	145	128	5
620	4656	1	47	9	38	38	94	214	1
422	4124	0	28	13	14	14	124	270	4
403	4259	1	28	16	12	23	135	95	5
544	5331	2	40	9	30	42	120	215	19
658	5530	2	42	8	34	58	109	270	2
488	3325	3	30	7	23	19	89	203	2
630	3057	0	16	8	8	7	101	219	4
460	5534	7	58	11	46	38	140	139	6
387	2832	2	31	6	25	13	67	122	4
440	3704	2	33	8	23	22	99	101	2
761	5043	0	30	16	13	24	154	197	4
595	4208	0	19	14	5	27	117	120	2
802	5413	3	56	22	34	32	144	469	13
674	3089	0	36	7	29	17	75	434	4
721	4998	2	63	15	48	26	130	355	8
671	3300	1	36	15	21	11	104	419	6
696	4017	2	40	16	24	24	119	273	7
714	2911	4	52	5	47	17	53	334	4
717	3410	1	41	5	36	29	59	293	5
666	3531	0	30	12	18	15	103	178	4
858	3895	1	26	15	11	15	130	226	5
550	4073	0	36	17	19	20	120	277	6
558	3235	1	18	14	4	6	123	98	5
829	5471	5	68	16	52	30	139	428	13
548	3558	1	35	13	22	11	121	314	6
438	2814	1	26	8	18	11	79	255	4
414	3422	1	29	14	15	8	121	148	7
811	5791	2	50	27	23	43	148	509	7
497	2354	0	25	9	16	8	66	191	2
625	2713	1	22	10	12	9	86	87	3
301	2109	0	12	8	4	4	79	35	3
306	1521	0	10	5	5	6	44	133	1
386	2608	1	27	7	20	10	76	190	6
464	2996	0	27	13	14	18	84	182	2
396	2580	1	16	9	7	6	96	79	4
537	3277	3	35	9	26	30	66	121	5
622	4811	4	35	21	14	12	176	150	8
487	2868	0	15	9	6	11	93	118	3
537	2397	0	13	10	3	6	82	88	3
393	2943	0	19	13	6	7	105	85	3
485	2498	0	16	7	9	6	90	231	3

806	6183	6	85	18	66	57	113	442	6
443	2603	1	15	10	5	6	100	111	3
407	2928	1	19	16	3	5	111	119	6
893	6675	4	68	29	39	62	138	506	15
1006	6483	2	66	26	40	66	123	634	9
403	2613	0	20	10	10	5	89	180	3
748	4105	4	38	18	20	12	141	155	6
425	2960	2	34	7	27	19	73	198	6
703	4873	1	32	13	19	29	159	423	4
662	2885	2	34	8	26	16	66	103	2
460	2880	0	25	11	14	11	84	226	3
622	2974	0	23	11	12	8	99	193	4
1037	4567	1	38	9	29	29	132	287	4
691	3605	2	24	14	10	11	133	145	5
618	4736	2	35	18	17	20	154	136	7
713	3905	3	30	16	14	10	141	134	5
601	3068	1	22	10	12	12	103	103	3
598	3393	2	25	14	11	10	116	180	6
471	2692	1	20	9	11	9	85	124	3
829	4116	1	32	12	20	18	128	288	3
683	3480	0	32	10	22	16	97	239	6
691	3175	0	27	8	19	19	80	189	3
691	3098	0	29	8	20	15	86	207	2
700	3447	2	30	8	21	18	96	201	4
492	1504	0	18	2	16	7	31	217	1
673	3525	2	34	11	23	16	97	165	4
902	5382	1	56	16	40	49	109	525	6
597	2769	0	21	11	10	9	90	186	4
791	4119	5	50	12	37	16	119	149	8
653	4006	1	27	20	7	21	122	100	5
573	2579	1	26	10	16	8	73	61	4
698	3707	1	38	11	26	15	100	206	4
488	3691	0	31	16	15	20	99	167	3
827	3794	1	32	9	22	23	98	246	4
600	2572	0	19	10	9	6	88	51	4
620	2783	0	22	11	11	7	93	83	4
671	2967	0	30	8	22	15	76	294	2
1091	5700	3	59	21	38	21	173	566	11
748	3181	0	19	11	8	15	103	170	4
822	4371	4	45	14	31	31	103	172	8
620	2704	0	25	7	18	14	77	99	2
767	3596	1	39	11	28	18	93	412	5
735	4335	1	39	12	27	22	131	269	4
693	3899	0	23	10	13	18	131	137	2
702	2773	0	17	7	9	13	91	60	2
1277	5770	2	57	19	37	28	165	718	8
1012	5842	2	59	19	40	40	148	508	8

482	4804	1	41	24	17	43	105	245	5
745	3346	1	23	11	12	11	111	208	4
743	3396	2	38	8	30	18	80	327	3
680	2749	0	23	10	13	10	83	188	4
746	3139	1	26	8	18	11	97	137	3
546	1758	0	9	5	4	1	67	62	1
652	3035	1	17	11	6	6	118	90	4
669	3113	1	18	9	8	9	116	125	3
658	3112	1	19	11	8	8	117	95	4
529	2259	0	15	7	8	6	78	141	2
657	3005	2	36	6	30	15	68	196	4
472	3959	1	32	20	12	20	116	133	5
880	5684	2	63	13	50	43	131	363	7
571	3652	3	50	9	41	23	79	111	5
711	3279	2	24	10	14	14	105	131	5
593	2910	0	22	10	12	11	91	112	3
806	3980	1	29	12	17	17	123	290	4
802	4525	1	34	19	15	28	129	285	5
713	2996	0	24	10	14	9	97	120	4
989	5372	4	46	18	28	26	168	557	7
698	3299	0	25	13	12	17	100	257	3
752	3245	0	27	9	18	14	90	340	3
731	4803	1	32	20	12	27	153	84	7
568	2910	1	33	9	24	11	86	223	5
599	3190	1	29	9	20	14	99	217	4
657	4020	2	28	19	9	9	153	207	6
587	3278	3	41	12	29	20	79	179	7
555	3733	2	33	14	19	15	122	65	6
692	4170	2	34	22	12	12	142	123	9
759	3523	2	39	13	26	17	93	342	6
603	2599	1	22	7	15	12	78	62	3
653	3955	2	34	14	20	25	108	170	6
566	3305	1	24	14	10	10	111	124	5
700	3036	2	30	8	22	14	80	96	2
756	3942	0	31	11	20	27	95	260	2
541	2682	0	23	7	16	12	77	96	2
654	3321	0	33	11	22	16	93	338	4
708	2858	0	19	10	9	5	102	126	3
619	3363	1	29	14	15	13	97	317	7
431	2252	1	24	6	18	12	57	235	3
701	2957	0	25	11	14	12	91	86	3
685	3881	2	33	18	15	12	126	250	7
593	4684	0	20	9	11	25	122	156	2
973	3765	0	14	4	10	26	78	155	1
238	2965	0	19	1	18	22	61	247	1
462	4624	0	35	2	31	37	69	415	2
572	3981	0	21	0	20	28	67	315	0

598	5387	0	34	11	22	36	122	412	3
535	4521	5	61	0	61	34	53	461	15
466	5654	4	54	3	49	45	91	421	13
485	4412	1	38	2	35	28	77	292	2
530	3514	0	15	4	10	16	82	148	2
557	3820	0	19	5	13	25	89	210	2
512	4249	1	27	4	22	23	98	390	3
348	3773	0	23	9	14	21	65	201	3
660	4767	0	26	1	25	29	94	395	3
556	4187	2	32	3	29	27	73	301	1
535	3511	0	26	0	25	14	72	261	4
661	4589	0	19	5	14	25	96	394	1
625	3122	0	17	2	14	18	64	156	1
1001	5147	2	42	3	38	37	87	305	8
541	5044	0	31	8	22	30	98	277	3
733	5740	1	36	9	27	44	116	603	3
423	2706	0	17	1	16	35	53	145	2
771	4866	0	48	5	43	28	103	397	4
356	3782	0	35	3	31	27	49	302	3
625	5431	0	41	4	37	44	92	364	3
539	3578	0	17	4	11	17	90	208	0
307	2138	0	12	2	10	10	40	141	1
379	3124	0	13	1	12	22	55	200	1
263	1708	0	7	2	5	28	35	55	2
465	3883	2	32	2	30	25	58	210	7
1020	5970	1	55	6	49	41	101	610	9
893	4776	1	37	5	32	23	109	288	6
662	5071	0	34	7	26	38	87	439	3
439	3888	0	23	9	14	18	95	165	2
698	4482	2	30	2	28	28	92	412	11
825	3594	1	26	3	22	16	81	227	6
528	4183	0	25	8	17	24	91	252	2
549	4192	1	26	7	19	12	117	200	5
248	2067	0	6	1	5	6	29	154	0
394	4072	1	26	3	23	37	62	192	0
845	4206	2	44	2	42	27	72	379	7
384	2375	0	11	3	8	8	49	113	0
700	3107	0	23	4	19	29	44	205	1
730	4107	1	22	4	18	16	103	302	1
282	2715	0	15	1	14	19	35	116	3
302	1941	0	11	2	9	16	42	65	1
465	3316	0	17	3	14	18	72	172	0
365	2266	0	8	2	6	18	46	107	0
161	2479	0	23	3	19	12	48	81	2
402	2489	0	14	3	11	12	46	111	3
354	2125	0	10	2	8	17	43	137	1
1097	3920	2	35	2	32	19	83	297	2

398	3053	0	15	3	12	13	69	147	3
454	4229	0	26	1	25	25	87	418	1
550	4864	4	52	3	48	37	73	334	11
470	3794	0	26	7	36	26	64	319	3
669	5618	2	60	6	53	36	103	520	10
578	4136	0	24	7	16	33	79	465	1
271	1903	0	11	2	9	16	36	53	2
964	4469	0	23	4	19	34	87	359	0
527	4072	1	25	8	17	17	103	210	7
383	3926	1	31	1	30	42	51	243	5
874	4913	0	37	6	30	29	123	290	2
862	3149	0	19	1	18	23	58	229	0
548	3873	0	22	4	18	16	88	188	4
696	5480	1	32	9	23	39	139	337	2
630	3838	0	15	3	12	18	94	245	2
364	2864	0	19	3	16	13	63	180	2
326	2597	0	10	2	8	10	64	153	1
290	2440	0	9	2	7	11	56	88	2
298	2475	0	17	2	62	15	46	186	4
887	3709	0	27	0	26	24	64	362	4
486	2497	0	15	3	11	15	51	229	1
1022	5729	1	49	9	40	41	129	506	1
506	3417	0	16	2	14	22	68	234	1
422	5031	1	38	19	18	17	124	172	7
443	3062	0	18	2	16	15	65	162	2
349	2718	0	16	3	13	13	52	82	2
385	3269	0	20	1	19	18	53	176	3
1021	3765	0	25	5	20	17	87	355	3
353	2340	0	10	2	8	11	47	97	1
480	3705	0	18	2	16	24	77	180	1
183	1428	0	4	2	2	5	34	64	0
305	2877	2	34	4	30	13	52	134	10
511	3795	3	37	5	32	16	93	179	12
257	2379	0	23	0	23	12	23	151	2
212	2018	0	13	1	12	13	22	133	3
406	4828	0	30	6	23	21	108	283	4
677	3845	0	29	11	17	20	86	362	8
245	1606	0	3	2	1	28	37	48	1
808	2928	0	12	4	8	12	49	172	2
655	2498	0	16	3	13	10	43	149	4
512	4249	1	27	4	22	23	98	390	3
436	3970	0	27	2	24	21	74	327	3
379	3213	0	20	2	18	28	51	218	0
538	3894	2	34	2	32	25	72	281	6
204	1253	0	12	2	10	26	29	69	2
510	3551	1	28	3	25	20	79	240	4
683	4155	1	31	7	23	18	104	260	5

324	2410	0	13	3	10	25	49	66	1
290	2071	0	8	2	6	18	48	102	2
864	3644	0	21	2	19	17	82	248	2
508	4453	0	33	9	23	30	98	326	3
361	3794	0	25	5	18	27	79	158	2
1062	4964	0	32	5	26	46	100	315	2
604	4923	2	42	1	39	42	65	252	4
406	3734	0	28	2	26	21	60	193	5
1232	4528	0	25	4	21	20	116	330	4
516	6056	5	60	4	56	64	83	186	6
1219	5232	3	48	4	42	36	101	346	12
149	1930	0	9	2	7	27	38	87	1
831	3852	1	33	3	30	24	73	274	8
1365	4862	2	34	4	29	32	119	399	3
356	3879	1	34	3	31	28	63	266	8
806	4904	3	53	4	49	31	87	443	15
422	4421	1	27	2	23	33	103	557	1
545	4776	0	38	5	33	26	107	380	1
272	1810	0	8	2	56	28	33	111	2
442	4202	0	30	5	63	23	79	485	4
209	1340	0	3	2	1	28	28	45	1
318	2291	0	15	4	11	11	41	187	1
1264	5195	1	37	4	32	34	115	694	2
859	2994	0	14	4	10	16	55	243	1
699	5544	1	37	3	33	35	101	433	2
309	2346	0	13	3	10	28	49	94	1
211	1720	0	4	2	2	25	35	61	0
688	5963	2	70	7	62	42	99	537	4
322	3326	1	25	3	21	21	55	164	0
502	3913	1	31	0	30	34	54	255	0
544	4825	0	32	0	29	30	84	448	3
440	3288	0	15	2	12	16	50	134	2
322	3045	0	21	3	17	16	41	179	3
1232	6278	0	36	9	26	47	135	302	3
895	3743	0	22	3	18	14	84	248	3
208	1263	0	2	2	0	18	31	40	1
611	4904	0	46	5	38	34	89	388	2
184	1426	0	5	4	1	19	43	39	1
466	5025	1	44	8	36	33	88	328	5
334	2795	0	17	2	15	18	40	194	0
408	2657	0	15	4	11	13	40	230	1
86	1741	0	5	2	3	16	36	79	0
666	6568	2	48	8	39	32	162	437	7
246	2813	0	15	0	15	13	52	301	0
693	5110	0	23	14	8	34	128	237	1
365	5397	1	52	2	49	50	74	399	4
932	5395	0	30	7	22	37	126	423	2

737	4130	0	23	3	18	16	115	301	2
645	4375	0	24	4	20	22	110	369	2
1144	4289	0	19	4	14	22	111	353	3
378	3179	0	10	2	8	14	87	220	2
458	4761	0	39	5	32	25	103	378	4
930	4941	0	27	4	22	32	110	365	1
241	1101	0	2	0	2	18	22	90	0
531	6175	1	65	5	58	47	84	510	7
198	3368	0	23	4	17	12	65	154	4
454	3271	0	17	1	15	15	75	211	2
293	5530	4	52	10	42	30	124	234	24
521	3058	0	16	2	14	13	63	169	2
395	5185	1	37	8	29	31	98	460	7
1158	5118	0	26	7	18	32	113	255	2
502	3404	0	23	6	15	29	55	137	2
580	5320	1	45	4	40	42	105	391	2
638	4002	0	25	2	22	19	98	225	2
747	5105	1	39	8	31	32	105	258	4
371	2499	1	23	3	20	15	42	422	2
492	3845	0	29	3	25	26	82	402	2
505	3727	0	21	5	15	19	80	160	0
568	5130	0	36	4	31	41	94	343	2
386	3040	0	18	2	16	14	60	103	3
772	5805	2	52	12	40	44	120	331	2
351	2570	0	10	2	8	11	53	163	2
385	3718	0	28	2	26	26	58	218	3
259	1767	0	10	2	7	3	30	57	0
1126	5119	1	35	5	29	43	98	297	3
540	5037	0	33	1	31	34	94	551	1
318	2262	0	8	2	6	19	44	84	1
504	4029	0	21	6	13	24	78	280	3
399	2909	0	18	2	15	10	59	136	3
587	5745	1	67	6	61	56	73	755	6
599	3323	0	8	4	4	14	93	135	1
415	3925	0	38	4	32	28	52	277	1
915	5521	0	37	7	30	36	108	540	7
391	2853	0	18	3	15	12	54	162	2
375	2867	0	13	3	10	10	52	135	3
507	4148	0	25	6	18	24	99	273	2
553	5068	0	39	5	33	36	79	358	4
582	5806	0	43	10	33	41	110	253	5
610	3078	0	15	2	13	16	79	109	1
737	2648	0	15	5	10	18	74	170	2
577	3374	0	11	0	11	17	73	269	0
939	4950	0	27	16	11	28	138	305	4
532	6213	3	65	1	62	68	80	399	4
294	2227	0	11	2	8	16	44	74	2

706	5443	0	26	12	13	26	168	314	3
480	6096	0	35	8	26	43	128	487	4
304	2756	2	24	1	23	18	42	162	5
1166	5179	0	27	9	18	34	125	335	5
381	3504	0	25	5	20	24	68	201	3
215	1430	0	6	2	4	36	32	39	1
219	1388	0	7	2	5	28	32	62	1
467	4784	0	33	9	24	35	85	254	4
864	5333	1	39	8	31	38	121	258	3
286	2642	0	15	3	12	13	37	143	3
549	3882	0	20	5	14	17	98	286	1
311	2860	0	15	3	12	14	42	150	1
369	2626	0	16	2	14	29	53	158	1
860	3481	1	25	3	22	37	48	172	0
459	4661	0	32	7	25	32	82	205	4
720	5320	1	42	5	37	38	125	708	4
510	3475	0	11	8	3	18	75	156	0
1394	6185	1	32	9	22	56	129	260	1
380	4064	0	32	6	26	31	57	277	3
293	3550	0	26	2	24	21	44	170	2
1112	4017	1	19	5	14	25	91	395	1
351	3974	0	37	4	33	24	69	343	3
940	3390	0	17	7	9	10	115	244	3
794	5084	0	31	8	23	35	102	339	3
717	3933	0	25	4	19	19	104	217	3
895	3583	0	21	3	18	27	78	187	1
334	2094	0	9	2	7	17	46	104	1
531	4430	0	31	6	25	28	66	285	4
169	2423	1	23	2	21	15	31	112	5
725	3581	0	17	3	14	25	82	165	1
158	1304	0	2	2	0	27	33	31	1
299	2321	0	8	1	65	12	38	95	6
479	3240	0	19	2	17	14	63	336	2
498	3488	2	16	12	4	27	129	73	4
229	1171	0	3	1	2	28	23	40	0
965	4029	0	27	7	19	22	82	294	1
757	5551	2	46	7	39	34	103	316	8
1118	4636	0	30	3	26	28	90	185	4
265	2014	0	9	3	6	29	44	85	1
972	5170	2	41	7	33	41	106	409	3
459	4465	0	31	3	28	28	77	209	4
251	2398	0	12	2	10	16	46	233	4
391	3314	0	32	0	31	22	36	220	5
546	4449	0	29	4	25	19	83	303	1
223	2280	0	15	1	66	13	28	182	4
966	5431	1	42	11	29	43	117	416	0

Mg (mg)	P (mg)	K (mg)	Na (mg)	Cl (mg)	Zn (mg)	Cu (mg)	Cr (mcg)	Se (mcg)	Mn (mcg)
156	585	1071	377	289	3	2	0	20	14
169	655	1143	499	308	3	2	0	15	16
130	368	896	776	113	4	2	0	13	12
169	630	1146	250	443	3	2	0	22	12
147	529	987	508	282	4	2	0	14	10
117	403	945	634	387	3	2	0	8	12
93	298	625	300	76	0	1	0	5	19
125	421	815	431	347	3	2	0	12	8
206	569	1292	1016	265	8	3	0	4	3
122	431	783	506	242	4	2	0	4	5
215	794	1680	651	484	3	2	0	20	15
199	558	1507	449	435	2	1	0	35	8
239	735	1426	908	613	6	3	0	26	18
111	412	836	406	246	3	2	0	17	8
118	428	1083	685	373	4	2	0	7	7
133	386	1041	351	304	2	1	0	12	8
136	441	1095	315	189	2	1	0	15	8
271	906	1771	994	166	8	3	0	14	4
178	580	1332	847	673	5	2	0	31	15
131	418	880	373	226	3	2	0	16	10
182	468	1250	461	256	3	2	0	36	4
173	578	1223	857	413	4	2	0	10	11
173	665	1393	977	792	6	3	0	22	19
255	823	2013	1004	925	5	2	0	42	22
284	845	1745	1260	961	7	3	0	56	31
217	694	1773	1031	876	3	2	0	33	11
74	213	507	300	159	1	1	0	8	7
255	775	1482	757	227	6	3	0	20	4
107	467	849	456	262	2	1	0	24	27
233	698	1505	1335	395	5	2	0	20	15
220	482	1517	393	461	4	2	0	55	3
180	512	1066	658	594	4	2	0	11	12
193	450	1237	1135	580	5	2	0	27	20
221	477	1292	667	316	5	2	0	29	11
101	338	592	306	81	2	1	0	11	7
66	210	507	192	107	0	1	0	7	5
176	617	1525	569	612	3	2	0	24	18
217	616	1076	345	209	4	2	0	17	16
107	391	600	558	460	3	2	0	10	12
152	507	1022	771	602	4	2	0	21	20
213	480	997	638	422	6	3	0	10	12
207	608	1398	699	255	4	2	0	25	18
206	665	1407	719	486	3	2	0	24	11
151	376	1007	440	237	2	1	0	23	8
209	695	1413	511	301	4	2	0	25	15
145	515	1143	743	411	3	2	0	33	11

171	476	882	356	226	3	2	0	4	2
213	621	1063	621	190	6	3	0	16	16
250	661	1487	896	383	8	3	0	15	4
266	881	2336	1626	449	7	3	0	36	13
280	821	2057	461	506	4	2	0	30	19
165	490	1162	876	610	4	2	0	13	12
101	554	1044	735	276	1	1	0	13	12
185	617	1086	1128	541	6	3	0	8	15
184	683	1461	1336	1121	4	2	0	14	22
177	938	1853	982	1152	5	2	0	47	22
108	343	918	485	361	2	1	0	18	13
153	509	1136	695	484	4	2	0	19	18
232	609	1432	681	221	6	3	0	16	11
210	680	1266	796	155	10	3	0	14	6
145	394	931	392	121	3	2	0	21	7
174	858	1539	560	581	4	2	0	27	34
206	1068	1814	1041	396	5	2	0	19	25
149	510	998	463	271	3	2	0	24	15
160	542	1059	515	262	2	1	0	16	23
165	588	1027	1087	427	6	3	0	5	12
221	752	1251	1077	344	7	3	0	10	15
243	678	1519	1151	159	8	3	0	19	5
280	814	1963	1372	567	8	3	0	30	14
69	245	664	460	188	1	1	0	7	7
58	216	571	303	185	1	1	0	7	7
166	515	1260	563	598	3	2	0	11	15
159	392	926	590	267	5	2	0	10	7
122	314	803	363	205	2	1	0	10	4
168	500	903	720	377	4	2	0	8	6
196	618	1250	610	65	6	3	0	7	1
132	479	805	314	222	4	2	0	10	7
125	462	765	307	312	4	2	0	10	12
193	630	1055	659	110	5	2	0	13	14
232	782	1951	533	432	3	2	0	32	22
149	482	1017	648	233	5	2	0	12	10
248	724	1530	849	146	6	3	0	11	9
151	494	1016	520	418	2	1	0	5	8
145	588	1092	527	342	4	2	0	19	14
207	640	1266	835	480	4	2	0	29	22
233	802	1535	737	406	7	3	0	19	9
105	288	620	299	111	1	1	0	4	4
139	433	858	491	374	2	1	0	10	9
160	410	1202	410	218	3	2	0	11	4
126	532	1003	618	624	2	1	0	17	26
100	445	702	598	609	1	1	0	12	21
111	448	789	596	368	1	1	0	6	11
293	632	1838	909	321	8	3	0	16	3

94	340	638	358	159	2	1	0	24	15
116	355	955	569	578	1	1	0	17	11
201	695	1944	1009	686	4	2	0	44	19
160	410	1202	410	218	3	2	0	11	4
221	798	1631	654	476	3	2	0	16	9
106	843	1374	2060	114	1	1	0	33	1
137	846	1328	2185	107	3	2	0	8	0
127	889	1339	1690	467	2	1	0	27	16
181	713	1261	897	475	5	2	0	20	18
219	650	1764	828	579	6	3	0	25	10
122	511	799	499	261	2	1	0	6	5
111	374	648	413	142	3	2	0	10	7
152	473	1063	602	677	3	2	0	16	5
137	432	788	1050	354	5	2	0	6	6
100	336	577	206	266	2	1	0	6	5
221	582	1141	770	496	4	2	0	19	5
194	1097	1719	1062	691	5	2	0	44	38
189	578	1444	407	419	3	2	0	40	20
113	384	942	506	363	2	1	0	25	8
141	434	887	618	238	4	2	0	7	1
185	674	1406	803	220	5	2	0	22	5
138	541	966	470	244	4	2	0	17	25
195	571	1425	1025	450	4	2	0	27	9
225	642	1575	764	476	4	2	0	21	25
206	665	1644	1033	529	4	2	0	20	12
202	825	1626	1258	404	6	3	0	32	22
168	478	1419	595	356	4	2	0	14	9
147	474	995	539	224	5	2	0	15	15
126	451	746	622	178	4	2	0	8	10
261	618	2197	1588	642	7	3	0	29	5
207	640	1372	875	429	6	3	0	37	30
276	821	1908	1326	480	9	3	0	21	6
323	845	2059	1153	465	8	3	0	37	9
155	537	1161	544	403	2	1	0	18	18
160	526	1053	563	283	5	2	0	11	7
162	474	1080	359	167	3	2	0	11	13
190	654	1399	972	298	4	2	0	12	15
134	464	870	518	437	4	2	0	17	14
122	440	695	332	192	2	1	0	15	15
175	492	1021	816	209	7	3	0	20	7
86	282	656	357	404	1	1	0	9	9
28	165	446	190	100	0	1	0	1	3
69	311	616	391	152	1	1	0	3	1
161	643	925	597	395	3	2	0	16	28
57	257	752	264	200	1	1	0	15	9
88	344	747	495	167	3	2	0	1	5
101	546	1095	1133	1261	2	1	0	9	26

34	250	420	253	79	0	1	0	0	4
111	676	956	527	456	2	1	0	21	30
89	408	722	363	302	1	1	0	18	25
138	747	1122	427	188	5	2	0	22	38
120	577	1003	793	158	3	2	0	8	8
109	411	894	971	579	2	1	0	12	8
166	561	1372	1030	678	1	1	0	23	19
170	598	1143	855	396	2	1	0	18	7
143	440	744	734	190	6	3	0	19	15
145	817	1369	1495	322	3	2	0	22	10
163	577	1336	757	918	2	1	0	37	20
115	364	778	304	277	2	1	0	4	4
133	431	909	478	286	3	2	0	8	6
143	701	1376	444	215	8	3	0	44	32
132	399	1020	486	255	3	2	0	28	14
117	414	1037	394	168	5	2	0	27	10
213	496	1310	717	334	5	2	0	30	7
144	391	1160	519	362	2	1	0	22	9
279	881	1792	1397	571	9	3	0	18	6
159	677	1152	514	304	4	2	0	16	10
204	899	1367	870	283	7	3	0	22	18
160	607	997	995	273	6	3	0	1	2
154	612	846	1051	434	5	2	0	25	33
113	686	1058	1361	532	3	2	0	5	15
118	536	1039	801	935	3	2	0	18	21
188	470	1215	660	375	4	2	0	13	12
196	456	1069	747	445	5	2	0	19	5
188	672	1092	762	142	5	2	0	6	4
152	354	900	656	116	6	3	0	8	0
269	964	1796	1512	597	10	3	0	16	13
162	637	868	761	176	4	2	0	6	18
129	482	811	408	335	3	2	0	6	12
162	453	743	593	171	6	3	0	4	4
320	939	1899	1013	566	8	3	0	12	8
108	413	700	641	195	3	2	0	9	6
118	357	657	448	144	4	2	0	7	10
97	234	432	366	48	2	1	0	1	0
50	194	425	437	200	2	1	0	5	7
120	457	736	417	215	3	2	0	8	10
169	477	880	413	187	3	2	0	10	5
129	358	535	124	115	3	2	0	6	6
97	534	594	790	564	4	2	0	42	48
205	577	1076	1208	176	9	3	0	5	8
123	318	735	396	128	3	2	0	11	6
102	240	625	580	168	3	2	0	4	1
120	331	589	623	218	4	2	0	6	4
122	349	739	231	315	2	1	0	5	2

250	1184	1869	1059	567	12	3	0	54	50
132	332	553	113	211	3	2	0	1	1
140	343	674	867	60	7	3	0	0	0
380	1130	2284	1074	620	9	3	0	33	22
358	1205	2336	1081	859	8	3	0	33	24
123	386	725	482	176	4	2	0	2	1
189	551	987	834	133	8	3	0	3	8
120	533	721	399	473	4	2	0	14	18
177	668	1080	559	386	3	2	0	1	4
118	397	878	483	152	4	2	0	15	14
123	419	854	693	155	3	2	0	7	7
132	438	814	638	203	4	2	0	9	7
156	611	1250	463	525	4	2	0	38	34
179	487	747	345	202	5	2	0	6	8
221	598	1061	808	377	6	3	0	12	12
192	539	882	487	141	6	3	0	9	11
145	398	666	297	223	4	2	0	14	7
141	449	808	798	208	6	3	0	6	7
116	376	646	466	188	2	1	0	6	7
171	484	1170	662	421	4	2	0	15	3
156	529	989	692	383	5	2	0	11	9
129	475	896	623	403	2	1	0	10	11
144	480	951	458	227	2	1	0	6	1
130	452	843	467	285	3	2	0	14	7
71	346	608	374	50	0	1	0	2	1
152	495	1053	606	267	5	2	0	12	14
242	969	1664	1074	785	6	3	0	20	24
126	406	714	592	196	4	2	0	6	8
168	623	1304	674	370	7	3	0	14	13
187	486	1010	855	294	7	3	0	13	9
112	349	705	580	154	4	2	0	4	1
160	572	1067	620	297	5	2	0	18	12
173	559	1005	627	162	5	2	0	12	10
135	522	1163	815	545	3	2	0	24	25
116	338	652	402	101	4	2	0	7	7
123	365	662	532	122	4	2	0	6	6
129	473	1028	568	339	3	2	0	6	3
268	1052	1855	1656	194	11	3	0	15	8
139	406	853	548	225	4	2	0	9	7
161	691	1115	1020	442	6	3	0	47	48
105	358	574	197	201	2	1	0	16	15
181	718	1258	814	354	6	3	0	18	10
196	695	1226	615	385	4	2	0	22	20
145	423	1085	542	399	2	1	0	9	6
100	286	610	175	91	2	1	0	9	6
314	979	2185	1058	544	7	3	0	35	9
241	956	1740	1687	746	10	3	0	24	23

237	708	1243	1156	544	7	3	0	6	7
154	488	908	527	238	3	2	0	8	8
135	510	1184	708	502	4	2	0	9	11
123	420	763	459	231	4	2	0	6	8
125	446	776	409	150	2	1	0	7	11
75	198	440	174	96	1	1	0	2	0
127	353	555	428	132	3	2	0	6	6
134	407	614	192	130	3	2	0	7	6
134	373	604	435	142	3	2	0	8	6
86	292	510	441	206	1	1	0	7	7
119	541	846	471	246	3	2	0	10	11
190	521	1066	947	232	7	3	0	4	5
177	882	1601	1152	549	6	3	0	24	20
124	512	1022	553	216	5	2	0	20	13
137	398	711	596	604	5	2	0	3	9
132	368	779	437	120	4	2	0	5	1
194	607	1277	547	249	4	2	0	17	10
239	683	1290	705	351	5	2	0	7	4
122	339	711	499	283	4	2	0	2	3
232	840	1529	1128	567	8	3	0	3	9
133	454	759	654	491	4	2	0	11	16
140	470	1002	747	488	3	2	0	6	8
193	579	877	754	94	6	3	0	13	15
135	556	916	454	97	4	2	0	11	7
133	522	881	388	57	3	2	0	6	0
196	512	1026	794	270	7	3	0	5	2
129	599	786	899	199	7	3	0	27	30
163	492	718	567	112	5	2	0	12	20
170	424	1007	1468	236	9	3	0	2	2
168	572	1274	891	388	6	3	0	14	3
98	324	584	345	185	2	1	0	8	6
133	534	925	1163	536	7	3	0	30	33
141	410	818	866	299	6	3	0	4	5
124	400	797	413	277	3	2	0	8	12
152	528	1274	936	539	3	2	0	16	10
107	359	536	310	96	2	1	0	9	10
149	615	890	779	210	4	2	0	15	16
118	356	649	456	100	4	2	0	1	1
191	471	1335	858	448	5	2	0	9	10
95	375	683	324	351	3	2	0	7	10
134	418	828	281	115	2	1	0	18	15
190	588	1098	994	339	7	3	0	3	3
126	520	1189	672	690	2	0	29	10	1018
108	459	1255	649	708	2	0	28	10	963
65	470	865	703	894	0	0	26	10	533
140	792	1602	1156	1156	1	0	29	19	1424
105	591	1457	1087	1024	0	0	33	19	1157

166	795	1621	821	570	2	0	31	16	2062
204	910	1849	1064	624	7	0	48	36	3445
150	1054	1907	2560	869	1	0	22	27	1876
122	730	1672	1369	1120	2	0	34	40	1247
91	414	1045	701	472	2	0	14	8	884
95	463	939	644	586	2	0	21	21	745
135	613	1314	617	689	3	0	27	25	1453
132	539	1350	656	427	1	0	26	18	1586
154	696	1877	944	599	0	0	45	18	2242
141	630	1604	794	643	1	0	30	17	2092
111	525	1242	877	475	1	0	18	10	1314
174	689	1743	864	789	1	0	27	17	1857
77	376	967	664	593	1	0	19	16	711
195	869	2153	1212	1040	3	1	51	35	3104
166	783	1809	1054	883	1	0	28	28	1590
157	966	1885	1285	1186	1	0	32	24	1157
66	367	804	675	242	1	0	14	7	1117
179	824	1809	1183	492	4	0	46	31	1099
124	676	1460	817	341	1	0	22	16	1341
154	957	1901	1361	1203	1	0	57	51	1524
93	450	1128	481	283	0	0	18	11	916
71	312	568	459	488	1	0	6	10	507
71	493	1050	676	358	0	0	9	7	728
57	219	438	190	123	1	0	5	4	589
94	706	1390	1501	395	1	0	18	13	1449
261	1082	2391	1734	1084	3	0	60	32	3411
154	663	1352	1140	726	3	0	22	18	1594
222	837	1986	1229	1099	3	0	36	28	2750
137	533	1066	751	826	2	0	26	21	1034
188	771	1903	1394	428	2	0	42	13	3263
130	536	1293	572	376	3	0	28	15	1465
142	592	1344	501	305	2	0	28	12	1776
135	536	1122	491	366	3	0	18	7	1244
58	284	770	515	392	0	0	16	8	1340
100	481	1157	703	812	0	0	17	19	1020
115	853	1566	1271	1232	3	1	33	34	1410
72	280	704	331	239	0	0	12	5	721
84	464	907	718	585	0	0	16	15	867
133	554	1407	660	435	2	0	22	12	1104
45	346	758	1010	984	3	0	8	13	594
60	287	551	312	130	0	0	16	11	617
71	405	975	456	421	0	0	26	10	705
69	256	739	299	237	0	0	23	3	768
52	388	516	766	467	0	0	2	24	519
83	336	869	373	307	1	0	26	10	951
74	327	760	327	302	1	0	23	10	608
95	588	1251	745	443	1	0	33	21	921

103	415	980	835	614	1	0	29	13	1014
118	738	1577	885	823	0	0	42	31	1461
102	909	1613	2469	832	0	0	32	28	1188
135	647	1693	1022	547	3	0	31	17	1643
184	975	1800	1397	1155	5	0	41	29	2117
130	797	1607	559	488	1	0	35	17	1361
57	248	480	384	293	1	0	10	9	533
115	650	1570	1029	1069	0	0	33	19	944
154	559	1262	790	353	3	0	18	2	1886
119	606	1406	1164	945	3	0	42	18	1461
120	676	1113	927	703	2	0	19	24	884
78	444	1016	714	1134	0	0	33	16	563
133	523	1231	804	607	2	0	33	17	1381
128	716	1394	768	732	2	0	28	24	773
121	564	1306	650	519	1	0	29	17	976
68	355	817	630	238	0	0	16	11	881
86	351	839	410	223	1	0	17	4	803
59	339	632	305	183	1	0	8	8	544
69	374	741	483	581	3	0	18	18	852
105	602	1412	981	1192	3	0	35	33	890
88	370	990	313	226	0	0	27	22	1701
147	747	1634	1082	1158	2	0	37	31	1382
93	549	1300	552	411	0	0	26	17	868
177	591	1365	1498	699	7	0	21	21	1524
83	450	917	428	372	1	0	21	18	740
63	303	572	514	433	1	0	4	9	697
92	486	1067	813	615	1	0	33	18	1234
136	558	1313	828	587	2	0	19	14	1233
65	302	632	385	292	1	0	12	11	569
106	501	1205	742	566	1	0	28	11	951
45	154	396	176	156	0	0	17	3	500
121	491	811	634	191	4	0	6	15	1813
154	598	1210	942	306	5	0	6	20	2485
63	416	825	633	397	1	0	15	15	805
55	347	708	521	263	2	0	11	11	501
131	636	1343	1237	607	3	0	12	15	1520
233	669	2093	431	238	1	0	32	5	3182
51	187	446	305	131	1	0	9	1	519
113	372	1051	627	460	1	0	33	10	1457
96	361	701	528	341	2	0	20	10	1260
135	613	1314	617	689	3	0	27	25	1453
133	648	1417	1060	986	1	0	31	21	1417
85	509	897	802	757	0	0	20	23	735
96	692	1326	967	347	1	1	32	33	1086
53	232	344	180	71	1	0	6	8	555
125	535	1174	510	490	2	0	46	16	1428
161	667	1368	486	364	3	1	23	31	1970

64	303	497	385	336	1	0	10	11	605
61	261	457	495	275	1	0	10	5	508
115	574	1287	491	325	1	0	28	12	951
152	689	1082	789	552	2	0	17	16	1073
83	480	963	1110	914	3	0	20	24	607
102	709	1354	1189	1313	1	0	15	28	712
150	727	1904	1342	1044	2	0	52	29	1870
137	651	1358	890	646	2	0	32	33	1754
162	640	1579	602	307	2	0	29	13	2140
108	1104	1870	1267	1468	7	4	41	79	1236
204	842	1899	1364	564	6	0	40	24	2658
49	240	429	281	250	1	0	11	3	392
124	695	1420	902	595	3	0	34	24	1730
151	642	1476	984	809	1	0	35	30	1611
122	679	1422	1030	671	3	0	30	23	1608
230	946	1986	946	406	6	0	48	28	3574
108	811	1447	642	886	1	0	46	22	755
154	770	1488	594	402	1	0	37	24	1359
69	267	669	385	294	1	0	6	4	604
132	755	1446	967	1037	3	0	31	38	1297
38	152	315	237	185	1	0	3	2	282
68	399	835	469	384	0	0	11	7	771
156	876	1852	1286	1516	1	0	37	28	959
106	409	1044	695	714	1	0	24	12	895
163	801	1813	1461	1537	2	0	32	30	1810
65	289	559	443	384	1	0	16	9	490
38	190	453	412	276	0	0	1	4	517
186	1108	2048	1811	1379	4	0	63	50	977
62	386	816	687	575	0	0	16	12	532
76	544	1152	810	804	1	0	35	16	657
156	764	1875	896	916	1	0	42	21	1781
93	434	987	822	734	1	0	25	15	1033
103	476	1176	1061	448	1	0	18	10	1363
143	729	1513	1280	992	2	0	22	27	1670
126	506	1292	422	334	2	0	29	15	1561
41	136	286	160	118	1	0	0	0	280
134	852	1598	1366	1089	1	0	41	39	880
28	113	240	135	129	1	0	9	0	229
183	901	1750	1347	657	3	0	41	31	1922
99	396	1033	636	506	0	0	16	7	1159
112	401	1052	506	426	1	0	15	5	1309
39	204	401	410	425	0	0	1	1	302
238	1030	1995	1471	981	4	0	37	37	2604
65	475	921	554	554	0	0	20	13	526
168	622	1692	602	372	1	0	18	6	2061
147	991	1761	1655	1521	3	1	30	55	1206
186	727	1695	896	1070	1	0	49	31	2024

124	536	1258	589	362	0	0	31	13	1213
138	647	1511	469	693	0	0	59	17	1359
141	582	1413	757	643	2	0	21	23	1225
87	384	902	605	760	1	0	14	11	740
170	780	1614	1041	655	3	0	29	29	1792
136	712	1581	968	1030	0	0	23	24	1468
30	138	367	153	164	0	0	6	2	380
197	1099	1987	1858	1337	3	0	29	54	2157
101	506	838	1087	713	2	0	5	18	1124
100	477	1061	650	400	1	0	15	8	1179
103	835	1439	3660	614	3	0	21	16	1354
85	424	1048	541	413	1	0	21	10	1221
202	877	1660	1236	1020	4	0	30	26	2165
155	761	1665	822	605	2	0	31	22	1476
66	356	724	1258	1097	3	0	7	17	682
143	836	1450	1369	1440	1	0	43	36	966
128	555	1121	600	426	2	0	24	23	1150
176	852	1630	804	662	4	1	51	44	1912
85	549	946	713	735	2	0	5	19	609
122	742	1326	546	441	0	0	15	28	1165
94	484	1017	810	816	0	0	34	23	1104
170	752	1829	1194	1387	1	0	37	33	2104
85	413	794	468	434	1	0	15	13	861
156	775	1493	1046	681	3	0	23	26	1340
75	378	797	395	300	1	0	21	6	733
91	634	1057	1385	1366	0	0	23	32	878
58	229	553	225	143	0	0	16	4	579
148	715	1526	1113	1163	4	0	55	31	1427
124	856	1757	1233	1353	0	0	41	39	1332
67	276	602	433	370	1	0	17	8	809
156	538	1553	1228	984	2	0	49	21	2097
111	397	920	467	198	1	0	22	20	1396
190	1382	2374	1782	760	3	0	46	17	1276
82	345	901	537	452	1	0	17	4	649
107	696	1329	1217	803	0	0	26	18	946
239	954	2209	1231	679	3	0	54	26	3576
92	448	930	522	298	1	0	8	9	969
100	399	984	704	478	2	0	25	9	1268
116	511	1178	1117	899	1	0	26	22	1073
167	820	1773	972	891	2	0	38	30	2152
186	961	1888	1347	871	3	0	32	34	2205
85	380	775	570	487	1	0	13	13	893
114	335	923	454	190	1	0	20	7	1122
100	405	1370	548	764	0	0	31	14	1073
175	705	1593	1105	547	4	0	15	8	1434
163	966	1918	1734	1948	4	1	42	54	1801
65	327	552	451	348	1	0	5	10	574

176	659	1321	554	662	3	0	22	15	1329
144	832	1620	1232	1094	1	0	32	28	1802
79	444	766	677	435	1	0	23	27	1058
191	768	1770	732	512	2	0	41	16	1766
121	555	1014	583	353	2	0	11	11	1197
41	152	283	136	98	1	0	3	2	314
41	167	347	133	135	1	0	10	3	291
175	722	1551	716	547	3	0	32	19	1900
128	756	1658	1106	911	2	0	22	28	1167
101	386	821	912	741	2	0	7	9	1171
125	536	1183	790	623	1	0	35	12	1005
86	407	968	805	468	0	0	26	6	1388
77	413	728	569	484	1	0	11	11	629
67	493	1137	694	687	0	0	11	10	712
178	673	1543	987	787	3	0	34	18	2100
134	870	1457	777	1156	1	0	29	25	1171
138	433	1283	633	482	1	0	29	7	1556
191	810	2232	1225	1172	2	0	41	25	1951
142	683	1403	924	791	2	0	38	24	1739
101	563	1170	1359	872	1	0	19	20	1370
130	622	1420	719	831	1	0	36	16	1104
101	671	1031	941	772	2	0	13	24	943
116	430	888	657	482	3	0	15	6	915
184	826	1896	962	653	2	0	31	15	1946
124	527	1417	643	211	1	0	27	13	1435
90	538	1252	550	397	2	0	36	9	661
66	265	683	235	243	1	0	22	3	541
161	737	1735	1153	581	3	0	36	19	1960
64	358	713	590	364	3	0	9	10	1023
88	501	1206	564	326	0	0	30	10	860
36	154	282	109	90	1	0	1	1	250
59	289	794	578	488	5	0	31	11	702
125	563	1297	516	322	1	0	28	14	1337
157	418	599	157	70	4	0	9	4	707
30	144	323	210	54	0	0	5	2	320
125	633	1352	805	727	0	0	34	26	1269
184	961	1955	2038	600	2	0	38	25	2164
143	680	1729	1276	1095	1	0	51	29	1584
61	262	611	325	381	1	0	24	4	642
141	819	1620	786	619	3	0	54	40	1064
135	724	1449	970	768	2	0	28	25	1471
75	346	839	426	108	3	0	12	2	1572
62	502	882	991	795	4	0	14	17	838
149	659	1676	875	811	0	0	40	24	1808
72	367	788	477	502	4	0	18	12	841
152	843	1429	1357	898	1	0	30	25	1627

I (mcg)	B (mcg)	Vitamin A	Total carot	B-Carotene	A-Carotene	Cryptoxan	Thiamin (n	Riboflavin	Niacin (mg
1279	24	349	42	3205	2701	992	9	0	1
1589	24	347	45	3077	2599	942	8	0	1
956	21	501	25	917	866	94	6	0	1
918	23	451	147	535	448	33	4	0	1
807	16	448	22	763	681	149	9	0	1
734	17	747	35	456	404	97	7	0	1
1373	5	246	6	710	598	126	94	0	1
798	9	753	28	312	252	77	34	0	1
1894	1	267	25	1357	1246	192	21	0	1
856	7	382	25	287	210	89	4	0	1
1228	17	538	125	3181	3021	155	39	0	1
1482	15	580	49	1137	1063	43	100	0	1
2098	19	585	35	1202	1165	57	12	0	1
735	12	217	78	461	381	71	10	0	1
1020	10	400	83	249	133	38	10	0	1
968	12	608	30	1242	1047	323	15	0	1
919	14	669	16	1278	1121	299	15	0	1
2978	6	591	15	1036	1026	7	11	0	2
1284	17	781	36	1180	1132	76	11	0	1
943	13	497	31	1243	1169	138	4	0	1
1399	8	726	23	1084	1061	29	11	0	1
1470	11	1112	31	453	434	18	15	0	1
793	19	489	64	533	497	54	14	0	1
2632	30	670	95	1355	1242	209	9	0	2
2890	36	725	49	1299	1199	188	9	0	1
1851	7	987	65	2223	2098	225	17	0	1
510	13	421	30	196	62	1	215	0	1
1382	5	877	30	884	781	169	15	0	1
637	43	471	93	425	365	3	13	0	1
1431	26	562	44	2253	1948	588	2	0	1
1749	8	1085	24	2733	2606	233	11	0	1
1365	13	379	50	912	867	81	3	0	1
1732	20	1263	16	2625	2496	72	178	0	1
2024	23	1052	22	6444	6312	159	45	0	1
573	11	243	15	143	138	2	2	0	1
597	6	257	8	969	874	186	0	0	1
1068	32	656	98	3635	3054	1158	0	0	1
1556	28	693	43	4310	3572	1455	5	0	1
764	17	152	29	114	111	5	2	0	1
676	28	663	79	64	52	15	2	0	1
1123	13	666	24	1516	1501	11	16	0	1
2073	33	1182	45	1673	1542	220	12	0	1
1693	15	939	95	1235	1118	32	97	0	1
1267	18	746	21	1230	1184	63	7	0	1
1561	31	1024	46	604	439	75	204	0	1
1197	19	933	124	825	737	41	1	0	1

728	2	1001	49	545	472	86	15	0	1
1030	26	744	40	2549	2175	725	12	0	1
2806	2	1858	22	5378	4544	1658	7	0	2
2329	24	2454	34	6656	6524	177	55	0	1
1956	29	1926	56	6525	5629	1770	17	0	1
1299	19	364	54	1494	1451	53	9	0	1
722	15	320	56	2208	1816	665	4	0	1
1130	15	149	70	241	183	16	3	0	1
1219	27	526	19	618	586	59	3	0	1
1182	35	978	344	1071	858	29	13	0	2
900	21	413	24	2729	2287	874	6	0	1
1067	27	861	61	336	302	40	9	0	1
1863	21	1192	28	558	462	130	24	0	1
2712	7	569	6	986	952	58	5	0	2
1128	14	486	13	537	504	46	14	0	1
824	46	614	148	545	404	113	15	0	1
870	36	677	101	681	530	193	13	0	1
1111	26	696	75	927	828	32	4	0	1
1079	28	619	42	1565	1552	6	16	0	1
850	10	185	22	373	365	4	1	0	1
1835	21	570	26	708	609	187	6	0	1
2748	7	1460	33	1751	1648	80	15	0	2
3138	19	1024	56	2084	1998	149	14	0	2
829	12	381	21	1046	876	178	154	0	1
831	12	586	21	793	698	180	2	0	1
917	19	560	65	178	154	28	14	0	1
1032	14	485	23	1010	940	142	0	0	1
782	5	1388	25	330	276	90	11	0	1
1518	6	155	19	429	372	17	92	0	1
1548	1	703	4	668	610	106	7	0	1
853	11	295	38	383	372	4	3	0	1
775	14	262	38	442	431	4	3	0	1
1290	23	263	29	120	100	30	3	0	1
1929	34	1404	59	4378	3913	913	14	0	1
846	13	577	39	5330	4427	1780	19	0	1
2026	9	434	39	719	540	152	103	0	1
755	2	217	60	1374	1273	84	13	0	1
599	21	411	83	521	446	30	14	0	1
1683	35	608	43	818	771	76	9	0	1
2413	16	440	60	2093	1772	630	8	0	1
841	6	549	11	965	872	181	0	0	1
1059	11	272	36	527	505	40	4	0	1
1274	7	1339	26	1635	1381	430	20	0	1
822	31	609	85	454	444	13	4	0	1
552	27	176	56	170	164	8	0	0	1
566	11	219	37	207	194	14	6	0	1
2881	5	2451	17	3251	3186	49	81	0	1

661	30	308	38	313	249	114	11	0	1
1030	13	647	25	287	264	26	14	0	1
2270	31	1677	45	5542	4781	1132	370	0	1
1274	7	1339	26	1635	1381	430	20	0	1
1198	15	714	60	1986	1662	635	4	0	1
1129	4	207	14	1828	1482	448	189	0	2
889	0	962	0	988	813	344	3	0	2
1090	30	561	49	2331	2067	492	21	0	2
1781	29	533	45	2006	1750	472	26	0	1
1444	20	1246	29	2087	1795	556	20	0	1
467	7	173	18	147	132	24	3	0	1
645	13	511	37	532	440	158	3	0	1
1226	5	958	22	1434	1323	210	5	0	1
613	7	379	32	152	118	34	1	0	1
683	7	164	22	160	132	24	13	0	1
1995	3	823	29	252	180	43	0	0	1
794	59	605	109	6400	5294	2201	5	0	1
1441	32	1553	44	766	722	43	32	0	1
984	13	337	56	5828	4837	1895	1	0	1
789	2	558	35	803	677	223	3	0	1
991	3	471	71	1089	920	96	11	0	1
959	26	284	31	1479	1253	445	4	0	1
1224	10	553	48	968	939	45	9	0	1
2362	10	862	77	1176	1036	169	7	0	1
1193	16	1441	100	1238	1092	167	5	0	1
1529	39	768	47	673	645	29	21	0	1
1129	14	1514	54	1376	1207	207	26	0	1
1184	16	498	29	766	645	84	152	0	1
711	17	579	47	330	255	98	12	0	1
2673	9	2226	31	3571	3422	166	21	0	1
1467	52	948	62	1355	1304	91	4	0	1
2010	14	1164	98	2634	2175	789	5	0	1
3169	12	1779	46	3824	3489	649	11	0	1
937	27	536	44	548	491	96	1	0	1
1241	11	676	35	726	701	32	2	0	1
1193	5	318	5	8518	7509	1977	35	0	1
1521	21	577	30	5437	4460	1930	23	0	1
976	23	868	29	284	258	42	8	0	1
830	26	322	45	571	508	89	16	0	1
1391	17	519	30	996	934	104	10	0	1
663	12	679	35	819	557	27	440	0	1
271	2	467	3	311	227	80	84	0	1
459	4	125	49	96	72	37	10	0	1
960	30	122	1007	1331	1135	367	1	0	1
487	7	226	21	669	565	145	13	0	1
410	2	430	27	2305	1902	795	4	0	1
823	19	288	12	447	344	161	20	0	1

370	1	194	597	379	339	73	1	0	1
499	43	97	52	105	43	9	11	0	1
664	41	338	51	1088	891	364	7	0	1
873	19	558	4430	2448	2025	813	1	0	2
1216	13	129	16	2025	1779	423	16	0	1
870	7	243	20	223	176	9	12	0	1
1091	22	535	26	1528	1494	22	19	0	1
1231	7	303	53	357	315	17	9	0	1
893	27	91	49	69	35	13	3	0	1
1252	12	565	616	2163	1672	423	393	0	2
1345	24	1387	67	634	497	208	4	0	1
643	2	231	48	126	88	7	13	0	1
772	4	836	611	616	528	100	60	0	1
1260	31	618	314	2980	2450	911	99	0	1
1377	14	568	12	4647	4351	530	44	0	1
695	11	364	20	580	507	130	11	0	1
1895	5	601	1	2094	2040	73	21	0	1
1633	13	735	13	3867	3263	1191	8	0	1
2709	4	780	17	1781	1693	169	0	0	1
741	15	404	47	966	925	74	4	0	1
907	30	465	52	348	300	72	17	0	1
542	0	79	46	141	131	14	0	0	1
485	52	131	111	94	91	1	0	0	1
502	2	251	46	112	107	0	10	0	1
942	30	291	78	1319	1111	414	4	0	1
1302	4	471	11	3170	3137	24	38	0	1
1504	3	794	23	1639	1573	112	12	0	1
1353	10	341	16	172	142	45	12	0	1
790	3	311	8	409	394	18	11	0	1
2031	7	517	25	843	831	7	15	0	2
545	18	148	32	116	116	0	0	0	1
529	13	155	55	152	144	9	2	0	1
858	5	89	29	107	96	14	3	0	1
2435	4	738	82	1044	993	88	10	0	1
573	11	189	20	420	399	33	3	0	1
546	13	241	24	460	393	126	3	0	1
426	0	228	1	150	145	0	3	0	1
293	11	104	36	235	192	84	0	0	1
743	13	217	35	669	605	124	1	0	1
1164	4	112	26	1073	988	156	9	0	1
574	11	124	21	382	318	126	0	0	1
409	82	181	110	127	107	33	1	0	1
895	7	344	9	158	147	3	15	0	1
862	14	432	21	539	490	97	0	0	1
677	2	372	9	377	362	20	7	0	1
579	5	115	15	122	113	14	3	0	1
769	4	134	46	171	152	33	1	0	1

1465	69	625	99	5309	4373	1839	29	0	1
587	0	56	19	105	100	6	1	0	1
476	0	220	8	87	73	0	20	0	1
4099	34	1705	82	3027	3012	20	1	0	2
2842	31	1806	67	3578	3466	210	3	0	1
536	0	162	23	310	265	81	3	0	1
831	5	309	18	158	140	0	30	0	1
622	27	153	66	216	210	7	1	0	1
889	0	459	46	70	56	4	18	0	1
671	17	439	24	506	484	34	6	0	1
675	14	205	30	115	106	21	1	0	1
527	11	195	36	160	152	6	6	0	1
1059	61	707	112	462	407	73	31	0	1
684	12	158	37	96	90	9	1	0	1
1261	15	445	24	764	681	156	4	0	1
696	14	165	25	181	175	0	8	0	1
729	13	342	21	528	494	60	3	0	1
645	12	286	32	340	293	84	4	0	1
519	11	294	22	438	370	129	2	0	1
1401	11	681	28	1033	945	156	10	0	1
1202	14	305	41	612	585	43	3	0	1
700	14	359	22	472	394	145	4	0	1
755	2	383	9	824	742	155	4	0	1
925	13	286	43	156	152	1	7	0	1
292	0	263	2	55	53	1	0	0	1
841	19	527	28	818	766	97	5	0	1
1756	30	833	74	1553	1506	89	1	0	1
494	11	193	36	107	97	0	13	0	1
986	13	503	27	362	329	30	35	0	1
1322	20	211	25	259	230	50	1	0	1
853	0	181	0	202	196	7	4	0	1
983	18	450	23	785	740	77	12	0	1
1167	14	414	24	574	509	114	7	0	1
882	42	532	82	944	842	194	11	0	1
601	12	252	15	506	436	129	3	0	1
541	11	180	21	157	148	9	2	0	1
609	3	370	40	551	452	36	155	0	1
1518	18	650	25	676	640	53	13	0	1
737	12	551	36	433	387	81	4	0	1
1154	83	417	111	806	722	153	6	0	1
537	29	218	46	149	123	42	7	0	1
1104	18	609	43	1910	1745	323	1	0	1
1000	36	354	33	253	196	84	20	0	1
1221	11	803	1	226	172	70	28	0	1
664	11	390	15	99	88	6	15	0	1
2686	11	1071	69	2513	2452	99	20	0	1
1501	31	679	67	861	824	64	3	0	1

1922	3	520	18	660	648	13	3	0	1
688	12	360	37	381	334	78	6	0	1
888	6	482	53	576	550	39	14	0	1
490	12	264	37	452	384	128	1	0	1
620	13	286	24	463	394	126	5	0	1
431	0	120	8	371	307	126	1	0	1
632	11	170	22	399	331	129	2	0	1
668	11	143	21	431	364	129	1	0	1
646	12	192	22	486	408	150	1	0	1
448	11	118	36	121	114	9	1	0	1
880	17	354	38	696	626	129	3	0	1
1166	7	699	16	483	373	8	210	0	1
1213	28	870	35	484	424	88	24	0	1
608	19	520	28	445	399	63	24	0	1
605	3	220	19	162	155	9	3	0	1
752	1	457	10	781	686	181	2	0	1
1232	16	865	33	1049	993	99	10	0	1
1697	2	796	18	1502	1456	88	0	0	1
627	0	528	20	129	119	9	7	0	1
1079	2	459	75	178	162	15	15	0	1
656	22	159	74	174	172	2	0	0	1
705	10	252	58	386	374	11	11	0	1
1391	26	513	32	153	112	4	69	0	1
636	13	382	14	208	199	0	18	0	1
755	1	348	0	157	148	0	18	0	1
950	4	180	34	250	233	31	1	0	1
654	52	132	65	76	74	0	3	0	1
775	29	167	35	43	34	4	10	0	1
1032	1	628	10	158	149	4	12	0	1
973	3	561	51	799	777	22	7	0	1
873	13	224	13	793	697	191	0	0	1
658	54	392	84	209	198	0	19	0	1
686	7	606	14	413	340	138	6	0	1
778	10	372	21	352	327	37	7	0	1
1314	11	841	17	1011	960	85	12	0	1
665	17	274	21	324	315	11	2	0	1
821	26	192	51	322	319	6	0	0	1
642	1	254	9	452	381	129	3	0	1
1334	6	370	40	3152	3055	163	31	0	1
438	14	215	63	796	700	182	3	0	1
761	28	289	32	149	145	4	7	0	1
891	0	146	24	202	195	10	3	0	1
10	668	527	1868	1564	481	72	0	0	4
11	842	452	2002	1702	468	75	0	0	2
14	483	1310	839	582	111	55	0	0	1
21	997	1205	3022	2460	923	1	0	0	3
20	986	596	1217	1052	141	58	0	0	1

20	845	1992	5182	4248	1581	57	0	0	4
58	790	727	1664	1486	165	37	0	2	5
20	956	2956	1998	1627	97	133	0	5	9
22	800	1857	2292	1718	529	78	0	0	4
11	870	1309	4903	4079	1537	3	0	0	3
29	371	803	1752	1448	477	4	0	0	3
21	906	1934	1460	1349	43	20	0	0	4
13	723	1148	1620	1361	271	126	0	0	3
23	1320	1226	2251	2014	192	140	0	0	4
15	1087	889	1801	1623	180	90	0	0	7
8	1189	1503	3575	3083	969	10	0	0	3
15	1013	1050	2538	2389	50	116	0	0	3
22	345	743	1847	1569	431	4	0	0	2
22	1142	5126	2867	2127	98	243	0	1	9
19	952	3170	3361	2533	882	162	0	0	6
22	616	1968	2049	1677	526	57	0	0	4
2	188	1161	611	469	123	0	0	0	1
42	874	534	1937	1612	497	103	0	0	12
18	836	1000	608	468	69	114	0	0	4
49	1112	3337	4166	3340	897	65	0	0	5
13	341	256	813	703	143	14	0	0	1
6	272	427	166	149	13	0	0	0	2
5	349	977	561	453	48	52	0	0	1
4	308	691	163	116	6	11	0	0	1
11	631	2391	4347	3498	1139	60	0	2	5
29	1366	3308	4079	3589	217	144	0	1	8
17	747	435	493	449	48	12	0	0	6
24	1074	2254	4171	3548	623	48	0	0	5
28	535	685	627	514	18	32	0	0	4
15	1506	1538	4564	4155	639	75	0	2	3
14	458	878	682	577	82	93	0	0	3
18	529	266	889	782	32	96	0	0	3
9	548	1340	4862	4060	1539	3	0	0	5
8	636	900	2728	2474	214	83	0	0	0
14	898	965	3290	2673	931	102	0	0	6
19	605	4903	3250	2178	792	57	0	1	7
5	666	223	716	628	155	11	0	0	2
19	431	962	506	464	11	1	0	0	2
10	850	756	1749	1507	424	25	0	0	6
10	223	103	281	230	27	11	0	0	0
7	276	2109	1982	1571	526	56	0	0	3
12	355	255	946	813	143	58	0	0	1
2	606	543	847	791	35	56	0	0	1
5	72	2659	277	61	15	0	0	0	4
11	442	1225	2866	2407	832	57	0	0	1
8	802	1615	2239	1825	562	56	0	0	2
24	1033	1620	4955	4094	1629	68	0	0	8

12	669	1200	2280	1932	629	13	0	0	2
34	720	2892	3923	3129	956	17	0	0	3
28	571	1705	1901	1769	40	13	0	5	5
19	940	909	718	615	45	10	0	0	2
26	420	2828	2135	1607	596	9	0	1	10
24	605	630	1061	815	292	3	0	0	2
11	190	796	234	218	8	10	0	0	1
17	647	754	3721	3190	887	74	0	0	4
6	683	323	1068	875	206	93	0	0	7
28	555	925	634	580	42	2	0	0	5
24	305	2085	283	68	4	0	0	0	5
26	484	321	1226	1009	209	151	0	0	2
21	754	1304	2834	2280	867	212	0	0	3
32	315	586	1610	1305	451	18	0	0	4
20	672	1101	1675	1364	464	79	0	0	2
12	626	965	1236	1192	33	8	0	0	3
8	581	260	611	569	31	4	0	0	2
8	208	788	128	85	5	10	0	0	1
12	313	1480	1312	1082	352	2	0	0	2
23	1055	2776	2443	1852	519	57	0	0	2
18	985	1202	1340	1236	69	119	0	0	0
43	657	691	806	587	122	256	0	0	4
20	629	873	524	415	34	79	0	0	1
7	400	2393	4246	3692	910	43	0	0	13
20	542	1046	1575	1319	455	2	0	0	3
8	232	462	162	148	12	1	0	0	1
19	628	1586	3127	2607	863	58	0	0	1
20	610	322	501	459	40	18	0	0	2
12	415	1141	2320	1907	730	0	0	0	1
11	668	506	2060	1754	466	63	0	0	3
7	234	181	670	597	137	2	0	0	0
14	305	1434	1497	1217	472	12	0	1	7
7	897	2453	1498	1165	162	108	0	1	6
10	301	2449	3022	2433	811	9	0	0	3
9	329	903	428	395	31	1	0	0	1
13	1196	1361	2845	2393	658	10	0	0	3
12	2080	1379	6962	6393	1053	55	0	0	6
1	215	760	2029	1743	461	59	0	0	1
12	975	1050	3617	3332	402	67	0	0	1
14	383	1369	2485	2182	455	23	0	0	1
21	906	1934	1460	1349	43	20	0	0	4
19	922	3035	3460	2717	915	10	0	0	4
32	228	209	514	429	38	72	0	0	3
26	535	4206	3159	2428	485	116	0	2	5
10	92	977	262	169	1	0	0	0	2
32	759	585	821	678	150	65	0	0	2
15	848	4404	2247	1543	480	4	0	1	7

18	200	514	178	159	29	0	0	0	1
12	125	258	853	727	231	10	0	0	4
15	726	856	419	299	32	94	0	0	3
22	347	1014	406	337	16	2	0	0	9
21	325	937	182	127	18	2	0	0	3
24	331	989	225	119	27	3	0	0	1
26	878	705	3538	2948	922	76	0	0	5
21	546	3167	3590	2843	858	63	0	0	4
12	882	1401	3340	2893	619	97	0	0	2
33	923	11930	4257	2495	980	59	0	4	16
35	1301	707	3128	2569	979	4	0	1	8
3	221	908	404	280	94	11	0	0	1
18	668	3096	3238	2515	877	63	0	0	7
33	964	1043	5193	4490	1275	19	0	0	9
13	554	3044	2934	2190	885	58	0	0	7
41	1171	1693	1982	1616	338	150	0	1	7
38	828	589	941	690	153	59	0	0	4
18	706	1428	5676	4814	1572	64	0	0	4
3	350	802	1442	1323	156	15	0	0	0
31	735	1160	1276	1073	140	61	0	0	2
2	157	163	127	115	6	10	0	0	1
3	543	1559	2256	1822	661	78	0	0	2
27	1306	1593	2202	1852	500	64	0	0	5
4	405	657	1528	1231	343	17	0	0	2
11	760	1371	5540	4588	1606	155	0	0	0
9	266	883	1887	1573	564	56	0	0	2
1	372	699	889	696	246	31	0	0	0
52	1082	2499	2284	1753	541	62	0	0	13
14	430	750	2099	1757	561	55	0	0	1
20	1185	726	1997	1593	568	149	0	0	2
15	1861	3006	4716	3848	973	239	0	0	3
11	840	2010	5614	4682	1699	57	0	0	1
11	663	415	1993	1686	471	5	0	0	1
27	698	661	766	633	94	101	0	0	6
13	958	1233	4775	3889	1294	408	0	0	2
2	136	225	137	101	6	10	0	0	1
33	938	3106	2247	1689	539	57	0	0	5
2	156	441	1426	1174	443	55	0	0	1
25	922	3083	2676	2070	563	57	0	0	8
4	933	2046	5570	4583	1623	123	0	0	1
6	680	1023	3596	3076	965	43	0	0	2
3	267	337	182	127	22	11	0	0	0
27	1178	3340	3184	2538	611	82	0	0	8
15	523	896	1761	1440	507	2	0	0	1
8	530	527	2557	2412	122	26	0	0	3
41	684	4274	5273	4074	1549	4	0	1	8
31	1093	2102	6935	5931	1682	137	0	0	1

13	577	1484	4058	3492	1042	9	0	0	4
30	727	1994	6927	5841	1977	74	0	0	1
17	751	2717	2697	2364	114	48	0	0	6
13	376	687	1408	1271	89	102	0	0	2
23	577	2151	1735	1377	147	157	0	0	5
27	694	1116	666	455	69	5	0	0	2
3	205	194	869	787	168	1	0	0	0
35	898	4064	3550	3012	462	20	0	1	6
7	123	1535	538	404	22	2	0	0	4
16	805	741	3815	3268	1060	5	0	0	2
13	515	2349	1481	1104	268	61	1	6	24
14	664	532	941	719	97	269	0	0	0
21	726	1746	3238	2648	893	6	0	0	6
23	788	2877	5400	4241	1583	61	0	0	6
15	288	829	362	311	44	2	0	0	3
53	1028	341	629	560	52	14	0	0	4
19	715	2598	3908	3325	688	16	0	0	6
49	953	5020	8292	6567	2571	64	0	1	8
9	227	2212	422	355	17	1	0	0	2
28	1359	2905	571	219	26	2	0	0	2
21	589	1320	1614	1311	296	62	0	0	2
29	1426	3410	11200	9384	3073	96	0	0	2
12	595	1143	517	443	113	3	0	0	1
21	786	392	815	711	116	5	0	0	9
7	456	1060	1756	1465	461	56	0	0	2
24	507	2482	532	261	21	57	0	0	7
4	252	879	1841	1535	528	58	0	0	2
33	920	1739	3836	3246	1083	60	0	0	3
39	1108	2772	3588	2763	954	15	0	0	3
11	954	657	1467	1292	237	63	0	0	1
20	1129	1219	4834	4206	962	51	0	0	3
19	571	1445	3961	3342	1128	6	0	0	1
28	647	1054	2389	1911	580	58	0	1	15
3	415	518	433	370	20	13	0	0	2
17	481	2100	3111	2467	817	56	0	0	4
25	1685	2779	4866	4231	613	132	0	0	8
10	728	1021	525	386	116	4	0	0	3
8	635	1016	1989	1637	533	58	0	0	2
36	649	992	4838	3960	1620	100	0	0	3
23	1034	3219	3826	3167	588	14	0	0	5
27	882	3121	3081	2356	859	10	0	0	6
7	600	1551	462	286	53	1	0	0	2
13	825	452	2013	1788	441	6	0	0	3
19	953	573	2824	2668	138	54	0	0	0
14	621	1040	823	673	164	12	0	0	8
46	1207	3732	2222	1803	26	17	0	1	7
10	203	1640	1500	1188	487	0	0	0	1

23	571	774	817	738	41	5	0	0	5
19	1466	1115	1225	1033	154	123	0	0	4
43	514	613	443	420	17	5	0	0	1
14	749	637	1046	880	133	60	0	0	4
11	243	490	416	359	45	15	0	0	4
5	199	134	114	106	7	1	0	0	1
6	164	500	878	732	209	32	0	0	1
18	672	1778	2710	2446	445	19	0	0	5
22	770	2952	727	407	48	3	0	0	6
5	414	602	916	838	74	44	0	0	1
14	656	606	838	750	131	3	0	0	4
3	373	1178	2091	1815	416	19	0	0	2
11	698	1235	482	400	40	1	0	0	3
14	608	168	443	331	87	3	0	0	0
25	846	1198	2496	2131	600	14	0	0	5
38	426	1126	713	516	42	7	0	0	1
15	931	853	4346	3621	1338	14	0	0	5
27	962	904	4296	3493	1309	17	0	0	8
22	586	1212	752	620	45	59	0	0	4
11	531	755	2190	1900	452	8	0	0	4
16	294	391	1149	1013	151	59	0	0	4
16	192	2687	646	395	29	1	0	0	5
6	245	859	179	140	33	2	0	0	5
15	858	1660	5053	4160	1552	21	0	0	6
10	230	152	319	252	30	3	0	0	1
17	376	461	1821	1498	499	60	0	0	3
4	370	652	1840	1576	451	60	0	0	2
15	981	2147	6006	5021	1575	67	0	0	4
7	154	198	336	310	7	10	0	0	1
15	874	684	1745	1434	472	64	0	0	3
0	124	240	74	57	1	0	0	0	1
14	602	598	3187	2648	917	122	0	0	0
14	1171	1211	2261	1837	549	159	0	0	2
8	186	479	106	99	5	0	0	0	6
6	210	125	104	95	5	1	0	0	0
27	597	2167	6699	5521	2076	60	0	0	2
21	958	1874	4430	3638	1194	62	0	2	6
10	1107	3582	6150	4950	1749	169	0	0	4
4	469	644	477	359	52	107	0	0	1
59	766	1278	1752	1453	508	16	0	0	3
22	2047	3109	5297	4205	1597	106	0	0	5
1	496	1232	5628	4748	1663	69	0	0	2
13	412	1002	1229	1060	311	4	0	0	2
21	2223	2072	3983	3272	1022	164	0	0	3
4	711	1848	1121	832	53	131	0	0	2
23	610	749	1794	1490	508	56	0	0	6

Vitamin B6	Folate	Vitamin B12 (	Pantothen	Biotin (mcg	Vitamin C (	Vitamin D	Vitamin E (	F (mcg)	Si (mcg)
5	308	3	3	0	32	21	5	0	81
7	261	3	3	1	35	25	5	0	94
6	291	3	1	0	19	23	3	0	94
5	330	3	2	0	35	48	3	0	103
8	283	3	3	0	24	23	3	0	59
6	228	3	2	0	18	44	2	0	59
2	166	2	2	0	30	13	0	0	36
3	235	3	3	0	24	24	1	0	29
12	365	3	1	0	21	13	0	0	45
6	216	3	3	0	13	29	2	0	30
9	344	3	3	0	34	34	7	0	135
3	357	3	2	0	36	40	1	0	109
14	335	3	1	1	34	32	1	0	120
5	206	3	1	0	17	12	1	0	52
4	254	3	1	0	22	30	0	0	44
5	205	3	1	1	12	51	0	0	63
7	200	2	3	1	13	52	2	0	75
16	365	3	3	0	35	29	5	0	97
11	389	3	3	1	61	63	1	0	115
3	242	3	2	0	26	13	1	0	90
6	380	3	3	0	48	42	0	0	100
8	337	3	3	0	51	37	1	0	76
11	402	3	3	1	63	44	1	0	83
8	337	3	1	0	31	71	3	0	129
14	370	3	1	0	39	52	3	0	119
5	302	3	2	0	36	59	0	0	125
3	135	1	1	0	7	26	1	0	39
16	464	3	3	1	27	26	4	0	47
5	204	3	2	2	27	16	6	0	126
13	461	3	3	0	26	59	5	0	93
5	294	3	1	0	27	53	0	0	152
8	269	3	1	0	10	19	1	0	68
6	284	3	1	0	15	31	1	0	155
9	366	3	1	0	23	103	1	0	132
4	196	2	1	0	6	13	1	0	38
1	78	1	1	0	4	8	0	0	49
6	296	3	3	0	44	54	3	0	96
7	417	3	1	0	26	11	3	0	97
4	280	3	2	0	36	10	1	0	45
8	296	3	1	0	20	17	3	0	73
9	447	3	1	0	21	21	1	0	82
7	304	3	1	0	20	54	4	0	141
8	256	3	1	1	19	25	2	0	125
4	240	3	1	0	11	30	1	0	120
7	515	3	3	1	69	77	5	0	86
6	207	3	2	0	12	18	4	0	94

8	321	3	2	0	31	20	0	0	36
11	466	3	2	0	37	9	4	0	103
9	305	3	1	0	20	30	0	0	70
17	428	3	3	1	69	113	6	0	172
10	448	3	1	2	40	69	3	0	216
8	338	3	1	0	17	65	1	0	60
6	188	2	3	1	31	14	3	0	65
14	322	3	1	1	22	7	1	0	50
13	307	3	3	1	28	97	5	0	99
4	275	3	3	1	48	41	8	0	187
6	173	2	1	1	16	30	1	0	84
8	262	3	1	1	22	33	3	0	96
13	366	3	1	1	22	30	1	0	89
12	332	3	3	1	52	34	0	0	68
6	265	3	1	0	12	21	1	0	71
8	297	3	3	2	33	27	10	0	230
17	274	3	3	1	21	52	15	0	162
5	338	3	3	0	60	41	3	0	107
7	253	3	2	1	18	11	4	0	129
17	357	3	3	2	37	17	2	0	57
13	449	3	3	1	68	25	3	0	67
14	272	3	3	1	17	27	2	0	109
13	323	3	1	1	30	57	1	0	153
1	88	1	1	0	6	22	1	0	59
0	68	1	1	0	6	10	1	0	50
7	304	3	1	1	16	46	1	0	65
7	348	3	1	0	14	46	1	0	75
6	240	3	1	0	9	16	0	0	34
7	264	3	2	0	21	14	0	0	31
12	298	3	3	0	9	14	5	0	39
6	375	3	3	1	67	19	2	0	51
8	373	3	3	2	68	19	1	0	66
11	331	3	2	0	13	14	4	0	58
11	296	3	3	3	47	63	5	0	219
10	315	3	2	1	32	20	1	0	56
16	380	3	1	1	23	53	0	0	63
8	256	3	3	1	20	14	2	0	45
7	241	3	2	0	18	25	6	0	135
9	339	3	2	0	32	28	4	0	122
12	362	3	3	0	56	22	2	0	58
3	207	3	2	0	21	7	0	0	48
4	263	3	2	0	28	25	1	0	60
7	270	3	1	1	22	78	0	0	65
7	241	3	2	3	37	28	3	0	110
5	204	3	3	0	29	9	4	0	70
5	170	2	3	0	7	5	3	0	42
13	348	3	1	0	12	30	0	0	210

5	185	2	2	0	19	19	4	0	82
2	181	2	1	0	12	32	1	0	56
9	251	3	3	2	29	165	3	0	207
7	270	3	1	1	22	78	0	0	65
10	375	3	3	0	32	109	5	0	65
8	157	2	3	0	14	35	3	0	25
11	233	3	1	0	14	43	0	0	27
8	209	3	3	0	32	29	5	0	144
10	297	3	3	0	34	44	5	0	119
11	477	3	3	0	65	45	1	0	112
8	238	3	3	0	22	10	4	0	22
5	215	3	1	0	8	14	1	0	39
6	360	3	3	1	69	43	0	0	70
9	295	3	1	0	12	3	1	0	40
3	229	3	2	0	24	10	0	0	22
12	268	3	2	0	6	28	1	0	13
14	256	3	3	1	42	13	19	0	192
8	412	3	2	1	59	131	3	0	173
4	171	2	1	0	20	35	1	0	73
8	320	3	3	0	23	15	1	0	24
14	322	3	3	1	41	50	5	0	75
14	212	3	1	6	22	39	1	0	113
11	301	3	2	1	17	50	1	0	84
8	305	3	1	1	20	73	0	0	79
11	300	3	2	1	21	62	2	0	104
13	296	3	3	0	32	62	12	0	147
7	272	3	1	0	18	302	1	1	92
10	252	3	1	3	24	44	1	0	94
7	307	3	2	0	28	15	1	0	52
13	367	3	1	0	31	93	0	0	237
12	356	3	2	2	29	24	6	0	192
18	467	3	2	0	31	48	1	0	73
17	465	3	2	0	47	74	0	0	157
5	282	3	2	0	25	91	4	0	93
8	372	3	3	0	52	25	1	0	77
9	411	3	3	2	62	110	1	0	100
12	380	3	2	1	42	225	3	1	73
6	371	3	3	1	70	43	3	0	72
4	287	3	2	0	36	18	3	0	75
10	352	3	2	0	30	32	1	0	77
2	202	3	2	0	29	46	1	0	57
2	18	1	2	1	4	27	1	0	20
7	161	2	3	0	6	7	4	0	17
9	390	3	2	2	55	17	3	0	98
6	104	1	1	3	18	35	0	0	58
9	250	3	2	1	9	21	2	0	20
6	134	1	3	0	6	85	6	0	29

1	123	1	2	0	25	13	1	0	25
6	189	2	3	2	19	11	7	0	97
3	162	2	1	1	19	48	4	0	101
11	846	3	3	4	183	62	5	0	138
9	254	3	3	0	9	14	5	0	41
6	264	3	2	0	7	15	1	0	22
5	289	3	2	0	15	39	3	0	138
7	275	3	2	0	6	30	1	0	30
10	334	3	1	0	18	21	3	0	61
8	337	3	3	0	35	71	2	0	79
5	229	3	1	1	37	44	1	0	129
4	202	3	1	1	8	32	0	0	25
7	310	3	3	0	28	29	2	0	35
13	322	3	3	3	35	59	2	0	83
7	169	2	1	3	11	44	0	0	78
10	193	2	1	3	22	40	0	0	70
7	371	3	1	0	17	215	0	1	96
6	223	3	1	0	11	196	1	1	70
18	526	3	3	0	55	41	5	0	88
9	281	3	3	0	34	35	6	0	80
13	461	3	3	1	73	22	7	0	80
14	313	3	3	0	20	5	4	0	5
10	320	3	2	1	36	3	7	0	117
9	343	3	3	1	88	17	2	0	15
5	258	3	3	0	52	47	3	0	77
8	358	3	2	1	29	26	0	0	111
7	339	3	1	0	24	22	0	0	82
14	343	3	3	0	33	15	4	0	36
10	340	3	1	0	24	27	0	0	38
23	477	3	3	3	65	37	5	0	93
11	302	3	3	0	25	7	5	0	46
8	301	3	3	1	35	12	2	0	45
9	365	3	2	0	27	4	0	0	19
17	535	3	3	1	53	46	0	0	80
7	222	3	3	0	23	5	3	0	45
8	281	3	2	1	30	17	1	0	49
5	252	3	2	0	21	9	0	0	14
3	104	1	1	0	6	6	1	0	26
9	334	3	3	2	55	21	2	0	54
9	275	3	2	1	24	8	0	0	25
5	324	3	2	0	33	7	1	0	29
6	264	3	3	2	44	7	12	0	176
20	427	3	3	1	19	14	2	0	41
5	231	3	1	0	10	45	1	0	52
6	191	2	1	0	8	16	0	0	34
7	257	3	1	0	16	9	0	0	23
4	232	3	1	0	18	13	0	0	19

24	361	3	3	5	55	28	12	0	180
5	310	3	2	0	22	7	0	0	9
13	315	3	2	0	10	9	1	0	7
17	501	3	3	1	67	46	3	0	269
21	496	3	3	1	67	58	8	0	259
7	272	3	3	0	23	8	1	0	12
17	408	3	3	3	33	17	1	0	37
6	434	3	3	1	96	12	3	0	75
10	284	3	3	0	14	12	5	0	11
9	193	2	1	3	17	35	1	0	81
8	222	3	3	0	11	38	3	0	39
8	310	3	3	0	30	13	2	0	42
6	259	3	2	1	34	40	7	0	167
9	410	3	2	0	29	10	1	0	37
12	456	3	2	1	35	28	1	0	58
13	382	3	2	1	19	15	2	0	57
6	325	3	2	0	28	26	1	0	50
11	361	3	3	0	35	8	2	0	36
6	252	3	3	0	28	15	2	0	40
8	298	3	3	0	28	31	2	0	70
7	310	3	3	0	34	9	2	0	64
6	207	3	3	0	11	25	3	0	43
7	225	3	3	0	8	24	2	0	28
5	297	3	3	0	49	24	1	0	50
5	130	1	3	0	13	14	4	0	13
12	324	3	3	3	36	53	2	0	96
18	399	3	3	1	61	36	8	0	159
8	278	3	2	0	18	8	2	0	35
16	507	3	3	5	107	43	0	0	86
13	335	3	1	0	17	16	1	0	62
6	282	3	3	0	42	24	0	0	29
10	340	3	3	1	37	20	3	0	81
12	316	3	3	1	31	22	2	0	60
8	246	3	2	2	28	49	5	0	129
6	302	3	2	0	35	16	1	0	46
7	299	3	2	0	25	8	1	0	36
7	195	2	2	0	9	52	1	0	31
26	513	3	3	0	52	27	12	0	85
7	327	3	2	0	30	24	1	0	49
9	394	3	3	2	63	15	12	0	208
4	257	3	2	0	28	9	3	0	64
12	359	3	3	0	53	19	7	0	103
11	313	3	3	0	19	24	8	0	94
6	197	2	3	0	10	32	2	0	53
3	178	2	1	0	10	18	1	0	44
13	396	3	3	1	29	53	5	0	165
19	487	3	3	0	67	56	8	0	112

15	422	3	3	0	50	21	1	0	51
7	326	3	3	0	31	20	2	0	47
10	233	3	2	3	30	54	1	0	64
7	303	3	3	0	37	14	2	0	41
7	248	3	3	1	28	14	2	0	53
3	195	2	2	0	20	10	0	0	10
6	290	3	2	0	25	7	1	0	30
6	297	3	3	0	26	8	2	0	34
6	298	3	2	0	25	6	1	0	30
4	199	2	2	0	24	8	1	0	34
6	376	3	3	1	89	16	3	0	67
14	381	3	2	0	28	55	0	0	46
16	386	3	3	3	79	36	9	0	97
11	350	3	3	3	72	32	1	0	77
7	351	3	3	0	42	16	0	0	21
6	277	3	2	0	25	21	0	0	30
11	376	3	3	1	43	72	3	0	106
13	375	3	3	0	26	21	2	0	97
6	296	3	2	0	31	11	0	0	20
18	400	3	3	1	24	24	4	0	30
6	245	3	1	0	14	6	3	0	57
7	280	3	3	0	28	64	2	0	41
12	354	3	1	0	19	13	3	0	71
11	325	3	3	0	47	21	5	0	58
9	316	3	3	0	45	22	4	0	29
12	432	3	2	0	29	17	0	0	28
12	363	3	3	1	60	10	9	0	121
13	347	3	1	2	19	16	3	0	71
18	379	3	1	0	11	10	0	0	20
10	403	3	3	1	69	37	1	0	69
4	290	3	3	1	57	22	1	0	48
11	348	3	3	1	49	21	7	0	136
11	331	3	3	0	25	11	1	0	27
8	246	3	2	3	29	22	0	0	61
10	262	3	3	1	39	68	2	0	93
6	242	3	3	0	24	8	3	0	60
10	245	3	3	0	16	5	7	0	74
7	266	3	3	0	25	11	1	0	19
9	434	3	2	0	39	55	0	0	81
5	253	3	3	0	44	10	1	0	47
4	314	3	1	0	28	47	3	0	79
13	434	3	3	0	37	12	2	0	23
0	239	1	0	12	54	1	1	53	1508
0	158	1	0	12	76	1	2	71	2365
0	114	11	0	10	33	0	0	98	151
0	213	5	0	29	46	4	0	93	334
0	157	2	0	23	63	1	1	122	1351

0	319	3	1	47	38	1	5	132	308
0	170	3	1	32	52	8	4	224	1384
0	334	22	1	37	80	1	1	163	1620
0	174	17	0	12	96	2	0	87	1591
0	213	1	0	23	29	0	2	79	718
0	179	3	0	16	53	3	5	84	1193
0	415	6	1	72	84	1	1	165	1212
0	309	3	0	37	106	0	1	103	843
0	303	5	0	51	93	4	3	197	1274
0	221	3	3	39	51	1	2	160	1162
0	262	7	0	40	53	2	0	91	450
0	313	2	0	28	116	1	1	137	1494
0	124	3	0	10	77	2	2	79	1958
0	320	45	2	35	110	1	2	246	1465
0	368	22	1	37	112	1	1	120	2997
0	405	8	1	61	80	2	5	94	229
0	134	7	0	22	85	1	1	38	475
0	260	8	1	23	144	10	5	139	2224
0	246	6	0	37	62	3	1	94	968
0	352	22	1	51	70	6	5	221	1759
0	166	1	0	7	56	2	2	46	1137
0	159	3	0	14	8	1	0	38	509
0	191	4	0	32	33	1	1	43	552
0	144	3	0	14	22	0	1	29	504
0	145	16	0	11	52	0	0	94	1418
0	369	24	1	47	101	3	4	247	1545
0	207	3	0	13	37	3	3	59	448
0	250	14	0	15	74	1	3	171	1219
0	234	4	0	14	39	2	1	78	902
0	309	3	0	35	82	1	1	181	796
0	266	3	0	33	68	2	1	87	1119
0	172	1	0	10	51	1	3	99	1242
0	269	2	0	22	36	1	0	63	224
0	93	3	0	12	36	0	2	76	612
0	137	1	2	18	32	0	2	61	1300
0	279	43	2	48	40	2	1	161	765
0	113	1	0	10	22	1	2	45	899
0	220	2	0	36	11	1	1	97	636
0	229	4	1	22	64	2	2	78	2868
0	68	0	0	3	28	0	0	29	498
0	253	13	0	32	36	0	0	56	584
0	117	1	0	6	38	1	0	49	1113
0	156	1	0	16	47	0	1	61	733
0	220	23	1	33	14	1	0	57	45
0	220	2	0	32	50	0	0	74	355
0	180	10	0	18	43	0	0	85	179
0	235	6	3	40	63	3	3	118	1348

0	248	2	0	33	59	0	1	72	182
0	266	17	1	44	44	3	1	203	440
0	248	9	0	36	52	2	0	120	1183
0	240	5	0	38	72	3	2	121	1186
0	327	21	0	46	56	4	2	126	680
0	173	2	0	27	52	2	3	114	1357
0	213	2	0	30	18	0	0	52	519
0	145	1	1	11	59	2	1	84	1212
0	227	1	0	7	24	1	0	45	1062
0	212	5	0	34	72	3	1	93	1296
0	224	20	1	16	15	3	3	94	306
0	107	1	0	12	38	2	1	91	1129
0	290	3	0	37	81	2	2	97	2476
0	250	1	0	20	50	4	4	96	2008
0	292	3	0	37	62	2	2	86	2388
0	236	3	0	35	16	2	2	90	1104
0	132	1	0	9	11	1	1	62	1077
0	224	2	0	30	15	0	0	47	508
0	265	5	1	53	26	1	2	71	112
0	268	18	0	41	69	0	1	149	336
0	237	3	1	50	62	1	2	160	1135
0	274	4	0	32	45	5	4	150	1597
0	215	3	0	35	61	2	2	88	2391
2	524	8	1	49	53	0	2	95	891
0	232	3	1	36	35	2	2	89	1441
0	155	1	0	16	12	0	0	35	671
0	223	6	0	34	54	2	2	96	1474
0	182	1	0	12	33	2	2	78	2433
0	178	4	0	20	23	1	1	66	572
0	151	1	1	5	59	1	0	63	2438
0	90	0	0	5	11	0	1	32	157
0	260	5	1	36	12	0	0	71	536
0	244	20	1	22	21	0	0	85	1145
0	149	18	0	22	71	1	0	94	523
0	192	3	0	32	47	1	1	43	564
0	256	5	0	26	31	1	1	97	962
0	252	3	0	19	21	3	3	327	1211
0	133	2	0	5	29	0	0	23	520
0	217	1	0	22	57	0	1	133	806
0	168	5	0	21	30	1	1	104	659
0	415	6	1	72	84	1	1	165	1212
0	267	21	1	35	37	2	1	132	3788
0	116	3	0	12	43	5	3	87	608
0	201	38	2	24	41	2	3	168	400
0	100	8	0	5	4	0	0	46	55
0	196	2	0	23	42	2	2	104	2644
0	314	40	2	34	30	1	2	147	1300

0	185	1	0	20	5	1	1	53	617
0	138	1	0	4	4	1	0	29	527
0	245	5	0	34	57	3	1	82	1215
0	306	5	0	40	12	3	2	92	1356
1	258	3	0	36	27	1	0	63	941
0	257	3	0	37	16	2	2	82	2176
0	145	1	1	13	107	2	2	105	2467
0	302	22	1	42	79	2	1	128	645
0	319	3	0	33	58	2	1	111	1122
1	370	116	6	58	81	2	2	259	485
0	203	3	0	18	26	6	3	104	548
0	142	5	0	11	22	0	0	29	175
0	261	22	1	40	57	3	1	112	2189
0	243	0	3	18	36	1	3	170	1240
0	262	22	1	41	74	2	1	101	904
0	314	11	0	52	78	6	3	194	637
0	151	0	1	27	36	2	0	140	932
0	241	4	0	25	38	3	5	116	1309
0	154	3	0	10	19	0	0	66	497
0	276	3	0	41	66	2	0	95	1468
0	87	0	0	3	10	0	0	7	465
0	233	9	0	37	33	2	1	62	1322
0	354	4	2	54	108	2	0	120	496
0	150	2	0	3	67	1	0	52	846
0	210	1	0	19	68	1	1	83	681
0	200	1	0	22	29	0	0	39	124
0	108	4	0	3	19	0	0	20	565
0	277	27	2	29	88	9	2	174	835
0	146	2	0	17	19	0	0	52	163
0	138	4	0	20	58	3	0	77	1051
0	252	21	1	30	133	1	4	180	641
0	249	5	0	35	47	0	1	83	784
0	120	1	0	5	39	1	1	75	719
0	298	2	0	24	39	3	6	106	2410
0	186	2	0	23	84	1	1	90	1408
0	92	1	0	1	4	0	0	10	474
0	320	24	1	47	73	4	3	141	315
0	107	0	0	6	25	0	0	9	40
0	344	22	1	42	59	2	2	150	1433
0	215	6	0	22	56	0	0	82	842
0	180	2	0	15	39	1	0	85	578
0	94	2	0	1	5	0	0	18	599
0	451	22	1	48	60	2	1	196	2017
0	164	2	0	25	28	2	0	77	278
0	252	0	0	10	141	0	4	93	1426
0	195	35	2	22	36	4	1	173	1924
0	374	2	0	46	110	2	5	157	376

0	262	5	0	33	55	2	0	94	1126
0	282	3	0	44	84	2	2	156	212
0	332	21	1	28	85	1	2	171	1506
0	205	1	0	16	94	0	0	81	1187
0	314	16	0	25	110	1	1	143	205
0	209	7	0	22	47	2	1	112	6296
0	48	0	0	0	6	0	0	24	520
0	465	25	2	72	63	2	1	211	1682
0	217	12	0	14	13	0	0	48	587
0	143	1	0	6	20	2	2	76	2293
1	410	20	1	24	44	2	2	83	783
0	161	2	0	19	73	1	1	72	2736
0	333	6	0	35	34	3	1	119	2273
0	244	20	1	18	68	1	3	118	2409
1	217	2	0	30	16	0	2	45	618
0	208	5	1	21	11	7	6	150	1468
0	245	20	2	17	20	2	5	134	260
0	324	34	1	41	66	3	4	204	2613
0	335	13	1	65	9	2	0	74	494
0	294	21	1	49	31	2	2	145	596
0	165	10	0	15	42	1	2	88	309
0	237	14	0	18	73	1	2	190	1441
0	270	3	1	43	17	0	0	69	1252
0	190	1	3	21	69	3	5	105	356
0	223	3	0	31	42	1	1	59	504
0	288	21	0	43	54	4	0	99	589
0	179	1	0	22	41	0	0	37	506
0	348	4	1	52	58	3	3	114	582
0	184	20	1	24	32	6	2	215	2169
0	187	1	0	16	40	0	0	63	578
0	259	1	0	22	72	0	1	150	611
0	262	2	0	35	31	1	2	98	595
0	156	22	0	15	84	13	6	113	333
0	197	1	0	18	40	0	1	42	1019
0	141	17	0	13	45	2	4	93	180
0	276	20	1	17	92	3	2	230	1702
0	190	7	0	19	27	1	1	62	1182
0	209	3	0	17	54	0	2	60	586
0	207	0	0	18	36	3	4	116	3571
0	288	21	1	39	79	1	2	173	1661
0	347	21	1	41	31	1	3	126	3963
0	206	13	0	19	15	0	0	49	492
0	181	1	0	6	39	2	1	85	1660
0	138	0	0	10	144	0	0	136	1297
1	360	3	0	35	31	1	1	91	3528
0	209	33	1	25	35	2	4	199	1288
0	248	7	0	34	9	0	0	53	567

0	383	2	0	26	122	2	3	114	1168
0	279	5	0	44	85	3	6	101	365
0	181	2	1	32	25	6	4	126	1057
0	297	2	0	29	87	2	4	70	260
0	194	3	0	18	40	1	1	56	410
0	85	0	0	2	1	0	0	17	489
0	129	0	0	12	23	0	0	25	105
0	398	5	1	59	37	2	4	138	1264
1	357	22	1	49	31	0	0	133	4885
0	158	1	0	12	13	0	0	42	755
0	227	4	0	19	48	3	1	67	222
0	175	4	0	23	42	0	0	67	103
0	208	9	0	25	20	2	0	60	629
0	88	0	0	6	18	1	1	43	1003
0	299	3	0	35	43	2	2	105	3687
0	239	3	0	42	119	4	2	138	2320
0	182	0	0	11	42	0	2	83	3620
0	229	1	1	18	141	2	3	113	3691
0	250	6	0	36	72	2	3	100	1515
0	101	4	1	7	34	1	0	50	605
0	182	0	1	10	45	1	1	63	291
0	243	24	1	38	18	4	1	94	779
1	312	3	0	32	28	1	1	47	1014
0	296	3	1	37	85	1	2	116	1677
0	128	1	0	6	197	2	2	33	758
0	130	3	0	12	78	3	1	63	1220
0	153	1	0	13	44	1	0	39	1065
0	281	6	0	38	60	1	2	124	1285
0	65	0	0	4	7	0	1	31	462
0	196	2	0	25	81	2	2	80	1327
0	96	0	0	7	12	0	0	5	212
0	90	0	0	5	93	1	0	57	1080
0	260	3	0	34	76	2	0	109	726
1	344	0	0	12	17	0	1	25	496
0	71	0	0	4	19	0	0	17	365
0	317	4	1	48	61	3	1	101	325
0	315	6	0	41	75	2	2	123	1340
0	287	22	1	42	167	1	2	123	2640
0	152	2	0	23	42	0	0	57	696
0	358	4	1	62	90	8	8	177	1400
0	242	21	1	25	37	2	1	123	1946
0	132	4	0	12	53	2	2	56	478
0	168	5	0	34	15	2	1	61	694
0	331	7	1	46	113	1	0	146	1356
0	122	16	0	8	53	1	1	60	622
0	212	4	0	23	56	3	7	93	151

Lycopene (	Lutein (mc	Vitamin K (mcg)
1521	643	491
1703	837	483
1637	203	1811
2126	566	892
2338	99	784
2368	199	490
297	261	1380
1626	199	52
1020	561	1871
1539	105	104
5382	908	6110
1023	972	5038
486	1107	2310
1355	483	270
5125	288	59
3560	423	1329
4475	434	1533
152	1396	2359
1227	820	2304
1205	537	2358
1064	1396	3703
3011	472	788
352	1107	342
1068	1433	4608
1099	933	7917
1940	1007	9253
515	199	23
676	423	4017
657	416	878
252	199	1899
244	1420	5469
167	293	1801
531	0	7072
118	94	25813
340	116	676
917	94	2844
2731	34	2980
1656	317	1012
561	222	592
1307	0	21
1122	199	3379
750	104	3127
2481	323	2876
3524	302	5436
3404	0	883
437	149	8599

1220	205	622
1220	110	1082
351	746	1845
443	1127	25741
2719	1099	4229
2177	128	6159
1271	323	1697
83	423	426
9156	423	861
1451	472	2142
6433	696	221
6040	730	338
7233	800	431
209	472	1894
3566	432	1058
6512	434	401
3241	393	382
263	472	2073
553	410	3387
273	738	896
408	168	647
1301	194	4354
1244	1161	4461
418	193	1438
227	204	1069
3579	457	37
2077	0	1821
295	214	531
162	199	863
336	982	974
350	373	903
314	373	1038
1203	99	21
2390	920	5970
1745	194	311
1039	1127	216
1003	657	2452
1829	944	496
6744	492	1789
2319	435	1582
257	193	1477
948	435	1037
3201	1100	494
1093	547	945
1049	184	292
328	173	301
1496	0	7511

3358	6	24
571	384	3294
4017	578	5958
3201	1100	494
3339	273	798
1833	197	6591
220	398	473
1496	219	2575
1241	491	1724
3449	1835	1574
171	293	22
338	99	126
1323	446	2610
406	203	23
168	304	22
1041	0	6065
1897	99	1169
4120	707	1321
103	490	154
1303	99	837
1227	1157	1725
295	547	556
263	696	4716
1188	551	4362
1254	447	1896
3682	1193	1246
706	688	1961
1065	920	1016
1201	99	114
1204	933	7898
6805	457	4049
4695	566	4247
377	1038	11014
1172	988	653
1093	293	1451
104	705	5892
633	501	428
1195	757	129
414	99	876
2484	222	2024
1141	110	1246
975	189	104
4131	47	58
448	189	48
42	1487	220
67	247	66
2098	23	220

860	3	831
1503	189	6
499	192	47
3004	132	92
75	15	3132
937	291	6
1049	0	2792
1174	54	85
1494	49	85
543	92	1430
1238	203	266
940	290	38
2354	98	654
651	705	141
1157	594	13066
1770	878	106
231	217	4703
3118	457	425
947	104	4240
1148	288	1764
2055	518	59
63	99	157
463	99	151
231	446	23
2160	435	47
319	770	6766
509	0	4172
4481	94	182
2053	933	487
117	1381	1790
74	0	241
969	323	161
165	238	16
976	1127	1879
1102	203	901
972	423	309
123	199	296
969	0	93
62	293	1000
165	205	4348
954	0	289
1431	110	18
492	527	178
2104	231	946
2256	273	869
84	373	21
4023	199	157

1276	947	244
880	199	154
46	110	156
292	657	7687
409	657	7875
985	199	245
249	448	173
1034	293	417
731	37	27
1056	647	889
2075	186	80
1047	472	168
7198	906	327
975	199	84
292	194	1019
155	696	177
1120	199	919
1547	11	379
1004	223	302
9865	0	2136
2473	216	1244
269	293	169
1075	199	1087
368	746	33
214	122	79
2092	647	1380
1089	657	3380
175	199	159
4450	1418	134
6417	397	296
1149	446	300
2464	446	1347
1177	592	536
2436	640	1151
1058	288	440
1058	110	291
1057	193	910
4565	293	1512
1135	249	472
1523	492	1057
2452	99	21
1074	110	3161
9671	94	151
10517	105	177
1404	409	31
2414	1157	6172
1237	490	1784

1034	293	1756
213	572	263
238	894	928
986	299	303
1065	423	308
892	213	149
1058	0	292
965	199	297
1039	99	303
965	199	157
1097	397	1042
311	199	806
3820	1129	90
689	1129	83
1310	412	154
1031	94	1064
1060	1127	1883
958	463	3374
1170	284	171
480	527	51
407	0	396
1169	4	1019
949	213	178
485	757	177
513	775	43
4016	273	420
297	423	14
175	99	7
509	0	428
2058	831	1810
2062	138	1055
735	746	190
2336	39	305
1045	647	472
299	1127	1881
1096	122	858
327	39	890
1101	299	304
2116	472	6110
1070	280	388
160	757	33
903	458	276
462	613	39
561	1007	96
144	840	58
292	1797	36
626	2619	174

701	801	93
228	5136	520
68	5240	485
612	675	41
515	609	93
0	177	40
235	3017	347
296	2837	199
1061	6632	597
678	5343	496
573	1453	171
435	7010	381
436	467	67
693	7274	772
290	598	107
231	621	31
48	1803	57
679	2072	94
726	1116	84
247	4581	299
223	225	27
86	101	68
124	380	79
214	209	44
156	2030	142
215	14190	1221
309	106	53
270	9234	733
250	724	77
216	12720	1078
121	441	130
320	1230	162
320	488	37
39	8009	581
45	601	105
118	691	58
114	1311	77
74	1768	145
696	439	78
172	311	32
48	1126	83
293	845	58
250	2146	177
94	9	17
292	1445	151
251	1206	144
715	1381	73

99	737	63
320	3731	330
357	6449	536
235	2277	316
574	3393	47
320	1750	56
73	634	52
435	1443	117
216	493	94
264	1800	186
148	52	38
594	511	47
312	2005	68
223	61	10
223	1546	20
262	4710	278
262	2044	80
34	341	21
213	3544	86
184	1428	148
421	4144	525
305	1231	232
262	376	28
145	4653	194
273	356	16
192	247	54
138	2467	78
312	451	29
123	619	44
273	2448	69
4	907	64
446	657	38
104	1524	91
333	1545	157
78	136	35
276	1708	138
67	13820	1426
71	1971	74
131	7119	636
6	2966	319
235	3017	347
173	2433	128
124	565	84
293	2808	160
111	574	58
204	3593	29
316	4075	195

49	371	36
99	330	15
329	656	95
198	22	25
312	64	39
199	61	27
543	2924	132
560	3662	133
173	5921	447
350	870	93
296	2194	32
71	337	33
223	2020	30
677	6634	256
350	1991	26
742	3943	532
323	750	18
347	2947	175
230	2172	204
285	2639	152
38	110	21
111	622	71
423	962	44
58	2534	132
424	1300	148
98	691	18
144	482	42
489	635	35
193	779	21
273	760	14
139	4142	425
404	2419	89
136	2620	196
307	1080	159
424	1516	131
60	165	27
344	638	28
52	558	4
193	2984	203
545	2723	322
385	3646	419
37	137	16
273	5000	491
273	190	16
541	3991	265
174	263	43
603	7748	298

397	1915	158
309	2723	172
202	5359	452
164	2810	184
201	4891	232
273	313	50
14	396	54
638	3857	279
39	761	50
138	1179	107
68	4178	123
247	775	60
177	888	106
792	679	22
74	384	70
238	542	53
647	3084	191
193	962	87
49	501	60
116	446	76
505	903	43
514	9905	898
193	108	32
754	325	48
86	1418	109
199	796	43
150	1262	91
113	4165	117
90	2652	304
89	2370	196
296	7430	556
436	3114	107
214	2368	114
400	30	51
524	2037	89
63	12940	1067
173	280	66
138	4081	105
472	1900	75
360	4990	433
133	3245	65
230	3051	27
199	1101	220
389	6430	458
406	973	107
184	3858	306
98	377	20

262	1021	196
309	1398	96
287	791	106
472	3380	64
300	219	18
73	277	22
48	870	37
327	4283	266
273	306	55
92	2731	358
296	2174	58
160	4986	379
223	225	33
198	803	28
533	4231	159
362	44	8
717	3629	97
980	3257	50
238	3737	55
248	3215	188
423	3836	33
238	27	19
104	2969	10
642	2713	159
293	32	19
288	638	40
261	1138	70
587	2809	243
193	420	53
293	607	70
0	78	13
148	1661	73
312	3105	248
86	296	19
86	286	21
759	731	21
622	1797	212
499	2393	261
43	702	42
472	315	32
293	686	97
112	3336	377
411	414	74
380	3934	352
57	1551	83
161	695	63
