# Supplementary material for: Effects of Cucurbita Moschata squash (Butternut) seed paste in improving zinc and iron status in children attending Early Childhood Development centres in Limpopo province, South Africa
Source: PLoS One. 2024 Apr 18;19(4):e0300845. doi: 10.1371/journal.pone.0300845 (PMC11025940; doi:10.1371/journal.pone.0300845)
Supplement: S1 Protocol — (DOCX) [file pone.0300845.s002.docx]

**EFFICACY OF *CUCURBITA MOSCHATA* SEED PASTE IN THE MANAGEMENT OF IRON AND ZINC DEFICIENCIES IN PRESCHOOL CHILDREN AGED 3-5 YEARS OLD IN VHEMBE DISTRICT, LIMPOPO PROVINCE, SOUTH AFRICA.**

STUDENT NAME: Mr. SELEKANE ANANIAS MOTADI

STUDENT NO: 17640954

Proposed Degree: PhD in Nutritional Sciences

DIVISION OF HUMAN NUTRITION

FACULTY OF MEDICINE AND HEALTH SCIENCES

STELLENBOSCH UNIVERSITY

PROMOTER: PROF XG MBHENYANE

Division Human Nutrition, SU

CO-PROMOTER: PROF J FREELAND-GRAVES

Department of Nutritional Sciences, University of Texas, Austin

2018

**Table of content**

**1.** **Background and motivation** 1

1.1. Overview 1

1.2. Micronutrient deficiency and nutritional status of children 4

1.3. Dietary patterns of preschool children 5

1.4. Zinc nutrition 7

1.4.1. Prevalence of zinc deficiency 7

1.4.2. Consequences of zinc deficiency in children 9

1.5. Iron nutrition 10

1.5.1. Prevalence of iron deficiency 10

1.5.2. Consequences of iron deficiency 12

1.6. Consumption of fruits and vegetables by children under the age of 5 years 13

1.6.1 Fruits and vegetables as sources of iron and zinc 14

1.7. *Cucurbita Moschata* (Butternut) seeds 15

1.7.1. Issues of Microbial and safety 18

1.8. Early childhood development centers as a point for lifestyle interventions 18

1.8.1. Early child development (ECD) Policy 19

1.9. Problem statement 20

**2. Motivation for the study** 21

2.1. Research question 22

2.2. Hypothesis 22

2.3. Aim of the study 22

2.4. Objectives of the study 22

2.5. Expected outcomes 23

2.6. Significance of the study 24

**3. Methods** 25

3.1 Design of study 25

3.2. Target population 25

3.3. Study Location 26

3.4. Sampling 26

3.5. Inclusion and exclusion criteria 27

3.6. Procedures of Data collection 27

- - 1. Phase One: Development of the intervention 27
    2. Phase Two: Baseline Survey 31
    3. Phase Three: The Intervention 41
    4. Phase Four: Post feeding trial 44
  1. Fieldworkers 45
  2. Institutional approval 46
  3. Ethical considerations 47
  4. Budget and sources of income 48
  5. Dissemination of information 49
  6. Selekane’s PhD plan of action 50

**References 50**

**Appendices**

Appendix A: Hedonic Scale 65

Appendix B: Baseline Survey Questionnaire 66

Appendix C: 24-hour recall 72

Appendix D: FFQ 74

Appendix E: Plate waste observation form 85

Appendix F: Food Diary 86

Appendix GL permission letter to Head master 87

Appendix H: Parents’ Information and consent form 88

**LIST OF FIGURES**

Figure 1: Figure1: Cucurbita Moschata Fruit and seeds (Department: Agriculture,

Forestry and Fisheries, 2017). 15

Figure 2: An illustration of the methodological sequencing of the phases 25

Figure 3: Recipe standardization cycle adopted from (Spears & Gregoire, 2010) 28

Figure 4: MUAC measuring tape. 34

Figure 5: MUAC interpretation scale

**LIST OF TABLES**

Table 1.1: Vegetables and fruits that contain iron and zinc. 14

Table 1.2: Levels of micronutrients in *Cucurbita moschata* seeds. 16

Table 3.1: Z-scores classification (<http://www.who.into/nutgrowthdb/intro>). 39

Table 3.2: An indication of lower cut-offs for serum zinc concentration (μmol/dL)

by age group, gender, time of day and time since last meal. 40

Table 3.3 An indication of lower cut-offs of serum iron concentration (μg/L) 40

Table 3.4: Schematic representation of the feeding trial. 43

Table 3.5: Proposed data analysis table 45

Table 3.6: Personnel responsibilities during data collection 46

Table 3.7: Proposed budget for the study 48

Table 3.8: Sources of Income 49

Table 3.8: Selekane’s PHD plan of action: 49

**LIST OF DEFINITIONS**

**Micronutrient deficiency** is a lack of essential vitamins and minerals required in small amounts by the body for proper growth and development.

***Cucurbita moschata*** is a species originating in either Central America or northern South America. It includes cultivars known as squash or pumpkin. C. moschata cultivars are generally more tolerant of hot, humid weather than cultivars of C. maxima or C. pepo.

**Undernutrition** is defined as insufficient intake of energy and nutrients to meet an individual's needs to maintain good health.

**Recipe is** a set of instructions for preparing a particular dish, including a list of the ingredients required

**LIST OF ABBREVIATION**

**CM:** *Cucurbita Moschata*

**UNICEF:** United Nations International Children's Fund.

**MND’s:** Micronutrient Deficiencies.

**WHO:** World Health Organization.

**SANHANES:** South African National Health and Nutrition Examination Survey.

**IDA:** Iron Deficiency Anaemia.

**ZnD:** Zinc Deficiency.

**Hb:** Hemoglobin.

**FAO:** Food and Agriculture Organization.

**IBGE:** Brazilian Institute of Geography and Statistics.

**DNA:** Deoxyribonucleic acid.

**NFCS**: National Food Consumption Survey.

**SAVACG:** South African Vitamin A Consultative Group.

**UK:** United Kingdom.

**FBDGs:** South African Food Based Dietary Guidelines.

**ECD:** Early Childhood Development.

**DoH:** [Department of Health](https://www.google.co.za/url?sa=t&rct=j&q=&esrc=s&source=web&cd=1&cad=rja&uact=8&ved=0ahUKEwih7Zvb8NTaAhViJMAKHayBDzcQFggoMAA&url=http%3A%2F%2Fwww.health.gov.za%2F&usg=AOvVaw1xxQVcVU0Fgk9XHH403c8B).

**RCT:** Randomized Control Trial.

**Stats SA:** [Statistics South Africa.](https://www.google.co.za/url?sa=t&rct=j&q=&esrc=s&source=web&cd=1&cad=rja&uact=8&ved=0ahUKEwinjOis8dTaAhVsK8AKHfF1BOsQFggoMAA&url=http%3A%2F%2Fwww.statssa.gov.za%2F&usg=AOvVaw2jl6j466BaZIJ-AAsJwzQ5)

**HACCP:** Hazard Analysis Critical Control Point.

**MUAC:** Mid Upper Arm Circumference.

**FFQ:** Food Frequency Questionnaire.

**CRP:** C- reactive protein.

**BMI:** Body Mass Index.

**IZiNCG:** [International Zinc Nutrition Consultative Group.](https://www.google.co.za/url?sa=t&rct=j&q=&esrc=s&source=web&cd=8&cad=rja&uact=8&ved=0ahUKEwiI2LeK89TaAhWJIsAKHe8MB6EQFgg9MAc&url=https%3A%2F%2Fwww.ncbi.nlm.nih.gov%2Fpubmed%2F18046856&usg=AOvVaw3p7LpTCgBIFo0jHHJzA42f)

**[HREC:](https://www.google.co.za/url?sa=t&rct=j&q=&esrc=s&source=web&cd=8&cad=rja&uact=8&ved=0ahUKEwiI2LeK89TaAhWJIsAKHe8MB6EQFgg9MAc&url=https%3A%2F%2Fwww.ncbi.nlm.nih.gov%2Fpubmed%2F18046856&usg=AOvVaw3p7LpTCgBIFo0jHHJzA42f)** [Health Research Ethics committee.](https://www.google.co.za/url?sa=t&rct=j&q=&esrc=s&source=web&cd=8&cad=rja&uact=8&ved=0ahUKEwiI2LeK89TaAhWJIsAKHe8MB6EQFgg9MAc&url=https%3A%2F%2Fwww.ncbi.nlm.nih.gov%2Fpubmed%2F18046856&usg=AOvVaw3p7LpTCgBIFo0jHHJzA42f)

**WAZ:** Weight for age.

**HAZ:** Height for age.

**WHZ:** Weight for height.

**SPSS:** [Statistical Package for Social Sciences.](https://www.googleadservices.com/pagead/aclk?sa=L&ai=DChcSEwi6idq4tNXaAhXOvO0KHfB9DBsYABAHGgJkZw&ohost=www.google.co.za&cid=CAESEeD2xJzsylunafLJ-tb0LTAr&sig=AOD64_3VQG5yfteNjm1v6137_5xuz0LJyg&q=&ved=0ahUKEwintNW4tNXaAhWqAcAKHesOB0QQ0QwINg&adurl=)

**[CSIR:](https://www.googleadservices.com/pagead/aclk?sa=L&ai=DChcSEwi6idq4tNXaAhXOvO0KHfB9DBsYABAHGgJkZw&ohost=www.google.co.za&cid=CAESEeD2xJzsylunafLJ-tb0LTAr&sig=AOD64_3VQG5yfteNjm1v6137_5xuz0LJyg&q=&ved=0ahUKEwintNW4tNXaAhWqAcAKHesOB0QQ0QwINg&adurl=)** [Council for Scientific and Industrial Research.](https://www.googleadservices.com/pagead/aclk?sa=L&ai=DChcSEwi6idq4tNXaAhXOvO0KHfB9DBsYABAHGgJkZw&ohost=www.google.co.za&cid=CAESEeD2xJzsylunafLJ-tb0LTAr&sig=AOD64_3VQG5yfteNjm1v6137_5xuz0LJyg&q=&ved=0ahUKEwintNW4tNXaAhWqAcAKHesOB0QQ0QwINg&adurl=)

1. **BACKGROUND AND MOTIVATION**

**1.1 Overview**

In Africa more than 2 billion people in the world today are estimated to be deficient in key vitamins and minerals of these vitamin A, iodine, iron and zinc. These deficiencies affect the most vulnerable groups, which are children and pregnant women (Engle-Stone *et al*., 2014). Micronutrient malnutrition, also known as “hidden hunger” and its consequences, often go unnoticed. Children from underprivileged and poor families in developing countries are at specific risk of these nutritional deficiencies due to inaccessibility to food, unequal distribution of food within the household, dietary taboos, infectious disease, low nutrient content in the diet and poor bioavailability (Amare *et al*., 2012).

Although good nutrition is accepted universally as a basic human right, it is estimated that more than 800 million people suffer from malnutrition globally in developing countries. Additionally, more than 20% of the populations are hungry (Khan Khattak and Ali, 2010). Micronutrient deficiencies are common in middle and low-income countries; coexistence of these multiple micronutrient deficiencies frequently occurs (Bailey *et al*., 2015). Manifestation of micronutrient deficiencies become less visible and begins to show when the condition is severe, and has already led to serious health burdens ([Bhandari](https://www.hindawi.com/40646590/) and [Banjara](https://www.hindawi.com/52430675/), 2015). Many children suffer from micronutrient deficiencies, which is caused by the lack of proper diet and shortage of food (Magnus, 2012; UNICEF, 2014). Certain diseases such as malaria, tuberculosis, protein-energy malnutrition and parasitic infestations such as hookworm aggravate these conditions, that reduce the capacity of the body to retain micronutrients (Smith and Brooker, 2010).

The most common deficiencies are vitamin A, folate, iron, iodine, and zinc; however, several other micronutrient deficiency (MND’s) disorders exist, including for selenium (Etani et al., 2014; Bailey *et al*., 2015). Micronutrient deficiencies may be coupled with protein or energy malnutrition and occur as part of a cycle of malnutrition. Children under five years of age are the most vulnerable group for these micronutrient deficiencies (Zlotkin, 2011). Muthayya *et al*. (2013) estimated global hidden hunger indices for iron, vitamin A, and zinc together. They determined that 18 of the 20 countries with the highest burden of multiple MNDs were in Africa, with Afghanistan and India (WHO region Asia) completing the list. According to Andersson *et al*. (2012), deficiencies of selenium, iron and vitamin A can exacerbate iodine deficiency, by altering thyroid function. These contribute to poor growth, intellectual impairments, increased risk of morbidity and mortality (Bailey *et al*., 2015), poor cognition, poor immune functions and reduced capacity of work (Smith and Brooker, 2010).

Deficiencies of iron, vitamin A, folic acid, zinc and iodine are considered public health problems because they affect a significant proportion of children in South Africa (Shisana et al., 2013). Micronutrient deficiency is more likely to occur in children as they are in a growth and development phase and have nutritional requirements that vary according to the stage of growth (Prieto and Cid, 2011). Micronutrient deficiencies and infectious diseases often coexist and exhibit complex interactions leading to the vicious cycle of malnutrition and infections among underprivileged populations of the developing countries. These occur particularly, in preschool children (Bhaskaram, 2014).

Numerous factors may limit the inclusion of micronutrient-rich foods in the diets of children. These include poverty, poor food choices, lack of accessibility and availability to certain foods, coupled with the lack of knowledge about the importance of food group diversity for the growth and development of young children including their health ([Cole](http://ajcn.nutrition.org/search?author1=Conrad+R+Cole&sortspec=date&submit=Submit) *et al.,* 2010). In the South African diet, maize meal porridge, rice and bread are the main staple foods. These staples usually are consumed with vegetables, legumes and a small amount of animal-derived food (SANHANES-1, 2013). Meat is the most important common dietary source of bioavailable iron and zinc. Poor quality of diets is the leading cause of micronutrient deficiency in Sub-Saharan Africa (Ferguson *et al*., 2015).

Several national nutrition programmes such as food fortification, micronutrient supplementation and food supplementation are in operation in developing countries (Serdula, 2010; Best *et al.,* 2011). They exist for the advantage of preschool children; however, the prevalence of micronutrient deficiencies among rural children under the age of five years continues to be of public health concern (Arlappa *et al*., 2011). Iron deficiency is prevalent universally, with approximately 1.62 billion people anemic. The highest prevalence is among preschool children (47%), followed by pregnant women (42%) (Salam *et al.,* 2013).

Black *et al*. (2013) reported that 4% of child deaths, and disability-adjusted life years worldwide, are due to zinc deficiency. Countries in sub-Saharan Africa and South and Southeast Asia have the greatest risk of the deficiency (Wessels and Brown, 2012). The prevalence of micronutrient deficiencies in children younger than five years in Pakistan was 43.8% exhibitive iron deficiency anaemia (IDA). In addition, 54% had Vitamin A (VAD), and 39.2%, zinc deficiency (ZnD) in 2011 (Wieser *et al*., 2017). In Africa and Asia, large segments of the population are at risk of dietary zinc and iron deficiency, resulting in a high rate of stunting (Wessels and Brown, 2012; Engle-Stone *et al*., 2014). MNDs have been recognized for years; although there has been a substantial improvement in iodine status, there has been very little progress in eradicating the micronutrient deficiencies (Horton and Steckel, 2013).

Despite the legislated fortification of staple foods in South Africa since 2003, micronutrient deficiencies persist. Nutritional deficiencies during childhood have resulted from a sustained negative balance of iron. This imbalance may be caused by inadequate dietary intake, absorption and/or utilization of iron, increased iron requirements during the growth period, or blood loss due to parasitic infections such as hookworm and malaria. According to SANHANES-1 (2013), the prevalence of anaemia in children less than five years of age was 10.7%; mild anaemia, 8.6%; and moderate anaemia 2.1%. However, no cases of severe anaemia have been reported. When comparing the results of SANHANES-1 with those of the 2005 NFCS, the prevalence of anaemia decreased by 63%, that of iron deficiency anaemia by 83.2%. and that of iron deficiency increased by 3.8%

The co-existence of iron and zinc deficiency is a major public health problem ([Cole](https://www.ncbi.nlm.nih.gov/pubmed/?term=Cole%20CR%5BAuthor%5D&cauthor=true&cauthor_uid=20147474) *et al*., 2010; Liu *et al*., 2011). These micronutrients are essential for human health. Deficiencies of these two trace elements remain a global problem, especially among women and children in developing countries (Antunes *et al*., 2010). However, it seems that dietary zinc reduces iron biochemical indices, including ferritin, haemoglobin (Hb) and transferrin saturation (Motadi *et al*., 2015). In addition, these deficiencies may be associated with infections and parasitic infestations (Setnick, 2010; Amare *et al*., 2012). The consequences of a combined two-micronutrient insufficiency may be growth faltering (Cole *et al.,* 2010; Alves *et al*., 2012), delayed development (Baumgartner *et al.,* 2013) and increased morbidity due to infectious disease (Harrison, 2010; Shaw and Friedman, 2011). All of these can dramatically affect the health, development and well-being of the child. Pre-school children will be the focus of this research, as this age is an important stage of life in which nutrition plays an important role and has long lasting effects in later life (Khan Khattak and Ali, 2010).

**1.2. Micronutrient deficiency and Nutritional status of children**

A major factor contributing to morbidity and mortality among malnourished children is micronutrient deficiencies (Saurabh *et al*., 2017). Children need vital micronutrients for adequate development and growth, especially iron, zinc, copper, vitamin A, iodine and folate (Shalaby *et al*., 2017). Undernutrition is synonymous with stunting, wasting, and deficiencies of micronutrients (Herrador *et al*., 2014). Undernutrition reduces immunological capacity to defend against diseases, and the presence of diseases deplete and deprive the body of essential nutrients (Mahan *et al*., 2012). Stunting is recognized as an indirect indicator of zinc status in some populations because zinc nutrition has been shown to have a significant impact on the growth of young children (Cole *et al.,* 2010). With limited micronutrients, the body is unable to produce sufficient enzymes, hormones, and other essential substances needed for proper growth (WHO, 2013).

According to Echessa *et al*. (2013), the deficiencies due to vitamin A, iodine, iron and zinc are characteristic of victims who live in low-income countries who are typically deficient in more than one micronutrient. The deficiencies of micronutrients will lead to growth retardation and undernutrition. In children less than five years of age micronutrient deficiencies are the main manifestation of malnutrition besides protein energy malnutrition (FAO, 2015; Abubakar *et al*., 2017). Requirements of micronutrients increase during infancy and childhood due to rapid growth and development (Zhou *et al*., 2012). Too much, or too little, of these trace elements affect nutritional status (Thomas, 2014). In low-income countries, the prevalence of micronutrient deficiencies among children contribute to impaired child development and stunted growth (Black *et al*. [2013](http://onlinelibrary.wiley.com/doi/10.1111/mcn.12243/full#mcn12243-bib-0001)). Furthermore, micronutrient intake is used as another component in measuring undernutrition in Africa (Amare *et al.,* 2012).

The consequences of wasting on children may not be apparent immediately, but it has significant effects on levels of educational attainment, acute growth faltering, reduced physical capacity and poor resistance to infections and diseases in children less than five years of age (Ibeanu *et al.,* 2012; Mamabolo and Alberts, 2013; Motadi et al., 2015). Long term nutritional deprivation in children often leads to zinc malnutrition and stunting; both are associated with delayed mental development, poor school performance and reduced intellectual capacity; all of which are the consequences of zinc deficiency (Dehghani *et al.,* 2011). Micronutrient deficiencies and parasitic infestation are responsible for high mortality rate in severe acute malnutrition (Saurabh *et al*., 2017).

Achieved height is the result of interactions between genetic endowment and both macro- and micronutrient availability during the growth period (Hegazy *et al*., 2010; Zhou *et al*., 2012). Imdad and Bhutta (2011) have identified zinc supplementation as an effective intervention to improve growth. In Pakistan Soofi *et al*. (2013) compared children receiving a micronutrient powder without zinc to those supplemented with zinc. The zinc supplemented children grew an extra 6 mm between 6 and 18 months of age, as compared to extra 3 mm in height in those without zinc. The study conducted by Motadi *et al*. (2015) in the Vhembe District of South Africa reported positive correlations between zinc and weight for height, weight for age and BMI-for-age. These findings signify that children who are wasted may have zinc deficiency, while the p value was significant, the correlation was weak. According to Vivienne *et al*. (2012) zinc deficiency in children leads to growth retardation. Similarly, in Saudi Arabia Shalaby *et al*. (2017) reported that serum iron and zinc positively correlated with weight-for-age, weight- for-height and BMI-for-age in preschool and school children.

**1.3. Dietary patterns of preschool children**

Early childhood is an important stage for shaping food consumption patterns that have implications for future health (Kudlová and Schneidrová, 2012). Children at this age are still fully dependent for food on their caregivers/parents who influence their future eating habits (Curtis *et al*., 2011). Dietary patterns denote a general profile of food and nutrient consumption based on usual eating habits (Rysha *et al*., 2017). According to Kudlová and Schneidrová (2012), proper feeding practices during early childhood stimulate psycho-social development, lead to good nutritional status and physical growth, reduce susceptibility to common childhood infections and improve resistance. In addition, Butte *et al*. (2010) reported that childhood is a stage where children require more micronutrients when compared to their energy needs.

In Kenya, Chege *et al*. (2015) reported that the Masai culture prohibits the consumption of wild animals, chicken and fish, which limits food choices. In addition, fish consumption is not common, as they perceive that aquatic animals as being not fit for human consumption. Although fish is one of the best and easily available sources of omega 3 fatty acids, it is not consumed normally. Chicken is perceived to be a bird, which should not be eaten. Additionally, green vegetables are perceived as livestock feed and are consumed rarely (Chege *et al*., 2015). Thus, most nutrition surveys have documented a high frequency of insufficient dietary intake of essential micronutrients such as vitamin A, iron and iodine in children (Labadarios *et al*., 2008; Vorster 2010). In addition, Petrou and Kupek (2010) reported that in developing countries, inadequate intake of food, energy and nutrients remains highly prevalent. South Africa is undergoing a rapid nutrition and lifestyle transition that is characterized by a shift from a traditional diet low in fat to diet high animal products, oils and fats (Vorster, 2010; Motadi *et al*., 2015).

Children like to eat foods with high energy density, especially those that are high in fat and sugar (Looney and Raynor, 2011). In addition, children prefer food that contain high levels of saturated fat and sugar and are low in dietary fibre (Fox *et al*., 2010). Butte *et al*. (2010) found that the usual dietary intakes of children, aged 2 to 3 years in the United States were high in saturated fat and sodium, and low in fibre. Yet, in Malaysia, Poh *et al*. (2012) reported that almost half of the pre-schoolers (45.3%) skipped their breakfast because they had no appetite. In South Africa maize meal porridge, bread and cereals are commonly eaten for breakfast (Shisana *et al*., 2013). In addition, more than two-thirds of children (68.4%) indicated that they ate breakfast before school, while 19% indicated that they did not (Shisana *et al*., 2013). The consumption of soft drinks and other sugary drinks in the first year of life has been reported among children aged 2 to 3 years in Brazil. This pattern may affect the consumption of fruit and vegetables, making this age group vulnerable to the development of obesity and micronutrient deficiencies (IBGE, 2011).

**1.4. Zinc nutrition**

Zinc is an imperative trace element responsible for several enzyme systems, gene expression, cell division, DNA synthesis, protein synthesis immunity, insulin secretion and reproduction (Mahan *et al*., 2012). Furthermore, zinc is responsible for normal growth and development, and cognitive function in children less than five years of age. Zinc is found in the vesicles of the mossy fiber system in the brain's hippocampus; these fibers play an important role in enhancing memory and thinking skills (Mahan *et al*., 2012).

This trace element acts as a neuromodulator at excitatory synapses and has a considerable role in the stress response and the functionality of zinc-dependent enzymes (aromatase, phosphoinositide kinase and choline acetyltransferase), contributing to maintaining brain compensatory capacity (Mahan *et al*., 2012). Zinc is required also for proper sense of taste and smell. In addition, zinc plays a vital role in the edifice of proteins and cell membranes. A finger-like structure, known as a zinc finger motif, stabilizes the structure of many proteins. The most available sources of zinc are from animal origin such as beef, pork, chicken, fish and shellfish (Ibeawuchi *et al*., 2017). Vegetables also provide a good source of zinc but are limited in its bioavailability.

**1.4.1. Prevalence of zinc deficiency**

Zinc is an essential nutrient for human health, with numerous structural, biochemical and regulatory functions (Mahan *et al*., 2012). Prevalence of zinc deficiency is articulated as the percentage of the population with plasma/serum zinc concentration below specific lower cut-offs in relation to reference data for age, sex, time of day and fasting status (Wessells and Brown, 2012). Several studies used the low plasma/serum blood zinc concentration >20%, inadequate dietary zinc intake > 25% and the presence of zinc inhibitors such as phytates, fiber, oxalates and tannins in the foods to determine the prevalence of zinc deficiency (Donangelo and King, 2012; Engle-Stone *et al*., 2014; Kumera *et al*., 2015). Phytates, oxalates, tannins and fiber inhibit iron and zinc absorption, and excess amount can lead to decreased bioavailability (Mahan *et al*., 2012).

According to Akhtar (2013), the prevalence of zinc deficiency in developing countries is quite common, as 61% of the population is at an increased risk of low dietary zinc intake. In Pakistan Ejaz and Latif (2010) reported that (54.2%) children and 37.1% preschool children are zinc deficient. Donangelo and King (2012) documented that malnutrition and poverty are the leading causes of high rate of zinc deficiency in Bangladesh, especially in the paediatric age group. Similarly, zinc deficiency is prevalent among various population groups in Sri Lanka, especially among preschool children and adolescents (Hettiarachchi and Liyanage, 2012). In Sri Lanka nearly 57.0% male and 50.0%, female preschool children were found to be zinc-deficient. In India, Kawade (2012) reported a high prevalence of zinc deficiency among children belonging to low socioeconomic groups in five major Indian states. An overall zinc deficiency of 43.8% was reported, with the highest in Orissa (51.3%), followed by Uttar Pradesh (48.1%), Gujarat (44.2%), Madhya Pradesh (38.9%), and Karnataka (36.2%).

Ibeanu *et al*. (2012) found that *14*% of preschool children in the urban areas of Nigeria were zinc deficient, while, Galetti *et al*. (2016) reported 65% of preschool children in Benin. Ibeawuchi *et al* (2017) in Udo, Nigeria indicated that the prevalence of zinc deficiency among preschool children was 87.3%. In addition, Onyeamaobi and Onimawo (2011) recorded 41.5% of children under-five as having zinc deficiency in Imo State, Nigeria. In Abuja, Steve-Edemba (2014) reported that 60% of children under the age of five (5) were zinc deficient. In addition, the same study revealed that zinc deficiency was slightly more prevalent among boys (8.1% compared to 7.8% in girls). The study done by Jaryum *et al*. (2017) suggested a high incidence of zinc deficiency of 59.09% among under five years old children, as determined by plasma zinc levels in the Kanam Local Government Area of North-central Nigeria.

Furthermore, the prevalence of zinc deficiency in 3-18 years old children was 7.9% in Shiraz, Iran (Dehghani *et al*., 2011). In South America 6.4% had a dietary inadequacy of zinc, which denotes the problem of zinc deficiency in the country (Wessells *et al*., 2012; Bresani *et al*., 2012). A study in Kibona, Uganda by Willems *et al*. (2011) reported that 94% of children had an iron deficiency in the diet while 98% had a zinc deficiency. Engle-Stone *et al*. (2014) and Kumera *et al*. (2015) documented that over 55% of children less than five (5) years in Sub-Saharan Africa were zinc deficient.

A study conducted in the Vhembe district in Limpopo province in South Africa in children, aged 3-5 years, revealed that zinc and iron deficiency were major public health problems (Motadi *et al.,* 2015). Furthermore, this study reported that 42.6% of children were zinc deficient while 28% were anaemic (Motadi *et al*., 2015). Like in many other developing populations, a national survey conducted in 2005 among South African children 1–9 years old revealed that, 27.9% were anaemic while 45.3% were zinc deficient (Labadarios *et al.,* 2008; Voster 2010). This national survey report clearly shows that micronutrient deficiencies are of public health importance among South African children.

**1.4.2. Consequences zinc deficiency in children**

Among consequences of micronutrient deficiencies in preschool age children is increased risk for acute diarrhoea and pneumonia (Morales-Ruán *et al*., 2012). Young children are prone to infection and growth retardation due to zinc deficiency (Christian and Stewart, 2010; Zlotkin, 2011). If sustained the lack of micronutrients can be associated with significantly poorer performance on psychomotor and mental development scales, lower scores on cognitive function tests and lower educational achievement tests (Black *et al*., 2011; Black, 2012; Jora *et al*., 2015). Thus, zinc deficiency has serious implications for impairments of long-term cognitive ability.

Deficiency of zinc in children contributes to stunted growth and other infections (Vivienne *et al*., 2012). Bresani *et al*. (2012) documented that zinc deficiency is linked to negative outcomes such as impairment and cognitive development, increased severity of infectious diseases, and growth deficits. According to Dehghani *et al.* (2011), zinc deficiency leads to weight loss, poor appetite, rough skin and taste abnormalities. Furthermore, a deficiency of zinc can cause primary and secondary alterations in brain development and brain growth (Murray-Kolb *et al*., 2012). According to Liu *et al*. (2011) zinc is a component of enzymes that affect growth in infancy and childhood, sexual maturation, neuromotor development, and immunity. In a study by Nepal *et al*. (2014) in eastern Nepal, the highest prevalence of Zn deficiency was observed in the overweight and stunted schoolchildren. Stunting in children is considered as an indirect indicator of zinc nutritional status. Adequate dietary intake of zinc has been shown to exert enriching effects on the skin, and this reduces the likelihood of restricted linear growth in young children (Akhtar, 2013).

**1.5. Iron nutrition**

Dietary iron is a vital nutrient that is required to produce red blood cells such as hemoglobin and myoglobin activities. Hemoglobin is a protein, which carries oxygen from lungs to the tissues. Iron is critical respiration, energy metabolism, proper immune function and cognitive performance. Furthermore, it is part of other proteins and enzymes involved in the synthesis of collagen and neurotransmitters (Mahan *et al*., 2012). Dietary iron may exist as heme iron which is found predominantly in animal foo; non-heme in plants. Iron may be stored within a protein complex called ferritin; transferrin is responsible for transporting iron within the blood to other locations for storage. The main location of iron storage is in the liver, skeletal muscle and reticuloendothelial cells (Mahan *et al*., 2012).

**1.5.1. Prevalence of iron deficiency**

Iron-Deficiency Anaemia occurs when the haemoglobin concentration is abnormally low, due to iron deficiency. This disease occurs when there is a long period of negative balance between the amount of biologically available iron and the need for this trace element (Mahan *et al*., 2012; WHO, 2015; Nobre *et al*., 2017). On the other hand, decreased iron stores (Mahan *et al*., 2012) characterize iron deficiency or iron depletion. Iron status can be determined using haemoglobin, serum ferritin, transferrin receptor and C- reactive protein (Lee and Nieman, 2010).

Iron deficiency anemia is caused by inadequate iron intake, high physiologic demands in early childhood and iron losses from parasitic infections malaria especially. These contribute to the high prevalence of anemia in many populations of low-income and middle-income countries (Akodu *et al*., 2016). Few studies have shown that preschool children in developing countries are at more risk of iron deficiency because of increased requirements during their rapid growth (Balarajan *et al*., 2012; Akodu *et al*., 2016). Iron deficiency is characterized by an imbalance of iron intake, iron absorption and iron loss (Ngui *et al*., 2012). According to Pasricha *et al*. (2010) and Dey *et al*. (2013), anaemia is considered as a proxy indicator of iron deficiency because it is defined as an abnormal iron biochemistry with or without anaemia (Kotecha, 2011). Preschool children are at the stage were their bodies are growing rapidly. This rapid growth requires high iron rich and nutritious food that may not be fulfilled by normal diet (Singh and Patra, 2014).

Nearly one third of the world’s population are estimated to be anemic, despite the recent data indicating a lower global prevalence of anemia in 2010 as compared to 1990 (Kassebaum *et al*., 2014). The global prevalence of anemia (defined as hemoglobin level of <110 g/L) in children, aged 6–59 months, is 43%. Half is attributable to iron deficiency anemia (IDA) which is defined as a hemoglobin level of <110 g/L and ferritin level of <12 μg/L (Habib *et al*., 2016; WHO, 2017). In addition, [Kisiangani](https://www.ncbi.nlm.nih.gov/pubmed/?term=Kisiangani%20I%5BAuthor%5D&cauthor=true&cauthor_uid=26405498) *et al*. (2015) documented that globally, iron deficiency ranks number nine and is responsible for about 60% of all anemia cases among preschool children. A higher prevalence of anemia was reported in Yemen among preschool children (48.7%). Moreover, the prevalence of IDA was 34.2%, which accounted for 70.2% of the anaemic cases in Yemen (Al-Zabedi *et al*., 2014). Most of the burden of IDA is in the resource poor settings of low-income and middle-income countries (Balarajan *et al*., 2012).

In Pakistan, Habib *et al*. (2016) reported that 33.2% of children aged 6–59 months to be iron deficient. In the western province of Kenya, [Kisiangani](https://www.ncbi.nlm.nih.gov/pubmed/?term=Kisiangani%20I%5BAuthor%5D&cauthor=true&cauthor_uid=26405498) *et al*. (2015) documented that iron deficiency was prevalent at 20.8% when low serum ferritin concentration (<12mgl^-1^) was the indicator. In Lagos, Akodu *et al*. (2016) revealed that the prevalence of IDA was 10.1%. This value was lower than the 25.0% reported by Olufemi *et al*. (2013) in four selected local government areas from Ondo State of Nigeria in children, aged 0 to 5 years. The prevalence of anemia among children in Rural Kivu, Democratic Republic of the Congo was 46.6% yet only 16.5% had iron deficiency. However, 4.4% had iron deficiency when using ferritin as the biomarker ([Bahizire](http://www.ajtmh.org/search?value1=Esto+Bahizire&option1=author&noRedirect=true) *et al*., 2017).

According to SANHANES-1 (2013), the prevalence of anaemia in children was 10.5% while iron deficiency was 11% and iron deficiency anaemia was 2.1%. In South Africa, the prevalence of anaemia in children less than five years of age has decreased by approximately *14*% since 2005 (Shisana *et al*., 2013). The prevalence of anaemia (hemoglobin < 11 g/dl) was lower in the SANHANES-1 survey in children under five years of age, as compared to NFCS of 2005 and the SAVACG survey, at 10.7%, 28.9% and 21.4%, respectively (Shisana *et al*., 2013). In the Capricorn District, Limpopo province, Mamabolo and Alberts (2014) reported that the prevalence of anaemia has dropped to 23.6% and the iron deficiency increased to 52.8% at one year of age when compared to 2005 NFCS. In the Vhembe District, Limpopo province Motadi *et al*. (2015) found that 28% of preschool children were anaemic. In addition, the prevalence of iron deficiency was 7.2% when using serum ferritin and even higher (28.1%) when with transferrin saturation as the indicator of iron status.

**1.5.2. Consequences of iron deficiency**

Iron deficiency anemia (IDA) in children adversely impacts child morbidity and mortality and impairs brain development and cognitive development ([Habib](https://www.ncbi.nlm.nih.gov/pubmed/?term=Habib%20MA%5BAuthor%5D&cauthor=true&cauthor_uid=27171139) *et al*., 2016). The negative impacts also may continue into adulthood and lead to low work productivity with effects on the economic productivity. This may trap the communities at risk of infections in a cycle of poverty, underdevelopment and diseases (Al-Zabedi *et al*., 2014; Mamabolo and Alberts, 2014). IDA has been associated with growth retardation, impaired cognition, reduced physical activity and postulated as a contributor to the high national infant mortality rate (Balarajan *et al*., 2011; Soofi *et al*., 2013; Kassebaum *et al*., 2014).

The role of iron deficiency anemia in cognitive impairment and psychomotor development is well also documented (Low *et al*., 2013). Iron deficiency alone, with or without anemia, lead to defects in neurodevelopment and delays in acquisition of motor and mental milestones. These effects may persist into middle childhood ([Kisiangani](https://www.ncbi.nlm.nih.gov/pubmed/?term=Kisiangani%20I%5BAuthor%5D&cauthor=true&cauthor_uid=26405498) *et al*., 2015). In the Capricorn District, Limpopo province, Mamabolo and Alberts (2014) reported the association between anaemia and stunting. The association of anaemia with stunting is easy to understand since the children who consume poor diet over a long period of time may show a deprivation in iron stores (Mamabolo and Alberts, 2014).

**1.6. Consumption of fruits and vegetables by children under the age of 5 years**

Despite several strategies in place to encourage consumption of fruits and vegetables, the prevalence of low vegetable and fruits intake was observed to be 72.2% in South Africa (Vorster *et al*., 2013). In addition, secondary analysis of the National Food Consumption Survey reported a mean fruit and vegetable intake of 180 g/day in children aged 1 to 3 years of age (Vorster *et al*., 2013). Dietary patterns established in the early years of childhood are more likely to remain throughout life, because early exposure to certain foods and flavors has a heavy influence on their acceptance in the short and long-term (Butte *et al*., 2010). Diets of many children in South Africa may be low in essential micronutrients and the contribution of fruits and vegetables nutrients in the diet are low (Vorster *et al*., 2013). Studies have revealed that the intake of fruits and vegetables remain low worldwide, particularly among children (Kim *et al*., 2014; Zalilah *et al*., 2015; Koo *et al*., 2016). According to Anzman-Frasca *et al*. (2012), children do not like to eat vegetables. Holley *et al*. (2017) reported that in the United Kingdom (UK), consumption of vegetables in children has fallen below recommended levels, with less than one in five preschools consuming five portions of fruit or vegetables a day.

According to Krebs-Smith *et al*. (2010), most children do not meet the recommended intakes of fruits and vegetables, despite their known health benefits in America. Children between the ages of 2- to 3-year-old had a total fruit intake below recommended levels while 80% consumed fewer vegetables than recommended levels (Krebs-Smith *et al*., 2010). In addition, Grimm *et al*. (2014) reported that one in three children consumed fruit less than once daily and one in five consumed vegetables less than once daily. In Malaysia, Koo *et al*. (2016) found that fruit and vegetable intakes were far below recommended levels in children. The intake of fruit and vegetable has been reported previously to be related closely to sociodemographic characteristics in developing countries (di Noia *et al*., 2014). In Brazil less than 50% of the children were observed to consume fruit daily, and only 12% consumed leafy vegetables (Bortolini *et al*., 2012).

**1.6.1 Fruits and vegetables as sources of iron and zinc**

Eating a variety of foods can help to meet daily requirements of minerals. Iron and zinc are vital minerals needed in children for proper growth and development, and cognitive function. Iron and zinc occur in a wide variety of vegetables (particularly dark green leafy vegetables, *amaranthus, Cleome, Biden’s pilosa*, lentils, peas and beans) and fruits such as avocado. Dried beans and vegetables (such as spinach, broccoli, sunflower seeds, and raisins) are sources of non-heme iron, which is more difficult to absorb than heme iron (Marles, 2017). To increase the absorption of non-heme iron, food sources of vitamin C, tomatoes and orange should be consumed.

Chickpeas and sunflowers are known to be among the best sources of inorganic zinc (Mahan *et al*., 2012). Mavengahama *et al*. (2013) reported that indigenous vegetables are rich sources of micronutrients, especially beta-carotene, zinc and iron. These indigenous vegetables are important for poor people as they are not purchased and contain a variety of micronutrients (Lewu and Mavengahama 2010; Kunyanga *et al*., 2013). Table 1 below lists selected fruits and vegetables known to contain iron and zinc.

**Table 1.1: Vegetables and fruits that contain iron and zinc**

| **Fruits and vegetables** | **Iron (mg)/100g** | **Zinc (mg)/100g** |
| --- | --- | --- |
| Spinach cooked | 6.43 |  |
| Potato skin | 4.08 |  |
| Baked beans (1 cup) | 8.2 | 1.7 |
| Soybean | 10.4 |  |
| Chickpeas |  | 1.3 |
| Kidney beans |  | 0.8 |
| Broccoli | 0.64 |  |
| Lentils | 3.3 |  |
| Raisins | 7.7 |  |
| Fruits |  | 1.6 |
| *Pumpkin leaves* | 50.80 | 5.10 |
| *Amaranth cruentus* | 0.3-3.8 | 0.02-8.4 |
| *Amaranth thunbergii* | 0.3-3.8 | 0.02-8.4 |
| *Amaranth hybrdus* | 21 | 18 |
| *Amaranth dubius* | 25 | *13* |
| *Amaranth spinosus* | 32 | 1 |
| *Amaranth grain* | 8.70 | 2.85 |
| *Cleome gynandra* | 2.6-2.9 | 0.6-0.8 |
| *Cleome monophylla* | 24 | 5 |
| *Bidens pilosa* | 17 | 22 |

Source: (Uvsiku *et al*., 2010; Mahan *et al*., 2012; Kunyanga *et al*., 2013).

**1.7. *Cucurbita Moschata* (Butternut) seeds.**

*Cucurbita moschata* seeds, also known as butternut seeds are white or brown in colour and are consumed directly by humans as a snack (Karanja *et al*., 2013). Farmers in Africa have cultivated and collected these vegetables and their seeds as an important food source for generations (Aruah *et al*., 2011). According to Cortez-Vega *et al*. (2014), *Cucurbita moschata* is a common seasonal crop that has been used traditionally both as food for humans and animals. In South Africa *Cucurbita Moschata* vegetables are usually grown in the Spring and Summer when there is sufficient rainfall (Shaffer, 2010). Many rural communities have been consuming traditional vegetables and their seeds for centuries. These have contributed immensely to household food security by providing direct access to readily accessible nutrients (Shaffer, 2010; Mpala *et al*., 2013). Currently, *Cucurbita moschata* seeds are discarded as by-products in food processing. Although *Cucurbita moschata* is grown locally in South Africa, the seeds are discarded as waste despite their great nutritional potential. After removing the flesh, in many communities the best seeds are set aside for cultivation the next season.

*Cucurbita moschata* seeds contain a rich range of nutrients such as selenium, iron, zinc, manganese, phosphorus and other important micronutrients (Karanja *et al*., 2013; Patela and Raufb, 2017). Seeds are important sources of protein, fats, carbohydrates and other minerals (Kim *et al*., 2012; Patel, 2013). According to Kim *et al*. (2012), seeds are a good source of zinc and polyunsaturated fatty acids. These seeds have numerous health benefits such as lowering cholesterol and are good source of protein, zinc, magnesium, manganese, phosphorus and phytosterols (Patel, 2013). Nwofia *et al*. (2012) reported that the seeds could be used as valuable food supplement due to their richness in magnesium, phosphorus, calcium, manganese, copper and zinc. In addition, Kanwal *et al*., (2015) revealed that seeds are good sources of fat, protein, carbohydrate and minerals. The use of indigenous vegetables, fruits and their seeds like those of *Cucurbita moschata* should be one of the remedies to counter micronutrient deficiencies (Echessa *et al.,* 2013). One serving (about ¼ cup) of *Cucurbita moschata* seed provide almost half the recommended daily amounts of manganese, magnesium, phosphorus, iron, copper, vitamin K and zinc (<http://www.squidoo.com/pepitas>). Table 1.2 below illustrate the levels of micronutrients in *Cucurbita moschata* seeds, determined in Kenya. No data is available for South Africa but for other countries (Nigeria, Mofunaya & Edu, 2015; Pakistan, Hussain *et al*., 2011).

**Table 1.2: Levels of micronutrients in *Cucurbita moschata* seeds**

| **Micronutrients** | **Mg/100g** |
| --- | --- |
| Vitamin C | 2.94 |
| β-Carotene | 0.27 |
| Thiamine (B_1_) | 0.22 |
| Riboflavin (B_2_) | 0.35 |
| Niacin (B_3_) | 0.82 |
| Pyridoxine (B_6_) | 0.14 |
| α-Tocopherol | 6.64 |
| Iron | 9.70 |
| Zinc | 9.70 |
| Selenium | 6.46µg/100g |

Source: (Echessa *et al*., 2013)

Increasing the availability of these traditional vegetables and their seeds to low income communities in rural areas offers a unique opportunity to provide affordable nutrition to avert malnutrition and micronutrient malnutrition (Mpala *et al*., 2013). The incorporation of traditional vegetables in the diet has the potential to reduce poverty and nutritional security. These foods are easily available, inexpensive, easy to grow, rich in vitamins and minerals, and phytochemicals, with anti-oxidants properties (Mpala *et al*., 2013). *Cucurbita Moschata* seeds and the oil also are commonly consumed in some countries (Kim *et al*., 2012). Across South America, Africa and the Middle East the seeds are used extensively in cooking, roasted, salted, and served as snack (Shaffer, 2010; Aruah *et al*., 2011).

Kwiri *et al*. (2014) reported that when the seeds are cooked, toasted/roasted and dried, they could be served as snacks and made into a paste that resembles peanut butter. The *Cucurbita Moschata* seeds are widely distributed and can be easily cultivated and consumed because they are less expensive (Kwiri *et al*., 2014). Consumption of nutritive local foodstuffs will help to supplement the nutrients of the staple carbohydrate foods of the poor who cannot afford enough protein foods of animal origin (Achu, 2013). Moreover, *Cucurbita Moschata* is widely grown in South Africa.

The most frequently consumed *Cucurbita* species in South Africa is *Cucurbita Moschata*. Studies have shown that the seeds of *Cucurbita* *spp* are consumed all over the world. However, at the domestic scale they are consumed raw, roasted or cooked (Aruah *et al*., 2011; Patel, 2013). Several studies have dealt with the benefits of consuming *Cucurbita Moschata* flesh and leaves (Yadav *et al*., 2010; [Kim](https://www.ncbi.nlm.nih.gov/pubmed/?term=Kim%20MY%5BAuthor%5D&cauthor=true&cauthor_uid=22413037) *et al*., 2012; Wang *et al*., 2012, Durante *et al*., 2014; Dias *et al*. 2015). Yet limited studies exist on the beneficial effects of consuming *Cucurbita moschata (CM)* seeds. The present study is, therefore, designed to evaluate effectiveness of *CM* (Butternut) seeds consumption in the management of iron and zinc deficiencies in preschool children, aged 3-5 years.

***1.7.1 Microbial and safety issues***

Controlling the development of microorganism that cause pathogens in food is vital for the health of the public, especially for a high-risk population such as children (Dolye and Glass, 2010). Food spoilage, pathogenic or usefulness are categorized and associated with microorganisms. Spoilage of food occur when microorganisms grow in food and cause undesirable changes in aesthetic factors such as flavor, texture, colour, and appearance (Rawat, 2015). Bacterial enzymes cause slow deterioration of frozen or dried foods when stored for a long time diminishing the quality of foods, this renders them unfit for human consumption due to changes in sensory characteristics (Rawat, 2015). Fruits and vegetables are susceptible to microbial spoilage produced by fungi, bacteria, yeast and moulds. Traditionally, salt is known to limit the growth of bacteria or microorganisms that cause spoilage and increase the shelf life of foods (Doyle and Glass, 2010). Due to the antimicrobial and antifungal activity contained in spices from their natural constituent, spices can affect the food spoilage microorganisms and human pathogens (Gottardi *et al*., 2016). Thus, *Cucurbita moschata* dishes are cooked with salt and herbs (spices) in order to limit the growth of pathogens. The addition of salt on the *Cucurbita moschata* seeds will be guided by the recommended daily intake of salt according to South African Food Based Dietary Guidelines (FBDGs) (Voster *et al*., 2013).

**1.8. Early Childhood Development centres as a point for lifestyle interventions**

The transition of introducing complementary food is accompanied often by micronutrient deficiencies in developing countries. Meals served outside home like school lunches offers one opportunity to increase micronutrients intake of preschool children (Bailey *et al*., 2015). A food-based approach can include addition or changes to complementary feeding practices when introducing solid and liquid nourishment (Bailey *et al*., 2015). Early childhood development centres are an appropriate setting to provide health and nutrition services, these can help to rectify micronutrient deficiencies at an early stage of life to disadvantaged children (Best *et al*., 2010). In addition, the effects of micronutrient deficiencies in children are severe and may affect the linear growth, cognition and muscle development; these often are irreversible during childhood (Hettiarachchi and Liyanage, 2012).

Early childhood development centres are a practical platform to deliver an integrated package of interventions. These include nutritious meals or snacks, micronutrient supplements or on-site fortification, infection control, health promotion, and life-skills education. Collectively these can improve the health and correct micronutrient deficiencies of children (Best *et al*., 2010). Most micronutrient deficiency disorders can be reversed with the provision of missing micronutrients at an early age. Yet some deficiency disorders result in irreversible, lifelong consequences if they remain untreated. The severity, timing, and the extent of the deficiency will determine the extent at which they can be reversed (Bailey *et al*., 2015). The impact that micronutrient deficiencies have across the lifespan is not surprising because they cause tremendous financial burdens to societies. Early-life nutrition has long-lasting impacts on the individual and society, including poorer children’s health, less educational attainment, diminished work capacity, and lower lifetime earning potential (Black *et al*., 2013).

***1.8.1. Early Child Development Policy***

The new National Integrated Early Childhood Development (ECD) Policy facilitates an inclusive package of service for young children. It priorities the delivery of vital components of the package (Hall *et al*., 2016). The package is comprised of services that are vital for the realisation of the child’s constitutional rights. Insufficient quality of nutrition may lead to poor health and educational outcomes for children, these can contribute to persistent inequalities. One of the packages of service in the national integrated early childhood development policy include nutritional support for children under the age of five.

In order to support nutrition at the ECD centres, the Department of Health developed nutrition guidelines in 2016. These guidelines provide ECD practitioners and caregivers with information on how to plan, prepare and serve appropriate, nutritious, adequate and safe foods to children in their care (DoH, 2016). ECD guidelines further serve as a guide for practitioners and caregivers on how complementary food can be introduced to children from 6 months to 6 years. Guidelines on nutrition for Early Childhood Development centres encourage children to follow a healthy eating plan by enjoying a variety of foods with suitable snacks and drinks (DoH, 2016). According to the Department of Health (2016), locally available foods can be used interchangeably with indigenous food to ensure inclusion of a variety of foods from all food groups. Food/snacks that do not provide good nutrition should not be included during mealtime for children 12 months to 6 years. Early childhood is the critical stage where children learn basics of healthy eating and to enjoy a healthy eating plan.

**1.9 Problem statement**

It has been estimated that zinc deficiency affects about one-third of the world’s population, with a prevalence of 4% to 73% across sub regions in Sub Saharan Africa. This degree of inadequacy is shocking when the requirements of iron and zinc are lacking. Growth is impeded, immune function is depressed, hair is lost, and susceptibility to and severity of infections is increased. In addition, neurobehavioral abnormalities occur due to increased number of opiate receptors in the hypothalamus, in parallel with decreased beta-endorphin and increase metenkephalin (Mahan *et al*., 2012). Iron deficiency and iron deficiency anaemia are two of the most common nutritional deficiencies among children in the world (Monárrez-Espino *et al*., 2011; Mahan *et al*., 2012). The WHO estimates that iron deficiency anaemia affects quarter of the world population and is more prevalent in pre-school children, particularly Africa. Furthermore, the deficiency of these micronutrients is caused by consumption of staple foods and cereal crops that have low micronutrient bioavailability (Uchendu, 2011).

*Cucurbita moschata* (butternut) seeds are considered generally to be agro-industrial wastes and are discarded (Patel, 2013; Patela and Raufb, 2017). In many communities around the world, the seeds are consumed raw, roasted or cooked, but only at the domestic scale. In addition, many communities in South Africa use to consume the roasted *Cucurbita moschata* seeds with porridge. Consumption has been associated with poverty. Instead of being used as a food source, these are discarded or preserved for future use. *Cucurbita moschata* contains adequate levels of minerals such as iron, zinc, copper, calcium, potassium and phosphorus (Patel, 2013; Patela and Raufb, 2017). Despite the legislated fortification of staple foods in South Africa since 2003 (SANHANES-1, 2013), there is a general shortage of micronutrients in a large part of the diet of the population. Inadequate micronutrient intake also has been identified among segments of infant’s in South Africa due to improper practices of feeding children.

Food-based approaches encourages the consumption of foods that are naturally rich in micronutrients; this approach favour locally available foods and considers local, culturally acceptance food habits. This approach recognizes the nutritional value of food for good nutrition by encouraging the enjoyment of variety of foods and supporting rural livelihoods (Darnton-Hill and Cogill, 2010). According to Gibson (2011), the approach equips people to consider their total diet in relation to their preferences, individual lifestyle factors, physiological requirements and physical activity. Neumann *et al*. (2011) revealed that food-based strategies are encouraged as safe methods for controlling and preventing micronutrient deficiencies (Kalimbira *et al*., 2010). Success of a food-based programme for the very poor occurs when each member in the household has access to adequate dietary intake that is optimal for maintaining micronutrient status (Arimond *et al*., 2011; Gibson, 2011).

Infections in children is caused by group of parasites commonly known as worms such as hookworms, whipworms and roundworms. These infections are soil transmitted helminth infections (WHO, 2017). Those living in poverty are most vulnerable to these infections which can impair their nutritional status causing diarrhoea, impairment of nutrient intake, digestion and absorption, intestinal inflammation and obstruction, internal bleeding which can lead to loss of iron and anaemia (Welch *et al.,* 2017).

According to WHO (2017) deworming is the best strategy to reduce worm burden and weight and height gain, morbidity. WHO recommend single dose of albendazole (400mg) or mebendazole (500mg) for children aged 12 to 23, preschool children aged 1-4 years of age, and school age children 5-12 years of age living in area where the baseline prevalence of soil transmitted infection is 20% or more among children (WHO, 2017).

This target group for this study is selected, as children under five years constitute the most vulnerable group of a community. Their nutritional status is a sensitive indicator of community health and nutrition. Furthermore, correcting deficiency of micronutrients can help reduce child mortality.

**2. Motivation for the study**

A study by Motadi *et al* (2015) in the Vhembe district in Limpopo province indicated that the prevalence of zinc deficiency among children, aged 3 to 5 years, was 42.6% and iron deficiency anaemia was 28%. These data reveal that two micronutrients are significant public health problems in this area of South Africa**.** This proposed study is a continuation from my master’s work. It aims to utilize a food-based approach to address micronutrient deficiencies observed by promoting neglected or underutilized crops such as *Cucurbita Moschata* seeds. In South Africa, *Cucurbita Moschata* is a vegetable grown for its leaves, flesh and flowers. At present the seeds are discarded mainly as a waste (Lewu and Mavengahama, 2010; Dweba and Mearns, 2011) while consuming leaves, flesh and flowers. Yet this practice may be an inadvertent waste of a precious nutritional source of essential nutrients.

These seeds are known to contain numerous vitamins and minerals such as vitamins A and C, zinc, iron, magnesium, and potassium, all of these are needed for resistance against infections (Dweba and Mearns, 2011; Patela and Raufb, 2017). The World Health Organization recommends that the consumption of these seeds may an excellent source of these nutrients (WHO, 2002; WHO/WFP/UNICEF, 2008; http://www.whfoods.com/genpage). Furthermore, Nowfia *et al*. (2012) reported that the seeds could be used as a valuable food supplement.

**2.1. Research Question**

Can the consumption of *Cucurbita moschata* (Butternut) seed paste improve the zinc and iron status of preschool children?

**2.2. Hypothesis**

A null and an alternate hypothesis have been determined.

**Null hypothesis:** Consumption of *Cucurbita moschata* seed paste does not improves zinc and iron status in deficient children.

**Alternative hypothesis:** Consumption of *Cucurbita moschata* seed paste improves zinc and iron status in deficient children.

**2.3. Main aim of the study**

The primary aim of this study is to test the efficacy of consumption of *Cucurbita moschata* (Butternut) seeds in improving zinc and iron status in preschool children in the Vhembe district, Limpopo province of South Africa, and the secondary aim is to describe the nutritional, zinc and iron status of the children. To attain the aims of this study the following objectives have been developed. Objectives of this study are outlined in each phase

**2.5. Expected outcomes**

- Zinc and iron status of the intervention group will improve from baseline levels after consumption of the seeds for 6 months.
- Eating patterns of the participants will improve because of the nutrition education.
- Consumption of fruits and vegetables will increase because of the nutrition education.

**2.6. Significance of the study**

South Africa is undergoing a rapid transition from a diet high in fibre rich carbohydrates to a diet high in fat, animal-source foods, sugar and other refined (low fibre) carbohydrates. with the existence of multiple diseases such as chronic diseases and micronutrients deficiencies. Several studies have observed low consumption of micronutrients (iron and zinc) rich foods among children. Promotion and consumption of underutilized or forgotten crops (seeds) will help address the deficiency of these micronutrients, as it is both a short and long-term strategy. This research will help communities, especially in rural areas, to understand the significance of consuming these seeds, rather than discarding them as waste or storage for future cultivation. The current South African Food Based Dietary Guidelines for children between the ages of 3 to 5 years encourage feeding children regular, small meals and healthy snacks. The *Cucurbita Moschata* seeds can be eaten raw or roasted as a snack (Karanja *et al*., 2013). Many communities have access to these seeds, which are rich in minerals, vitamins, phytochemicals and antioxidants. This study seeks to develop a model to promote this underutilized or forgotten crop (seeds) and encourage communities to include these seeds in their daily meal as a snack. It is important to conduct this study to promote the value of this seeds.

1. **METHODOLOGY**

**3.1. Study design**

The study will use mixed methods depending on the phase of the study. This will include experimental, observation, qualitative, cross-sectional survey and a pretest-posttest control group trial. The designs are explained in detail under each phase, see figure below for sequencing of the phases.

**Figure 2: An illustration of the methodological sequencing of the phases**

**3.2. Target population:**

Preschool children, aged 3-5 years, in the Vhembe district of South Africa will be the participants in this study. This target group was selected because children under five years constitute the most vulnerable group of community and their nutritional status is a sensitive indicator of community health and nutrition (Ibeanu *et al.,* 2012). Furthermore, pre-school children are at an important stage of life where nutrition plays an important role in growth and development and has long lasting effects on health in later life (Khan Khattak and Ali, 2010).

**3.3. Location of study:**

The study will be conducted at registered government preschools in the Vhembe district of Limpopo province in South Africa. Vhembe district is one of five districts of Limpopo province, and is divided into four municipalities namely Musina**,** Thulamela**,** Makhado and Collins Chabane (Lim 345). The Vhembe district has a total population of 1,294,722, with 703,226 (53.3%) females and 591,496 (46.7%) males, 1*14* 984 (12.7%) children under five years. Furthermore, 67.2% of this population speaks *Tshivenda,* 24.8% *Xitsonga*, 1.3% Afrikaans, and 1.6% *Sesotho sa Leboa*. The District has very high unemployment rate of almost 24% (Stats SA, 2011). The total number of pre-schools (ECDs) registered under Department of Social Development in the government are 730 in 2018. The total number of children in preschools around the Vhembe district is 40291 (Department of Social Development, 2018). The researcher, Motadi *et al*. (2015) previously reported 42.6% zinc prevalence and 28% iron prevalence in preschools in Vhembe district.

**3.4. Sampling**

Musina municipality will be purposefully selected from Vhembe District. These are in geographic proximity of the University of Venda where researcher is located and where the previous study was conducted (Motadi *et al*., 2015). Simple random sampling will be used to select six preschools, three each from the two local municipalities. Simple random sampling is a sampling procedure, which provides equal opportunity of selection for each element in a population (Lee and Nieman, 2010). A list of pre-schools will be obtained from the Department of Social Development in Vhembe district and each pre-school will be assigned a number. The researcher will randomly select one number at a time. The following Slovin formula is used to calculate the total sample size used in the study: n = N/ (1 + (N X e^2^)) where

n= sample size

N= total number of children in the pre-schools; and

e = the accepted level of error

n = N/ (1 + (N X e^2^))

=40291/ (1+(40291 x 0.05)

=40291/ 101.7275.

= 396

= plus 10%

= 435.6

= 436

The formula yielded 396 participants, and an additional 10% will be added to account for attrition, making 436. Total sample size was calculated from the total number of children registered in the Early Childhood Development under Social Development in South Africa in 2017 in Vhembe district (Government Gazette, 2017). The sample frame will be six preschools, which are yet to be randomly selected. If the total number of participants in these six selected preschools does not reach the total number of 436, two more preschools will be added to the sample frame. The six preschools will be randomly assigned to the treatment or control.

**3.5. Inclusion**

Children who are 3-5 years old (parents consented) and present on the day of data collection will be included in the study. Children who are free from infections (such as malaria, and tuberculosis) and taking any medication (such as antacids, famotidine and ranitidine, omeprazole, pantoprazole, proton pump inhibitors and calcium supplements) will be included in this study because medication alter the serum concentration and level. Mothers/guardian will be requested to record the health condition suffered by the child in the past 15 days (**see appendix B**)

**3.5.1 Exclusion criteria**

Children whose parents/guardians did not give consent will be excluded from the study. Children with physical disabilities that would hinder taking anthropometric measurements will be excluded because of lack of equipment to measure height for people with disability.

***3.6. DATA COLLECTION PROCEDURE***

The study will be conducted in four phases: phase one: development of intervention, phase two: baseline survey, phase three: implementation of the Intervention, and phase four: post intervention survey as depicted in Figure 1. The sections below provide details for each phase.

**3.6.1.** **PHASE ONE: DEVELOPMENT AND PILOTING OF THE INTERVENTION**

**3.6.1.1. Aim:** To develop the intervention consisting of *Cucurbita Moschata* snack and nutrition education.

**3.6.1.2. Objectives**

3.6.1.2.1. Develop *Cucurbita moschata* dish.

3.6.1.2.2. Develop a nutrition education lesson on the importance of *Cucurbita moschata seeds.*

3.6.1.2.3. Testing the microbial activity, toxicity level, micronutrient content (bioavailability of Fe, and Zn), shelve life, nutrient stability and the presence of iron and zinc inhibiters using standardized methods.

**3.6.1.3. Study design**

The design for this phase will be experimental and observation for the development of recipes and a qualitative approach for development of the song, following Homophonic parallelism (good lyrics, harmonies and melodies).

**3.6.1.4. Recipe Development and Preparation**

Four (4) recipes will be developed during using standardization cycle adopted from Spears and Gregoire (2010). The steps to be followed will be recipe formulation, product evaluation, quantity adjustment, and sensory evaluation. The process is illustrated below.

**Figure 3:** Recipe Standardization Cycle Adopted from (Spears & Gregoire, 2010)

The recipe development and sensory evaluation will be conducted in the food science laboratory at the University of Venda. The sensory evaluation will help standardize the recipes. This will be followed by testing the microbial activity in a microbiology laboratory at the University of Venda. In addition, toxicity, micronutrient content, life span and nutrient stability will be tested in a food science laboratory. The presence of iron and zinc inhibiters in the recipes will be evaluated in the food science laboratory at the CSIR and Central Analytical Facilities (CAF) in Stellenbosch University using standardized methods.

The recipes will be tested for total plate count microbial activity in accordance with codex principles of Hazard Analysis Critical Control Point (HACCP). Recipes will be examined for a specific pathogen or its toxins (such as coliform- for hygiene, yeast, moulds and fungi) (Khalaf and Raizada, 2016). This examination will detect organisms that may be indicative of the possible presence of pathogens or to detect the presence of specific spoilage organisms. Testing for microbial activity of the recipes is vital to assure that critical raw materials are satisfactory for their intended use.

***3.6.1.5. Cucurbita moschata seeds dishes***
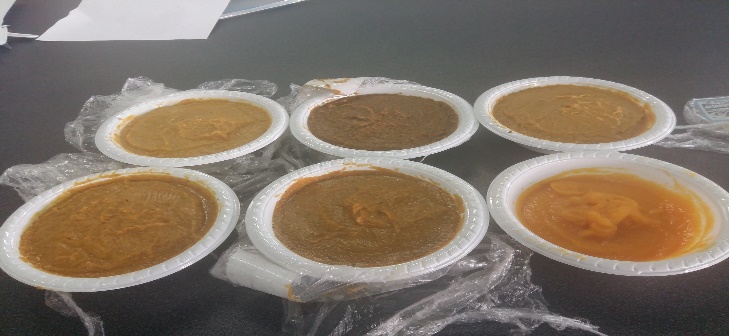


***(a) Grounded Cucurbita moschata seeds mixed cooked flesh and sugar***

After the seeds are removed from *Cucurbita moschata,* they will be rinsed with water to remove any strings and bits of squash. The seeds will be patted dry and placed in a small bowl. Once the seeds are dried they will be grounded in a machine until they form a powder*. The skin of Cucurbita moschata* will be peeled off and the flesh will be chopped into cubes, chopped flesh of *Cucurbita moschata* will be cooked with 2 cups of water and 30g of sugar. The cooked *Cucurbita moschata* will be mixed with the powder (grounded seeds) to make a paste.

**(b)** ***Paste mixed with cinnamon***

The cooked *Cucurbita moschata* will be mixed with the powder (grounded seeds) to make a paste. Cinnamon will be added to paste to enhance flavour and taste.

**(c) *Paste mixed with vanilla essence and honey.***

Vanilla essence and honey in the food increase the appearance, flavour and taste. The same procedures as in (a) will be followed, except that the sugar will be replaced with honey.

**(d) *Paste cooked with butter***

Butter also known to add flavour and taste to food. Fats assist in the absorption of vitamins. Same procedure as in (a) above.

**(e) Sensory evaluation procedure using hedonic scale**

Children below five years are not considered able to express differences in taste perception by use of the preferential methods. A child’s own spontaneous verbal judgement will be used to estimate the difference; preference and acceptability of the recipe using the hedonic scale **(see Appendix A).** The children will select the most liked or preferred dish from the four dishes, then the researcher will rank them accordingly according to “most liked by children”. Two will be used for this intervention to allow for variety.

**3.6.1.6.** **Development of the nutrition education song on the consumption of *Cucurbita moschata***

The design will follow Homophonic parallelism (Boamah, 2012) in composing the song. Harmonic parallelism is the harmonizing of a single melody or subordinate melody and moving with it in parallel. This means the notes that harmonize the melody follow its characteristic shape and rhythm and this type of parallelism is common to all African peoples. The researcher will lead the composition of the song along with the children during piloting.

The lyrics as spelled out below have been determined. The lyrics will be repeated in three languages (English, *Xitsonga* and *Tshivenda)*

1. *We love butternut x2 (Chorus) 3. Ri funa mafhuri (Chorus)*

*We eat seeds x2 Rila na thanga x2*

*And we get zinc, iron dzi rinea pfushi x2*

*We eat seeds Rila thanga x2*

*We eat seeds.*

1. *Hi randza makwembe x2 (Chorus)*

*Ma kahle ka hina x2*

*Hi dya tinghwembe ta kona hi kula*

*Hi kula kahle*

*Hi van a matimba x2*

1. *Thanga dza fhuri (Chorus)*

*ndi zwavhudi u dzila x2*

*dzi rinea pfushi x2*

*kha dzi liwe x2*

Harmonies and rhythms will be tested and practised with the children until an acceptable tune is reached. The acceptable rhythm will be recorded, so that it can be used in the preschools during the intervention. Six to ten tapes will be made for each preschool and to keep extras in the event of damage or loss.

**3.6.1.7.** **Piloting of the intervention and training package.**

The song and the recipes will be pre-tested and piloted using the University of Venda preschool children for a week. The participants will form part of informal sensory evaluation panel during the recipe standardization. The song also will be tested with the children. Permission to use the preschool will be sought from the University and the parents, mostly University employees.

**3.6.1.8. Outcome of phase one**

The outcome will be developed recipes with microbial activity, toxicity level, micronutrient content analysis and identified inhibitors. Two most liked recipes will be selected. The dosage of the Zn and Fe will be determined as well as the portion size and the frequency of supplementation required. The song is composed and recorded for use later.

**3.6.2. PHASE TWO: BASELINE SURVEY.**

**3.6.2.1. Aim:** The aim will be to recruit, and screen potential participants and collect baseline data.

**3.6.2.2. Objectives:**

3.6.2.2.1. To determine the demographic and environmental factors of the participants.

3.6.2.2.2. To measure the anthropometry of the study participants (Weight, Height, MUAC)

3.6.2.2.3. Determine the dietary diversity scores of participants using 24- hour recall

- - - - 1. Determine dietary patterns of the study participants using FFQ.
        2. To determine serum zinc concentrations among pre-school children using flame atomic absorption spectrophotometer before consuming *Cucurbita moschata* seeds.
        3. To determine the iron status of pre-school children using hemoglobin, serum ferritin, transferrin receptor and C- reactive protein (CRP) before consuming the *Cucurbita moschata* (butternut) seeds.

**3.6.2.3. Design:** The study design will be a cross sectional survey in which the researcher will measure the demographic and environmental factors, anthropometries, diet patterns, zinc and iron status of pre-school children. A cross sectional study is a method that involve observation of a population or a representative subset at one specific point in time (Leedy and Ormrod, 2010). All measurements parameters will be done at one point using a quantitative approach.

**3.6.2.4. Participants recruitment**

The researcher will recruit study participants from the randomly selected preschools form the two purposefully selected municipalities of the Vhembe district.

***Visit 1:***

A list of pre-schools will be obtained from the district office and preschools from Musina and Collins Chabane will be randomly selected. The selected preschools will be visited, and the research aims, and objectives will be explained to the headmaster. In addition, permission to include pre-schools in the sample will be requested.

***Visit 2:***

A parents’ meeting will be arranged. The researcher will provide explanation to parents who need clarity before giving consent for children to participate in the study. A list of children whose parents will consent will be generated.

**Visit 3 and 4**

Arrangements for data collection will be made with the pre-school management. A full day or two will be required for each preschool and space for data collection will be requested form the preschool management.

The baseline survey will thus require four visits per preschool (visit 1: 2 days; visit 2: 6 days; visit 3 and 4: 12 days) for a total of 20 days.

**3.6.2.5. Measurements**

The following measurements will be carried out during the baseline survey: demographic and environmental factors, anthropometrics, dietary intakes and patterns, and biochemical parameter.

1. ***Physical arrangements***

A separate classroom will be requested to be used to ensure privacy for the children and their parents. Two stations will be arranged; (1) measurements of anthropometrics and interviews, and (2) collection of blood samples.

1. ***Demographic and environmental factors***

In this study, demographic factors will be collected, such as age, gender and socioeconomic status. Environmental factors will include the abiotic (temperature, amount of sunlight, and pH of the water) and the biotic (disease, competition, parasitism, and dietary consumption). A validated questionnaire will be used (**see Appendix B**). Since children are under five, mothers will be requested to provide this information.

1. ***Anthropometrics measurements***

Anthropometrics will include weight, height and mid upper arm circumference will be used to assess nutritional status. The weight and height will be taken twice, and average computed to reduce error.

***Weight***

Children will be weighed without shoes and wearing light clothes, standing still in the middle of the platform of the scale without touching anything. Body weight will be equally distributed on both feet. Weights will be taken by a registered nutritionist using a (Seca solar scale model 0213). The Seca solar scale will be calibrated against an electronic scale. Weight measurements will be taken twice, and the average will be calculated. Weight will be recorded to the nearest 0.1 kg (Lee and Nieman, 2010). A solar scale will be used because some pre-schools do not have electricity and **Appendix B** will be used for recording.

***Height***

Height will be measured using a stadiometer (Seca model 0123). The participants will be barefoot, standing with heels close together, arms to side, legs straight and knees together, looking straight ahead and wearing minimal clothing. The participants will stand with shoulders relaxed and looking straight ahead. Heels, buttocks, scapular (shoulder blade) and back of the head will be against the vertical surface in a Frankfurt position (Lee and Nieman, 2010). Height measurements will be taken twice, and the average will be calculated. Height will be recorded in centimetres to the nearest 0.1 cm (Lee and Nieman, 2010) (**see Appendix B**) for recording.

***Mid Upper Arm Circumference***


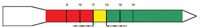


***Figure 4: MUAC measuring tape.***

The MUAC is the circumference of the left upper arm that is measured at the mid-point between the tips of the shoulder and elbow. The participant’s left arm will be bent then the researcher will find and mark the mid-point between the two marks. There arm will be hanging straight down, and a MUAC tape will be wrapped around the arm at the midpoint mark. The MUAC will be measured to the nearest 1m (**see Appendix B**) for recording.

1. ***Dietary intake and patterns***

A one day 24-hour recall will be used to collect detailed information about all foods and beverages consumed using the multiple pass method (Steinfeldt *et al*., 2013). Foods will have been consumed in the past 24 hours, most commonly, from midnight to midnight the previous day (**see Appendix C)**. Six daily intervals will be included, namely: breakfast (period up to 09h00), In-between snack (between 09h00 and 12h00), lunch (12h00 to 14h00), In-between snack (14h00 to 18h00), dinner (18h00 to 20h00), and after dinner (after 20h00). Guardian/parents will provide this information. A FFQ will be used to establish food consumption patterns including the *Cucurbita moschata* seeds. A selected pictorial FFQ will be designed for the children to establish if they know and consume the seeds (**see Appendix D**). Preschool menu will also be evaluated at this stage using the 24 hr recall form for recording at least once a week during the implementation of the intervention.

1. ***Biochemical measurements***

Zinc status will be determined by serum zinc concentrations and iron status via haemoglobin, serum ferritin, transferrin receptor and C- reactive protein (CRP) among pre-school children using flame atomic absorption spectrophotometer. The anthropometric status of the preschool children will be assessed by measuring weight and height to calculate the body mass index (BMI).

***Blood collection***

The children will be given single-dose of albendazole (400 mg) or mebendazole (500 mg) for deworming at this phase. A phlebotomist will collect 5ml blood samples from the participants. The participant’s skin will be cleaned with cotton wool and 70% alcohol at the site of the antecubital vein and the participants’ arm will be restricted with a tourniquet for < 1 minute. A trace element-free, siliconized 23-G needle will be inserted into an antecubital vein, and 5ml blood drawn into two trace element-free evacuated tubes. These will be centrifuged at room temperature for 10 min at 2000 × g and divided into aliquots to separate serum from the blood at the preschool site. Serum will be transferred into new tubes for storage in a cooler box with crushed ice prior to being frozen at −20°C until further analysed. Analysis will be conducted subsequently at Ampath laboratories in Polokwane, South Africa. routine quality control measures will be employed.

**Zinc parameter analysis (provided by Ampath)**

A 125I-radioimmunoassay (Ramco Laboratories, Inc., Stafford, TX, USA) will be used for the quantitative determination of zinc in serum.

**Iron parameter analysis (provided by Ampath)**

The following blood constituents will be analysed: haemoglobin, serum ferritin, C-reactive protein and transferrin saturation.

***Haemoglobin***

A full blood count will be used to evaluate overall health and detect a wide range of disorders, including anemia and infection in preschool children. Purple (Lavender) tube which will be used for full blood count. About 1ml of blood is sufficient to do a full blood count.

***Serum Ferritin***

Ferritin will be assed is a two–site immunoenzymatic (“sandwich”) assay (Ramco Laboratories, Inc., Stafford, TX, USA). A blood sample will be added to a reaction vessel with goat anti-ferritin-alkaline conjugate, and paramagnetic particles coated with goat anti-mouse: mouse anti-ferritin complexes. The Access Ferritin assay is a two-site immunoenzymatic (sandwich) assay. A sample will be added to a reaction vessel with goat anti-ferritin-alkaline phosphate conjugate, and paramagnetic particles coated with goat anti-mouse: mouse anti-ferritin complexes. Serum or plasma (heparin) ferritin binds to the immobilized monoclonal anti-ferritin on the solid phase, while the goat anti-ferritin enzyme conjugate reacts with different antigenic sites on the ferritin molecules. After incubation in a reaction vessel, materials bound to the solid phase will be held in a magnetic field while unbound materials are washed away. Then, the chemiluminescent substrate Lumi-Phos 530 will be added to the vessel and light generated by the reaction will be measured with a luminometer (Ramco Laboratories, Inc., Stafford, TX, USA). The light production is directly proportional to the concentration of ferritin in the sample. The amount of analytic will be the sample will be determined from a sorted, multi-point calibration curve.

***C-reactive protein and transferrin***

The C-reactive protein and transferrin will be assessed with the SYNCHRON LX® System(s), UniCel® DxC 600/800 System(s) and SYNCHRON® Systems CAL 5 Plus respectively (Human Biochemical and Diagnostic Laboratories, South Africa). CRP and transferrin reagents will be used to measure C-reactive protein and Transferrin concentration by a turbidimetric method (Ramco Laboratories, Inc., Stafford, TX, USA).

***Transferrin saturation***

TRFN reagent is used to measure the TRFN concentration by a turbidimetric method.1,2 In the reaction, TRFN combines with specific antibody to form insoluble antigen-antibody complexes. The SYNCHRON® System(s) automatically dilutes sample and dispenses the appropriate sample and reagent volumes into a cuvette. The ratio used is one-part diluted sample to 37.5 parts reagent. The system monitors the change in absorbance at 340 nanometers. This change in absorbance is proportional to the concentration of transferrin in the sample and is used by the System to calculate and express the transferrin concentration based upon a single-point calibration.

1. ***Tools and specific procedures: Record sheet.***

Data will be collected using a record information sheet for demographic data, environmental parameters, recent food intake, dietary intake, anthropometrics, and biochemical parameters. The record sheet will be developed, and pilot tested to check for anything that could impede the instrument's ability to collect data in economical and systematic fashion.

1. ***Validity and Reliability***

The validity of a measurement instrument is the extent to which the instrument measures what it is supposed to measure. Furthermore, reliability is the consistency with which a measuring instrument yields results with repeated measurements, preferably more than 10 repeats. Both validity and reliability, then, reflect the degree to which we may have errors in our measurements (Leedy and Ormrod, 2010). To ensure validity, the measurements for anthropometry will be taken twice and the average will be calculated, and the developed questionnaire and intervention will be piloted and pre-tested before data collection.

To ensure reliability, the developed questionnaire and educational lesson (the song) will be translated into *Tshivenda* and *Xitsonga*, which are the local language spoken in Musina and Collins Chabane municipalities. Experts from the MER Centre for African languages at the University of Venda will carry this exercise. One expert will translate the questionnaire from English to *Tshivenda* and *Xitsonga* and another expert from the same department will translate the *Tshivenda* and *Xitsonga* version of the questionnaire back to English.

1. ***Data analysis***

Demographic, environmental, dietary diversity score, FFQ, zinc and iron data will be captured on the spreadsheet excel for further statistical analysis using SPSS version 24 or latest. Means, frequencies, chi-square, median and interquartile range, Pearson’s correlation and regression analysis will be used for descriptive statistics.

***Anthropometrics***

Anthropometric measurements (weight, height and MUAC) will be analysed using World Health Organization Anthro and Anthroplus version 3.2.2 (WHO, 2011). Weight for age, height-for-age, and weight-for-height will be interpreted using the Z-scores. The Z-scores system expresses the anthropometric values as a number of standard or Z-scores below or above the reference mean or median value (<http://www.who.into/nutgrowthdb/into>, Accessed 15/02/2018). Z-scores, which are gender and age independent thus permitting the evaluation of children’s growth status, will be determined by weight for age (WAZ), height for age (HAZ) and weight for height (WHZ). The Z-scores classification indicated in Table 3.1 was used for interpretation.

**Table 3.1: WHO child growth standard (World Health Organization, 2008)**

| **Z-score** | **Growth indicators** | | | |
| --- | --- | --- | --- | --- |
|  | **Length/height for-age** | **Weight-for age** | **Weight-for length/height** | **BMI-for-age** |
| **Above 3** | See note 1 | See note 2 | Obese | Obese |
| **Above 2** |  |  | Overweight | Overweight |
| **Above 1** |  |  | Possible risk of overweight | Possible risk of overweight |
| **0 (median)** |  |  |  |  |
| **Below -1** |  |  |  |  |
| **Below -2** | Stunted | Underweight | Wasted | Wasted |
| **Below -3** | Severely stunted | Severely underweight | Severely wasted | Severely wasted |

*1. A child in this range is very tall. Tallness is rarely a problem, unless it is so excessive that it may indicate an endocrine disorder such as a growth-hormone-producing tumor. Refer a child in this range for assessment if you suspect an endocrine disorder (e.g. if parents of normal height have a child who is excessively tall for his or her age).*

*2. A child whose weight-for-age falls in this range may have a growth problem, but this is better assessed from weight-for-length/height or BMI-for-age.*

**Interpretation of MUAC indicators**

- MUAC less than 110mm (11.0cm), RED COLOUR, indicates Severe Acute Malnutrition (SAM). The child should be immediately referred for treatment.
- MUAC of between 110mm (11.0cm) and 125mm (12.5cm), RED COLOUR (3-colour Tape) or ORANGE COLOUR (4-colour Tape), indicates Moderate Acute Malnutrition (MAM). The child should be immediately referred for supplementation.
- MUAC of between 125mm (12.5cm) and 135mm (13.5cm), YELLOW COLOUR, indicates that the child is at risk for acute malnutrition and should be counselled and followed-up for Growth Promotion and Monitoring (GPM).
- MUAC over 135mm (13.5cm), GREEN COLOUR, indicates that the child is well nourished.

**
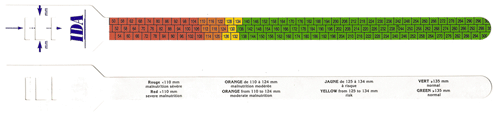
Figure 5: MUAC interpretation scale**

According to WHO, the recommended cut-offs for MUAC are valid from 6 months of age and defined as MUAC of less than 115 mm as severe malnutrition and less than 125 mm as malnutrition. Furthermore, MUAC z-scores of −3 SD is defined as severe malnutrition and MUAC z-score of −2 SD as malnutrition. These will be analysed using Anthroplus WHO software.

***Dietary patterns***

The 24-hour recall will be analysed using the 11-food item to determine dietary diversity score. The FFQ will be analysed using SPSS version 23 for frequencies as daily, weekly, monthly, seasonal or never. Consumption of indigenous foods will be determined using frequencies. Food finder (MRC) will also be used to analyse the nutrient intakes.

***Biochemical***

The table below indicates the cut-off points that will be used to compare normal values of pre-school children (IZiNCG, 2007) and iron.

**Table 3.2: An indication of lower cut-offs for serum zinc concentration (μmol/dL) by age group, gender, time of day and time since last meal** (IZiNCG, 2007).

| **Time of Day and Fasting Status** | **<10 years** | **≥10 years** | |
| --- | --- | --- | --- |
|  | **Males and Females** | **Non-pregnant females** | **Males** |
| Morning (fasting) | Not available | 70 μg/dL | 74 μg/dL |
| Morning (non-fasting) | 65 μg/dL | 66 μg/dL | 70 μg/dL |
| Afternoon (non-fasting) | 57 μg/dL | 59 μg/dL | 61 μg/dL |

***For conversion to μmol/L, divide by 6.54***

**Interpretation of Iron indicators**

***Table 3.3: An indication of lower cut-offs of serum iron concentration (*μg/L) by age**

| Anaemia | Hb levels <11 g/dL |
| --- | --- |
| Moderate anaemia | Hb level between 7 to 9.9 g/dL |
| Mild anaemia | Hb >10 to <11 g/dL |
|  | Hb<7 g/dL |
| Serum ferritin | (SF) <12 μg/L |
| Transferrin saturation | TSAT <15%. |
| C-reactive protein | 1 and 2.9 mg/L |

*de Benoist B, McLean E, Egli I, Cogswell M, editors. Worldwide prevalence of anaemia 1993–2005: WHO global database on anaemia accessed 20/01/ 2018*

**3.6.3 PHASE THREE: THE IMPLEMENTATION OF THE INTERVENTION**

**3.6.3.1. Aim:** To conduct the feeding trial using the *Curcubita Moschata* snack meal and the song.

**3.6.3.2. Objectives**:

3.6.3.2.1. To feed the children with the intervention or control for six months

- - - - 1. To assess adherence to the dietary intervention in terms of consuming Cucurbita *moschata* seed paste

**3.6.3.3. Design:** Study design will be a pretest-posttest control group trial, using cluster randomisation, which is a type of scientific experiment, which aims to reduce bias when testing a new treatment (Leedy and Ormrod, 2010). The preschools participating in the trial are randomly allocated either to the group receiving the treatment under investigation or to a group receiving standard treatment (or placebo treatment) as the control (Leedy and Ormrod, 2010). Randomized minimizes selection bias and the different comparison groups allow the researchers to determine any effects of the treatment when compared with the no treatment (control) group, while other variables are kept constant (Leedy and Ormrod, 2010). Randomization will take place at the preschool level. The research will be using single blinded trial with a knowledge of who is receiving the treatment amongst the participants.

- - - 1. **Intervention and data collection:**

The experimental group will receive their normal diet plus paste (grounded *Cucurbita moschata* seeds mixed with cooked *Cucurbita moschata* flesh) and the educational lesson through the song for six (6) months while the control group will receive normal diet plus mashed *Cucurbita moschata* flesh. The participants will consume ½ cup (64 g) (***to be confirmed after development and pilot***) paste (grinded *Cucurbita moschata* seeds mixed with mash *Cucurbita moschata* flesh) twice per week, during lunch with their normal diet while the control will consume mashed *Cucurbita moschata* flesh at the preschool sites over a six (6) month period. Two dishes made from grinded *Cucurbita moschata* seeds mixed with *Cucurbita moschata flesh* will be used to bring variety and improve compliance.

Grounded *Cucurbita moschata seeds* will be packaged at the food science laboratory at the university of Venda before being delivered to preschools. The packaged grounded *Cucurbita moschata seeds will be mixed with cooked Cucurbita moschata flesh at the preschool site. Mashed Cucurbita moschata flesh mixed with grinded seeds and Mashed Cucurbita moschata flesh* will be served to children during their lunchtime under the supervision of the researcher and trained field worker.

Promotion of the consumption of the seeds and nutrition education will be done in the form of a song, which is known to be the best method for educating children under the age of five years. The training on the consumption of the seeds will involve the guardian/parents during participant’s recruitment because children at this age are still fully dependent for food on their caregivers/parents who thus influence their eating habits (Curtis *et al*., 2011). The focus of the training will be on the health benefits of consuming seeds of *Cucurbita* *spp.* The songs will be easily learned by preschool children and will become favourites because of their familiarity. Furthermore, the songs will build confidence in preschool children and even shy children are likely to participate as part of a group or whole class. The recorded lyrics during the Pilot will be used. The table below is a schematic representation of the feeding trial.

**Table 3.3: Schematic representation of the feeding trial.**

| **Group** |  | **Estimated no** | **Intervention** | **Intervention days (three teams)** | **Quality control** |
| --- | --- | --- | --- | --- | --- |
| Experimental | Preschool 1 | 73 | Normal diet plus seed paste.  Song | Monday  Wednesday | Plate wastage observations and adherence recording |
|  | Preschool 2 | 73 | Normal diet plus seed paste  Song | Tuesday  Thursday | Plate wastage observations and adherence recording |
|  | Preschool 3 | 73 | Normal diet plus seed paste  Song | Monday  Thursday | Plate wastage observations and adherence recording |
|  | Preschool 4 | 73 | Normal diet plus seed paste  Song | Wednesday  Friday | Plate wastage observations and adherence recording |
| Control | Preschool 5 | 73 | Normal diet plus mashed butternut (placebo) | Monday  Wednesday | Plate wastage observations and adherence recording |
|  | Preschool 6 | 73 | Normal diet plus mashed butternut (placebo) | Tuesday  Thursday | Plate wastage observations and adherence recording |
|  | Preschool 7 | 73 | Normal diet plus mashed butternut (placebo) | Monday  Thursday | Plate wastage observations and adherence recording |
|  | Preschool 8 | 73 | Normal diet plus mashed butternut (placebo) | Wednesday  Friday | Plate wastage observations and adherence recording |

The intervention will be implemented over six months or 180 days. On intervention days, Monday to Thursday, the field workers and the researcher will be on site for quality control. They will work in teams of two, one team per preschool. The researcher will change team daily to ensure standardisation and quality control**.**

**3.6.3.5. Monitoring and quality control**

Plate wastage and consumption adherence will be monitored and recorded by trained field workers. Monitoring for plate wastage and adherence will be done each day randomly. In order to assess the plate wastage and adherence on the consumption of *Cucurbita moschata* seeds **Appendix E** will be used for recording. Trained field workers will record amount given and remaining after feeding on randomly selected children, ten each time, they will be expected to do this twice a week. To assess the consumption of other iron and zinc rich foods, the parents/caregivers will be requested to fill out a food dairy to record for food consumed by their children at home. A food diary will be given to parents/caregivers to record food that is been given to children at home. This will help to monitor the compliance on the consumption of iron and zinc rich food. They will be asked to fill one day per week covering all seven-week days over 6 months (**see Appendix F)**. Sensory evaluation will be done once a month in the experimental preschools by the researcher at the preschool site.

**3.6.3.6. Data analysis**

A record of consumption will be kept on whether the children did consume mashed *Cucurbita moschata* flesh mixed with grounded *Cucurbita moschata* seeds and will be recorded in frequencies taking into consideration the wastage. Plate wastage observations will be quantified from the adherence record and plate wastage studies. Thematic analysis will be used to synthesize and interpret the observed data. Frequencies will also be used for food patterns analyses from the food diary using SPSS version 24 or latest.

A short quiz with two questions on the importance of the *Cucurbita moschata* seeds will be created to test the effectiveness of the song. The researcher conducts the quiz at the end of the six months with the children and analyse the findings.

**3.6.4.** **PHASE FOUR: POST FEEDING TRIAL**

**3.6.4.1. Aim:** To determine efficacy of *Cucurbita moschata* seeds on iron and zinc status of preschool children.

**3.6.4.2.** **Objectives**

3.6.4.2.1. To determine the demographic and environmental factors of the participants.

3.6.4.2.2. To measure the anthropometry of the study participants (Weight, Height, MUAC)

3.6.4.2.3. Determine the dietary diversity score of participants using 24- hour recall.

3.6.4.2.4. Determine dietary patterns of the study participants using FFQ.

3.6.4.2.5. To determine serum zinc concentrations among pre-school children using flame atomic absorption spectrophotometer before consuming *Cucurbita moschata* seeds.

3.6.4.2.6. To determine the iron status of pre-school children using hemoglobin, serum ferritin, transferrin receptor and C- reactive protein (CRP) before consuming the *Cucurbita moschata* (butternut) seeds.

**3.6.4.3. Design:** The study design will be post trial cross-sectional survey, similar to phase two. Quantitative data will be collected.

**3.6.4.4. Measurements, procedures and analysis**

The following measurements will be done: demographic and environmental factors, anthropometrics, dietary intakes and patterns, and biochemical data. Same parameters and procedures, which were done at the baseline and described in section 3.6.2.4, will be followed. Data will be analysed using same procedures as described for baseline, with addition regression and ANOVA to measure changes from baseline to post intervention, and between experimental and control. See table 3.5 below. This is done to determine the changes in nutritional status that is likely to have occurred because of the intervention. A statistician will be consulted.

**Table 3.5: Proposed data analysis table**

| **Descriptive Statistics** | **Categorical data** | **Numerical data** | |
| --- | --- | --- | --- |
|  |  | **Normally distributed** | **Non-normally distributed** |
| ***Socio demographic information***   - Age - Gender   ***Anthropometric measures***   - Weight, height, MUAC - Categorization according to Z-scores: height for age, weight for age and BMI for age   ***Nutrient intake from 24hr recall***   - Total intake, - Consumption pattern   ***Dietary patterns***  Frequency of inclusion of food item in the FFQ  Morbidity diary  **Biochemical measures**   - Serum zinc - haemoglobin - Serum Ferritin - C-reactive protein - Transferrin saturation - Serum ferritin | Frequencies n (%) | Mean $\pm$ SD | Median and IQR |
| ***Analytical statistics-*** |  |  |  |
| ***Comparison of micronutrient status by subject characteristics*** | Pearson’s Chi-square test/Fishers exact test | ANOVA/T-test | Kruskal-Wallis/Mann-Whitney U tests |
| ***Association between dietary intakes and micronutrient status*** | Regression analyses with dietary intakes and anthropometric indices as dependent variables and all other as independent | | |
| ***Validation of dietary intake and micronutrient status against the intervention*** | Spearman’s correlation coefficient  Comparison of means/means Weighted kappa statistic for agreement testing  Bland Altman plots for bias and agreement testing | | |

**3.7. Fieldworkers**

Five nutritionists will be recruited for monitoring, implementation of the feeding trial. The researcher will train field workers and methods of data collection will be standardized during piloting. The total team will be six including the researcher. Phlebotomist will be responsible for drawing blood as it is a requirement by the Health Professional Council of South Africa and these will be provided as part of the package from Ampath laboratories. The appointment of fieldworkers will be based on their expertise, which will be required for the study and for good general practice. There will be three teams with each visiting one primary school on the day of the feeding trial for monitoring. The table below illustrates the data collection responsibilities.

**Table 3.6: Personnel responsibilities during data collection**

| **PERSON RESPONSIBLE** | **Phase 1: Development of an intervention** | **Phase 2: Implementation of NE, NSFP meal and dish** | | |
| --- | --- | --- | --- | --- |
|  |  | **Phase Two** | **Phase 3:**  **Implement intervention** | **Phase 4:**  **Evaluate efficacy** |
| Promoters | Quality control | Quality control | Quality control | Quality control |
| Researcher | Recipe development and standardization  Training of field workers  Feedback meetings with promoters and quality control  Transport dishes for nutrient and microbial analysis | Arrange all resources needed for data collection car, equipment, pens etc.  Data collection with field workers  Feedback meetings with promoters and quality control  Data capturing and analysis | Purchasing of ingredients for preparation of Snack  Provide snack for preschools  Provide nutrition education using song  Quality control once every two weeks | Arrange all resources needed for data collection car, equipment, pens etc.  Data collection with field workers  Feedback meetings with promoters and quality control  Data capturing and analysis |
| Field workers  Nutritionist | Form part of the team  Assist in sensory evaluation  Pilot | Anthropometric measurements Questionnaire  Data capturing and cleaning | Intervention implementation Prepare the dishes  Serve the children  Data collection  Compliance  Plate waste studies | Questionnaires  Anthropometric measurements  Data capturing and cleaning |
| Field worker-  Phlebotomist | N/A | Draw blood  Transport to the lab storage and analysis | N/A | Draw blood and transport to the lab for storage and analysis |
| Pre-School teacher/principal | N/A | Subject recruitment  Allocate space | Receive ingredients/food  Monitoring | Participate in monitoring and quality control |

**3.8. Institutional approval**

The research proposal will be submitted to Health Research Ethics committee (HREC) of Stellenbosch University and to the Higher Degrees and Ethics committee of University of Venda for approval prior to data collection. An ethical clearance certificate will be issued, and it will be submitted to the Departments of Health and Social Development provincial department and district office together with the letter requesting permission to conduct research in pre-schools (**Appendix G**). Once permission is granted, a list of pre-schools will be obtained from the Department of Social Development, Vhembe district and the local municipalities will be consulted. Permission to include pre-schools into the sample will be sought with the headmasters and the school governing body.

**3.9. Ethical considerations**

No participants will be enrolled into the study until the protocol, participants’ information sheet and recruitment material are approved in writing by the Ethics Committees of the two Universities. The study will be performed in accordance with principles of the Declaration of Helsinki (2013), Good Clinical Practice and the laws of South Africa. No participants will be entered in the study without signed informed consent by parents after the researcher (see Appendix H) has provided a full and adequate oral and written explanation of the study, including possible risks and benefits. Verbal ascent will be obtained from the children before data collection. Consenting process will be conducted in the local languages (*Tshivenda* and *Xitsonga*) and consent forms will be made available in the same languages. Participants will have the right to withdraw from the study at any time. If in the process of the research a participant is diagnosed with a nutrition-related health risk or disease, the parents/guardian will be informed and referred to appropriate health professional or service to address the problem. Data generated from the study will be stored in a computer database, and in a manner, that maintains participants’ confidentiality. For data verification and quality control, purposes regulatory authorities and/ or members of the Ethics Committee of the Universities might be allowed access to participant data under conditions of strict confidentiality. This will be stated in the patient information sheet. The anonymity of participants will be ensured by using codes instead of names and in any publication of the data.

**3.10 Budget and sources of Income**

**Table 3.7: Proposed budget for the study**

| Item | Description | Unit | Unit price | Actual cost |
| --- | --- | --- | --- | --- |
| *Transport* | **UNIVEN to Polokwane, circuits in Vhembe district, selected preschools, Ampath lab and return (3302KM for data collection of first phase).**  **UNIVEN to selected preschools in Vhembe district (6900KM for 6 months’ data collection of second phase).**  **UNIVEN to selected preschools, Ampath Laboratory at Polokwane (3824KM for data collection of third phase)** | **3302KM**  **6900KM**  **3824KM** | **R3.55** | **R11722.1**  **R146970**  **R13575.2** |
| *Consultation visits at SU* | **Accommodation**  **Return Flight**  **Ground transportation for 5 days x2 trips** | **2**  **2**  **2** | **R1300**  **R5000**  **R3905** | **R16505** |
| *Equipment’s* | **centrifuge machine**  **needles**  **Test tubes**  **alcohol**  **cotton wool swabs.** |  |  | **R52500** |
| *Ampath* | **Package Nurse, blood collection and analysis**  **Full blood count & platelet count**  **Serum iron**  **Ferritin**  **Transferrin**  **CRP**  **Zinc** | **436 x 2** | **152.8**  **80.9**  **148.9**  **140.3**  **130**  **217.6** | **R759076** |
| *Consumables* | **Cucurbita Moschata seeds**  **Cool box**  **Stationary**  **Tape recorder**  **Digital discs or tapes** |  |  | **R18 000** |
| *Field worker* | **Nutritionist (9 months x 4 days per week at 16 days per month)** | **5** | **150 per day^1^** | **R108 000** |
| *Printing and editing* | **Editing (R30) per page**  **Printing of first draft**  **Printing of Second draft**  **Printing of questionnaires (3 pages x400 copies 1500)**  **Printing of final copy (4x80)**  **Binding (4 X R1000)**  **Spiral Binding (4 x R50)** | **80**  **160**  **160**  **2000**  **320**  **4**  **4** | **R30**  **R3**  **R3**  **R3**  **R3**  **R1000**  **R50** | **R14520** |
| *Analysis cost* | **microbial activity, toxicity level, micronutrient content, life span** |  |  | **R60 000** |
| *TOTAL* |  |  |  | **R1 114 668.3** |

**^1^per day value based on UNIVEN rates**

**Table 3.8: Source of Income**

| Source | Amount | Status |
| --- | --- | --- |
| UNIVEN capacity development grant (DHET) | **R300 000.00** | To apply once registered |
| UNIVEN support for research running costs staff registered for PHD | **R100 000.00** | To be accessed once the proposal is approved by Ethics Commitees |
| NRF (THUTHUKA) | R454 662.00 | Allocated /awarded for  2018-2020 |
| Other |  | To be identified with the support of promoters |
| Total | **R554 662.00** | **Available** |

**3.11 Dissemination of the information**

The results of this study will be reported using ta mixed dissertation format. Manuscripts for consideration in peer-reviewed journals will be drafted alongside during the finalisation of the dissertation. The proposed chapters will be:

Chapter one: Introduction

Chapter two: Literature review

Chapter three: Methodology

Chapter four: Development of *Cucurbita moschata* seeds recipes and nutrition education lesson on the importance of *Cucurbita moschata* using music.

Chapter five: Socio-economic and environmental parameters, nutritional status of children at baseline and six months after the intervention.

Chapter six: Consumption patterns of *Cucurbita moschata* (butternut) seeds, and their contribution to nutrient intake.

Chapter seven: The efficacy of the consumption of *Cucurbita moschata* seeds between the intervention and control groups, according to changes in zinc and iron status from baseline to post intervention (six months).

Chapter eight: Conclusions and recommendations

At least four articles are envisaged, two national and one international conferences presentations. A technical report will be prepared for sharing with the relevant institutions or government authority like the local and district municipalities, National or provincial Department of Social development, Department of Health, Department of Agriculture, Forestry and Fisheries and Department of Basic Education.

**3.12 Selekane’s PHD plan of action:**

| **Item No** | **Description of Activity** | **Time Frame** |
| --- | --- | --- |
|  | Application | 2017 |
|  | Registration | February 2018 |
|  | Proposal/protocol development   - Submission of the draft of proposal | February – March 2018 |
|  | Feedback from the supervisor | April 2018 |
|  | Correction and submission | April 2018 |
|  | Feedback from the supervisor | May 2018 |
|  | Correction and finalization of the proposal | May 2018 |
|  | - Meeting with Supervisor and Co-supervisor at Stellenbosch - Presentation of Protocol to Evaluation Committee | June/July 2018 |
|  | Corrections and submission | 1 week |
|  | Approval of Protocol by the Faculty | July 2018 |
|  | Ethics submission and approval   - SU HREC - UNIVEN Research and Ethics committee | Aug – Sep 2018 |
|  | Permission to conduct the study   - Department of Social development at Provincial office. - Vhembe District Department of Social Development and the Local Municipalities - Selected preschools | October - November 2018 |
|  | Registration | January 2019 |
|  | Purchasing of ingredients & consumables | January 2019 |
|  | Phase 1: Recipe development | January 2019 |
|  | Piloting | February 2019 |
|  | Phase 2: Baseline data collection | April 2021 |
|  | Phase 3: Conduct feeding trial | May 2021 |
|  | Phase 4: Post intervention data collection | November 2021 |
|  | Data capturing and cleaning | November 2021 |
|  | Write up and consultation visit (1 week in Jan) | January-March 2022 |
|  | Consult statistician (data analysis) | January 2022 |
|  | Feedback, 1 week writing retreat and consultation | April 2022 |
|  | Corrections and submission | May 2022 |
|  | Submission for Examination | August 2022 |
|  | Oral Examination | Oct/Nov 2022 |
|  | Graduation | December 2022 |

**References**

Abubakar N, Atiku MK, Alhassan AJ, Mohammed IY, Garba RM, and Gwarzo G D. (2017). An assessment of micronutrient deficiency: A comparative study of children with protein-energy malnutrition and apparently healthy controls in Kano, Northern Nigeria. *Tropical Journal of Medical Research.* 20 (1):61-5.

Achu MB. (2013). "Chemical evaluation of protein quality and phenolic compound levels of some Cucurbitaceae oilseeds from Cameroon. *International journal of Nutrition and Food science*. 12(7): 735-743."

[Akodu](https://www.ncbi.nlm.nih.gov/pubmed/?term=Akodu%20OS%5BAuthor%5D&cauthor=true&cauthor_uid=27358614) SO, [Disu](https://www.ncbi.nlm.nih.gov/pubmed/?term=Disu%20EA%5BAuthor%5D&cauthor=true&cauthor_uid=27358614) EA, [Njokanma](https://www.ncbi.nlm.nih.gov/pubmed/?term=Njokanma%20OF%5BAuthor%5D&cauthor=true&cauthor_uid=27358614) FO, and [Kehinde](https://www.ncbi.nlm.nih.gov/pubmed/?term=Kehinde%20OA%5BAuthor%5D&cauthor=true&cauthor_uid=27358614) OA. (2016). Iron deficiency anaemia among apparently healthy pre-school children in Lagos, Nigeria. [*African journal of Health Sci*](https://www.ncbi.nlm.nih.gov/pmc/articles/PMC4915399/)*ences*. 16(1): 61–68.

Amare B, Moges B, Fantahun B, Tafess K, Woldeyohannes D, Yismaw G, Ayane T, Yabutani T, Mulu A, Ota F, and Kassu A. (2012) Micronutrient levels and nutritional status of school children living in Northwest Ethiopia. *Journal of Nutrition*. 108:1-8.

Akhtar S. (2013). Zinc Status in South Asian Populations—An Update *Journal of Health Population Nutrition*. 31(2):139-149.

Andersson M, Karumbunathan V, and Zimmermann MB. (2012). Global iodine status in 2011 and trends over the past decade. *Journal of Nutrition*. 142: 744–750.

Arimond M, Hawkes C, Ruel MT, Sifri Z, Berti PR, Leroy JL, Low JW, Brown LR, and Frongillo EA. (2011) Agricultural interventions and nutrition: lessons from the past and new evidence. In: Thompson, B. and Amoroso, L. (eds) Combating Micronutrient Deficiencies: Food-based Approaches. Food and Agriculture Organization, Rome and CAB International, Wallingford, UK, 41–75.

Alves CX, Vale SHL., Dantas MMG, Maia AA, França MC, Marchini JS, Leite LD, and Brandao-Neto J. (2012). Positive effects of zinc supplementation on growth, GH, IGF1, and IGFBP3 in eutrophic children. *Journal of Pediatric. Endocrinology and Metabolism*. 25 (9-10):881–887.

Al-Zabedi ME, Kaid FA, Sady H, Al-Adhroey AH, Amran AA, and Al-Maktari TM. (2014). Prevalence and risk factors of iron deficiency anemia among children in Yemen. *American Journal of Health Research*. 2(5): 319-326.

Antunes MFR, Leite LD, Rocha EDM, Brito NJN, França MC, Silva CAB, Almeida MG, Rezende AA, Marchini JS, and Brandão-Neto J. (2010). Competitive interaction of zinc and iron after venous and oral zinc administration in eutrophic children. Trace Element. *Electrolytes*. 27:185–191.

Anzman-Frasca S, Savage JS, Marini ME, Fisher JO, and Birch LL. (2012). Repeated exposure and associative conditioning promote preschool children’s liking of vegetables. *Appetite*. 58(2):543-53.

Arlappa A, Laxmaiah A, Balakrishna N, Harikumar R, Mallikharjuna RK, Ch Gal R, Saradkumar S, Ravindranath M, and Brahmam GNV. (2011). Micronutrient deficiency disorders among the rural children of West Bengal, India. *Annals of Human Biology*. 38(3):281–289.

Balarajan Y, Ramakrishnan U, Özaltin E, Shankar AH, and Subramanian SV. (2011). Anaemia in low-income and middle-income countries. *The Lancet.* *14*78 (9809):2123–35.

Bailey RL, West KP, and Black RE. (2015). The Epidemiology of Global Micronutrient Deficiencies. *Annals of Nutrition and Metabolism*. 66(2):22–33.

Baumgartner J, Smuts CM, Aleberli I, Malan L, Tjalsma H, and Zimmermann MB. (2013). Overweight impairs efficacy of iron supplementation in iron-deficient South African children: a randomized controlled intervention. *International journal of Obesity*. 37(1):24-30.

[Bahizire](http://www.ajtmh.org/search?value1=Esto+Bahizire&option1=author&noRedirect=true) E, [Bahwere](http://www.ajtmh.org/search?value1=Paluku+Bahwere&option1=author&noRedirect=true) P, [Donnen](http://www.ajtmh.org/search?value1=Philippe+Donnen&option1=author&noRedirect=true) P, [Tugirimana](http://www.ajtmh.org/search?value1=P.+Lundimu+Tugirimana&option1=author&noRedirect=true) LP, [Balol'ebwami](http://www.ajtmh.org/search?value1=Serge+Balol%26apos%3Bebwami&option1=author&noRedirect=true) S, [Dramaix](http://www.ajtmh.org/search?value1=Mich%C3%A8le+Dramaix&option1=author&noRedirect=true) M, [Nfundiko](http://www.ajtmh.org/search?value1=Chouchou+Nfundiko&option1=author&noRedirect=true) C, [Chirimwami](http://www.ajtmh.org/search?value1=Rapha%C3%ABl+Chirimwami&option1=author&noRedirect=true) R, and [Mubagwa](http://www.ajtmh.org/search?value1=Kanigula+Mubagwa&option1=author&noRedirect=true) K. (2017). High Prevalence of Anemia but Low Level of Iron Deficiency in Preschool Children during a Low Transmission Period of Malaria in Rural Kivu, Democratic Republic of the Congo. [*The American Journal of Tropical Medicine and Hygiene*](http://www.ajtmh.org/content/journals/14761645)*.* [97(2](http://www.ajtmh.org/content/journals/14761645/97/2)):489 – 496.

[Bhandari](https://www.hindawi.com/40646590/) S and [Banjara](https://www.hindawi.com/52430675/) MR. (2015). Micronutrients Deficiency, a Hidden Hunger in Nepal: Prevalence, Causes, Consequences, and Solutions. *International Scholarly Research Notices*. 1-9.

Black MM. (2012). Integrated strategies needed to prevent iron deficiency and to promote early child development. *Journal of Trace Elements in Medicine and Biology.* 26(0):120–123.

[Black RE](https://www.ncbi.nlm.nih.gov/pubmed/?term=Black%20RE%5BAuthor%5D&cauthor=true&cauthor_uid=23746772), [Victora CG](https://www.ncbi.nlm.nih.gov/pubmed/?term=Victora%20CG%5BAuthor%5D&cauthor=true&cauthor_uid=23746772), [Walker SP](https://www.ncbi.nlm.nih.gov/pubmed/?term=Walker%20SP%5BAuthor%5D&cauthor=true&cauthor_uid=23746772), [Bhutta ZA](https://www.ncbi.nlm.nih.gov/pubmed/?term=Bhutta%20ZA%5BAuthor%5D&cauthor=true&cauthor_uid=23746772), [Christian P](https://www.ncbi.nlm.nih.gov/pubmed/?term=Christian%20P%5BAuthor%5D&cauthor=true&cauthor_uid=23746772), [de Onis M](https://www.ncbi.nlm.nih.gov/pubmed/?term=de%20Onis%20M%5BAuthor%5D&cauthor=true&cauthor_uid=23746772), [Ezzati M](https://www.ncbi.nlm.nih.gov/pubmed/?term=Ezzati%20M%5BAuthor%5D&cauthor=true&cauthor_uid=23746772), [Grantham-McGregor S](https://www.ncbi.nlm.nih.gov/pubmed/?term=Grantham-McGregor%20S%5BAuthor%5D&cauthor=true&cauthor_uid=23746772), [Katz J](https://www.ncbi.nlm.nih.gov/pubmed/?term=Katz%20J%5BAuthor%5D&cauthor=true&cauthor_uid=23746772), [Martorell R](https://www.ncbi.nlm.nih.gov/pubmed/?term=Martorell%20R%5BAuthor%5D&cauthor=true&cauthor_uid=23746772), and [Uauy R](https://www.ncbi.nlm.nih.gov/pubmed/?term=Uauy%20R%5BAuthor%5D&cauthor=true&cauthor_uid=23746772). (2013). Maternal and child undernutrition and overweight in low-income and middle-income countries. *Lancet;* 382(9890):427-451.

Black MM, Quigg AM, Hurley KM, and Pepper MR. (2011). Iron deficiency and iron-deficiency anemia in the first two years of life: Strategies to prevent loss of developmental potential. *Nutrition Review.* 69(1): S64–S70.

Best C, Neufingerl N, van Geel L, van den Briel T, and Osendarp S. (2010). The nutritional status of school-aged children: Why should we care? *Food and Nutrition Bulletin*, 31(3): 400-417.

Best C, Neufingerl N, Del Rosso JM, Transler C, van den Briel T, and Osendarp S. (2011) Can multi micronutrient food fortification improve the micronutrient status, growth, health, and cognition of schoolchildren? A systematic review. *Nutrition Reviews*. 69 (4):186-204.

Best C, Neufingerl N, van Geel L, van den Briel T, and Osendarp S. (2010). The nutritional status of school-aged children: why should we care? *Food Nutrition Bulletin*.31(3):400-17.

Beinner MA, Velasquez-Melendez G, Pessoa MC, and Greiner T. (2010). Iron-fortified rice is as efficacious as supplemental iron drops in infants and young children. *Journal of Nutrition.* 140:49–53.

Bhaskaram P. (2014). Micronutrient Malnutrition, Infection, and Immunity: *An Overview Nutrition Reviews.* 60 (5):S40-S45.

Bresani CC, Wessells KR, and Brown KH. (2012). Estimating the Global Prevalence of Zinc Deficiency: Results Based on Zinc Availability in National Food Supplies and the Prevalence of Stunting. *PLOS ONE*. 7(11):1-11.

Bortolini GA, Gubert MB, and Santos LM. (2012). Food consumption in Brazilian children by 6 to 59 months of age. *Report in Public Health*. 28:71-1759.

Butte NF, Fox MK, Briefel RR, Siega-Riz AM, Dwyer JT, Deming DM, and Reidy KC. (2010). Nutrient intakes of US infants, toddlers, and preschoolers meet or exceed dietary reference intakes. *Journal of the American Dietetic Association*. 110 (12 Suppl): S27-37.

Castillo JJ. (2009). Simple Random Sampling. Retrieved 06 may, 2017, from <http://www.experimentresources.com/simple-randomsampling.html>.

Christian P, and Stewart CP. (2010). Maternal micronutrient deficiency, fetal development, and the risk of chronic disease. *Journal of Nutrition.* 140(3):437-45.

[Cole](https://www.ncbi.nlm.nih.gov/pubmed/?term=Cole%20CR%5BAuthor%5D&cauthor=true&cauthor_uid=20147474) RC,  [Grant](https://www.ncbi.nlm.nih.gov/pubmed/?term=Grant%20FK%5BAuthor%5D&cauthor=true&cauthor_uid=20147474) KF,  [Swaby-Ellis](https://www.ncbi.nlm.nih.gov/pubmed/?term=Swaby-Ellis%20ED%5BAuthor%5D&cauthor=true&cauthor_uid=20147474) DE,  [Smith](https://www.ncbi.nlm.nih.gov/pubmed/?term=Smith%20JL%5BAuthor%5D&cauthor=true&cauthor_uid=20147474) JL, [Jacques](https://www.ncbi.nlm.nih.gov/pubmed/?term=Jacques%20A%5BAuthor%5D&cauthor=true&cauthor_uid=20147474) A,  [Northrop-Clewes](https://www.ncbi.nlm.nih.gov/pubmed/?term=Northrop-Clewes%20CA%5BAuthor%5D&cauthor=true&cauthor_uid=20147474) AC,  [Caldwell](https://www.ncbi.nlm.nih.gov/pubmed/?term=Caldwell%20KL%5BAuthor%5D&cauthor=true&cauthor_uid=20147474) KL,  [Pfeiffer](https://www.ncbi.nlm.nih.gov/pubmed/?term=Pfeiffer%20CM%5BAuthor%5D&cauthor=true&cauthor_uid=20147474) CM, and  [Ziegler](https://www.ncbi.nlm.nih.gov/pubmed/?term=Ziegler%20TR%5BAuthor%5D&cauthor=true&cauthor_uid=20147474) RT. (2010). Zinc and iron deficiency and their interrelations in low-income African American and Hispanic children in Atlanta. [*American Journal of Clinical Nutr*](https://www.ncbi.nlm.nih.gov/pmc/articles/PMC2844684/)*ition*. 91 (4):1027–1034.

Cortez-Vega RW, Piotrowicz IBB, Prentice C, and Borges CD. (2014). Influence of different edible coatings in minimally processed pumpkin (Cucurbita moschata Duch). *International Food Research Journal* 21(5):2017-2023.

Chaudhary J, Jora R, Sharma P, Gehlot R, and Sushil A. (2015). A study of iron and zinc deficiency on short term memory in children & effect of their supplementation. *Asian Journal of Biomedical and Pharmacutical Sciences*, 5(42):12-15.

[Chege](https://www.ncbi.nlm.nih.gov/pubmed/?term=Chege%20PM%5BAuthor%5D&cauthor=true&cauthor_uid=26450270) PM, [Kimiywe](https://www.ncbi.nlm.nih.gov/pubmed/?term=Kimiywe%20JO%5BAuthor%5D&cauthor=true&cauthor_uid=26450270) JO, and [Ndungu](https://www.ncbi.nlm.nih.gov/pubmed/?term=Ndungu%20ZW%5BAuthor%5D&cauthor=true&cauthor_uid=26450270) WZ. (2015). Influence of culture on dietary practices of children under five years among Maasai pastoralists in Kajiado, Kenya. *International Journal of Behavioral Nutrition and Physical Activity*. 12(131):1-6.

Curtis P, Stapleton H, and James A. (2011). Intergenerational relations and the family food environment in families with a child with obesity. *Annals of Human Biology.* 38(4): 1–9.

Darnton-Hill I, and Cogill B. (2010). Maternal and young child nutrition adversely affected by external shocks such as increasing global food prices. *Journal of Nutrition* 140:162S–169S

Dehghani SM, Katibeh P, Haghighat M, Moravej H and Asadi S. (2011). prevalence of zinc deficiency in 3-18 years old children in Shiraz-Iran. *Iran Red Crescent Medical Journal*. 13(1):4-8.

Dey S, Gosawmi S, and Dey T. (2013). “Identifying predictors of childhood anaemia in north-east India,” *Journal of Health and Population Nutrition*. 31(4):462–470.

Di Noia J, and Byrd-Bredbenner C. (2014). Determinants of fruit and vegetable intake in low-income children and adolescents. *Nutrition Review*. 72(9):575–590

Durante M, Lenucci SM, and Mita G. (2014). Supercritical Carbon Dioxide Extraction of Carotenoids from Pumpkin (Cucurbita spp.): A Review. *International Journal of Molecular Sciences*. 15 (4):6725-6740.

Donangelo CM, and King JC. (2012). Maternal zinc intakes and homeostatic adjustments during pregnancy and lactation. *Nutrients*. 4(7):782-98.

Doyle ME, and Glass KA. (2010). Sodium reduction and its effect on food safety, food quality, and human health. *Comprehensive review in food science and food safety*. 9:44-55.

DoH. (2016). Nutrition Guidelines for Early Childhood Development Centres, South Africa.

Echessa ACP, Nyambaka H, Ondigi NA, Omuterema S, Toili W, Afihini SMI, and Sande A. (2013). Evaluation of micronutrients in seeds of Pumpkin varieties grown by smallholder farmers in the Lake Victoria Basin. *African Journal of Food Science and Technology*. 4(10):221-228.

Engle-Stone R, Killilea DW, Ndjebayi AO, Nankap M, and Brown KH. (2014). Stunting Prevalence, Plasma Zinc Concentrations, and Dietary Zinc Intakes in a Nationally Representative Sample Suggest a High Risk of Zinc Deficiency Among Women and Young Children in Cameroon. *Journal of Nutrition*.144(3):382-91.

Ejaz MS, and Latif N. (2010). Stunting and micronutrient deficiencies in malnourished children. *Journal of the Pakistan Medical Association*. 60 (7):543-7.

Engle-Stone R, Ndjebayi AO, Nankap M, Killilea DW, and Brown KH. (2014). Stunting prevalence, plasma zinc concentrations and dietary zinc intakes in a nationally representative sample suggest a high risk of zinc deficiency among women and young children in Cameroon. *Journal of Nutrition.* 144 (27):382-391.

[Etani Y](https://www.ncbi.nlm.nih.gov/pubmed/?term=Etani%20Y%5BAuthor%5D&cauthor=true&cauthor_uid=25294396), [Nishimoto Y](https://www.ncbi.nlm.nih.gov/pubmed/?term=Nishimoto%20Y%5BAuthor%5D&cauthor=true&cauthor_uid=25294396), [Kawamoto K](https://www.ncbi.nlm.nih.gov/pubmed/?term=Kawamoto%20K%5BAuthor%5D&cauthor=true&cauthor_uid=25294396), [Yamada H](https://www.ncbi.nlm.nih.gov/pubmed/?term=Yamada%20H%5BAuthor%5D&cauthor=true&cauthor_uid=25294396), [Shouji Y](https://www.ncbi.nlm.nih.gov/pubmed/?term=Shouji%20Y%5BAuthor%5D&cauthor=true&cauthor_uid=25294396), [Kawahara H](https://www.ncbi.nlm.nih.gov/pubmed/?term=Kawahara%20H%5BAuthor%5D&cauthor=true&cauthor_uid=25294396), [Ida S](https://www.ncbi.nlm.nih.gov/pubmed/?term=Ida%20S%5BAuthor%5D&cauthor=true&cauthor_uid=25294396). (2014). Selenium deficiency in children and adolescents nourished by parenteral nutrition and/or selenium-deficient enteral formula. [Journal of Trace Element and Medical Biology.](https://www.ncbi.nlm.nih.gov/pubmed/25294396) 28(4):409-13.

FAO, IFAD, and WFP. The State of Food Insecurity in the World 2015. Meeting the 2015 International Hunger Targets: Taking Stock of Uneven Progress. Rome: FAO; 2015.

Ferguson F, Chege P, Kimiywe J, Wiesmann D, and Hotz C. (2015). Zinc, iron and calcium are major limiting nutrients in the complementary diets of rural Kenyan children. *Maternal and child Nutrition*. 11(3):6-20.

Fox MK, Condon E, Briefel RR, Reidy KC, and Deming DM. (2010). Food consumption patterns of young preschoolers: are they starting off on the right path? *Journal of the American Dietetic Association*. 110(12):S52**-**9.

Galetti V, Mitchikpe CES, Kujinga P, Tossou F, Hounhouigan DJ, Zimmermann MB, and Moretti D. (2016). Rural Beninese children are at risk of zinc deficiency according to stunting prevalence and plasma zinc concentration but not dietary zinc status. *Journal of Nutrition* 146(1):114-23.

Gibson RS. (2005): Principles of nutritional assessment. 2nd ed. Oxford University Press, Oxford.

Gibson RS. (2011). Strategies for preventing multi-micronutrient deficiencies: a review of experiences with food-based approaches in developing countries. In: Combating Micronutrient Deficiencies: Food-based Approaches. Food and Agriculture Organization, Rome and CAB International, Wallingford, UK, pp. 7–27.

Grimm KA, Kim SA, Yaroch LA, and Scanlon KS. (2014). Fruit and Vegetable Intake During Infancy and Early Childhood. [*Pediatrics*](http://pediatrics.aappublications.org/) [134(1](http://pediatrics.aappublications.org/content/134/Supplement_1)):S*14*- S69.

Gottardi D, Bukvicki D, Prasad S, and Tyagi AK. (2016). Beneﬁcial Effects of Spices in Food Preservation and Safety. *Frontier in Microbiology*. 7:1394.

[Habib](https://www.ncbi.nlm.nih.gov/pubmed/?term=Habib%20MA%5BAuthor%5D&cauthor=true&cauthor_uid=27171139) MA, [Black](https://www.ncbi.nlm.nih.gov/pubmed/?term=Black%20K%5BAuthor%5D&cauthor=true&cauthor_uid=27171139) K, [Soofi](https://www.ncbi.nlm.nih.gov/pubmed/?term=Soofi%20SB%5BAuthor%5D&cauthor=true&cauthor_uid=27171139) SB,  [Hussain](https://www.ncbi.nlm.nih.gov/pubmed/?term=Hussain%20I%5BAuthor%5D&cauthor=true&cauthor_uid=27171139) I,  [Bhatti](https://www.ncbi.nlm.nih.gov/pubmed/?term=Bhatti%20Z%5BAuthor%5D&cauthor=true&cauthor_uid=27171139) Z,  [Bhutta](https://www.ncbi.nlm.nih.gov/pubmed/?term=Bhutta%20ZA%5BAuthor%5D&cauthor=true&cauthor_uid=27171139) ZA, and  [Raynes-Greenow](https://www.ncbi.nlm.nih.gov/pubmed/?term=Raynes-Greenow%20C%5BAuthor%5D&cauthor=true&cauthor_uid=27171139) C. (2012). Prevalence and Predictors of Iron Deficiency Anemia in Children under Five Years of Age in Pakistan, A Secondary Analysis of National Nutrition Survey Data 2011–2012. Editor: François Blachie.PLoS ONE 11(5): e0155051. doi:10.1371/ journal. pone.0155051.

Hall K, Sambu W, Berry L, Giese S, Almeleh C, and Rosa S. (2016). South African Early Childhood Review 2016. Cape Town: Children’s Institute, University of Cape Town and Ilifa Labantwana.

Harrison GG. (2010). Public health interventions to combat micronutrient deficiencies. *Public Health Review*s. 32 (1):2*13*-266.

Herrador Z, Sordo L, Gadisa E, Buño A, Gómez-Rioja R, Iturzaeta JM, de Armas LF, Benito A, Aseffa A, Moreno J, Cañavate C, and Custodio E. (2014). [Micronutrient deficiencies and related factors in school-aged children in Ethiopia: a cross-sectional study in Libo Kemkem and Fogera districts, Amhara Regional State.](https://www.ncbi.nlm.nih.gov/pubmed/25546056) *PLoS One*. 29;9(12): e112858.

Hegazy AA, Zaher MM, Abd el-hafez MA, Morsy AA, and Saleh RA. (2010). Relation between anemia and blood levels of lead copper, zinc and iron among children. *BMC research notes*. 3:133.

Hettiarachchi M, and Liyanage C. (2012). Coexisting micronutrient deficiencies among Sri Lankan pre-school children: a community-based study. *Maternal Child Nutrition*. 8:259-66.

Holley EC, Farrow C, and Haycraft E. (2017). A Systematic Review of Methods for Increasing Vegetable Consumption in Early Childhood. [*Current Nutrition Reports*](https://link.springer.com/journal/13668). 6:157–170.

Horton S, and Steckel RH. (2013). Malnutrition. In: Lomborg, B, ed. How Much Have Global Problems Cost the World? Cambridge, UK: Cambridge University Press; 247–272.

Hussain J, Rehman NU, Khan AL, Hussain H, Al-Harrasi A, Ali L, Sami F and Shinwari Z. (2011). Determination of macro and micronutrients and nutritiona; prospects of six vegetable species of Mardan, Pakistan. *Pakistan Journal of Botany.* 43(6):2829-2833.

Ibeanu V, Okeke E, Onyechi U, and Ejiofor U. (2012). Assessment of anthropometric indices and zinc status of preschoolers in peri-urban community in south east Nigeria. *International Journal of Basic and Applied Science*. 12 (5):31-37.

Ibeawuchi ANE, Onyiriuka AN, and Abiodun PO. (2017). High prevalence of zinc deficiency in rural Nigerian preschool children: a community-based cross-sectional study. *Romanian Journal of Diabetes Nutrition & Metabolic Diseases.* 24(1):31-38.

Imdad A, and Bhutta ZA. (2011). Effect of preventive zinc supplementation on linear growth in children under 5 years of age in developing countries: a meta-analysis of studies for input to the lives saved tool. *BMC Public Health*. 11 (3): S22.

Instituto Brasileiro de Geografia e Estatística (IBGE). Pesquisa de Orc¸amentos Familiares 2008-2009: Análise do consumo alimentar pessoal no Brasil. Rio de Janeiro: IBGE; 2011.

Jaryum KH, Okoye ZSC, and Stoecker BJ. (2017). Incidence of Zinc Deficiency Among Under-five Children of Kanam Local Government Area, North-central Nigeria. *Asian Journal of Clinical Nutrition*. 9 (1):1-8.

Jooste PL, Labadarios D, Nel H, and Strydom E. (2007). Iodine content of household salt, drinking water and iodine status of women and children. In: Labadarios D. National food consumption survey: fortification baseline, Chapter 8: South Africa 2005. Stellenbosch: Department of Health.

Kassebaum NJ, Jasrasaria R, Naghavi M, Wulf SK, Johns N, Lozano R, Regan M, Weatherall D, Chou DP, Eisele TP, Flaxman SR, Pullan RL, Brooker SJ, and Murray CJ. (2014) A systematic analysis of global anemia burden from 1990 to 2010*. Blood*. 123(5):615-24.

Kawade R. (2012). Zinc status and its association with the health of adolescents: a review of studies in India. *Glob Health Action*. 5 (1):1-10.

Khalaf ME and Raizada MN. (2016). Taxonomic and functional diversity of cultured seed associated microbes of the cucurbit family. *BMC Microbiology*. 16(131):1-16.

Khan Khattak, MMA, and Ali S. (2010). Malnutrition and associated risk factors in pre-school children Pakistan. *Journal of Medical Sciences*. 10 (2):34-39.

Khan MS, Shah FU, Ahmed Z, and Shah A (2015) Hidden Deficiency of Micronutrients in Apparently Healthy Children of District Bannu, Khyber Pakhtunkhwa, Pakistan. *Biochemistry & Pharmacology*: Open Access 4(3):1-6.

Kalimbira AA, MacDonald C, and Simpson JR. (2010a) The impact of an integrated community-based micronutrient and health programme on stunting in Malawian preschool children. *Public Health Nutrition.* 13:720–729

Karanja JK, Mugendi BJ, Khamis FM, and Muchugi AN. (2013). Nutritional composition of pumpkin (Ccurbita spp) seed cultivated from selected Regions in Kenya. *Journal of Horticulture*. 3(1):17-22.

[Kim](https://www.ncbi.nlm.nih.gov/pubmed/?term=Kim%20MY%5BAuthor%5D&cauthor=true&cauthor_uid=22413037) MY,  [Kim](https://www.ncbi.nlm.nih.gov/pubmed/?term=Kim%20EJ%5BAuthor%5D&cauthor=true&cauthor_uid=22413037) EJ,  [Kim](https://www.ncbi.nlm.nih.gov/pubmed/?term=Kim%20YN%5BAuthor%5D&cauthor=true&cauthor_uid=22413037) NY,  [Choi](https://www.ncbi.nlm.nih.gov/pubmed/?term=Choi%20C%5BAuthor%5D&cauthor=true&cauthor_uid=22413037) C, and  [Lee](https://www.ncbi.nlm.nih.gov/pubmed/?term=Lee%20BH%5BAuthor%5D&cauthor=true&cauthor_uid=22413037) HB. (2012). Comparison of the chemical compositions and nutritive values of various pumpkin (*Cucurbitaceae*) species and parts. [*Nutrition Research and Pract*](https://www.ncbi.nlm.nih.gov/pmc/articles/PMC3296918/)*ice.* 6(1):21–27.

Kim SA, Moore LV, Galuska D, Wright AP, Harris D, Grummer-Strawn LM, Merlo CL, Nihiser AJ, and Rhodes DG. (2014). Vital signs: Fruit and vegetable intake among children—United States, 2003–2010. *Morbidity Mortality Weekly*. *14*:671–676

[Kisiangani](https://www.ncbi.nlm.nih.gov/pubmed/?term=Kisiangani%20I%5BAuthor%5D&cauthor=true&cauthor_uid=26405498) I, [Mbakaya](https://www.ncbi.nlm.nih.gov/pubmed/?term=Mbakaya%20C%5BAuthor%5D&cauthor=true&cauthor_uid=26405498) C, [Makokha](https://www.ncbi.nlm.nih.gov/pubmed/?term=Makokha%20A%5BAuthor%5D&cauthor=true&cauthor_uid=26405498) A, and [Magu](https://www.ncbi.nlm.nih.gov/pubmed/?term=Magu%20D%5BAuthor%5D&cauthor=true&cauthor_uid=26405498) D.(2015). Assessment of iron status among preschool children (6 to 59 months) with and without malaria in Western Province, Kenya. [*Pan African Medical J*](https://www.ncbi.nlm.nih.gov/pmc/articles/PMC4564432/)*ournal.* 21(62):1-13.

Kotecha PV. (2011). “Nutritional anemia in young children with focus on Asia and India.” *Indian Journal of Community Medicine*. 36(1):8–16.

Koo HC, Poh BK, Lee ST, Chong KH, Bragt MC, and Ruzita AT. (2016). Are Malaysian children achieving dietary guideline recommendations? *Asia Pacific Journal of Public Health*. 28(5):8S-20S.

Krebs-Smith SM, Guenther PM, Subar AF, Kirkpatrick SI, and Dodd KW. (2010). Americans do not meet federal dietary recommendations. Journal of Nutrition. 140(10):1832–1838.

Kudlová E, and Schneidrová D. (2012). Dietary patterns and their changes in early childhood. Cent *European Journal of Public Health*. 20 (2):126–134.

Kumera GT, Awoke T, Melese AT, Eshetie S, Mekuria G, Mekonnen F, Ewunetu T, and Gedle D. (2015). Prevalence of zinc deficiency and its association with dietary, serum albumin and intestinal parasitic infection among pregnant women attending antenatal care at the University of Gondar Hospital, Gondar, Northwest Ethiopia. *BMC Nutrition.* 1(31):1-11.

Kunyanga CN, Imungi JK, and Vellingir V (2013). Nutritional evaluation of indigenous foods with potential food-based solution to alleviate hunger and malnutrition in Kenya. *Journal of Applied Biosciences*. 67:5277–5288.

Kwiri R, Winini C, Musengi A, Mudyiwa M, Nyambi C, Muredzi P, and Malunga A. (2014). Proximate composition of pumpkin gourd (Cucurbita pepo) seeds from Zimbabwe. *International Journal of Nutrition and Food Sciences*. 3(4):279-283.

Lee DR, and Nieman CD. (2010). Nutritional Assessment. Fifth (5^th^) edition. McGraw-Hill, New York.

Leedy PD, and Ormrod JE. (2010). Practical Research planning and design. 9^th^ (Ninth) edition, person Education Inc, upper saddle river, New Jersey.

Lewu FB, and Mavengahama S. (2010). Wild vegetables in northern KwaZulu-Natal, South Africa: Current status of production and research needs. *Scientific Research and Essays*. 5(20):3044–3048.

Liu J, Ai YX, Hanlon A, Shi Z, Dickerman B, and Compher C. (2011). Micronutrients deficiency and associated sociodemographic factors in Chinese children. *World Journal Pediatric*. 7(3):217-223.

Looney SM, and Raynor HA (2011). Impact of portion size and energy density on snack intake in preschool-aged children. *Journal of the* *American Dietetic Association.* 111(3): 414-418.

Low M, Farrell A, Biggs BA, and Pasricha SR. (2013). Effects of daily iron supplementation in primary-school–aged children: systematic review and meta-analysis of randomized controlled trials. *Canadian Medical Association Journal*. 185(17): E791-802.

Mahan LK, Escott-Stump S, and Raymond JL. (2012). Krause’s Food and Nutrition Therapy, 13^th^ edition. St Louis, Missouri. Elsevier Saunders.

Magnus H. (2012). Malnutrition in South-Asia: Poverty, Diet or Lack of Female Empowerment? The Chr. Michelsen Institute (CMI), Bergen, Norway.

Mamabolo RL, and Alberts M. (2013). ‘Prevalence of anaemia and its associated factors in African children at one and three years residing in the Capricorn District of Limpopo Province, South Africa’, *Curationis.* 37(1):1-9.

Marles RJ. (2017). Mineral nutrient composition of vegetables, fruits and grains: The context of reports of apparent historical declines. *Journal of food composition and analysis*, *13*: 93-106.

Mavengahama S, McLachlan M, and de Clercq W. (2013). The role of wild vegetable species in household food security in maize based subsistence cropping systems. [*Food Security*](https://link.springer.com/journal/12571). 5 (2):227–233.

Mofunaya AAJ, and Edu EA. (2015). Physiological and biochemical changes in C*urcubita moschata Duch.Ex.Poir* inoculated with Nigerian strain of Moroccan watermelon *Mosaic* virus (MWMV): Lagenaria breviflora isolate. *International journal of plant pathplogy,* 6:36-47.

Monárrez-Espino J, López-Alarcón M, and Greiner T. (2011). Randomized placebo-controlled trial of guava juice as a source of ascorbic acid to reduce iron deficiency in Tarahumara indigenous schoolchildren of northern Mexico. *Journal of the American College of Nutrition.* 30*:191–200.*

Morales-Ruán MC, Villalpando S, García-Guerra A, Shamah-Levy T, Robledo-Pérez R, Ávila-Arcos MA, and Rivera JA. (2012). Iron, zinc, copper, and magnesium nutritional status in Mexican children aged 1 to 11 years. *Journal of* ***Public Health of Mexico***. 54:125-134.

Motadi SA, Mbhenyane XG, Mbhatsani HV, Mabapa NS, and Mamabolo RL. (2015). Prevalence of iron and zinc deficiencies among preschool child.ren ages 3 to 5 years in Vhembe district, Limpopo province, South Africa. *Journal of Nutrition.* 31:452–458.

Murray-Kolb LE, Khatry SK, Schaefer BA, Cole PM, LeClerq SC, Katz J, Morgan ME, Tielsch JM, and Christian P. (2012). Preschool Micronutrient Supplementation Effects on Intellectual and Motor Function in School-aged Nepalese Children. *Archivers of pediatrics and adolescent medicine*. 166(5):404-410.

Muthayya S, Rah JH, Sugimoto JD, Roos FF, Kraemer K, and Black RE. (2013). The global hidden hunger indices and maps: an advocacy tool for action. PLoS One 8(6): e67860. doi**.**org**/**10.1371**/**journal**.**pone**.**0067860.

Nepal AK, Gela B, Mehta K, Lamsal M, Pokharel PK and Baral N. (2014). Plasma zinc levels, anthropometric and socio-demographic characteristics of school children in eastern Nepal. *BMC Research Notes*. 7(18):1-6.

Neumann CG, Bwibo NO, Gewa CA, and Drorbaugh N. (2011). Animal-source foods as a food-based approach to address nutrient deficiencies and functional outcomes: a study among Kenyan schoolchildren. In: Combating Micronutrient Deficiencies: Food-based Approaches. Food and Agriculture Organization, Rome and CAB International, Wallingford, UK:117–136.

Ngui R, Lim YAL, Chong Kin L, Sek Chuen C, and Jaffar S. (2012). Association between Anaemia, Iron Deficiency Anaemia, Neglected Parasitic Infections and Socioeconomic Factors in Rural Children of West Malaysia. PLoS Negl Trop Dis. 6(3): e1550. doi: 10.1371/journal.pntd.0001550**.**

Nobre LN, Lessa AC, de Oliveira HC, Lamounier JA, and Francischini SCC. (2017). Iron-deficiency anemia and associated factors among preschool children in Diamantina, Minas Gerais, Brazil. *Brazilian Journal of Nutrition*. 30(2):185-196.

Olufemi SS, Gbadamosi OF, Akinrinmade R, and Oladapo AA. (2013). Infant feeding practices and Prevalence of Iron Deficiency Anemia among Children of 0 - 5 Years in Ondo State, Nigeria. *Journal of Pharmacy and Biological Sciences*. 5(3):34–39.

Onyeamaobi G, and Onimawo I. (2011). Zinc status of under-five children in rural and urban Imo State. *Nigeria Journal of Basic and Applied Sciences Research*. 1(6): 451-455.

Patel S. (2013). Pumpkin Cucurbita sp.) seeds as nutraceutic: a review on status quo and scopes. *Mediterranean Journal of Nutrition* *and* *Metabolism* 6 (3):183-189.

Patela S, and Raufb A. (2017). Edible seeds from Cucurbitaceae family as potential functional foods: Immense promises, few concerns. *Biomedicine & Pharmacotherapy* 91: 330–337.

Prieto MB, and Cid JL-H. (2011). Malnutrition in the critically ill child: the importance of enteral nutrition. *International Journal of Environmental Research and Public Health.* 8(11):4353–4366.

Pasricha S, Black JS. Muthayya, Shet A, Bhat V, Nagaraj S, Prashanth SN, Sudarshan H, Biggs BA, and Shet SA. (2010). “Determinants of anaemia among young children in rural India,” *Pediatrics*. 126(1): e140–e149.

Petrou S, and Kupek E. (2010). Poverty and childhood undernutrition in developing countries: A multi-national cohort study. *Social Science & Medicine*. 71 (7): 1366-1373.

Poh BK, Kathryn Tham BL, Wong SN, Winnie Chee SS, and Tee ES. (2012). Nutritional Status, Dietary Intake Patterns and Nutrition Knowledge of Children Aged 5-6 Years Attending Kindergartens in the Klang Valley, Malaysia. *Malaysian journal of Nutrition* 18(2):231 – 242.

Rawat S. (2015). Food Spoilage: Microorganisms and their prevention. Asian Journal of Plant Science and Research, 5(4):47-*13*.

Rysha A, Gjergji TM, and Ploeger A. (2017). Dietary habits and food intake frequency of preschool children. *Nutrition & Food Science*. 47(4):534-542.

Saurabh K, Shilpi Ranjan S, and Narayan JP. (2017). Co-morbidities and micronutrient deficiencies in children with severe acute malnutrition. *International Journal of Contemporary Pediatrics.* 4(4):1225-1227.

Salam A, MacPhail C, Das KJ, and Bhutta AZ. (2013). Effectiveness of Micronutrient Powders (MNP) in women and children. *BMC Public Health*. 13(Suppl 3): S22; 1-9.

Serdula M. (2010). Maximizing the impact of flour fortification to improve vitamin and mineral nutrition in populations. *Food and nutrition bulletin*. 31 (1):S86-93.

[Setnick J](https://www.ncbi.nlm.nih.gov/pubmed/?term=Setnick%20J%5BAuthor%5D&cauthor=true&cauthor_uid=20413694). (2010). Micronutrient deficiencies and supplementation in anorexia and bulimia nervosa: a review of literature. [*Nutrition Clinical Practice*.](https://www.ncbi.nlm.nih.gov/pubmed/20413694) 25(2):137-42.

Shaw JG, and Freiedman FJ. (2011). Iron Deficiency Anemia: Focus on Infectious Diseases in Lesser Developed Countries. *Anemia*. pp1-10. <http://dx.doi.org/10.1155/2011/260380>. Last accessed 08 January 2018

Shisana O, Labadarios D, Rehle T, Simbayi L, Zuma K, Dhansay A, Reddy P, Parker W, Hoosain E, Naidoo P, Hongoro C, Mchiza Z, Steyn NP, Dwane N, Makoae M, Maluleke T, Ramlagan S, Zungu N, Evans MG, Jacobs L, Faber M, and SANHANES-1 Team (2013) South African National Health and Nutrition Examination Survey (SANHANES-1). Cape Town: HSRC Press.

[Singh](https://www.hindawi.com/67245750/) KR, and [Patra](https://www.hindawi.com/84919814/) S. (2014). Extent of Anaemia among Preschool Children in EAG States, India: A Challenge to Policy Makers. *Anemia:*1-9 pages. Available from <http://www.hindawi.com/journals/anemia/2014/868752>. Last accessed 04 February 2018

Smith JL, and Brooker S. (2010). “Impact of hookworm infection and deworming on anaemia in non-pregnant populations: a systematic review,” *Tropical Medicine and International Health*. 15 (7):776–795.

**Shalaby NM, Shalaby MN, and Sayed OA.** (2017). Impact of parasitic infections on nutritional status and micronutrients in Saudi children. *Current Paediatric Research*. 21 (1): 1-7

Steve-Edemba CL. (2014). Biochemical Assessment of Zinc Status of Under-Five Children in Orphanages of Federal Capital Territory, Abuja, Nigeria. *IOSR Journal of Dental and Medical Sciences.* 13(7):60-70.

Steinfeldt L, Anand J, and Muray T. (2013). Food reporting patterns in the USDA Automated Multiple pass method. *Procedia food science*. 2: 145-1*13*.

**Padbidri Bhaskaram, M. D.**

Dr. Bhaskaram is with the National Institute of Nutrition (Indian Council of Medical Research), Jamai-Osmania P.O., Hyderabad-500007, Andhra Pradedsh, India

**Search for other works by this author on:**

[Oxford Academic](https://academic.oup.com/search-results?f_Authors=Padbidri+Bhaskaram)

[PubMed](http://www.ncbi.nlm.nih.gov/pubmed?cmd=search&term=Bhaskaram%20P)

[Google Scholar](http://scholar.google.com/scholar?q=%22author:Bhaskaram%20author:P.%22)

Soofi S, Cousens S, Iqbal SP, Akhund T, Khan J, Ahmed I, Zaidi AKM, Bhutta ZA. (2013)> Effect of provision of daily zinc and iron with several micronutrients on growth and morbidity among young children in Pakistan: a cluster-randomized trial. *The Lancet*. 382(9886):29–40.

Spears MC and Gregoire MB. (2010). Foodservice organizations: a managerial and systems approach. Upper Saddle River, N.J., Prentice Hall.

Thomas S. (2014). The role of nutrition in health promotion and chronic disease prevention. *Journal of the Academy of Nutrition and Dietetics*. 89:278-83.

Uchendu FN. (2011). Micronutrient Malnutrition, A Tragedy to Childhood Growth and Education Global. *Journal of Medical research*. 11(1):26-34.

Uusiku NP, Oelofse A, Duodu KG, Bester MG, and Faber M. 2010. Nutritional value of leafy vegetables of Sub-Saharan Africa and their potential contribution of human health: A review. *Journal of Food Composition and Analysis*. 23:499-509.

United Nation Children's Fund. (2014). Undernutrition Contributes to Half of All Deaths in Children under 5 and is Widespread in Asia and Africa, United Nation Children's Fund (UNICEF), New York, NY, USA, <http://data.unicef.org/nutrition/malnutrition> accessed 28 October 2017.

Vorster HH, Badham JB, and Venter CS. (2013). An introduction to the revised food-based dietary guidelines for South Africa. *South African Journal of Clinical Nutrition*. 26(3): S1-S164

Vorster H. (2010). The link between Poverty and Malnutrition: A South African Perspective. Health SA. *Gesondheid* .15 (1):1-6.

Vorster HH, Badham JB, and Venter CS. (2013). An introduction to the revised food-based dietary guidelines for South Africa. *South African Journal Clinical Nutrition*, 26(3): S1-S164.

Vivienne I, Elizabeth O, Uchenna O, and Uju E. (2012). Assessment of anthropometric indices, iron and zinc status of preschoolers in a peri-urban community in south east Nigeria. *International Journal of Basic and Applied Sciences*. 12(5):31–37.

Wang YS, Huang CW, Liu CC, Wang FM, Ho SC, Huang PW, Hou CC, Chuang LH, and Huang CC. (2012) Pumpkin (Cucurbita moschata) Fruit Extract Improves Physical Fatigue and Exercise Performance in Mice. *Molecules* .17:11864-11876.

Wessells KR,and Brown KH. (2012). Estimating the global prevalence of zinc deficiency: results based on zinc availability in national food supplies and the prevalence of stunting. *PLoS One.7*: e50*13*8.

Wessells KR, Singh GM, and Brown KH. (2012). Estimating the Global Prevalence of Inadequate Zinc Intake from National Food Balance Sheets: Effects of Methodological Assumptions. *PLOS ONE*. 7(11):1-13.

Willems MW, Nansubuga R, and Doornbos A. (2011). Iron and Zinc Intake in Children Living in Kibona, Rural Uganda. Thames Street, Wallingford Oxfordshire, United Kingdom.

Wieser S, Brunner B, Tzogiou C, Plessow R, Zimmermann MB, Farebrother J, Soofi S, Bhatti Z, Ahmed I, and Bhutta ZA. (2017). Societal Costs of Micronutrient Deficiencies in 6- to 59-month-old Children in Pakistan. *Food and Nutrition Bulletin*. 38(4):485-500.

WHO. Micronutrient Deficiencies- Iron Deficiency Anemia. WHO; Available: <http://www.who.int/nutrition/topics/ida/en/>. Accessed 14 November 2017**.**

World Health Organization. The global prevalence of anemia in 2011. Geneva: WHO; 2015.

WHO/WFP/UNICEF. (2008). Joint Statement on Improving Existing Recommendations on Treatment of Moderate malnutrition in Children under 5. Volume 1, Number 3, Geneva. Switzerland.

WHO. (2002). Keep fit for life. Fifty-Fifth World Health Assembly, Geneva, World Health Organization, <http://www.who.int/gb/>.

WHO. (2017). Guideline: preventive chemotherapy to control soil-transmitted helminth infections in at-risk population groups. Geneva: World Health Organization; 2017. Licence: CC BY-NC-SA 3.0 IGO, Barcelona, Spain.

Yadav M, Jain S, Tomar R, Prasad GB, and Yadav H. (2010). Medicinal and biological potential of pumpkin: an updated review. Nutrition Research Review. 23:184-90.

World Health Organization (WHO). Guidelines for Food Fortification; World Health Organization: Geneva, Switzerland, 2006. Available online: http://www.who.int/nutrition/publications/guide_food_fortification_ micronutrients.pdf (accessed on 15 August 2016)**.**

Welch VA, Ghogomu E, Hossain A, Awasthi S, Bhutta ZA, Cumberbatch C, Fletcher R, McGowan J, Krishnaratne S, Kristjansson E, Sohani S, Suresh S, Tugwell P, White H, Wells GA. (2017). Mass deworming to improve developmental health and wellbeing of children in low-income and middle-income countries: a systematic review and network meta-analysis. The Lancet 5(1), e40–e50.

Zalilah MS, Khor GL, Sarina S, Lee HS, Chin YS, Barakatun Nisak MY, Chan YM, and Maznorila M. (2015). The relationship between house hold income and dietary intakes of 1–10-year-old urban Malaysian. *Nutrition Research and Practice.* 9(3):278–287.

Zlotkin S. (2011). Micronutrient deficiencies and effect of supplements on correcting them. Nestle Nutrition Workshop Ser Paediatrics Program.68:127–134; discussion. 134–140.

Zhou SJ, Gibson RS, and Makrides M. (2012). Nutrient intakes and status of preschool children in Adelaide, South Australia. *Medical Journal of Australia*. 196 (11):696-700.

**Appendix: A: Hedonic Scale**

| **Sensory evaluation form** |
| --- |

**Date of data collection…………………….Subject Code:……………………………….**

TITLE OF THE RESEARCH PROJECT:

Efficacy of consumption of *Cucurbita moschata* seeds in management of zinc and iron status in preschool children in Vhembe district, Limpopo province.

**REFERENCE NUMBER:**

**PRINCIPAL INVESTIGATOR:**

Motadi Selekane Ananias

**ADDRESS:**

Department of Human Nutrition, University of Venda, Po Box 5050, Thohoyandou

**CONTACT NUMBERS:**

0797455449/0670708030

**Office hours**: 015 962 8683

**
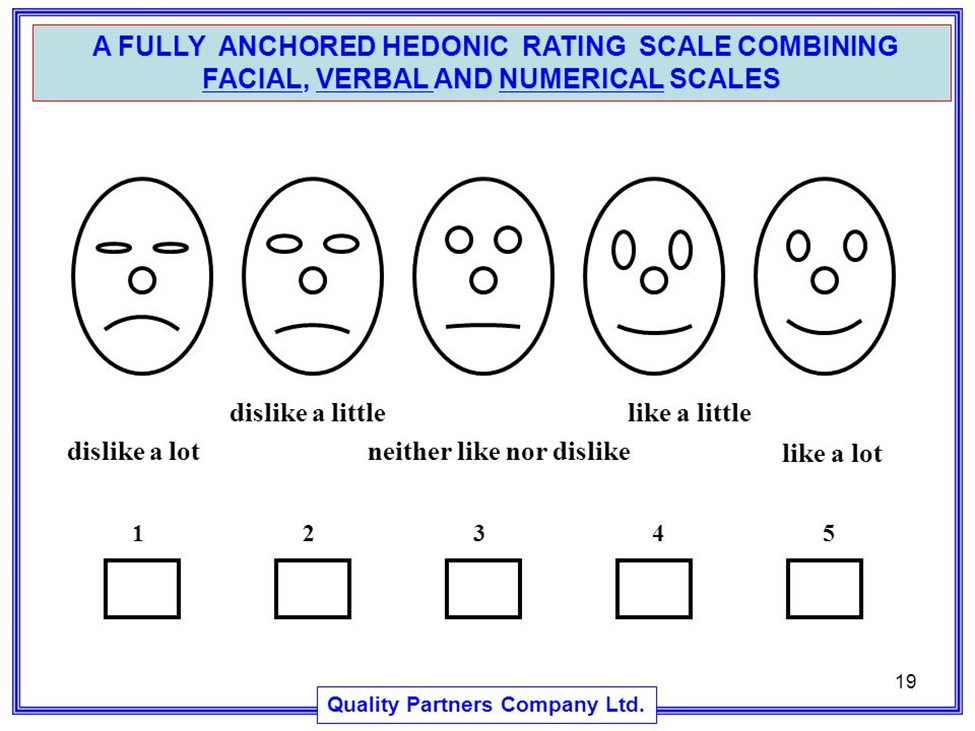
**

**Adopted from Quality partners company Ltd, 2018.**

**APPENDIX B: Baseline survey Date of data collection…………………….Subject Code:……………………………….**

TITLE OF THE RESEARCH PROJECT:

Efficacy of consumption of *Cucurbita moschata* seeds in management of zinc and iron status in preschool children in Vhembe district, Limpopo province.

**REFERENCE NUMBER:**

**PRINCIPAL INVESTIGATOR:**

Motadi Selekane Ananias

**ADDRESS:**

Department of Human Nutrition

University of Venda

Po Box 5050, Thohoyandou

**CONTACT NUMBERS:**

0797455449/0670708030

**Office hours**: 015 962 8683

**DEMOGRAPHIC DATA**

1. Marital status of mother/caregiver

|  | Code | Mark with an X |
| --- | --- | --- |
| Single | 1 |  |
| Married | 2 |  |
| Widowed | 3 |  |
| Divorced | 4 |  |

2.Highest education of mother

|  | Code | Mark with an X |
| --- | --- | --- |
| Never attended school | 1 |  |
| Grade 1 – 4 | 2 |  |
| Grade 5 – 7 | 3 |  |
| Grade 8 – 10 | 4 |  |
| Grade 11 – 12 | 5 |  |
| Tertiary | 6 |  |

3.Is the mother working?

|  | Code | Mark with an X |
| --- | --- | --- |
| Yes | 1 |  |
| No | 2 |  |

4.Who is responsible for household income?

|  | Code | Mark with an X |
| --- | --- | --- |
| Mother | 1 |  |
| Father | 2 |  |
| Grandparents | 3 |  |
| Mother and Father | 4 |  |
| Other, Specify ………………………… | 5 |  |

5.Type of income/employment

|  | Code | Mark with an X |
| --- | --- | --- |
| Health worker | 1 |  |
| Educator | 2 |  |
| Police | 3 |  |
| Retail |  |  |
| Social grant, Specify………… | 4 |  |
| Other, Specify ………………. | 5 |  |

6.Monthly income of the family/household

|  | Code | Mark with an X |
| --- | --- | --- |
| Less than R500 | 1 |  |
| R1000 – R2000 | 2 |  |
| R2000 – R5000 | 3 |  |
| R6000 – R10000 | 4 |  |
| R10000 above | 5 |  |

7. Does the child receive Child Grant

| Yes | No |
| --- | --- |

8. Any other form of grant

| Yes | No |
| --- | --- |

Comment:………………………………………………………………………………………….

**BIOPHYSICAL ENVIRONMENT**

1. **Type of house**

| **Type (a)**  **(yes 1; no 2)** | | **Number of rooms (b)** |
| --- | --- | --- |
| Hut |  |  |
| Brick and mortar |  |  |
| Shack |  |  |
| Other |  |  |

1. **Source of Water**

|  | **Source (yes 1; no 2)** | |
| --- | --- | --- |
| 10.1 | Tap in house |  |
| 10.2 | Tap outside |  |
| 10.3 | Communal tap |  |
| 10.4 | River |  |
| 10.5 | Tank (rain water harvest) |  |
| 10.6 | Bore hole |  |
| 10.7 | Spring/ well |  |
| 10.8 | Other |  |

1. **Type of Toilet**

| **Code** | **Type** | **Number and Tick appropriate boxes** |
| --- | --- | --- |
| 11.1 | Flush Toilet in house |  |
| *11*.2 | Flush Toilet outside |  |
| *11*.3 | Pit latrine |  |
| *11*.4 | Bush |  |
| *11*.5 | Other |  |

1. **Type of energy used for cooking in the household**

| **Code** | **Type of energy(yes 1; no 2)** | |
| --- | --- | --- |
| *12*.1 | Firewood |  |
| *12*.2 | Coal/ charcoal |  |
| *12*.3 | Electricity |  |
| *12*.4 | Gas |  |
| *12*.5 | Paraffin |  |
| *12*.6 | Gel |  |
| *12*.7 | Cow dung |  |
| *12*.*11* | Solar energy |  |
| *12*.10 | **Other** |  |

**13. Ownership of other assets**

| **Code** | **Type (yes 1; no 2)** | |
| --- | --- | --- |
| *13*.1 | Radio |  |
| *13*.2 | TV |  |
| *13*.3 | DSTV |  |
| *13*.4 | Video/DVD player |  |
| *13*.5 | House telephone |  |
| *13*.6 | Stove |  |
| *13*.7 | Microwave oven |  |
| *13*.*11* | Dining table and chairs |  |
| *13*.9 | Sofas |  |
| *13*.*10* | Play station (PS-VITA, X-box) |  |
| *13*.11 | Other, specify |  |

**FOOD SYSTEMS INVENTORY**

1. **Place of food purchase**

| **Code** | **Place (yes 1; no 2)** | |
| --- | --- | --- |
| *14*.1 | Local formal shops |  |
| *14*.2 | Spaza shops |  |
| *14*.3 | Shoprite |  |
| *14*.4 | Pick n Pay |  |
| *14*.5 | Spar |  |
| *14*.6 | Boxer |  |
| *14*.7 | USave |  |
| *14*.*8* | Checkers |  |
| *14*.9 | Choppies |  |
| *14*.10 | Game |  |
| *14*.*11* | Street vendors |  |
| *14*.12 | SaveMor |  |
| *14*.13 | OK grocery |  |
| *14*.14 | Pick up wild foods |  |
| *14*.15 | Other |  |

1. **Household food production (plants)**

| **Code** | **Type (yes 1; no 2)** | |
| --- | --- | --- |
| *15*.1 | Garden |  |
| *15*.2 | Orchard/ Fruit tree |  |
| *15*.3 | Field in the Household |  |
| *15*.4 | Fields away from household |  |
| *15*.5 | Small holder farm |  |
| *15*.6 | Large farm |  |
| *15*.7 | **Other** |  |

1. **Places for food storage**

| **Code** | **Type (yes 1; no 2)** | |
| --- | --- | --- |
| *16*.1 | Refrigerator |  |
| *16*.2 | Deep Freezer |  |
| *16*.3 | Dry room storage |  |
| *16*.4 | Cup board |  |
| *16*.5 | Not specific |  |
| *16*.6 | **Other** |  |

1. **Resources for food preparation**

| Code | Type (yes 1; no 2) | |
| --- | --- | --- |
| *17*.1 | Open fire outside |  |
| *17*.2 | Open fire inside |  |
| *17*.3 | Gas stove |  |
| *17*.4 | Paraffin stove (primus) |  |
| *17*.5 | Electric stove |  |
| *17*.6 | Coal stove |  |
| *17*.7 | Gel stove |  |
| *17*.*11* | Microwave |  |
| *17*.12 | Wonder box |  |
| *17*.13 | Other |  |

| **SECTION B: RECORD SHEET** |
| --- |

1. Name of the preschool……………………………………………
2. Child’s ID…………………………………………….
3. (ii.) Date of Birth……………… day………. Month………………year
4. (iii.) Age………………………….
5. Gender:

| 1 | 2 |
| --- | --- |
| Female | Male |

1. Did the child receive Deworming?

| 1 | 2 |
| --- | --- |
| Yes | No |

1. (a) Body weight……………………………… kg

(b) Body weight…………………………… kg

© Average weight…………………………….. kg

1. (a) Height………………………………. cm

(b) Height………………………………. cm

© Average height……………………………. cm

1. (a) MUAC ………………………………… cm

(b) MUAC………………………………… cm

© Average MUAC …………………. Cm

27 Was the faeces of the collected?

| 1 | 2 |
| --- | --- |
| Yes | No |

28.Temperature of the participants……………………………………

29.Has your child suffered from any health conditions in the last 15 days?

…………………………………………………………………………………………………………………………………………………………………………………………

30.Does the child have a Road to Health Booklet (RtHB)?

| Yes | No |
| --- | --- |

31. Has the child completed all immunisations?

| Yes | No |
| --- | --- |

Comment ………………………………………………………………………………..

32 Blood sample taken:

| Yes | No |
| --- | --- |

- 1. Time of blood collection………………………………
  2. Time of the previous meal……………………………

**3.3 Morbidity checklist**

| **Diseases** | **Mark with X** |
| --- | --- |
| **Diseases of the respiratory system** |  |
| Flu |  |
| Throat problems |  |
| Asthma/bronchitis |  |
| Pneumonia |  |
| **Signs and symptoms, not elsewhere classified** |  |
| Stomachache |  |
| fever |  |
| Headache |  |
| vomiting |  |
| **Infections and parasitic diseases** |  |
| Diarrhea |  |
| Chickenpox |  |
| Worm infections |  |
| Hepatitis |  |
| **Diseases of the ear** |  |
| Earache |  |
| Ear discharge |  |
| Ear problem/inflammation |  |
| **Diseases of the digestive system** |  |
| Toothache |  |
| Gastrointestinal problem/stomach ache and nausea |  |
| Mouth sores or disease |  |
| Hernia |  |
| Appendicitis |  |
| **Diseases of the skin** |  |
| Wound |  |
| Allergy |  |
| Tumor or abscess |  |
| Itching |  |
|  |  |

**APPENDIX C: 24-hour recall**

| **Section: Dietary Patterns and Intakes of children** |
| --- |

**Please describe the foods (meals and snacks) that you ate yesterday during the day and night, whether at home or outside the home. Start with the first food eaten in the morning**

*Write down all food and drinks mentioned by the respondent. When the respondent has finished probe for meals and snacks not mentioned*

| **Meal** | **Time of meal** | **Food item description** |
| --- | --- | --- |
| **Breakfast** |  |  |
| **Snack** |  |  |
| **Lunch** |  |  |
| **Snack** |  |  |
| **Dinner** |  |  |
| **Snack** |  |  |

**[Household level: consider foods eaten by the child, and exclude foods purchased and eaten outside of the home or at the preschool]**

*When the respondent recall is complete, fill in the food groups based on the information recorded above. For any food groups not mentioned, ask the respondent if a food item from this group was consumed.*

| Question  number | Food group | Examples | Yes = 1  No = 0 |
| --- | --- | --- | --- |
|  | CEREALS | corn/maize, rice, wheat, sorghum, millet or any other grains or foods made from these (e.g. bread, noodles, porridge or other grain products) + *insert local foods e.g.* *ugali, nshima, porridge or pastes or other locally* *available grains* |  |
|  | VITAMIN A RICH VEGETABLES AND TUBERS | pumpkin, carrots, squash, or sweet potatoes that are orange inside + *other locally available vitamin-A rich* *vegetables (e.g. red sweet pepper)* |  |
|  | WHITE TUBERS AND ROOTS | white potatoes, white yams, white cassava, or other foods made from roots |  |
|  | DARK GREEN LEAFY VEGETABLES | dark green/leafy vegetables, including wild ones + *locally available vitamin-A rich leaves such as amaranth, cassava leaves, kale, spinach etc.* |  |
|  | OTHER VEGETABLES | other vegetables (e.g. tomato, onion, eggplant),  including wild vegetables |  |
|  | VITAMIN A RICH FRUITS | ripe mangoes, cantaloupe, apricots (fresh or dried), ripe papaya, dried peaches + *other locally available vitamin* *A-rich fruits* |  |
|  | OTHER FRUITS | other fruits, including wild fruits |  |
|  | ORGAN MEAT (IRON RICH) | liver, kidney, heart or other organ meats or blood-based foods |  |
|  | FLESH MEATS | beef, pork, lamb, goat, rabbit, wild game, chicken, duck, or other birds |  |
|  | EGGS | chicken, duck, guinea hen or any other egg |  |
|  | FISH | fresh or dried fish or shellfish |  |
|  | LEGUMES, NUTS AND SEEDS | beans, peas, lentils, nuts, seeds or foods made from  these |  |
|  | MILK AND MILK PRODUCTS | milk, cheese, yogurt or other milk products |  |
|  | OILS AND FATS | oil, fats or butter added to food or used for cooking |  |
|  | RED PALM PRODUCTS | Red palm oil, palm nut or palm nut pulp sauce |  |
|  | SWEETS | sugar, honey, sweetened soda or sugary foods such as chocolates, candies, cookies and cakes |  |
|  | SPICES, CONDIMENTS, BEVERAGES | spices (black pepper, salt), condiments (soy sauce, hot sauce), coffee, tea, alcoholic beverages OR *local*  *examples* |  |
| 47. Total yes: | | | Yes = 1  No = 0 |
| Individual level only | Did you eat anything (meal or snack) OUTSIDE of the home yesterday? | |  |
| Household level only | Did you or anyone in your household eat anything (meal or snack) OUTSIDE of the home yesterday? | |  |

**APPENDIX: D**

**SELECTED FOOD FREQUENCY QUESTIONNAIRE**

|  |  | **Average use** | | | | | | | |
| --- | --- | --- | --- | --- | --- | --- | --- | --- | --- |
| **Code** | **Food items** | **<1 month** | **1-3**  **Month** | **1 week** | **2-4**  **week** | **5-6**  **week** | **1 day** | **2-3**  **day** | **4+**  **day** |
| **Starchy food** | | | | | | | | | |
| **50** | Stiff MM porridge |  |  |  |  |  |  |  |  |
| **51** | Soft MM porridge |  |  |  |  |  |  |  |  |
| **52** | Fermented Soft MM porridge |  |  |  |  |  |  |  |  |
| **53** | Fermented Stiff MM porridge |  |  |  |  |  |  |  |  |
| **54** | Fermented home-made soft porridge |  |  |  |  |  |  |  |  |
| **55** | Samp |  |  |  |  |  |  |  |  |
|  | Rice |  |  |  |  |  |  |  |  |
|  | Macaroni |  |  |  |  |  |  |  |  |
|  | Spaghetti |  |  |  |  |  |  |  |  |
|  | Cooked maize cob |  |  |  |  |  |  |  |  |
|  | Roasted maize cob |  |  |  |  |  |  |  |  |
|  | Oats porridge |  |  |  |  |  |  |  |  |
|  | Muesli |  |  |  |  |  |  |  |  |
|  | Cornflakes |  |  |  |  |  |  |  |  |
|  | All bran |  |  |  |  |  |  |  |  |
|  | Brown Bread |  |  |  |  |  |  |  |  |
|  | Whole wheat bread |  |  |  |  |  |  |  |  |
|  | White bread |  |  |  |  |  |  |  |  |
|  | Home-made bread |  |  |  |  |  |  |  |  |
|  | Dumpling |  |  |  |  |  |  |  |  |
|  | Fat-cakes |  |  |  |  |  |  |  |  |
|  | Scones |  |  |  |  |  |  |  |  |
|  | Cakes |  |  |  |  |  |  |  |  |
|  | Muffins |  |  |  |  |  |  |  |  |
|  | Biscuits |  |  |  |  |  |  |  |  |
| **Protein foods (Meat and meat product)** | | | | | | | | | |
|  | Chicken drumstick (F/S) |  |  |  |  |  |  |  |  |
|  | Chicken thigh (F/S) |  |  |  |  |  |  |  |  |
|  | Chicken breast (F/S) |  |  |  |  |  |  |  |  |
|  | Chicken wing (F/S) |  |  |  |  |  |  |  |  |
|  | Stewed chicken feet |  |  |  |  |  |  |  |  |
|  | Chicken heads |  |  |  |  |  |  |  |  |
|  | Chicken liver |  |  |  |  |  |  |  |  |
|  | Chicken gizzard |  |  |  |  |  |  |  |  |
|  | Chicken necks |  |  |  |  |  |  |  |  |
|  | Chicken giblets |  |  |  |  |  |  |  |  |
|  | Stewed chicken |  |  |  |  |  |  |  |  |
|  | Barbequed/grilled chicken |  |  |  |  |  |  |  |  |
|  | Braai or dusty chicken |  |  |  |  |  |  |  |  |
|  | Stewed Turkey |  |  |  |  |  |  |  |  |
|  | Beef stew |  |  |  |  |  |  |  |  |
|  | Braai beef |  |  |  |  |  |  |  |  |
|  | minced meat |  |  |  |  |  |  |  |  |
|  | Ox Tripe |  |  |  |  |  |  |  |  |
|  | Ox Liver |  |  |  |  |  |  |  |  |
|  | Ox tail |  |  |  |  |  |  |  |  |
|  | Canned beef |  |  |  |  |  |  |  |  |
|  | Lamb/mutton stew |  |  |  |  |  |  |  |  |
|  | Pork stew |  |  |  |  |  |  |  |  |
|  | Grilled pork |  |  |  |  |  |  |  |  |
|  | Goat stew |  |  |  |  |  |  |  |  |
|  | Polony |  |  |  |  |  |  |  |  |
|  | Russian |  |  |  |  |  |  |  |  |
|  | Vienna |  |  |  |  |  |  |  |  |
|  | Bacon |  |  |  |  |  |  |  |  |
|  | Wors |  |  |  |  |  |  |  |  |
|  | Canned Fish |  |  |  |  |  |  |  |  |
|  | Fried fish |  |  |  |  |  |  |  |  |
|  | Steamed/boiled fish |  |  |  |  |  |  |  |  |
|  | Crumbed fish |  |  |  |  |  |  |  |  |
|  | Dried smoked fish (bak fish) |  |  |  |  |  |  |  |  |
|  | Locust |  |  |  |  |  |  |  |  |
|  | Mopani worms/Matomani |  |  |  |  |  |  |  |  |
|  | Termites |  |  |  |  |  |  |  |  |
|  | Ntshwamakhura/Tintshwa |  |  |  |  |  |  |  |  |
|  | Jengelengende |  |  |  |  |  |  |  |  |
| **Milk and milk product** | | | | | | | | | |
|  | Fresh cow milk |  |  |  |  |  |  |  |  |
|  | Sour cow milk |  |  |  |  |  |  |  |  |
|  | Pasteurised full cream cow’s milk |  |  |  |  |  |  |  |  |
|  | Skimmed/ fat free milk |  |  |  |  |  |  |  |  |
|  | Low fat milk |  |  |  |  |  |  |  |  |
|  | Powdered cow milk (Nespray, Klim) |  |  |  |  |  |  |  |  |
|  | Condensed milk |  |  |  |  |  |  |  |  |
|  | Fresh full cream Yoghurt |  |  |  |  |  |  |  |  |
|  | Low fat, sweetened yoghurt |  |  |  |  |  |  |  |  |
|  | Drinking yoghurt (Yogi sip |  |  |  |  |  |  |  |  |
|  | Coffee creamer (cremora, ellis brown etc) |  |  |  |  |  |  |  |  |
|  | Ice-cream |  |  |  |  |  |  |  |  |
|  | Cheese (specify) |  |  |  |  |  |  |  |  |
|  | Boiled Eggs |  |  |  |  |  |  |  |  |
|  | Fried eggs |  |  |  |  |  |  |  |  |
|  | Scrambled eggs |  |  |  |  |  |  |  |  |
|  | Mayo |  |  |  |  |  |  |  |  |
|  | Inkomazi/Maas |  |  |  |  |  |  |  |  |
| **Legumes** | | | | | | | | | |
|  | Baked beans |  |  |  |  |  |  |  |  |
|  | Sugar beans |  |  |  |  |  |  |  |  |
|  | soypeas |  |  |  |  |  |  |  |  |
|  | Kidney beans |  |  |  |  |  |  |  |  |
|  | Chicken peas |  |  |  |  |  |  |  |  |
|  | Boiled Jugo beans |  |  |  |  |  |  |  |  |
|  | Boiled dried cowpeas |  |  |  |  |  |  |  |  |
|  | Boiled fresh cowpeas in pod |  |  |  |  |  |  |  |  |
|  | Stewed cowpeas |  |  |  |  |  |  |  |  |
|  | Butter beans |  |  |  |  |  |  |  |  |
|  | Red Lentils |  |  |  |  |  |  |  |  |
|  | Brown/black lentils |  |  |  |  |  |  |  |  |
|  | Soya beans |  |  |  |  |  |  |  |  |
|  | Boiled fresh groundnuts |  |  |  |  |  |  |  |  |
|  | Roasted groundnuts |  |  |  |  |  |  |  |  |
|  | Soya mince |  |  |  |  |  |  |  |  |
|  | Processed and packaged peanuts |  |  |  |  |  |  |  |  |
|  | Macadamia nuts |  |  |  |  |  |  |  |  |
|  | Pecan nuts |  |  |  |  |  |  |  |  |
|  | Marula nuts |  |  |  |  |  |  |  |  |
|  | Roasted butternuts seeds |  |  |  |  |  |  |  |  |
|  | Canned Peas |  |  |  |  |  |  |  |  |
|  | Frozen or Fresh cooked peas |  |  |  |  |  |  |  |  |
|  | Dried boiled peas |  |  |  |  |  |  |  |  |
|  | Mungbean (Ndodzi) |  |  |  |  |  |  |  |  |
| **Fruits** | | | | | | | | | |
|  | Apple |  |  |  |  |  |  |  |  |
|  | Banana |  |  |  |  |  |  |  |  |
|  | Orange |  |  |  |  |  |  |  |  |
|  | Avocado |  |  |  |  |  |  |  |  |
|  | Granadilla |  |  |  |  |  |  |  |  |
|  | Lemon |  |  |  |  |  |  |  |  |
|  | Litchi |  |  |  |  |  |  |  |  |
|  | plums |  |  |  |  |  |  |  |  |
|  | Pawpaw |  |  |  |  |  |  |  |  |
|  | Grapes |  |  |  |  |  |  |  |  |
|  | Guava |  |  |  |  |  |  |  |  |
|  | Mango |  |  |  |  |  |  |  |  |
|  | Naartjie |  |  |  |  |  |  |  |  |
|  | Peach |  |  |  |  |  |  |  |  |
|  | Pear |  |  |  |  |  |  |  |  |
|  | Pineapple |  |  |  |  |  |  |  |  |
|  | Sugar cane |  |  |  |  |  |  |  |  |
|  | Apricots |  |  |  |  |  |  |  |  |
|  | Watermelon |  |  |  |  |  |  |  |  |
|  | Mudoro |  |  |  |  |  |  |  |  |
|  | Titshuguru (Natal plum) |  |  |  |  |  |  |  |  |
|  | Titsengele (Sour plum) |  |  |  |  |  |  |  |  |
|  | Mapfilwa |  |  |  |  |  |  |  |  |
|  | Makwakwa (Baboon orange) |  |  |  |  |  |  |  |  |
|  | Masala |  |  |  |  |  |  |  |  |
|  | Baobab fruit |  |  |  |  |  |  |  |  |
|  | Fig |  |  |  |  |  |  |  |  |
|  | Tintoma |  |  |  |  |  |  |  |  |
|  | Nombela (African Litchie) |  |  |  |  |  |  |  |  |
|  | Marula fruit |  |  |  |  |  |  |  |  |
|  | Tinyiyi |  |  |  |  |  |  |  |  |
|  | Xirhomberhombe |  |  |  |  |  |  |  |  |
|  | Mobola plum(muvhula/ timbulwa) |  |  |  |  |  |  |  |  |
| **Vegetables** | | | | | | | | | |
|  | Cooked spinach |  |  |  |  |  |  |  |  |
|  | Stewed spinach (tomato, onion) |  |  |  |  |  |  |  |  |
|  | Stewed spinach (with potato) |  |  |  |  |  |  |  |  |
|  | Stewed spinach with nuts |  |  |  |  |  |  |  |  |
|  | Stewed spinach with milk/cream |  |  |  |  |  |  |  |  |
|  | Boiled Potato skin |  |  |  |  |  |  |  |  |
|  | Shallow fried potato |  |  |  |  |  |  |  |  |
|  | Baked potato |  |  |  |  |  |  |  |  |
|  | Mashed potato |  |  |  |  |  |  |  |  |
|  | Potato salad with mayonnaise |  |  |  |  |  |  |  |  |
|  | Boiled White-flesh sweet potato |  |  |  |  |  |  |  |  |
|  | Boiled Yellow-flesh sweet potato |  |  |  |  |  |  |  |  |
|  | Boiled Orange-flesh sweet potato |  |  |  |  |  |  |  |  |
|  | Boiled carrots |  |  |  |  |  |  |  |  |
|  | Stewed carrots |  |  |  |  |  |  |  |  |
|  | Raw carrot |  |  |  |  |  |  |  |  |
|  | Carrot salad |  |  |  |  |  |  |  |  |
|  | Boiled pumpkin leaves |  |  |  |  |  |  |  |  |
|  | Boiled Butternut |  |  |  |  |  |  |  |  |
|  | Butternut cooked with margarine and sugar |  |  |  |  |  |  |  |  |
|  | Boiled mushrooms |  |  |  |  |  |  |  |  |
|  | Stewed Baby marrow |  |  |  |  |  |  |  |  |
|  | Boiled Rhanga |  |  |  |  |  |  |  |  |
|  | Tomato gravy with onion |  |  |  |  |  |  |  |  |
|  | Raw Tomato with salt |  |  |  |  |  |  |  |  |
|  | Tomato salad |  |  |  |  |  |  |  |  |
|  | Beetroot salad |  |  |  |  |  |  |  |  |
|  | Canned/bottled beetroot |  |  |  |  |  |  |  |  |
|  | Stewed beetroot leaves |  |  |  |  |  |  |  |  |
|  | Boiled Cabbage |  |  |  |  |  |  |  |  |
|  | Fried cabbage |  |  |  |  |  |  |  |  |
|  | Stewed cabbage (onion, tomato or potato and oil) |  |  |  |  |  |  |  |  |
|  | Cabbage salad |  |  |  |  |  |  |  |  |
|  | Stewed Cowpea leaves with oil, tomato and onion. |  |  |  |  |  |  |  |  |
|  | Stewed Cowpea leaves with peanuts |  |  |  |  |  |  |  |  |
|  | Dried cowpea  leaves with oil, onion and tomato |  |  |  |  |  |  |  |  |
|  | Dried cowpea leaves with crushed peanuts or peanut butter |  |  |  |  |  |  |  |  |
|  | Stewed Pumpkin leaves with flower and baby marrow with groundnuts |  |  |  |  |  |  |  |  |
|  | Stewed Pumpkin leaves with crushed groundnuts |  |  |  |  |  |  |  |  |
|  | Stewed pumpkin leaves with tomato |  |  |  |  |  |  |  |  |
|  | Stewed dried Pumpkin leaves with flower and groundnut s |  |  |  |  |  |  |  |  |
|  | Stewed Muxiji with onion, tomato and salt |  |  |  |  |  |  |  |  |
|  | Stewed Muxiji with crushed groundnuts or peanut butter |  |  |  |  |  |  |  |  |
|  | Stewed Muxiji with Amaranthus and salt |  |  |  |  |  |  |  |  |
|  | Boiled Cleome gynandra with salt (Bangala) |  |  |  |  |  |  |  |  |
|  | Stewed Cleome with tomato, onion and oil |  |  |  |  |  |  |  |  |
|  | Stewed Cleome gynandra with tomato and onion |  |  |  |  |  |  |  |  |
|  | Boiled Cleome gynandra crushed groundnuts |  |  |  |  |  |  |  |  |
|  | Stewed Dried Cleome and crushed groundnuts |  |  |  |  |  |  |  |  |
|  | Boiled Amaranthus with salt (Vowa)) |  |  |  |  |  |  |  |  |
|  | Stewed Amaranthus with tomato, onion and oil |  |  |  |  |  |  |  |  |
|  | Stewed with Amaranthus with tomato and onion |  |  |  |  |  |  |  |  |
|  | Boiled Amaranthus with crushed groundnuts |  |  |  |  |  |  |  |  |
|  | Stewed Dried Amaranthus and crushed groundnuts |  |  |  |  |  |  |  |  |
|  | Boiled fresh Guxe (Jute plant) |  |  |  |  |  |  |  |  |
|  | Boiled dried Guxe, with *Mormodica balsamina* (Nkanka) |  |  |  |  |  |  |  |  |
|  | Boiled dried Guxe with tomato |  |  |  |  |  |  |  |  |
|  | Boiled Okra leaves |  |  |  |  |  |  |  |  |
|  | Boiled Okra leaves with tomato |  |  |  |  |  |  |  |  |
|  | Boiled okra leaves and fruit |  |  |  |  |  |  |  |  |
|  | Boiled okra fruit with tomatoes |  |  |  |  |  |  |  |  |
|  | Stewed Mormodica leaves with fruits, tomatoes and onions |  |  |  |  |  |  |  |  |
|  | Stewed Mormodica with crushed groundnuts |  |  |  |  |  |  |  |  |
|  | Boiled Chinese spinach |  |  |  |  |  |  |  |  |
|  | Stewed Chinese spinach with tomato, onion and oil |  |  |  |  |  |  |  |  |
|  | Stewed Chinese spinach with crushed groundnut |  |  |  |  |  |  |  |  |
|  | Green pepper |  |  |  |  |  |  |  |  |
|  | Red Pepper |  |  |  |  |  |  |  |  |
|  | Yellow pepper |  |  |  |  |  |  |  |  |
|  | Onion |  |  |  |  |  |  |  |  |
|  | Kale or MuRhodesia (Choumolia- green leafy veg |  |  |  |  |  |  |  |  |
|  | Fried Onion |  |  |  |  |  |  |  |  |
|  | Cucumber |  |  |  |  |  |  |  |  |
|  | Lettuce |  |  |  |  |  |  |  |  |
| **Beverages** | | | | | | | | | |
|  | Water |  |  |  |  |  |  |  |  |
|  | Tea |  |  |  |  |  |  |  |  |
|  | Rooibos |  |  |  |  |  |  |  |  |
|  | Coffee |  |  |  |  |  |  |  |  |
|  | Fruit Juice |  |  |  |  |  |  |  |  |
|  | Squashes (diluted e.g Oros) |  |  |  |  |  |  |  |  |
|  | Cold drinks |  |  |  |  |  |  |  |  |
|  | Fizzy drink |  |  |  |  |  |  |  |  |
|  | Dairy fruit mix |  |  |  |  |  |  |  |  |
|  | Homemade mahewu |  |  |  |  |  |  |  |  |
|  | Home-made Ginger drink (Gemmer) |  |  |  |  |  |  |  |  |
|  | Purchased mageu |  |  |  |  |  |  |  |  |
|  | Flavoured mageu |  |  |  |  |  |  |  |  |
|  |  |  |  |  |  |  |  |  |  |
|  |  |  |  |  |  |  |  |  |  |
|  |  |  |  |  |  |  |  |  |  |

**Appendix: E**

| **PLATE WASTE OBSERVATION FORM** |
| --- |

**Child’s ID: ………………………….**

**Child’s Name: …………………….**

**Pre-school Name: …………………………** **Date: ………………………………**

| ***Cucurbita moschata (butternuts) seeds*** | | | |
| --- | --- | --- | --- |
| Children: M/F | | | |
| Day of the week: | | | |
| Amount served (g) | Start time of feeding | End time of feeding | Amount wasted (g) |
|  |  |  |  |
|  |  |  |  |
|  |  |  |  |
|  |  |  |  |

**Appendix F: Food Diary**

**Please describe the foods (meals and snacks) that your child ate yesterday during the day and night at home. Record this once a week during the six months.**

*Month:*

*Day of the week:*

| **Meal** | **Time of meal** | **Food item description** |
| --- | --- | --- |
| **Breakfast** |  |  |
| **Snack** |  |  |
| **Lunch** |  |  |
| **Snack** |  |  |
| **Dinner** |  |  |
| **Snack** |  |  |

**[Each caregiver will be given 35 forms bound to cover 4 days x 6 moths. These will be collected at the end of each month by the field workers]**

**APPENDIX G**

| **Permission letter for Principal** |
| --- |

TO: DEPARTMENT OF SOCIAL DEVELOPEMNT

PROVINCIAL OFFICE

POLOKWANE

0700

FROM: SELEKANE ANANIAS MOTADI

DATE: 12 APRIL 201*11*

Dear Sir/Madam

**RE: PERMISSION TO USE PRE-SCHOOLS FOR RESEARCH**

I Selekane Ananias Motadi, a registered PhD student in Nutritional Sciences at the Division of Human Nutrition, Faculty of Medicine and Health Sciences at Stellenbosch University. With regard to the abovementioned matter, the department of Health Research Ethics Committee would like to request your office to grant Mr Motadi SA (**17640954**) permission to use pre-school children as participants for his PhD project. The title of the project is “***Efficacy of Cucurbita moschata (Butternut) seeds consumption in the management of iron and zinc deficiency in preschool children aged 3-5 years in the Vhembe district, South Africa****”* and the project no is

The study will be conducted amongst pre-school children during the stipulated period in Vhembe district. The study is divided into five phases of which only three (phase 3 ,4 and 5) will be implemented for the children who will be selected in this pre-school. Participants profiling by the research team will follow this. A questionnaire will be used to gather the information from parent and guardian. Parents/guardian will answer the questions related to demographics, nutrient intake, dietary pattern and children will answer questions such as nutritional status and biochemical test. A record sheet will be used for demographics, anthropometric, biochemical test while questionnaires will be used for dietary patterns and nutrient intake. The information mentioned above will be collected from parents/guardian and children during phase 3 and 5. Subsequently, during phase 4 children will be given prepared *Cucurbita moschata* seeds dishes as part of their daily meal at the pre-school.

The study has been approved by Health Research Ethics Committee from Stellenbosch University, Research Ethics Committee from University of Venda, and the Department of Social Development. Therefore, I have enclosed a copy of the research study protocol as well as the approval letters from these units at your perusal.

For more information regarding the project please contact Mr SA Motadi at 015 962 *11*6*11*5/0797455449 or selekane.motadi@univen.ac.za.

…………………………………………………….

Student (Motadi SA)

……………………………………………………

Supervisor (XG Mbhenyane)

| **Appendix H:** Parents of participants information and consent leaflet |
| --- |

TITLE OF THE RESEARCH PROJECT:

Efficacy of consumption of *Cucurbita moschata* seeds in management of zinc and iron status in preschool children in Vhembe district, Limpopo province.

**REFERENCE NUMBER:**

**PRINCIPAL INVESTIGATOR:**

Motadi Selekane Ananias

**ADDRESS:**

Department of Human Nutrition

University of Venda

Po Box 5050, Thohoyandou

**CONTACT NUMBERS:**

0797455449/067070*11*030

**Office hours**: 015 962 *11*6*11*3

Dear Parent/ Legal guardian

My name is Selekane Ananias Motadi and I am a PhD nutritional sciences student at Stellenbosch University. I humbly request your child to participate in my research project. Please read the information presented here, which explain the details of project and feel free to contact me if you require further clarification or explanation of any characteristic of the project. Similarly, the participation of your child is voluntary. If you decline that your child should not participate in the study, this will not affect your child negatively in anyway or whatsoever. You are free to withdraw your child from the study at any point in time, even if you approve that your child should take part. This study has been approved by the Health Research Ethics Committee (HREC) at Stellenbosch University and will be conducted according to principles of the Declaration of Helsinki (2013), Good Clinical Practice and the laws, National and international ethical guidelines of South Africa.

## **What is this research study all about?**

The aim of this study is to test efficacy of consumption of *Cucurbita moschata* (Butternut) seeds in improving zinc and iron status in preschool children (with low serum levels) in the Vhembe district, Limpopo province. The study will be conducted in preschools in Vhembe district among children aged 3-5 years of age. children identified as having severe micronutrient deficiencies will be referred to appropriate health professional or service to address the problem.

You will be expected to answer questions regarding food intake and dietary patterns of the child. The research team will also take the following measurements weight, height, and MUAC and draw blood. Your child will be given lessons patterning to the benefit of consuming *Cucurbita moschata* (butternuts) seeds. Four dishes will be prepared from *Cucurbita moschata* and will be provided as part of your child’s school feeding.

## **Why has your child been invited to participate?**

Your child has been invited to participate because he/she is attending preschool at selected preschools in Vhembe district

## **Will you or your child benefit from taking part in this research?**

A possible benefit could be that children with low serum levels of iron and zinc could be identified. The parents/guardian will be informed and be referred to the local clinic. The study will help identify neglected nutritious local foods for suitable for children.

## **Are there in risks involved in your taking part in this research?**

5ml of blood will be drawn from your child to assess the status of micronutrients (iron and zinc). Qualified phlebotomists will perform this task to minimise the risks. Only two attempts will be made to draw blood from your child’ body. If in the second attempt the Phlebotomists could not succeed in drawing the blood from the child, the process will be stopped.

## **Who will have access to your medical records?**

The information provided by you and your child will be accessed by the research team and will be treated with confidentiality. The information will be used for the purpose of research. The name of your child will be required for referral if in the process of the research your child is diagnosed with a particular nutrition related health risk or disease. During reporting code will be used instead of the child’s name.

## **What will happen in the unlikely event of some form injury occurring as a direct result of your child taking part in this research study?**

There will be a minimal risk of needle-prick injury: *taking of blood can be uncomfortable and may leave a burning sensation for a short while*.

Will you be paid to take part in this study and are there any costs involved?

Travel, time and inconvenience will be reimbursed for the caregivers for the two days that they will be interviewed at the preschool. The preschools will receive cooking utensil (such as pots), and anthropometric measuring equipment used during the study, and educational toys as part of the compensation for the child’s participation.

Is there anything else that you should know or do?

If you have any further queries you can contact Mr Selekane Ananias Motadi on 079 7455449 or 067070*11*030. If you have any concerns or complaints that have not been sufficiently addressed, you can conduct Health Research Ethics Committee at 021-93*11* 9207. You will receive a copy of this information and consent form for your own records.

### **Declaration by parent/legal guardian of participant**

By signing below, I …………………………………..…………………………….………. agree to allow my child, (name)…………………………………….. to take part in a research study entitled test efficacy of consumption of *Cucurbita moschata* (Butternut) seeds in improving zinc and iron status in preschool children (with low serum levels) in the Vhembe district, Limpopo province.

I declare that:

- I have read the attached information leaflet and it is written in a language with which I am fluent and comfortable.
- I have had a chance to ask questions and all my questions have been adequately answered.
- I understand that taking part in this study is **voluntary** and I have not been pressurized to take part.
- I may choose to leave the study at any time and will not be penalized or prejudiced in any way.
- I may be asked to leave the study before it has finished, if the researcher feels it is in my best interests, or if I do not follow the study plan, as agreed to.

Signed at (*place*) ......................…........…………….. On (*date*) …………....……….. 201*11*.

………………………………

Signature of participant parent/legal guardian Witness

**Declaration by Investigator**

**I ……………………………………………………….declare that:**

- I explained the information in this leaflet
- I encouraged him/her to ask questions and took adequately responded to each one of them
- I am satisfied that he/she understood all research aspects as stipulated
- I will further obtain permission by accent from the child named above

Signed at (*place*) ......................…........…………….. On (*date*) …………....……….. 201….

Signature of investigator
